# Supplementary material for: Direct transfer of tri- and di-fluoroethanol units enabled by radical activation of organosilicon reagents
Source: Nat Commun. 2020 Jun 2;11:2756. doi: 10.1038/s41467-020-16380-9 (PMC7265496; doi:10.1038/s41467-020-16380-9)
Supplement: Supplementary file 1 — Supplementary information [file 41467_2020_16380_MOESM1_ESM.pdf]

SUPPLEMENTARY INFORMATION

Direct Transfer of Tri- and di- fluoroethanol Units Enabled by Radical Activation of Organosilicon Reagents

Chen *et al.*

Supplementary Methods

General information

Chromatography: HaiLang Silica Flash P60 size 40~63 µm (200~300 mesh), TLC: HaiLang silica gel 60 (0.25mm). Visualization of the chromatogram was performed by UV, phosphomolybdic acid and $KMnO_4$ staining. Mass spectra were recorded on Bruker UltiMate3000 & Compact, Thermo ISQ LT, LTQ XL and VELOS pro & ORBITRIP mass spectrometers. ^1H, ^{13}C, ^{19}F were recorded on Bruker 400, Bruker 600 and JNM–ECZ 400 using $CDCl_3$ or DMSO-d6 as solvent. Chemical shift values are reported in ppm with the solvent resonance as the internal standard ($CDCl_3$: δ 7.26 for ^1H, δ 77.16 for ^{13}C). Data are reported as follows: chemical shifts, multiplicity (s = singlet, bs = broad singlet, d = doublet, dd = doublet of doublets, t = triplet, td = triplet of doublets, m = multiplet), coupling constants (Hz), and integration. Infrared spectra were recorded on an Agilent Technologies Cary 630 FTIR and wavelengths are reported in cm^{-1}. Melting point was measured by INESA SGW X–4. All reagents were used as received and solvents were dried and degassed according to standard procedure. If no special description, all reactions were conducted under nitrogen. MnF_3, $MnPO_4$ hydrate and $Mn(OAc)_3$•$2H_2O$ was purchased from Alfa, $Mn(OAc)_2$•$4H_2O$ was purchased from adamas, L(−)-menthol, Epiandrosterone, Cholesterol, Diosgenin for preparation of **7ai, 7aj, 7ak, 7am** were purchased from energy-chemicals and Testosterone, Estrone, (8α)-Estradiol for preparation of **7al, 7ao, 7ap** were purchased from adamas. Vitamin E for preparation of **7n** was purchased from TCI. Other alcohols and ketones were purchased from Bidepharm and Meryer. Cinnamic acids **12a~12l** were purchased from adamas and used without further purification.

Synthesis of α-silyl trifluoroethanols

Synthesis of 1-((dimethyl)(phenyl)silyl)-2,2,2-trifluoroethan-1-ol (1a)

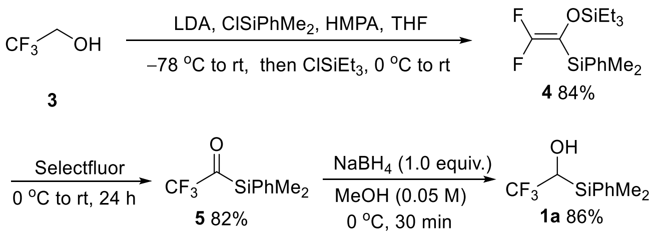

Supplementary Figure. 1 Synthesis of 1-((dimethyl)(phenyl)silyl)-2,2,2-trifluoroethan-1-ol (1a)

1-((Dimethyl)(phenyl)silyl)-2,2,2-trifluoroethan-1-ol was synthesized according to Welch's protocol with slightly variation and modification.[1] To a stirring solution of 2,2,2-trifluoroethanol (6.0 g, 60 mmol) and phenyldimethylchlorosilane (10.2 g, 60 mmol, 1.0 equiv.) and HMPA (6.0 mL) in dry THF (60 mL) in low temperature bath under −78 °C, was added LDA (prepared by Diisopropylamine with *n*-BuLi in THF, 3.5 equiv.) dropwise with syringe. The mixture was kept for 4 h under −78 °C and allowed to rt, after which the mixture was stirred for another 15 h. After addition of triethylchlorosilane (15 mL, 90 mmol, 1.5 equiv.) to the mixture at 0 °C, the mixture was stirred for 4 h, and then quenched by the addition of a saturated aqueous solution of NH_4Cl (20 mL), **4** was isolated by silica gel column chromatography (siliga: 200~300 mesh) using PE as eluent (colorless oil, 16.5 g, 50.4 mmol, 84% yield).

To a solution of Selectfluor (5.4 g, 15 mmol, 1.5 equiv.) in 40 mL of MeCN was added a solution of compound **4** (3.3 g, 10 mmol) in 10 mL of DCM at 0 °C. The resulting mixture was stirred at room temperature for 24 h and quenched by the addition of 20 mL water, then extracted with DCM. The organic layer was dried over Na_2SO_4, filtered and concentrated. Product **5** was purified by either silica gel column chromatography (siliga: 200~300 mesh) using PE as eluent (slight yellow oil, 1.9 g, 8.2 mmol, 82% yield).

To a stirring solution of **5** (0.46 g, 2.0 mmol) in MeOH (0.05 M) at 0 °C was added $NaBH_4$ (0.074 g, 2.0 mmol, 1.0 equiv.) in portions, the resulting mixture was stirred for 0.5 h and quenched by water, then extracted with DCM. The organic layer was dried over Na_2SO_4, filtered, and concentrated, 1-((dimethyl)(phenyl)silyl)-2,2,2-trifluoroethan-1-ol (**1a**) was further purified by silica gel column chromatography (siliga: 200~300 mesh) using PE/EA (50/1, v/v) (colorless oil, 0.40 g, 1.72 mmol, 86% yield).

1-(dimethyl(phenyl)silyl)-2,2,2-trifluoroethan-1-one (5) NMR spectroscopy: ^1H NMR (400 MHz, $CDCl_3$) δ 7.56 (d, J = 6.7 Hz, 2H), 7.49–7.40 (m, 3H), 0.65 (s, 6H); ^{13}C NMR (100 MHz, $CDCl_3$) δ 222.4 (q, J = 36.4 Hz), 134.3, 131.2, 130.9, 128.5, 116.0 (q, J = 294.7 Hz), –5.0; ^{19}F NMR (375 MHz, $CDCl_3$) δ –79.4 (s, 3F). IR (ATR): 3075, 1689, 1431, 1271, 1193, 1133, 738, 697 cm^{-1}. HRMS (EI+, m/z): calcd for $C_{10}H_{11}F_3OSi^+$ $(M)^+$: 232.0531; Found: 232.0533.

1-((Dimethyl)(phenyl)silyl)-2,2,2-trifluoroethanol (1a) NMR spectroscopy: ^1H NMR (400 MHz, $CDCl_3$) δ 7.61 (d, J = 6.1 Hz, 2H), 7.47–7.39 (m, 3H), 3.84 (q, J = 11.1 Hz, 1H), 0.49 (d, J = 3.1 Hz, 6H); ^{13}C NMR (100 MHz, $CDCl_3$) δ 134.4, 134.2, 130.3, 128.2, 127.1 (q, J = 278.4 Hz), 65.3 (q, J = 33.1 Hz), –4.7, –5.4; ^{19}F NMR (375 MHz, $CDCl_3$) δ –70.6 (d, J = 8.9 Hz, 3F). IR (ATR): 3441, 2963, 2919, 1428, 1253, 1148, 1085, 1044, 738, 701 cm^{-1}. HRMS (ESI, m/z): calcd for $C_{10}H_{13}F_3NaO^+$ $(M+Na)^+$: 257.0580; Found: 257.0570.

Synthesis of 1-((methyl)(diphenyl)silyl)-2,2,2-trifluoroethan-1-ol (1b)

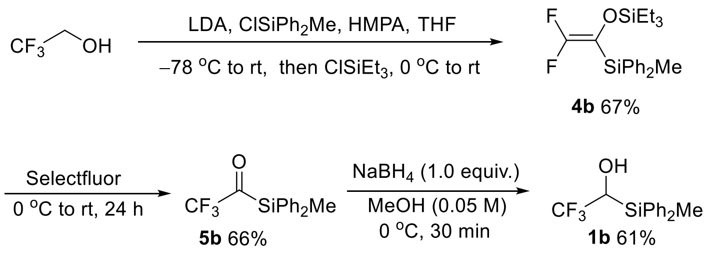

Supplementary Figure. 2 Synthesis of 1-((methyl)(diphenyl)silyl)-2,2,2-trifluoroethan-1-ol (1b)

1-((Diphenyl)(methyl)silyl)-2,2,2-trifluoroethan-1-ol was synthesized according to Welch's protocol with slightly variation and modification.[1] To a stirring solution of 2,2,2-trifluoroethanol (3.0 g, 30 mmol) and methyldiphenylchlorosilane (6.96 g, 30 mmol, 1.0 equiv.) and HMPA (3 mL) in dry THF (30 mL) in low temperature bath under –78 °C, was added LDA (2.0 M in THF, 3.5 equiv.) dropwise with syringe, the mixture was kept for 4 h under –78 °C and allowed to RT, after which the mixture was stirred for 15 h. After addition of triethylchlorosilane (7.5 mL, 45 mmol, 1.5 equiv.) to the mixture under 0 °C, the resulting mixture was stirred for 4 h and then quenched by the addition of a saturated aqueous solution of NH_4Cl (20 mL), compound **4b** was coarsely isolated by silica gel column chromatography (siliga: 200~300 mesh) using PE as eluent (colorless oil, 7.9 g, 20.1 mmol, 67% yield).

To a solution of Selectfluor (5.4 g, 15 mmol, 1.5 equiv.) in 40 mL of MeCN was added a solution of compound **4b** (3.9 g, 10 mmol) in 10 mL of DCM under 0 °C. The resulting mixture was stirred at room temperature for 24 h and quenched by the addition of 20 mL water, then extracted with DCM. The organic layer was dried over Na_2SO_4, filtered, and concentrated. Product **5b** was purified by silica gel column chromatography (siliga: 200~300 mesh) using PE as eluent (slight yellow oil, 1.0 g, 3.3 mmol, 66% yield).

To a stirring solution of **5b** (0.58 g, 2.0 mmol) in MeOH (0.05 M) under 0 °C was added $NaBH_4$ (0.074 g, 2.0 mmol, 1.0 equiv.) in portions, the resulting mixture was stirred for 0.5 h and quenched by water, then extracted with DCM. The organic layer was dried over Na_2SO_4, filtered, and concentrated, 1-((Diphenyl)(methyl)silyl)-2,2,2-trifluoroethan-1-ol (**1b**) was further purified by silica gel column chromatography (siliga: 200~300 mesh) using PE/EA (50/1, v/v) (colorless oil, 0.35 g, 1.2 mmol, 61% yield).

Trifluoroacetyldiphenylmethylsilane (5b) NMR spectroscopy: ^{1}H NMR (400 MHz, $CDCl_3$) δ 7.64 (d, J = 6.7 Hz, 4H), 7.53 (t, J = 7.2 Hz, 2H), 7.46 (t, J = 7.2 Hz, 4H), 0.97 (s, 3H); ^{13}C NMR (100 MHz, $CDCl_3$) δ 221.1 (q, J = 34.8 Hz), 135.2, 131.1, 129.9, 128.6, 115.9 (q, J = 295.2 Hz), –5.5; ^{19}F NMR (375 MHz, $CDCl_3$) δ –78.9 (s, 3F). IR (ATR): 3075, 1685, 1431, 1267, 1193, 1137, 731, 697 cm^{-1}. HRMS (ESI, m/z): calcd for $C_{15}H_{13}F_3NaOSi^+$ $(M+Na)^+$: 317.0580; Found: 317.0570.

1-((methyl)(Diphenyl)silyl)-2,2,2-trifluoroethanol (1b) NMR spectroscopy: ^{1}H NMR (400 MHz, $CDCl_3$) δ 7.67–7.64 (m, 4H), 7.48–7.40 (m, 6H), 4.23 (q, J = 11.1 Hz, 1H), 0.78 (s, 3H), 2.00 (s, 1H); ^{13}C NMR (100 MHz, $CDCl_3$) δ 135.5, 135.0, 132.8, 132.0, 130.5, 130.4, 128.3, 128.3, 126.8 (q, J = 278.6 Hz), 64.9 (q, J = 32.8 Hz), –5.9; ^{19}F NMR (375 MHz, $CDCl_3$) δ –69.8 (d, J = 11.9 Hz, 3F). IR (ATR): 3429, 2919, 1428, 1256, 1152, 1085, 1044, 731, 697 cm^{-1}. HRMS (ESI, m/z): calcd for $C_{15}H_{15}F_3NaOSi^+$ $(M+Na)^+$: 319.0736; Found: 319.0739.

Synthesis of 2,2,2-trifluoro-1-((triethyl)silyl)ethan-1-ol (1c)

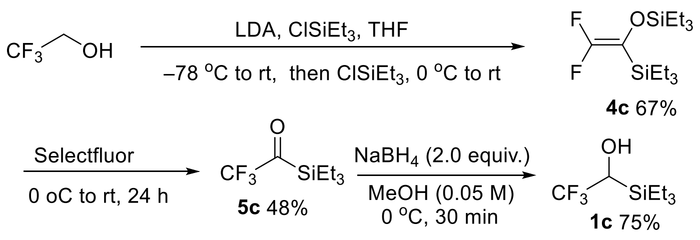

Supplementary Figure. 3 Synthesis of 2,2,2-trifluoro-1-((triethyl)silyl)ethan-1-ol (1c)

2,2,2-trifluoro-1-((triethyl)silyl)-ethan-1-ol was synthesized according to Welch's protocol with slightly variation and modification.[1] To a stirring solution of 2,2,2-trifluoroethanol (3.0 g, 30 mmol) and triethylchlorosilane (5.0 mL, 30 mmol, 1.0 equiv.) in dry THF (30 mL) in low temperature bath under –78 °C, was added LDA (2.0 M in THF, 3.5 equiv.) dropwise with syringe, the mixture was kept for 2 h under –78 °C and allowed to rt, after which the mixture was stirred for 3 h. After addition of triethylchlorosilane (7.5 mL, 45 mmol, 1.5 equiv.) to the mixture at 0 °C, the resulting mixture was stirred for 4 h and then quenched by the addition of a saturated aqueous solution of NH_4Cl (20 mL), compound **4c** was coarsely isolated with 67% yield (colorless oil, 6.2 g, 20.1 mmol) by silica gel column chromatography using PE as eluent.

To a solution of Selectfluor (2.66 g, 7.5 mmol) in 50 mL of MeCN was added a solution of compound **4c** (5 mmol) in 12.5 mL of DCM at 0 °C. The resulting mixture was stirred at room temperature for 12 h and quenched by the addition of 20 mL water, then extracted with DCM. The organic layer was dried over Na_2SO_4, filtered, and concentrated. Product **5c** was purified by distillation under reduced pressure using cold trap (slight yellow oil, 0.5 g, 2.4 mmol, 48% yield).

To a stirring solution of **5c** (0.42 g, 2.0 mmol) in MeOH (0.05 M) at 0 °C was added $NaBH_4$ (0.148 g, 4.0 mmol, 2.0 equiv.) in portions, the resulting mixture was stirred for 0.5 h and quenched by water, then extracted with DCM. The organic layer was dried over Na_2SO_4, filtered, and concentrated, 2,2,2-trifluoro-1-((triethyl)silylethan-1-ol (**1c**) was further purified by silica gel column chromatography using PE/EA (10/1, v/v) (colorless oil, 0.32 g, 1.5 mmol, 75% yield).

1-((triethyl)silyl)-2,2,2-trifluoroethanol (1c) NMR spectroscopy: ^{1}H NMR (400 MHz, $CDCl_3$) δ 3.79 (q, J = 11.5 Hz, 1H), 1.01 (t, J = 7.8 Hz, 9H), 0.76–0.69 (m, 6H), 1.82 (s, 1H); ^{13}C NMR (100 MHz, $CDCl_3$) δ 127.5 (q, J = 278.4 Hz), 64.1 (q, J = 33.1 Hz), 7.2, 1.9; ^{19}F NMR (375 MHz, $CDCl_3$) δ –71.0 (d, J = 8.9 Hz, 3F). IR(ATR): 3429, 2960, 2881, 1461, 1264, 1152, 1085, 723, 686 cm^{-1}. HRMS (EI, m/z): calcd for $C_6H_{12}F_3OSi^+$ $(M–Et)^+$: 185.0610; Found: 185.0607.

Synthesis of 1-triphenylsilyl-2,2,2-trifluoroethanol (1d)

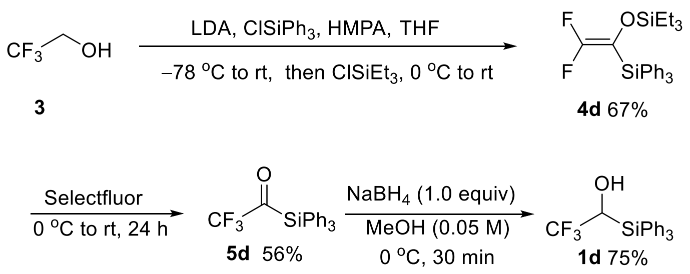

Supplementary Figure. 4 Synthesis of 1-triphenylsilyl-2,2,2-trifluoroethanol (1d)

2,2,2-Trifluoro-1-(triphenylsilyl)-ethan-1-ol was synthesized according to Welch's protocol with slightly variation and modification.[1] To a stirring solution of 2,2,2-trifluoroethanol (3.0 g, 30 mmol) and triphenylchlorosilane (8.82 g, 30 mmol, 1.0 equiv.) and HMPA (3 mL) in dry THF (30 mL) in low temperature bath under −78 °C, was added LDA (2.0 M in THF, 3.5 equiv.) dropwise with syringe. The mixture was kept for 4 h under −78 °C and allowed to rt, after which the mixture was stirred for 15 h. After addition of triethylchlorosilane (7.5 mL, 45 mmol, 1.5 equiv.) to the mixture at 0 °C, the mixture was stirred for 4 h, and then quenched by the addition of a saturated solution of NH_4Cl (20 mL), compound **4d** was coarsely isolated by silica gel column chromatography (siliga: 200~300 mesh) using PE as eluent (colorless oil, 9.1 g, 20.1 mmol, 67% yield).

To a solution of Selectfluor (5.40 g, 15 mmol, 1.5 equiv.) in 40 mL of MeCN was added a solution of compound **4d** (4.5 g, 10 mmol) in 10 mL of DCM at 0 °C. The resulting mixture was stirred at room temperature for 12 h and quenched by the addition of water, then extracted with DCM. The organic layer was dried over Na_2SO_4, filtered, and concentrated. Product **5d** was purified by silica gel column chromatography (siliga: 200~300 mesh) using PE as eluent (white solid, 2.0 g, 5.6 mmol, 56% yield).

To a stirring solution of **5d** (0.71 g, 2.0 mmol) in MeOH (0.05 M) at 0 °C was added $NaBH_4$ (0.074 g, 2.0 mmol, 1.0 equiv.) in portions, the resulting mixture was stirred for 0.5 h and quenched by water, then extracted with DCM. The organic layer was dried over Na_2SO_4, filtered, and concentrated, 2,2,2-trifluoro-1-(triphenylsilyl)ethan-1-ol (**1d**) was further purified by silica gel column chromatography (siliga: 200~300 mesh) using PE/EA (50/1, v/v) (white solid, 0.54 g, 1.5 mmol, 75% yield).
1-((Triphenyl)silyl)-2,2,2-trifluoroethanol (1d), white solid, mp: 127 °C–130 °C. NMR spectroscopy: ^{1}H NMR (400 MHz, $CDCl_3$) δ 7.65 (dd, J = 7.9, 1.2 Hz, 6H), 7.52–7.47 (m, 3H), 7.44–7.40 (m, 6H), 4.55 (q, J = 11.1 Hz, 1H), 2.13 (s, 1H); ^{13}C NMR (100 MHz, $CDCl_3$) δ 136.5, 130.9, 130.6, 130.6, 128.3, 126.7 (q, J = 278.4 Hz), 65.4 (q, J = 33.1 Hz); ^{19}F NMR (375 MHz, $CDCl_3$) δ −68.4 (d, J = 11.9 Hz, 3F). IR (ATR): 3470, 2922, 1379, 1260, 1148, 1111, 737, 701 cm^{-1}. HRMS (ESI, m/z): calcd for $C_{20}H_{18}F_3O^+$ $(M+H)^+$: 359.1074; Found: 359.1066.

Synthesis of 1-phenyldimethylsilyl-2,2-difluoroethanol (2a)

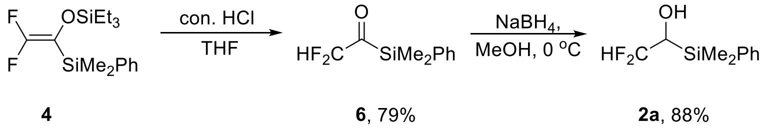

Supplementary Figure. 5 Synthesis of 1-phenyldimethylsilyl-2,2-difluoroethanol (2a)

To a stirring solution of **4** (15.7g, 48 mmol, 1.0 equiv.) in dry THF (10 mL) at 0 °C was added con. HCl (20 mL), the reaction medium was brought to room temperature and stirred for 3.0 h, then extracted with EA. The combined organic phase was washed with brine, dried over Na_2SO_4, filtered, and concentrated. Product **6** was purified by silica gel column chromatography (siliga: 200~300 mesh) using PE as eluent (light yellow oil, 8.1 g, 37.9 mmol, 79% yield).

To a stirring solution of **6** (0.43 g, 2.0 mmol) in MeOH (0.05 M) at 0 ºC was added $NaBH_4$ (0.074 g, 2.0 mmol, 1.0 equiv.) in portions, the resulting mixture was stirred for 0.5 h and quenched by water, then extracted with DCM. The organic layer was dried over Na_2SO_4, filtered, and concentrated, 2,2-difluoro-1-(phenyldimethylsilyl)ethan-1-ol (**2a**) was further purified by silica gel column chromatography (siliga: 200~300 mesh) using PE/EA (50/1, v/v) (colorless oil, 0.38 g, 1.8 mmol, 88% yield).

Difluoroacetylphenyldimethylsilane (6) NMR spectroscopy: ^{1}H NMR (400 MHz, $CDCl_3$) δ 7.57 (d, J = 6.4 Hz, 2H), 7.45–7.39 (m, 3H), 5.39 (t, J = 54.9 Hz, 1H), 0.62 (s, 6H); ^{13}C NMR (100 MHz, $CDCl_3$) δ 233.0 (t, J = 32.0 Hz), 134.3 132.4, 130.5, 128.4, 112.2 (t, J = 249.9 Hz), –4.7; ^{19}F NMR (375 MHz, $CDCl_3$) δ –125.3 (d, J = 53.6 Hz, 3F). IR (ATR): 3071, 2960, 1670, 1428, 1252, 1118, 1044, 828, 787, 697 cm^{-1}. HRMS (ESI, m/z): calcd for $C_{10}H_{13}F_2OSi^+$ $(M+H)^+$: 215.0698; Found: 215.0693.

1-((Phenyl)(dimethyl)silyl)-2,2-difluoroethanol (2a) NMR spectroscopy: ^{1}H NMR (400 MHz, $CDCl_3$) δ 7.62–7.59 (m, 2H), 7.44–7.40 (m, 3H), 5.77 (td, J = 56.4, 4.1 Hz, 1H), 3.70 (ddd, J = 21.5, 14.4, 4.1 Hz, 1H), 0.47 (d, J = 2.8 Hz, 6H); ^{13}C NMR (100 MHz, $CDCl_3$) δ 134.7, 134.3, 130.1, 128.3, 117.7 (t, J = 242.3 Hz), 66.2 (t, J = 24.1 Hz), -5.01, -5.14; ^{19}F NMR (375 MHz, $CDCl_3$) δ –121.1– –123.1 (m, 2F). IR (ATR): 3567, 3422, 3071, 2960, 2915, 1457, 1428, 1379, 1252, 1110, 1059, 1014, 962, 820, 783, 701 cm^{-1}. HRMS (ESI, m/z): calcd for $C_{10}H_{14}F_2NaOSi^+$ $(M+Na)^+$: 239.0674; Found: 239.0668.

Preparation of substituted allylic sulfone.

Synthesis of phenyl-2-acylallylic sulfone

Phenyl-2-acylallilic sulfones (**7b~7i, 7k~7p, 7ah**) were synthesized according to reported procedure [2].

Synthesis of 2-((Phenylsulfonyl)methyl)acrylate

2-((Phenylsulfonyl)methyl)acryloyl chloride was prepared according to reported procedure.[3] 4-bromobut-2-en-1-ol was prepared by reduction of methyl 4-bromocrotonate with DIBAL-H.[4] Estradiol was protected with benzyl group before esterification.[5] Allylic sulfone (**7j**, **7r~7z, 7ae, 7ai~7ap**) were prepared following the general esterification procedure shown below:

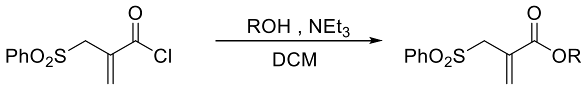

Supplementary Figure. 6 Synthesis of 2-((Phenylsulfonyl)methyl)acrylate

Under N_2 atmosphere, to a solution of corresponding alcohol (4.0 mmol) and triethylamine (505 mg, 5 mmol, 1.25 equiv.) in DCM (10 mL) was added acryl chloride (1220.0 mg, 5 mmol, 1.25 equiv.) in 5 mL DCM at 0 °C. Then the resulting mixture was stirred at room temperature overnight. The reaction was quenched by addition of water (10 mL), and the resulting mixture was extracted three times with DCM (3×30 mL) and the combined organic layer was dried over Na_2SO_4. The solvent was removed under reduced pressure and the residue was purified with column chromatography on silica gel (siliga: 200~300 mesh; PE/EA) to afford the desired product.

4-Bromo-but-2-en-1-yl 2-(((phenyl)sulfonyl)methyl)acrylate (7j)

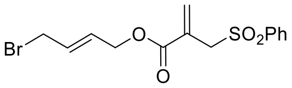

R_f = 0.14 (PE/EA = 4/1, v/v). Colorless oil, (1.0 g, 3.2 mmol, 46% yield). NMR spectroscopy: ^{1}H NMR (400 MHz, $CDCl_3$) δ 7.87–7.85 (m, 2H), 7.65 (t, J = 7.5 Hz, 1H), 7.65–7.53 (m, 2H), 6.54 (s, 1H), 5.95 (s, 1H), 5.88–5.72 (m, 2H), 4.49–4.48 (m, 2H), 4.16 (s, 2H), 4.06–4.04 (m, 2H); ^{13}C NMR (100 MHz, $CDCl_3$) δ 164.5, 138.4, 134.1, 134.0, 130.2,

129.2, 128.9, 127.9, 64.7, 57.6, 43.9. IR (ATR): 2960, 2922, 2855, 1722, 1446, 1260, 1085, 1010, 790, 734 cm^{-1}. HRMS (ESI, m/z): calcd for $C_{14}H_{15}BrO_4NaS^+$ (M+Na)$^+$: 380.9767; Found: 380.9767.

Methoxyprop-2-yl 2-(((phenyl)sulfonyl)methyl)acrylate (7r)

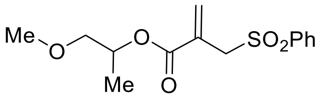

R_f = 0.16 (PE/EA = 4:1, v/v). Colorless oil (1.0 g, 3.3 mmol, 83% yield). NMR spectroscopy:^{1}H NMR (400 MHz, $CDCl_3$) δ 7.85 (d, J = 7.4 Hz, 2H), 7.63 (t, J = 7.4 Hz, 1H), 7.55–7.51 (m, 2H), 6.50 (s, 1H), 5.91 (s, 1H), 5.03–4.82 (m, 1H), 4.23–4.08 (m, 2H), 3.43–3.28 (m, 5H), 1.11 (d, J = 6.5 Hz, 3H); ^{13}C NMR (100 MHz, $CDCl_3$) δ 164.4, 138.4, 134.0, 133.6, 129.2, 129.1, 128.9, 74.8, 70.8, 59.2, 57.4, 16.4. IR (ATR): 2982, 2937, 2885, 1718, 1446, 1308, 1148, 1085, 727 cm^{-1}. HRMS (ESI, m/z): calcd for $C_{14}H_{18}NaO_5S^+$ (M+Na)$^+$: 321.0767; Found: 321.0776.

2,3-Dihydro-1H-inden-1-yl 2-(((phenyl)sulfonyl)methyl)acrylate (7s)

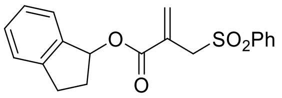

R_f = 0.16 (PE/EA = 8:1, v/v). White solid, mp:70 °C–72 °C, (1.0 g, 2.6 mmol, 87% yield). NMR spectroscopy: ^{1}H NMR (400 MHz, $CDCl_3$) δ 7.84 (d, J = 7.6 Hz, 2H), 7.64–7.48 (m, 3H), 7.32–7.19 (m, 4H), 6.49 (s, 1H), 6.08–6.05 (m, 1H), 5.91 (s, 1H), 4.17 (s, 2H), 3.10–3.03 (m, 1H), 2.90–2.82 (m, 1H), 2.48–2.39 (m, 1H), 1.99–1.91 (m, 1H); ^{13}C NMR (100 MHz, $CDCl_3$) δ 164.9, 144.5, 140.5, 138.5, 133.9, 133.7, 129.2, 129.2, 128.9, 126.9, 125.7, 124.9, 79.8, 57.5, 32.2, 30.3. IR (ATR): 2989, 2937, 1703, 1446, 1293, 1141, 1085, 757, 690 cm^{-1}. HRMS (ESI, m/z): calcd for $C_{19}H_{18}NaO_4S^+$ (M+Na)$^+$: 365.0818; Found: 365.0829.

Benzo[d][1,3]dioxol-5-ylmethyl 2-(((phenyl)sulfonyl)methyl)acrylate (7t)

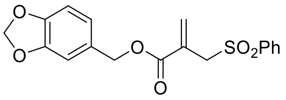

R_f = 0.19 (PE/EA = 4:1, v/v). White solid, mp: 87 °C–90 °C, (1.4 g, 3.9 mmol, 78% yield). NMR spectroscopy: ^{1}H NMR (400 MHz, $CDCl_3$) δ 7.83 (d, J = 7.3 Hz, 2H), 7.62 (t, J = 7.5 Hz, 1H), 7.50 (t, J = 7.7 Hz, 2H), 6.81–6.71 (m, 3H), 6.53 (s, 1H), 5.98 (s, 2H), 5.95 (s, 1H), 4.89 (s, 2H), 4.16 (s, 2H); ^{13}C NMR (100 MHz, $CDCl_3$) δ 164.7, 147.9, 138.3, 134.1, 134.0, 129.2, 129.1, 128.9, 128.9, 122.5, 109.2, 108.4, 101.4, 67.3, 57.5. IR (ATR): 2960, 2926, 1715, 1491, 1256, 1141, 1085, 1036, 798, 731 cm^{-1}. HRMS (ESI, m/z): calcd for $C_{18}H_{16}NaO_6S^+$ (M+Na)$^+$: 383.0560; Found: 383.0570.

2-Idobenzyl 2-(((phenyl)sulfonyl)methyl)acrylate (7u)

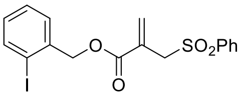

R_f = 0.24 (PE/EA = 4/1, v/v). White solid, mp: 85 °C–86 °C, (1.1 g, 2.6 mmol, 88% yield). NMR spectroscopy: ^{1}H NMR (400 MHz, $CDCl_3$) δ 7.85 (t, J = 6.9 Hz, 3H), 7.60 (t, J = 7.3 Hz, 1H), 7.48 (t, J = 7.6 Hz, 2H), 7.35 (t, J = 7.5 Hz, 1H), 7.28 (d, J = 6.7 Hz, 1H), 7.04 (t, J = 7.0 Hz, 1H), 6.61 (s, 1H), 6.00 (s, 1H), 5.02 (s, 2H), 4.19 (s, 2H); ^{13}C NMR (100 MHz, $CDCl_3$) δ 164.5, 139.7, 137.9, 134.4, 134.0, 130.2, 129.9, 129.2, 128.9, 128.8, 128.5, 98.6, 70.9, 57.5. IR (ATR): 2982, 2933, 1718, 1305, 1148, 1407, 760, 690 cm^{-1}. HRMS (ESI, m/z): calcd for $C_{17}H_{15}INaO_4S^+$ (M+Na)$^+$: 464.9628; Found: 464.9646.

4-Bromobenzyl 2-(((phenyl)sulfonyl)methyl)acrylate (7v)

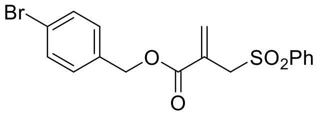

R_f = 0.47 (PE/EA = 2/1, v/v). White solid, mp: 64 °C–66 °C, (1.1 g, 2.7 mmol, 68% yield). NMR spectroscopy: ^{1}H NMR (400 MHz, $CDCl_3$) δ 7.83 (d, J = 7.6 Hz, 2H), 7.61 (t, J = 7.5 Hz, 1H), 7.50–7.47 (m, 4H), 7.16 (d, J = 7.9 Hz, 2H), 6.54 (s, 1H), 5.94 (s, 1H), 4.97 (s, 2H), 4.16 (s, 2H); ^{13}C NMR (100 MHz, $CDCl_3$) δ 164.7, 138.4, 134.4, 134.2, 134.0, 131.9, 130.1, 129.2, 128.9, 128.8, 122.6, 77.5, 77.2, 76.8, 66.6, 57.5. IR (ATR): 2933, 2930, 1715, 1446, 1293, 1337, 1085, 753, 686 cm^{-1}. HRMS (ESI, m/z): calcd for $C_{17}H_{15}BrNaO_5S^+$ (M+Na)$^+$: 416.9767; Found: 416.9783.

Cyclohexylpropyl 2-(((phenyl)sulfonyl)methyl)acrylate (7x)

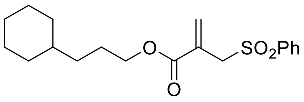

R_f = 0.59 (DCM). White solid, mp: 57 °C–59 °C, (1.2 g, 3.5 mmol, 69% yield). NMR spectroscopy: ^{1}H NMR (400 MHz, $CDCl_3$) δ 7.84 (d, J = 7.4 Hz, 2H), 7.63 (t, J = 7.4 Hz, 1H), 7.52 (t, J = 7.7 Hz, 2H), 6.49 (s, 1H), 5.90 (s, 1H), 4.15 (s, 2H), 3.90 (t, J = 6.8 Hz, 2H), 1.73–1.60 (m, 5H), 1.58–1.47 (m, 2H), 1.27–1.08 (m, 6H), 0.94–0.75 (m, 2H); ^{13}C NMR (100 MHz, $CDCl_3$) δ 164.9, 138.4, 134.0, 133.4, 129.1, 128.8, 66.1, 57.6, 37.3, 33.5, 33.3, 26.7, 26.4, 25.8. IR (ATR): 2930, 2848, 1715, 1472, 1320, 1148, 1085 cm^{-1}. HRMS (ESI, m/z): calcd for $C_{19}H_{26}NaO_4S^+$ (M+Na)$^+$: 373.1444; Found: 373.1450.

Adamantan-2-yl 2-(((phenyl)sulfonyl)methyl)acrylate (7y)

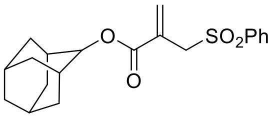

R_f = 0.46 (PE/EA = 4/1, v/v). White solid, mp: 75 °C–78 °C, (1.3 g, 3.5 mmol, 88% yield). NMR spectroscopy: ^{1}H NMR (400 MHz, $CDCl_3$) δ 7.85 (d, J = 7.3 Hz, 2H), 7.62 (t, J = 7.4 Hz, 1H), 7.52 (t, J = 7.7 Hz, 2H), 6.55 (s, 1H), 5.95 (s, 1H), 4.79 (s, 1H), 4.19 (s, 2H), 1.98–1.48 (m, 14H); ^{13}C NMR (100 MHz, $CDCl_3$) δ 164.1, 138.4, 133.9, 133.2, 129.6, 129.1, 128.9, 78.5, 57.4, 37.3, 36.3, 32.0, 31.7, 27.2, 26.9. IR (ATR): 2907, 2855, 1707, 1446, 1297, 1141, 984, 895, 757, 686 cm^{-1}. HRMS (ESI, m/z): calcd for $C_{20}H_{24}NaO_4S^+$ (M+Na)$^+$: 383.1288; Found: 383.1303.

N,N-diphenyl 2-(((phenyl)sulfonyl)methyl)acrylamide (7z)

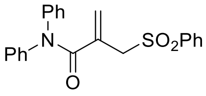

R_f = 0.28 (PE/EA = 2/1, v/v). White solid, mp: 154 °C–155 °C, (0.9 g, 2.4 mmol, 60% yield after recrystallization). NMR spectroscopy: ^{1}H NMR (400 MHz, $CDCl_3$) δ 7.91 (d, J = 7.6 Hz, 2H), 7.64 (t, J = 7.3 Hz, 1H), 7.53 (t, J = 7.6 Hz, 2H), 7.37–7.33 (m, 4H), 7.27–7.24 (m, 6H), 5.69 (s, 1H), 5.33 (s, 1H), 3.84 (s, 2H); ^{13}C NMR (100 MHz, $CDCl_3$) δ 167.7, 143.3, 138.5, 134.0, 133.7, 131.4, 129.5, 129.2, 128.7, 127.6, 127.0, 60.1. IR (ATR): 1651, 1625, 1364, 1305, 1148, 1081, 760, 690 cm^{-1}. HRMS (ESI, m/z): calcd for $C_{22}H_{19}NNaO_3S^+$ (M+Na)$^+$: 400.0978; Found: 400.0991.

Cyclobutyl 2-(((phenyl)sulfonyl)methyl)acrylate (7ae)

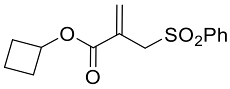

R_f = 0.14 (PE/EA = 8:1, v/v). White solid, mp: 43 °C–45 °C, (0.5 g, 1.7 mmol, 57% yield). NMR spectroscopy: ^{1}H NMR (400 MHz, $CDCl_3$) δ 7.84 (d, J = 7.5 Hz, 2H), 7.64 (t, J = 7.4 Hz, 1H), 7.53 (t, J = 7.7 Hz, 2H), 6.50 (s, 1H), 5.92 (s, 1H), 4.76 (p, J = 7.5 Hz, 1H), 4.14 (s, 2H), 2.30–2.16 (m, 2H), 2.01–1.84 (m, 2H), 1.81–1.68 (m, 1H), 1.63–1.50 (m, 1H); ^{13}C NMR (100 MHz, $CDCl_3$) δ 164.1, 138.4, 134.0, 133.7, 129.2, 129.1, 128.9, 69.9, 57.5, 30.1, 13.5. IR (ATR): 2989, 2937,

1722, 1450, 1323, 1249, 1144, 1070, 753, 686 cm^{-1}. HRMS (ESI, m/z): calcd for $C_{14}H_{18}NaO_4S^+$ (M+Na)$^+$: 303.0662; Found: 303.0668.

(1S,2R,5S)-2-Isopropyl-5-methylcyclohexyl 2-((phenylsulfonyl)methyl)acrylate (7ai)

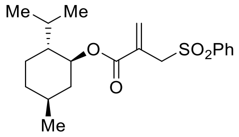

R$_f$ = 0.50 (PE/EA = 4:1, v/v). Colorless oil, (1.2 g, 3.2 mmol, 79% yield). NMR spectroscopy: ^{1}H NMR (400 MHz, CDCl$_3$) δ 7.85 (dt, J = 8.2, 1.6 Hz, 2H), 7.64–7.60 (m, 1H), 7.52 (t, J = 7.5 Hz, 2H), 6.48 (d, J = 0.6 Hz, 1H), 5.96 (d, J = 0.6 Hz, 1H), 4.58–4.52 (m, 1H), 4.17 (s, 2H), 1.77–1.69 (m, 2H), 1.67–1.62 (m, 2H), 1.45–1.33 (m, 2H), 1.05–0.95 (m, 1H), 0.88–0.77 (m, 8H), 0.67 (d, J = 7.0 Hz, 3H); ^{13}C NMR (100 MHz, CDCl$_3$) δ 164.4, 138.5, 134.0, 133.1, 129.4, 129.2, 128.9, 75.8, 57.3, 47.0, 40.6, 34.2, 31.4, 26.5, 23.6, 22.1, 20.8, 16.6. IR (ATR): 2952, 2930, 2870, 1711, 1446, 1312, 1245, 1193, 1144, 1085, 794, 753 cm^{-1}. HRMS (ESI, m/z): calcd for $C_{20}H_{28}NaO_4S^+$ (M+Na)$^+$: 387.1601; Found: 387.1599.

(3S,5S,9S,10R,13S,14S)-5,13-Dimethyl-17-oxohexadecahydro-1H-cyclopenta[a]phenanthren-3-yl 2-((phenylsulfonyl)methyl)acrylate (7aj)

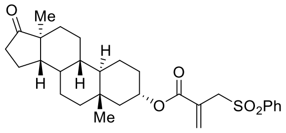

R$_f$ = 0.43 (PE/EA = 2:1, v/v). White solid, mp: 147 ºC–148 ºC, (1.0 g, 2.0 mmol, 90% yield). NMR spectroscopy: ^{1}H NMR (400 MHz, CDCl$_3$) δ 7.87–7.84 (m, 2H), 7.65–7.61 (m, 1H), 7.55–7.51 (m, 2H), 6.48 (d, J = 0.6 Hz, 1H), 5.88 (d, J = 0.6 Hz, 1H), 4.60–4.52 (m, 1H), 4.15 (s, 2H), 2.46–2.40 (m, 1H), 2.11–2.01 (m, 1H), 1.95–1.89 (m, 1H), 1.81–1.77 (m, 2H), 1.74–1.62 (m, 3H), 1.58–1.39 (m, 4H), 1.37–1.13 (m, 7H), 1.02–0.94 (m, 2H), 0.85 (s, 3H), 0.83 (s, 3H), 0.72–0.66 (m, 1H); ^{13}C NMR (100 MHz, CDCl$_3$) δ 221.4, 164.4, 138.6, 134.0, 133.2, 129.5, 129.2, 128.9, 75.0, 57.6, 54.4, 51.5, 47.9, 44.7, 36.7, 36.0, 35.7, 35.1, 33.8, 31.6, 30.9, 28.4, 27.3, 21.9, 20.6, 13.9, 12.4. IR (ATR): 2933, 2851, 1733, 1715, 1305, 1241, 1189, 1141, 1085, 1014, 764, 705 cm^{-1}. HRMS (ESI, m/z): calcd for $C_{29}H_{38}NaO_5S^+$ (M+Na)$^+$: 521.2332; Found: 521.2329.

(8R,9S,10R,13S,14S,17S)-10,13-Dimethyl-3-oxo-2,3,6,7,8,9,10,11,12,13,14,15,16,17-tetradecahydro-1H-cyclo-penta[a]phenanthren-17-yl 2-(((phenyl)sulfonyl)methyl)acrylate (7al)

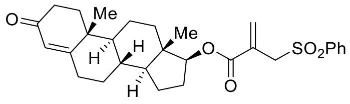

R$_f$ = 0.21 (PE/EA = 2:1, v/v). White solid, mp: 133 ºC–135 ºC, (0.9 g, 2.6 mmol, 66% yield after recrystallization). NMR spectroscopy: ^{1}H NMR (400 MHz, CDCl$_3$) δ 7.85 (d, J = 7.6 Hz, 2H), 7.63 (t, J = 7.3 Hz, 1H), 7.52 (t, J = 7.6 Hz, 2H), 6.48 (s, 1H), 5.86 (s, 1H), 5.72 (s, 1H), 4.49 (t, J = 8.1 Hz, 1H), 4.19–4.10 (m, 2H), 2.41–2.25 (m, 4H), 2.09–2.00 (m, 2H), 1.85–1.54 (m, 6H), 1.42–1.30 (m, 3H), 1.18 (s, 3H), 1.15–0.89 (m, 4H), 0.83 (s, 3H); ^{13}C NMR (100 MHz, CDCl$_3$) δ 199.5, 170.9, 164.8, 138.5, 133.9, 133.2, 129.3, 129.2, 128.9, 124.1, 83.8, 57.5, 53.8, 50.3, 42.8, 38.7, 36.7, 35.8, 35.5, 34.0, 32.8, 31.6, 27.4, 23.6, 20.6, 17.5, 12.3. IR (ATR): 2956, 2930, 1726, 1659, 1305, 1193, 1156, 898, 708 cm^{-1}. HRMS (ESI, m/z): calcd for $C_{29}H_{36}NaO_5S^+$ (M+Na)$^+$: 519.2176; Found: 519.2195.

(4S,5'R,6aR,6bS,8aS,8bR,9S,10R,11aS,12aS,12bS)-5',6a,8a,9-Tetramethyl-1,3,3',4,4',5,5',6,6a,6b,6',7,8,8a,8b,9,11a,12,12a,12b-icosahydrospiro[naphtho[2',1':4,5]indeno[2,1-b]furan-10,2'-pyran]-4-yl 2-(((phenyl)sulfonyl)methyl)acrylate (7am)

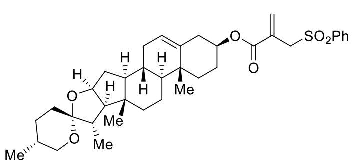

R_f = 0.10 (PE/EA = 10/1, v/v). Slight yellow solid, mp: 197 ºC–199 ºC, (2.0 g, 3.0 mmol, 76% yield after recrystallization). NMR spectroscopy: ^{1}H NMR (400 MHz, $CDCl_3$) δ 7.85 (d, J = 7.3 Hz, 2H), 7.64 (t, J = 7.3 Hz, 1H), 7.53 (t, J = 7.6 Hz, 2H), 6.49 (s, 1H), 5.91 (s, 1H), 5.33–5.29 (m, 1H), 4.49–4.38 (m, 2H), 4.16 (s, 2H), 3.48–3.34 (m, 2H), 2.22–2.16 (m, 2H), 2.01–1.95 (m, 2H), 1.88–1.71 (m, 6H), 1.68–1.57 (m, 5H), 1.53–1.43 (m, 3H), 1.32–1.06 (m, 5H), 1.02–0.91 (m, 7H), 0.78 (s, 6H); ^{13}C NMR (100 MHz, $CDCl_3$) δ 164.3, 139.5, 138.6, 134.0, 133.3, 129.5, 129.2, 129.0, 122.7, 109.4, 80.9, 75.4, 67.0, 62.2, 57.6, 56.5, 50.0, 41.7, 40.4, 39.8, 37.9, 36.9, 36.8, 32.2, 32.0, 31.5, 30.4, 28.9, 27.6, 20.9, 19.5, 17.3, 16.4, 14.7. IR (ATR): 2941, 2855, 1711, 1446, 1331, 1193, 1051, 731, 686 cm^{-1}. HRMS (ESI, m/z): calcd for $C_{37}H_{50}NaO_6S^+$ (M+Na)$^+$: 645.3220; Found: 645.3210.

(R)-2,5,7,8-Tetramethyl-2-((4R,8R)-4,8,12-trimethyltridecyl)chroman-6-yl 2-((phenylsulfonyl)methyl)acrylate (7an)

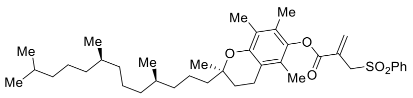

R_f = 0.50 (PE/EA = 4:1, v/v). White solid, mp: 43 ºC–45 ºC, (1.7 g, 2.6 mmol, 88% yield). NMR spectroscopy: ^{1}H NMR (400 MHz, $CDCl_3$) δ 7.92 (d, J = 7.6 Hz, 2H), 7.64 (t, J = 7.3 Hz, 1H), 7.54 (t, J = 7.6 Hz, 2H), 6.84 (s, 1H), 6.20 (s, 1H), 4.30 (s, 2H), 2.55 (t, J = 6.6 Hz, 2H), 2.06 (s, 3H), 1.85 (s, 3H), 1.83 (s, 3H), 1.80–1.71 (m, 2H), 1.52–1.07 (m, 23H), 0.88–0.84 (m, 13H); ^{13}C NMR (100 MHz, $CDCl_3$) δ 163.8, 149.7, 140.4, 138.6, 134.5, 134.1, 129.3, 128.8, 128.4, 126.8, 125.1, 123.3, 117.6, 75.2, 57.1, 39.5, 37.6, 37.4, 32.9, 28.1, 24.9, 24.6, 22.9, 22.8, 21.2, 20.7, 19.9, 19.8, 13.0, 12.1, 11.9. IR (ATR): 2922, 2863, 1733, 1446, 1320.0, 1152, 1103, 753 cm^{-1}. HRMS (ESI, m/z): calcd for $C_{39}H_{58}NaO_5S^+$ (M+Na)$^+$: 661.3897; Found: 661.3906.

(8R,9S,13S,14S)-13-Methyl-17-oxo-7,8,9,11,12,13,14,15,16,17-decahydro-6H-cyclopenta[a]phenanthren-3-yl 2-(((phenyl)sulfonyl)methyl)acrylate (7ao)

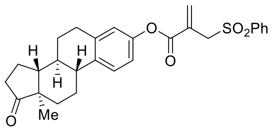

R_f = 0.23 (PE/EA = 2/1, v/v). Slight yellow solid, mp: 203 ºC–205 ºC, (1.5 g, 3.2 mmol, 79% yield). NMR spectroscopy: ^{1}H NMR (400 MHz, $CDCl_3$) δ 7.91 (d, J = 7.6 Hz, 2H), 7.67 (t, J = 7.5 Hz, 1H), 7.56 (t, J = 7.6 Hz, 2H), 7.25 (d, J = 10.4 Hz, 1H), 6.72–6.63 (m, 3H), 6.08 (s, 1H), 4.26 (s, 2H), 2.88–2.86 (m, 2H), 2.54–2.47 (m, 1H), 2.41–2.38 (m, 1H), 2.29–2.27 (m, 1H), 2.17–1.95 (m, 4H), 1.66–1.41 (m, 6H), 0.91 (s, 3H); ^{13}C NMR (100 MHz, $CDCl_3$) δ 220.8, 163.9, 148.4, 138.5, 138.2, 137.8, 135.0, 134.1, 129.3, 129.0, 128.8, 126.5, 121.4, 118.5, 57.7, 50.5, 48.1, 44.3, 38.1, 36.0, 31.7, 29.5, 26.4, 25.9, 21.7, 14.0. IR (ATR): 2933, 2881, 1722, 1495, 1301, 1245, 1133, 760, 686 cm^{-1}. HRMS (ESI, m/z): calcd for $C_{28}H_{30}KO_5S^+$ (M+K)$^+$: 517.1446; Found: 517.1462.

(8R,9S,13S,14S,17S)-3-(Benzyloxy)-13-methyl-7,8,9,11,12,13,14,15,16,17-decahydro-6H-cyclopenta[a]phenanthren-17-yl 2-(((phenyl)sulfonyl)methyl)acrylate (7ap)

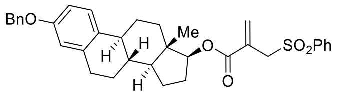

R_f = 0.26 (PE/EA = 4/1, v/v). Slight yellow solid, mp: 127 ºC–128 ºC, (1.9 g, 3.3 mmol, 83% yield). NMR spectroscopy: ^{1}H NMR (400 MHz, $CDCl_3$) δ 7.88 (d, J = 7.9 Hz, 2H), 7.66 (t, J = 7.4 Hz, 1H), 7.55 (t, J = 7.7 Hz, 2H), 7.49–7.29 (m, 5H), 7.21 (d, J = 8.6 Hz, 1H), 6.80 (dd, J = 8.5, 2.3 Hz, 1H), 6.73 (bs, 1H), 6.53 (s, 1H), 5.93 (s, 1H), 5.04 (s, 2H), 4.67–4.50 (m, 1H), 4.19 (q, J = 13.8 Hz, 2H), 2.99–2.76 (m, 2H), 2.35–2.02 (m, 3H), 1.93–1.69 (m, 3H), 1.51–1.22 (m,

7H), 0.83 (s, 3H); ^{13}C NMR (100 MHz, $CDCl_3$) δ 164.8, 156.8, 138.4, 138.0, 137.3, 133.9, 133.3, 132.7, 129.3, 129.2, 128.9, 128.7, 127.9, 127.5, 126.4, 114.9, 112.4, 84.0, 70.0, 57.4, 49.7, 43.8, 43.2, 38.6, 36.9, 29.8, 27.5, 27.3, 26.2, 23.3, 12.3. IR (ATR): 2945, 2848, 1711, 1495, 1312, 1189, 1152, 1085, 727 cm^{-1}. HRMS (ESI, m/z): calcd for $C_{35}H_{38}NaO_5S^+$ $(M+Na)^+$: 593.2332; Found: 593.2349.

3.3 Synthesis of phenyl-2-(tolsulfonyl)allylic sulfone (7ab)

Phenyl-2-(tolsulfonyl)allylic sulfone **7ab** was synthesized according to reported protocol.[6]

Phenyl-2-(tolsulfonyl)allylic sulfone (7ab)

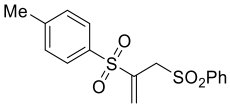

R_f = 0.30 (PE/EA = 2/1). White solid, mp: 125 °C–127 °C, (8.4 mmol, 84% yield for 3 steps from phenyl propargyl sulfide). NMR spectroscopy: ^{1}H NMR (400 MHz, $CDCl_3$) δ 7.77–7.74 (m, 2H), 7.66 (tt, J = 7.5, 1.4 Hz, 1H), 7.60 (dt, J = 8.3, 1.8 Hz, 2H), 7.51 (t, J = 7.8 Hz, 2H), 7.28 (d, J = 7.9 Hz, 2H), 6.64 (d, J = 0.9 Hz, 1H), 6.50 (d, J = 1.2 Hz, 1H), 4.05 (s, 2H), 2.44 (s, 3H); ^{13}C NMR (100 MHz, $CDCl_3$) δ 145.3, 139.8, 137.9, 134.8, 134.4, 130.8, 130.2, 129.4, 128.7, 128.6, 54.3, 21.8. IR (ATR): 2956, 2922, 2855, 1595, 1446, 1312, 1141, 1081, 727 cm^{-1}. HRMS (ESI, m/z): calcd for $C_{16}H_{16}O_4NaS_2^+$ $(M+Na)^+$: 359.0382; Found: 359.0383.

Preparation of ((3-methyl-2-metnylene-3-phenylbutyl)sulfonyl)benzene (7ad)

((3-Methyl-2-metnylene-3-phenylbutyl)sulfonyl)benzene (**7ad**) was prepared according to reported methods.[7,8] Synthetic routine is shown as below:

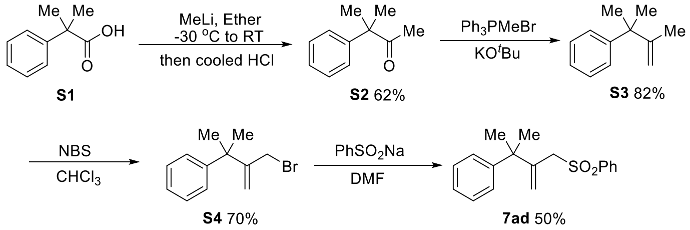

Supplementary Figure 7 Preparation of ((3-methyl-2-metnylene-3-phenylbutyl)sulfonyl)benzene (7ad)

To a solution of 2-methyl-2-phenylpropionic acid (4.92 g, 30 mmol) in diethyl ether at –30 °C was added a solution of methyl lithium (1.6 M, 56.0 mL, 3.0 equiv.) dropwise with syringe pump, after which the resulting mixture was allowed to RT and kept stirring for 1.5 h. The reaction was cooled to 0 °C and poured into iced hydrogen chloride solution, extracted with PE (3×100 mL), the organic phase was combined, concentrated under reduced pressure and purified with column chromatography on silica gel (siliga: 200~300 mesh) and PE as eluent to afford the colorless oil **S2** (3.0 g, 18.6 mmol, 62% yield).

To a solution of Wittig reagent prepared by Ph_3PMeBr (8.92 g, 25 mmol, 2.1 equiv.) and NaO^{t}Bu (2.8 g, 25 mmol, 2.1 equiv.) in THF (70 mL) stirring for 2 h at room temperature in Schlenk tube was added **S2** (1.82 g, 12 mmol) slowly. Then the resulting mixture was stirred at 60 °C. Upon full consumption of **S2** (monitored by TLC), the mixture was cooled to ambient temperature and filtered under reduced pressure, the filtrate was extracted with PE (3×50mL), the organic phase was dried over Na_2SO_4, filtrated and removed the solvent, purification was conducted with column chromatography on silica gel (siliga: 200~300 mesh) and PE as eluent to afford the colorless oil **S3** (1.57 g, 9.84 mmol, 82% yield).

To a 10 mL tube was added **S3** (0.7 g, 4.3 mmol), NBS (0.84 g, 4.73 mmol, 1.1 equiv.) and chloroform (2 M), then the tube was sealed, the resulting mixture was stirred at 100 °C till NBS was completely dissolved, then the mixture was moved to ambient temperature and filtered under reduced pressure, the filtrate was washed with brine, dried over Na_2SO_4, removed solvent and purified with column chromatography on silica gel (siliga: 200~300 mesh) and PE as eluent to

afford the slight yellow oil **S4** (0.72 g, 3.0 mmol, 70% yield).

To a 50 mL round bottom flask was added **S4** (0.7g, 3.0 mmol) and phenylsulfinate (0.98 g, 6.0 mmol) and DMF (0.25 M), the resulting mixture was stirred at 60 ºC for 12 h, then washed with water and brine, dried over Na_2SO_4, purified with column chromatography on silica gel (200~300 mesh) and PE/EA (20/1~5/1,v/v) as eluent to afford the white solid **7ad** (0.45 g, 1.5 mmol, 50% yield).

((3-methyl-2-metnylene-3-phenylbutyl)sulfonyl)benzene (7ad) mp: 47 ºC–49 ºC. NMR spectroscopy: ^{1}H NMR (400 MHz, $CDCl_3$) δ 7.80 (d, J = 7.3 Hz, 2H), 7.62 (t, J = 7.3 Hz, 1H), 7.50 (t, J = 7.6 Hz, 2H), 7.26–7.13 (m, 5H), 5.70 (s, 1H), 5.53 (s, 1H), 3.50 (s, 2H), 1.34 (s, 6H); ^{13}C NMR (100 MHz, $CDCl_3$) δ 146.3, 143.2, 139.6, 133.7, 129.2, 128.6, 128.6, 126.4, 126.2, 116.7, 58.7, 44.7, 27.8. IR (ATR): 2971, 2930, 1305, 1148, 1085, 913, 768, 723, 686 cm^{-1}. HRMS (ESI, m/z): calcd for $C_{18}H_{20}NaO_2S^+$ (M+Na)$^+$: 323.1076; Found: 323.1085.

Preparation of 6-hydroxylhexyl 2-(((phenyl)sulfonyl)methyl)acrylate (7af)

6-Hydroxylhexyl 2-(((phenyl)sulfonyl)methyl)acrylate was prepared according to reported method.[9]

6-Hydroxyhexyl 2-(((phenyl)sulfonyl)methyl)acrylate (7af)

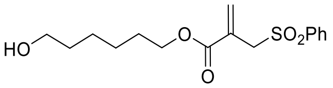

R_f = 0.26 (PE/EA = 1/1, v/v). Colorless oil, (1.8 g, 5.7 mmol, 57% yield). NMR spectroscopy: ^{1}H NMR (400 MHz, $CDCl_3$) δ 7.83 (d, J = 7.6 Hz, 2H), 7.63 (t, J = 7.3 Hz, 1H), 7.52 (t, J = 7.6 Hz, 2H), 6.47 (s, 1H), 5.86 (s, 1H), 4.14 (s, 2H), 3.96 (t, J = 6.7 Hz, 2H), 3.61 (t, J = 6.6 Hz, 2H), 1.75 (s, 1H), 1.55 (t, J = 6.7 Hz, 4H), 1.34–1.31 (m, 4H); ^{13}C NMR (100 MHz, $CDCl_3$) δ 164.9, 138.4, 134.0, 133.4, 129.2, 128.8, 65.6, 62.7, 57.6, 32.6, 28.4, 25.7, 25.4. IR (ATR): 3541, 3422, 2933, 2859, 1715, 1308, 1189, 1148, 1085, 913, 731 cm^{-1}. HRMS (ESI, m/z): calcd for $C_{16}H_{22}NaO_5S^+$ (M+Na)$^+$: 349.1080; Found: 349.1078.

Preparation of 6-oxohexyl 2-(((phenyl)sulfonyl)methyl)acrylate (7ag)

6-Oxohexyl 2-(((phenyl)sulfonyl)methyl)acrylate was prepared via oxidation of **4ax** with DMP (3.0 equiv.) in DCM at ambient temperature.

6-Oxohexyl 2-(((phenyl)sulfonyl)methyl)acrylate (7ag)

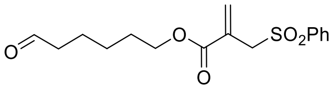

R_f = 0.16 (PE/EA = 2/1, v/v). Colorless oil, (298 mg, 0.92 mmol, 46% yield). NMR spectroscopy: ^{1}H NMR (400 MHz, $CDCl_3$) δ 9.75 (t, J = 1.4 Hz, 1H), 7.84 (d, J = 8.6 Hz, 2H), 7.63 (t, J = 7.5 Hz, 1H), 7.53 (t, J = 7.6 Hz, 2H), 6.47 (s, 1H), 5.86 (s, 1H), 4.14 (s, 2H), 3.97 (t, J = 6.6 Hz, 2H), 2.44 (td, J = 7.3, 1.4 Hz, 2H), 1.66–1.54 (m, 4H), 1.38–1.30 (m, 2H); ^{13}C NMR (100 MHz, $CDCl_3$) δ 202.3, 164.9, 138.5, 134.0, 133.4, 129.2, 129.1, 128.8, 65.3, 57.6, 43.8, 28.3, 25.5, 21.7. IR (ATR): 2937, 2863, 1715, 1446, 1308, 1245, 1185, 1144, 1085, 757 cm^{-1}. HRMS (ESI, m/z): calcd for $C_{16}H_{20}NaO_5S^+$ (M+Na)$^+$: 347.0924; Found: 347.0922.

synthesis of other starting materials

synthesis of acrylamides 10

Acrylamides **10a~10i** was synthesized via condensation of corresponding amine and acyl chloride according to reported reference.[10]

Synthesis of dimethyl 4-(3-(3-methyl-4-hydroxyphenyl)propyl)phthalate 15

Dimethyl 4-(3-(3-methyl-4-hydroxyphenyl)propyl)phthalate **15** was synthesized according to reported reference[11]

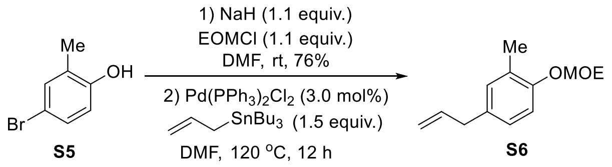

Supplementary Figure 8 Synthesis of S6

Under N_2 atmosphere, to a stirring mixture of NaH (3.5 g, 87 mmol, 1.1 equiv.) in DMF (30 mL) was added a solution of 4-bromo-2-methylphenol **S5** (14.8 g, 79 mmol) in DMF (100 mL) slowly, the resulting mixture was kept stirring for 0.5 h then ethoxymethyl chloride (EOMCl) (9.0 g, 95.7 mmol, 1.1 equiv) was added slowly in 10 min, the reaction medium was stirred for another 5 h and quenched by addition of water, the mixture was extracted with EA (200 mL×3 times), the combined organic phase was washed with water, dried over Na_2SO_4, concentrated under reduced pressure, the residue was purified through a silica plug, a yellow oil was obtained (15.3 g, 60.0 mmol, 76% yield).

The product (7.4 g, 30.0 mmol) was dissolved in DMF (65 mL), to which was added allyltributyltin (14.3 mL, 45.0 mmol, 1.5 equiv.), then the mixture is degassed, then 900 mg of dichlorobis(triphenylphosphino)palladium was added, the resulting mixture was stirred at 120 °C for 10 h. the reaction was quenched by addition of water, and extracted with EA, the combined organic layer was dried over Na_2SO_4, and concentrated under reduced pressure, the residue was purified by chromatography on a 10% wt K_2CO_3-silica column with PE as an eluent.[12] A yellow oil **S6** is obtained (5.0 g, 24 mmol, 81% yield).

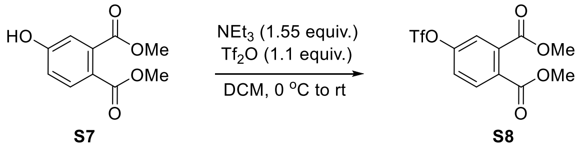

Supplementary Figure 9 Synthesis of S8

Under N_2 atmosphere, to a stirring solution of **S7** (8.4 g, 40 mmol), NEt_3 (8.4 mL, 62mmol, 1.55 equiv.) and DCM (200 mL) was added Tf_2O (11.8 g, 42 mmol, 1.05 equiv.) slowly in 10 min at 0 °C, the resulting mixture was brought to room temperature and stirred for 2 h, and then quenched by addition of water and extracted with DCM (100 mL×3 times), the organic phase was washed with dilute sodium bicarbonate and dried over Na_2SO_4, and concentrated under reduced pressure, the residue was purified by chromatography on a silica column with PE/EA (8/1, v/v) as an eluent. A yellow oil **S8** is obtained (12.3 g, 36.0 mmol, 90% yield).

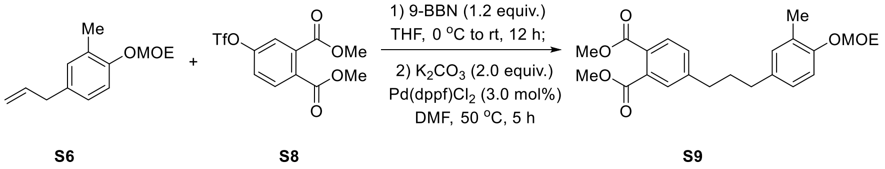

Supplementary Figure 10 Synthesis of S9

Under N_2 atmosphere, 3.5 g (16 mmol) of **S6** was dissolved in 40 mL anhydrous THF, the resulting mixture was cooled to 0 °C, 40.8 mL 9-BBN (0.5 M, 1.3 equiv.) was added, and the medium was brought to room temperature and stirred for 12 h. A solution of **S8** in 70 mL DMF was added, as well as 4.7 g (34 mmol, 2.1 equiv.) of potassium carbonate and $Pd(dppf)Cl_2$ (652.8 mg, 0.8 mmol, 5 mol%), the reaction mixture was degassed, heated to 50 °C for 3 h and quenched with ammonium chloride solution. The mixture was extracted with EA, the organic phase was washed with dilute sodium bicarbonate and dried over Na_2SO_4, and concentrated under reduced pressure, the residue was purified by chromatography on a silica column with PE/EA (8/1, v/v) as an eluent. A colorless oil **S9** is obtained (4.1 g, 10.2 mmol, 64% yield).

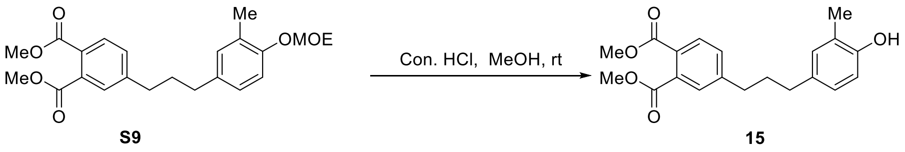

Supplementary Figure 11 Synthesis of 15

Under air atmosphere, to a solution of **S5** (2.9 g, 7.3 mmol) in MeOH (30 mL) was added con. HCl (30 mL) slowly at room temperature, the resulting mixture was stirred for 3 h and monitored by TLC, upon completion, extracted with EA, the organic phase was dried over Na_2SO_4, and concentrated under reduced pressure, the residue was purified by chromatography on a silica column with PE/ EA (4/1, v/v) as an eluent. A colorless oil **15** is obtained (2.3 g, 6.9 mmol, 94% yield).

Investigation of reaction conditions

Investigation of metallic salts

Supplementary Table 1 Investigation of metallic salts

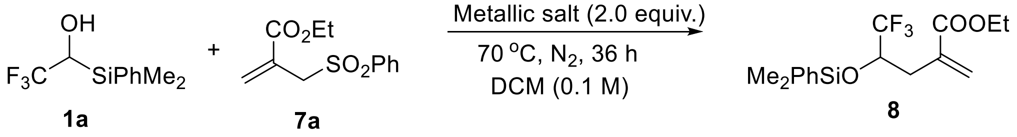

entry	metallic salt	yield /% [b,c]	c.r. /%[c]
1	$Ce(NH_4)(NO_3)_6$	0	100
2	$Ce(SO_4)_2·4H_2O$	0	100
3	$Fe(NO_3)_3·9H_2O$	0	100
4	$Fe_2(SO_4)_3·xH_2O$	0	100
5	$Cu(OAc)_2$	0	6
6	$Co(acac)_3$	0	10
7	--	0	5
8	$Mn(OAc)_3·2H_2O$	68	92
9	MnF_3	0	0
10	$Mn(PO_4)_3$	0	0
11	$Mn(acac)_3$	8	12

a) reaction condition: **1a** (0.1 mmol), **7a** (1.2 equiv.), metallic salt (2.0 equiv.), DCM, 70 ^oC, N_2, 36 h; b) yield by ^{19}F NMR with $PhCF_3$ as internal standard.

Investigation of solvents

Supplementary Table 2 Investigation of solvents

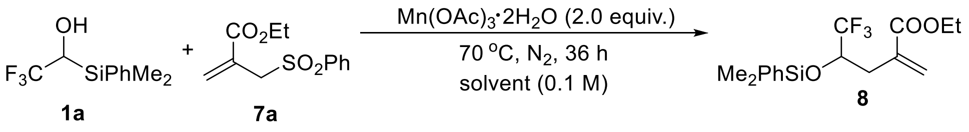

entry	solvent	yield /% [b]	c.r. /%
1	DCM	70 (67)[c]	90 (98)[c]
2	Chloroform	10	82
3	Hexane	47	83
4	Decalin	56	62
5	DMAc	60	84
6	DMF	11	59
7	NMP	10	47
8	MeCN	38	70
9	CyHexane	26	48
10	MCPE	28	50
11	EA	33	65

a) reaction condition: **1a** (0.1 mmol), **7a** (1.2 equiv.), $Mn(OAc)_3 \cdot 2H_2O$ (2.0 equiv.), solvent (0.1 M) ,70 °C, N_2, 36 h; b) yield by ^{19}F NMR with $PhCF_3$ as internal standard; c) repeated reaction. MCPE = methyl cyclopentyl ether.

Investigation of oxidant for catalytic reaction conditions

Supplementary Table 3 Investigation of oxidant for catalytic reaction conditions

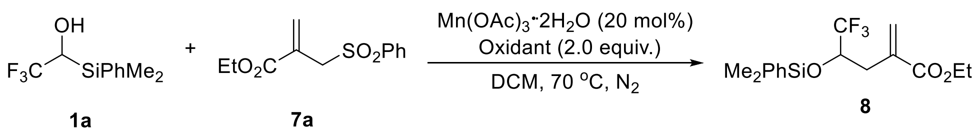

Entry	Oxidant	Yield (%)	c.r (%)
1	WO_3	12	11
2	NCS	32	34
3	TEAPC	--	--
4	*p*-NPO	0	0
5	TBPB	60	75
6[c]	DCP	0	0
7[c]	TBPB	0	0
8[d]	TBPB	39	41
9[e]	TBPB	59	60
10[f]	TBPB	64	81
11	PIDA	8	100
12	PIDTFA	0	100
13	I_2	0	0
14	NBS	16	100
15	MnO_2	14	34
16	$NaBrO_3$	20	55
17	DQ	6	31
18	DMP	0	100

a) reaction condition: **1a** (0.1 mmol), **7a** (1.2 equiv.), $Mn(OAc)_3$ $2H_2O$ (2.0 equiv.), DCM (0.1 M) ,70 °C, N_2, 12 h; b) yield by ^{19}F NMR with TMFB as interior label; c) absence of Mn^{III}; d) 1.0 equiv of TBPB was used; e) 1.5 equiv. of TBPB was used; f) 2.5 equiv. of TBPB was used; g) TEAPC = tetraethylammonium perchlorate, PIDTFA = [Bis(trifluoroacetoxy)iodo]benzene, *p*-NPO = 4-Nitropyridine N-oxide.

Radical inhibition experiments

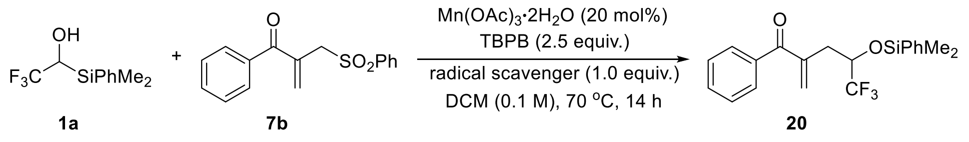

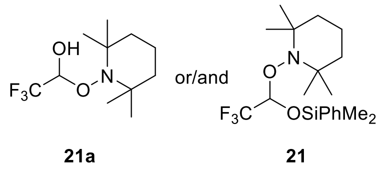

radical scavenger	yield of **20** (%)	recovery of **7b** (%)
TEMPO	0	83
BHT	0	66

Supplementary Figure 12 Radical inhibition experiments

Under N_2 atmosphere, to a dried 10 mL Schlenk tube equipped with a magnetic stir bar containing $Mn(OAc)_3$•$2H_2O$ (16.1 mg, 0.06 mmol, 20 mol%), **7b** (257.4 mg, 0.9 mmol, 3.0 equiv.) and TEMPO (2,2,6,6-tetramethylpiperidine-1-oxyl) (45.7 mg, 0.3 mmol, 1.0 equiv.) or BHT (butylated hydroxytoluene) (66.1 mg, 0.3 mmol, 1.0 equiv.) was added DCM (3.0 mL, 0.1 M), **1a** (70.2 mg, 0.3 mmol) and TBPB (146.0 mg, 0.75 mmol, 2.5 equiv.) sequentially. The tube was sealed, and the resulting mixture was stirred at 70 °C in heating block for 14 h. After the mixture was cooled to ambient temperature, yield of **20** and conversion ratio of **1a** were determined via ^{19}F NMR with $PhCF_3$ as an internal standard. Recovery of **7b** was determined via ^1H NMR with 1,3,5-trimethoxylbenzene as an internal standard. We found reaction was totally inhibited when radical scavenger (TEMPO or BHT) was added to the mixture and compound **21** was detected by HRMS. HRMS (ESI, m/z): calcd for $C_{19}H_{30}F_3NaNO_2Si^+$ $(M+Na)^+$: 412.1890; Found:412.1899; calcd for $C_{19}H_{31}F_3NO_2Si^+$ $(M+H)^+$: 390.2071; Found:390.2059.

Proposed mechanism for allylation, alkylation and alkenylation via radical C-Si bond activation

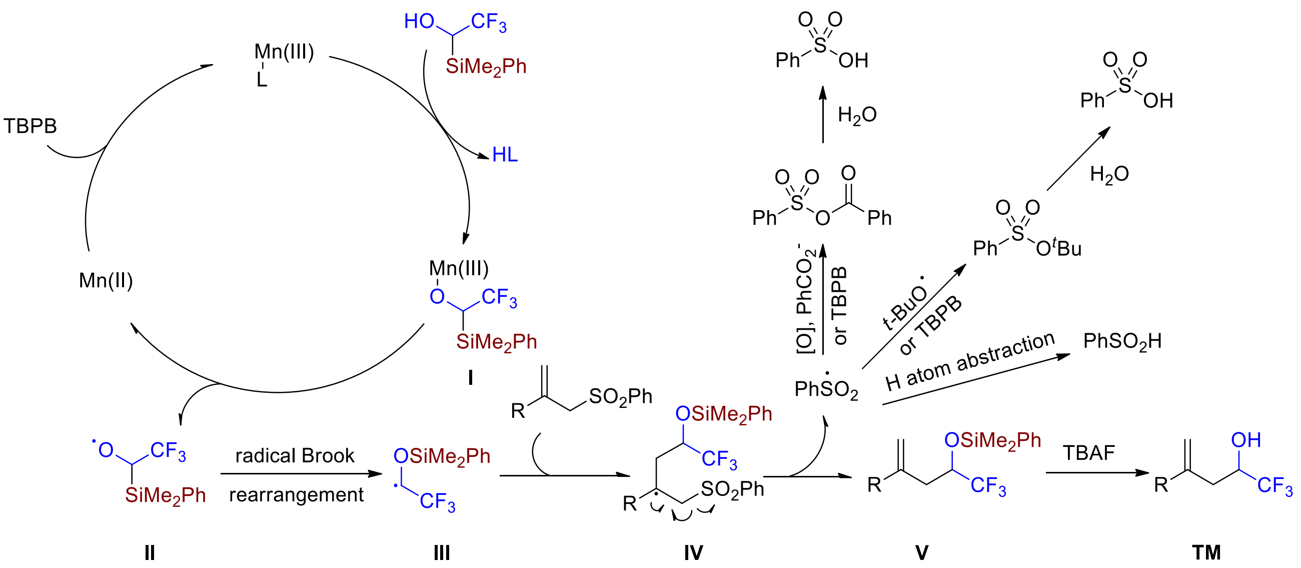

Supplementary Figure 13 Proposed Mechanism for allylation via radical C-Si activation

The radical inhibitation experiments indicate that a radical process might be involved. We found that $Mn(OAc)_3$•$2H_2O$ is able to mediate the reaction without external oxidant, but $Mn(OAc)_2$•$4H_2O$ can not mediate the reaction without TBPB (Table 1, entries 1 and 8 in the manuscript). The HRMS analysis of the reaction mixture of **1a** and **7a** suggests the generation of benzenesulfonyl benzoic anhydride, *tert*-butyl benzenesulfonate, benzesulfonic acid and benzenesulfinic acid as by-products. Based on these experimental results and literature about allylation from allylic sulfones,[13,14] we propose a possible mechanism (Fig. S2). Ligand exchange between Mn(III) species and alcohol **1a** might generate intermediate **I**, which undergoes homolysis to produce alkoxyl radical **II** and Mn(II) intermediate. Carbon radical **III** would be generated through Brook rearrangement, and then undergo radical addition reaction to generate intermediate

IV. Compound **V** would be generated after β-elimination of sulfonyl radical. The alcohol product **TM** would be generated after the desilylation step. Mn(III) catalyst is likely to be regenerated by the oxidation of Mn(II) by TBPB. The sulfonyl radical is likely to be captured by TBPB, generating the side-product benzenesulfonyl benzoic anhydride. The sulfonyl radical might also be oxidized and captured by $PhCO_2^-$ to generate benzenesulfonyl benzoic anhydride. Meanwhile, sulfonyl radical could react with TBPB or *tert*-butoxy radical to form *tert*-butyl benzenesulfonate. Benzenesulfonyl benzoic anhydride and *tert*-butyl benzenesulfonate could be hydrolyzed to generate benzenesulfonic acid. Moreover, the sulfonyl radical could be transformed to sulfinic acid via H atom abstraction reaction under the reaction condition.

Benzenesulfonyl benzoic anhydride: HRMS (ESI, m/z): calcd for $C_{13}H_{10}NaO_4S^+$ $(M+Na)^+$: 285.0192; Found: 285.0195.

tert-Butyl benzenesulfonate: HRMS (ESI, m/z): calcd for $C_{10}H_{14}NaO_3S^+$ $(M+Na)^+$: 237.0556; Found: 237.0561

Benzenesulfinic acid: HRMS (ESI, m/z): calcd for $C_6H_6NaO_2S^+$ $(M+Na)^+$: 164.9981; Found: 164.9975.

Benzenesulfonic acid: HRMS (ESI, m/z): calcd for $C_6H_7O_3S^+$ $(M+H)^+$: 159.0110; Found: 159.0115.

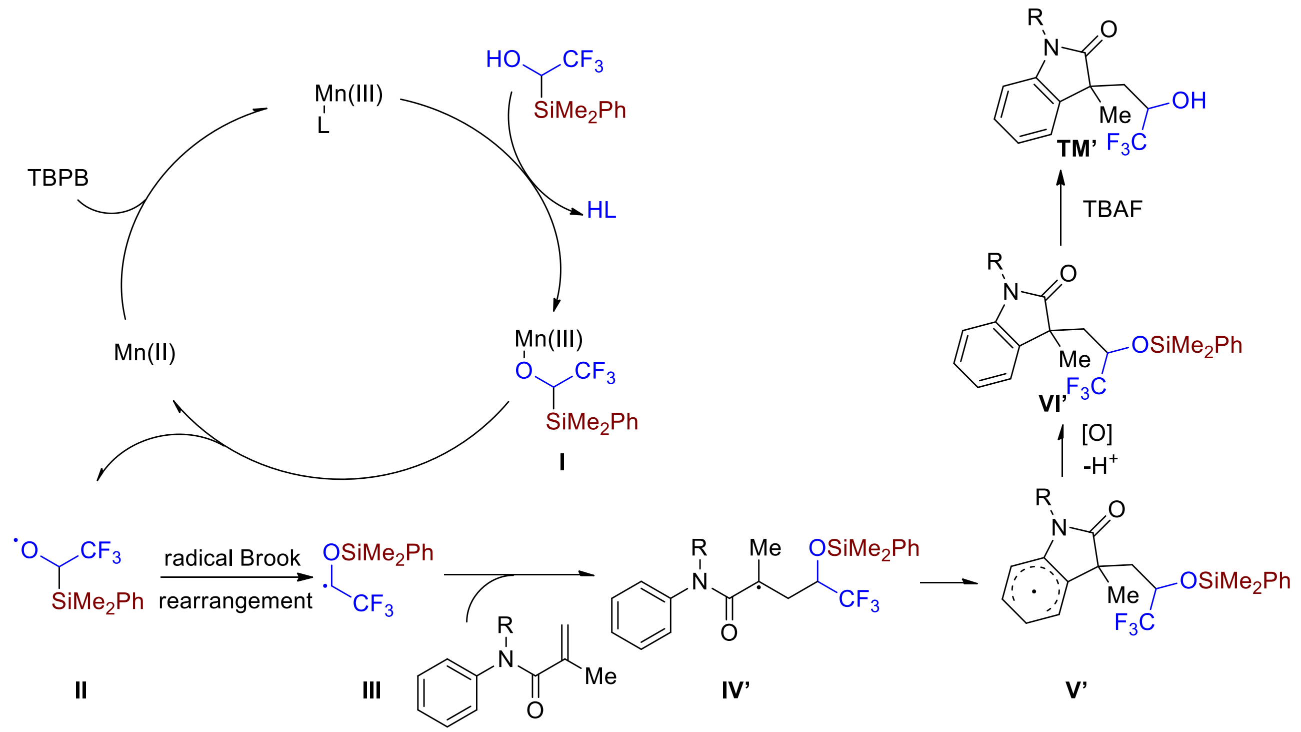

Supplementary Figure 14. Proposed Mechanism for alkylation via radical C-Si activation

For the alkylation reaction, we propose that radical **III** would be generated following similar mechanism as that in the allylation reaction (Fig. S3.). When an acryl amide was used as the radical acceptor instead of an allylic sulfone, we propose that radical **III** could undergo addition reaction to generate intermediate **IV'**, which undergo intramolecular addition to generate intermediate **V'**. Aromatization reaction via radical oxidation and deprotonation then would generate compound **VI'**. The alcohol product **TM'** would be generated after the desilylation step. Mn(III) catalyst is likely to be regenerated by the oxidation of Mn(II) by TBPB. Similar oxdative aromatization process was also proposed in the Fe and Ag catalyzed radical reactions of acryl amides. [15,16]

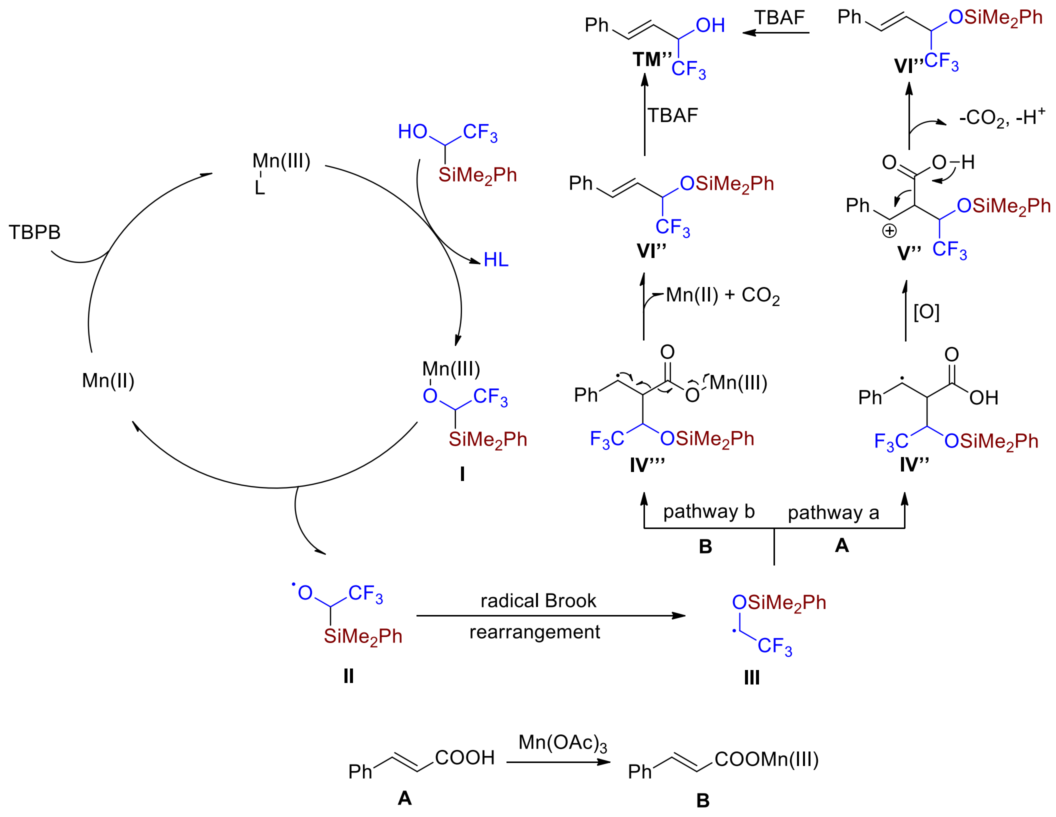

Supplementary Figure 15 Proposed Mechanism for alkenylation via radical C-Si activation

There are reports on radical decarboxylative alkenylation with α,β-unsaturated carboxylic acids.[17,18] Based on our experimental results and literature reports,[17,18] we propose a possible mechanism for our reaction as shown in Fig. S4. Ligand exchange between Mn(III) species and alcohol **1a** might generate intermediate **I**, which undergoes homolysis to produce alkoxyl radical **II** and Mn(II) intermediate. Carbon radical **III** would be generated through Brook rearrangement, and then undergo radical addition reaction via two possible pathways to generate **TM''**.

Pathway a: addition of radical **III** to the α-position of the double bond in an α,β-unsaturated carboxylic acid would generate intermediate **IV''**. Intermediate IV'' was oxidized to cation intermediate **V''** which then eliminated carbon dioxide and proton to generate the product **VI''**. Similar proposal was proposed in Ni-catalyzed radical alkenylation with α,β-unsaturated carboxylic acids.[17] The alcohol product **TM''** would be generated after the desilylation step.

Pathway b: compound **A** could be transformed to compound **B** via ligand exchange process. Addition of radical **III** to the α-position of the double bond in compound **B** would generate intermediate **IV''',** which then eliminate carbon dioxide and Mn(II) to generate compound **VI''**. Similar proposal was proposed in Cu-catalyzed alkenylation with α,β-unsaturated carboxylic acids. The alcohol product **TM''** would be generated after the desilylation step.

Mn(III) catalyst is likely to be regenerated by the oxidation of Mn(II) by TBPB.

Synthesis of α-CF_3 substituted homoallylic alcohols

Ethyl 5,5,5-trifluoro-4-(((dimethyl)(phenyl)silyl)oxy)-2-methylenepentanoate (8a)

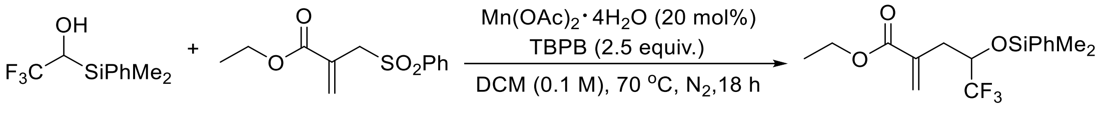

1a **7a** **8a**

Under N_2 atmosphere, to a dried 10 mL Schlenk tube equipped with a magnetic stir bar containing $Mn(OAc)_2$•$4H_2O$ (4.9 mg, 0.02 mmol, 20 mol%) was added DCM (1 mL, 0.1 M), **1a** (23.4 mg, 0.1 mmol), **7a** (50.8 mg, 0.2 mmol, 2.0 equiv.) and TBPB (48.6 mg, 0.25 mmol, 2.5 equiv.) sequentially. The tube was sealed, and the resulting mixture was kept stirring at 70 °C in heating block for 18 h. The reaction mixture was quenched with water (2 mL), extracted with DCM

(3×10 mL) and organic phase was combined and washed with brine, dried over Na_2SO_4, concentrated under reduced pressure. The crude product was purified with flash column chromatography on silica gel (200~300 mesh) and PE/EA (50/1~20/1, v/v) as eluent to afford 22.1 mg of the title compound as a colorless oil (64% yield).

R_f = 0.60 (PE/EA = 20/1, v/v). NMR Spectroscopy: ^{1}H NMR (400 MHz, $CDCl_3$) δ 7.52 (d, J = 6.4 Hz, 2H), 7.43–7.35 (m, 3H), 6.26 (s, 1H), 5.65 (s, 1H), 4.23–4.09 (m, 3H), 2.78–2.42 (m, 2H), 1.24 (t, J = 7.3 Hz, 3H), 0.38 (s, 6H); ^{13}C NMR (100 MHz, $CDCl_3$) δ 166.4, 136.1, 134.9, 133.8, 130.1, 130.0, 128.0, 125.0 (q, J = 281.0 Hz), 70.1 (q, J = 30.7 Hz), 61.0, 34.6, 14.2, –1.27, –1.47; ^{19}F NMR (375 MHz, $CDCl_3$) δ –78.5 (bs, 3F). IR (ATR): 2956, 2922, 2855, 1715, 1260, 1170, 1129, 1018, 790, 701 cm^{-1}. HRMS (ESI, m/z): calcd for $C_{16}H_{22}F_3O_3Si^+$ (M+H)$^+$: 347.1289; Found: 347.1273.

4-((Dimethyl(phenyl)silyl)oxy)-5,5,5-trifluoro-2-methylene-1-phenylpentan-1-one (20)

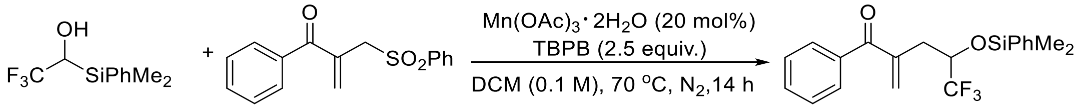

1a **7b** **20**

Under N_2 atmosphere, to a dried 10 mL Schlenk tube equipped with a magnetic stir bar containing $Mn(OAc)_3•2H_2O$ (26.8 mg, 0.1 mmol, 20 mol%), **7b** (429.5 mg, 1.5 mmol, 3.0 equiv.) was added DCM (5 mL), **1a** (117.1mg, 0.5 mmol) and TBPB (242.7 mg, 1.25 mmol, 2.5 equiv.) sequentially. The tube was then sealed, and the resulting mixture was stirred at 70 °C in a heating block for 14 h. The reaction mixture was quenched with water (2 mL) and extracted with DCM (3×10 mL). The organic phase was combined and washed with brine, dried over Na_2SO_4, concentrated under reduced pressure. The crude product was purified with column chromatography on silica gel (200~300 mesh) and PE/EA (20/1~10/1, v/v) as eluent to afford 149.4 mg of the title compound as a colorless oil (79% yield).

R_f = 0.30 (PE/EA = 80/1, v/v). ^{1}H NMR (400 MHz, $CDCl_3$) δ 7.55 (d, J = 7.5 Hz, 2H), 7.45–7.41 (m, 3H), 7.32–7.23 (m, 5H), 5.88 (s, 1H), 5.64 (s, 1H), 4.30–4.22 (m, 1H), 2.88–2.53 (m, 2H), 0.29–0.28 (m, 6H); ^{13}C NMR (100 MHz, $CDCl_3$) δ 197.4, 141.8, 137.5, 136.5, 133.7, 132.3, 131.4, 130.1, 129.6, 128.3, 128.0, 127.8, 125.1 (q, J = 281.7 Hz), 70.0 (q, J = 30.7 Hz), 34.9, –1.2, –1.7; ^{19}F NMR (375 MHz, $CDCl_3$) δ –77.6 (d, J = 6.0 Hz, 3F). IR(ATR): 3071, 2960, 1655, 1446, 1338, 1282, 1163, 1126, 1051, 790 cm^{-1}. HRMS (ESI, m/z): calcd for $C_{20}H_{21}F_3NaO_2Si^+$ (M+Na)$^+$: 401.1155; Found: 401.1161.

Ethyl 5,5,5-trifluoro-4-hydroxy-2-methylenepentanoate (9a)

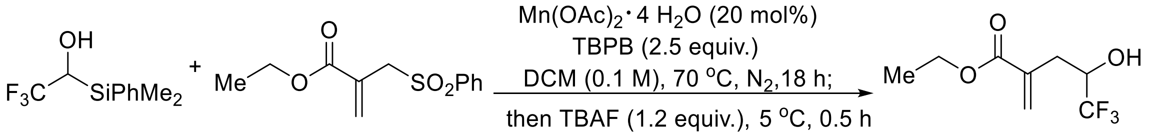

1a **7a** **9a**

Under N_2 atmosphere, to a dried 10 mL Schlenk tube equipped with a magnetic stir bar containing $Mn(OAc)_2•4H_2O$ (14.7 mg, 0.06 mmol, 20 mol%) was added DCM (3 mL, 0.1 M), **1a** (70.2 mg, 0.3 mmol), **7a** (152.4 mg, 0.6 mmol, 2.0 equiv.) and TBPB (145.7 mg, 0.75 mmol, 2.5 equiv.) sequentially. The tube was sealed, and the resulting mixture was kept stirring at 70 °C in heating block for 18 h. The mixture was then cooled to 5 °C with ice bath, TBAF (1.0 M in THF, 0.36 mL, 0.36 mmol, 1.2 equiv.) was added and the resulting mixture was stirred at 5 °C for 0.5 h. The reaction mixture was quenched with water (2 mL), extracted with DCM (3×10 mL) and organic phase was combined and washed with brine, dried over Na_2SO_4, concentrated under reduced pressure. The crude product was purified with column chromatography on silica gel (200~300 mesh) and PE/EA (20/1~10/1, v/v) as eluent to afford 40.0 mg of the title compound as a colorless oil (62% yield).

R_f = 0.23 (PE/EA = 8/1, v/v). NMR Spectroscopy: ^{1}H NMR (400 MHz, $CDCl_3$) δ 6.35 (s, 1H), 5.80 (s, 1H), 4.25 (q, J = 7.2 Hz, 2H), 4.16–4.08 (m, 1H), 3.63 (s, 1H), 2.78–2.57 (m, 2H), 1.32 (t, J = 7.2 Hz, 3H); ^{13}C NMR (100 MHz, $CDCl_3$) δ 168.1, 135.2, 129.8, 124.9 (q, J = 280.5 Hz), 70.0 (q, J = 30.7 Hz), 61.8, 33.5, 14.2; ^{19}F NMR (375 MHz, $CDCl_3$) δ –79.6 (d, J = 6.0 Hz, 3F). IR (ATR): 3444, 2986, 2937, 1700, 1633, 1413, 1316, 1275, 1163, 1126, 1021, 712 cm^{-1}. HRMS (ESI, m/z): calcd for $C_8H_{11}F_3NaO_3^+$ (M+Na)$^+$: 235.0552; Found: 235.0556.

5,5,5-Trifluoro-4-hydroxy-2-methylene-1-phenylpentan-1-one (9b)

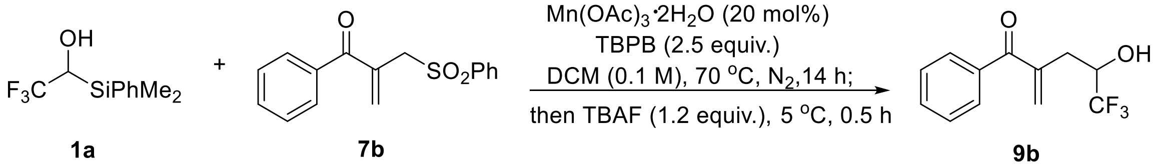

Under N_2 atmosphere, to a dried 10 mL Schlenk tube equipped with a magnetic stir bar containing $Mn(OAc)_3$•$2H_2O$ (16.1 mg, 0.06 mmol, 20 mol%) and **7b** (257.4 mg, 0.9 mmol, 3.0 equiv.) was added DCM (3 mL, 0.1 M), **1a** (70.2 mg, 0.3 mmol) and TBPB (145.7 mg, 0.75 mmol, 2.5 equiv.) sequentially. The tube was then sealed, and the resulting mixture was stirred at 70 °C in a heating block for 14 h, after which the mixture was cooled to 5 °C with ice bath. TBAF (1.0 M in THF, 0.36 mL, 0.36 mmol, 1.2 equiv.) was added and the mixture was stirred at 5 °C for 0.5 h. The reaction mixture was quenched with water (2 mL) and extracted with DCM (3×10 mL). The organic phase was combined and washed with brine, dried over Na_2SO_4, concentrated under reduced pressure. The crude product was purified with column chromatography on silica gel (200~300 mesh) and PE/EA (20/1~10/1, v/v) as eluent to afford 53.0 mg of the title compound as a yellow oil (71% yield).

R_f = 0.40 (PE/EA = 5/1, v/v). ^{1}H NMR (400 MHz, $CDCl_3$) δ 7.77 (d, J = 7.3 Hz, 2H), 7.60 (t, J = 7.3 Hz, 1H), 7.47 (t, J = 7.6 Hz, 2H), 6.16 (s, 1H), 5.90 (s, 1H), 4.32 (d, J = 5.2 Hz, 1H), 4.15–4.14 (m, 1H), 2.90–2.71 (m, 2H); ^{13}C NMR (100 MHz, $CDCl_3$) δ 199.5, 142.2, 136.7, 133.2, 131.9, 130.1, 128.5, 125.0 (q, J = 280.6 Hz), 70.5 (q, J = 31.2 Hz), 33.8; ^{19}F NMR (375 MHz, $CDCl_3$) δ –79.3 (d, J = 6.0 Hz, 3F). IR (ATR): 3418, 3064, 2933, 1648, 1446, 1338, 1275, 1223, 1163, 1036, 753 cm^{-1}. HRMS (ESI, m/z): calcd for $C_{12}H_{12}F_3O_2^+$ $(M+H)^+$: 245.0784; Found: 245.0778.

1-([1,1'-Biphenyl]-4-yl)-5,5,5-trifluoro-4-hydroxy-2-methylenepentan-1-one (9c)

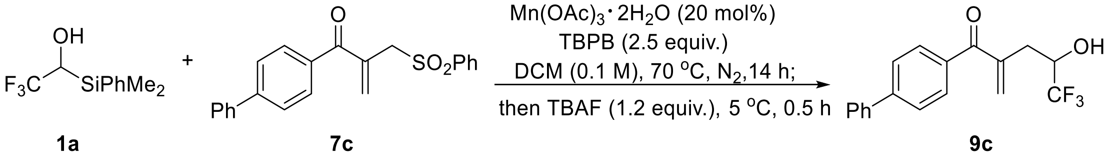

Under N_2 atmosphere, to a dried 10 mL Schlenk tube equipped with a magnetic stir bar containing $Mn(OAc)_3$•$2H_2O$ (16.1 mg, 0.06 mmol, 20 mol%) and **7c** (326.2 mg, 0.9 mmol, 3.0 equiv.) was added DCM (3 mL, 0.1 M), **1a** (70.2 mg, 0.3 mmol) and TBPB (145.7 mg, 0.75 mmol, 2.5 equiv.) sequentially. The tube was then sealed, and the resulting mixture was stirred at 70 °C in a heating block for 14 h, after which the mixture was cooled to 5 °C with ice bath. TBAF (1.0 M in THF, 0.36 mL, 0.36 mmol, 1.2 equiv.) was added and the mixture was stirred at 5 °C for 0.5 h. The reaction mixture was quenched with water (2 mL) and extracted with DCM (3×10 mL). The organic phase was combined and washed with brine, dried over Na_2SO_4, concentrated under reduced pressure. The crude product was purified with column chromatography on silica gel (200~300 mesh) and PE/EA (20/1~10/1, v/v) as eluent to afford 74.9 mg of the title compound as a white solid (78% yield).

Gram scale experiment

Under N_2 atmosphere, to a dried 100 mL Schlenk tube equipped with a magnetic stir bar containing $Mn(OAc)_3$•$2H_2O$ (321.7 mg, 1.2 mmol, 20 mol%) and **7c** (6.52 g, 18.0 mmol, 3.0 equiv.) was added DCM (50 mL), **1a** (1.41 g, 6 mmol) and TBPB (2.91 g, 15 mmol, 2.5 equiv.) sequentially. The tube was then sealed, and the resulting mixture was stirred at 70 °C in oil bath for 14 h, after which the mixture was cooled to 5 °C with ice bath. TBAF (1.0 M in THF, 7.2 mL, 7.2 mmol, 1.2 equiv.) was added and the mixture was stirred at 5 °C for 0.5 h. The reaction mixture was quenched with water (20 mL) and extracted with DCM (3×100 mL). The organic phase was combined and washed with brine, dried over Na_2SO_4, concentrated under reduced pressure. The crude product was purified with column chromatography on silica gel (200~300 mesh) and PE/EA (20/1~10/1, v/v) as eluent to afford 1.25 g of the title compound as a white solid (65% yield) and starting material **7c** was 61% recovered (3.97 g, 11.0 mmol).

R_f = 0.50 (PE/EA = 5/1, v/v). mp: 69 °C–71 °C (from PE and EA). ^{1}H NMR (400 MHz, $CDCl_3$) δ 7.88 (d, J = 8.3 Hz,

2H), 7.69 (d, J = 8.3 Hz, 2H), 7.63 (d, J = 7.3 Hz, 2H), 7.49 (t, J = 7.3 Hz, 2H), 7.42 (t, J = 7.2 Hz, 1H), 6.18 (s, 1H), 5.95 (s, 1H), 4.38 (d, J = 4.9 Hz, 1H), 4.18–4.17 (m, 1H), 2.92–2.74 (m, 2H); ^{13}C NMR (100 MHz, $CDCl_3$) δ 199.1, 146.1, 142.3, 139.8, 135.3, 131.5, 130.8, 129.1, 128.5, 127.4, 127.2, 125.0 (q, J = 280.8 Hz), 70.6 (q, J = 30.7 Hz), 33.9; ^{19}F NMR (375 MHz, $CDCl_3$) δ –79.3 (d, J = 6.0 Hz, 3F). IR (ATR): 3392, 3060, 2926, 1640, 1599, 1409, 1344, 1275, 1163, 1129, 1029, 757 cm^{-1}. HRMS (ESI, m/z): calcd for $C_{18}H_{16}F_3O_2^+$ $(M+H)^+$: 321.1097; Found: 321.1096.

5,5,5-Trifluoro-1-(4-fluorophenyl)-4-hydroxy-2-methylenepentan-1-one (9d)

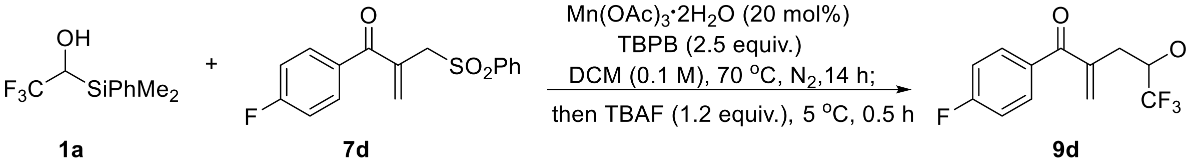

Under N_2 atmosphere, to a dried 10 mL Schlenk tube equipped with a magnetic stir bar containing $Mn(OAc)_3$•$2H_2O$ (16.1 mg, 0.06 mmol, 20 mol%) and **7d** (273.6 mg, 0.9 mmol, 3.0 equiv.) was added DCM (3 mL, 0.1 M), **1a** (70.2 mg, 0.3 mmol) and TBPB (145.7 mg, 0.75 mmol, 2.5 equiv.) sequentially. The tube was then sealed, and the resulting mixture was stirred at 70 °C in a heating block for 14 h, after which the mixture was cooled to 5 °C with ice bath. TBAF (1.0 M in THF, 0.36 mL, 0.36 mmol, 1.2 equiv.) was added and the mixture was stirred at 5 °C for 0.5 h. The reaction mixture was quenched with water (2 mL) and extracted with DCM (3×10 mL). The organic phase was combined and washed with brine, dried over Na_2SO_4, concentrated under reduced pressure. The crude product was purified with column chromatography on silica gel (200~300 mesh) and PE/EA (20/1~10/1, v/v) as eluent to afford 49.0 mg of the title compound as a yellow oil (62% yield).

R_f = 0.50 (PE/EA = 5/1, v/v). ^1H NMR (400 MHz, $CDCl_3$) δ 7.84–7.81 (m, 2H), 7.17–7.12 (m, 2H), 6.14 (s, 1H), 5.86 (s, 1H), 4.15 (bs, 2H), 2.89–2.70 (m, 2H); ^{13}C NMR (100 MHz, $CDCl_3$) δ 197.9, 165.9 (d, J = 255.3 Hz), 142.2, 132.9 (d, J = 2.9 Hz), 132.7 (d, J = 8.7 Hz), 131.4, 124.9 (q, J = 280.8 Hz), 115.8 (d, J = 22.2 Hz), 70.4 (q, J = 30.8 Hz), 33.8; ^{19}F NMR (375 MHz, $CDCl_3$) δ –79.4 (d, J = 6.0 Hz, 3F), –104.6 (s, 1F). IR (ATR): 3425, 2930, 1644, 1416, 1338, 1275, 1156, 1129, 1029, 850 cm^{-1}. HRMS (ESI, m/z): calcd for $C_{12}H_{11}F_4O_2^+$ $(M+H)^+$: 263.0690; Found: 263.0684.

1-(4-Chlorophenyl)-5,5,5-trifluoro-4-hydroxy-2-methylenepentan-1-one (9e)

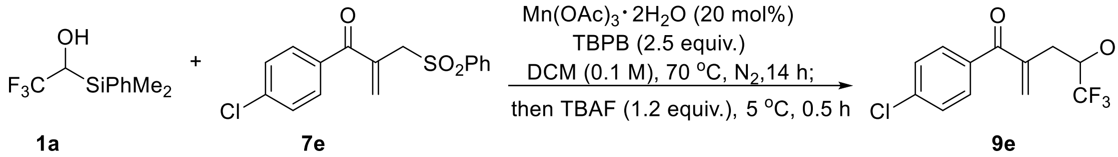

Under N_2 atmosphere, to a dried 10 mL Schlenk tube equipped with a magnetic stir bar containing $Mn(OAc)_3$•$2H_2O$ (16.1 mg, 0.06 mmol, 20 mol%) and **7e** (288.0 mg, 0.9 mmol, 3.0 equiv.) was added DCM (3 mL, 0.1 M), **1a** (70.2 mg, 0.3 mmol), TBPB (145.7 mg, 0.75 mmol, 2.5 equiv.) sequentially. The tube was then sealed, and the resulting mixture was stirred at 70 °C in a heating block for 14 h, after which the mixture was cooled to 5 °C with ice bath. TBAF (1.0 M in THF, 0.36 mL, 0.36 mmol, 1.2 equiv.) was added and the mixture was stirred at 5 °C for 0.5 h. The reaction mixture was quenched with water (2 mL) and extracted with DCM (3×10 mL). The organic phase was combined and washed with brine, dried over Na_2SO_4, concentrated under reduced pressure. The crude product was purified with column chromatography on silica gel (200~300 mesh) and PE/EA (20/1~10/1, v/v) as eluent to afford 57.2 mg of the title compound as a yellow oil (69% yield).

R_f = 0.40 (PE/EA = 5/1, v/v). ^1H NMR (400 MHz, $CDCl_3$) δ 7.73 (d, J = 8.6 Hz, 2H), 7.45 (d, J = 8.3 Hz, 2H), 6.16 (s, 1H), 5.87 (s, 1H), 4.15 (s, 1H), 3.96 (s, 1H), 2.90–2.71 (m, 2H); ^{13}C NMR (100 MHz, $CDCl_3$) δ 198.1, 142.1, 139.7, 135.0, 131.7, 131.4, 128.9, 124.9 (q, J = 281.8 Hz), 70.4 (q, J = 30.7 Hz), 33.8; ^{19}F NMR (375 MHz, $CDCl_3$) δ –79.4 (d, J = 8.9 Hz, 3F). IR (ATR): 3437, 2930, 1651, 1588, 1478, 1402, 1334, 1275, 1163, 1129, 1092, 790 cm^{-1}. HRMS (ESI, m/z): calcd for $C_{12}H_{11}ClF_3O_2^+$ $(M+H)^+$: 279.0394; Found: 279.0389.

1-(3-Chlorophenyl)-5,5,5-trifluoro-4-hydroxy-2-methylenepentan-1-one (9f)

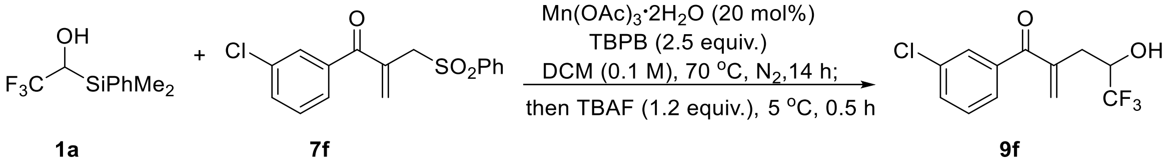

Under N_2 atmosphere, to a dried 10 mL Schlenk tube equipped with a magnetic stir bar containing $Mn(OAc)_3$•$2H_2O$ (16.1 mg, 0.06 mmol, 20 mol%) and **7f** (288.0 mg, 0.9 mmol, 3.0 equiv.) was added DCM (3 mL, 0.1 M), **1a** (70.2 mg, 0.3 mmol), TBPB (145.7 mg, 0.75 mmol, 2.5 equiv.) sequentially. The tube was then sealed, and the resulting mixture was stirred at 70 °C in a heating block for 14 h, after which the mixture was cooled to 5 °C with ice bath. TBAF (1.0 M in THF, 0.36 mL, 0.36 mmol, 1.2 equiv.) was added and the mixture was stirred at 5 °C for 0.5 h. The reaction mixture was quenched with water (2 mL) and extracted with DCM (3×10 mL). The organic phase was combined and washed with brine, dried over Na_2SO_4, concentrated under reduced pressure. The crude product was purified with column chromatography on silica gel (200~300 mesh) and PE/EA (20/1~10/1, v/v) as eluent to afford 65.1 mg of the title compound as a yellow oil (78% yield).

R_f = 0.40 (PE/EA = 5/1, v/v). ^{1}H NMR (400 MHz, $CDCl_3$) δ 7.73 (s, 1H), 7.63 (d, J = 8.0 Hz, 1H), 7.56 (d, J = 8.0 Hz, 1H), 7.41 (t, J = 7.8 Hz, 1H), 6.19 (s, 1H), 5.90 (s, 1H), 4.19–4.13 (m, 1H), 3.85 (d, J = 5.8 Hz, 1H), 2.91–2.71 (m, 2H); ^{13}C NMR (100 MHz, $CDCl_3$) δ 197.9, 142.0, 138.5, 134.8, 133.0, 132.3, 129.9, 129.9, 128.0, 124.9 (q, J = 280.8 Hz), 70.2 (q, J = 30.9 Hz), 33.6; ^{19}F NMR (375 MHz, $CDCl_3$) δ –79.4 (d, J = 6.0 Hz, 3F). IR (ATR): 3418, 3071, 2930, 1651, 1420, 1334, 1275, 1163, 1129, 1033, 768 cm^{-1}. HRMS (ESI, m/z): calcd for $C_{12}H_{10}ClF_3NaO_2^+$ (M+Na)$^+$: 301.0214; Found: 301.0216.

1-(2-Chlorophenyl)-5,5,5-trifluoro-4-hydroxy-2-methylenepentan-1-one (9g)

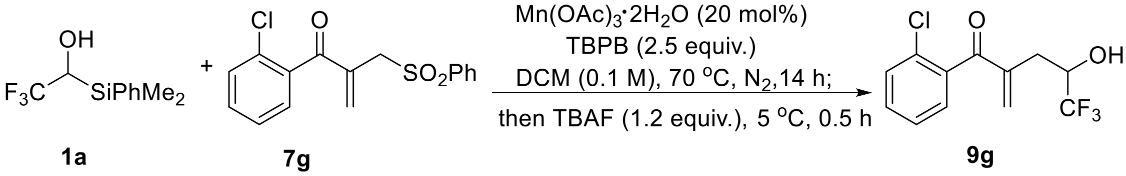

Under N_2 atmosphere, to a dried 10 mL Schlenk tube equipped with a magnetic stir bar containing $Mn(OAc)_3$•$2H_2O$ (16.1 mg, 0.06 mmol, 20 mol%) and **7g** (288.0 mg, 0.9 mmol, 3.0 equiv.) was added DCM (3 mL, 0.1 M), **1a** (70.2 mg, 0.3 mmol) and TBPB (145.7 mg, 0.75 mmol, 2.5 equiv.) sequentially. The tube was then sealed, and the resulting mixture was stirred at 70 °C in a heating block for 14 h, after which the mixture was cooled to 5 °C with ice bath. TBAF (1.0 M in THF, 0.36 mL, 0.36 mmol, 1.2 equiv.) was added and the mixture was stirred at 5 °C for 0.5 h. The reaction mixture was quenched with water (2 mL) and extracted with DCM (3×10 mL). The organic phase was combined and washed with brine, dried over Na_2SO_4, concentrated under reduced pressure. The crude product was purified with column chromatography on silica gel (200~300 mesh) and PE/EA (20/1~10/1, v/v) as eluent to afford 69.4 mg of the title compound as a yellow oil (83% yield).

R_f = 0.40 (PE/EA = 5/1, v/v). ^{1}H NMR (400 MHz, $CDCl_3$) δ 7.42–7.41 (m, 2H), 7.36–7.28 (m, 2H), 6.24 (s, 1H), 5.84 (s, 1H), 4.26–4.18 (m, 1H), 3.44 (s, 1H), 2.96–2.71 (m, 2H); ^{13}C NMR (100 MHz, $CDCl_3$) δ 198.4, 143.1, 137.7, 135.4, 131.5, 131.2, 130.2, 129.0, 126.7, 125.0 (q, J = 280.5 Hz), 69.7 (q, J = 31.2 Hz), 32.3; ^{19}F NMR (375 MHz, $CDCl_3$) δ –79.4 (d, J = 6.0 Hz, 3F). IR(ATR): 3429, 3071, 2937, 1655, 1435, 1342, 1275, 1163, 1129, 1033, 746 cm^{-1}. HRMS (ESI, m/z): calcd for $C_{12}H_{11}ClF_3O_2^+$ (M+H)$^+$: 279.0394; Found: 279.0388.

1-(4-Bromophenyl)-5,5,5-trifluoro-4-hydroxy-2-methylenepentan-1-one (9h)

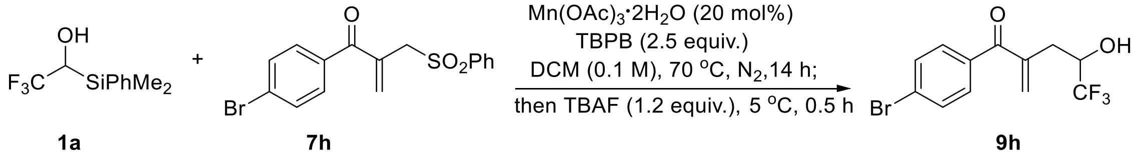

Under N_2 atmosphere, to a dried 10 mL Schlenk tube equipped with a magnetic stir bar containing $Mn(OAc)_3•2H_2O$ (16.1 mg, 0.06 mmol, 20 mol%) and **7h** (329.0 mg, 0.9 mmol, 3.0 equiv.) was added DCM (3 mL, 0.1 M), **1a** (70.2 mg, 0.3 mmol) and TBPB (145.7 mg, 0.75 mmol, 2.5 equiv.) sequentially. The tube was then sealed, and the resulting mixture was stirred at 70 °C in a heating block for 14 h, after which the mixture was cooled to 5 °C with ice bath. TBAF (1.0 M in THF, 0.36 mL, 0.36 mmol, 1.2 equiv.) was added and the mixture was stirred at 5 °C for 0.5 h. The reaction mixture was quenched with water (2 mL) and extracted with DCM (3×10 mL). The organic phase was combined and washed with brine, dried over Na_2SO_4, concentrated under reduced pressure. The crude product was purified with column chromatography on silica gel (200~300 mesh) and PE/EA (20/1~10/1, v/v) as eluent to afford 78.9 mg of the title compound as a slight yellow oil (81% yield).

R_f = 0.50 (PE/EA = 5/1, v/v). ^1H NMR (400 MHz, $CDCl_3$) δ 7.66–7.60 (m, 4H), 6.17 (s, 1H), 5.87 (s, 1H), 4.15–4.14 (m, 1H), 3.92–3.91 (m, 1H), 2.90–2.71 (m, 2H); ^{13}C NMR (100 MHz, $CDCl_3$) δ 198.3, 142.1, 135.5, 131.9, 131.8, 131.5, 128.4, 124.9 (q, J = 280.8 Hz), 70.4 (q, J = 30.9 Hz), 33.7; ^{19}F NMR (375 MHz, $CDCl_3$) δ –79.4 (d, J = 6.0 Hz, 3F). IR (ATR): 3422, 2920, 2855, 1648, 1584, 1398, 1275, 1167, 1133, 1074, 790 cm^{-1}. HRMS (ESI, m/z): calcd for $C_{12}H_{11}BrF_3O_2^+$ $(M+H)^+$: 322.9889; Found: 322.9888.

5,5,5-Trifluoro-4-hydroxy-1-(4-iodophenyl)-2-methylenepentan-1-one (9i)

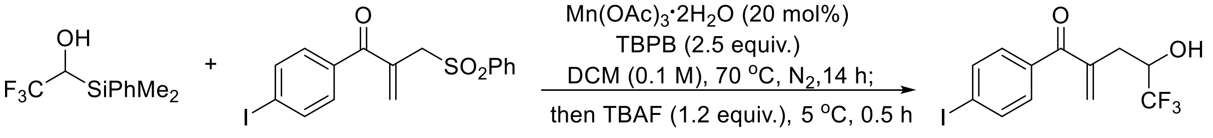

Under N_2 atmosphere, to a dried 10 mL Schlenk tube equipped with a magnetic stir bar containing $Mn(OAc)_3•2H_2O$ (16.1 mg, 0.06 mmol, 20 mol%), **7i** (370.8 mg, 0.9 mmol, 3.0 equiv.) was added DCM (3 mL, 0.1 M), **1a** (70.2 mg, 0.3 mmol) and TBPB (145.7 mg, 0.75 mmol, 2.5 equiv.) sequentially. The tube was then sealed, and the resulting mixture was stirred at 70 °C in a heating block for 14 h, after which the mixture was cooled to 5 °C with ice bath. TBAF (1.0 M in THF, 0.36 mL, 0.36 mmol, 1.2 equiv.) was added and the mixture was stirred at 5 °C for 0.5 h. The reaction mixture was quenched with water (2 mL) and extracted with DCM (3×10 mL). The organic phase was combined and washed with brine, dried over Na_2SO_4, concentrated under reduced pressure. The crude product was purified with column chromatography on silica gel (200~300 mesh) and PE/EA (20/1~10/1, v/v) as eluent to afford 72.0 mg of the title compound as a colorless oil (65% yield).

R_f = 0.50 (PE/EA = 5/1, v/v). ^1H NMR (400 MHz, $CDCl_3$) δ 7.83 (d, J = 8.3 Hz, 2H), 7.48 (d, J = 8.3 Hz, 2H), 6.16 (s, 1H), 5.86 (s, 1H), 4.14 (s, 1H), 3.95 (d, J = 4.6 Hz, 1H), 2.89–2.70 (m, 2H); ^{13}C NMR (100 MHz, $CDCl_3$) δ 198.5, 142.1, 137.9, 136.0, 131.8, 131.4, 124.9 (q, J = 280.8 Hz), 101.0, 70.3 (q, J = 31.1 Hz), 33.8; ^{19}F NMR (375 MHz, $CDCl_3$) δ –79.4 (d, J = 6.0 Hz, 3F). IR (ATR): 3429, 2926, 2855, 1648, 1480, 1390, 1275, 1163, 1126, 1100, 787 cm^{-1}. HRMS (ESI, m/z): calcd for $C_{12}H_{10}F_3INaO_2^+$ $(M+Na)^+$: 392.9570; Found: 392.9560.

4-Bromobut-2-en-1-yl 5,5,5-trifluoro-4-hydroxy-2-methylenepentanoate (9j)

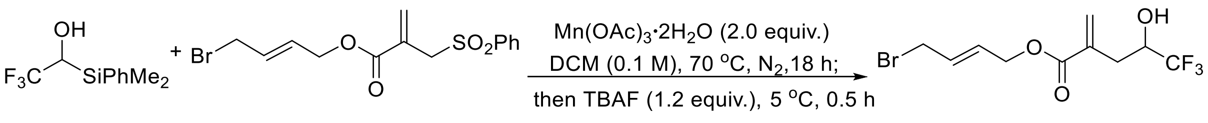

Under N_2 atmosphere, to a dried Schlenk tube containing $Mn(OAc)_3•2H_2O$ (168.0 mg, 0.6 mmol, 2.0 equiv.), **7j** (323.1 mg, 0.9 mmol, 3.0 equiv.) was added DCM (3 mL, 0.1 M), **1a** (70.2 mg, 0.3 mmol) sequentially. The tube was sealed, and the resulting mixture was kept stirring at 70 °C in heating block for 6 h. After which the mixture was cooled to –10 °C with low temperature bath, TBAF (1.0 M in THF, 0.36 mL, 0.36 mmol, 1.2 equiv.) was added and the mixture was stirred at –10 °C for 0.5 h. The reaction mixture was quenched with water (2 mL), extracted with DCM (3×10 mL) and organic phase was combined and washed with brine, dried over Na_2SO_4, concentrated under reduced pressure. The crude

product was purified with column chromatography on silica gel (200~300 mesh) and PE/EA (20/1~5/1, v/v) as eluent to afford 45.7 mg of the title compound as a colorless oil (48% yield).
R_f = 0.57 (PE/EA = 4/1 v/v). NMR Spectroscopy: ^{1}H NMR (400 MHz, $CDCl_3$) δ 6.39 (s, 1H), 5.95–5.92 (m, 2H), 5.85 (s, 1H), 4.71 (d, J = 4.3 Hz, 2H), 4.18–4.07 (m, 3H), 3.28 (s, 1H), 2.80–2.58 (m, 2H); ^{13}C NMR (100 MHz, $CDCl_3$) δ 167.5, 134.9, 130.4, 130.3, 127.9, 124.9 (q, J = 281.2 Hz), 69.9 (q, J = 31.2 Hz), 64.7, 43.9, 33.5. ^{19}F NMR (375 MHz, $CDCl_3$) δ –79.6 (d, J = 8.9 Hz, 3F). IR (ATR): 3429, 2922, 2855, 2359, 2259, 1715, 1126, 783 cm^{-1}. HRMS (ESI, m/z): calcd for $C_{10}H_{12}F_3O_3^+$ (M–Br)$^+$: 237.0733; Found: 237.0726.

5,5,5-Trifluoro-4-hydroxy-1-(4-methoxyphenyl)-2-methylenepentan-1-one (9k)

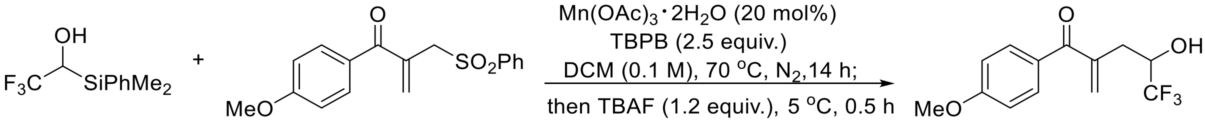

1a **7k** **9k**

Under N_2 atmosphere, to a dried 10 mL Schlenk tube equipped with a magnetic stir bar containing $Mn(OAc)_3$•$2H_2O$ (16.1 mg, 0.06 mmol, 20 mol%), **7k** (284.4 mg, 0.9 mmol, 3.0 equiv.) was added DCM (3 mL, 0.1 M), **1a** (70.2 mg, 0.3 mmol) and TBPB (145.7 mg, 0.75 mmol, 2.5 equiv.) sequentially. The tube was then sealed, and the resulting mixture was stirred at 70 ºC in a heating block for 14 h, after which the mixture was cooled to 5 ºC with ice bath. TBAF (1.0 M in THF, 0.36 mL, 0.36 mmol, 1.2 equiv.) was added and the mixture was stirred at 5 ºC for 0.5 h. The reaction mixture was quenched with water (2 mL) and extracted with DCM (3×10 mL). The organic phase was combined and washed with brine, dried over Na_2SO_4, concentrated under reduced pressure. The crude product was purified with column chromatography on silica gel (200~300 mesh) and PE/EA (20/1~10/1, v/v) as eluent to afford 56.0 mg of the title compound as a colorless oil (68% yield).
R_f = 0.30 (PE/EA = 5/1, v/v). ^{1}H NMR (400 MHz, $CDCl_3$) δ 7.83 (d, J = 8.8 Hz, 2H), 6.95 (d, J = 9.2 Hz, 2H), 6.08 (s, 1H), 5.83 (s, 1H), 4.78 (d, J = 3.4 Hz, 1H), 4.11 (s, 1H), 3.88 (s, 3H), 2.85–2.67 (m, 2H); ^{13}C NMR (100 MHz, $CDCl_3$) δ 198.2, 164.1, 142.2, 132.7, 130.0, 129.0, 125.0 (q, J = 280.5 Hz), 113.9, 70.8 (q, J = 30.9 Hz), 55.7, 34.1; ^{19}F NMR (375 MHz, $CDCl_3$) δ –79.4 (d, J = 6.0 Hz, 3F). IR(ATR): 3414, 2937, 2844, 1636, 1595, 1424, 1260, 1163, 1129, 1029, 794 cm^{-1}. HRMS (ESI, m/z): calcd for $C_{13}H_{14}F_3O_3^+$ (M + H)$^+$: 275.0890; Found: 275.0880.

5,5,5-Trifluoro-4-hydroxy-2-methylene-1-(4-nitrophenyl)pentan-1-one (9l)

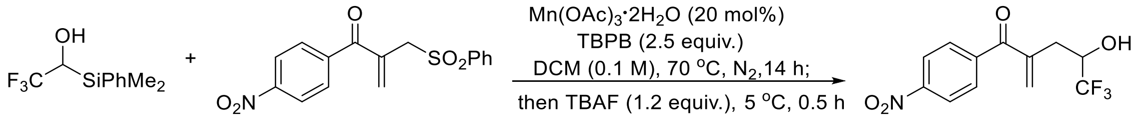

1a **7l** **9l**

Under N_2 atmosphere, to a dried 10 mL Schlenk tube equipped with a magnetic stir bar containing $Mn(OAc)_3$•$2H_2O$ (16.1 mg, 0.06 mmol, 20 mol%), **7l** (297.9mg, 0.9 mmol, 3.0 equiv.) was added DCM (3 mL, 0.1 M), **1a** (70.2 mg, 0.3 mmol) and TBPB (145.7 mg, 0.75 mmol, 2.5 equiv.) sequentially. The tube was then sealed, and the resulting mixture was stirred at 70 ºC in a heating block for 14 h, after which the mixture was cooled to 5 ºC with ice bath. TBAF (1.0 M in THF, 0.36 mL, 0.36 mmol, 1.2 equiv.) was added and the mixture was stirred at 5 ºC for 0.5 h. The reaction mixture was quenched with water (2 mL) and extracted with DCM (3×10 mL). The organic phase was combined and washed with brine, dried over Na_2SO_4, concentrated under reduced pressure. The crude product was purified with column chromatography on silica gel (200~300 mesh) and PE/EA (20/1~10/1, v/v) as eluent to afford 69.4 mg of the title compound as a yellow oil (80% yield).
R_f = 0.30 (PE/EA = 5/1, v/v). ^{1}H NMR (400 MHz, $CDCl_3$) δ 8.31 (d, J = 9.0 Hz, 2H), 7.89 (d, J = 9.0 Hz, 2H), 6.27 (s, 1H), 5.88 (s, 1H), 4.21–4.20 (m, 1H), 3.37 (s, 1H), 2.96–2.76 (m, 2H); ^{13}C NMR (100 MHz, $CDCl_3$) δ 197.1, 150.1, 142.3, 142.2, 133.2, 130.6, 124.6 (q, J = 281.0 Hz), 123.7, 69.9 (q, J = 31.1 Hz), 33.2; ^{19}F NMR (375 MHz, $CDCl_3$) δ –79.4 (d, J = 6.0 Hz, 3F). IR (ATR): 3444, 3109, 2930, 1655, 1416, 1349, 1275, 1163, 1129, 1029, 753 cm^{-1}. HRMS (ESI, m/z): calcd for $C_{12}H_{10}F_3NaNO_3^+$ (M+Na)$^+$: 312.0454; Found: 312.0450.

4-(5,5,5-Trifluoro-4-hydroxy-2-methylenepentanoyl)benzonitrile (9m)

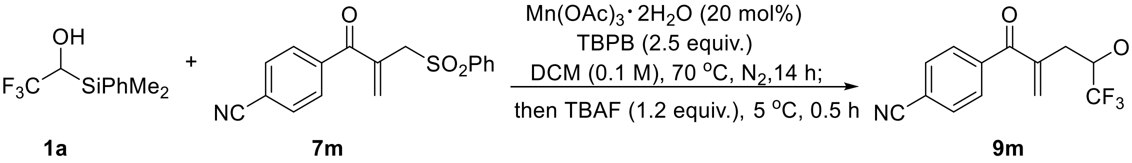

Under N_2 atmosphere, to a dried 10 mL Schlenk tube equipped with a magnetic stir bar containing $Mn(OAc)_3$•$2H_2O$ (16.1 mg, 0.06 mmol, 20 mol%), **7m** (280.2 mg, 0.9 mmol, 3.0 equiv.) was added DCM (3 mL, 0.1 M), **1a** (70.2 mg, 0.3 mmol) and TBPB (145.7 mg, 0.75 mmol, 2.5 equiv.) sequentially. The tube was then sealed, and the resulting mixture was stirred at 70 ºC in a heating block for 14 h, after which the mixture was cooled to 5 ºC with ice bath. TBAF (1.0 M in THF, 0.36 mL, 0.36 mmol, 1.2 equiv.) was added and the mixture was stirred at 5 ºC for 0.5 h. The reaction mixture was quenched with water (2 mL) and extracted with DCM (3×10 mL). The organic phase was combined and washed with brine, dried over Na_2SO_4, concentrated under reduced pressure. The crude product was purified with column chromatography on silica gel (200~300 mesh) and PE/EA (20/1~10/1, v/v) as eluent to afford 65.0 mg of the title compound as a yellow oil (80% yield).

R_f = 0.30 (PE/EA = 5/1, v/v). ^{1}H NMR (400 MHz, $CDCl_3$) δ 7.83 (d, J = 8.6 Hz, 2H), 7.77 (d, J = 8.6 Hz, 2H), 6.24 (s, 1H), 5.86 (s, 1H), 4.23–4.15 (m, 1H), 3.42 (s, 1H), 2.95–2.74 (m, 2H); ^{13}C NMR (100 MHz, $CDCl_3$) δ 197.4, 142.0, 140.7, 132.9, 132.4, 130.2, 124.9 (q, J = 280.3 Hz), 118.0, 116.1, 69.9 (q, J = 30.9 Hz), 33.3; ^{19}F NMR (375 MHz, $CDCl_3$) δ –79.4 (d, J = 6.0 Hz, 3F). IR (ATR): 3448, 2922, 2851, 2233, 1655, 1402, 1275, 1163, 1129, 1029, 798 cm^{-1}. HRMS (ESI, m/z): calcd for $C_{13}H_{10}F_3NNaO_2^+$ (M+Na)$^+$: 292.0556; Found: 292.0567.

5,5,5-Trifluoro-1-(furan-2-yl)-4-hydroxy-2-methylenepentan-1-one (9n)

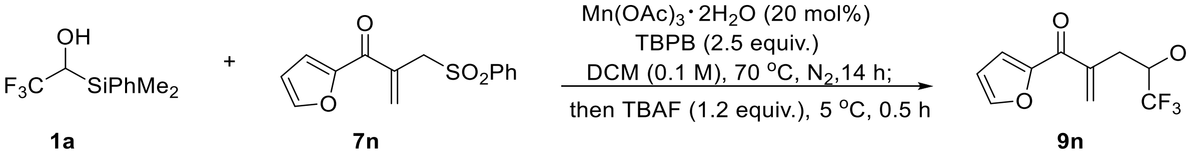

Under N_2 atmosphere, to a dried 10 mL Schlenk tube equipped with a magnetic stir bar containing $Mn(OAc)_3$•$2H_2O$ (16.1 mg, 0.06 mmol, 20 mol%), **7n** (248.7 mg, 0.9 mmol, 3.0 equiv.) was added DCM (3 mL, 0.1 M), **1a** (70.2 mg, 0.3 mmol) and TBPB (145.7 mg, 0.75 mmol, 2.5 equiv.) sequentially. The tube was then sealed and the resulting mixture was stirred at 70 ºC in a heating block for 14 h, after which the mixture was cooled to 5 ºC with ice bath. TBAF (1.0 M in THF, 0.36 mL, 0.36 mmol, 1.2 equiv.) was added and the mixture was stirred at 5 ºC for 0.5 h. The reaction mixture was quenched with water (2 mL) and extracted with DCM (3×10 mL). The organic phase was combined and washed with brine, dried over Na_2SO_4, concentrated under reduced pressure. The crude product was purified with column chromatography on silica gel (200~300 mesh) and PE/EA (20/1~10/1, v/v) as eluent to afford 56.0 mg of the title compound as a yellow oil (80% yield).

R_f = 0.40 (PE/EA = 5/1, v/v). ^{1}H NMR (400 MHz, $CDCl_3$) δ 7.71–7.70 (m, 1H), 7.26–7.25 (m, 1H), 6.59–6.58 (m, 1H), 6.27 (s, 1H), 6.07 (s, 1H), 4.15–4.07 (m, 1H), 2.83–2.65 (m, 2H); ^{13}C NMR (100 MHz, $CDCl_3$) δ 184.7, 151.4, 148.3, 141.8, 130.0, 124.9 (q, J = 280.8 Hz), 121.9, 112.6, 70.5 (q, J = 31.2 Hz), 34.0; ^{19}F NMR (375 MHz, $CDCl_3$) δ –79.4 (d, J = 6.0 Hz, 3F). IR (ATR): 3407, 3142, 2933, 1618, 1465, 1394, 1275, 1163, 1129, 1029, 768 cm^{-1}. HRMS (ESI, m/z): calcd for $C_{10}H_{10}F_3O_3^+$ (M + H)$^+$: 235.0577; Found: 235.0575.

5,5,5-Trifluoro-4-hydroxy-2-methylene-1-(thiophen-2-yl)pentan-1-one (9o)

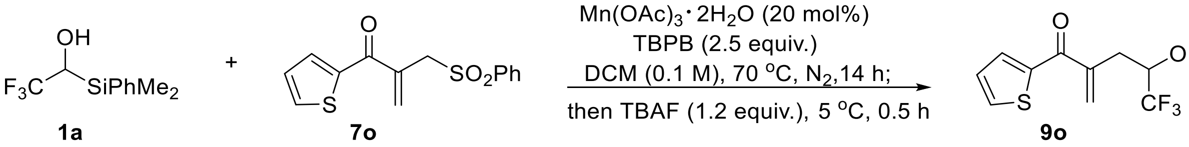

Under N_2 atmosphere, to a dried 10 mL Schlenk tube equipped with a magnetic stir bar containing $Mn(OAc)_3$•$2H_2O$ (16.1 mg, 0.06 mmol, 20 mol%), **7o** (261.0 mg, 0.9 mmol, 3.0 equiv.) was added DCM (3 mL, 0.1 M), **1a** (70.2 mg, 0.3

mmol) and TBPB (145.7 mg, 0.75 mmol, 2.5 equiv.) sequentially. The tube was then sealed, and the resulting mixture was stirred at 70 °C in a heating block for 14 h, after which the mixture was cooled to 5 °C with ice bath. TBAF (1.0 M in THF, 0.36 mL, 0.36 mmol, 1.2 equiv.) was added and the mixture was stirred at 5 °C for 0.5 h. The reaction mixture was quenched with water (2 mL) and extracted with DCM (3×10 mL). The organic phase was combined and washed with brine, dried over Na_2SO_4, concentrated under reduced pressure. The crude product was purified with column chromatography on silica gel (200~300 mesh) and PE/EA (20/1~10/1, v/v) as eluent to afford 61.4 mg of the title compound as a yellow solid (82% yield).

R_f = 0.40 (PE/EA = 5/1, v/v). mp: 42 °C–44 °C (from PE and EA). ^{1}H NMR (400 MHz, $CDCl_3$) δ 7.77–7.72 (m, 2H), 7.18–7.16 (m, 1H), 6.10 (s, 1H), 6.05 (s, 1H), 4.46 (s, 1H), 4.14–4.11 (m, 1H), 2.85–2.66 (m, 2H); ^{13}C NMR (100 MHz, $CDCl_3$) δ 190.6, 142.4, 142.2, 135.8, 135.6, 129.3, 128.4, 124.9 (q, J = 280.8 Hz), 70.7 (q, J = 30.7 Hz), 34.1; ^{19}F NMR (375 MHz, $CDCl_3$) δ –79.3 (d, J = 6.0 Hz, 3F). IR (ATR): 3418, 3101, 2930, 1614, 1513, 1413, 1357, 1275, 1163, 1129, 1055, 727 cm^{-1}. HRMS (ESI, m/z): calcd for $C_{10}H_{10}F_3O_2S^+$ $(M+H)^+$: 251.0348; Found: 251.0341.

5,5,5-Trifluoro-4-hydroxy-2-methylene-1-(naphthalen-2-yl)pentan-1-one (9p)

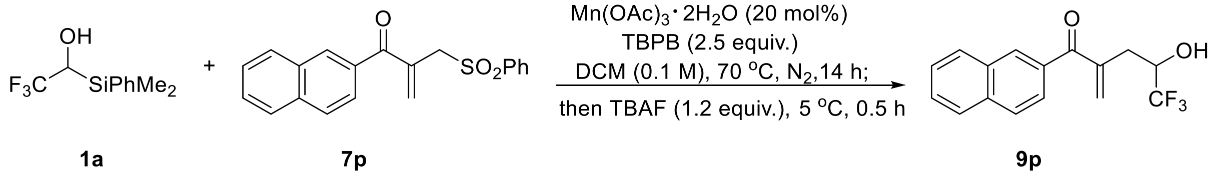

Under N_2 atmosphere, to a dried 10 mL Schlenk tube equipped with a magnetic stir bar containing $Mn(OAc)_3$•$2H_2O$ (16.1 mg, 0.06 mmol, 20 mol%), **7p** (302.4 mg, 0.9 mmol, 3.0 equiv.) was added DCM (3 mL, 0.1 M), **1a** (70.2 mg, 0.3 mmol) and TBPB (145.7 mg, 0.75 mmol, 2.5 equiv.) sequentially. The tube was then sealed, and the resulting mixture was stirred at 70 °C in a heating block for 14 h, after which the mixture was cooled to 5 °C with ice bath. TBAF (1.0 M in THF, 0.36 mL, 0.36 mmol, 1.2 equiv.) was added and the mixture was stirred at 5 °C for 0.5 h. The reaction mixture was quenched with water (2 mL) and extracted with DCM (3×10 mL). The organic phase was combined and washed with brine, dried over Na_2SO_4, concentrated under reduced pressure. The crude product was purified with column chromatography on silica gel (200~300 mesh) and PE/EA (20/1~10/1, v/v) as eluent to afford 63.0 mg of the title compound as a white solid (72% yield).

R_f = 0.40 (PE/EA = 5/1, v/v). mp: 42 °C–44 °C (from PE and EA). ^{1}H NMR (400 MHz, $CDCl_3$) δ 8.30 (s, 1H), 7.95–7.85 (m, 4H), 7.65–7.55 (m, 2H), 6.21 (s, 1H), 5.97 (s, 1H), 4.42 (d, J = 5.2 Hz, 1H), 4.25–4.15 (m, 1H), 2.96–2.77 (m, 2H); ^{13}C NMR (100 MHz, $CDCl_3$) δ 199.5, 142.4, 135.7, 133.9, 132.2, 132.1, 131.7, 129.6, 128.8, 128.7, 128.0, 127.1, 125.5, 125.0 (q, J = 280.7 Hz), 70.6 (q, J = 30.9 Hz), 34.0; ^{19}F NMR (375 MHz, $CDCl_3$) δ –79.3 (d, J = 6.0 Hz, 3F). IR (ATR): 3411, 3060, 2933, 1644, 1469, 1357, 1275, 1167, 1126, 1033, 746 cm^{-1}. HRMS (ESI, m/z): calcd for $C_{16}H_{14}F_3O_2^+$ $(M+H)^+$: 295.0940; Found: 295.0932.

N,N-diphenyl 5,5,5-trifluoro-4-hydroxy-2-methylenepentanamide (9q)

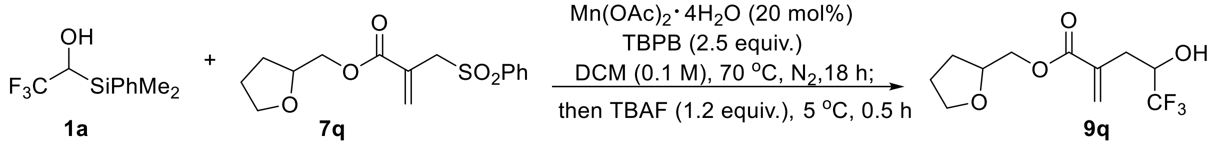

Under N_2 atmosphere, to a dried 10 mL Schlenk tube equipped with a magnetic stir bar containing $Mn(OAc)_2$•$4H_2O$ (14.7 mg, 0.06 mmol, 20 mol%), **7q** (279.0 mg, 0.9 mmol, 3.0 equiv.) was added DCM (3 mL, 0.1 M), **1a** (70.2 mg, 0.3 mmol) and TBPB (145.7 mg, 0.75 mmol, 2.5 equiv.) sequentially. The tube was sealed, and the resulting mixture was kept stirring at 70 °C in heating block for 18 h. After which the mixture was cooled to 5 °C with ice bath, TBAF (1.0 M in THF, 0.36 mL, 0.36 mmol, 1.2 equiv.) was added and the mixture was stirred at 5 °C for 0.5 h. The reaction mixture was quenched with water (2 mL), extracted with DCM (3×10 mL) and organic phase was combined and washed with brine, dried over Na_2SO_4, concentrated under reduced pressure. The crude product was purified with column chromatography on silica gel (200~300 mesh) and PE/EA (8/1, v/v) as eluent to afford 46.0 mg of the title compound as

a colorless oil (57% yield).

R_f = 0.24 (PE/EA = 4/1, v/v). NMR Spectroscopy: ^1H NMR (400 MHz, $CDCl_3$) δ 6.39 (s, 1H), 5.80 (s, 1H), 4.31–4.09 (m, 4H), 3.92–3.78 (m, 2H), 2.78–2.61 (m, 2H), 2.08–1.92 (m, 3H), 1.69–1.62 (m, 1H); ^{13}C NMR (100 MHz, $CDCl_3$) δ 167.6, 135.1, 130.2, 125.0 (q, *J* = 281.0 Hz), 76.5, 76.4 (C'), 70.1 (q, *J* = 30.7 Hz, C), 70.1 (q, *J* = 30.7 Hz, C'), 68.6, 68.6 (C'), 67.4, 67.3 (C'), 33.6, 28.0, 25.8; ^{19}F NMR (375 MHz, $CDCl_3$) δ –79.6 (d, *J* = 6.0 Hz, 3F), –79.7 (d, *J* = 6.0 Hz, 3F'). IR (ATR): 3384, 2956, 2878, 1715, 1633, 1413, 1316, 1275, 1167, 1126, 1021, 712 cm^{-1}. HRMS (ESI, m/z): calcd for $C_{11}H_{15}F_3NaO_4^+$ $(M+Na)^+$: 290.0815; Found: 290.0810.

1-Methoxypropan-2-yl 5,5,5-trifluoro-4-hydroxy-2-methylenepentanoate (9r)

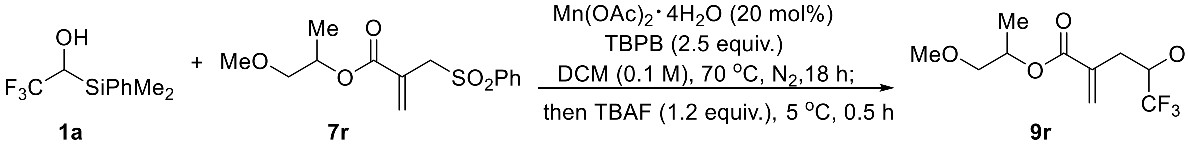

Under N_2 atmosphere, to a dried 10 mL Schlenk tube equipped with a magnetic stir bar containing $Mn(OAc)_2•4H_2O$ (24.5 mg, 0.1 mmol, 20 mol%), **7r** (298.4 mg, 1.5 mmol, 3.0 equiv.) was added DCM (5 mL, 0.1 M), **1a** (117.1 mg, 0.5 mmol) and TBPB (242.7 mg, 1.5 mmol, 2.5 equiv.) sequentially. The tube was then sealed, and the resulting mixture was stirred at 70 °C in a heating block for 18 h, after which the mixture was cooled to 5 °C with ice bath. TBAF (1.0 M in THF, 0.60 mL, 0.60 mmol, 1.2 equiv.) was added and the mixture was stirred at 5 °C for 0.5 h. The reaction mixture was quenched with water (2 mL) and extracted with DCM (3×10 mL). The organic phase was combined and washed with brine, dried over Na_2SO_4, concentrated under reduced pressure. The crude product was purified with column chromatography on silica gel (200~300 mesh) and PE/EA (20/1~10/1, v/v) as eluent to afford 93.0 mg of the title compound as a colorless oil (73% yield).

R_f = 0.40 (PE/EA = 5/1, v/v). ^1H NMR (400 MHz, $CDCl_3$) δ 6.35–6.34 (m, 1H), 5.77–5.74 (m, 1H), 5.19–5.10 (m, 1H), 4.20–4.00 (m, 1H), 3.52–3.36 (m, 6H), 2.88–2.81 (m, 2H), 1.28 (d, *J* = 6.4 Hz, 3H); ^{13}C NMR (100 MHz, $CDCl_3$) δ 167.3, 167.2 (C'), 135.8, 135.3 (C'), 130.0, 129.4 (C'), 125.0 (q, *J* = 281.5 Hz), 75.0, 75.0 (C'), 70.8, 70.7, 70.6 (q, *J* = 28.7 Hz), 69.7 (q, *J* = 29.2 Hz, C'), 59.2, 59.1 (C'), 33.6, 33.3 (C'), 16.5, 16.5 (C'); ^{19}F NMR (375 MHz, $CDCl_3$) δ –77.0 (d, *J* = 8.9 Hz, 3F), –77.4 (d, *J* = 8.9 Hz, 3F'). IR (ATR): 3422, 2986, 2937, 2889, 1711, 1633, 1454, 1275, 1170, 1126, 1033, 708 cm^{-1}. HRMS (ESI, m/z): calcd for $C_{10}H_{15}F_3NaO_4^+$ $(M+Na)^+$: 279.0814; Found: 279.0808.

2,3-Dihydro-1H-inden-1-yl 5,5,5-trifluoro-4-hydroxy-2-methylenepentanoate (9s)

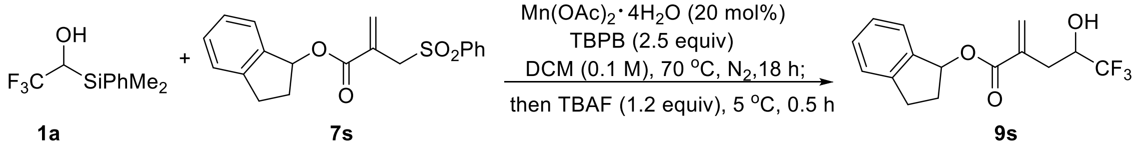

Under N_2 atmosphere, to a dried 10 mL Schlenk tube equipped with a magnetic stir bar containing $Mn(OAc)_2•4H_2O$ (14.7 mg, 0.06 mmol, 20 mol%), **7s** (205.2 mg, 0.6 mmol, 2.0 equiv.) was added DCM (3 mL, 0.1 M), **1a** (70.2 mg, 0.3 mmol) and TBPB (145.7 mg, 0.75 mmol, 2.5 equiv.) sequentially. The tube was sealed, and the resulting mixture was kept stirring at 70 °C in heating block for 18 h. After which the mixture was cooled to 5 °C with ice bath, TBAF (1.0 M in THF, 0.36 mL, 0.36 mmol, 1.2 equiv.) was added and the mixture was stirred at 5 °C for 0.5 h. The reaction mixture was quenched with water (2 mL), extracted with DCM (3×10 mL) and organic phase was combined and washed with brine, dried over Na_2SO_4, concentrated under reduced pressure. The crude product was purified with column chromatography on silica gel (200~300 mesh) and PE/EA (20/1, v/v) as eluent to afford 66.0 mg of the title compound as a colorless oil (73 % yield).

R_f = 0.30 (PE/EA = 8/1, v/v). NMR Spectroscopy: ^1H NMR (400 MHz, $CDCl_3$) δ 7.43 (d, *J* = 7.3 Hz, 1H), 7.35–7.31 (m, 2H), 7.25 (t, *J* = 6.3 Hz, 1H), 6.32–6.28 (m, 2H), 5.79 (s, 1H), 4.14–4.12 (m, 1H), 3.67 (s, 1H), 3.18–3.11 (m, 1H), 2.96–2.89 (m, 1H), 2.80–2.76 (m, 1H), 2.66–2.52 (m, 2H), 2.20–2.13 (m, 1H); ^{13}C NMR (100 MHz, $CDCl_3$) δ 168.1, 144.6, 140.6, 135.3, 130.1, 130.0 (C'), 129.4, 127.0, 125.8, 125.7 (C'), 125.0, 124.9 (q, *J* = 280.8 Hz), 79.9, 71.2 (q, *J* = 30.9 Hz, C), 70.1 (q, *J* = 31.2 Hz, C'), 33.6, 32.4, 30.3; ^{19}F NMR (375 MHz, $CDCl_3$) δ –79.5– –79.5 (m, 3F, 3F'). IR

(ATR): 3425, 2941, 2855, 1703, 1633, 1435, 1320, 1275, 1170, 1126, 1014, 708 cm^{-1}. HRMS (ESI, m/z): calcd for $C_{15}H_{15}F_3NaO_3^+$ $(M+Na)^+$: 323.0866; Found: 323.0867.

Benzo[d][1,3]dioxol-5-ylmethyl 5,5,5-trifluoro-4-hydroxy-2-methylenepentanoate (9t)

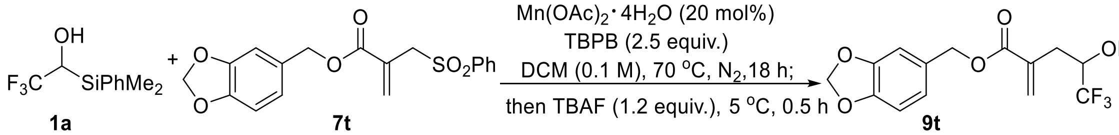

1a **7t** **9t**

Under N_2 atmosphere, to a dried 10 mL Schlenk tube equipped with a magnetic stir bar containing $Mn(OAc)_2$•$4H_2O$ (14.7 mg, 0.06 mmol, 20 mol%), **7t** (216 mg, 0.6 mmol, 2.0 equiv.) was added DCM (3 mL, 0.1 M), **1a** (70.2 mg, 0.3 mmol) and TBPB (145.7 mg, 0.75 mmol, 2.5 equiv.) sequentially. The tube was sealed, and the resulting mixture was kept stirring at 70 ºC in heating block for 18 h. After which the mixture was cooled to 5 ºC with ice bath, TBAF (1.0 M in THF, 0.36 mL, 0.36 mmol, 1.2 equiv.) was added and the mixture was stirred at 5 ºC for 0.5 h. The reaction mixture was quenched with water (2 mL), extracted with DCM (3×10 mL) and organic phase was combined and washed with brine, dried over Na_2SO_4, concentrated under reduced pressure. The crude product was purified with column chromatography on silica gel (200~300 mesh) and PE/EA (8/1, v/v) as eluent to afford 61.0 mg of the title compound as a colorless oil (64% yield).

R_f = 0.33 (PE/EA = 4/1, v/v). NMR Spectroscopy: ^{1}H NMR (400 MHz, $CDCl_3$) δ 6.85–6.77 (m, 3H), 6.35 (s, 1H), 5.96 (s, 2H), 5.81 (s, 1H), 5.11 (s, 2H), 4.15–4.07 (m, 1H), 3.10 (s, 1H), 2.78–2.56 (m, 2H); ^{13}C NMR (100 MHz, $CDCl_3$) δ 167.7, 148.0, 147.8, 135.0, 130.2, 129.2, 124.9 (q, J = 280.0 Hz), 122.5, 109.1, 108.4, 101.4, 69.9 (q, J = 31.1 Hz), 67.4, 33.5; ^{19}F NMR (375 MHz, $CDCl_3$) δ −79.6 (d, J = 6.0 Hz, 3F). IR (ATR): 3422, 2900, 1707, 1633, 1491, 1446, 1327, 1252, 1167, 1122, 1036, 712 cm^{-1}. HRMS (ESI, m/z): calcd for $C_{14}H_{13}F_3NaO_5^+$ $(M+Na)^+$: 341.0607; Found: 341.0594.

2-Iodobenzyl 5,5,5-trifluoro-4-hydroxy-2-methylenepentanoate (9u)

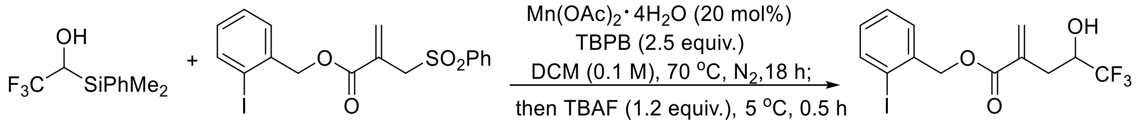

1a **7u** **9u**

Under N_2 atmosphere, to a dried 10 mL Schlenk tube equipped with a magnetic stir bar containing $Mn(OAc)_2$•$4H_2O$ (14.7 mg, 0.06 mmol, 20 mol%), **7u** (406.8 mg, 0.9 mmol, 3.0 equiv.) was added DCM (3 mL, 0.1 M), **1a** (70.2 mg, 0.3 mmol) and TBPB (145.7 mg, 0.75 mmol, 2.5 equiv.) sequentially. The tube was sealed, and the resulting mixture was kept stirring at 70 ºC in heating block for 18 h. After which the mixture was cooled to 5 ºC with ice bath, TBAF (1.0 M in THF, 0.36 mL, 0.36 mmol, 1.2 equiv.) was added and the mixture was stirred at 5 ºC for 0.5 h. The reaction mixture was quenched with water (2 mL), extracted with DCM (3×10 mL) and organic phase was combined and washed with brine, dried over Na_2SO_4, concentrated under reduced pressure. The crude product was purified with column chromatography on silica gel (200~300 mesh) and PE/EA (12/1, v/v) as eluent to afford 83.0 mg of the title compound as a colorless oil (68 % yield).

R_f = 0.31 (PE/EA = 8/1 v/v). NMR Spectroscopy: ^{1}H NMR (400 MHz, $CDCl_3$) δ 7.87 (d, J = 7.6 Hz, 1H), 7.39–7.34 (m, 2n), 7.06–7.02 (m, 1H), 6.44 (s, 1H), 5.85 (s, 1H), 5.24 (s, 2H), 4.18–4.12 (m, 1H), 3.22 (s, 1H), 2.82–2.59 (m, 2H); ^{13}C NMR (100 MHz, $CDCl_3$) δ 167.4, 139.8, 137.9, 134.7, 130.7, 130.3, 130.0, 128.6, 124.9 (q, J = 280.7 Hz), 98.7, 71.0, 69.9 (q, J = 69.9 Hz), 33.5; ^{19}F NMR (375 MHz, $CDCl_3$) δ −79.5 (d, J = 6.0 Hz, 3F). IR (ATR): 3291, 2919, 2848, 1707, 1633, 1439, 1327, 1275, 1167, 1122, 1036, 712 cm^{-1}. HRMS (ESI, m/z): calcd for $C_{13}H_{12}F_3INaO_3^+$ $(M+Na)^+$: 422.9675; Found: 422.9664.

(4-Bromophenyl)methyl 5,5,5-trifluoro-4-hydroxy-2-methylenepentanoate (9v)

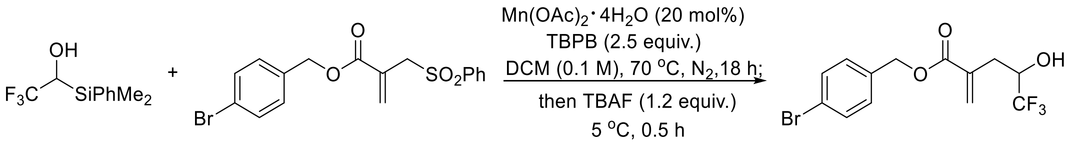

1a **7v** **9v**

Under N_2 atmosphere, to a dried 10 mL Schlenk tube equipped with a magnetic stir bar containing with $Mn(OAc)_2{\bullet}4H_2O$ (14.7 mg, 0.06 mmol, 20 mol%), **7v** (355.5 mg, 0.9 mmol, 3.0 equiv.) was added DCM (3 mL, 0.1 M), **1a** (70.2 mg, 0.3 mmol) and TBPB (145.7 mg, 0.75 mmol, 2.5 equiv.) sequentially. The tube was sealed, and the resulting mixture was kept stirring at 70 °C in heating block for 18 h. After which the mixture was cooled to 5 °C with ice bath, TBAF (1.0 M in THF, 0.36 mL, 0.36 mmol, 1.2 equiv.) was added and the mixture was stirred at 5 °C for 0.5 h. The reaction mixture was quenched with water (2 mL), extracted with DCM (3×10 mL) and organic phase was combined and washed with brine, dried over Na_2SO_4, concentrated under reduced pressure. The crude product was purified with column chromatography on silica gel (200~300 mesh) and PE/EA (8/1, v/v) as eluent to afford 60.0 mg of the title compound as a colorless oil (56% yield).

R_f = 0.23 (PE/EA = 4/1, v/v). NMR Spectroscopy: ^{1}H NMR (400 MHz, $CDCl_3$) δ 7.51 (d, J = 8.3 Hz, 2H), 7.25 (d, J = 8.4 Hz, 2H), 6.39 (s, 1H), 5.85 (s, 1H), 5.17 (s, 2H), 4.17–4.09 (m, 1H), 2.81–2.59 (m, 3H); ^{13}C NMR (100 MHz, $CDCl_3$) δ 167.6, 134.8, 134.5, 132.0, 130.4, 130.1, 124.9 (q, J = 279.8 Hz), 122.7, 69.9 (q, J = 31.1 Hz), 66.6, 33.4; ^{19}F NMR (375 MHz, $CDCl_3$) δ –79.6 (d, J = 6.0 Hz, 3F). IR (ATR): 3422, 3528, 2498, 1715, 1633, 1439, 1331, 1275, 1170, 1126, 1014, 712 cm^{-1}. HRMS (ESI, m/z): calcd for $C_{13}H_{12}BrF_3NaO_3^+$ $(M+Na)^+$: 374.9814; Found:374.9807.

Naphthalen-1-ylmethyl 5,5,5-trifluoro-4-hydroxy-2-methylenepentanoate (9w)

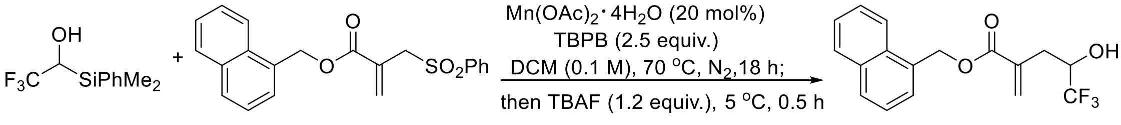

1a **7w** **9w**

Under N_2 atmosphere, to a dried 10 mL Schlenk tube equipped with a magnetic stir bar containing $Mn(OAc)_2{\bullet}4H_2O$ (14.7 mg, 0.06 mmol, 20 mol%), **7w** (329.8 mg, 0.9 mmol, 3.0 equiv.) was added DCM (3 mL, 0.1 M), **1a** (70.2 mg, 0.3 mmol)and TBPB (145.7 mg, 0.75 mmol, 2.5 equiv.) sequentially. The tube was then sealed, and the resulting mixture was stirred at 70 °C in a heating block for 14 h, after which the mixture was cooled to 5 °C with ice bath. TBAF (1.0 M in THF, 0.36 mL, 0.36 mmol, 1.2 equiv.) was added and the mixture was stirred at 5 °C for 0.5 h. The reaction mixture was quenched with water (2 mL) and extracted with DCM (3×10 mL). The organic phase was combined and washed with brine, dried over Na_2SO_4, concentrated under reduced pressure. The crude product was purified with column chromatography on silica gel (200~300 mesh) and PE/EA (20/1~10/1, v/v) as eluent to afford 70.0 mg of the title compound as a colorless oil (72% yield).

R_f = 0.3 (PE/EA = 10/1, v/v). ^{1}H NMR (400 MHz, $CDCl_3$) δ 8.03 (d, J = 7.9 Hz, 1H), 7.89 (t, J = 8.9 Hz, 2H), 7.60–7.52 (m, 3H), 7.47 (t, J = 7.5 Hz, 1H), 6.35 (s, 1H), 5.80 (s, 1H), 5.70 (s, 2H), 4.17–4.09 (m, 1H), 3.31 (s, 1H), 2.81–2.59 (m, 2H); ^{13}C NMR (100 MHz, $CDCl_3$) δ 167.8, 134.9, 133.9, 131.8, 131.0, 130.5, 129.7, 128.95, 127.87, 126.86, 126.19, 125.40, 124.9 (q, J = 280.8 Hz), 123.5, 69.9 (q, J = 30.7 Hz), 65.8, 33.5; ^{19}F NMR (375 MHz, $CDCl_3$) δ –79.6 (d, J = 6.0 Hz, 3F). IR(ATR): 3444, 3049, 2937, 1707, 1633, 1413, 1320, 1271, 1167, 1126, 1029, 775 cm^{-1}. HRMS (ESI, m/z): calcd for $C_{17}H_{15}F_3NaO_3^+$ $(M+Na)^+$: 347.0866; Found: 347.0875.

3-(Cyclohexyl)propenyl 5,5,5-trifluoro-4-hydroxy-2-methylenepentanoate (9x)

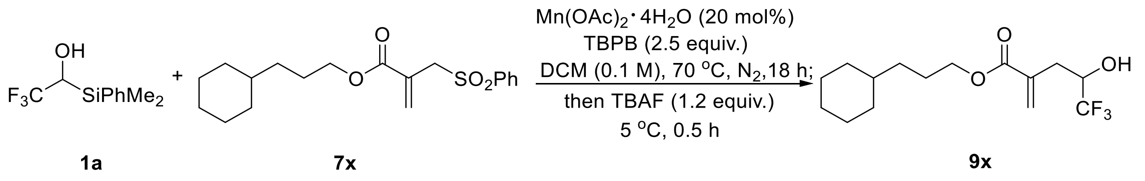

Under N_2 atmosphere, to a dried 10 mL Schlenk tube equipped with a magnetic stir bar containing $Mn(OAc)_2$•$4H_2O$ (14.7 mg, 0.06 mmol, 20 mol%), **7x** (210.0 mg, 0.6 mmol, 2.0 equiv.) was added DCM (3 mL, 0.1 M), **1a** (70.2 mg, 0.3 mmol) and TBPB (145.7 mg, 0.75 mmol, 2.5 equiv.) sequentially. The tube was sealed, and the resulting mixture was kept stirring at 70 °C in heating block for 18 h. After which the mixture was cooled to 5 °C with ice bath, TBAF (1.0 M in THF, 0.36 mL, 0.36 mmol, 1.2 equiv.) was added and the mixture was stirred at 5 °C for 0.5 h. The reaction mixture was quenched with water (2 mL), extracted with DCM (3×10 mL) and organic phase was combined and washed with brine, dried over Na_2SO_4, concentrated under reduced pressure. The crude product was purified with column chromatography on silica gel (200~300 mesh) and PE/EA (15/1, v/v) as eluent to afford 61.0 mg of the title compound as a colorless oil (66% yield).

R_f = 0.54 (PE/EA = 4/1, v/v). NMR Spectroscopy: ^{1}H NMR (400 MHz, $CDCl_3$) δ 6.34 (s, 1H), 5.80 (s, 1H), 4.18–4.08 (m, 3H), 2.79–2.58 (m, 3H), 1.71–1.64 (m, 7H), 1.26–1.12 (m, 6H), 0.92–0.87 (m, 2H); ^{13}C NMR (100 MHz, $CDCl_3$) δ 168.2, 135.3, 129.8, 124.9 (q, J = 280.5 Hz), 70.1 (q, J = 31.1 Hz), 66.3, 37.4, 33.7, 33.6, 33.4, 26.7, 26.4, 26.0; ^{19}F NMR (375 MHz, $CDCl_3$) δ –79.6 (d, J = 6.0 Hz, 3F). IR (ATR): 3422, 2922, 2851, 1711, 1633, 1413, 1320, 1275, 1174, 1129, 1033, 712 cm^{-1}. HRMS (ESI, m/z): calcd for $C_{15}H_{24}F_3O_3^+$ $(M+H)^+$: 309.1672; Found: 309.1665.

Adamantan-2-yl 5,5,5-trifluoro-4-hydroxy-2-methylenepentanoate (9y)

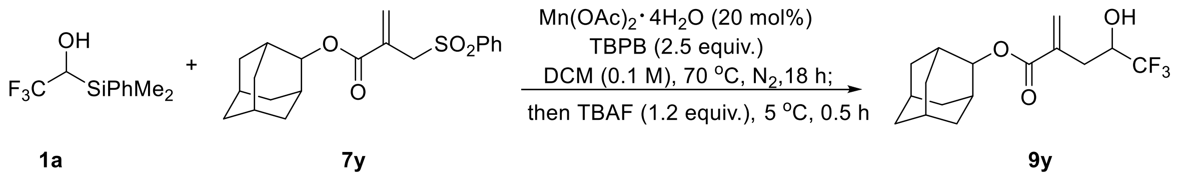

Under N_2 atmosphere, to a dried 10 mL Schlenk tube equipped with a magnetic stir bar containing $Mn(OAc)_2$•$4H_2O$ (14.7 mg, 0.06 mmol, 20 mol%), **7y** (216.0 mg, 0.6 mmol, 2.0 equiv.) was added DCM (3 mL, 0.1 M), **1a** (70.2 mg, 0.3 mmol) and TBPB (145.7 mg, 0.75 mmol, 2.5 equiv.) sequentially. The tube was sealed, and the resulting mixture was kept stirring at 70 °C in heating block for 18 h. After which the mixture was cooled to 5 °C with ice bath, TBAF (1.0 M in THF, 0.36 mL, 0.36 mmol, 1.2 equiv.) was added and the mixture was stirred at 5 °C for 0.5 h. The reaction mixture was quenched with water (2 mL), extracted with DCM (3×10 mL) and organic phase was combined and washed with brine, dried over Na_2SO_4, concentrated under reduced pressure. The crude product was purified with column chromatography on silica gel (200~300 mesh) and PE/EA (20/1, v/v) as eluent to afford 78.0 mg of the title compound as a colorless oil (82 % yield).

R_f = 0.43 (PE/EA = 8/1, v/v). NMR Spectroscopy: ^{1}H NMR (400 MHz, $CDCl_3$) δ 6.40 (s, 1H), 5.81 (s, 1H), 5.03 (s, 1H), 4.15–4.12 (m, 1H), 3.66 (s, 1H), 2.81–2.60 (m, 2H), 2.06–2.00 (m, 4H), 1.90–1.76 (m, 8H), 1.63–1.60 (m, 2H); ^{13}C NMR (100 MHz, $CDCl_3$) δ 167.5, 135.9, 129.5, 125.0 (q, J = 280.8 Hz), 78.7, 70.2 (q, J = 30.7 Hz), 37.4, 36.4, 33.6, 32.1, 32.0, 27.3, 27.1; ^{19}F NMR (375 MHz, $CDCl_3$) δ –79.5 (d, J = 6.0 Hz, 3F). IR (ATR): 3444, 2907, 2855, 1696, 1633, 1413, 1316, 1275, 1174, 1129, 1044, 712 cm^{-1}. HRMS (ESI, m/z): calcd for $C_{16}H_{21}F_3NaO_3^+$ $(M+Na)^+$: 341.1335; Found: 341.1330.

N,N-diphenyl 5,5,5-trifluoro-4-hydroxy-2-methylenepentanamide (9z)

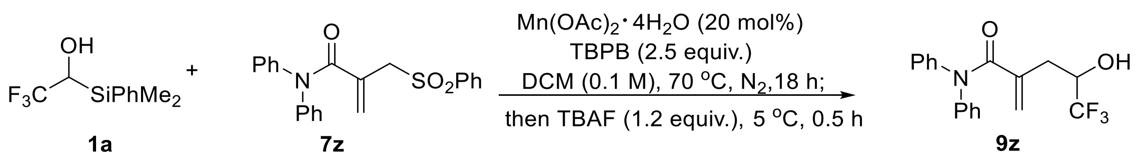

Under N_2 atmosphere, to a dried 10 mL Schlenk tube equipped with a magnetic stir bar containing $Mn(OAc)_2$•$4H_2O$ (14.7 mg, 0.06 mmol, 20 mol%), **7z** (339.3 mg, 0.9 mmol, 3.0 equiv.) was added DCM (3 mL, 0.1 M), **1a** (70.2 mg, 0.3 mmol) and TBPB (145.7 mg, 0.75 mmol, 2.5 equiv.) sequentially. The tube was sealed, and the resulting mixture was kept stirring at 70 °C in heating block for 18 h. After which the mixture was cooled to 5 °C with ice bath, TBAF (1.0 M in THF, 0.36 mL, 0.36 mmol, 1.2 equiv.) was added and the mixture was stirred at 5 °C for 0.5 h. The reaction mixture was quenched with water (2 mL), extracted with DCM (3×10 mL) and organic phase was combined and washed with brine, dried over Na_2SO_4, concentrated under reduced pressure. The crude product was purified with column chromatography on silica gel (200~300 mesh) and PE/EA (8/1, v/v) as eluent to afford 54.0 mg of the title compound as a colorless oil (54% yield).

R_f = 0.27 (PE/EA = 4/1, v/v). NMR Spectroscopy: ^{1}H NMR (400 MHz, $CDCl_3$) δ 7.37 (t, J = 7.8 Hz, 4H), 7.29–7.25 (m, 2H), 7.19–7.17 (m, 4H), 5.45 (s, 1H), 5.32 (s, 1H), 4.15–4.07 (m, 1H), 2.64–2.50 (m, 2H); ^{13}C NMR (100 MHz, $CDCl_3$) δ 172.4, 143.2, 138.6, 129.5, 127.3, 127.2, 125.8, 125.0 (q, J = 280.3 Hz), 71.1 (q, J = 30.7 Hz), 34.8; ^{19}F NMR (375 MHz, $CDCl_3$) δ –79.5 (d, J = 6.0 Hz, 3F). IR (ATR): 3288, 2963, 2930, 1644, 1592, 1491, 1364, 1275, 1163, 1126, 1029, 693 cm^{-1}. HRMS (ESI, m/z): calcd for $C_{18}H_{17}F_3NO_2^+$ $(M+H)^+$: 336.1206; Found: 336.1197.

5,5,5-Trifluoro-4-hydroxy-N,N-dimethyl-2-methylenepentanamide (9aa)

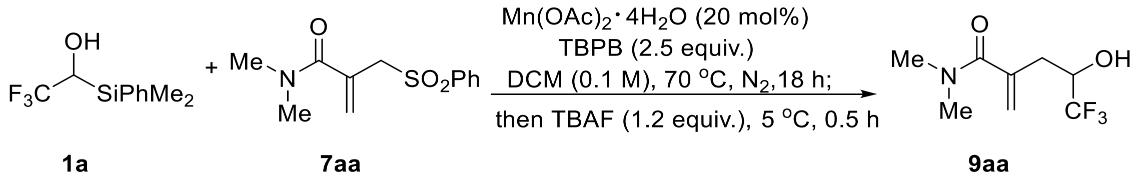

Under N_2 atmosphere, to a dried 10 mL Schlenk tube equipped with a magnetic stir bar containing $Mn(OAc)_2$•$4H_2O$ (14.7 mg, 0.06 mmol, 20 mol%), **7aa** (227.9 mg, 0.9 mmol, 3.0 equiv.) was added DCM (3 mL, 0.1 M), **1a** (70.2 mg, 0.3 mmol) and TBPB (145.7 mg, 0.75 mmol, 2.5 equiv.) sequentially. The tube was then sealed, and the resulting mixture was stirred at 70 °C in a heating block for 14 h, after which the mixture was cooled to 5 °C with ice bath. TBAF (1.0 M in THF, 0.36 mL, 0.36 mmol, 1.2 equiv.) was added and the mixture was stirred at 5 °C for 0.5 h. The reaction mixture was quenched with water (2 mL) and extracted with DCM (3×10 mL). The organic phase was combined and washed with brine, dried over Na_2SO_4, concentrated under reduced pressure. The crude product was purified with column chromatography on silica gel (200~300 mesh) and PE/EA (20/1~10/1, v/v) as eluent to afford 40.0 mg of the title compound as a colorless oil (63% yield).

R_f = 0.30 (PE/EA = 2/1, v/v). ^{1}H NMR (400 MHz, $CDCl_3$) δ 5.58 (s, 1H), 5.37 (s, 1H), 4.10–4.02 (m, 1H), 3.14 (s, 3H), 3.03 (s, 3H), 2.64–2.41 (m, 2H); ^{13}C NMR (100 MHz, $CDCl_3$) δ 172.4, 137.6, 125.1 (q, J = 280.3 Hz), 121.8, 71.1 (q, J = 30.7 Hz), 39.8, 35.5, 34.8; ^{19}F NMR (375 MHz, $CDCl_3$) δ –79.4 (d, J = 6.0 Hz, 3F). IR (ATR): 3329, 2930, 1610, 1454, 1264, 1167, 1118, 1029, 734 cm^{-1}. HRMS (ESI, m/z): calcd for $C_8H_{12}F_3NNaO_2^+$ $(M+Na)^+$: 234.0712; Found: 234.0707.

1,1,1-Trifluoro-4-tosylpent-4-en-2-ol (9ab)

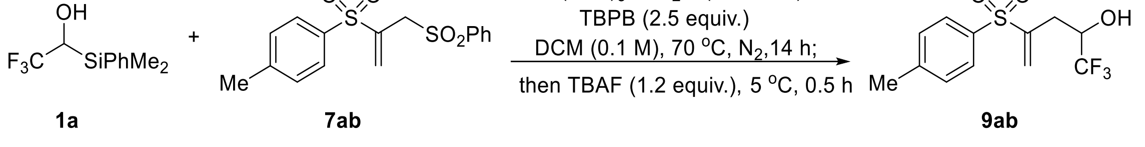

Under N_2 atmosphere, to a dried 10 mL Schlenk tube equipped with a magnetic stir bar containing $Mn(OAc)_3$•$2H_2O$ (16.1 mg, 0.06 mmol, 20 mol%), **7ab** (302.7 mg, 0.9 mmol, 3.0 equiv.) was added DCM (3 mL, 0.1 M), **1a** (70.2 mg, 0.3 mmol) and TBPB (145.7 mg, 0.75 mmol, 2.5 equiv.) sequentially. The tube was then sealed, and the resulting mixture was stirred at 70 °C in a heating block for 14 h, after which the mixture was cooled to 5 °C with ice bath. TBAF (1.0 M in THF, 0.36 mL, 0.36 mmol, 1.2 equiv.) was added and the mixture was stirred at 5 °C for 0.5 h. The reaction mixture was quenched with water (2 mL) and extracted with DCM (3×10 mL). The organic phase was combined and washed with brine, dried over Na_2SO_4, concentrated under reduced pressure. The crude product was purified with column

chromatography on silica gel (200~300 mesh) and PE/EA (20/1~10/1, v/v) as eluent to afford 63.5 mg of the title compound as a colorless oil (72% yield).

R_f = 0.30 (PE/EA = 5/1, v/v). ^{1}H NMR (400 MHz, $CDCl_3$) δ 7.76 (d, J = 8.4 Hz, 2H), 7.37 (d, J = 8.0 Hz, 2H), 6.47 (s, 1H), 5.96 (s, 1H), 4.30–4.22 (m, 1H), 2.63–2.46 (m, 5H); ^{13}C NMR (100 MHz, $CDCl_3$) δ 145.5, 145.0, 134.7, 130.3, 128.6, 128.3, 124.6 (q, J = 277.9 Hz), 69.1 (q, J =31.4 Hz), 31.2, 21.8; ^{19}F NMR (375 MHz, $CDCl_3$) δ –79.7 (d, J = 6.0 Hz, 3F). IR(ATR): 3474, 2930, 2855, 1595, 1431, 1279, 1137, 1081, 734 cm^{-1}. HRMS (ESI, m/z): calcd for $C_{12}H_{13}F_3NaO_3S^+$ $(M+Na)^+$: 317.0430; Found: 317.0432.

1,1,1-Trifluoro-4-phenylpent-4-en-2-ol (9ac)

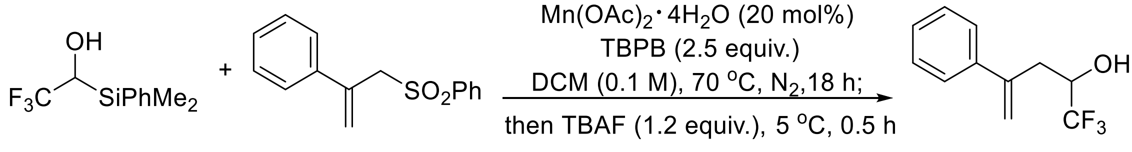

1a **7ac** **9ac**

Under N_2 atmosphere, to a dried 25 mL Schlenk tube equipped with a magnetic stir bar containing $Mn(OAc)_2$•$4H_2O$ (29.4 mg, 0.12 mmol, 20 mol%) and **7ac** (465.0 mg, 1.8 mmol, 3.0 equiv.) was added DCM (6 mL, 0.1 M), **1a** (140.4 mg, 0.6 mmol), and TBPB (291.3 mg, 1.5 mmol, 2.5 equiv.) sequentially. The tube was then sealed, and the resulting mixture was stirred at 70 °C in a heating block for 18 h, after which the mixture was cooled to 5 °C with ice bath. TBAF (1.0 M in THF, 0.72 mL, 0.72 mmol, 1.2 equiv.) was added and the mixture was stirred at 5 °C for 0.5 h. The reaction mixture was quenched with water (10 mL) and extracted with DCM (3×10 mL). The organic phase was combined and washed with brine, dried over Na_2SO_4, concentrated under reduced pressure. The crude product was purified with column chromatography on silica gel (200~300 mesh) and PE/EA (30/1~20/1, v/v) as eluent to afford 56.0 mg of the title compound as a colorless oil (43% yield).

R_f = 0.47 (PE/EA = 8/1, v/v). NMR Spectroscopy: ^{1}H NMR (400 MHz, $CDCl_3$) δ 7.42–7.31 (m, 5H), 5.49 (s, 1H), 5.28 (s, 1H), 4.00 (bs, 1H), 3.11–2.67 (m, 2H), 2.21 (s, 1H); ^{13}C NMR (100 MHz, $CDCl_3$) δ 142.7, 139.4, 128.8, 128.3, 126.3, 125.2 (q, J = 279.8 Hz), 121.1, 117.0, 68.7 (q, J = 30.9 Hz), 36.3; ^{19}F NMR (375 MHz, $CDCl_3$) δ –79.5 (d, J = 6.0 Hz, 3F). IR (ATR): 3422, 3086, 3030, 2960, 2930, 1633, 1446, 1390, 1029, 701 cm^{-1}. HRMS (ESI, m/z): calcd for $C_{11}H_{12}F_3O^+$ $(M+H)^+$: 217.0835; Found: 217.0828.

1,1,1-Trifluoro-5-methyl-5-phenylhex-4-en-2-ol (9ad)

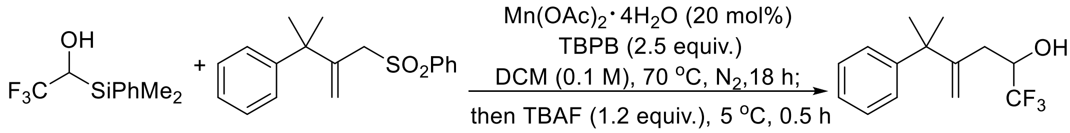

1a **7ad** **9ad**

Under N_2 atmosphere, to a dried 25 mL Schlenk tube equipped with a magnetic stir bar containing $Mn(OAc)_2$•$4H_2O$ (29.4 mg, 0.12 mmol, 20 mol%) and **7ad** (540.0 mg, 1.8 mmol, 3.0 equiv.) was added DCM (6 mL, 0.1 M), **1a** (140.4 mg, 0.6 mmol), and TBPB (291.3 mg, 1.5 mmol, 2.5 equiv.) sequentially. The tube was then sealed, and the resulting mixture was stirred at 70 °C in a heating block for 14 h, after which the mixture was cooled to 5 °C with ice bath. TBAF (1.0 M in THF, 0.72 mL, 0.72 mmol, 1.2 equiv.) was added and the mixture was stirred at 5 °C for 0.5 h. The reaction mixture was quenched with water (10 mL) and extracted with DCM (3×10 mL). The organic phase was combined and washed with brine, dried over Na_2SO_4, concentrated under reduced pressure. The crude product was purified with column chromatography on silica gel (200~300 mesh) and PE/EA (30/1~20/1, v/v) as eluent to afford 62.0 mg of the title compound as a colorless oil (40% yield).

R_f = 0.42 (PE/EA = 8/1, v/v). NMR Spectroscopy: ^{1}H NMR (400 MHz, $CDCl_3$) δ 7.32–7.31 (m, 4H), 7.24–7.19 (m, 1H), 5.37 (s, 1H), 5.15 (s, 1H), 3.81–3.73 (m, 1H), 2.28–2.00 (m, 2H), 1.79 (s, 1H), 1.48–1.47 (m, 6H); ^{13}C NMR (100 MHz, $CDCl_3$) δ 150.8, 146.9, 128.6, 126.5, 126.2, 125.0 (q, J = 280.3 Hz), 111.5, 69.4 (q, J = 30.7 Hz), 44.4, 32.9, 28.3; ^{19}F NMR (375 MHz, CDCl3) δ –80.0 (d, J = 6.0 Hz, 3F). IR (ATR): 3444, 2974, 2878, 1640, 1446, 1383, 1275, 1167, 1126, 1029, 701 cm^{-1}. HRMS (ESI, m/z): calcd for $C_{14}H_{17}F_3NaO^+$ $(M+Na)^+$: 281.1124; Found: 281.1121.

Cyclobutyl 5,5,5-trifluoro-4-hydroxy-2-methylenepentanoate (9ae)

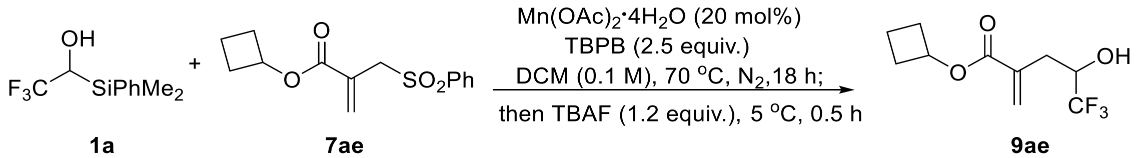

Under N_2 atmosphere, to a dried 10 mL Schlenk tube equipped with a magnetic stir bar containing $Mn(OAc)_2$•$4H_2O$ (14.7 mg, 0.06 mmol, 20 mol%), **4ae** (252.0 mg, 0.9 mmol, 3.0 equiv.) was added DCM (3 mL, 0.1 M), **3a** (70.2 mg, 0.3 mmol) and TBPB (145.7 mg, 0.75 mmol, 2.5 equiv.) sequentially. The tube was sealed, and the resulting mixture was kept stirring at 70 °C in heating block for 18 h. After which the mixture was cooled to 5 °C with ice bath, TBAF (1.0 M in THF, 0.36 mL, 0.36 mmol, 1.2 equiv.) was added and the mixture was stirred at 5 °C for 0.5 h. The reaction mixture was quenched with water (2 mL), extracted with DCM (3×10 mL) and organic phase was combined and washed with brine, dried over Na_2SO_4, concentrated under reduced pressure. The crude product was purified with column chromatography on silica gel (200~300 mesh) and PE/EA (15/1~ 8/1, v/v) as eluent to afford 50.0 mg of the title compound as a colorless oil (70% yield).

R_f = 0.36 (PE/EA = 8/1, v/v). NMR Spectroscopy: ^{1}H NMR (400 MHz, $CDCl_3$) δ 6.35 (s, 1H), 5.80 (s, 1H), 5.09–5.02 (m, 1H), 4.15–4.07 (m, 1H), 2.77–2.56 (m, 2H), 2.42–2.35 (m, 2H), 2.18–2.07 (m, 2H), 1.87–1.60 (m, 2H); ^{13}C NMR (100 MHz, $CDCl_3$) δ 167.5, 135.4, 129.8, 124.9 (q, J = 283.4 Hz) , 70.1 (q, J = 30.9 Hz), 70.1, 33.48, 30.34, 13.6; ^{19}F NMR (375 MHz, $CDCl_3$) δ –79.6 (bs, 3F). IR (ATR): 3444, 2993, 2952, 1700, 1633, 1435, 1320, 1275, 1163, 1126, 1029, 712 cm^{-1}. HRMS (ESI, m/z): calcd for $C_{10}H_{14}F_3O_3^+$ $(M+H)^+$: 239.0890; Found: 239.0881.

6-Hydroxyhexyl 5,5,5-trifluoro-4-hydroxy-2-methylenepentanoate (9af)

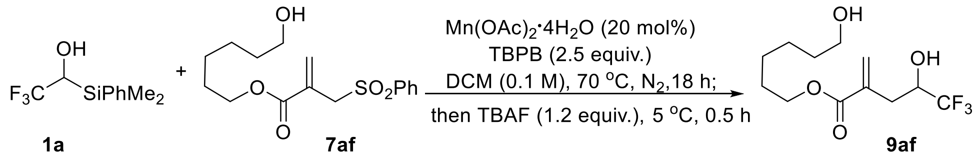

Under N_2 atmosphere, to a dried 10 mL Schlenk tube equipped with a magnetic stir bar containing $Mn(OAc)_2$•$4H_2O$ (14.7 mg, 0.06 mmol, 20 mol%), **7af** (302.4 mg, 0.9 mmol, 3.0 equiv.) was added DCM (3 mL, 0.1 M), **1a** (70.2 mg, 0.3 mmol) and TBPB (145.7 mg, 0.75 mmol, 2.5 equiv.) sequentially. The tube was sealed, and the resulting mixture was kept stirring at 70 °C in heating block for 18 h. After which the mixture was cooled to 5 °C with ice bath, TBAF (1.0 M in THF, 0.36 mL, 0.36 mmol, 1.2 equiv.) was added and the mixture was stirred at 5 °C for 0.5 h. The reaction mixture was quenched with water (2 mL), extracted with DCM (3×10 mL) and organic phase was combined and washed with brine, dried over Na_2SO_4, concentrated under reduced pressure. The crude product was purified with column chromatography on silica gel (200~300 mesh) and PE/EA (20/1 ~2/1, v/v) as eluent to afford 40.0 mg of the title compound as a colorless oil (45 % yield).

R_f = 0.45 (PE/EA = 1/1, v/v). NMR Spectroscopy: ^{1}H NMR (400 MHz, $CDCl_3$) δ 6.33 (s, 1H), 5.79 (s, 1H), 4.22–4.09 (m, 3H), 3.65 (t, J = 6.4 Hz, 2H), 2.79–2.56 (m, 2H), 2.30 (s, 2H), 1.73–1.70 (m, 2H), 1.59–1.56 (m, 2H), 1.42–1.40 (m, 4H); ^{13}C NMR (100 MHz, $CDCl_3$) δ 168.0, 135.2, 129.7, 125.0 (q, J = 280.8 Hz), 69.8 (q, J = 30.9 Hz), 65.6, 62.9, 33.5, 32.6, 28.5, 25.8, 25.4; ^{19}F NMR (375 MHz, $CDCl_3$) δ –79.5 (d, J = 6.0 Hz, 3F). IR (ATR): 3384, 2937, 2863, 1707, 1633, 1435, 1316, 1275, 1167, 1126, 1033, 708 cm^{-1}. HRMS (ESI, m/z): calcd for $C_{12}H_{19}F_3NaO_4^+$ $(M+Na)^+$: 307.1128; Found: 307.1117.

6-Oxohexyl 5,5,5-trifluoro-4-hydroxy-2-methylenepentanoate (9ag)

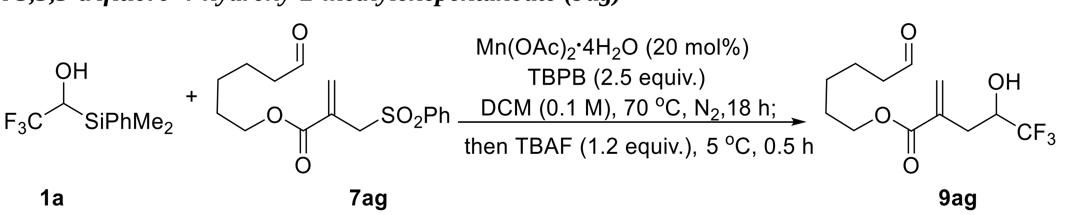

Under N_2 atmosphere, to a dried 10 mL Schlenk tube equipped with a magnetic stir bar containing $Mn(OAc)_2$•$4H_2O$

(14.7 mg, 0.06 mmol, 20 mol%), **7ag** (291.6 mg, 0.9 mmol, 3.0 equiv.) was added DCM (3 mL, 0.1 M), **1a** (70.2 mg, 0.3 mmol) and TBPB (145.7 mg, 0.75 mmol, 2.5 equiv.) sequentially. The tube was sealed, and the resulting mixture was kept stirring at 70 °C in heating block for 18 h. After which the mixture was cooled to 5 °C with ice bath, TBAF (1.0 M in THF, 0.36 mL, 0.36 mmol, 1.2 equiv.) was added and the mixture was stirred at 5 °C for 0.5 h. The reaction mixture was quenched with water (2 mL), extracted with DCM (3×10 mL) and organic phase was combined and washed with brine, dried over Na_2SO_4, concentrated under reduced pressure. The crude product was purified with column chromatography on silica gel (200~300 mesh) and PE/EA (20/1–2/1, v/v) as eluent to afford 39.0 mg of the title compound as a colorless oil (46 % yield).

R_f = 0.61 (PE/EA = 2/1, v/v). NMR Spectroscopy: ^{1}H NMR (400 MHz, $CDCl_3$) δ 9.77 (t, J = 1.5 Hz, 1H), 6.33 (s, 1H), 5.80 (s, 1H), 4.22–4.09 (m, 3H), 2.79–2.57 (m, 2H), 2.47 (td, J = 7.2, 1.3 Hz, 2H), 1.76–1.64 (m, 4H), 1.46–1.38 (m, 2H); ^{13}C NMR (100 MHz, $CDCl_3$) δ 202.5, 167.9, 135.2, 129.8, 124.9 (q, J = 281.1 Hz), 69.9 (q, J = 30.7 Hz), 65.3, 43.8, 33.5, 28.4, 25.6, 21.7; ^{19}F NMR (375 MHz, $CDCl_3$) δ –79.6 (d, J = 6.0 Hz, 3F). IR (ATR): 3425, 2930, 2859, 1711, 1275, 1167, 1126, 1029, 734 cm^{-1}. HRMS (ESI, m/z): calcd for $C_{12}H_{17}F_3NaO_4^+$ (M+Na)$^+$: 305.0971; Found: 305.0970.

1-Cyclopropyl-5,5,5-trifluoro-4-hydroxy-2-methylenepentan-1-one (9ah)

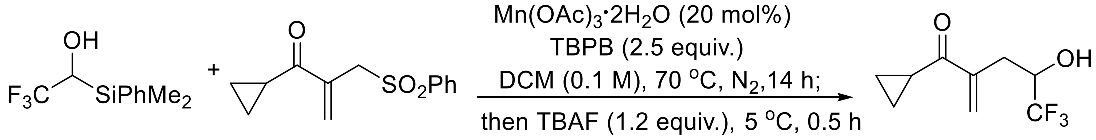

Under N_2 atmosphere, to a dried 10 mL Schlenk tube equipped with a magnetic stir bar containing $Mn(OAc)_3•2H_2O$ (16.1 mg, 0.06 mmol, 20 mol%) was added DCM (3 mL), **1a** (70.2 mg, 0.3 mmol), **7ah** (225.0 mg, 0.9 mmol, 3.0 equiv.) and TBPB (145.7 mg, 0.75 mmol, 2.5 equiv.) sequentially. The tube was then sealed, and the resulting mixture was stirred at 70 °C in a heating block for 14 h, after which the mixture was cooled to 5 °C with ice bath. TBAF (1.0 M in THF, 0.36 mL, 0.36 mmol, 1.2 equiv.) was added and the mixture was stirred at 5 °C for 0.5 h. The reaction mixture was quenched with water (2 mL) and extracted with DCM (3×10 mL). The organic phase was combined and washed with brine, dried over Na_2SO_4, concentrated under reduced pressure. The crude product was purified with column chromatography on silica gel (200~300 mesh) and PE/EA (20/1~10/1, v/v) as eluent to afford 38.7 mg of the title compound as a yellow oil (62% yield).

R_f = 0.50 (PE/EA = 5/1, v/v). ^{1}H NMR (400 MHz, $CDCl_3$) δ 6.38 (s, 1H), 6.04 (s, 1H), 4.27 (s, 1H), 4.00 (s, 1H), 2.71–2.57 (m, 2H), 2.49–2.43 (m, 1H), 1.16–1.00 (m, 4H); ^{13}C NMR (100 MHz, $CDCl_3$) δ 204.5, 144.3, 129.3, 125.0 (q, J = 280.8 Hz), 70.5 (q, J = 30.7 Hz), 33.4, 16.8, 12.7, 12.3; ^{19}F NMR (375 MHz, $CDCl_3$) δ –79.5 (d, J = 6.0 Hz, 3F). IR(ATR): 3437, 3012, 2922, 2855, 1651, 1443, 1398, 1275, 1163, 1129, 1062, 746 cm$^{–1}$. HRMS (ESI, m/z): calcd for $C_9H_{12}F_3O_2^+$ (M+H)$^+$: 209.0784; Found: 209.0784.

(1R,2S,5R)-2-Isopropyl-5-methylcyclohexyl 5,5,5-trifluoro-4-hydroxy-2-methylenepentanoate (9ai)

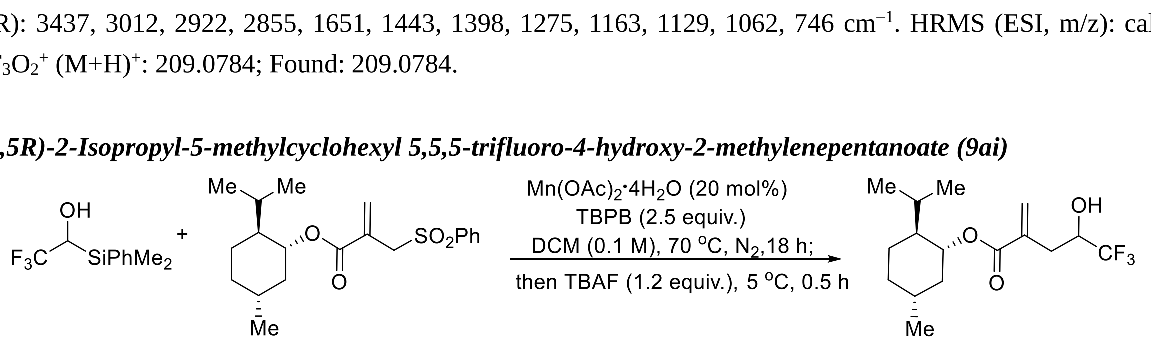

Under N_2 atmosphere, to a dried 10 mL Schlenk tube equipped with a magnetic stir bar containing $Mn(OAc)_2•4H_2O$ (14.7 mg, 0.06 mmol, 20 mol%), **7ai** (218.4 mg, 0.6 mmol, 2.0 equiv.) was added DCM (3 mL, 0.1 M), **1a** (70.2 mg, 0.3 mmol) and TBPB (145.7 mg, 0.75 mmol, 2.5 equiv.) sequentially. The tube was sealed, and the resulting mixture was kept stirring at 70 °C in heating block for 18 h. After which the mixture was cooled to 5 °C with ice bath, TBAF (1.0 M in THF, 0.36 mL, 0.36 mmol, 1.2 equiv.) was added and the mixture was stirred at 5 °C for 0.5 h. The reaction mixture was quenched with water (2 mL), extracted with DCM (3×10 mL) and organic phase was combined and washed with brine, dried over Na_2SO_4, concentrated under reduced pressure. The crude product was purified with column

chromatography on silica gel (200~300 mesh) and PE/EA (15/1~ 8/1, v/v) as eluent to afford 63.0 mg of the title compound as a colorless oil (65% yield).

R_f = 0.50 (PE/EA = 10/1, v/v). NMR Spectroscopy: ^{1}H NMR (400 MHz, $CDCl_3$) δ 6.31 (s, 1H), 5.78 (s, 1H), 4.81–4.75 (m, 1H), 4.11–4.10 (m, 1H), 3.72–3.66 (m, 1H), 2.78–2.59 (m, 2H), 2.04–2.01 (m, 1H), 1.87–1.81 (m, 1H), 1.72–1.69 (m, 2H), 1.53–1.43 (m, 2H), 1.13–0.99 (m, 2H), 0.93–0.89 (m, 7H), 0.77–0.76 (m, 3H); ^{13}C NMR (100 MHz, $CDCl_3$) δ 167.9, 135.7, 129.4, 129.4 (C'), 124.9 (q, J = 272.4 Hz), 76.0, 70.2 (q, J = 30.7 Hz), 47.2, 40.8, 34.3, 33.7, 33.6 (C'), 31.6, 26.6, 23.7, 23.6 (C'), 22.1, 20.8, 20.8 (C'), 16.6, 16.5 (C'); ^{19}F NMR (375 MHz, $CDCl_3$) δ –79.5 (d, J = 6.0 Hz, 3F), –79.5 (d, J = 6.0 Hz, 3F'). IR (ATR): 3444, 2960, 2870, 1707, 1633, 1457, 1316, 1275, 1178, 1129, 1036, 712 cm^{-1}. HRMS (ESI, m/z): calcd for $C_{16}H_{25}F_3NaO_3^+$ (M+Na)$^+$: 345.1648; Found: 345.1643.

(3S,5S,8R,9S,10S,13S,14S)-10,13-Dimethyl-17-oxohenadecahydro-1H-cyclopenta[a]pennanthren-3-yl 5,5,5-trifluoro-4-hydroxy-2-methylenepentanoate (9aj)

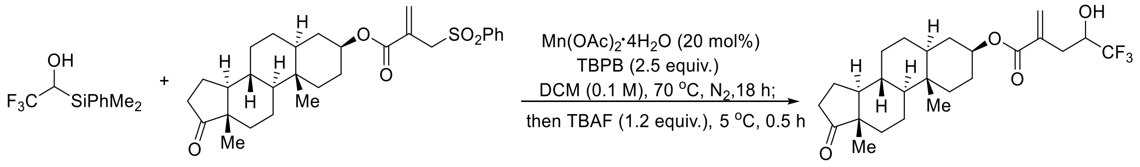

1a 7aj 9aj

Under N_2 atmosphere, to a dried 10 mL Schlenk tube equipped with a magnetic stir bar containing $Mn(OAc)_2$•$4H_2O$ (14.7 mg, 0.06 mmol, 20 mol%), **7aj** (298.8 mg, 0.6 mmol, 2.0 equiv.) was added DCM (3 mL, 0.1 M), **1a** (70.2 mg, 0.3 mmol) and TBPB (145.7 mg, 0.75 mmol, 2.5 equiv.) sequentially. The tube was sealed, and the resulting mixture was kept stirring at 70 ^{o}C in heating block for 18 h. After which the mixture was cooled to 5 ^{o}C with ice bath, TBAF (1.0 M in THF, 0.36 mL, 0.36 mmol, 1.2 equiv.) was added and the mixture was stirred at 5 ^{o}C for 0.5 h. The reaction mixture was quenched with water (2 mL), extracted with DCM (3×10 mL) and organic phase was combined and washed with brine, dried over Na_2SO_4, concentrated under reduced pressure. The crude product was purified with column chromatography on silica gel (200~300 mesh) and PE/EA (5/1, v/v) as eluent to afford 81.0 mg of the title compound as a white solid (59 % yield).

R_f = 0.24 (PE/EA = 2/1, v/v). mp: 95 ^{o}C–97 ^{o}C. NMR Spectroscopy: ^{1}H NMR (400 MHz, $CDCl_3$) δ 6.31 (s, 1H), 5.77 (s, 1H), 4.81–4.73 (m, 1H), 4.11–4.08 (m, 1H), 3.82 (s, 1H), 2.76–2.55 (m, 2H), 2.46–2.39 (m, 1H), 2.11–2.01 (m, 1H), 1.95–1.75 (m, 5H), 1.67–1.19 (m, 12H), 1.09–0.96 (m, 2H), 0.86 (s, 3H), 0.85 (s, 3H), 0.75–0.69 (m, 1H); ^{13}C NMR (100 MHz, $CDCl_3$) δ 221.7, 167.5, 135.5, 129.5, 124.9 (q, J = 280.8 Hz), 75.1, 70.0 (q, J = 30.9 Hz), 54.3, 51.4, 47.9, 44.7, 36.7, 36.0, 35.7, 35.1, 33.9, 33.5, 31.6, 30.9, 28.3, 27.4, 21.9, 20.6, 13.9, 12.3; ^{19}F NMR (375 MHz, $CDCl_3$) δ –79.5– –79.5 (m, 3F, 3F'). IR (ATR): 3370, 2933, 2855, 1718, 1633, 1405, 1312, 1291, 1178, 1122, 1014, 716 cm^{-1}. HRMS (ESI, m/z): calcd for $C_{25}H_{35}F_3NaO_4^+$ (M+Na)$^+$: 479.2380; Found: 479.2370.

(3S,8S,9S,10R,13R,14S,17R)-10,13-Dimethyl-17-((R)-6-methylheptan-2-yl)-2,3,4,7,8,9,10,11,12,13,14,15,16,17-tetradecahydro-1H-cyclopenta[a]pennanthren-3-yl 5,5,5-trifluoro-4-hydroxy-2-methylenepentanoate (9ak)

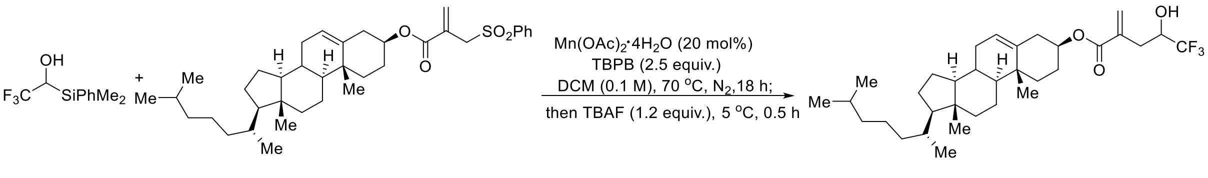

1a 7ak 9ak

Under N_2 atmosphere, to a dried 10 mL Schlenk tube equipped with a magnetic stir bar containing $Mn(OAc)_2$•$4H_2O$ (14.7 mg, 0.06 mmol, 20 mol%), **4ak** (357.6 mg, 0.6 mmol, 2.0 equiv.) was added DCM (3 mL, 0.1 M), **3a** (70.2 mg, 0.3 mmol) and TBPB (145.7 mg, 0.75 mmol, 2.5 equiv.) sequentially. The tube was sealed, and the resulting mixture was kept stirring at 70 ^{o}C in heating block for 18 h. After which the mixture was cooled to 5 ^{o}C with ice bath, TBAF (1.0 M in THF, 0.36 mL, 0.36 mmol, 1.2 equiv.) was added and the mixture was stirred at 5 ^{o}C for 0.5 h. The reaction mixture was quenched with water (2 mL), extracted with DCM (3×10 mL) and organic phase was combined and washed with

brine, dried over Na_2SO_4, concentrated under reduced pressure. The crude product was purified with column chromatography on silica gel (200~300 mesh) and PE/EA (5/1, v/v) as eluent to afford 99.5 mg of the title compound as a white solid (60 % yield).

R_f = 0.48 (PE/EA = 8/1, v/v). mp: 83 °C–85 °C. NMR Spectroscopy: ^{1}H NMR (400 MHz, $CDCl_3$) δ 6.33 (s, 1H), 5.78 (s, 1H), 5.40 (d, J = 4.3 Hz, 1H), 4.73–4.65 (m, 1H), 4.12–4.09 (m, 1H), 3.75 (s, 1H), 2.77–2.57 (m, 2H), 2.37 (d, J = 7.6 Hz, 2H), 2.03–1.95 (m, 2H), 1.92–1.79 (m, 3H), 1.70–1.43 (m, 7H), 1.40–1.25 (m, 4H), 1.20–1.08 (m, 7H), 1.03–0.95 (m, 6H), 0.91 (d, J = 6.4 Hz, 3H), 0.86 (dd, J = 6.7, 1.8 Hz, 6H), 0.68 (s, 3H); ^{13}C NMR (100 MHz, $CDCl_3$) δ 167.6, 139.4, 135.6, 129.6, 124.9 (q, J = 280.8 Hz), 123.2, 75.64, 70.1 (q, J = 30.7 Hz), 56.8, 56.2, 50.1, 42.4, 39.8, 39.6, 38.1, 37.0, 36.7, 36.3, 35.9, 33.6, 32.0, 31.9, 28.4, 28.1, 27.8, 24.4, 24.0, 23.0, 22.7, 21.2, 19.5, 18.8, 12.0; ^{19}F NMR (375 MHz, $CDCl_3$) δ –79.5 (bs, 3F). IR (ATR): 3422, 2937, 2870, 1711, 1633, 1465, 1331, 1275, 1170, 1129, 1029, 734 cm^{-1}. HRMS (ESI, m/z): calcd for $C_{33}H_{51}F_3NaO_3^+$ (M+Na)$^+$: 575.3683; Found: 575.3671.

(8R,9S,10R,13S,14S,17S)-10,13-dimethyl-3-oxo--2,3,6,7,8,9,10,11,12,13,14,15,16,17-tetradecahydro-1H-cyclo-penta[a]phenanthrene-17-yl 5,5,5-trifluoro-4-hydroxy-2-methylenepentanoate (9al)

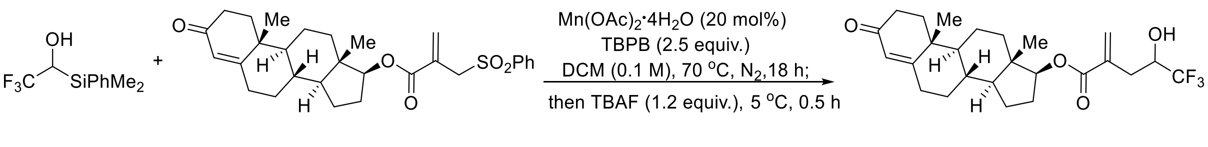

1a 7al 9al

Under N_2 atmosphere, to a dried 10 mL Schlenk tube equipped with a magnetic stir bar containing with $Mn(OAc)_2$•$4H_2O$ (14.7 mg, 0.06 mmol, 20 mol%), **7al** (297.6 mg, 0.6 mmol, 2.0 equiv.) was added DCM (3 mL, 0.1 M), **1a** (70.2 g, 0.3 mmol) and TBPB (145.7 mg, 0.75 mmol, 2.5 equiv.) sequentially. The tube was sealed, and the resulting mixture was kept stirring at 70 °C in heating block for 18 h. After which the mixture was cooled to 5 °C with ice bath, TBAF (1.0 M in THF, 0.36 mL, 0.36 mmol, 1.2 equiv.) was added and the mixture was stirred at 5 °C for 0.5 h. The reaction mixture was quenched with water (2 mL), extracted with DCM (3×10 mL) and organic phase was combined and washed with brine, dried over Na_2SO_4, concentrated under reduced pressure. The crude product was purified with column chromatography on silica gel (200~300 mesh) and PE/EA (8/1, v/v) as eluent to afford 86.0 mg of the title compound as a white solid (63% yield).

R_f = 0.43 (PE/EA = 2/1, v/v). mp: 105 °C–107 °C. NMR Spectroscopy: ^{1}H NMR (400 MHz, $CDCl_3$) δ 6.34 (s, 1H), 5.79 (s, 1H), 5.73 (s, 1H), 4.68 (t, J = 7.3 Hz, 1 H), 4.14–4.10 (m, 1H), 3.56 (s, 1H), 2.79–2.58 (m, 2H), 2.47–2.19 (m, 5H), 2.05–2.01 (m, 1H), 1.88–1.79 (m, 2H), 1.74–1.57 (m, 7H), 1.44–1.37 (m, 2H), 1.19 (s, 3H), 1.14–1.04 (m, 2H), 0.99–0.93 (m, 1H), 0.89 (s, 3H); ^{13}C NMR (100 MHz, $CDCl_3$) δ 199.7, 171.0, 167.9, 135.4, 129.7, 129.6 (C'), 125.0 (q, J = 276.5 Hz) 124.1, 83.9, 83.9 (C'), 70.1 (q, J = 32.4 Hz, C), 70.0 (q, J = 32.1 Hz, C'), 53.8, 50.3, 43.0, 38.7, 36.8, 35.8, 35.5, 34.0, 33.6, 33.5 (C'), 32.9, 31.6, 27.6, 23.7, 20.7, 17.5, 12.3; ^{19}F NMR (375 MHz, $CDCl_3$) δ –79.5 (d, J = 6.0 Hz, 3F), –79.6 (d, J = 6.0 Hz, 3F'). IR (ATR): 3355, 2937, 2855, 1711, 1659, 1435, 1376, 1275, 1170, 1126, 1036, 730 cm^{-1}. HRMS (ESI, m/z): calcd for $C_{25}H_{34}F_3O_4^+$ (M+H)$^+$: 455.2404; Found: 455.2394.

(4S,5'R,6aR,6bS,8aS,8bR,9S,10R,11aS,12aS,12bS)-5',6a,8a,9-tetramethyl-1,3,3',4,4',5,5',6,6a,6b,6',7,8,8a,8b,9,11a,12,12a,12b-icosahydrospiro[naphtho[2',1':4,5]indeno[2,1-b]furan-10,2'-pyran]-4-yl 5,5,5-trifluoro-4-hydroxy-2-methylenepentanoate (9am)

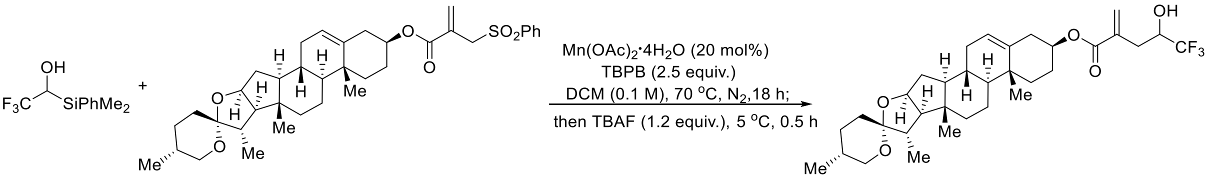

1a 7am 9am

Under N_2 atmosphere, to a dried 10 mL Schlenk tube equipped with a magnetic stir bar containing $Mn(OAc)_2$•$4H_2O$ (14.7 mg, 0.06 mmol, 20 mol%), **7am** (373.5 mg, 0.6 mmol, 2.0 equiv.) was added DCM (3 mL, 0.1 M), **1a** (70.2 mg,

0.3 mmol) and TBPB (145.7 mg, 0.75 mmol, 2.5 equiv.) sequentially. The tube was sealed, and the resulting mixture was kept stirring at 70 °C in heating block for 18 h. After which the mixture was cooled to 5 °C with ice bath, TBAF (1.0 M in THF, 0.36 mL, 0.36 mmol, 1.2 equiv.) was added and the mixture was stirred at 5 °C for 0.5 h. The reaction mixture was quenched with water (2 mL), extracted with DCM (3×10 mL) and organic phase was combined and washed with brine, dried over Na_2SO_4, concentrated under reduced pressure. The crude product was purified with column chromatography on silica gel (200~300 mesh) and PE/EA (12/1, v/v) as eluent to afford 108.0 mg of the title compound as a white solid (62 % yield).

R_f = 0.29 (PE/EA = 10/1, v/v). mp: 163 °C–165 °C (from EA and PE). NMR Spectroscopy: ^{1}H NMR (400 MHz, $CDCl_3$) δ 6.33 (s, 1H), 5.79 (s, 1H), 5.40 (d, J = 4.0 Hz, 1H), 4.73–4.65 (m, 1H), 4.41 (q, J = 7.4 Hz, 1H), 4.12–4.09(m, 1H), 3.64 (s, 1H), 3.49–3.34 (m, 2H), 2.77–2.58 (m, 2H), 2.38 (d, J = 7.6 Hz, 2H), 2.03–1.96 (m, 2H), 1.90–1.84 (m, 3H), 1.80–1.42 (m, 12H), 1.33–1.12 (m, 4H), 1.05 (s, 3H), 0.98–0.96 (m, 4H), 0.79–0.78 (m, 6H); ^{13}C NMR (100 MHz, $CDCl_3$) δ 167.5, 139.5, 135.6, 129.5, 125.0 (q, J = 280.0 Hz), 122.9, 109.4, 81.0, 75.6, 70.2 (q, J = 30.1 Hz), 67.0, 62.2, 56.6, 50.1, 41.8, 40.4, 39.9, 38.1, 37.0, 36.9, 33.6, 33.6, 32.2, 32.0, 31.6, 30.4, 29.0, 27.8, 21.0, 19.5, 17.3, 16.4, 14.7; ^{19}F NMR (375 MHz, $CDCl_3$) δ −79.5 (d, J = 6.0 Hz, 3F). IR (ATR): 3422, 2948, 2904, 1711, 1633, 1454, 1331, 1275, 1170, 1129, 1051, 734 cm^{-1}. HRMS (ESI, m/z): calcd for $C_{33}H_{48}F_3O_5^+$ (M+H)$^+$: 581.3448; Found: 581.3435.

(R)-2,5,7,8-tetramethyl-2-((4R,8R)-4,8,12-trimethyltridecyl)chroman-6-yl 5,5,5-trifluoro-4-hydroxy-2-methylenepentanoate (9an)

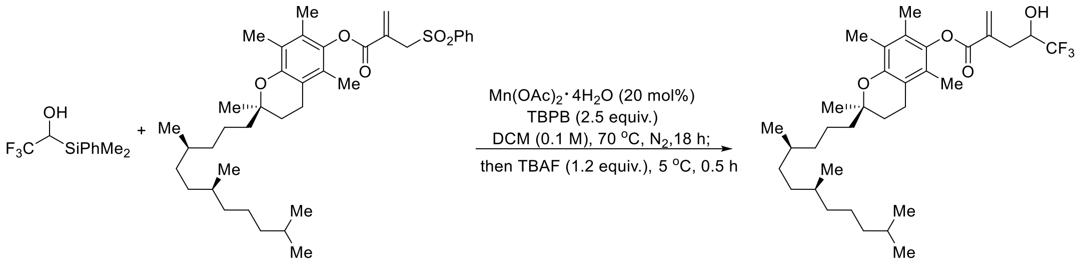

1a **7an** **9an**

Under N_2 atmosphere, to a dried 10 mL Schlenk tube equipped with a magnetic stir bar containing $Mn(OAc)_2{\bullet}4H_2O$ (14.7 mg, 0.06 mmol, 20 mol%), **7an** (297.6 mg, 0.6 mmol, 2.0 equiv.) was added DCM (3 mL, 0.1 M), **1a** (70.2 mg, 0.3 mmol) and TBPB (145.7 mg, 0.75 mmol, 2.5 equiv.) sequentially. The tube was sealed, and the resulting mixture was kept stirring at 70 °C in heating block for 18 h. After which the mixture was cooled to 5 °C with ice bath, TBAF (1.0 M in THF, 0.36 mL, 0.36 mmol, 1.2 equiv.) was added and the mixture was stirred at 5 °C for 0.5 h. The reaction mixture was quenched with water (2 mL), extracted with DCM (3×10 mL) and organic phase was combined and washed with brine, dried over Na_2SO_4, concentrated under reduced pressure. The crude product was purified with column chromatography on silica gel (200~300 mesh) and PE/EA (15/1 ~ 10/1, v/v) as eluent to afford 131.0 mg of the title compound as a colorless oil (73% yield).

R_f = 0.41 (PE/EA = 8/1, v/v). NMR Spectroscopy: ^{1}H NMR (400 MHz, $CDCl_3$) δ 6.68 (s, 1H), 6.01 (s, 1H), 4.23–4.20 (m, 1H), 2.91–2.72 (m, 3H), 2.62–2.59 (m, 2H), 2.11 (s, 3H), 2.01 (s, 3H), 1.97 (s, 3H), 1.87–1.77 (m, 2H), 1.56–1.06 (m, 23H), 0.88–0.84 (m, 13H); ^{13}C NMR (100 MHz, $CDCl_3$) δ 167.0, 149.8, 140.4, 134.8, 131.1, 126.7, 125.0, 124.9 (q, J = 280.0 Hz), 123.4, 117.7, 75.3, 70.1 (q, J = 30.8 Hz) , 39.5, 37.6, 37.4, 33.8, 32.9, 31.2 (q, J = 27.5 Hz), 28.1, 24.9, 24.6, 22.9, 22.8, 21.1, 20.7, 19.9, 19.8, 13.0, 12.2, 12.0; ^{19}F NMR (375 MHz, $CDCl_3$) δ −79.3 (bs, 3F). IR (ATR): 3265, 2930, 2866, 1730, 1636, 1461, 1342, 1275, 1167, 1133, 1051, 731 cm^{-1}. HRMS (ESI, m/z): calcd for $C_{35}H_{55}F_3NaO_4^+$ (M+Na)$^+$: 619.3945; Found: 619.3960.

(8R,9S,13S,14S)-13-methyl-17-oxo-7,8,9,11,12,13,14,15,16,17-decahydro-6H-cyclopenta[a]phenanthrene-3-yl 5,5,5-trifluoro-4-((dimethyl(phenyl)silyl)oxy)-2-methylenepentanoate (9ao)

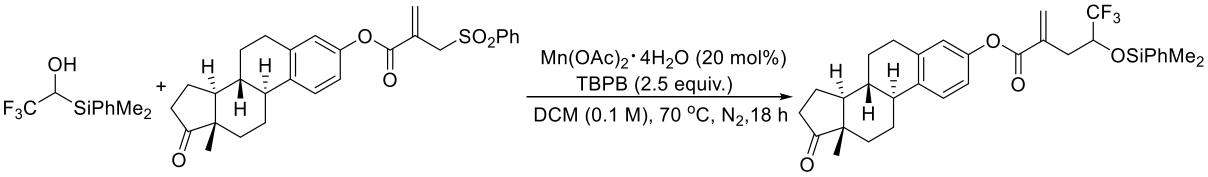

1a **7ao** **9ao**

Under N_2 atmosphere, to a dried 10 mL Schlenk tube equipped with a magnetic stir bar containing $Mn(OAc)_2$•$4H_2O$ (14.7 mg, 0.06 mmol, 20 mol%), **7ao** (286.8 mg, 0.6 mmol, 2.0 equiv.) was added DCM (3 mL, 0.1 M), **1a** (70.2 mg, 0.3 mmol) and TBPB (145.7 mg, 0.75 mmol, 2.5 equiv.) sequentially. The tube was sealed, and the resulting mixture was kept stirring at 70 °C in heating block for 18 h. The reaction mixture was quenched with water (2 mL), extracted with DCM (3×10 mL) and organic phase was combined and washed with brine, dried over Na_2SO_4, concentrated under reduced pressure. The crude product was purified with flash column chromatography on silica gel (200~300 mesh) and PE/EA (6/1, v/v) as eluent to afford 80.0 mg of the title compound as a white solid (46% yield).

R_f = 0.23 (PE/EA = 4/1, v/v). mp: 82 °C–84 °C. NMR Spectroscopy: ^{1}H NMR (400 MHz, $CDCl_3$) δ 7.56 (d, J = 6.4 Hz, 2H), 7.42–7.36 (m, 3H), 7.30 (d, J = 8.6 Hz, 1H), 6.79 (d, J = 8.6 Hz, 1H), 6.74 (s, 1H), 6.49 (s, 1H), 5.85 (s, 1H), 4.31–4.25 (m, 1H), 2.93–2.85 (m, 3H), 2.59–2.49 (m, 2H), 2.44–2.41 (m, 1H), 2.33–2.29 (m, 1H), 2.21–1.97 (m, 4H), 1.70–1.44 (m, 6H), 0.93 (s, 3H), 0.42–0.42 (m, 6H); ^{13}C NMR (100 MHz, $CDCl_3$) δ 221.0, 165.2, 148.5, 138.3, 137.7, 136.2, 134.3, 133.8, 131.8, 130.2, 128.08, 126.6, 124.9 (q, J = 275.5 Hz), 121.6, 118.7, 70.0 (q, J = 30.8 Hz), 50.5, 48.1, 44.3, 38.1, 36.0, 34.7, 31.7, 29.5, 26.6, 25.9, 21.7, 14.0, –1.2, –1.4; ^{19}F NMR (375 MHz, $CDCl_3$) δ –78.4 (m, 3F). IR (ATR): 3425, 2922, 2855, 1733, 1636, 1405, 1334, 1282, 1155, 1118, 1044, 700 cm^{-1}. HRMS (ESI, m/z): calcd for $C_{32}H_{37}F_3NaO_4Si^+$ $(M+Na)^+$: 593.2305; Found:593.2294.

(8R,9S,13S,14S,17S)-3-(benzyloxy)-13-methyl-7,8,9,11,12,13,14,15,16,17-decahydro-6H-cyclopenta[a]phenanthren-17-yl 5,5,5-trifluoro-4-hydroxy-2-methylenepentanoate (9ap)

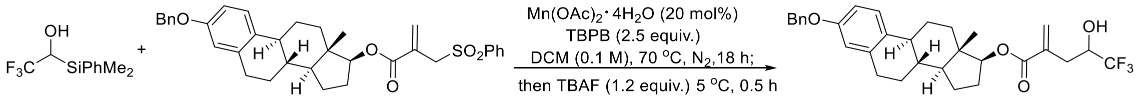

1a **7ap** **9ap**

Under N_2 atmosphere, to a dried 10 mL Schlenk tube equipped with a magnetic stir bar containing $Mn(OAc)_2$•$4H_2O$ (14.7 mg, 0.06 mmol, 20 mol%), **7ap** (342.0 mg, 0.6 mmol, 2.0 equiv.) was added DCM (3 mL, 0.1 M), **1a** (70.2 mg, 0.3 mmol) and TBPB (145.7 mg, 0.75 mmol, 2.5 equiv.) sequentially. The tube was sealed, and the resulting mixture was kept stirring at 70 °C in heating block for 18 h. After which the mixture was cooled to 5 °C with ice bath, TBAF (1.0 M in THF, 0.36 mL, 0.36 mmol, 1.2 equiv.) was added and the mixture was stirred at 5 °C for 0.5 h. The reaction mixture was quenched with water (2 mL), extracted with DCM (3×10 mL) and organic phase was combined and washed with brine, dried over Na_2SO_4, concentrated under reduced pressure. The crude product was purified with column chromatography on silica gel (200~300 mesh) and PE/EA (15/1~ 8/1, v/v) as eluent to afford 112.0 mg of the title compound as a white solid (71% yield).

R_f = 0.20 (PE/EA = 8/1 v/v). mp: 106 °C–108 °C. NMR Spectroscopy: ^{1}H NMR (400 MHz, $CDCl_3$) δ 7.46–7.37 (m, 4H), 7.35–7.31 (m, 1H), 7.21 (d, J = 8.6 Hz, 1H), 6.80 (dd, J = 8.6, 2.8 Hz, 1H), 6.74 (d, J = 2.8 Hz, 1H), 6.37 (s, 1H), 5.81 (s, 1H), 5.04 (s, 2H), 4.81–4.76 (m, 1H), 4.19–4.11 (m, 1H), 2.90–2.60 (m, 4H), 2.34–2.21 (m, 3H), 1.93–1.89 (m, 2H), 1.84–1.76 (m, 1H), 1.67–1.58 (m, 1H), 1.55–1.27 (m, 6H), 0.90 (s, 3H); ^{13}C NMR (100 MHz, $CDCl_3$) δ 168.1, 168.0 (C'), 156.9, 138.0, 137.4, 135.4, 132.7, 129.7, 129.6 (C'), 128.67, 127.98, 127.57, 126.48, 124.9 (q, J = 280.8 Hz), 114.9, 112.4, 84.2, 84.2 (C'), 70.1 (q, J = 30.9 Hz, C), 70.1 (q, J = 30.9 Hz, C'), 70.1, 69.6, 49.8, 43.9, 43.4, 38.6, 37.1, 37.1 (C'), 33.5, 27.7, 27.3, 26.3, 23.4, 12.4 12.3 (C'); ^{19}F NMR (375 MHz, $CDCl_3$) δ –79.4 (d, J = 6.0 Hz, 3F), –79.5 (d, J = 6.0 Hz, 3F'). IR (ATR): 3422, 2922, 2851, 1707, 1610, 1453, 1312, 1275, 1170, 1129, 1025, 731 cm^{-1}. HRMS (ESI, m/z): calcd for $C_{31}H_{35}F_3NaO_4^+$ $(M+Na)^+$: 551.2380; Found: 551.2389.

Synthesis of α-trifluoromethylated alkyl alcohols

3-Methyl-1-phenyl-3-(3,3,3-trifluoro-2-hydroxypropyl)indolin-2-one (11a)

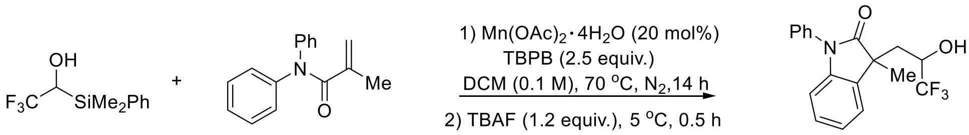

1a **10a** **11a**

Under N_2 atmosphere, to a dried 25 mL Schlenk tube equipped with a magnetic stir bar containing $Mn(OAc)_2$•$4H_2O$ (29.4 mg, 0.12 mmol, 20 mol%), **10a** (170.7 mg, 0.72 mmol, 1.2 equiv.) was added DCM (6 mL, 0.1 M), **1a** (140.4 mg, 0.6 mmol) and TBPB (291.5 mg, 1.5 mmol, 2.5 equiv.) sequentially. The tube was sealed, and the resulting mixture was kept stirring at 70 °C in heating block for 14 h. Then, the reaction was moved to 5 °C, and TBAF (188.3 mg, 0.72 mmol, 1.2 equiv.) was added, and the resulting mixture was stirred for 30 min. Then, the reaction mixture was quenched with water (8 mL), extracted with DCM (3×20 mL) and organic phase was combined and washed with saturated sodium carbonate solution and brine, dried over Na_2SO_4, concentrated under reduced pressure. The crude product was purified with column chromatography on silica gel (300–400 mesh) and PE/EA (10/1~5/1, v/v) as eluent to afford 183.0 mg of the title compound as a white solid (91% yield, the yield of two diastereomers).

More polar diastereomer: R_f = 0.32 (PE/EA = 5/1 v/v). mp: 116 °C–118 °C. NMR Spectroscopy: ^{1}H NMR (400 MHz, $CDCl_3$) δ 7.54–7.49 (m, 2H), 7.43–7.39 (m, 3H), 7.26–7.22 (m, 2H), 7.16–7.12 (m, 1H), 6.85–6.82 (m, 1H), 3.66–3.57 (m, 1H), 2.53–2.22 (m, 2H), 1.91 (s, 1H), 1.54 (s, 3H); ^{13}C NMR (150 MHz, $CDCl_3$) δ 180.4, 143.8, 134.6, 131.6, 129.8, 128.6, 128.3, 126.8, 124.8 (q, J = 280.3 Hz), 123.4, 122.9, 110.0, 68.7 (q, J = 31.2 Hz), 46.1, 37.8, 25.8; ^{19}F NMR NMR (375 MHz, $CDCl_3$) δ –80.0 (d, J = 7.2 Hz, 3F). IR (ATR): 3377, 3056, 2967, 2926, 1703, 1610, 1506, 1379, 1282, 1163, 1126, 1028, 854, 760 cm^{-1}. HRMS (ESI, m/z): calcd for $C_{18}H_{16}F_3NO_2Na^+$ $(M+Na)^+$: 358.1025; Found: 358.1012.

1,3-Dimethyl-3-(3,3,3-trifluoro-2-hydroxypropyl)indolin-2-one (11b)

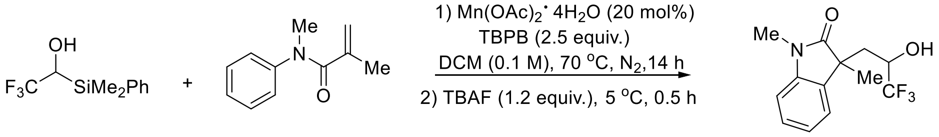

1a **10b** **11b**

Under N_2 atmosphere, to a dried 25 mL Schlenk tube equipped with a magnetic stir bar containing $Mn(OAc)_2$•$4H_2O$ (29.4 mg, 0.12 mmol, 20 mol%), **10b** (126.1 mg, 0.72 mmol, 1.2 equiv.) was added DCM (6 mL, 0.1 M), **1a** (140.4 mg, 0.6 mmol) and TBPB (291.5 mg, 1.5 mmol, 2.5 equiv.) sequentially. The tube was sealed, and the resulting mixture was kept stirring at 70 °C in heating block for 14 h. Then, the reaction was moved to 5 °C, and TBAF (188.3 mg, 0.72 mmol, 1.2 equiv.) was added, and the resulting mixture was stirred for 30 min. Then, the reaction mixture was quenched with water (8 mL), extracted with DCM (3×20 mL) and organic phase was combined and washed with saturated sodium carbonate solution and brine, dried over Na_2SO_4, concentrated under reduced pressure. The crude product was purified with column chromatography on silica gel (300–400 mesh) and PE/EA (5/1~2/1, v/v) as eluent to afford 124.6 mg of the title compound as a light yellow solid (76% yield, the yield of two diastereomers).

More polar diastereomer: R_f = 0.34 (PE/EA = 2/1 v/v). mp: 99 °C–100 °C. NMR Spectroscopy: ^{1}H NMR (400 MHz, $CDCl_3$) δ 7.32 (td, J = 7.7, 1.4 Hz, 1H), 7.19-7.16 (m, 1H), 7.10 (td, J = 7.5, 1.0 Hz, 1H), 6.89 (d, J = 7.8 Hz, 1H), 3.58–3.49 (m, 1H), 3.21 (s, 3H), 2.43–2.13 (m, 3H), 1.42 (s, 3H); ^{13}C NMR (150 MHz, $CDCl_3$) δ 180.9, 143.6, 132.1, 128.7, 124.8 (q, J = 280.2 Hz), 123.0, 122.6, 108.8, 68.4 (q, J = 31.3 Hz), 46.0, 37.4, 26.6, 25.4.^{19}F NMR (375 MHz, $CDCl_3$) δ -80.2 (d, J = 7.5 Hz, 3F). IR (ATR): 3377, 3056, 2967, 2930, 1696, 1614, 1495, 1379, 1353, 1308, 1279, 1167, 1122, 1028, 954, 757 cm^{-1}. HRMS (ESI, m/z): calcd for $C_{13}H_{15}F_3NO_2^+$ $(M+H)^+$: 274.1049; Found: 274.1041.

1-Benzyl-3-methyl-3-(3,3,3-trifluoro-2-hydroxypropyl)indolin-2-one (11c)

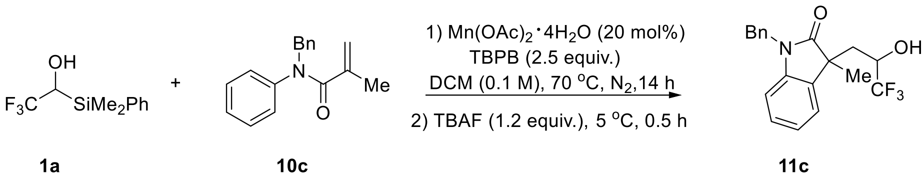

Under N_2 atmosphere, to a dried 25 mL Schlenk tube equipped with a magnetic stir bar containing $Mn(OAc)_2•4H_2O$ (29.4 mg, 0.12 mmol, 20 mol%) was added DCM (6 mL, 0.1 M), **1a** (140.4 mg, 0.6 mmol), **10c** (180.7 mg, 0.72 mmol, 1.2 equiv.) and TBPB (291.5 mg, 1.5 mmol, 2.5 equiv.) sequentially. The tube was sealed, and the resulting mixture was kept stirring at 70 °C in heating block for 14 h. Then, the reaction was moved to 5 °C, and TBAF (188.3 mg, 0.72 mmol, 1.2 equiv.) was added, and the resulting mixture was stirred for 30 min. Then, the reaction mixture was quenched with water (8 mL), extracted with DCM (3×20 mL) and organic phase was combined and washed with saturated sodium carbonate solution and brine, dried over Na_2SO_4, concentrated under reduced pressure. The crude product was purified with column chromatography on silica gel (300–400 mesh) and PE/EA (10/1~5/1, v/v) as eluent to afford 164.9 mg of the title compound as a light yellow solid (79% yield, The yield of two diastereomers).

More polar diastereomer: R_f = 0.36 (PE/EA = 5/1 v/v). mp: 110 °C–112 °C. NMR Spectroscopy: ^{1}H NMR (400 MHz, $CDCl_3$) δ 7.34–7.30 (m, 4H), 7.30–7.23 (m, 1H), 7.21–7.17 (m, 2H), 7.08–7.04 (m, 1H), 6.77–6.74 (m, 1H), 4.91 (d, J = 1.3 Hz, 2H), 3.66–3.56 (m, 1H), 2.50–2.18 (m, 3H), 1.47 (s, 3H); ^{13}C NMR (150 MHz, $CDCl_3$) δ 180.9, 142.7, 135.9, 132.1, 128.9, 128.5, 127.8, 127.5, 124.8 (q, J = 280.4 Hz), 123.0, 122.7, 109.8, 68.4 (q, J = 31.0 Hz), 46.1, 44.1, 37.1, 26.0; ^{19}F NMR (375 MHz, $CDCl_3$) δ –80.0 (d, J = 7.4 Hz, 3F). IR (ATR): 3384, 3064, 2930, 1692, 1610, 1491, 1383, 1308, 1275, 1163, 1126, 1029, 999, 954, 790 cm^{-1}. HRMS (ESI, m/z): calcd for $C_{19}H_{19}F_3NO_2^+$ $(M+H)^+$: 350.1362; Found: 350.1357.

7-Fluoro-1,3-dimethyl-3-(3,3,3-trifluoro-2-hydroxypropyl)indolin-2-one (11d)

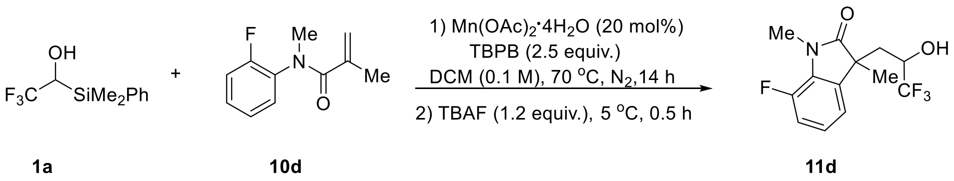

Under N_2 atmosphere, to a dried 25 mL Schlenk tube equipped with a magnetic stir bar containing $Mn(OAc)_2•4H_2O$ (29.4 mg, 0.12 mmol, 20 mol%) was added DCM (6 mL, 0.1 M), **1a** (140.4 mg, 0.6 mmol), **10d** (138.9 mg, 0.72 mmol, 1.2 equiv.) and TBPB (291.5 mg, 1.5 mmol, 2.5 equiv.) sequentially. The tube was sealed, and the resulting mixture was kept stirring at 70 °C in heating block for 14 h. Then, the reaction was moved to 5 °C, and TBAF (188.3 mg, 0.72 mmol, 1.2 equiv.) was added, and the resulting mixture was stirred for 30 min. Then, the reaction mixture was quenched with water (8 mL), extracted with DCM (3×20 mL) and organic phase was combined and washed with saturated sodium carbonate solution and brine, dried over Na_2SO_4, concentrated under reduced pressure. The crude product was purified with column chromatography on silica gel (300–400 mesh) and PE/EA (10/1~5/1, v/v) as eluent to afford 139.1 mg of the title compound as a white solid (80% yield, The yield of two diastereomers).

More polar diastereomer: R_f = 0.26 (PE/EA = 5/1 v/v). mp: 111 °C–113 °C. NMR Spectroscopy: ^{1}H NMR (400 MHz, $CDCl_3$) δ 7.06–7.00 (m, 2H), 6.98–6.93 (m, 1H), 3.60–3.47 (m, 1H), 3.41 (d, J = 2.6 Hz, 3H), 2.58 (d, J = 8.3 Hz, 1H), 2.43–2.10 (m, 2H), 1.41 (s, 3H); ^{13}C NMR (150 MHz, $CDCl_3$) δ 180.5, 148.2 (d, J = 243.0 Hz), 135.1 (d, J = 3.2 Hz), 130.3 (d, J = 8.1 Hz), 124.7 (q, J = 280.2 Hz), 123.6 (d, J = 6.4 Hz), 118.4 (d, J = 3.1 Hz), 116.7 (d, J = 19.1 Hz), 68.3 (q, J = 31.3 Hz), 46.3 (d, J = 1.9 Hz), 37.5, 29.0 (d, J = 5.9 Hz), 25.5; ^{19}F NMR (375 MHz, $CDCl_3$) δ –80.1 (d, J = 7.3 Hz, 3F), –135.5– –135.6 (m, 1F). IR (ATR): 3384, 2974, 2930, 1700, 1633, 1599, 1484, 1375, 1279, 1241, 1167, 1118, 1029, 910, 783, 738 cm^{-1}. HRMS (ESI, m/z): calcd for $C_{13}H_{13}F_4NO_2Na^+$ $(M+Na)^+$: 314.0775; Found: 314.0764.

5-Fluoro-1,3-dimethyl-3-(3,3,3-trifluoro-2-hydroxypropyl)indolin-2-one (11e)

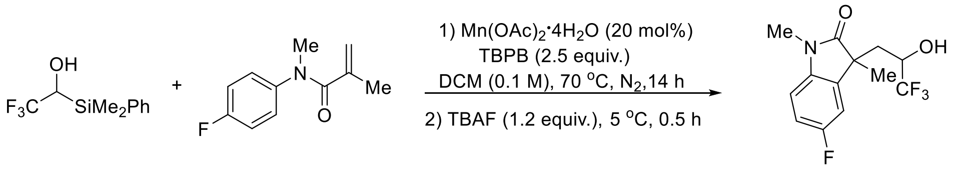

Under N_2 atmosphere, to a dried 25 mL Schlenk tube equipped with a magnetic stir bar containing $Mn(OAc)_2$•$4H_2O$ (29.4 mg, 0.12 mmol, 20 mol%) was added DCM (6 mL, 0.1 M), **1a** (140.4 mg, 0.6 mmol), **10e** (138.9 mg, 0.72 mmol, 1.2 equiv.) and TBPB (291.5 mg, 1.5 mmol, 2.5 equiv.) sequentially. The tube was sealed, and the resulting mixture was kept stirring at 70 °C in heating block for 14 h. Then, the reaction was moved to 5 °C, and TBAF (188.3 mg, 0.72 mmol, 1.2 equiv.) was added, and the resulting mixture was stirred for 30 min. Then, the reaction mixture was quenched with water (8 mL), extracted with DCM (3×20 mL) and organic phase was combined and washed with saturated sodium carbonate solution and brine, dried over Na_2SO_4, concentrated under reduced pressure. The crude product was purified with column chromatography on silica gel (300–400 mesh) and PE/EA (10/1~2/1, v/v) as eluent to afford 147.4 mg of the title compound as a light yellow solid (84% yield, the yield of two diastereomers).

More polar diastereomer: R_f = 0.21 (PE/EA = 2/1 v/v). mp: 164 °C–166 °C. NMR Spectroscopy: ^{1}H NMR (400 MHz, $CDCl_3$) δ 7.05–7.00 (m, 1H), 6.94 (dd, *J* = 7.7, 2.6 Hz, 1H), 6.81 (dd, *J* = 8.5, 4.1 Hz, 1H), 3.65-3.50 (m, 1H), 3.20 (s, 3H), 2.47 (d, *J* = 8.4 Hz, 1H), 2.43–2.10 (m, 2H), 1.42 (s, 3H); ^{13}C NMR (150 MHz, $CDCl_3$) *δ* 180.5, 159.6 (d, *J* = 240.2 Hz), 139.5 (d, *J* = 2.0 Hz), 133. 8 (d, *J* = 7.7 Hz), 124.7 (q, *J* = 280.4 Hz), 114.9 (d, *J* = 23.3 Hz), 110.9 (d, *J* = 24.4 Hz), 109.3 (d, *J* = 8.0 Hz), 68.3 (q, *J* = 31.2 Hz), 46.5 (d, *J* = 1.8 Hz), 37.3, 26.7, 25.2; ^{19}F NMR (375 MHz, $CDCl_3$) *δ* -80.1 (d, *J* = 8.2 Hz, 3F), –119.8– –119.9 (m, 1F). IR (ATR): 3396, 2922, 2855, 1692, 1621, 1498, 1375, 1312, 1275, 1234, 1170, 1126, 1029, 898, 812, 701 cm^{-1}. HRMS (ESI, m/z): calcd for $C_{13}H_{13}F_4NO_2Na^+$ $(M+Na)^+$: 314.0775; Found: 314.0765.

5-Iodo-1,3-dimethyl-3-(3,3,3-trifluoro-2-hydroxypropyl)indolin-2-one (11f)

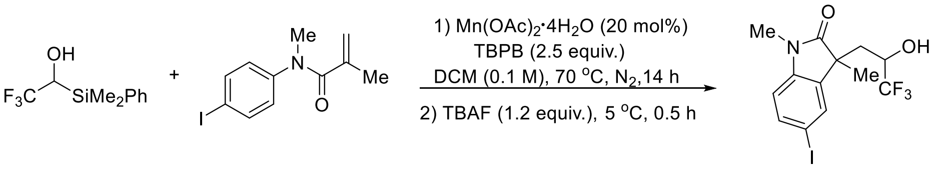

Under N_2 atmosphere, to a dried 25 mL Schlenk tube equipped with a magnetic stir bar containing $Mn(OAc)_2$•$4H_2O$ (29.4 mg, 0.12 mmol, 20 mol%), **10f** (216.7 mg, 0.72 mmol, 1.2 equiv.) was added DCM (6 mL, 0.1 M), **1a** (140.4 mg, 0.6 mmol) and TBPB (291.5 mg, 1.5 mmol, 2.5 equiv.) sequentially. The tube was sealed, and the resulting mixture was kept stirring at 70 °C in heating block for 14 h. Then, the reaction was moved to 5 °C, and TBAF (188.3 mg, 0.72 mmol, 1.2 equiv.) was added, and the resulting mixture was stirred for 30 min. Then, the reaction mixture was quenched with water (8 mL), extracted with DCM (3×20 mL) and organic phase was combined and washed with saturated sodium carbonate solution and brine, dried over Na_2SO_4, concentrated under reduced pressure. The crude product was purified with column chromatography on silica gel (300–400 mesh) and PE/EA (10/1~2/1, v/v) as eluent to afford 172.6 mg of the title compound as a white solid (72% yield, the yield of two diastereomers).

More polar diastereomer: R_f = 0.26 (PE/EA = 2/1 v/v). mp: 176 °C–178 °C. NMR Spectroscopy: ^{1}H NMR (400 MHz, $CDCl_3$) δ 7.64 (dd, *J* = 8.1, 1.7 Hz, 1H), 7.46 (d, *J* = 1.7 Hz, 1H), 6.67 (d, *J* = 8.2 Hz, 1H), 3.65–3.54 (m, 1H), 3.18 (s, 3H), 2.42–2.10 (m, 3H), 1.41 (s, 3H); ^{13}C NMR (150 MHz, $CDCl_3$) *δ* 180.1, 143.3, 137.6, 134.6, 131.5, 124.7 (q, *J* = 280.1 Hz), 110.8, 85.4, 68.3 (q, *J* = 31.2 Hz), 46.0, 37.1, 26.6, 25.3; ^{19}F NMR (375 MHz, $CDCl_3$) *δ* –80.0 (d, *J* = 7.3 Hz, 3F). IR (ATR): 3332, 2967, 2926, 1700, 1603, 1491, 1454, 1416, 1349, 1275, 1174, 1126, 1021, 880, 809, 734 cm^{-1}. HRMS (ESI, m/z): calcd for $C_{13}H_{13}F_3INO_2Na^+$ $(M+Na)^+$: 421.9835; Found: 421.9829.

5-Methoxy-1,3-dimethyl-3-(3,3,3-trifluoro-2-hydroxypropyl)indolin-2-one (11g)

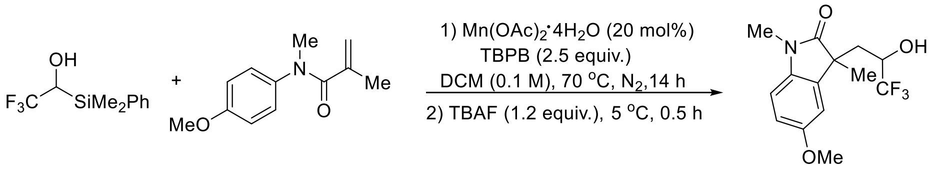

1a **10g** **11g**

Under N_2 atmosphere, to a dried 25 mL Schlenk tube equipped with a magnetic stir bar containing $Mn(OAc)_2$•$4H_2O$ (29.4 mg, 0.12 mmol, 20 mol%) was added DCM (6 mL, 0.1 M), **1a** (140.4 mg, 0.6 mmol), **10g** (147.7 mg, 0.72 mmol, 1.2 equiv.) and TBPB (291.5 mg, 1.5 mmol, 2.5 equiv.) sequentially. The tube was sealed, and the resulting mixture was kept stirring at 70 ºC in heating block for 14 h. Then, the reaction was moved to 5 °C, and TBAF (188.3 mg, 0.72 mmol, 1.2 equiv.) was added, and the resulting mixture was stirred for 30 min. Then, the reaction mixture was quenched with water (8 mL), extracted with DCM (3×20 mL) and organic phase was combined and washed with saturated sodium carbonate solution and brine, dried over Na_2SO_4, concentrated under reduced pressure. The crude product was purified with column chromatography on silica gel (300–400 mesh) and PE/EA (10/1~2/1, v/v) as eluent to afford 129.4 mg of the title compound as a white solid (71% yield, the yield of two diastereomers).

More polar diastereomer: R_f = 0.17 (PE/EA = 2/1 v/v). mp: 145 ºC–147 ºC. NMR Spectroscopy: ^{1}H NMR (400 MHz, $CDCl_3$) δ 6.84–6.82 (m, 1H), 6.80–6.78 (m, 2H), 3.81 (s, 3H), 3.62–3.53 (m, 1H), 3.19 (s, 3H), 2.57 (d, J = 8.4 Hz, 1H), 2.41–2.10 (m, 2H), 1.41 (s, 3H); ^{13}C NMR (150 MHz, $CDCl_3$) δ 180.6, 156.4, 137.0, 133.6, 124.8 (q, J = 280.3 Hz), 112.4, 110.4, 109.1, 68.4 (q, J = 31.2 Hz), 56.0, 46.5, 37.4, 26.7, 25.4; ^{19}F NMR (375 MHz, $CDCl_3$) δ –80.1(d, J = 6.4 Hz, 3F). IR (ATR): 3377, 2930, 1692, 1603, 1498, 1435, 1372, 1282, 1167, 1126, 1040, 805, 698 cm^{-1}. HRMS (ESI, m/z): calcd for $C_{14}H_{17}F_3NO_3^+$ $(M+H)^+$: 304.1155; Found: 304.1143.

1-Methyl-1-(3,3,3-trifluoro-2-hydroxypropyl)-5,6-dihydro-1H-pyrrolo[3,2,1-ij]quinolin-2(4H)-one (11h)

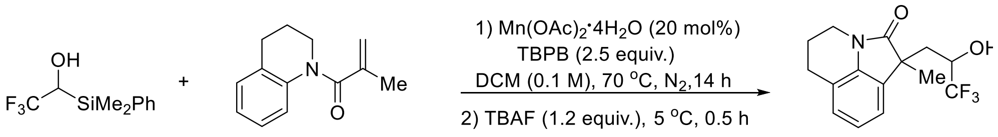

1a **10h** **11h**

Under N_2 atmosphere, to a dried 25 mL Schlenk tube equipped with a magnetic stir bar containing $Mn(OAc)_2$•$4H_2O$ (29.4 mg, 0.12 mmol, 20 mol%), **10h** (144.8 mg, 0.72 mmol, 1.2 equiv.) was added DCM (6 mL, 0.1 M), **1a** (140.4 mg, 0.6 mmol) and TBPB (291.5 mg, 1.5 mmol, 2.5 equiv.) sequentially. The tube was sealed, and the resulting mixture was kept stirring at 70 ºC in heating block for 14 h. Then, the reaction was moved to 5 °C, and TBAF (188.3 mg, 0.72 mmol, 1.2 equiv.) was added, and the resulting mixture was stirred for 30 min. Then, the reaction mixture was quenched with water (8 mL), extracted with DCM (3×20 mL) and organic phase was combined and washed with saturated sodium carbonate solution and brine, dried over Na_2SO_4, concentrated under reduced pressure. The crude product was purified with column chromatography on silica gel (300–400 mesh) and PE/EA (10/1~1.5/1, v/v) as eluent to afford 138.1 mg of the title compound as a light yellow solid (77% yield, the yield of two diastereomers).

More polar diastereomer: R_f = 0.48 (PE/EA = 1.5/1 v/v). mp: 69 ºC–72 ºC. NMR Spectroscopy: ^{1}H NMR (400 MHz, $CDCl_3$) δ 7.07–6.96 (m, 3H), 3.76-3.64 (m, 2H), 3.61–3.53 (m, 1H), 2.81–2.78 (m, 2H), 2.41-2.12 (m, 2H), 2.05–1.98 (m, 2H), 1.42 (s, 3H); ^{13}C NMR (150 MHz, $CDCl_3$) δ 179.8, 139.3, 130.6, 127.4, 124.9 (q, J = 280.3 Hz), 122.4, 120.9, 120.4, 68.4 (q, J = 31.0 Hz), 47.4, 39.1, 37.3, 25.1, 24.7, 21.2; ^{19}F NMR (375 MHz, $CDCl_3$) δ -80.1 (d, J = 6.2 Hz, 3F). IR (ATR): 3377, 2963, 2930, 2874, 1692, 1638, 1484, 1394, 1361, 1279, 1163, 1126, 958, 783, 697 cm^{-1}. HRMS (ESI, m/z): calcd for $C_{15}H_{17}F_3NO_2^+$ $(M+H)^+$: 300.1206; Found: 300.1201.

Methyl 1,3-dimethyl-2-oxo-3-(3,3,3-trifluoro-2-hydroxypropyl)indoline-5-carboxylate (11i)

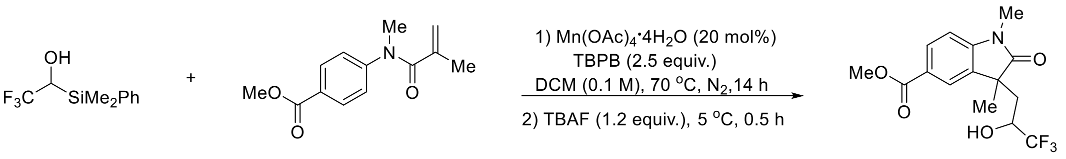

1a **10i** **11i**

Under N_2 atmosphere, to a dried 25 mL Schlenk tube equipped with a magnetic stir bar containing $Mn(OAc)_2$•$4H_2O$ (29.4 mg, 0.12 mmol, 20 mol%), **10i** (167.8 mg, 0.72 mmol, 1.2 equiv.) was added DCM (6 mL, 0.1 M), **1a** (140.4 mg, 0.6 mmol) and TBPB (291.5 mg, 1.5 mmol, 2.5 equiv.) sequentially. The tube was sealed, and the resulting mixture was kept stirring at 70 °C in heating block for 14 h. Then, the reaction was moved to 5 °C, and TBAF (188.3 mg, 0.72 mmol, 1.2 equiv.) was added, and the resulting mixture was stirred for 30 min. Then, the reaction mixture was quenched with water (8 mL), extracted with DCM (3×20 mL) and organic phase was combined and washed with saturated sodium carbonate solution and brine, dried over Na_2SO_4, concentrated under reduced pressure. The crude product was purified with column chromatography on silica gel (300 400 mesh) and PE/EA (10/1~1.5/1, v/v) as eluent to afford 125.1 mg of the title compound as a light yellow solid (63% yield, the yield of two diastereomers).

More polar diastereomer: R_f = 0.39 (PE/EA = 1.5/1, v/v). mp: 118 °C–120 °C. NMR Spectroscopy: ^{1}H NMR (400 MHz, $CDCl_3$) δ 8.05 (dd, J = 8.2, 1.7 Hz, 1H), 7.85 (dd, J = 1.7, 0.5 Hz, 1H), 6.91 (d, J = 7.8 Hz, 1H), 3.91 (s, 3H), 3.59–3.49 (m, 1H), 3.23 (s, 3H), 2.67 (d, J = 8.3 Hz, 1H), 2.45–2.16 (m, 2H), 1.43 (s, 3H); ^{13}C NMR (150 MHz, $CDCl_3$) δ 181.2, 166.9, 147.8, 132.1, 131.4, 124.8, 124.7 (q, J = 280.4 Hz), 124.0, 108.2, 68.3 (q, J = 31.2 Hz), 52.3, 45.8, 37.2, 26.8, 25.1; ^{19}F NMR (375 MHz, $CDCl_3$) δ –79.9 (d, J = 7.5 Hz, 3F). IR (ATR): 3422, 2956, 1707, 1618, 1498, 1457, 1375, 1286, 1256, 1167, 1126, 1025, 917, 835, 775, 738 cm^{-1}. HRMS (ESI, m/z): calcd for $C_{15}H_{17}F_3NO_4^+$ $(M+H)^+$: 332.1104; Found: 332.1099.

Synthesis of α-trifluoromethylated allylic alcohols

(E)-1,1,1-trifluoro-4-(2-fluorophenyl)but-3-en-2-ol (13a)

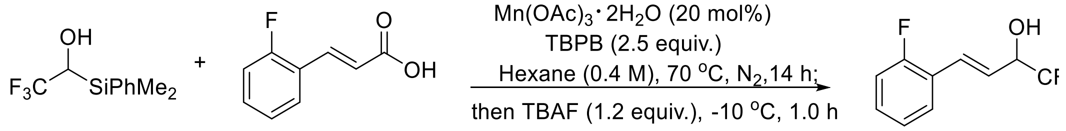

1a **12a** **13a**

Under N_2 atmosphere, to a dried 10 mL Schlenk tube equipped with a magnetic stir bar containing $Mn(OAc)_3$•$2H_2O$ (21.4 mg, 0.08 mmol, 20 mol%) and **12a** (132.8 mg, 0.8 mmol, 2.0 equiv.) was added hexane (1 mL, 0.4 M), TBPB (194.3 mg, 1.0 mmol, 2.5 equiv.) and **1a** (93.6 mg, 0.4 mmol) sequentially. The tube was sealed, and the resulting mixture was kept stirring at 70 °C in heating block for 14 h. The mixture was then cooled to −10 °C with low temperature bath, TBAF (1.0 M in THF, 0.48 mL, 0.48 mmol, 1.2 equiv.) was added and the resulting mixture was stirred at −10 °C for 1.0 h. The reaction mixture was quenched with water (2 mL), extracted with DCM (3×10 mL) and organic phase was combined and washed with brine, dried over Na_2SO_4, concentrated under reduced pressure. The crude product was purified with column chromatography on silica gel (200~300 mesh) and PE/EA (20/1~10/1, v/v) as eluent to afford 63.8 mg of the title compound as a colorless oil (73% yield).

R_f = 0.56 (PE/EA = 4/1, v/v). NMR Spectroscopy: ^{1}H NMR (400 MHz, $CDCl_3$) δ 7.47 (t, J = 7.2 Hz, 1H), 7.31–7.26 (m, 1H), 7.15–7.00 (m, 3n), 6.31 (dd, J = 16.2, 6.4 Hz, 1H), 4.69–4.63 (m, 1H), 2.44 (s, 1H); ^{13}C NMR (100 MHz, $CDCl_3$) δ 160.7 (d, J = 250.5 Hz), 130.3 (d, J = 8.7 Hz), 129.0 (d, J = 2.9 Hz), 128.1 (d, J = 2.9 Hz), 124.4 (q, J = 273.4 Hz), 124.4 (d, J = 3.9 Hz), 123.4, 123.4 (d, J = 6.7 Hz), 116.1 (d, J = 22.2 Hz), 71.9 (q, J = 32.1 Hz); ^{19}F NMR (375 MHz, $CDCl_3$) δ –78.9 (d, J = 6.0 Hz, 3F), –117.0– –117.0 (m, 1F). IR (ATR): 3396, 2922, 1659, 1491, 1457, 1267, 1174, 1125, 969, 883, 753 cm^{-1}. HRMS (ESI, m/z): calcd for $C_{10}H_6F_4O^-$ $(M–H)^-$: 219.0439; Found:219.0441.

(E)-1,1,1-Trifluoro-4-(3-fluorophenyl)but-3-en-2-ol (13b)

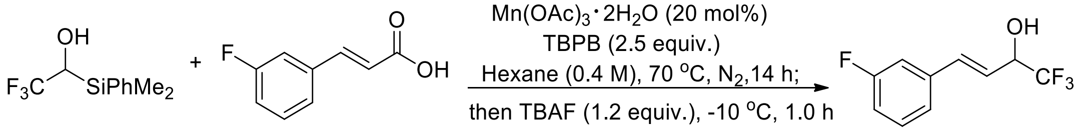

1a **12b** **13a**

Under N_2 atmosphere, to a dried 10 mL Schlenk tube equipped with a magnetic stir bar containing $Mn(OAc)_3$•$2H_2O$ (21.4 mg, 0.08 mmol, 20 mol%) and **12b** (132.8 mg, 0.8 mmol, 2.0 equiv.) was added hexane (1 mL, 0.4 M), TBPB (194.3 mg, 1.0 mmol, 2.5 equiv.) and **1a** (93.6 mg, 0.4 mmol) sequentially. The tube was sealed, and the resulting mixture was kept stirring at 70 ºC in heating block for 14 h. The mixture was then cooled to –10 ºC with low temperature bath, TBAF (1.0 M in THF, 0.48 mL, 0.48 mmol, 1.2 equiv.) was added and the resulting mixture was stirred at –10 ºC for 1.0 h. The reaction mixture was quenched with water (2 mL), extracted with DCM (3×10 mL) and organic phase was combined and washed with brine, dried over Na_2SO_4, concentrated under reduced pressure. The crude product was purified with column chromatography on silica gel (200~300 mesh) and PE/EA (20/1~10/1, v/v) as eluent to afford 52.9 mg of the title compound as a colorless oil (60% yield).

R_f = 0.64 (PE/EA = 4/1, v/v). NMR Spectroscopy: ^{1}H NMR (400 MHz, $CDCl_3$) δ 7.33–7.28 (m, 1H), 7.18 (d, J = 7.6 Hz, 1H), 7.11 (d, J = 9.8 Hz, 1H), 6.99 (t, J = 8.3 Hz, 1H), 6.83 (d, J = 15.9 Hz, 1H), 6.20 (dd, J = 15.9, 6.1 Hz, 1H), 4.66–4.63 (m, 1H), 2.39 (s, 1H); ^{13}C NMR (150 MHz, $CDCl_3$) δ 163.20 (d, J = 246.3 Hz), 137.8 (d, J = 8.0 Hz), 135.1 (d, J = 2.6 Hz), 130.4 (d, J = 8.5 Hz), 123.0 (d, J = 2.8 Hz), 124.3 (q, J = 280.4 Hz), 122.2, 115.7 (d, J = 21.5 Hz), 113.5 (d, J = 21.9 Hz), 71.5 (q, J = 32.5 Hz); ^{19}F NMR (375 MHz, $CDCl_3$) δ –79.0 (d, J = 6.0 Hz, 3F), –112.9– –113.0 (m, 1F). IR (ATR): 3418, 2904, 1588, 1491, 1267, 1178, 1129, 969, 783 cm^{-1}. HRMS (ESI, m/z): calcd for $C_{10}H_6F_4O^-$ (M–H)$^-$: 219.0439; Found: 219.0441.

(E)-1,1,1-Trifluoro-4-(4-fluorophenyl)but-3-en-2-ol (13c)

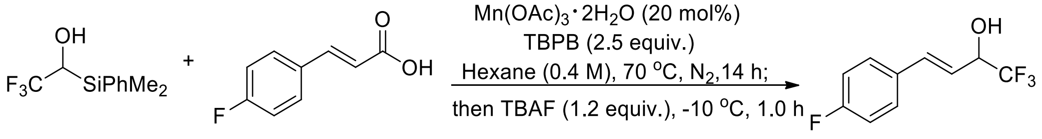

1a **12c** **13c**

Under N_2 atmosphere, to a dried 10 mL Schlenk tube equipped with a magnetic stir bar containing $Mn(OAc)_3$•$2H_2O$ (21.4 mg, 0.08 mmol, 20 mol%) and **12c** (132.8 mg, 0.8 mmol, 2.0 equiv.) was added hexane (1 mL, 0.4 M), TBPB (194.3 mg, 1.0 mmol, 2.5 equiv.) and **1a** (93.6 mg, 0.4 mmol) sequentially. The tube was sealed, and the resulting mixture was kept stirring at 70 ºC in heating block for 14 h. The mixture was then cooled to –10 ºC with low temperature bath, TBAF (1.0 M in THF, 0.48 mL, 0.48 mmol, 1.2 equiv.) was added and the resulting mixture was stirred at –10 ºC for 1.0 h. The reaction mixture was quenched with water (2 mL), extracted with DCM (3×10 mL) and organic phase was combined and washed with brine, dried over Na_2SO_4, concentrated under reduced pressure. The crude product was purified with column chromatography on silica gel (200~300 mesh) and PE/EA (20/1~10/1, v/v) as eluent to afford 56.3 mg of the title compound as a colorless oil (64% yield).

R_f = 0.56 (PE/EA = 4/1, v/v). NMR Spectroscopy: ^{1}H NMR (400 MHz, $CDCl_3$) δ 7.38 (dd, J = 7.6, 5.8 Hz, 2H), 7.04 (t, J = 8.7 Hz, 2H), 6.81 (d, J = 15.9 Hz, 1H), 6.12 (dd, J = 15.9, 6.6 Hz, 1H), 4.66–4.59 (m, 1H), 2.78 (bs, 1H); ^{13}C NMR (100 MHz, $CDCl_3$) δ 163.1 (d, J = 248.5 Hz), 135.2, 131.7, 128.7 (d, J = 8.7 Hz), 124.4 (d, J = 280.8 Hz), 120.5, 115.9 (d, J = 21.2 Hz), 71.7 (q, J = 32.4 Hz); ^{19}F NMR (375 MHz, $CDCl_3$) δ –78.9 (d, J = 6.0 Hz, 3F), –112.5– –112.5 (m, 1F). IR (ATR): 3377, 2049, 2919, 1662, 1603, 1510, 1264, 1174, 1126, 969, 839, 693 cm^{-1}. HRMS (ESI, m/z): calcd for $C_{10}H_6F_4O^-$ (M–H)$^-$: 219.0439; Found: 219.0441.

(E)-1,1,1-Trifluoro-4-(2,6-difluorophenyl)but-3-en-2-ol (13d)

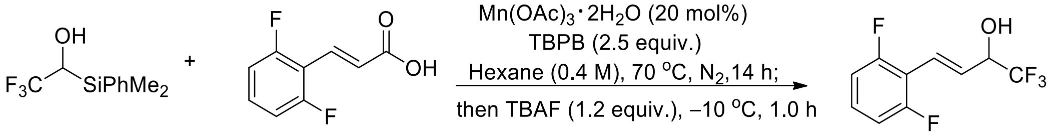

1a **12d** **13d**

Under N_2 atmosphere, to a dried 10 mL Schlenk tube equipped with a magnetic stir bar containing $Mn(OAc)_3$•$2H_2O$ (21.4 mg, 0.08 mmol, 20 mol%) and **12d** (147.2 mg, 0.8 mmol, 2.0 equiv.) was added hexane (1 mL, 0.4 M), TBPB (194.3 mg, 1.0 mmol, 2.5 equiv.) and **1a** (93.6 mg, 0.4 mmol) sequentially. The tube was sealed, and the resulting mixture was kept stirring at 70 °C in heating block for 14 h. The mixture was then cooled to −10 °C with low temperature bath, TBAF (1.0 M in THF, 0.48 mL, 0.48 mmol, 1.2 equiv.) was added and the resulting mixture was stirred at −10 °C for 1.0 h. The reaction mixture was quenched with water (2 mL), extracted with DCM (3×10 mL) and organic phase was combined and washed with brine, dried over Na_2SO_4, concentrated under reduced pressure. The crude product was purified with column chromatography on silica gel (200~300 mesh) and PE/EA (20/1~10/1, v/v) as eluent to afford 69.3 mg of the title compound as a colorless oil (67% yield).

R_f = 0.38 (PE/EA = 4/1, v/v). NMR Spectroscopy: ^{1}H NMR (400 MHz, $CDCl_3$) δ 7.26–7.18 (m, 1H), 6.95–6.88 (m, 3H), 6.56 (dd, J = 16.2, 6.1 Hz, 1H), 4.66 (d, J = 5.8 Hz, 1H), 2.58 (d, J = 5.2 Hz, 1H); ^{13}C NMR (100 MHz, $CDCl_3$) δ 161.2 (dd, J = 252.9, 7.2 Hz), 129.6 (t, J = 11.1 Hz), 127.5 (t, J = 7.2 Hz), 124.3 (d, J = 280.8 Hz), 122.6, 113.0 (t, J = 14.9 Hz), 111.8 (dd, J = 19.7, 6.3 Hz), 72.15 (q, J = 32.4 Hz); ^{19}F NMR (375 MHz, $CDCl_3$) δ –78.9 (d, J = 6.0 Hz, 3F), –112.6 (t, J = 7.5 Hz, 2F). IR (ATR): 3358, 2907, 2851, 1621, 1461, 1267, 1200, 1118, 995, 876, 782 cm^{-1}. HRMS (ESI, m/z): calcd for $C_{10}H_8F_5O^+$ $(M+H)^+$: 239.0490; Found: 239.0481.

(E)-1,1,1-Trifluoro-4-(2,4-difluorophenyl)but-3-en-2-ol (13e)

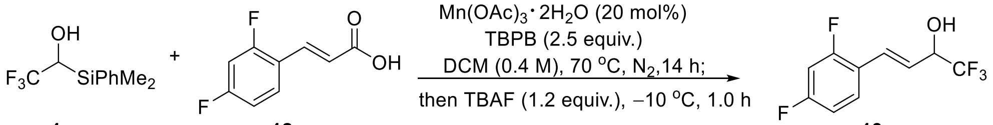

1a **12e** **13e**

Under N_2 atmosphere, to a dried 10 mL Schlenk tube equipped with a magnetic stir bar containing $Mn(OAc)_3$•$2H_2O$ (21.4 mg, 0.08 mmol, 20 mol%) and **12e** (147.2 mg, 0.8 mmol, 2.0 equiv.) was added DCM (1 mL, 0.4 M), TBPB (194.3 mg, 1.0 mmol, 2.5 equiv.) and **1a** (93.6 mg, 0.4 mmol) sequentially. The tube was sealed, and the resulting mixture was kept stirring at 70 °C in heating block for 14 h. The mixture was then cooled to −10 °C with low temperature bath, TBAF (1.0 M in THF, 0.48 mL, 0.48 mmol, 1.2 equiv.) was added and the resulting mixture was stirred at −10 °C for 1.0 h. The reaction mixture was quenched with water (2 mL), extracted with DCM (3×10 mL) and organic phase was combined and washed with brine, dried over Na_2SO_4, concentrated under reduced pressure. The crude product was purified with column chromatography on silica gel (200~300 mesh) and PE/EA (20/1~10/1, v/v) as eluent to afford 60.1 mg of the title compound as a white solid (63% yield).

R_f = 0.68 (PE/EA = 4/1, v/v). mp: 47 °C–49 °C NMR Spectroscopy: ^{1}H NMR (600 MHz, $CDCl_3$) δ 7.45 (td, J = 8.6, 6.3 Hz, 2H), 6.95 (d, J = 16.2 Hz, 1H), 6.91–6.85 (m, 2H), 6.85–6.79 (m, 2H), 6.24 (dd, J = 16.1, 6.3 Hz, 2H), 4.68–4.62 (m, 2H), 2.44 (d, J = 5.3 Hz, 2H); ^{13}C NMR (150 MHz, $CDCl_3$) δ 163.1 (dd, J = 249.6, 12.0 Hz), 160.8 (dd, J = 251.8, 11.7 Hz), 129.0 (dd, J = 9.6, 5.0 Hz), 128.1–128.1 (m), 124.3 (q, J = 282.1 Hz), 123.0–122.9 (m), 119.8 (dd, J = 12.1, 4.1 Hz), 111.9 (dd, J = 21.3, 3.7 Hz), 104.5 (t, J = 25.4 Hz), 71.8 (q, J = 32.0 Hz); ^{19}F NMR (375 MHz, $CDCl_3$) δ –79.0 (d, J = 6.0 Hz, 3F), –108.9– –108.8 (m, 1F), –112.7– –112.6 (m, 1F). IR (ATR): 3358, 2915, 1659, 1614, 1502, 1431, 1274, 1174, 1126, 969, 854, 731 cm^{-1}. HRMS (ESI, m/z): calcd for $C_{10}H_6F_5O^-$ $(M-H)^-$: 237.0344; Found: 237.0346.

(E)-1,1,1-trifluoro-4-(3,5-difluorophenyl)but-3-en-2-ol (13f)

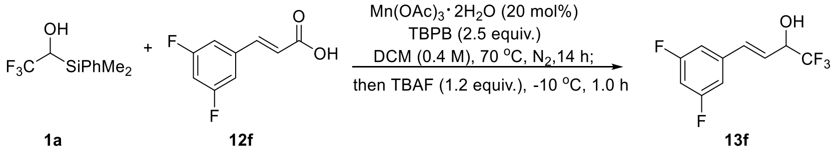

Under N_2 atmosphere, to a dried 10 mL Schlenk tube equipped with a magnetic stir bar containing $Mn(OAc)_3$•$2H_2O$ (21.4 mg, 0.08 mmol, 20 mol%) and **12f** (147.2 mg, 0.8 mmol, 2.0 equiv.) was added DCM (1 mL, 0.4 M), TBPB (194.3 mg, 1.0 mmol, 2.5 equiv.) and **1a** (93.6 mg, 0.4 mmol) sequentially. The tube was sealed, and the resulting mixture was kept stirring at 70 ºC in heating block for 14 h. The mixture was then cooled to −10 ºC with low temperature bath, TBAF (1.0 M in THF, 0.48 mL, 0.48 mmol, 1.2 equiv.) was added and the resulting mixture was stirred at −10 ºC for 1.0 h. The reaction mixture was quenched with water (2 mL), extracted with DCM (3×10 mL) and organic phase was combined and washed with brine, dried over Na_2SO_4, concentrated under reduced pressure. The crude product was purified with column chromatography on silica gel (200~300 mesh) and PE/EA (20/1~10/1, v/v) as eluent to afford 71.5 mg of the title compound as a colorless oil (75% yield).

R_f = 0.25 (PE/EA = 10/1, v/v). NMR Spectroscopy: ^{1}H NMR (600 MHz, $CDCl_3$) δ 6.94–6.92 (m, 2H), 6.80 (d, J = 16.0 Hz, 1H), 6.75 (tt, J = 8.8, 2.3 Hz, 1H), 6.22 (dd, J = 15.9, 5.9 Hz, 1H), 4.72–4.61 (m, 1H), 2.55 (s, 1H); ^{13}C NMR (150 MHz, $CDCl_3$) δ 163.4 (dd, J = 248.5, 13.0 Hz), 138.8 (t, J = 9.5 Hz), 134.0 (t, J = 2.8 Hz), 124.2 (q, J = 32.4 Hz), 123.5, 109.8 (dd, J = 20.4, 5.2 Hz), 104.1 (t, J = 25.5 Hz), 71.2 (q, J = 32.4 Hz); ^{19}F NMR (375 MHz, $CDCl_3$) δ –78.9 (d, J = 6.0 Hz, 3F), –109.6 (t, J = 8.9 Hz, 2F). IR (ATR): 3384, 3094, 2904, 1621, 1595, 1439, 1267, 1118, 969, 854, 667 cm^{-1}. HRMS (ESI, m/z): calcd for $C_{10}H_6F_5O^-$ $(M–H)^-$: 237.0344; Found: 237.0335.

(E)-1,1,1-trifluoro-4-(4-chlorophenyl)but-3-en-2-ol (13g)

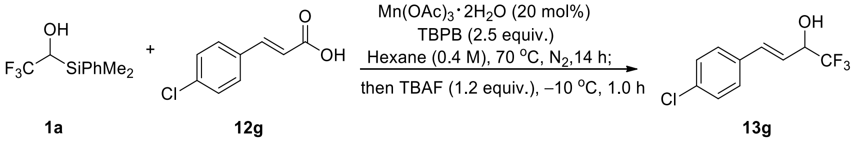

Under N_2 atmosphere, to a dried 10 mL Schlenk tube equipped with a magnetic stir bar containing $Mn(OAc)_3$•$2H_2O$ (21.4 mg, 0.08 mmol, 20 mol%) and **12g** (145.6 mg, 0.8 mmol, 2.0 equiv.) was added hexane (1 mL, 0.4 M), TBPB (194.3 mg, 1.0 mmol, 2.5 equiv.) and **1a** (93.6 mg, 0.4 mmol) sequentially. The tube was sealed, and the resulting mixture was kept stirring at 70 ºC in heating block for 14 h. The mixture was then cooled to −10 ºC with low temperature bath, TBAF (1.0 M in THF, 0.48 mL, 0.48 mmol, 1.2 equiv.) was added and the resulting mixture was stirred at −10 ºC for 1.0 h. The reaction mixture was quenched with water (2 mL), extracted with DCM (3×10 mL) and organic phase was combined and washed with brine, dried over Na_2SO_4, concentrated under reduced pressure. The crude product was purified with column chromatography on silica gel (200~300 mesh) and PE/EA (20/1~10/1, v/v) as eluent to afford 63.2 mg of the title compound as a white solid (67% yield).

R_f = 0.22 (PE/EA = 10/1, v/v). mp: 41 ºC–42 ºC. NMR Spectroscopy: ^{1}H NMR (400 MHz, $CDCl_3$) δ 7.36–7.31 (m, 4H), 6.82 (d, J = 15.9 Hz, 1H), 6.18 (dd, J = 15.9, 6.1 Hz, 1H), 4.65–4.63 (m, 1H), 2.44 (d, J = 4.0 Hz, 1H); ^{13}C NMR (100 MHz, $CDCl_3$) δ 135.1, 134.7, 134.0, 129.1, 128.3, 124.3 (q, J = 279.4 Hz), 121.4, 71.6 (q, J = 32.1 Hz); ^{19}F NMR (375 MHz, $CDCl_3$) δ –79.0 (d, J = 6.0 Hz, 3F). IR (ATR): 3399, 2911, 1491, 1267, 1129, 1092, 969, 831 cm^{-1}. HRMS (ESI, m/z): calcd for $C_{10}H_7ClF_3O^-$ $(M–H)^-$: 235.0143; Found: 237.0146.

(E)-1,1,1-trifluoro-4-(4-bromophenyl)but-3-en-2-ol (13h)

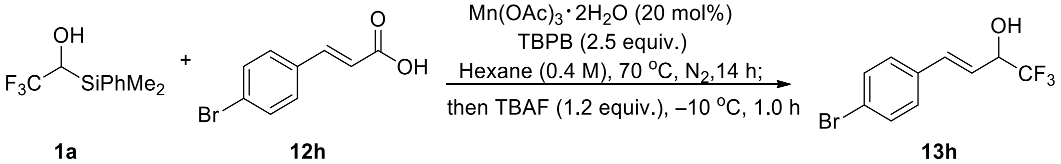

Under N_2 atmosphere, to a dried 10 mL Schlenk tube equipped with a magnetic stir bar containing $Mn(OAc)_3$•$2H_2O$

(21.4 mg, 0.08 mmol, 20 mol%) and **5e** (181.6 mg, 0.8 mmol, 2.0 equiv.) was added hexane (1 mL, 0.4 M), TBPB (194.3 mg, 1.0 mmol, 2.5 equiv.) and **3a** (93.6 mg, 0.4 mmol) sequentially. The tube was sealed, and the resulting mixture was kept stirring at 70 °C in heating block for 14 h. The mixture was then cooled to −10 °C with low temperature bath, TBAF (1.0 M in THF, 0.48 mL, 0.48 mmol, 1.2 equiv.) was added and the resulting mixture was stirred at −10 °C for 1.0 h. The reaction mixture was quenched with water (2 mL), extracted with DCM (3×10 mL) and organic phase was combined and washed with brine, dried over Na_2SO_4, concentrated under reduced pressure. The crude product was purified with column chromatography on silica gel (200~300 mesh) and PE/EA (20/1~10/1, v/v) as eluent to afford 68.6 mg of the title compound as a white solid (61% yield).

R_f = 0.23 (PE/EA = 10/1, v/v). mp: 55 °C–57 °C. NMR Spectroscopy: ^{1}H NMR (400 MHz, $CDCl_3$) δ 7.48 (d, J = 8.3 Hz, 2H), 7.28 (d, J = 8.3 Hz, 2H), 6.81 (d, J = 16.2 Hz, 1H), 6.19 (dd, J = 16.0, 6.3 Hz, 1H), 4.63 (bs, 1H), 2.46 (s, 1H); ^{13}C NMR (100 MHz, $CDCl_3$) δ 135.1, 134.5, 132.1, 128.5, 124.3 (q, J = 280.8 Hz), 122.8, 121.6, 71.6 (q, J = 32.4 Hz); ^{19}F NMR (375 MHz, $CDCl_3$) δ –78.9 (d, J = 6.0 Hz, 3F). IR (ATR): 3340, 2926, 1655, 1487, 1267, 1178, 1126, 977, 823, 697 cm^{-1}. HRMS (ESI, m/z): calcd for $C_{10}H_7BrF_3O^-$ (M–H)$^-$: 278.9638; Found: 278.9640.

(E)-1,1,1-trifluoro-4-(4-methyloxyphenyl)but-3-en-2-ol (13i)

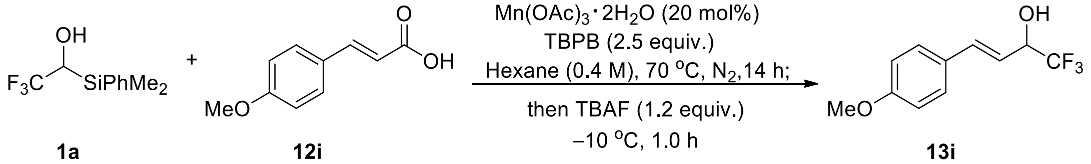

Under N_2 atmosphere, to a dried 10 mL Schlenk tube equipped with a magnetic stir bar containing $Mn(OAc)_3$•$2H_2O$ (21.4 mg, 0.08 mmol, 20 mol%) and **12i** (142.4 mg, 0.8 mmol, 2.0 equiv.) was added hexane (1 mL, 0.4 M), TBPB (194.3 mg, 1.0 mmol, 2.5 equiv.) and **1a** (93.6 mg, 0.4 mmol) sequentially. The tube was sealed, and the resulting mixture was kept stirring at 70 °C in heating block for 14 h. The mixture was then cooled to −10 °C with low temperature bath, TBAF (1.0 M in THF, 0.48 mL, 0.48 mmol, 1.2 equiv.) was added and the resulting mixture was stirred at −10 °C for 1.0 h. The reaction mixture was quenched with water (2 mL), extracted with DCM (3×10 mL) and organic phase was combined and washed with brine, dried over Na_2SO_4, concentrated under reduced pressure. The crude product was purified with column chromatography on silica gel (200~300 mesh) and PE/EA (20/1~10/1, v/v) as eluent to afford 50.1 mg of the title compound as a white solid (54% yield).

R_f = 0.38 (PE/EA = 4/1, v/v). mp: 47 °C–49 °C. NMR Spectroscopy: ^{1}H NMR (400 MHz, $CDCl_3$) δ 7.36 (d, J = 8.6 Hz, 2H), 6.88 (d, J = 8.6 Hz, 2H), 6.79 (d, J = 15.9 Hz, 1H), 6.06 (dd, J = 15.9, 6.7 Hz, 1H), 4.63–4.57 (m, 1H), 3.82 (s, 3H), 2.42 (s, 1H); ^{13}C NMR (100 MHz, $CDCl_3$) δ 160.2, 136.1, 128.4, 128.3, 124.5 (d, J = 280.8 Hz), 118.5, 114.3, 72.0 (q, J = 32.4 Hz, 03), 55.5; ^{19}F NMR (375 MHz, $CDCl_3$) δ –79.0 (d, J = 6.0 Hz, 3F). IR (ATR): 3407, 3008, 2960, 2840, 1606, 1513, 1252, 1170, 1126, 1033, 969, 835, 693 cm^{-1}. HRMS (ESI, m/z): calcd for $C_{11}H_{12}F_3O_2^+$ (M+H)$^+$: 233.0784; Found: 233.0783.

(E)-1,1,1-trifluoro-4-(4-benzyloxyphenyl)but-3-en-2-ol (13j)

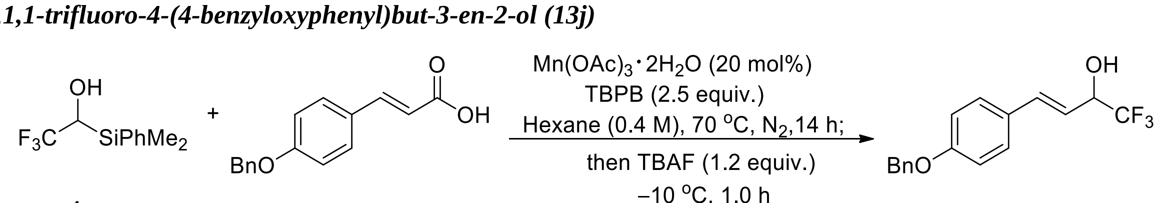

Under N_2 atmosphere, to a dried 10 mL Schlenk tube equipped with a magnetic stir bar containing $Mn(OAc)_3$•$2H_2O$ (21.4 mg, 0.08 mmol, 20 mol%) and **12j** (203.2 mg, 0.8 mmol, 2.0 equiv.) was added hexane (1 mL, 0.4 M), TBPB (194.3 mg, 1.0 mmol, 2.5 equiv.) and **1a** (93.6 mg, 0.4 mmol) sequentially. The tube was sealed, and the resulting mixture was kept stirring at 70 °C in heating block for 14 h. The mixture was then cooled to −10 °C with low temperature bath, TBAF (1.0 M in THF, 0.48 mL, 0.48 mmol, 1.2 equiv.) was added and the resulting mixture was stirred at −10 °C for 1.0 h. The reaction mixture was quenched with water (2 mL), extracted with DCM (3×10 mL) and organic phase was

combined and washed with brine, dried over Na_2SO_4, concentrated under reduced pressure. The crude product was purified with column chromatography on silica gel (200~300 mesh) and PE/EA (20/1~10/1, v/v) as eluent to afford 62.9 mg of the title compound as a white solid (51% yield).

R_f = 0.40 (PE/EA = 4/1, v/v). mp: 89 °C–91 °C. NMR Spectroscopy: ^{1}H NMR (400 MHz, $CDCl_3$) δ 7.45–7.34 (m, 7H), 6.96 (d, J = 8.6 Hz, 2H), 6.79 (d, J = 15.9 Hz, 1H), 6.07 (dd, J = 16.0, 6.9 Hz, 1H), 5.09 (s, 2H), 4.64–4.56 (m, 1H), 2.33 (d, J = 5.5 Hz, 1H); ^{13}C NMR (100 MHz, $CDCl_3$) δ 159.4, 136.8, 136.1, 128.8, 128.5, 128.4, 128.2, 127.6, 124.5 (d, J = 279.9 Hz), 118.6, 115.2, 71.9 (q, J = 32.1 Hz), 70.2; ^{19}F NMR (375 MHz, $CDCl_3$) δ –79.0 (d, J = 6.0 Hz, 3F). IR (ATR): 3373, 3037, 2922, 2855, 1610, 1513, 1454, 1264, 1170, 1125, 1036, 977, 738, 697 cm^{-1}. HRMS (ESI, m/z): calcd for $C_{17}H_{16}F_3O_2^+$ $(M+H)^+$: 309.1097; Found: 309.1089.

(E)-1,1,1-trifluoro-4-((3-trifluoromethyl)phenyl)but-3-en-2-ol (13k)

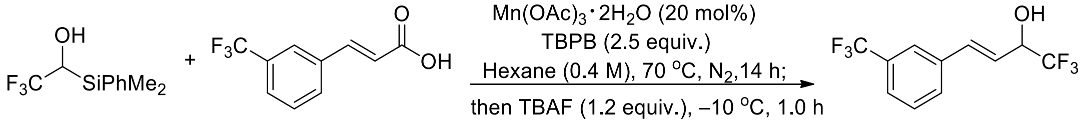

1a **12k** **13k**

Under N_2 atmosphere, to a dried 10 mL Schlenk tube equipped with a magnetic stir bar containing $Mn(OAc)_3$•$2H_2O$ (21.4 mg, 0.08 mmol, 20 mol%) and **12k** (172.8 mg, 0.8 mmol, 2.0 equiv.) was added hexane (1 mL, 0.4 M), TBPB (194.3 mg, 1.0 mmol, 2.5 equiv.) and **1a** (93.6 mg, 0.4 mmol) sequentially. The tube was sealed, and the resulting mixture was kept stirring at 70 °C in heating block for 14 h. The mixture was then cooled to –10 °C with low temperature bath, TBAF (1.0 M in THF, 0.48 mL, 0.48 mmol, 1.2 equiv.) was added and the resulting mixture was stirred at –10 °C for 1.0 h. The reaction mixture was quenched with water (2 mL), extracted with DCM (3×10 mL) and organic phase was combined and washed with brine, dried over Na_2SO_4, concentrated under reduced pressure. The crude product was purified with column chromatography on silica gel (200~300 mesh) and PE/EA (20/1~10/1, v/v) as eluent to afford 70.6 mg of the title compound as a colorless oil (65% yield).

R_f = 0.46 (PE/EA = 4/1, v/v). NMR Spectroscopy: ^{1}H NMR (400 MHz, $CDCl_3$) δ 7.66–7.46 (m, 4H), 6.92 (d, J = 16.2 Hz, 1H), 6.28 (dd, J = 16.0, 6.0 Hz, 1H), 4.68 (d, J = 5.8 Hz, 1H), 2.48 (d, J = 4.9 Hz, 1H); ^{13}C NMR (150 MHz, $CDCl_3$) δ 136.3, 134.7, 131.4 (q, J = 32.1 Hz), 130.1, 129.4, 125.4 (q, J = 3.7 Hz), 124.3 (q, J = 280.4 Hz), 124.1 (q, J = 270.8 Hz), 123.7 (q, J = 3.8 Hz), 122.7, 71.4 (q, J = 32.1 Hz); ^{19}F NMR (375 MHz, $CDCl_3$) δ –62.8 (s, 3F), –78.9 (d, J = 6.0 Hz, 3F). IR (ATR): 3370, 3053, 2922, 1331, 1267, 1167, 1122, 1074, 794, 693 cm^{-1}. HRMS (ESI, m/z): calcd for $C_{11}H_7F_6O^-$ $(M–H)^-$: 269.0407; Found: 269.0409.

(E)-1,1,1-trifluoro-4-((4-trifluoromethyl)phenyl)but-3-en-2-ol (13l)

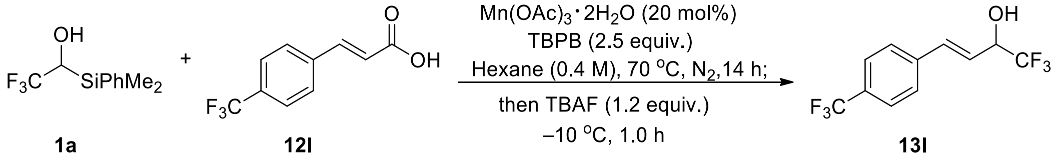

1a **12l** **13l**

Under N_2 atmosphere, to a dried 10 mL Schlenk tube equipped with a magnetic stir bar containing $Mn(OAc)_3$•$2H_2O$ (21.4 mg, 0.08 mmol, 20 mol%) and **12l** (172.8 mg, 0.8 mmol, 2.0 equiv.) was added hexane (1 mL, 0.4 M), TBPB (194.3 mg, 1.0 mmol, 2.5 equiv.) and **1a** (93.6 mg, 0.4 mmol) sequentially. The tube was sealed, and the resulting mixture was kept stirring at 70 °C in heating block for 14 h. The mixture was then cooled to –10 °C with low temperature bath, TBAF (1.0 M in THF, 0.48 mL, 0.48 mmol, 1.2 equiv.) was added and the resulting mixture was stirred at –10 °C for 1.0 h. The reaction mixture was quenched with water (2 mL), extracted with DCM (3×10 mL) and organic phase was combined and washed with brine, dried over Na_2SO_4, concentrated under reduced pressure. The crude product was purified with column chromatography on silica gel (200~300 mesh) and PE/EA (20/1~10/1, v/v) as eluent to afford 77.8 mg of the title compound as a colorless oil (72% yield).

R_f = 0.43 (PE/EA = 4/1, v/v). NMR Spectroscopy: ^{1}H NMR (400 MHz, $CDCl_3$) δ 7.60 (d, J = 7.9 Hz, 2H), 7.51 (d, J =

7.6 Hz, 2H), 6.91 (d, J = 15.9 Hz, 1H), 6.30 (dd, J = 15.9, 5.8 Hz, 1H), 4.70–4.67 (m, 1H), 2.66 (bs, 1H); ^{13}C NMR (100 MHz, $CDCl_3$) δ 139.0, 134.7, 130.6 (q, J = 32.4 Hz), 127.2, 125.9 (q, J = 3.9 Hz), 124.3 (q, J = 280 .5 Hz), 124.2 (q, J = 270.7 Hz), 123.4, 71.4 (q, J = 32.4 Hz); ^{19}F NMR (375 MHz, $CDCl_3$) δ –62.6 (s, 3F), –78.9 (d, J = 6.0 Hz, 3F). IR (ATR): 3377, 2922, 1618, 1416, 1323, 1167, 1122, 969, 835, 697 cm^{-1}. HRMS (ESI, m/z): calcd for $C_{11}H_7F_6O^-$ (M–H)$^-$: 269.0407; Found: 269.0407.

Synthesis of α-difluoromethylated alcohols

5,5-Difluoro-1-phenyl-4-hydroxy-2-methylenepentan-1-one (14a)

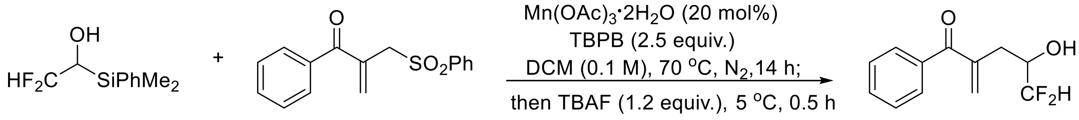

2a **7b** **14a**

Under N_2 atmosphere, to a dried 10 mL Schlenk tube equipped with a magnetic stir bar containing $Mn(OAc)_3$•$2H_2O$ (16.1 mg, 0.06 mmol, 20 mol%) and **7b** (257.4 mg, 0.9 mmol, 3.0 equiv.) was added DCM (3 mL, 0.1 M), **2a** (64.8 mg, 0.3 mmol) and TBPB (145.7 mg, 0.75 mmol, 2.5 equiv.) sequentially. The tube was then sealed, and the resulting mixture was stirred at 70 ºC in a heating block for 14 h, after which the mixture was cooled to 5 ºC with ice bath. TBAF (1.0 M in THF, 0.36 mL, 0.36 mmol, 1.2 equiv.) was added and the mixture was stirred at 5 ºC for 0.5 h. The reaction mixture was quenched with water (2 mL) and extracted with DCM (3×10 mL). The organic phase was combined and washed with brine, dried over Na_2SO_4, concentrated under reduced pressure. The crude product was purified with column chromatography on silica gel (200~300 mesh) and PE/EA (20/1 v/v) as eluent to afford 54.3 mg of the title compound as a colorless oil (80% yield).

R_f = 0.25 (PE/EA = 10/1, v/v). NMR Spectroscopy: ^{1}H NMR (400 MHz, $CDCl_3$) δ 7.77 (d, J = 7.3 Hz, 2H), 7.58 (t, J = 7.3 Hz, 1H), 7.45 (t, J = 7.6 Hz, 2H), 6.11 (s, 1H), 5.86–5.58 (m, 2H), 3.98–3.91 (m, 1H), 3.83 (s, 1H), 2.84–2.63 (m, 2H); ^{13}C NMR (100 MHz, $CDCl_3$) δ 199.5, 143.1, 137.0, 133.0, 130.9, 130.0, 128.5, 116.1 (t, J = 244.2 Hz), 70.9 (t, J = 24.1 Hz), 33.7; ^{19}F NMR (375 MHz, $CDCl_3$) δ –128.5– –131.2 (m, 2F). IR (ATR): 3452, 3064, 2941, 2292, 2251, 1655, 1446, 1409, 1375, 1330, 1219, 1174, 1140, 1059, 947, 757 cm^{-1}. HRMS (ESI, m/z): calcd for $C_{12}H_{13}F_2O_2^+$ (M+H)$^+$: 227.0878; Found: 227.0871.

5,5-Difluoro-1-(4-fluorophenyl)-4-hydroxy-2-methylenepentan-1-one (14b)

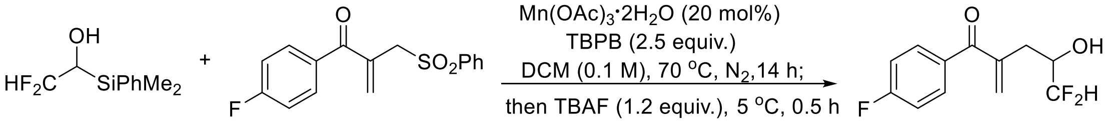

2a **7d** **14b**

Under N_2 atmosphere, to a dried 10 mL Schlenk tube equipped with a magnetic stir bar containing $Mn(OAc)_3$•$2H_2O$ (16.1 mg, 0.06 mmol, 20 mol%) and **7d** (273.6 mg, 0.9 mmol, 3.0 equiv.) was added DCM (3 mL, 0.1 M), **2a** (64.8 mg, 0.3 mmol) and TBPB (145.7 mg, 0.75 mmol, 2.5 equiv.) sequentially. The tube was then sealed, and the resulting mixture was stirred at 70 ºC in a heating block for 14 h, after which the mixture was cooled to 5 ºC with ice bath. TBAF (1.0 M in THF, 0.36 mL, 0.36 mmol, 1.2 equiv.) was added and the mixture was stirred at 5 ºC for 0.5 h. The reaction mixture was quenched with water (2 mL) and extracted with DCM (3×10 mL). The organic phase was combined and washed with brine, dried over Na_2SO_4, concentrated under reduced pressure. The crude product was purified with column chromatography on silica gel (200~300 mesh) and PE/EA (20/1~10/1, v/v) as eluent to afford 48.3 mg of the title compound as a colorless oil (66% yield).

R_f = 0.32 (PE/EA = 5/1, v/v). NMR Spectroscopy: ^{1}H NMR (400 MHz, $CDCl_3$) δ 7.86–7.79 (m, 2H), 7.18–7.09 (m, 2H), 6.09 (s, 1H), 5.87–5.56 (m, 2H), 4.02–3.85 (m, 1H), 3.65 (m, 1H), 2.86–2.59 (m, 2H); ^{13}C NMR (100 MHz, $CDCl_3$) δ 197.8, 165.8 (d, J = 252.3 Hz), 143.1, 133.2 (d, J = 3.2 Hz), 132.7 (d, J = 9.1 Hz), 130.2, 116.1 (t, J = 242.0 Hz), 115.7 (d, J = 21.9 Hz), 70.9 (t, J = 23.7 Hz), 33.7 (t, J = 4.1 Hz); ^{19}F NMR (375 MHz, $CDCl_3$) δ –104.9 (m, 1F), –126.2– –132.7 (m, 2F). IR (ATR): 3422, 2926, 1648, 1595, 1506, 1413, 1156, 1059, 939, 794 cm^{-1}. HRMS (ESI, m/z): calcd for $C_{12}H_{12}F_3O_2^+$ (M+H)$^+$: 267.0603; Found: 267.0604.

5,5-Difluoro-4-hydroxy-2-methylene-N,N-diphenylpentanamide (14c)

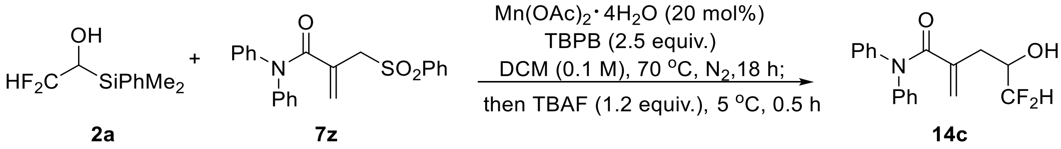

Under N_2 atmosphere, to a dried 10 mL Schlenk tube equipped with a magnetic stir bar containing $Mn(OAc)_2$•$4H_2O$ (14.7 mg, 0.06 mmol, 20 mol%), **7z** (339.3 mg, 0.9 mmol, 3.0 equiv.) was added DCM (3 mL, 0.1 M), **2a** (64.8 mg, 0.3 mmol) and TBPB (145.7 mg, 0.75 mmol, 2.5 equiv.) sequentially. The tube was sealed, and the resulting mixture was kept stirring at 70 °C in heating block for 18 h. After which the mixture was cooled to 5 °C with ice bath, TBAF (1.0 M in THF, 0.36 mL, 0.36 mmol, 1.2 equiv.) was added and the mixture was stirred at 5 °C for 0.5 h. The reaction mixture was quenched with water (2 mL), extracted with DCM (3×10 mL) and organic phase was combined and washed with brine, dried over Na_2SO_4, concentrated under reduced pressure. The crude product was purified with column chromatography on silica gel (200~300 mesh) and PE/EA (3/1, v/v) as eluent to afford 68.2 mg of the title compound as a pale yellow oil (72% yield).

R_f = 0.55 (PE/EA = 3/1, v/v). NMR Spectroscopy: ^{1}H NMR (400 MHz, $CDCl_3$) δ 7.40–7.33 (m, 4H), 7.29–7.23 (m, 2H), 7.21–7.16 (m, 4H), 5.71 (td, J = 56.0, 4.0 Hz, 1H), 5.40 (s, 1H), 5.29 (s, 1H), 4.01–3.85 (m, 1H), 2.60–2.42 (m, 2H); ^{13}C NMR (100 MHz, $CDCl_3$) δ 172.6, 143.3, 139.6, 129.5, 127.3, 125.0, 116.1 (t, J = 241.8 Hz), 71.5 (t, J = 24.1 Hz), 34.7 (t, J = 4.3 Hz); ^{19}F NMR (375 MHz, $CDCl_3$) δ –127.5– –132.3 (m, 2F). IR (ATR): 3366, 2970, 1648, 1588, 1491, 1361, 1252, 1137, 760, 697 cm^{-1}. HRMS (ESI, m/z): calcd for $C_{18}H_{18}F_2NO_2^+$ (M+H)$^+$: 318.1300; Found: 318.1298.

(8R,9S,10R,13S,14S,17S)-10,13-Dimethyl-3-oxo-2,3,6,7,8,9,10,11,12,13,14,15,16,17-tetradecahydro-1H-cyclopenta[a] phenanthren-17-yl 5,5-difluoro-4-hydroxy-2-methylenepentanoate (14d)

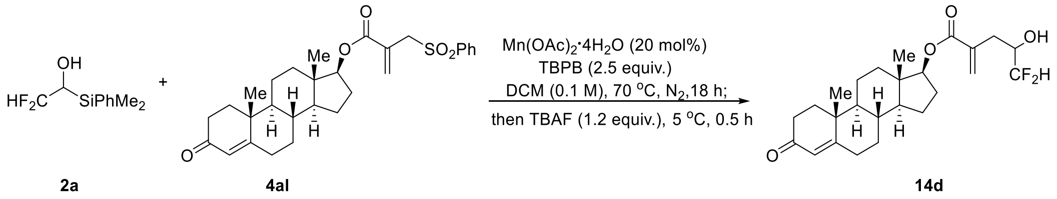

Under N_2 atmosphere, to a dried 10 mL Schlenk tube equipped with a magnetic stir bar containing with $Mn(OAc)_2$•$4H_2O$ (14.7 mg, 0.06 mmol, 20 mol%), **7al** (297.6 mg, 0.6 mmol, 2.0 equiv.) was added DCM (3 mL, 0.1 M), **2a** (70.2 mg, 0.3 mmol) and TBPB (145.7 mg, 0.75 mmol, 2.5 equiv.) sequentially. The tube was sealed, and the resulting mixture was kept stirring at 70 °C in heating block for 18 h. After which the mixture was cooled to 5 °C with ice bath, TBAF (1.0 M in THF, 0.36 mL, 0.36 mmol, 1.2 equiv.) was added and the mixture was stirred at 5 °C for 0.5 h. The reaction mixture was quenched with water (2 mL), extracted with DCM (3×10 mL) and organic phase was combined and washed with brine, dried over Na_2SO_4, concentrated under reduced pressure. The crude product was purified with column chromatography on silica gel (200~300 mesh) and PE/EA (8/1, v/v) as eluent to afford 109.0 mg of the title compound as a white solid (83% yield).

R_f = 0.37 (PE/EA = 2/1, v/v). mp: 87.3 °C–88.9 °C. NMR Spectroscopy: ^{1}H NMR (400 MHz, $CDCl_3$) δ 6.31 (s, 1H), 5.86–5.52 (m, 3H), 4.72–4.62 (m, 1H), 3.92 (q, J = 12.8, 11.4 Hz, 1H), 2.70 (dd, J = 13.3, 2.9 Hz, 1H), 2.51 (dd, J = 14.2, 8.6 Hz, 1H), 2.46–2.17 (m, 5H), 2.05–1.98 (m, 1H), 1.90–1.76 (m, 2H), 1.75–1.64 (m, 2H), 1.64–1.52 (m, 3H), 1.47–1.33 (m, 2H), 1.30–1.22 (m, 1H), 1.18 (s, 3H), 1.14–0.90 (m, 3H), 0.88 (s, 3H); ^{13}C NMR (100 MHz, $CDCl_3$) δ 199.7, 171.1, 167.8, 136.1, 129.0, 124.1, 116.0 (t, J = 241.8 Hz), 83.7, 70.4 (td, J = 23.7, 4.4 Hz), 53.8, 50.3, 42.9 (d, J = 3.0 Hz), 38.7, 36.8 (d, J = 2.8 Hz), 35.8, 35.5, 34.0, 33.3 (q, J = 3.5 Hz), 32.8, 31.6, 27.6, 23.6, 20.6, 17.5, 12.3; ^{19}F NMR (375 MHz, $CDCl_3$) δ –128.5– –131.6 (m, 2F). IR (ATR): 3422, 2945, 1715, 1435, 1312, 1200, 1156, 1058, 943, 731 cm^{-1}. HRMS (ESI, m/z): calcd for $C_{25}H_{34}F_2O_4Na^+$ (M+Na)$^+$: 459.2317; Found: 459.2315.

(4S,5'R,6aR,6bS,8aS,8bR,9S,10R,11aS,12aS,12bS)-5',6a,8a,9-Tetramethyl-1,3,3',4,4',5,5',6,6a,6b,6',7,8,8a,8b,9,11a,12,12a,12b-icosahydrospiro[naphtho[2',1':4,5]indeno[2,1-b]furan-10,2'-pyran]-4-yl 5,5-difluoro-4-hydroxy-2-methylenepentanoatee (14e)

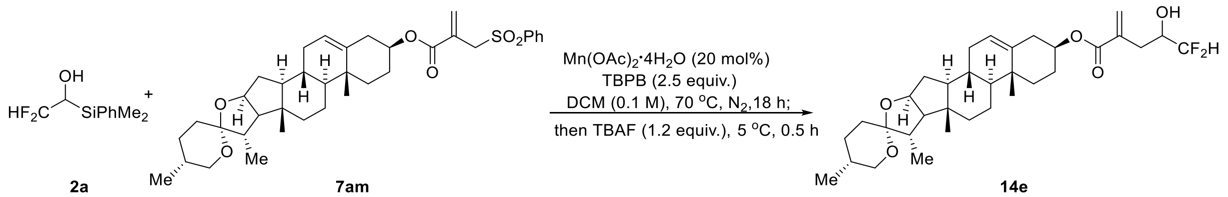

2a **7am** **14e**

Under N_2 atmosphere, to a dried 10 mL Schlenk tube equipped with a magnetic stir bar containing $Mn(OAc)_2$•$4H_2O$ (14.7 mg, 0.06 mmol, 20 mol%), **7am** (373.5 mg, 0.6 mmol, 2.0 equiv.) was added DCM (3 mL, 0.1 M), **2a** (70.2 mg, 0.3 mmol) and TBPB (145.7 mg, 0.75 mmol, 2.5 equiv.) sequentially. The tube was sealed, and the resulting mixture was kept stirring at 70 ºC in heating block for 18 h. After which the mixture was cooled to 5 ºC with ice bath, TBAF (1.0 M in THF, 0.36 mL, 0.36 mmol, 1.2 equiv.) was added and the mixture was stirred at 5 ºC for 0.5 h. The reaction mixture was quenched with water (2 mL), extracted with DCM (3×10 mL) and organic phase was combined and washed with brine, dried over Na_2SO_4, concentrated under reduced pressure. The crude product was purified with column chromatography on silica gel (200~300 mesh) and PE/EA (12/1, v/v) as eluent to afford 112.9 mg of the title compound as a white solid (67 % yield).

R_f = 0.42 (PE/EA = 5/1, v/v). mp: 126.1 ºC–127.4 ºC. NMR Spectroscopy: ^{1}H NMR (400 MHz, $CDCl_3$) δ 6.29 (d, J = 1.3 Hz, 1H), 5.84–5.52 (m, 2H), 5.39 (d, J = 5.1 Hz, 1H), 4.74–4.61 (m, 1H), 4.40 (dd, J = 14.3, 8.1 Hz, 1H), 3.98–3.84 (m, 1H), 3.50–3.43 (m, 1H), 3.36 (t, J = 10.9 Hz, 1H), 2.69 (dd, J = 14.4, 3.3 Hz, 1H), 2.51 (dd, J = 14.4, 9.0 Hz, 1H), 2.37 (d, J = 7.9 Hz, 2H), 2.06–1.40 (m, 18H), 1.02 (s, 3H), 0.96 (d, J = 7.0 Hz, 3H), 0.78 (d, J = 4.8 Hz, 6H); ^{13}C NMR (100 MHz, $CDCl_3$) δ 167.4, 139.5, 136.3, 122.8, 116.0 (t, J = 244.3 Hz), 109.4, 80.9, 75.3, 70.6 (t, J = 23.8 Hz), 67.0, 62.2, 56.6, 41.7, 40.4, 39.8, 38.1, 37.0, 36.9, 33.4 (t, J = 4.0 Hz), 32.2, 32.0, 31.5, 28.9, 27.8, 20.9, 19.5, 17.2, 16.4, 14.6; ^{19}F NMR (375 MHz, $CDCl_3$) δ –128.7– –131.6 (m, 2F). IR (ATR): 3418, 2945, 1710, 1454, 1375, 1327, 1245, 1051, 980, 83 cm^{-1}. HRMS (ESI, m/z): calcd for $C_{33}H_{48}F_3O_5^+$ $(M+H)^+$: 563.3543; Found: 563.3533.

3-(3,3-Difluoro-2-hydroxypropyl)-3-methyl-1-phenylindolin-2-one (14f)

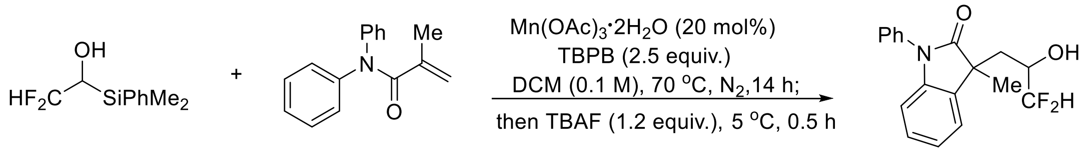

2a **10a** **14f**

Under N_2 atmosphere, to a dried 10 mL Schlenk tube equipped with a magnetic stir bar containing $Mn(OAc)_3$•$2H_2O$ (16.1 mg, 0.06 mmol, 20 mol%) and **10a** (85.3 mg, 0.36 mmol, 1.2 equiv.) was added DCM (3 mL, 0.1 M), **2a** (64.8 mg, 0.3 mmol) and TBPB (145.7 mg, 0.75 mmol, 2.5 equiv.) sequentially. The tube was then sealed, and the resulting mixture was stirred at 70 ºC in a heating block for 14 h, after which the mixture was cooled to 5 ºC with ice bath. TBAF (1.0 M in THF, 0.36 mL, 0.36 mmol, 1.2 equiv.) was added and the mixture was stirred at 5 ºC for 0.5 h. The reaction mixture was quenched with water (2 mL) and extracted with DCM (3×10 mL). The organic phase was combined and washed with saturated Na_2CO_3 and brine, dried over Na_2SO_4, concentrated under reduced pressure. The crude product was purified with column chromatography on silica gel (200~300 mesh) and PE/EA (20/1~10/1, v/v) as eluent to afford 76.5 mg of the title compound **14f** (81% yield, **14f-a**:**14f-b** = 48:52)**.**

R_f (**14f-a**) = 0.29 (PE/EA = 5/1, v/v), (37.0 mg, 39% yield, pale yellow oil). NMR Spectroscopy: ^{1}H NMR (400 MHz, $CDCl_3$) δ 7.58–7.52 (m, 2H), 7.47–7.40 (m, 3H), 7.30–7.28 (m, 1H), 7.24 (td, J = 7.7, 1.4 Hz, 1H), 7.18–7.14 (m, 1H), 6.86–6.84 (m, 1H), 5.83–5.54 (m, 1H), 4.51 (s, 1H), 4.26–4.16 (m, 1H), 2.29–1.93 (m, 2H), 1.60 (s, 3H); ^{13}C NMR (100 MHz, $CDCl_3$) δ 181.7, 142.4, 134.5, 134.0, 129.8, 128.6, 128.4, 126.6, 123.9, 122.9, 116.1 (dd, J = 242.7, 240.6 Hz), 110.1, 68.7 (dd, J = 25.0, 23.4 Hz), 46.8, 36.7–36.0 (m), 23.1; ^{19}F NMR (375 MHz, $CDCl_3$) δ –126.5– –130.8 (m, 2F). IR (ATR): 3399, 2967, 1707, 1610, 1502, 1379, 1204, 1055, 757, 697 cm^{-1}. HRMS (ESI, m/z): calcd for $C_{18}H_{17}F_2NO_2Na^+$ $(M+Na)^+$: 340.1120; Found: 340.1116

Rf (**14f-b**) = 0.23 (PE/EA = 5/1, v/v), (39.5 mg, 42% yield, white solid, mp: 136.1 °C–137.4 °C). NMR Spectroscopy: ^{1}H NMR (400 MHz, $CDCl_3$) δ 7.54–7.48 (m, 2H), 7.43–7.38 (m, 3H), 7.25–7.19 (m, 2H), 7.17–7.08 (m, 1H), 6.83 (dt, J = 7.5, 0.8 Hz, 1H), 5.55 (td, J = 56.2, 3.9 Hz, 1H), 3.53–3.30 (m, 1H), 2.43–2.13 (m, 2H), 1.53 (s, 3H); ^{13}C NMR (100 MHz, $CDCl_3$) δ 180.8, 143.7, 134.8, 132.2, 129.7, 128.3, 128.2, 126.8, 123.2, 123.0, 116.0 (t, J = 242.5 Hz), 109.8, 69.1 (t, J = 23.9 Hz), 46.2, 37.8, 25.7; ^{19}F NMR (375 MHz, $CDCl_3$) δ –126.5– –132.5 (m, 2F). IR (ATR): 3399, 2926, 1707, 1454, 1379, 1297, 1208, 1137, 1059, 760 cm^{-1}. HRMS (ESI, m/z): calcd for $C_{18}H_{18}F_2NO_2H^+$ $(M+H)^+$: 318.1300; Found: 318.1298.

1-Benzyl-3-(3,3-difluoro-2-hydroxypropyl)-3-methylindolin-2-one (14g)

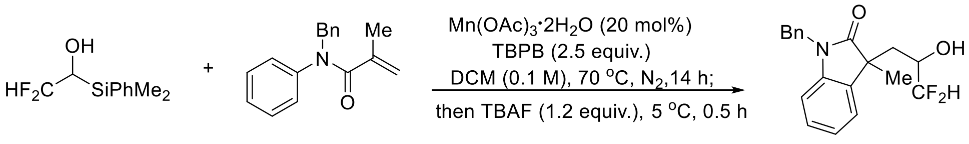

2a **10g** **14g**

Under N_2 atmosphere, to a dried 10 mL Schlenk tube equipped with a magnetic stir bar containing $Mn(OAc)_3 \cdot 2H_2O$ (16.1 mg, 0.06 mmol, 20 mol%) and **10g** (90.4 mg, 0.36 mmol, 1.2 equiv.) was added DCM (3 mL, 0.1 M), **2a** (64.8 mg, 0.3 mmol) and TBPB (145.7 mg, 0.75 mmol, 2.5 equiv.) sequentially. The tube was then sealed, and the resulting mixture was stirred at 70 °C in a heating block for 14 h, after which the mixture was cooled to 5 °C with ice bath. TBAF (1.0 M in THF, 0.36 mL, 0.36 mmol, 1.2 equiv.) was added and the mixture was stirred at 5 °C for 0.5 h. The reaction mixture was quenched with water (2 mL) and extracted with DCM (3×10 mL). The organic phase was combined and washed with saturated Na_2CO_3 and brine, dried over Na_2SO_4, concentrated under reduced pressure. The crude product was purified with column chromatography on silica gel (200~300 mesh) and PE/EA (20/1~10/1, v/v) as eluent to afford 77.6 mg of the title compound **14g** (78% yield, **14g-a**:**14g-b** = 50:50).

Rf (**14g-a**) = 0.24 (PE/EA = 5/1, v/v), (39.0 mg, 39% yield, pale yellow oil). NMR Spectroscopy: ^{1}H NMR (400 MHz, $CDCl_3$) δ 7.37–7.26 (m, 5H), 7.23–7.16 (m, 2H), 7.08 (td, J = 7.5, 1.1 Hz, 1H), 6.78 (dt, J = 7.8, 0.9 Hz, 1H), 5.86–5.56 (m, 1H), 5.00 – 4.87 (m, 2H), 4.79 (s, 1H), 4.26–4.17 (m, 1H), 2.22–1.82 (m, 2H), 1.55 (s, 3H); ^{13}C NMR (100 MHz, $CDCl_3$) δ 182.4, 141.4, 135.5, 134.8, 129.1, 128.4, 128.0, 127.3, 123.6, 122.7, 116.2 (t, J=236.9 Hz), 109.9, 68.9 (t, J=24.2 Hz), 46.7, 44.1, 36.1, 22.7; ^{19}F NMR (375 MHz, $CDCl_3$) δ –126.7– –132.9 (m, 2F). IR (ATR): 3358, 2967, 1681, 1491, 1383, 1182, 1055, 943, 805, 753 cm^{-1}. HRMS (ESI, m/z): calcd for $C_{19}H_{20}F_2NO_2^+$ $(M+H)^+$: 332.1457; Found: 332.1456.

Rf (**14g-b**) = 0.17 (PE/EA = 5/1, v/v). (38.6 mg, 39% yield, white solid, mp: 105.3 °C–106.7 °C). NMR Spectroscopy: ^{1}H NMR (400 MHz, $CDCl_3$) δ 7.35–7.25 (m, 5H), 7.19–7.15 (m, 2H), 7.08–7.02 (m, 1H), 6.80–6.69 (m, 1H), 5.55 (td, J = 56.2, 3.9 Hz, 1H), 5.01–4.83 (m, 2H), 3.48–3.33 (m, 1H), 2.43–2.13 (m, 2H), 1.46 (s, 3H); ^{13}C NMR (100 MHz, $CDCl_3$) δ 181.3, 142.7, 136.1, 132.5, 128.9, 128.3, 127.7, 127.5, 122.8, 122.8, 115.9 (t, J = 242.3 Hz), 109.7, 68.9 (t, J = 24.0 Hz), 46.2, 44.1, 37.0, 26.1; ^{19}F NMR (375 MHz, $CDCl_3$) δ –126.7– –132.9 (m, 2F). IR (ATR): 3418, 2926, 1700, 1491, 1383, 1305, 1182, 1059, 932, 752 cm^{-1}. HRMS (ESI, m/z): calcd for $C_{19}H_{20}F_2NO_2^+$ $(M+H)^+$: 332.1457; Found: 332.1456.

Methyl 3-(3,3-difluoro-2-hydroxypropyl)-3-methyl-2-oxo-1-phenylindoline-6-carboxylate (14h)

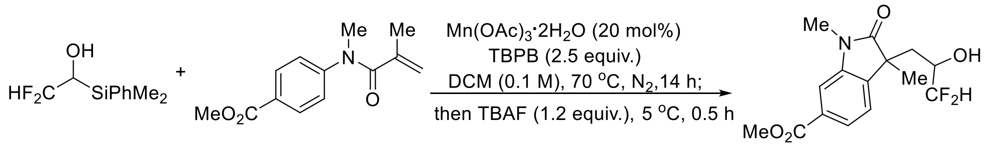

2a **10i** **14h**

Under N_2 atmosphere, to a dried 10 mL Schlenk tube equipped with a magnetic stir bar containing $Mn(OAc)_3 \cdot 2H_2O$ (16.1 mg, 0.06 mmol, 20 mol%) and **10i** (83.4 mg, 0.36 mmol, 1.2 equiv.) was added DCM (3 mL, 0.1 M), **2a** (64.8 mg, 0.3 mmol) and TBPB (145.7 mg, 0.75 mmol, 2.5 equiv.) sequentially. The tube was then sealed, and the resulting mixture was stirred at 70 °C in a heating block for 14 h, after which the mixture was cooled to 5 °C with ice bath. TBAF

(1.0 M in THF, 0.36 mL, 0.36 mmol, 1.2 equiv.) was added and the mixture was stirred at 5 °C for 0.5 h. The reaction mixture was quenched with water (2 mL) and extracted with DCM (3×10 mL). The organic phase was combined and washed with saturated Na_2CO_3 and brine, dried over Na_2SO_4, concentrated under reduced pressure. The crude product was purified with column chromatography on silica gel (200~300 mesh) and PE/EA (20/1~10/1, v/v) as eluent to afford 71.3 mg of the title compound **14h** (76% yield, **14h-a**:**14h-b** = 51:49)

R_f (**14h-a**) = 0.28 (PE/EA = 2/1, v/v). (36.8 mg, 39% yield, white solid, mp: 107.2–108.9 °C. NMR Spectroscopy: ^{1}H NMR (400 MHz, $CDCl_3$) δ 8.05 (dd, J = 8.1, 1.7 Hz, 1H), 7.88 (s, 1H), 6.92 (d, J = 8.2 Hz, 1H), 5.81–5.52 (m, 1H), 4.44 (s, 1H), 4.19–4.01(m, 1H), 3.91 (s, 3H), 3.28 (s, 3H), 2.18–1.80 (m, 2H), 1.49 (s, 3H); ^{13}C NMR (100 MHz, $CDCl_3$) δ 182.4, 166.8, 146.4, 134.8, 131.2, 125.5, 123.9, 116.0 (t, J = 240.6 Hz), 108.4, 68.6 (t, J = 24.1 Hz), 52.3, 46.5, 36.0, 26.9, 22.6; ^{19}F NMR (375 MHz, $CDCl_3$) δ –124.8– –133.7 (m, 2F). IR (ATR): 3414, 2926, 1703, 1498, 1457, 1286, 1103, 1051, 977, 772 cm^{-1}. HRMS (ESI, m/z): calcd for $C_{15}H_{18}F_2NO_4^+$ $(M+H)^+$: 314.1198; Found: 314.1198.

R_f (**14h-b**) = 0.17 (PE/EA = 2/1, v/v). (34.5 mg, 37% yield, white solid, mp: 128.4 °C–129.8 °C). NMR Spectroscopy: ^{1}H NMR (400 MHz, $CDCl_3$) δ 8.05 (dd, J = 8.1, 1.7 Hz, 1H), 6.92 (d, J = 8.2 Hz, 1H), 5.81–5.52 (m, 1H), 4.43 (s, 1H), 4.21–4.07 (m, 1H), 3.91 (s, 3H), 3.27 (s, 3H), 2.23–1.76 (m, 1H), 1.49 (s, 3H); ^{13}C NMR (100 MHz, $CDCl_3$) δ 181.6, 167.0, 147.8, 132.5, 131.2, 124.7, 124.0, 115.8 (t, J = 242.5 Hz), 108.1, 68.8 (t, J = 23.8 Hz), 52.2, 45.9, 37.2, 26.7, 25.2; ^{19}F NMR (375 MHz, $CDCl_3$) δ –126.5– –133.0 (m, 2F). IR (ATR): 3422, 2922, 1707, 1498, 1457, 1372, 1286, 1055, 977, 772 cm^{-1}. HRMS (ESI, m/z): calcd for $C_{15}H_{18}F_2NO_4^+$ $(M+H)^+$: 314.1198; Found: 314.1197.

3-(3,3-Difluoro-2-hydroxypropyl)-6-methoxy-1,3-dimethylindolin-2-one (14i)

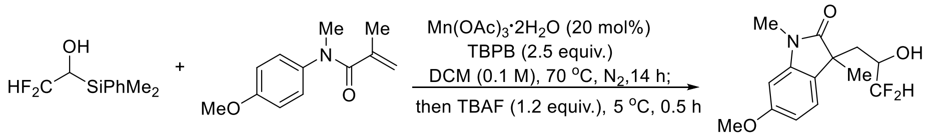

2a **10j** **14i**

Under N_2 atmosphere, to a dried 10 mL Schlenk tube equipped with a magnetic stir bar containing $Mn(OAc)_3$•$2H_2O$ (16.1 mg, 0.06 mmol, 20 mol%) and **10j** (73.8 mg, 0.36 mmol, 1.2 equiv.) was added DCM (3 mL, 0.1 M), **2a** (64.8 mg, 0.3 mmol) and TBPB (145.7 mg, 0.75 mmol, 2.5 equiv.) sequentially. The tube was then sealed, and the resulting mixture was stirred at 70 °C in a heating block for 14 h, after which the mixture was cooled to 5 °C with ice bath. TBAF (1.0 M in THF, 0.36 mL, 0.36 mmol, 1.2 equiv.) was added and the mixture was stirred at 5 °C for 0.5 h. The reaction mixture was quenched with water (2 mL) and extracted with DCM (3×10 mL). The organic phase was combined and washed with saturated Na_2CO_3 and brine, dried over Na_2SO_4, concentrated under reduced pressure. The crude product was purified with column chromatography on silica gel (200~300 mesh) and PE/EA (20/1~10/1, v/v) as eluent to afford 69.5 mg of the title compound **14i** (82% yield, **14i-a**:**14i-b** = 51:49)

R_f (**14i-a**) = 0.33 (PE/EA = 2/1, v/v). (35.5 mg, 42% yield, pale yellow oil). NMR Spectroscopy: ^{1}H NMR (400 MHz, $CDCl_3$) δ 6.86–6.73 (m, 3H), 5.86–5.51 (m, 1H), 5.12 (s, 1H), 4.25–4.09 (m, 1H), 3.80 (s, 3H), 3.22 (s, 3H), 2.10–1.76 (m, 2H), 1.46 (s, 3H).^{13}C NMR (100 MHz, $CDCl_3$) δ 181.9, 156.8, 136.2, 135.6, 116.2 (t, J = 239.6 Hz), 112.6, 110.1, 109.2, 68.5 (t, J = 24.1 Hz), 56.0, 47.1, 35.9, 26.7, 22.3; ^{19}F NMR (375 MHz, $CDCl_3$) δ –126.3– –133.4 (m, 2F). IR (ATR): 3384, 2920, 1674, 1498, 1435, 1383, 1286, 1047, 873, 741 cm^{-1}. HRMS (ESI, m/z): calcd for $C_{14}H_{18}F_2NO_3^+$ $(M+H)^+$: 286.1249; Found: 286.1249.

R_f (**14i-b**) = 0.19 (PE/EA = 2/1, v/v). (34.0 mg, 40% yield, white solid, mp: 134.5 °C–136.1 °C). NMR Spectroscopy: ^{1}H NMR (400 MHz, $CDCl_3$) δ 6.86–6.74 (m, 3H), 5.52 (td, J = 56.2, 3.8 Hz, 1H), 3.81 (s, 3H), 3.43–3.31 (m, 1H), 3.18 (s, 3H), 2.30–2.04 (m, 2H), 1.40 (s, 3H); ^{13}C NMR (100 MHz, $CDCl_3$) δ 180.9, 156.3, 137.1, 134.0, 115.9 (t, J = 242.2 Hz), 112.3, 110.5, 108.9, 68.9 (t, J = 23.8 Hz), 56.0, 46.6, 37.3, 26.6, 25.5; ^{19}F NMR (375 MHz, $CDCl_3$) δ –127.7– –132.3 (m, 2F). IR (ATR): 3392, 2930, 1685, 1498, 1290, 1238, 1126, 1036, 883, 701 cm^{-1}. HRMS (ESI, m/z): calcd for $C_{14}H_{18}F_2NO_3^+$ $(M+H)^+$: 286.1249; Found: 286.1249.

(E)-4-(2,6-Difluorophenyl)-1,1-difluorobut-3-en-2-ol (14j)

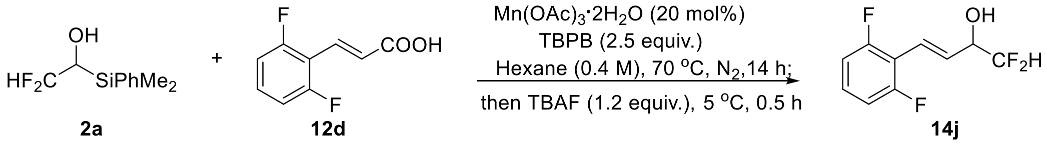

Under N_2 atmosphere, to a dried 10 mL Schlenk tube equipped with a magnetic stir bar containing $Mn(OAc)_3•2H_2O$ (16.1 mg, 0.06 mmol, 20 mol%) and **12d** (110.4 mg, 0.6 mmol, 2.0 equiv.) was added Hexane (0.75 mL, 0.4 M), **2a** (64.8 mg, 0.3 mmol) and TBPB (145.7 mg, 0.75 mmol, 2.5 equiv.) sequentially. The tube was sealed, and the resulting mixture was stirred at 70 °C in a heating block for 14 h, after which the mixture was cooled to 5 °C with ice bath. TBAF (1.0 M in THF, 0.36 mL, 0.36 mmol, 1.2 equiv.) was added and the mixture was stirred at 5 °C for 0.5 h. The reaction mixture was quenched with water (2 mL) and extracted with DCM (3×10 mL). The organic phase was combined and washed with saturated Na_2CO_3 and brine, dried over Na_2SO_4, concentrated under reduced pressure. The crude product was purified with column chromatography on silica gel (200~300 mesh) and PE/EA (20/1~10/1, v/v) as eluent to afford 39.6 mg of the title compound as a pale yellow oil (60% yield).

R_f = 0.40 (PE/EA = 5/1, v/v). NMR Spectroscopy: ^{1}H NMR (400 MHz, $CDCl_3$) δ 7.24–7.16 (m, 1H), 6.94–6.84 (m, 3H), 6.59–6.53(m, 1H), 5.89–5.59 (m, 1H), 4.48 (dq, J = 10.3, 5.2 Hz, 1H), 2.23 (s, 1H); ^{13}C NMR (150 MHz, $CDCl_3$) δ 161.2 (dd, J = 250.3, 7.3 Hz), 129.7–129.4 (m), 129.2 (t, J = 10.8 Hz), 121.2, 115.5 (t, J = 243.8 Hz), 113.4 (t, J = 15.1 Hz), 111.7 (dd, J = 21.5, 4.8 Hz), 72.8 (t, J = 24.4 Hz); ^{19}F NMR (375 MHz, $CDCl_3$) δ –112.7 (s, 2F), –126.3– –130.0 (m, 2F). IR (ATR): 3396, 2926, 1621, 1584, 1464, 1267, 1118, 1062, 999, 909 cm^{-1}. HRMS (ESI, m/z): calcd for $C_{10}H_8F_4ONa^+$ $(M+Na)^+$: 243.0404; Found: 243.0412.

5,5-Difluoro-1-(4-fluorophenyl)-4-hydroxy-2-methylenepentan-1-one (14k)

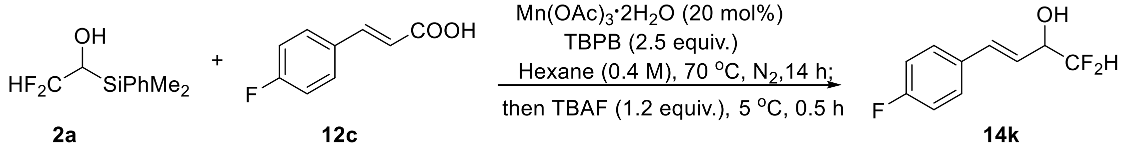

Under N_2 atmosphere, to a dried 10 mL Schlenk tube equipped with a magnetic stir bar containing $Mn(OAc)_3•2H_2O$ (16.1 mg, 0.06 mmol, 20 mol%) and **12c** (99.6 mg, 0.6 mmol, 2.0 equiv.) was added Hexane (0.75 mL, 0.4 M), **2a** (64.8 mg, 0.3 mmol) and TBPB (145.7 mg, 0.75 mmol, 2.5 equiv.) sequentially. The tube was sealed, and the resulting mixture was stirred at 70 °C in a heating block for 14 h, after which the mixture was cooled to 5 °C with ice bath. TBAF (1.0 M in THF, 0.36 mL, 0.36 mmol, 1.2 equiv.) was added and the mixture was stirred at 5 °C for 0.5 h. The reaction mixture was quenched with water (2 mL) and extracted with DCM (3×10 mL). The organic phase was combined and washed with saturated Na_2CO_3 and brine, dried over Na_2SO_4, concentrated under reduced pressure. The crude product was purified with column chromatography on silica gel (200~300 mesh) and PE/EA (20/1~10/1, v/v) as eluent to afford 35.4 mg of the title compound as a colorless oil (58% yield).

R_f = 0.32 (PE/EA = 5/1, v/v). NMR Spectroscopy: ^{1}H NMR (400 MHz, $CDCl_3$) δ 7.42–7.35 (m, 2H), 7.08–6.98(m, 2H), 6.78 (dd, J = 16.0, 1.4 Hz, 1H), 6.16–6.10 (m, 1H), 5.88–5.56 (m, 1H), 4.49–4.20 (m, 1H), 2.29 (s, 1H); ^{13}C NMR (100 MHz, $CDCl_3$) δ 162.9 (d, J = 245.5 Hz), 133.8, 132.1 (d, J = 3.2 Hz), 128.5 (d, J = 8.0 Hz), 122.4, 115.8 (d, J = 21.5 Hz), 115.6 (t, J = 242.3 Hz), 72.3 (t, J = 24.4 Hz); ^{19}F NMR (375 MHz, $CDCl_3$) δ –113.0 (td, J = 9.9, 4.9 Hz, 1F), –126.5– –133.0 (m, 2F). IR (ATR): 3411, 2971, 1703, 1603, 1510, 1230, 1051, 969, 854, 746 cm^{-1}. HRMS (ESI, m/z): calcd for $C_{10}H_{10}F_3O^+$ $(M+H)^+$: 203.0678; Found: 203.0686.

(E)-1,1-Difluoro-4-(4-methoxyphenyl)but-3-en-2-ol (14l)

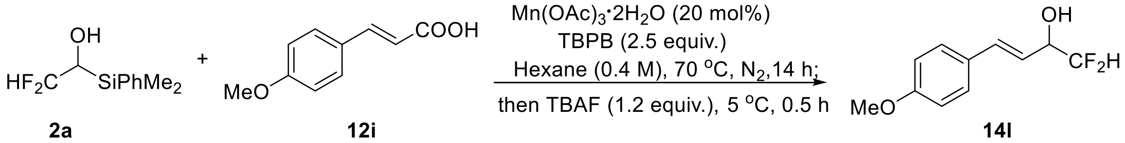

Under N_2 atmosphere, to a dried 10 mL Schlenk tube equipped with a magnetic stir bar containing $Mn(OAc)_3•2H_2O$ (16.1 mg, 0.06 mmol, 20 mol%) and **12i** (106.8 mg, 0.6 mmol, 2.0 equiv.) was added Hexane (0.75 mL, 0.4 M), **2a** (64.8

mg, 0.3 mmol) and TBPB (145.7 mg, 0.75 mmol, 2.5 equiv.) sequentially. The tube was then sealed, and the resulting mixture was stirred at 70 °C in a heating block for 14 h, after which the mixture was cooled to 5 °C with ice bath. TBAF (1.0 M in THF, 0.36 mL, 0.36 mmol, 1.2 equiv.) was added and the mixture was stirred at 5 °C for 0.5 h. The reaction mixture was quenched with water (2 mL) and extracted with DCM (3×10 mL). The organic phase was combined and washed with saturated Na_2CO_3 and brine, dried over Na_2SO_4, concentrated under reduced pressure. The crude product was purified with column chromatography on silica gel (200~300 mesh) and PE/EA (20/1~10/1, v/v) as eluent to afford 34.7 mg of the title compound as a pale yellow solid (54% yield).

R_f = 0.31 (PE/EA = 5/1, v/v). mp: 51.3°C–52.8 °C. NMR Spectroscopy: ^{1}H NMR (400 MHz, $CDCl_3$) *δ* 7.39–7.29 (m, 1H), 6.93–6.83 (m, 2H), 6.74 (dd, *J* = 15.9, 1.4 Hz, 1H), 6.08–6.02 (m, Hz, 1H), 5.71 (td, *J* = 56.1, 4.1 Hz, 1H), 4.49–4.37 (m, 1H), 2.29 (d, *J* = 4.7 Hz, 1H); ^{13}C NMR (100 MHz, $CDCl_3$) *δ* 160.0, 134.7, 128.7, 128.2, 120.3 (t, *J* = 4.1 Hz), 115.7 (t, *J* = 242.7 Hz), 114.2, 72.5 (t, *J* = 24.3 Hz), 55.4; ^{19}F NMR (375 MHz, $CDCl_3$) *δ* –119.5– –135.0 (m, 2F). IR (ATR): 3273, 2956, 1603, 1510, 1469, 1297, 1254, 1144, 809, 701 cm^{-1}. HRMS (ESI, m/z): calcd for $C_{11}H_{13}F_2O_2^+$ $(M+H)^+$: 215.0878; Found: 215.0870.

Application of radical C-Si bond activation in the synthesis of antitumor agent Z and its difluoro analog Z'.

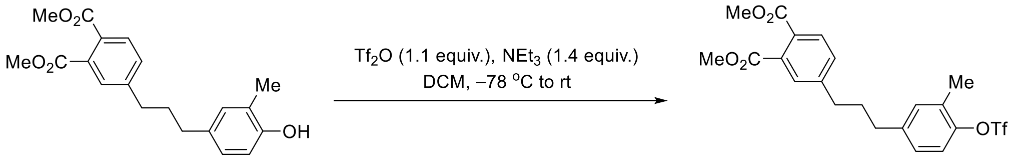

15 **16**

Under N_2 atmosphere, to a solution of **15** (2.3 g, 6.8 mmol) and NEt_3 (1.4 mL, 9.5 mmol, 1.4 equiv.) in DCM (35 mL) at −78 °C was added Tf_2O (1.3 mL, 7.5 mmol, 1.1 equiv.) slowly in 15 min, the reaction medium was brought to room temperature and stirred for another 2 h, upon completion, a sodium bicarbonate solution was added to quench the reaction, the resulting mixture was extracted with EA (50 mL×3 times), the combined organic phase was dried over Na_2SO_4, and concentrated under reduced pressure, the residue was purified by chromatography on a silica column with PE/ EA (4/1, v/v) as an eluent. A colorless oil **16** is obtained (3.1 g, 6.6 mmol, 97% yield).

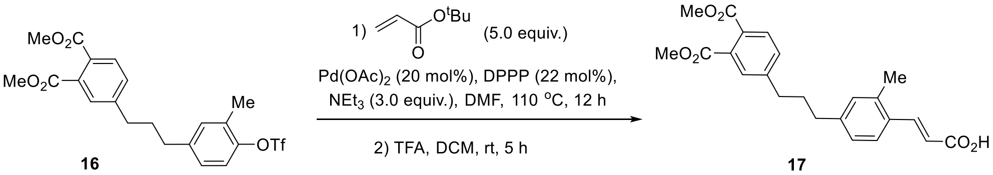

16 **17**

Under N_2 atmosphere, to a stirring solution of **16** (3.03 g, 6.4 mmol), $Pd(OAc)_2$ (144.0 mg, 0.64 mmol, 10 mol%), dppp (287.9 mg, 0.704 mmol, 11 mol%) and DMF (10 mL) was added NEt_3 (2.9 mL, 19.2 mmol, 3.0 equiv.) and tertbutyl acrylate (4.1 g, 32.0 mmol, 5.0 equiv.). The resulting mixture was stirred at 110 °C for 15 h, quenched by addition of water, extracted with EA (50 mL×3 times), the combined organic phase was dried over Na_2SO_4, and concentrated under reduced pressure, the residue was through a silica plug and used for next step without further purification. To the solution of tertbutyl substituted acrylate in DCM (6 mL) was added TFA (30 mL) slowly, the resulting mixture was open to air and stirred for 12 h. The solvent and excess TFA were removed under reduced pressure, the residue was extracted with EA, the combined organic phase was dried over Na_2SO_4, and concentrated under reduced pressure, the residue was purified by chromatography on a silica column with DCM/ MeOH (10/1, v/v) as an eluent. A pale solid **17** is obtained (1.9 g, 76% yield for two steps).

R_f = 0.5 (DCM/MeOH = 10/1, v/v). mp: 80 °C–82 °C. ^{1}H NMR (600 MHz, DMSO-*d*$_6$ (treated with anhydrous Na_2SO_4)) δ 7.78 (d, *J* = 15.8 Hz, 1H), 7.66 (d, *J* = 7.8 Hz, 1H), 7.58 (d, *J* = 7.8 Hz, 1H), 7.53–7.44 (m, 2H), 7.04 (d, *J* = 9.7 Hz, 2H), 6.36 (d, *J* = 15.9 Hz, 1H), 3.79 (s, 3H), 3.79 (s, 3H), 2.66 (t, *J* = 7.7 Hz, 2H), 2.54 (t, *J* = 6.9 Hz, 2H), 2.32 (s, 3H), 1.86 (p, *J* = 7.8 Hz, 2H); ^{13}C NMR (150 MHz, DMSO-*d*$_6$) δ 167.9, 167.9, 167.3, 146.5, 144.1, 141.3, 137.4, 132.3, 131.4, 130.9, 130.6, 129.2, 128.6, 128.5, 126.7, 126.6, 119.2, 52.7, 52.7, 34.6, 34.5, 31.9, 19.4. IR (ATR): 2997, 2941, 2855, 2699, 2594, 1715, 1685, 1607, 1498, 1424, 1282, 1197, 1126, 1073, 936, 824, 689 cm^{-1}. HRMS (ESI, m/z): calcd for $C_{23}H_{25}O_6^+$ $(M+H)^+$: 397.1646; Found: 397.1635.

(E)-dimethyl 4-(3-(3-methyl-4-(4,4,4-trifluoro-3-hydroxybut-1-en-1-yl)phenyl)propyl)phthalate (18)

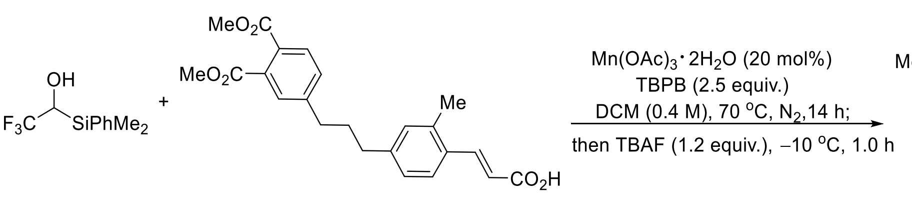

Under N_2 atmosphere, to a dried 10 mL Schlenk tube equipped with a magnetic stir bar containing $Mn(OAc)_3$•$2H_2O$ (5.3 mg, 0.02 mmol, 20 mol%) and **17** (79.2 mg, 0.2 mmol, 2.0 equiv.) was added DCM (0.5 mL, 0.2 M), TBPB (48.5 mg, 0.25 mmol, 2.5 equiv.) and **3a** (23.4 mg, 0.1 mmol) sequentially. The tube was sealed, and the resulting mixture was kept stirring at 70 °C in heating block for 14 h. The mixture was then cooled to −10 °C with low temperature bath, TBAF (1.0 M in THF, 0.12 mL, 0.12 mmol, 1.2 equiv.) was added and the resulting mixture was stirred at −10 °C for 1.0 h. The reaction mixture was quenched with water (2 mL), extracted with DCM (3×10 mL) and organic phase was combined and washed with brine, dried over Na_2SO_4, concentrated under reduced pressure. The crude product was purified with column chromatography on silica gel (200~300 mesh) and PE/EA (20/1~10/1, v/v) as eluent to afford 27.0 mg of the title compound as a colorless oil (71% yield). The reaction conducted on 0.4 mmol scale afford 123.0 mg of the title compound (68% yield).

R_f = 0.43 (PE/EA = 2/1, v/v). NMR Spectroscopy: ^{1}H NMR (400 MHz, $CDCl_3$) δ 7.68 (d, J = 7.9 Hz, 1H), 7.48 (s, 1H), 7.38 (d, J = 7.9 Hz, 1H), 7.33 (d, J = 7.6 Hz, 1H), 7.07–6.96 (m, 3H), 6.06 (dd, J = 15.7, 6.6 Hz, 1H), 4.65–4.62 (m, 1H), 3.90 (s, 3H), 3.89 (s, 3H), 2.69 (t, J = 7.5 Hz, 2H), 2.60 (t, J = 7.5 Hz, 2H), 2.34 (s, 3H), 1.99–1.91 (m, 2H); ^{13}C NMR (100 MHz, $CDCl_3$) δ 168.7, 168.0, 146.4, 142.4, 136.1, 134.1, 132.7, 132.5, 131.0, 130.7, 129.4, 129.0, 128.8, 126.5, 126.2, 124.5 (q, J = 280.6 Hz), 121.6, 71.9 (q, J = 32.1 Hz), 52.8, 52.7, 35.2, 35.1, 32.4, 19.8; ^{19}F NMR (375 MHz, $CDCl_3$) δ –79.0 (d, J = 8.9 Hz, 3F). IR (ATR): 3448, 3004, 2952, 2863, 1722, 1610, 1498, 1435, 1286, 1167, 1126, 969, 790, 738 cm^{-1}. HRMS (ESI, m/z): calcd for $C_{24}H_{26}F_3O_5^+$ $(M+H)^+$: 451.1727; Found: 451.1712.

(E)-dimethyl 4-(3-(3-methyl-4-(4,4 -difluoro-3-hydroxybut-1-en-1-yl)phenyl)propyl)phthalate (19)

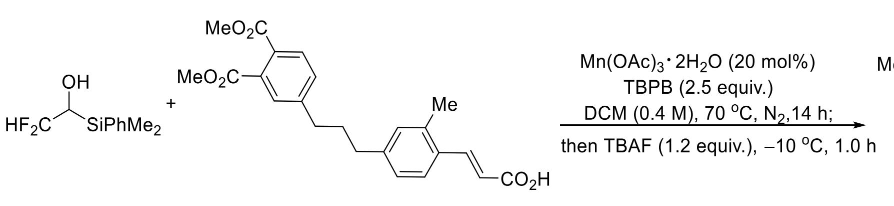

Under N_2 atmosphere, to a dried 10 mL Schlenk tube equipped with a magnetic stir bar containing $Mn(OAc)_3$•$2H_2O$ (10.6 mg, 0.04 mmol, 20 mol%) and **17** (158.4 mg, 0.4 mmol, 2.0 equiv.) was added DCM (1.0 mL, 0.2 M), TBPB (97.0 mg, 0.50 mmol, 2.5 equiv.) and **2a** (43.2 mg, 0.2 mmol) sequentially. The tube was sealed, and the resulting mixture was kept stirring at 70 °C in heating block for 14 h. The mixture was then cooled to −10 °C with low temperature bath, TBAF (1.0 M in THF, 0.24 mL, 0.24 mmol, 1.2 equiv.) was added and the resulting mixture was stirred at −10 °C for 1.0 h. The reaction mixture was quenched with water (2 mL), extracted with DCM (3×10 mL) and organic phase was combined and washed with brine, dried over Na_2SO_4, concentrated under reduced pressure. The crude product was purified with column chromatography on silica gel (200~300 mesh) and PE/EA (20/1~10/1, v/v) as eluent to afford 59.2 mg of the title compound as a colorless oil (69% yield).

R_f = 0.43 (PE/EA = 2/1, v/v). NMR Spectroscopy: ^{1}H NMR (400 MHz, $CDCl_3$) δ 7.68 (d, J = 7.6 Hz, 1H), 7.48 (s, 1H), 7.39–7.32 (m, 2H), 7.01–6.96 (m, 3H), 6.07 (dd, J = 15.7, 6.3 Hz, 1H), 5.72 (td, J = 56.1, 3.9 Hz, 1H), 4.47–4.45 (m, 1H), 3.90 (s, 3H), 3.89 (s, 3H), 2.69 (t, J = 7.5 Hz, 2H), 2.60 (t, J = 7.3 Hz, 2H), 2.33 (s, 3H), 1.99–1.91 (m, 2H); ^{13}C NMR (150 MHz, $CDCl_3$) δ 168.7, 168.0, 146.4, 142.0, 136.0, 132.8, 132.7, 132.6, 131.0, 130.7, 129.4, 129.0, 128.8, 126.4, 126.0, 123.4 (t, J = 4.0 Hz), 115.7 (t, J = 243.6 Hz), 72.5 (t, J = 24.2 Hz), 52.8, 52.7, 35.2, 35.0, 32.4, 19.8; ^{19}F NMR (375 MHz, $CDCl_3$) δ –125.9– –131.7 (m, 2F). IR (ATR): 3459, 2948, 1722, 1607, 1435, 1286, 1126, 1070, 969, 734 cm^{-1}. HRMS (ESI, m/z): calcd for $C_{24}H_{26}F_3O_5Na^+$ $(M+Na)^+$: 455.1641; Found: 455.1625.

(E)-(4-(3-(3-methyl-4-(4,4,4-trifluoro-3-hydroxybut-1-en-1-yl)phenyl)propyl)-1,2-phenylene)dimethanol (antitumor agent Z)

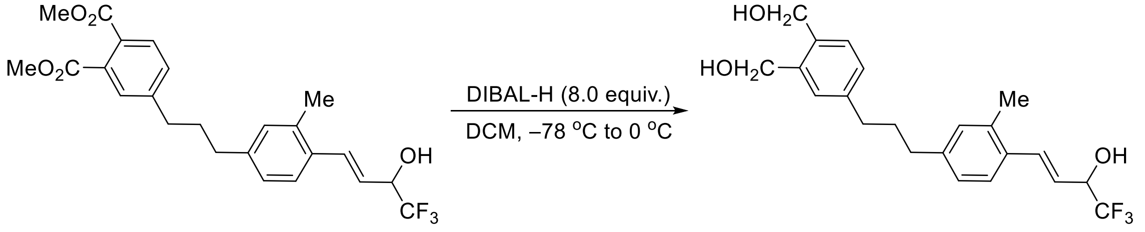

18 **antitumor agent Z**

Under N_2 atmosphere, to a dried 10 mL round bottom flask equipped with a magnetic stir bar containing **18** (58.5 mg, 0.13 mmol) and DCM (1.5 mL) was added DIBAL-H (1.0 mL, 1.0 M in hexane, 1.0 mmol, 8.0 equiv.) slowly in 10 min, the reaction medium was brought to 0 °C gradually. The reaction was quenched with 1.0 M HCl, then the resulting mixture was extracted with EA, and organic phase was combined and washed with brine, dried over Na_2SO_4, concentrated under reduced pressure. The crude product was purified with column chromatography on silica gel (200~300 mesh) and PE/EA (1/2, v/v) as eluent to afford 48.1 mg of the title compound as a white solid (94% yield).

R_f = 0.22 (PE/EA = 1/1, v/v). mp: 79 °C–81 °C. NMR Spectroscopy: ^{1}H NMR (400 MHz, $CDCl_3$) δ 7.57 (d, J = 7.6 Hz, 1H), 7.45 (d, J = 9.2 Hz, 1H), 7.36–7.32 (m, 2H), 7.22–7.18 (m, 3H), 6.25 (dd, J = 15.9, 6.7 Hz, 1H), 4.90 (s, 4H), 4.82 (s, 1H), 3.28 (s, 2H), 3.10 (s, 1H), 2.86–2.79 (m, 4H), 2.53 (s, 3H), 2.17–2.10 (m, 2H); ^{13}C NMR (150 MHz, $CDCl_3$) 143.0, 142.8, 139.5, 136.9, 136.1, 134.1, 132.3, 130.7, 130.2, 130.1, 128.6, 126.5, 126.1, 124.5 (q, J = 280.1 Hz), 121.5, 71.9 (q, J = 31.9 Hz), 64.5, 64.2, 35.2, 35.2, 32.8, 19.9; ^{19}F NMR (375 MHz, $CDCl_3$) –78.9 (d, J = 8.9 Hz, 3F). IR (ATR): 3340, 2922, 2855, 1610, 1454, 1267, 1170, 1126, 1010, 969, 831, 734 cm^{-1}. HRMS (ESI, m/z): calcd for $C_{24}H_{25}F_3O_3Na^+$ $(M+Na)^+$: 417.1648; Found: 455.1636.

(E)-(4-(3-(4-(4,4-difluoro-3-hydroxybut-1-en-1-yl)-3-methylphenyl)propyl)-1,2-phenylene)dimethanol (difluoro analog Z' of antitumor agent Z)

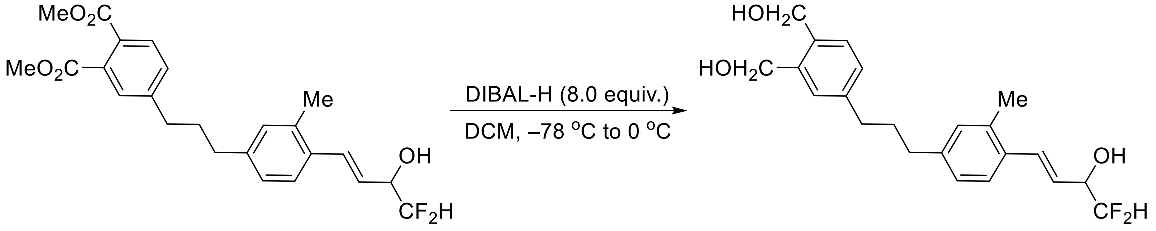

19 **difluoro analog Z' of antitumor agent Z**

Under N_2 atmosphere, to a dried 10 mL round bottom flask equipped with a magnetic stir bar containing **19** (43.2 mg, 0.1 mmol) and DCM (1.5 mL) was added DIBAL-H (0.8 mL, 1.0 M in hexane, 0.8 mmol, 8.0 equiv.) slowly in 10 min, the reaction medium was brought to 0 °C gradually. The reaction was quenched with 1.0 M HCl, then the resulting mixture was extracted with EA, and organic phase was combined and washed with brine, dried over Na_2SO_4, concentrated under reduced pressure. The crude product was purified with column chromatography on silica gel (200~300 mesh) and PE/EA (1/2, v/v) as eluent to afford 33.5 mg of the title compound as a white solid (89% yield).

R_f = 0.33 (PE/EA = 1/2, v/v). mp: 79 °C–80 °C. NMR Spectroscopy: ^{1}H NMR (600 MHz, $CDCl_3$) δ 7.38 (d, J = 7.8 Hz, 1H), 7.27–7.24 (m, 2H), 7.17 (s, 1H), 7.13 (d, J = 7.5 Hz, 1H), 7.03–6.93 (m, 3H), 6.06 (dd, J = 15.9, 6.4 Hz, 1H), 5.71 (td, J = 56.1, 4.2 Hz, 1H), 4.71 (s, 4H), 4.51–4.40 (m, 1H), 2.64 (t, 2H), 2.60 (t, J = 7.7 Hz, 2H), 2.45 (bs, 3H), 2.33 (s, 3H), 1.97–1.88 (m, 2H); ^{13}C NMR (150 MHz, $CDCl_3$) 143.0, 142.5, 139.6, 136.9, 135.9, 132.7, 132.7, 130.7, 130.1, 130.0, 128.6, 126.5, 126.0, 123.3 (t, J = 3.9 Hz), 115.7 (t, J = 243.7 Hz), 72.6 (t, J = 24.3 Hz), 64.6, 64.2, 35.2, 35.2, 32.8, 19.9; ^{19}F NMR (375 MHz, $CDCl_3$) –127.3– –129.6 (m, 2F). IR (ATR): 3355, 3213, 2922, 2855, 1614, 1461, 1372, 1129, 1066, 999, 831 cm^{-1}. HRMS (ESI, m/z): calcd for $C_{24}H_{26}F_2O_3Na^+$ $(M+Na)^+$: 399.1742; Found: 399.1730.

NMR spectra for new compounds

^{1}H NMR of **5** ($CDCl_3$, 400 MHz, 25 ºC)

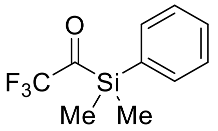

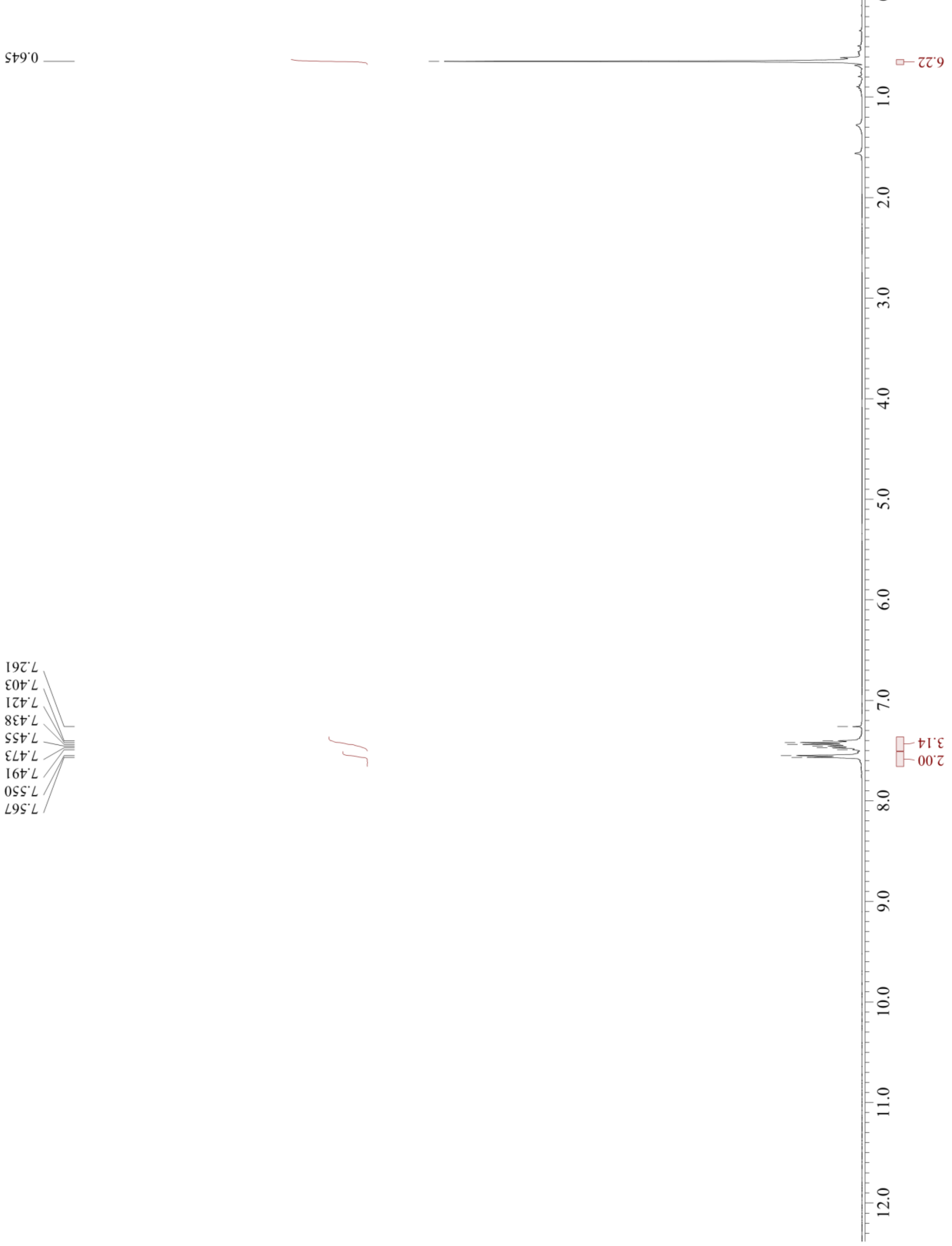

^{13}C NMR of **5** ($CDCl_3$, 100 MHz, 25 ºC)

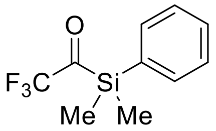

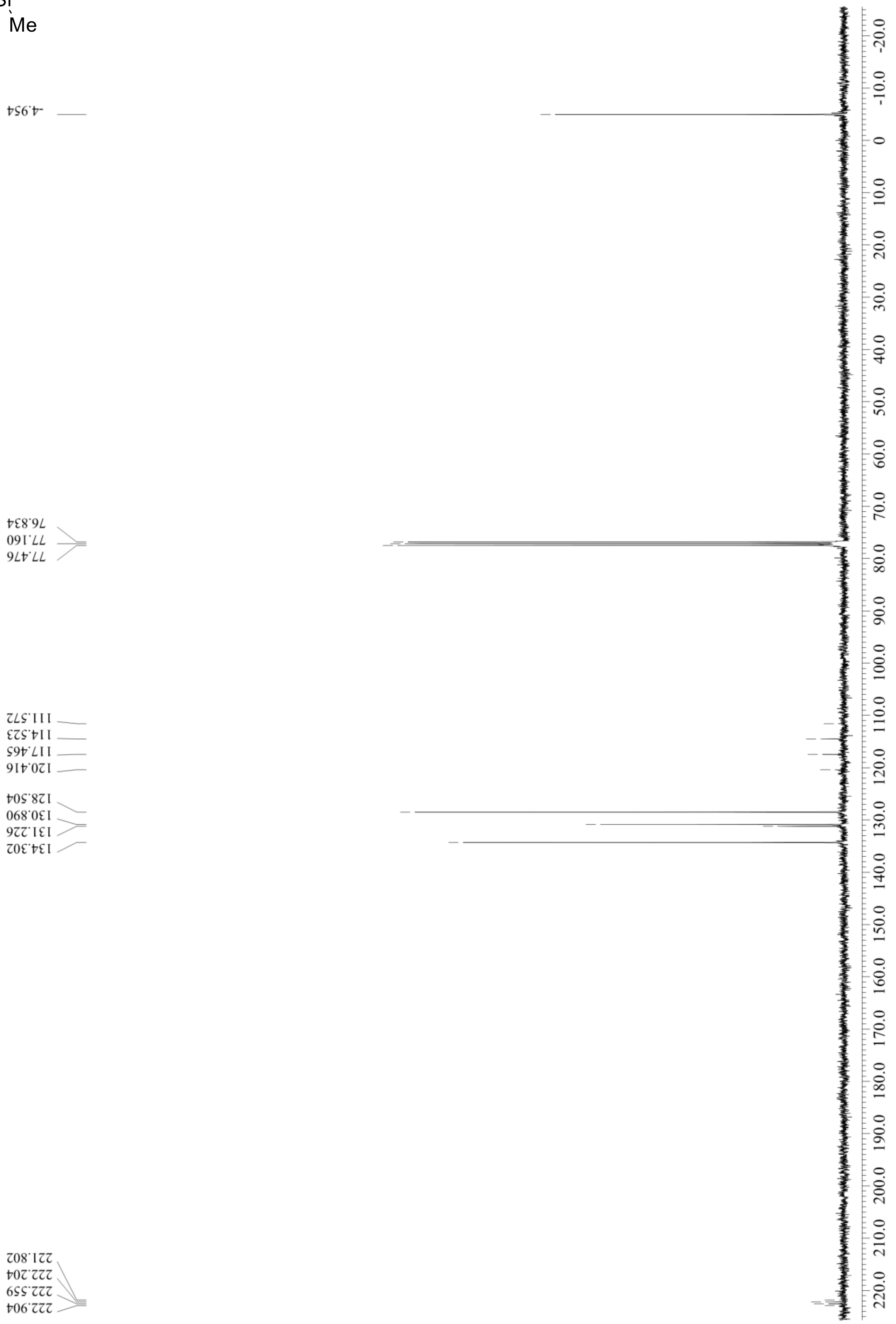

^{19}F NMR of **5** ($CDCl_3$, 375 MHz, 25 ºC)

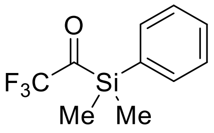

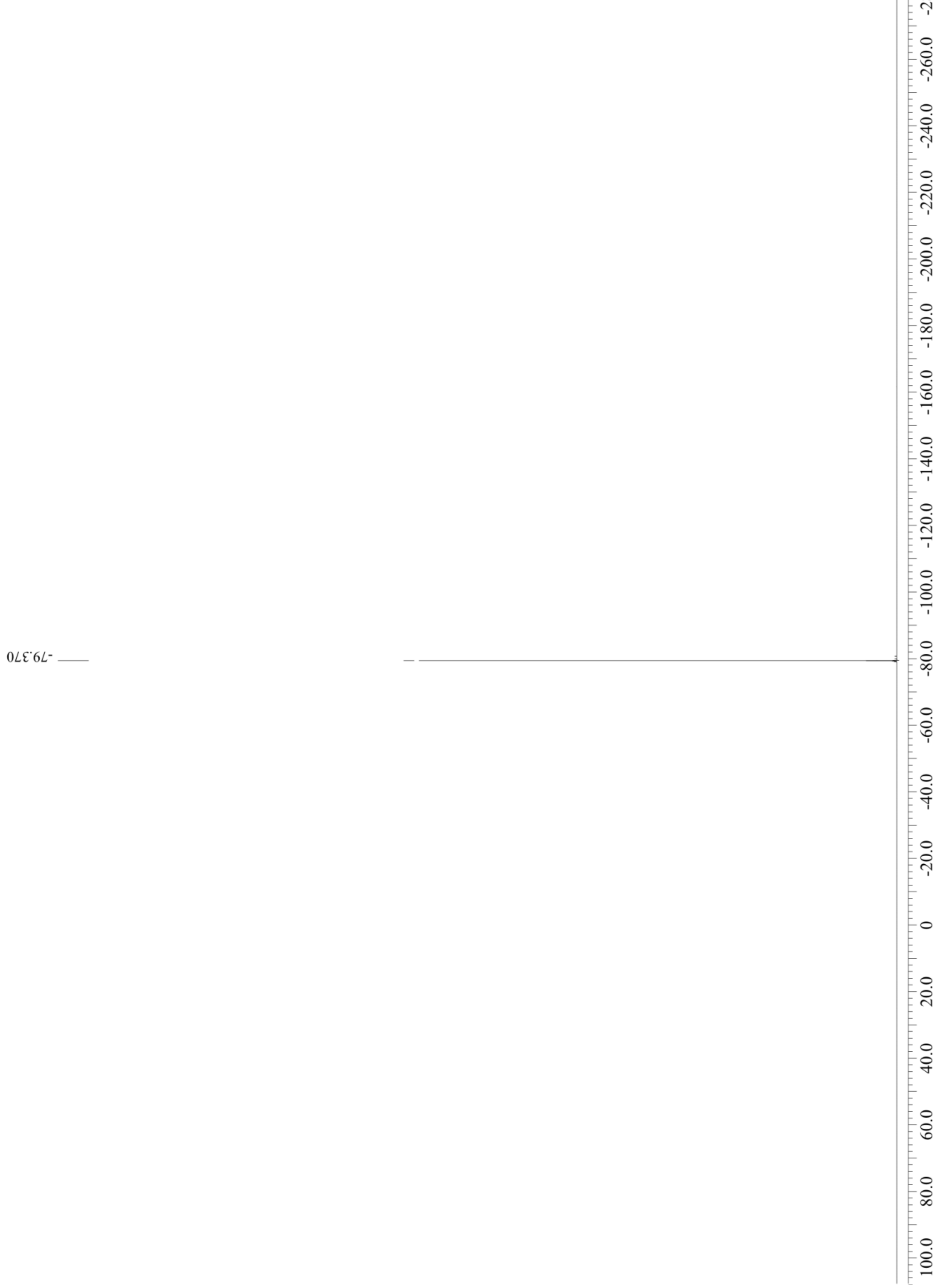

^{1}H NMR of **1a** ($CDCl_3$, 400 MHz, 25 ºC)

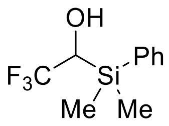

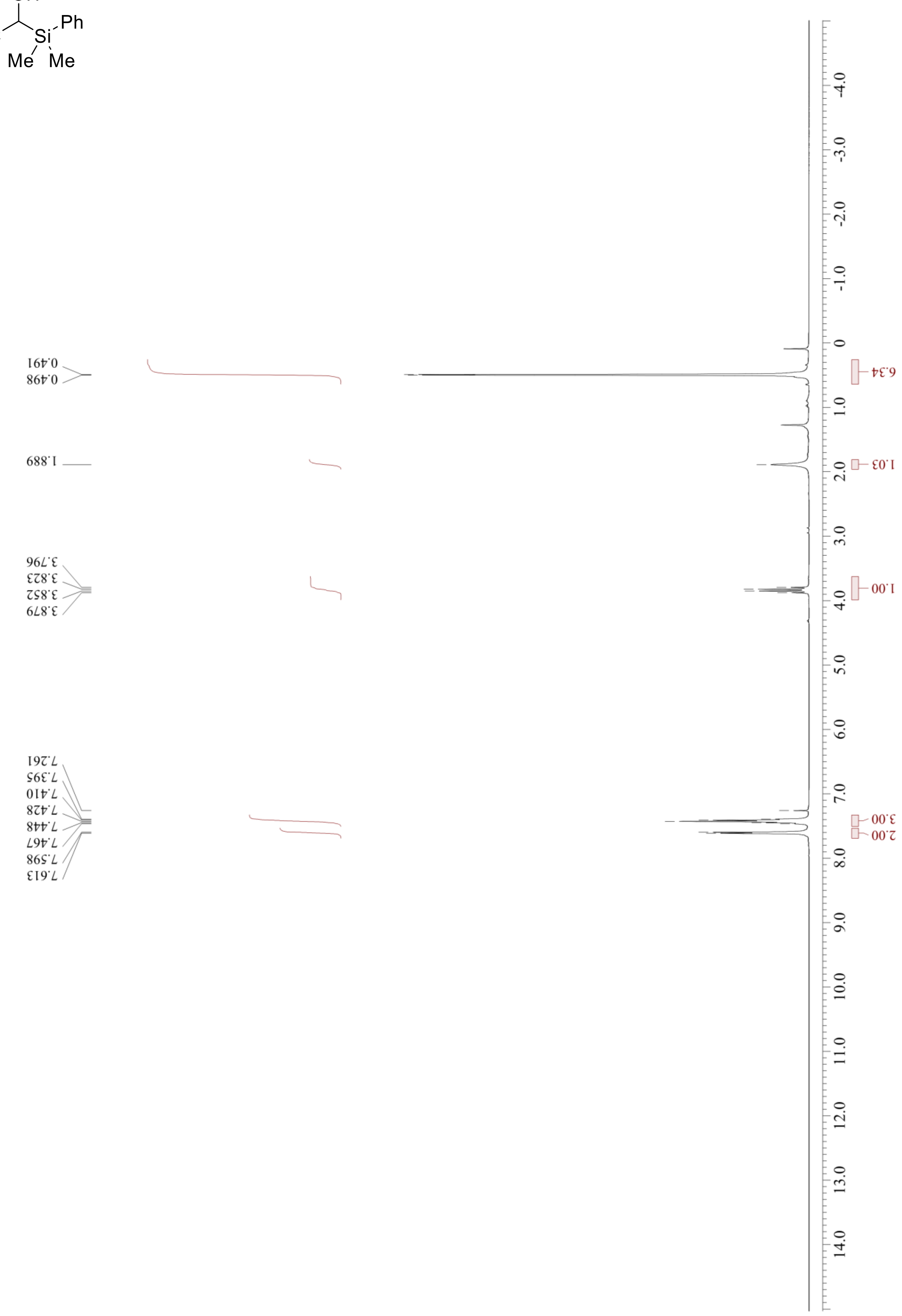

^{13}C NMR of **1a** ($CDCl_3$, 100 MHz, 25 ºC)

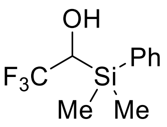

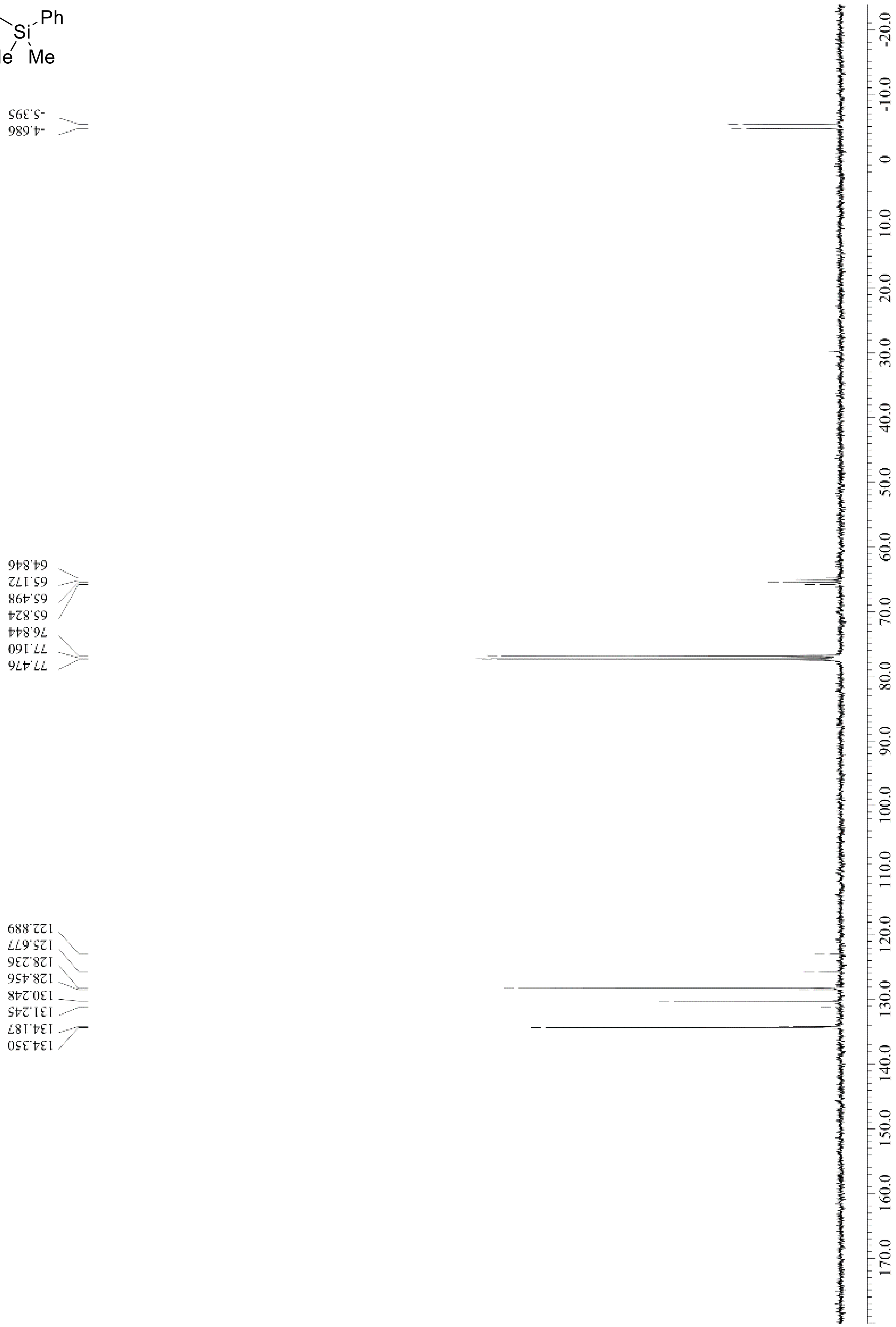

^{19}F NMR of **1a** ($CDCl_3$, 375 MHz, 25 ºC)

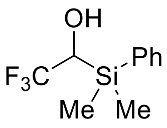

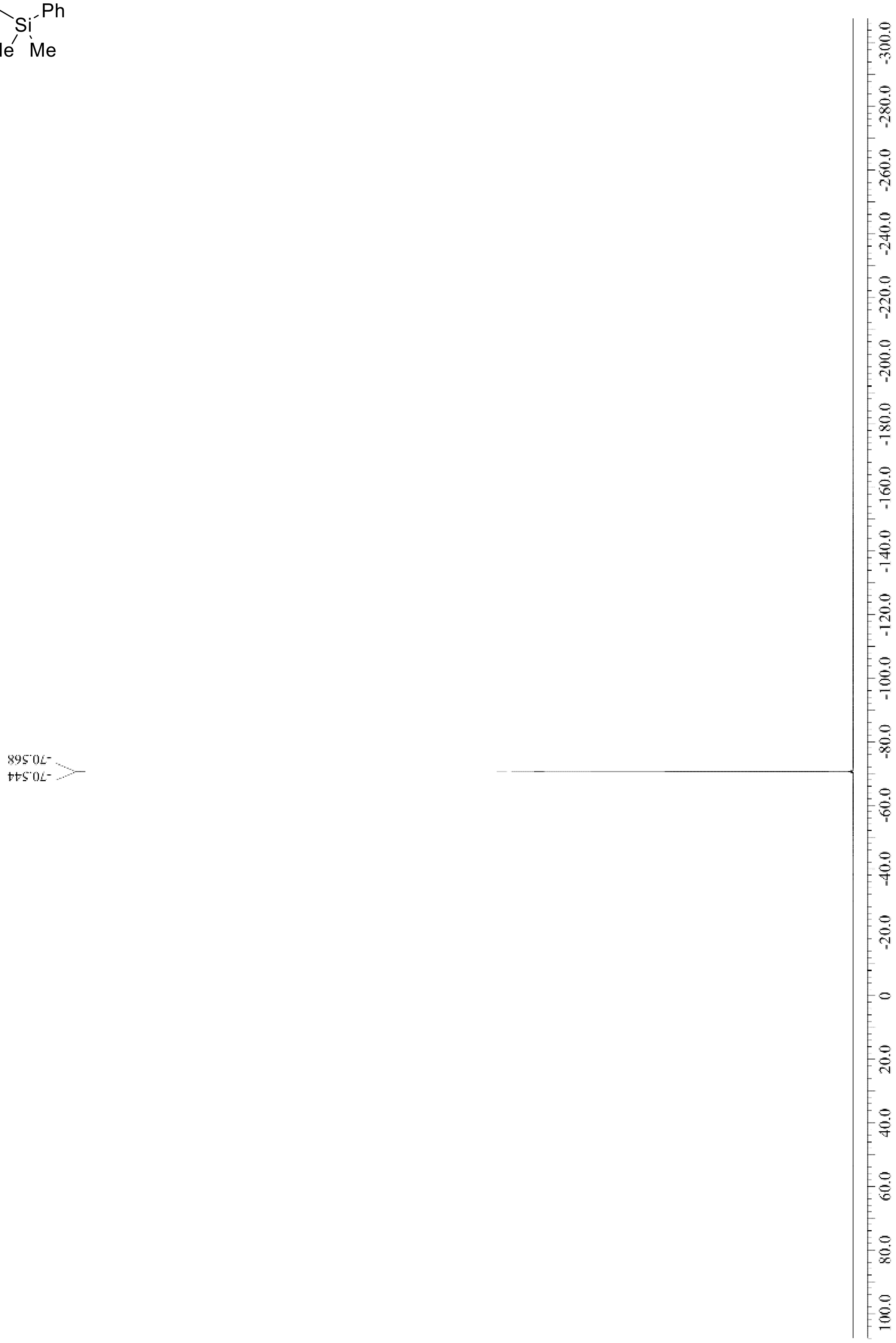

^{1}H NMR of **5b** ($CDCl_3$, 400 MHz, 25 ºC)

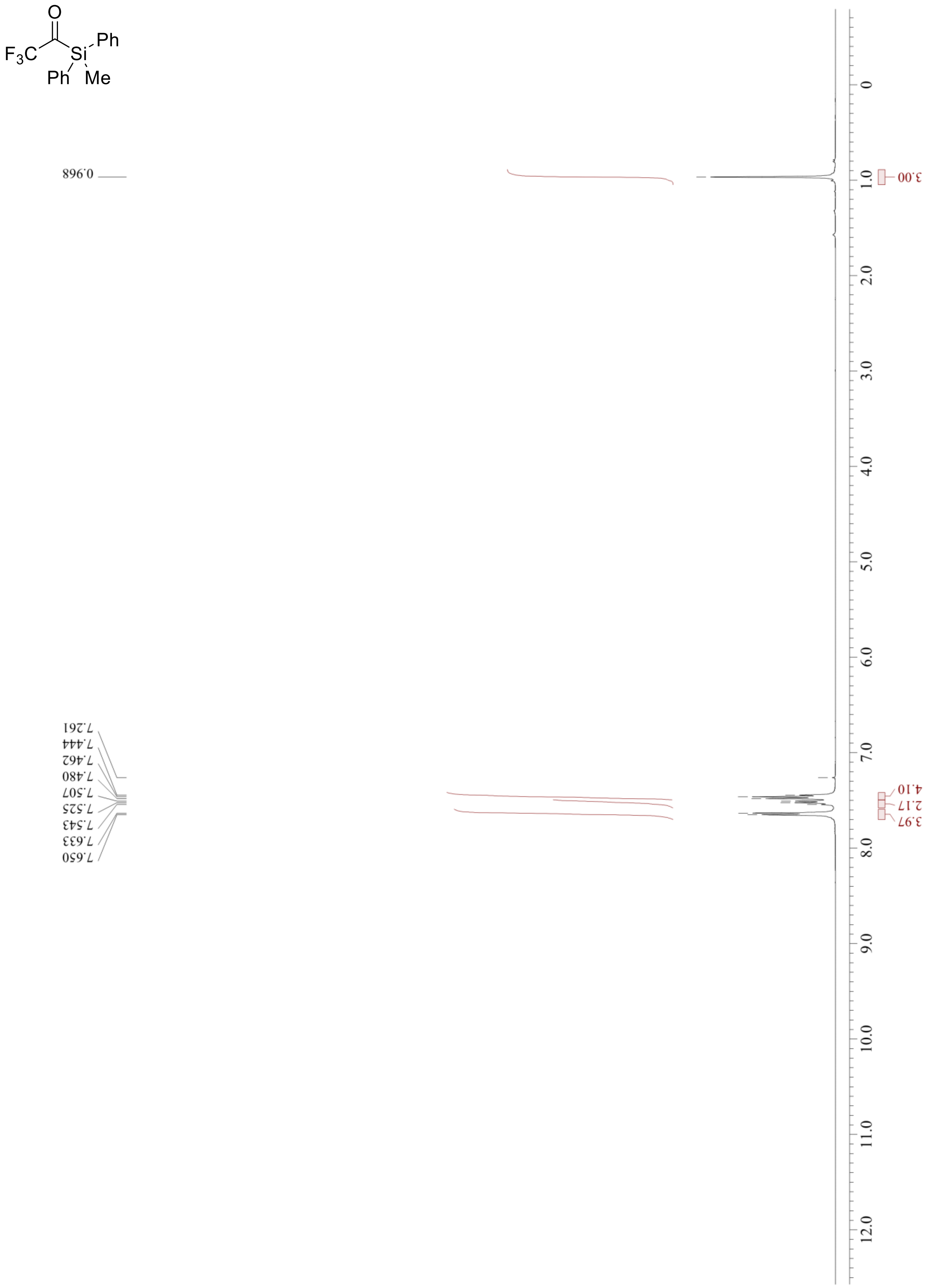

^{13}C NMR of **5b** ($CDCl_3$, 100 MHz, 25 ºC)

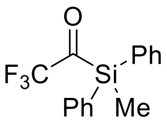

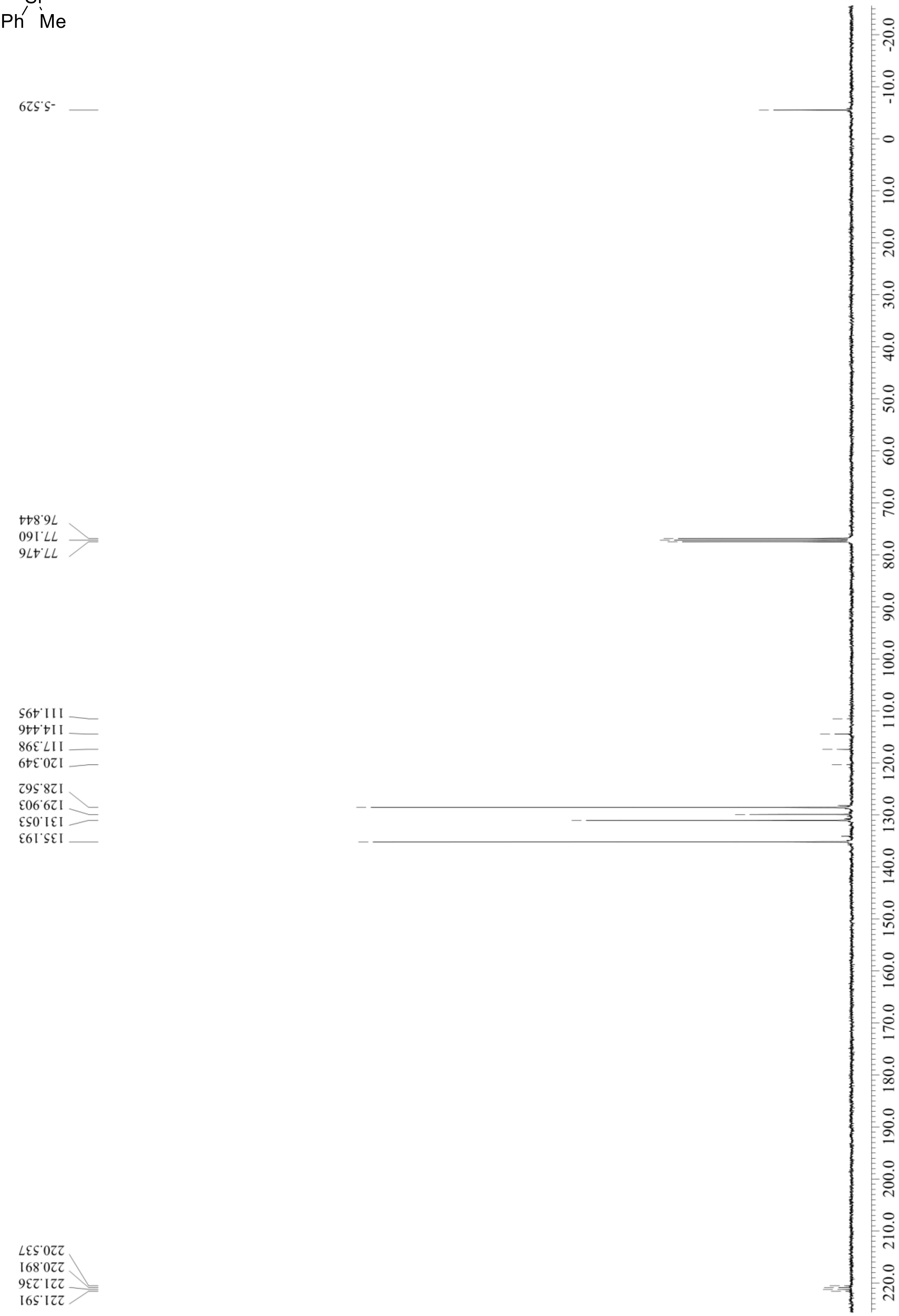

^{19}F NMR of **5b** ($CDCl_3$, 375 MHz, 25 ºC)

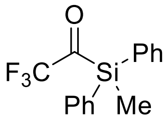

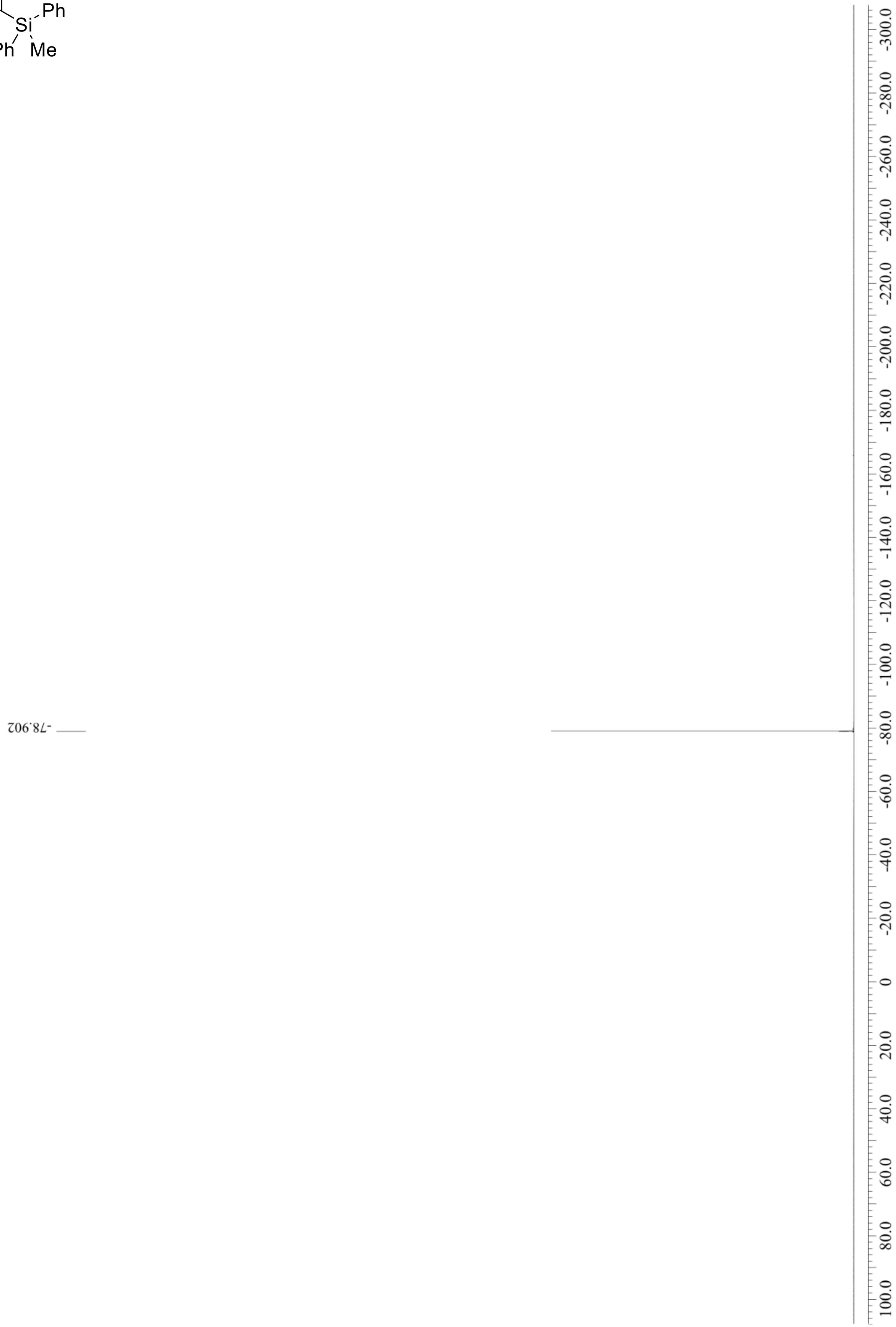

^{1}H NMR of **1b** ($CDCl_3$, 400 MHz, 25 ºC)

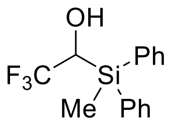

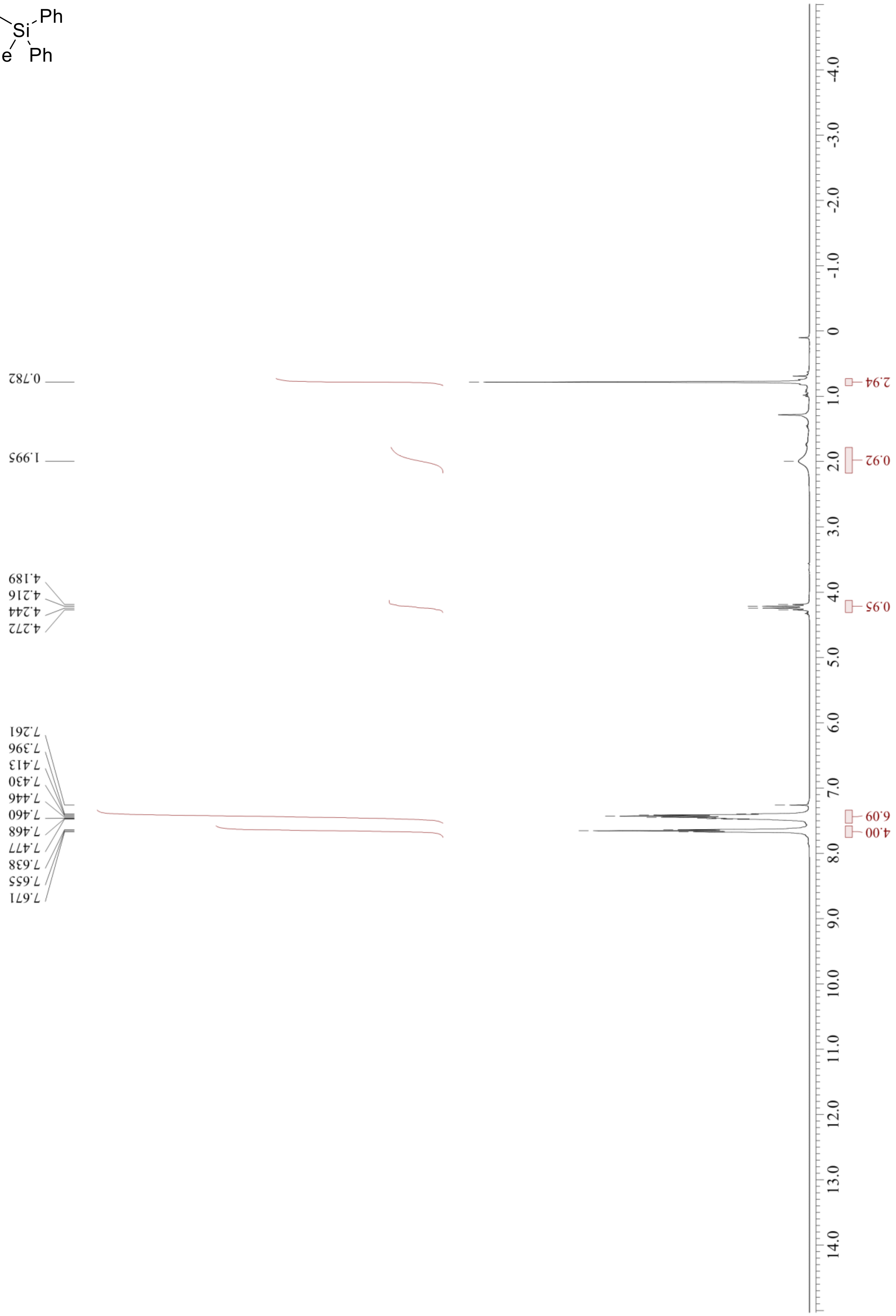

^{13}C NMR of **1b** ($CDCl_3$, 100 MHz, 25 ºC)

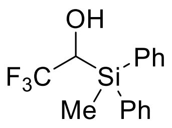

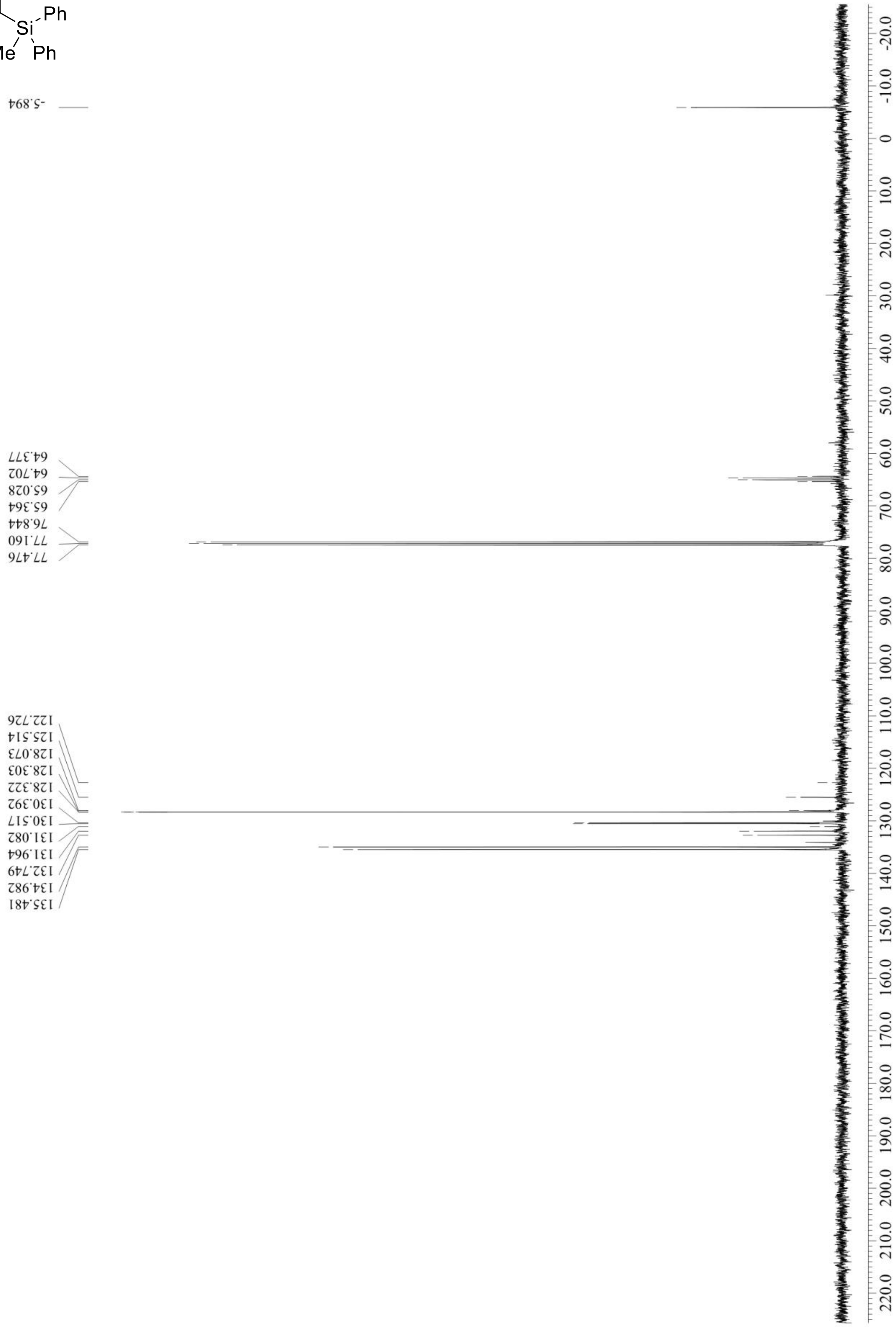

^{19}F NMR of **1b** ($CDCl_3$, 375 MHz, 25 ºC)

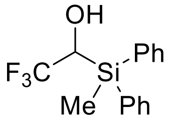

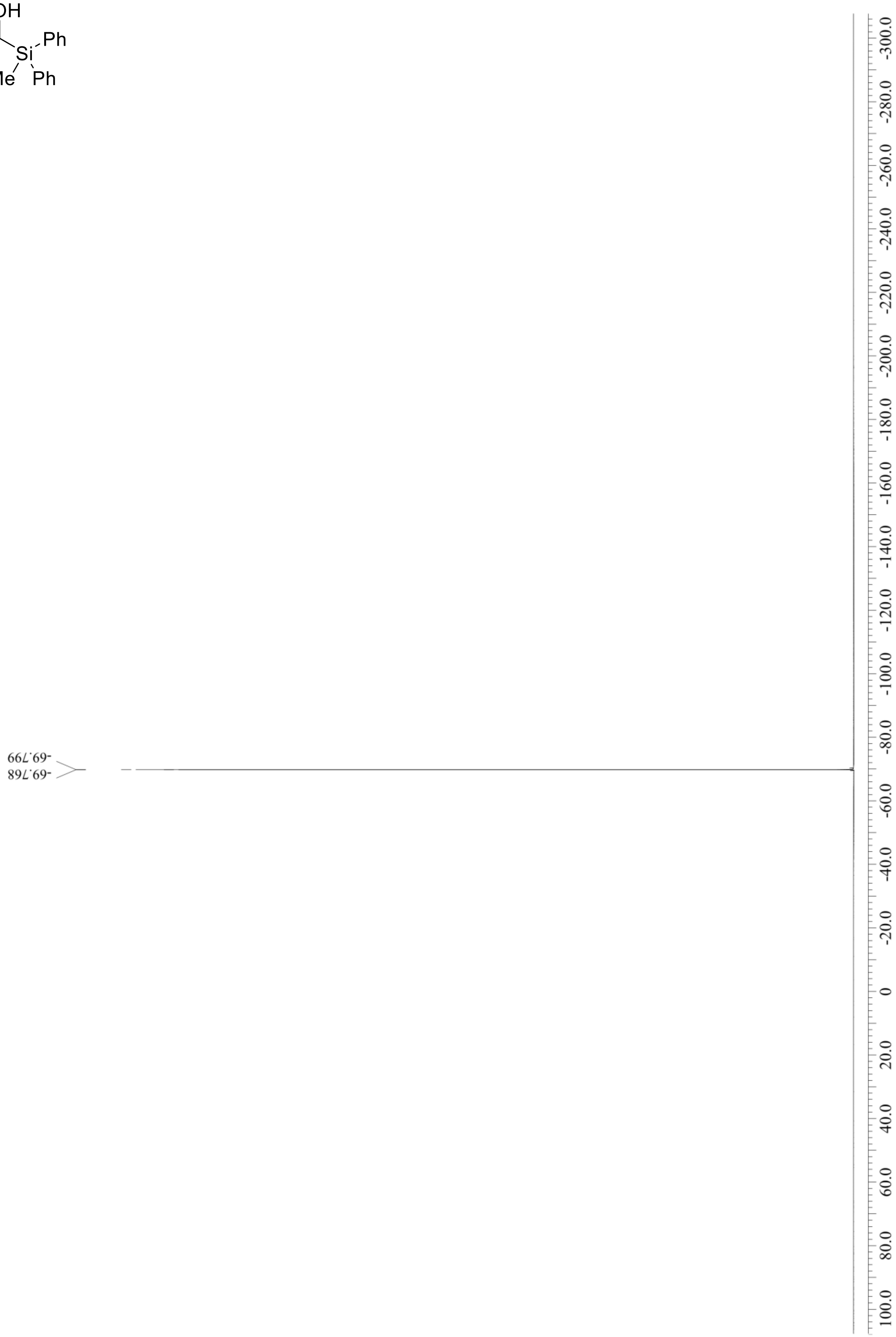

^{1}H NMR of **1c** ($CDCl_3$, 400 MHz, 25 ºC)

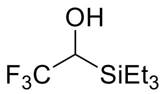

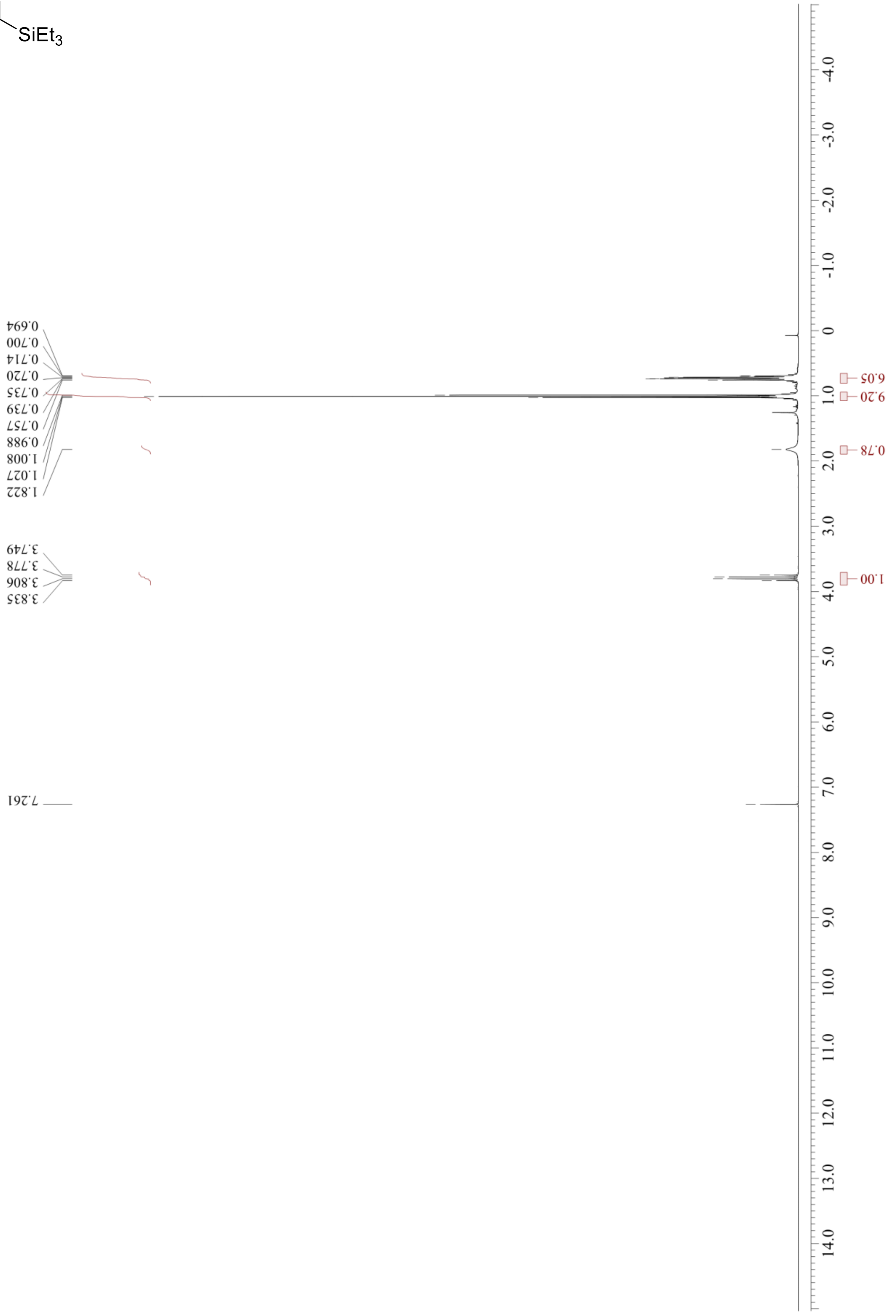

^{13}C NMR of **1c** ($CDCl_3$, 100 MHz, 25 ºC)

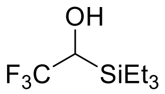

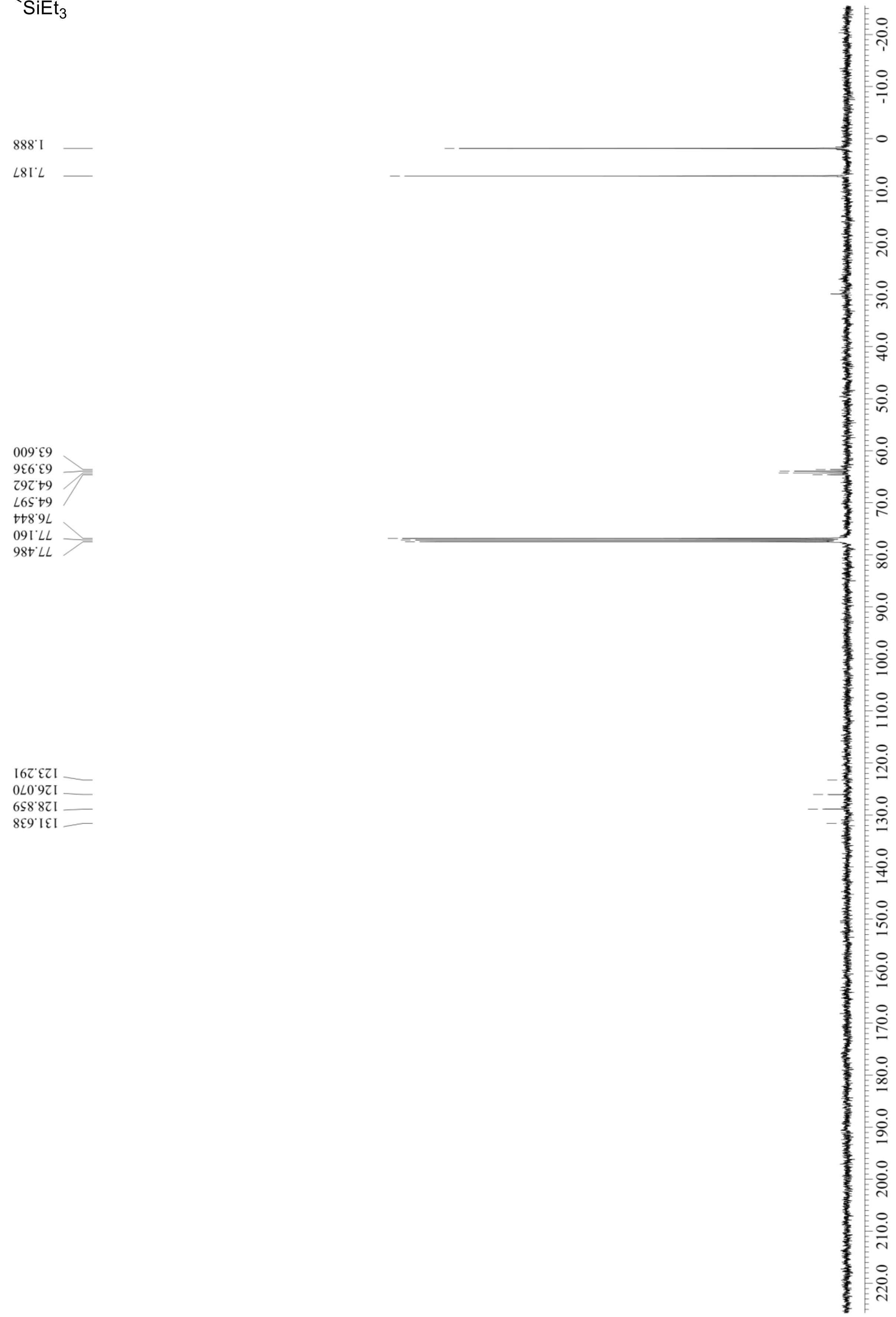

^{19}F NMR of **1c** ($CDCl_3$, 375 MHz, 25 ºC)

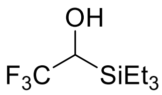

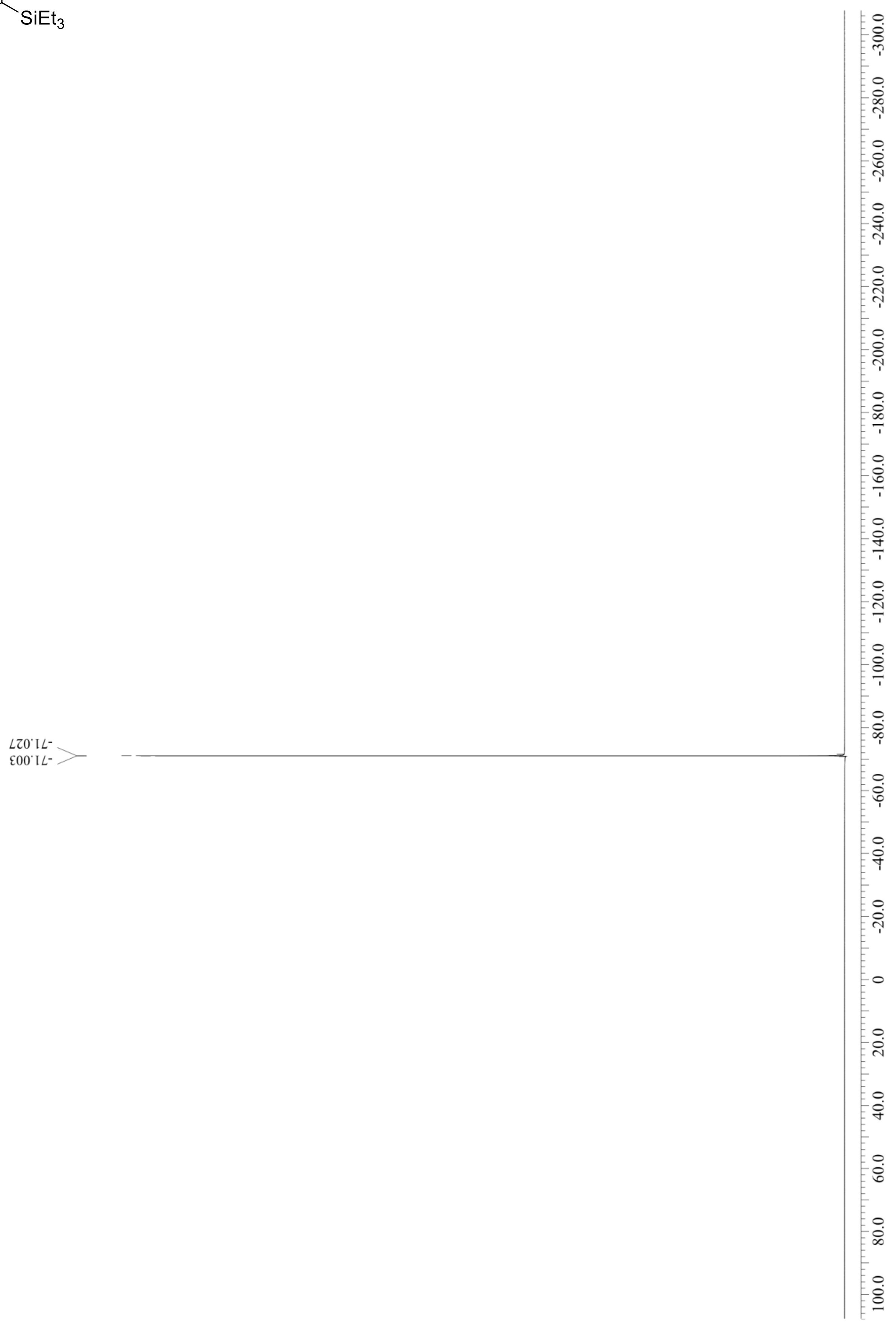

^{1}H NMR of **1d** ($CDCl_3$, 400 MHz, 25 °C)

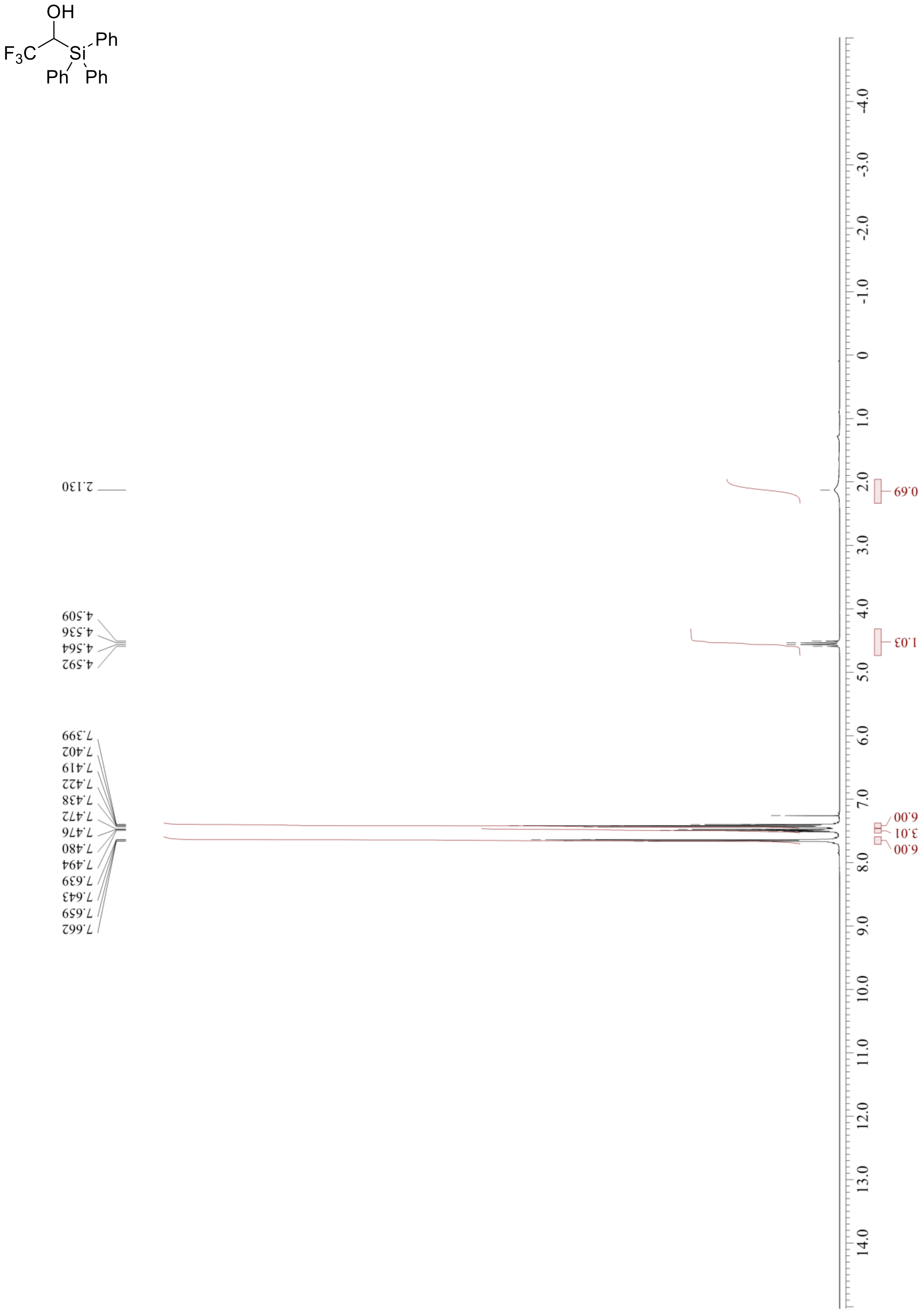

^{13}C NMR of **1d** ($CDCl_3$, 100 MHz, 25 ºC)

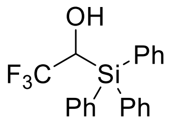

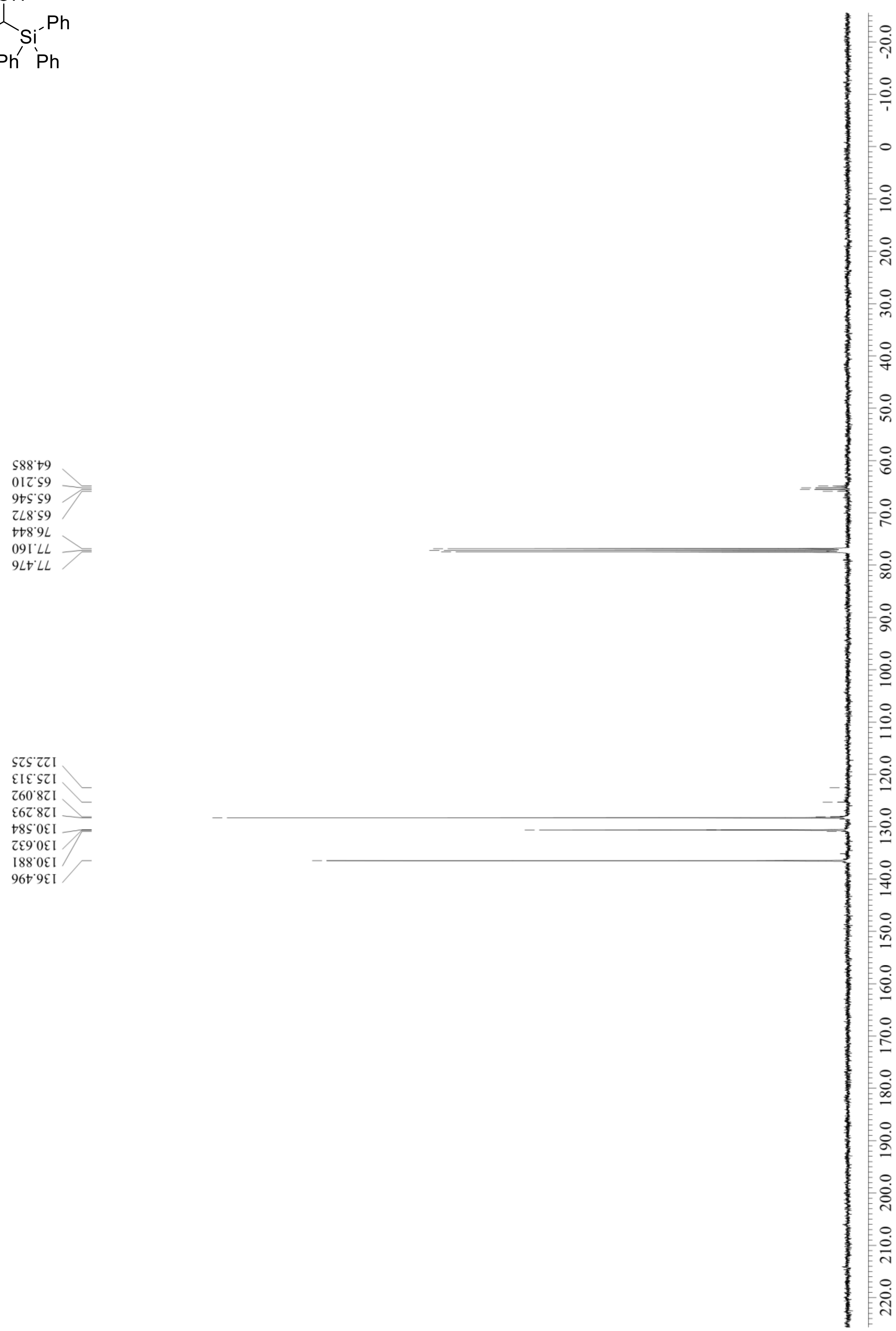

^{19}F NMR of **1d** ($CDCl_3$, 375 MHz, 25 ºC)

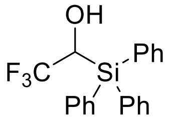

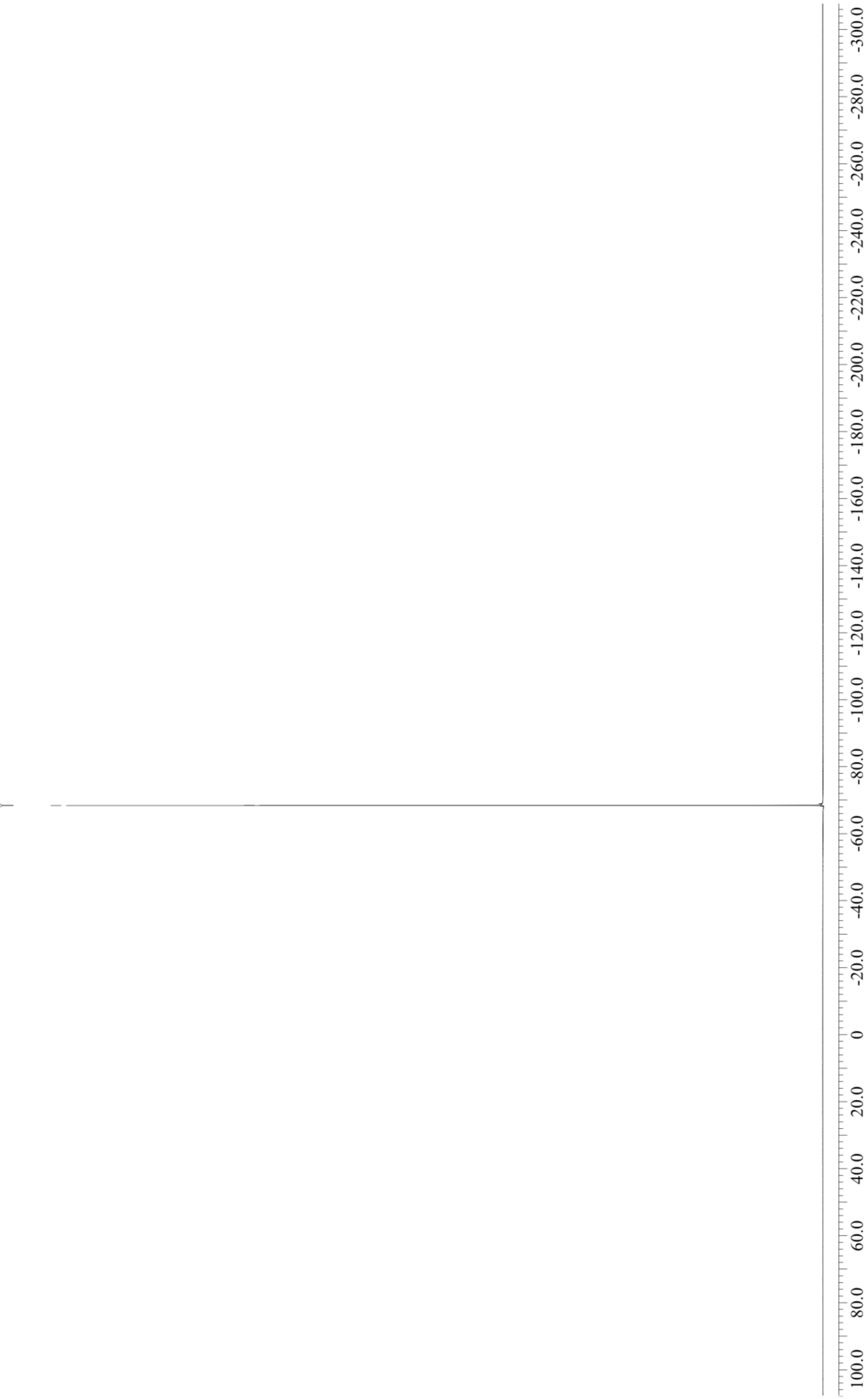

^{1}H NMR of **6** ($CDCl_3$, 400 MHz, 25 ºC)

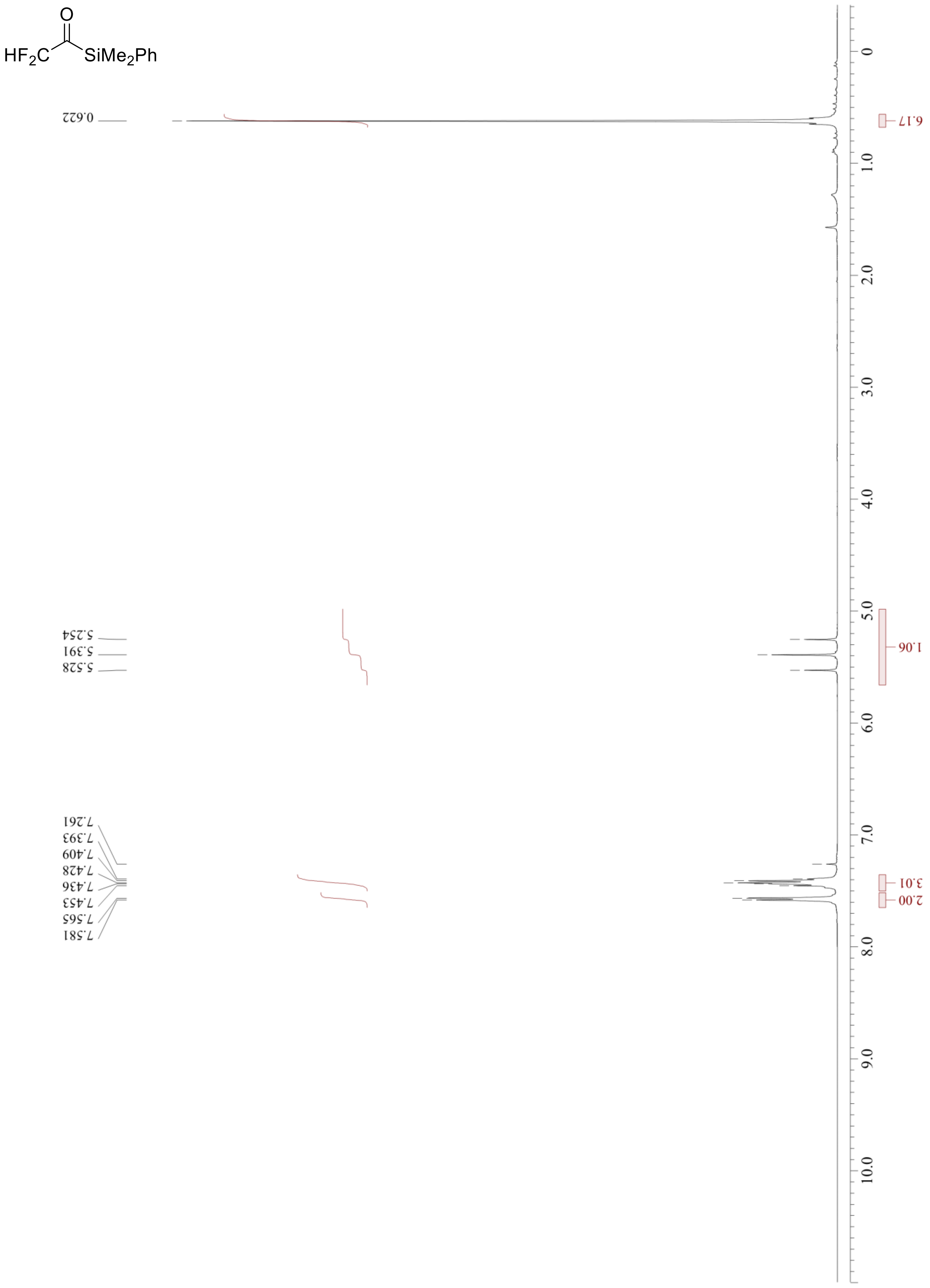

^{13}C NMR of **6** ($CDCl_3$, 100 MHz, 25 ºC)

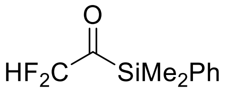

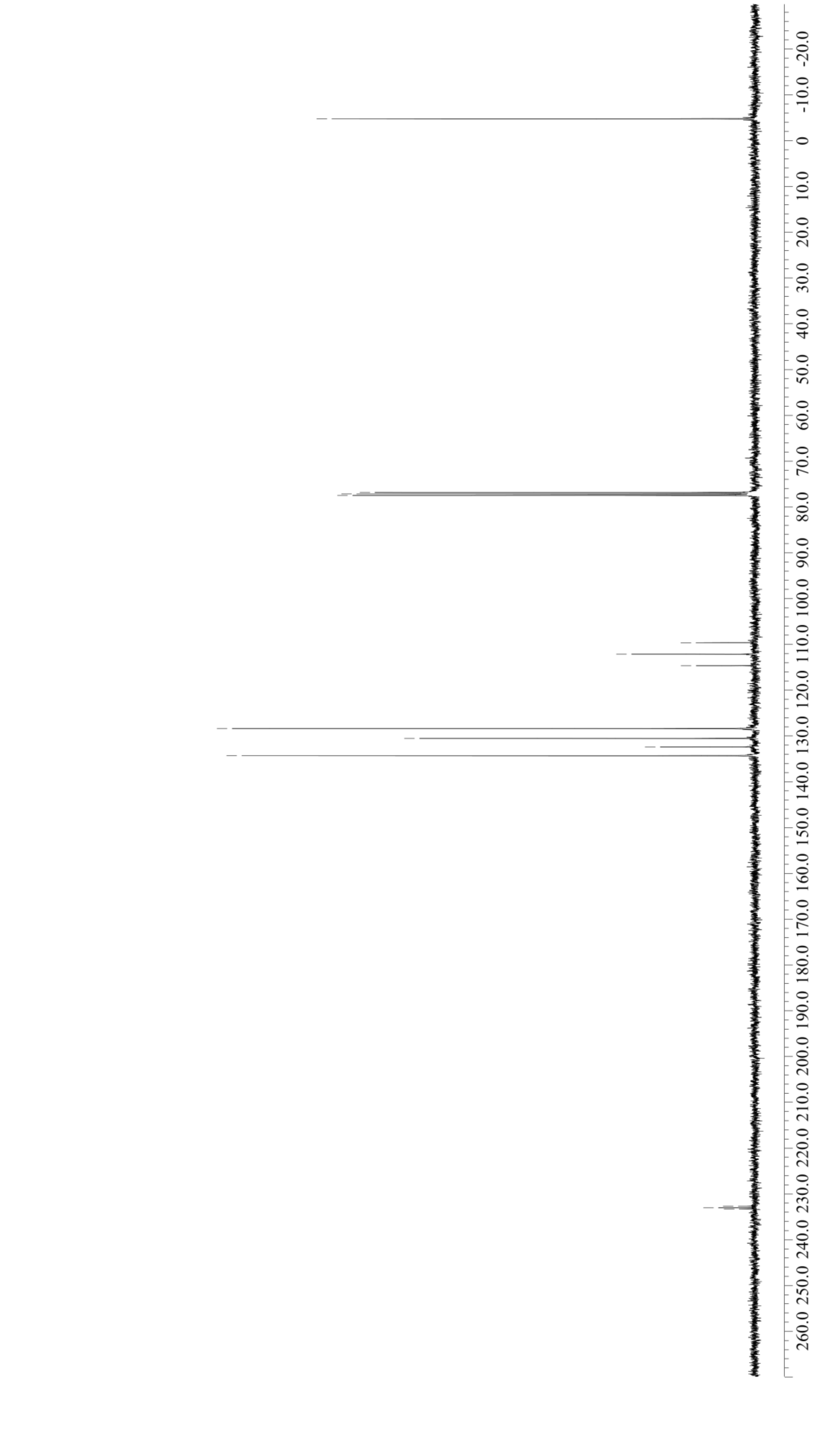

^{19}F NMR of **6** ($CDCl_3$, 375 MHz, 25 ºC)

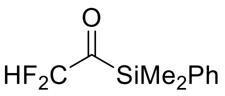

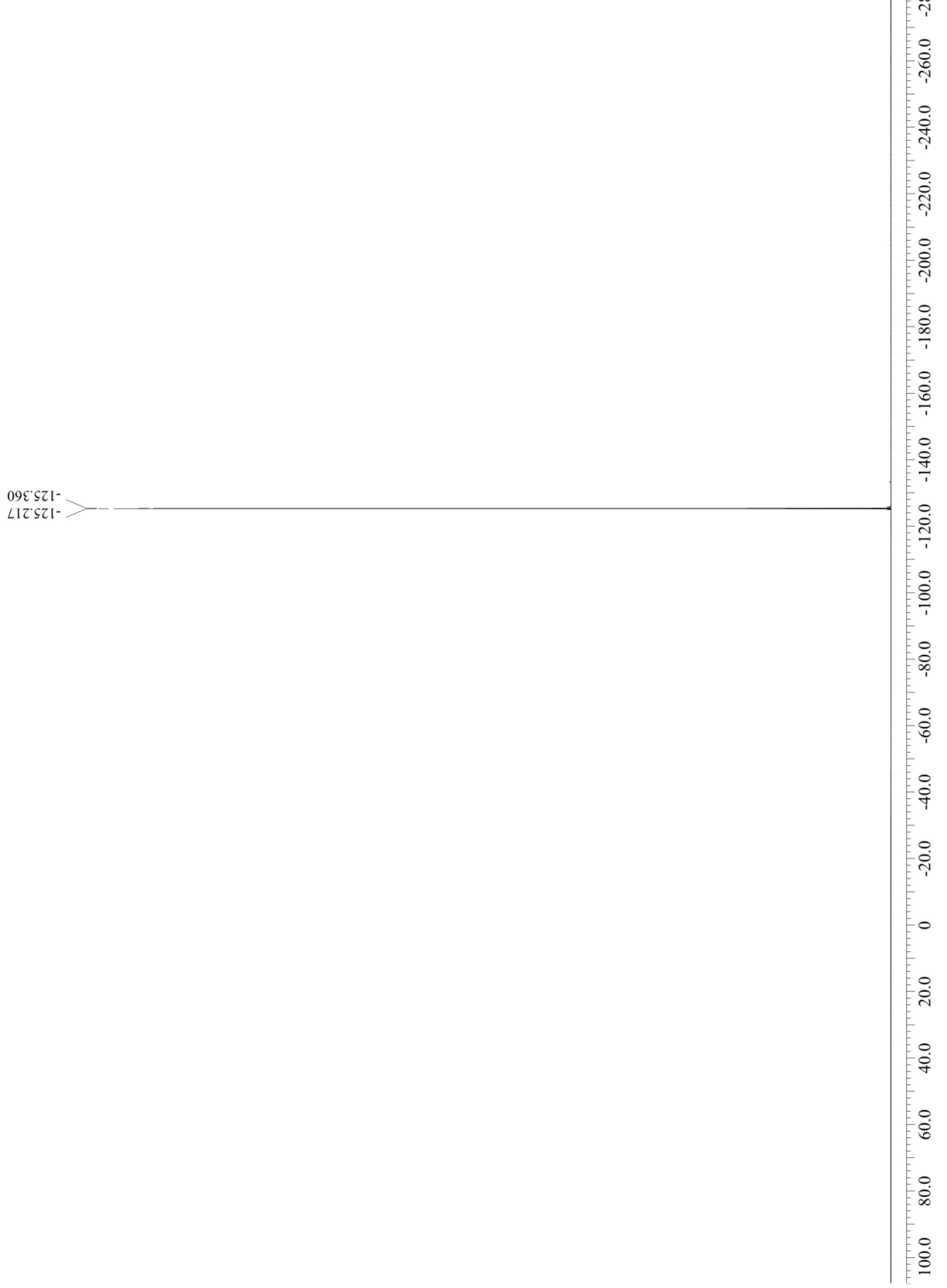

^{1}H NMR of **2a** ($CDCl_3$, 400 MHz, 25 ºC)

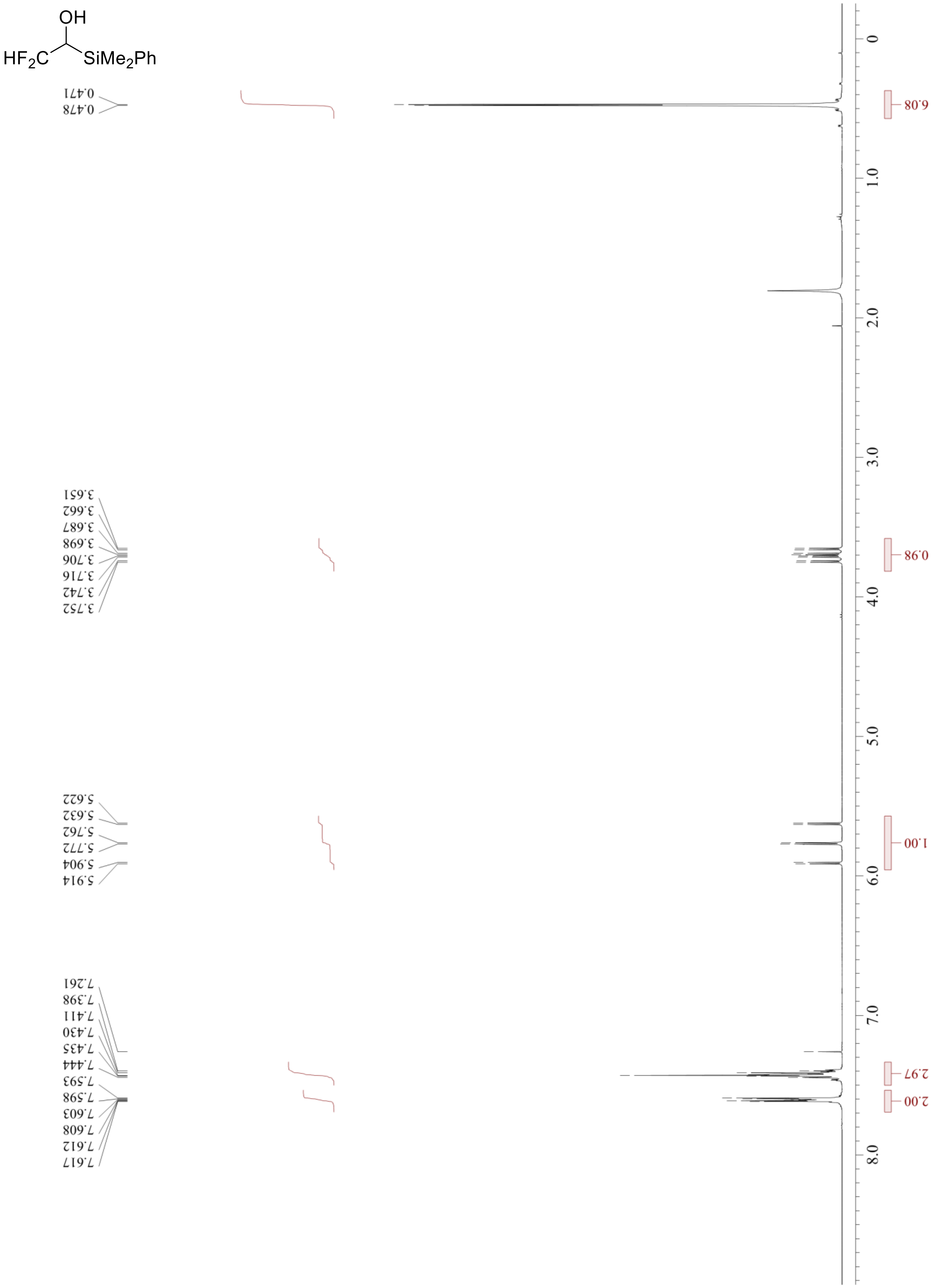

^{13}C NMR of **2a** ($CDCl_3$, 100 MHz, 25 ºC)

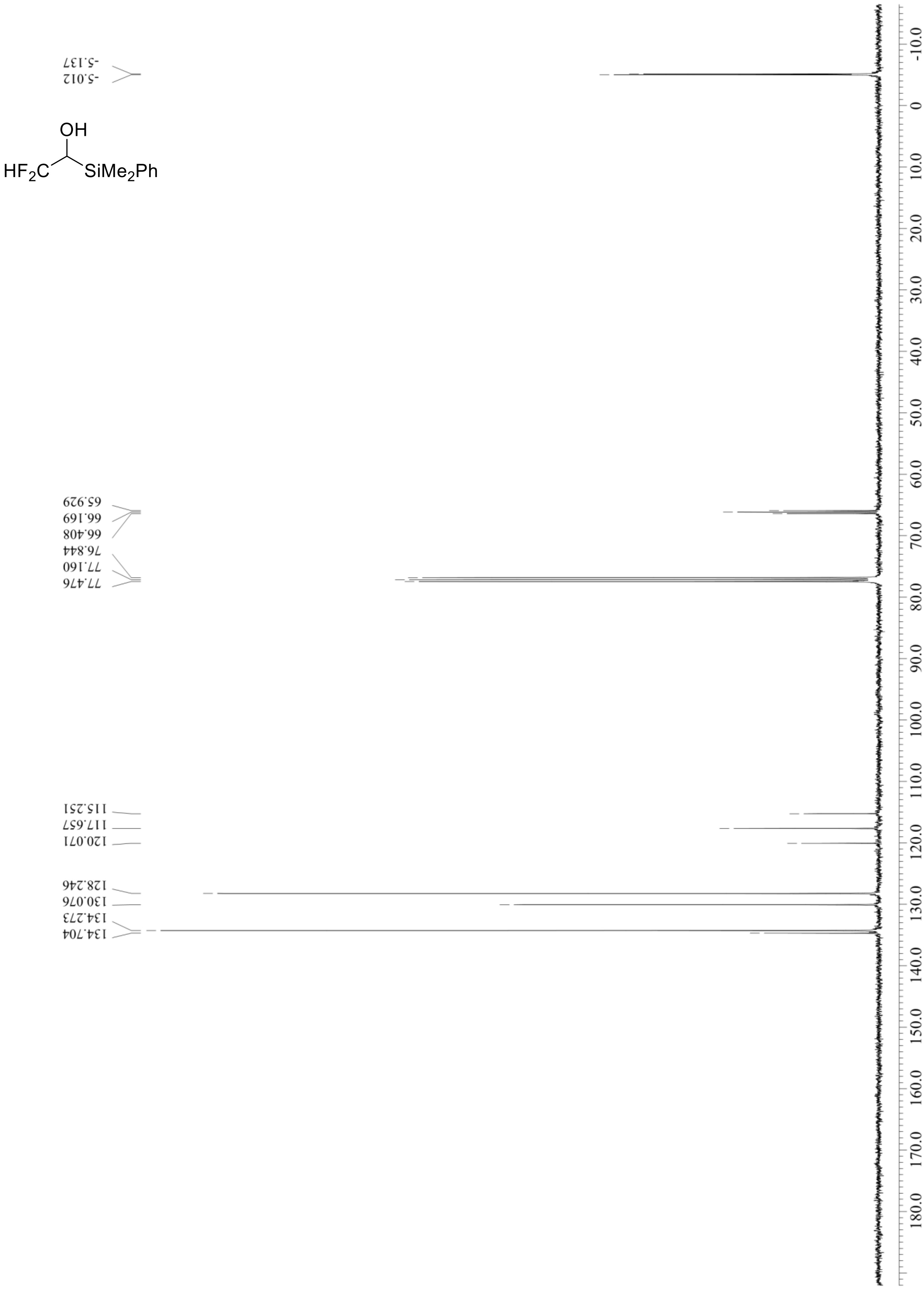

^{19}F NMR of **2a** ($CDCl_3$, 375 MHz, 25 ºC)

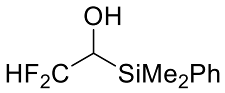

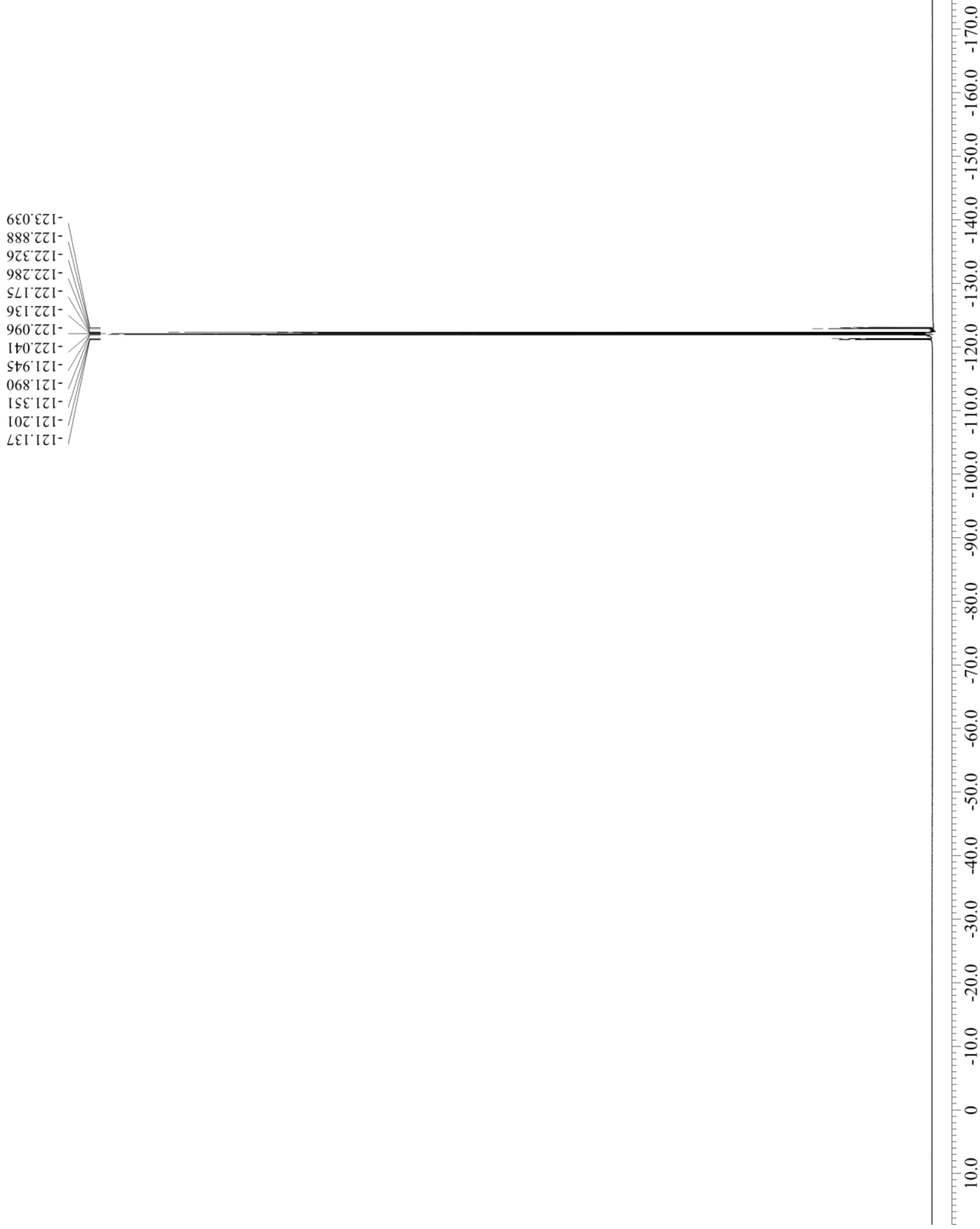

^{1}H NMR of **7j** ($CDCl_3$, 400 MHz, 25 ºC)

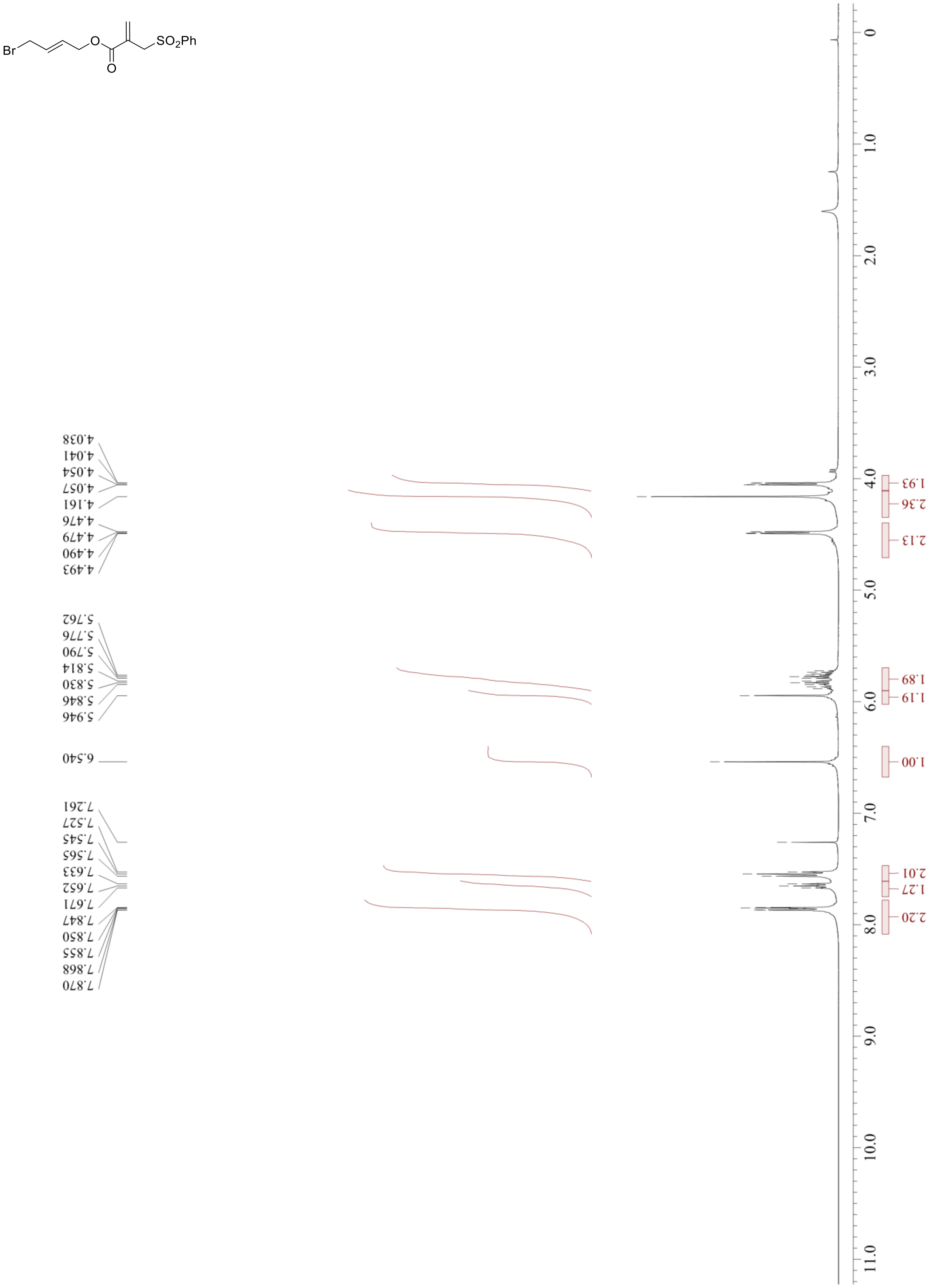

^{13}C NMR of **7j** ($CDCl_3$, 100 MHz, 25 ºC)

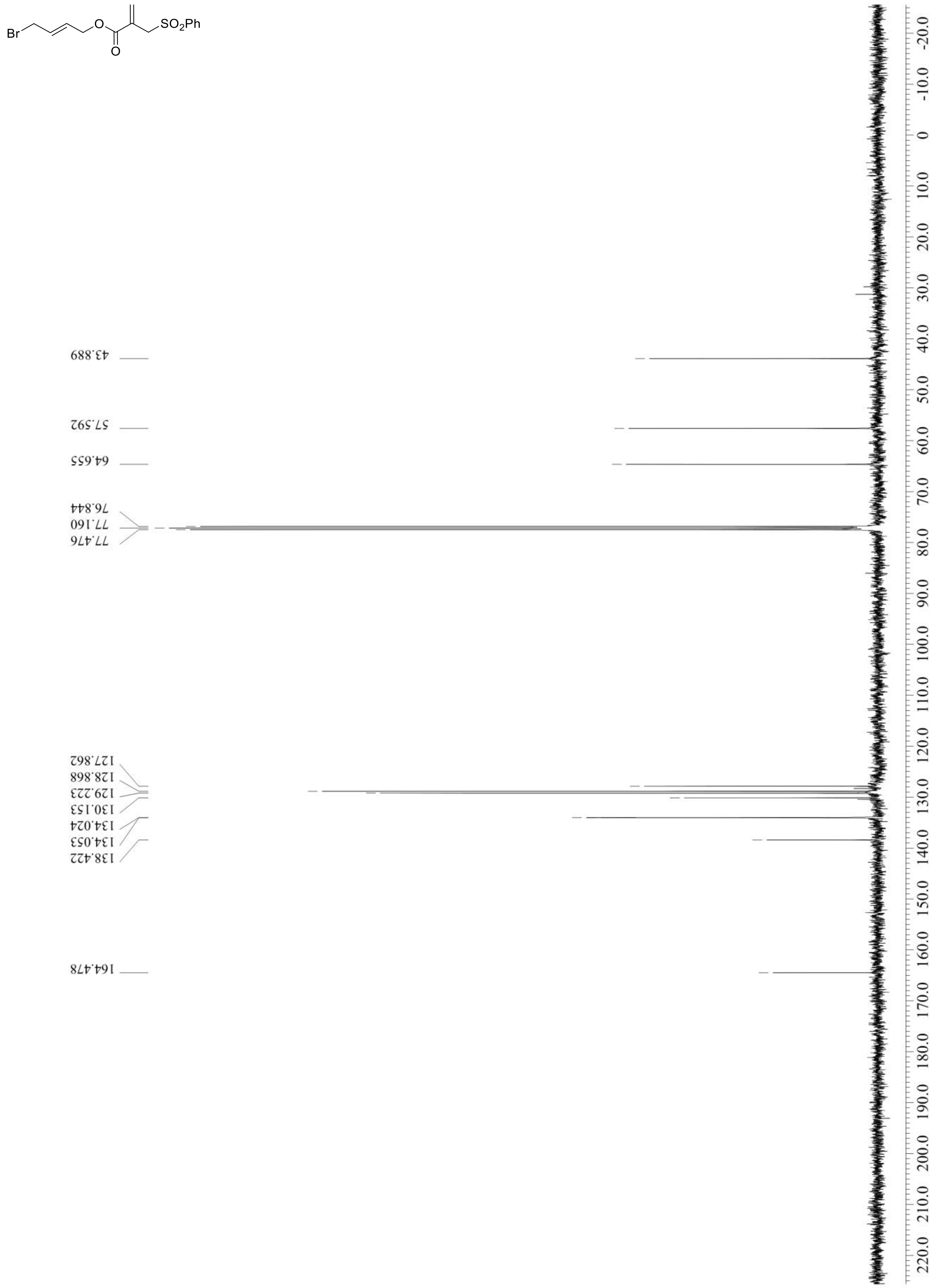

^{1}H NMR of **7r** ($CDCl_3$, 400 MHz, 25 ºC)

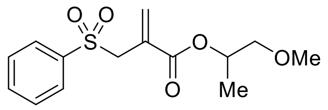

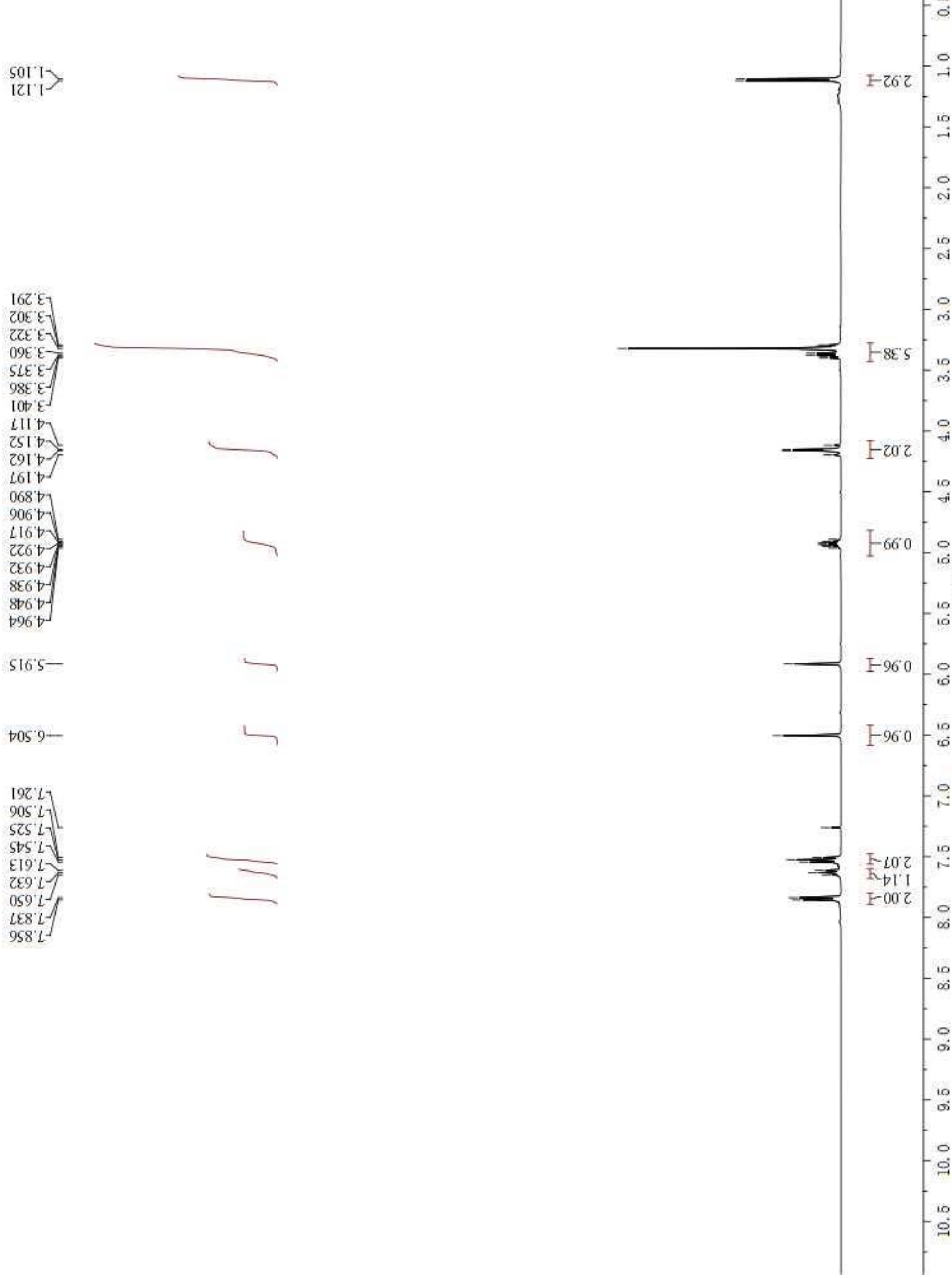

^{13}C NMR of **7r** ($CDCl_3$, 100 MHz, 25 ºC)

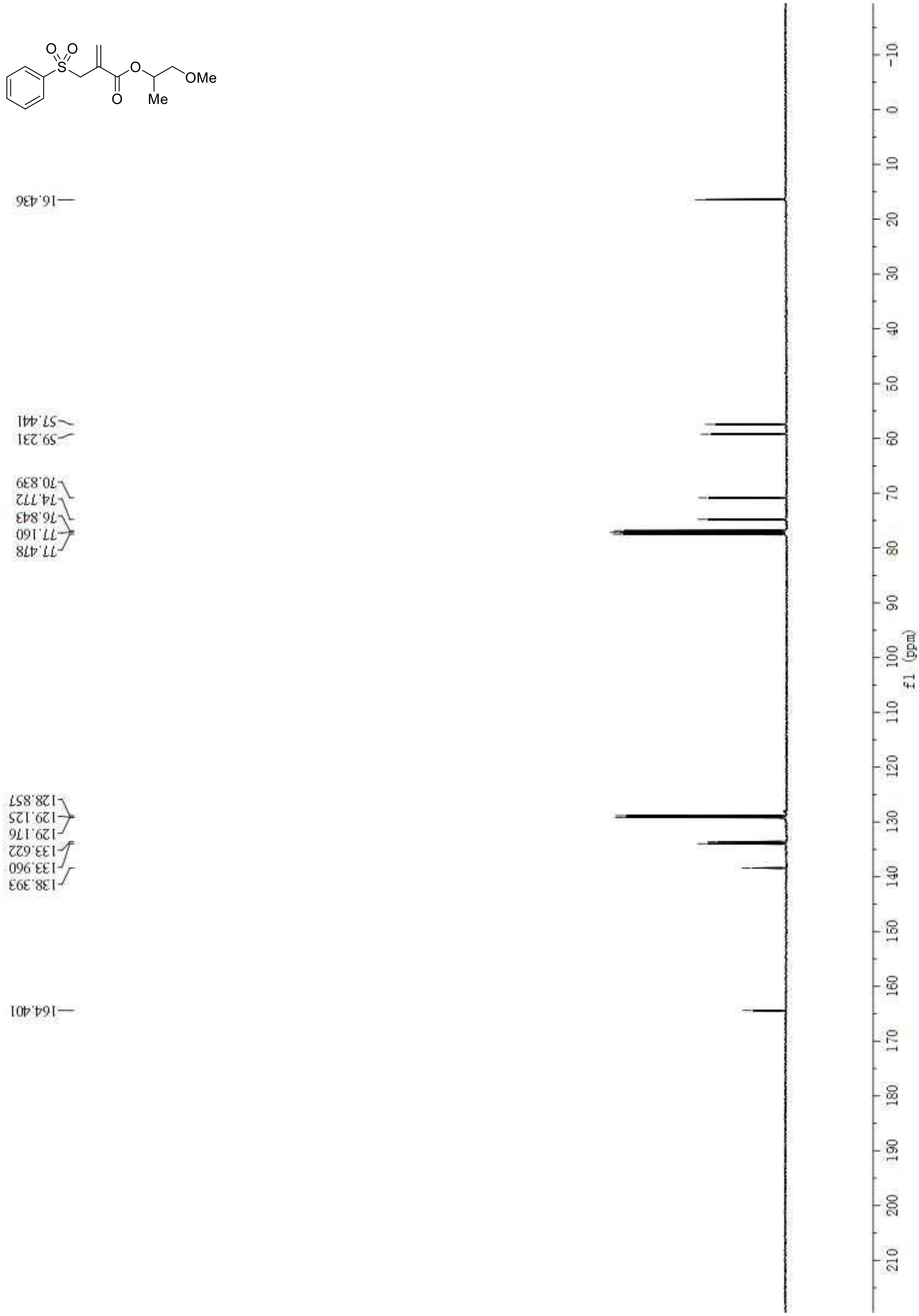

^{1}H NMR of **7s** ($CDCl_3$, 400 MHz, 25 ºC)

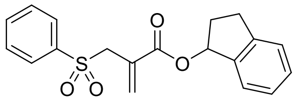

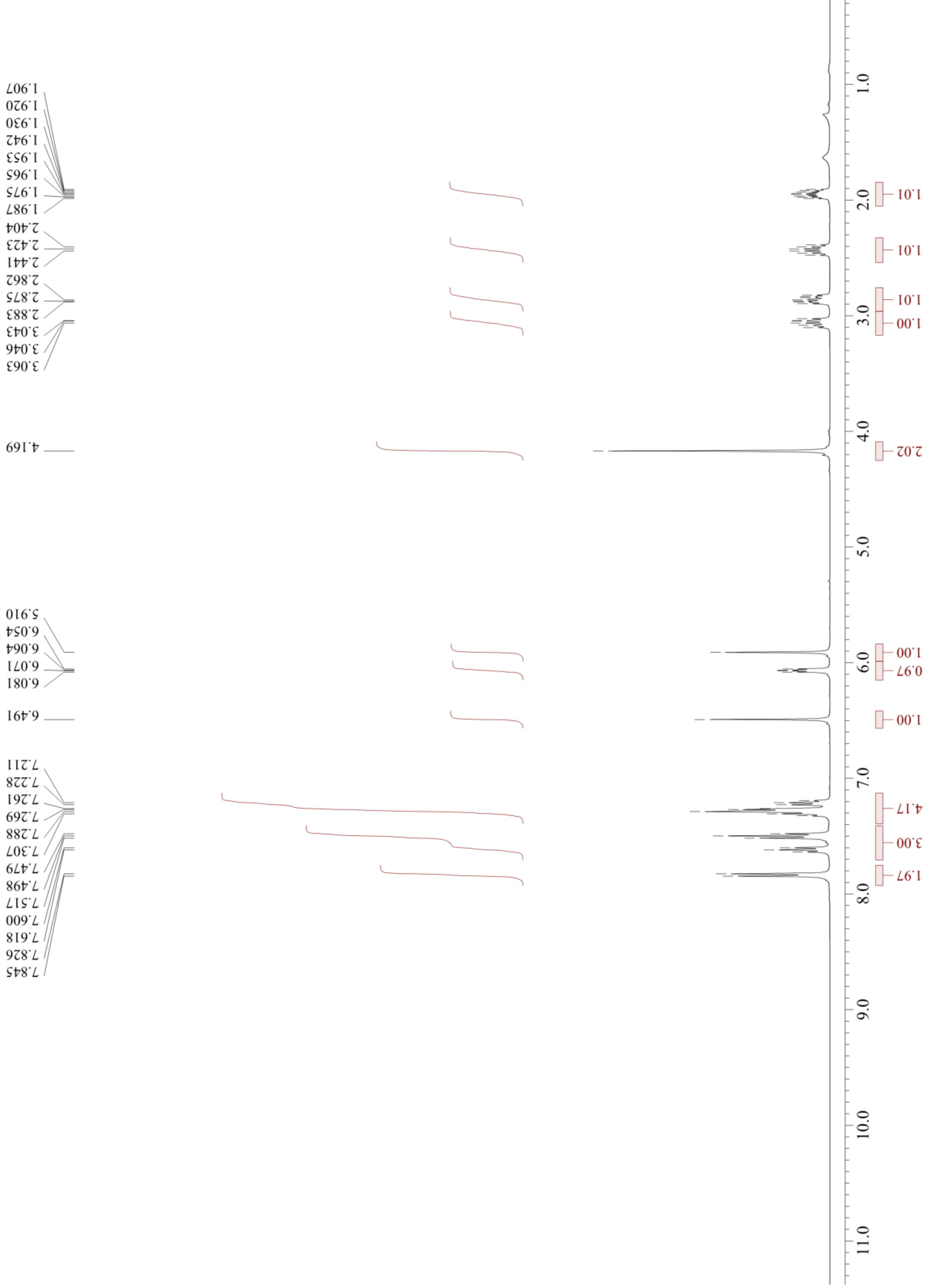

^{13}C NMR of **7s** ($CDCl_3$, 100 MHz, 25 ºC)

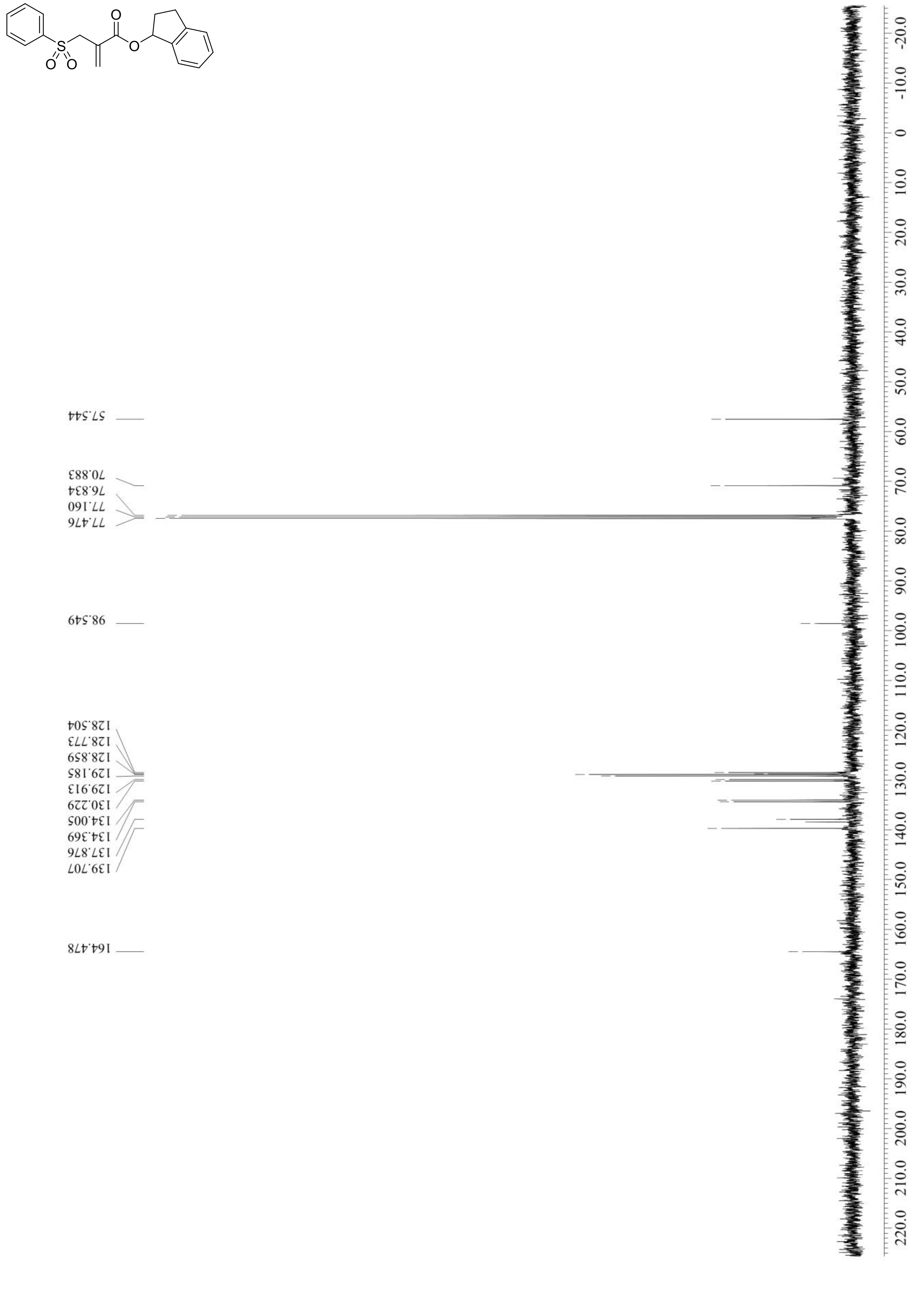

^{1}H NMR of **7t** ($CDCl_3$, 400 MHz, 25 ºC)

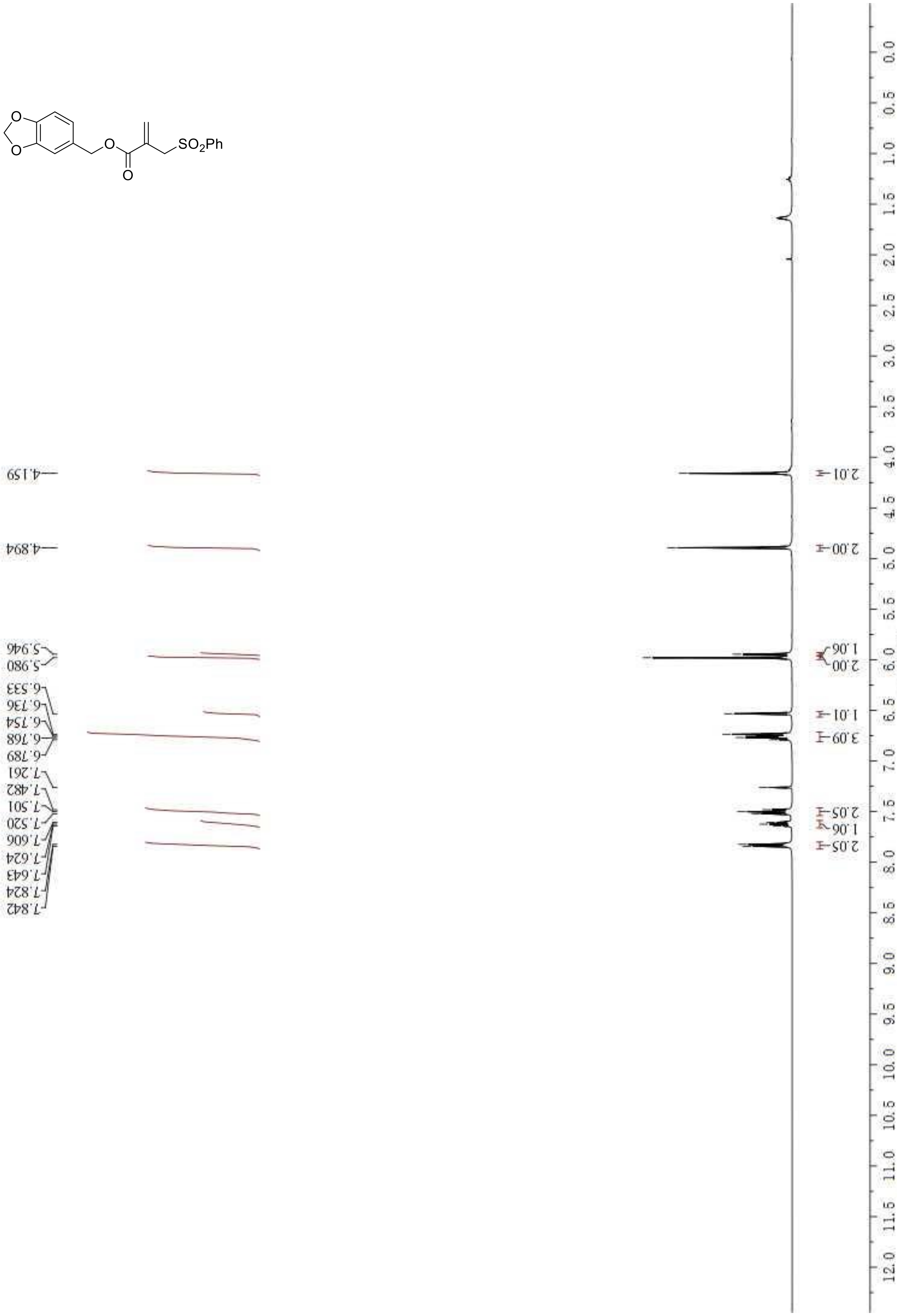

^{13}C NMR of **7t** ($CDCl_3$, 100 MHz, 25 ºC)

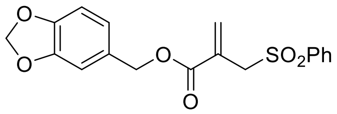

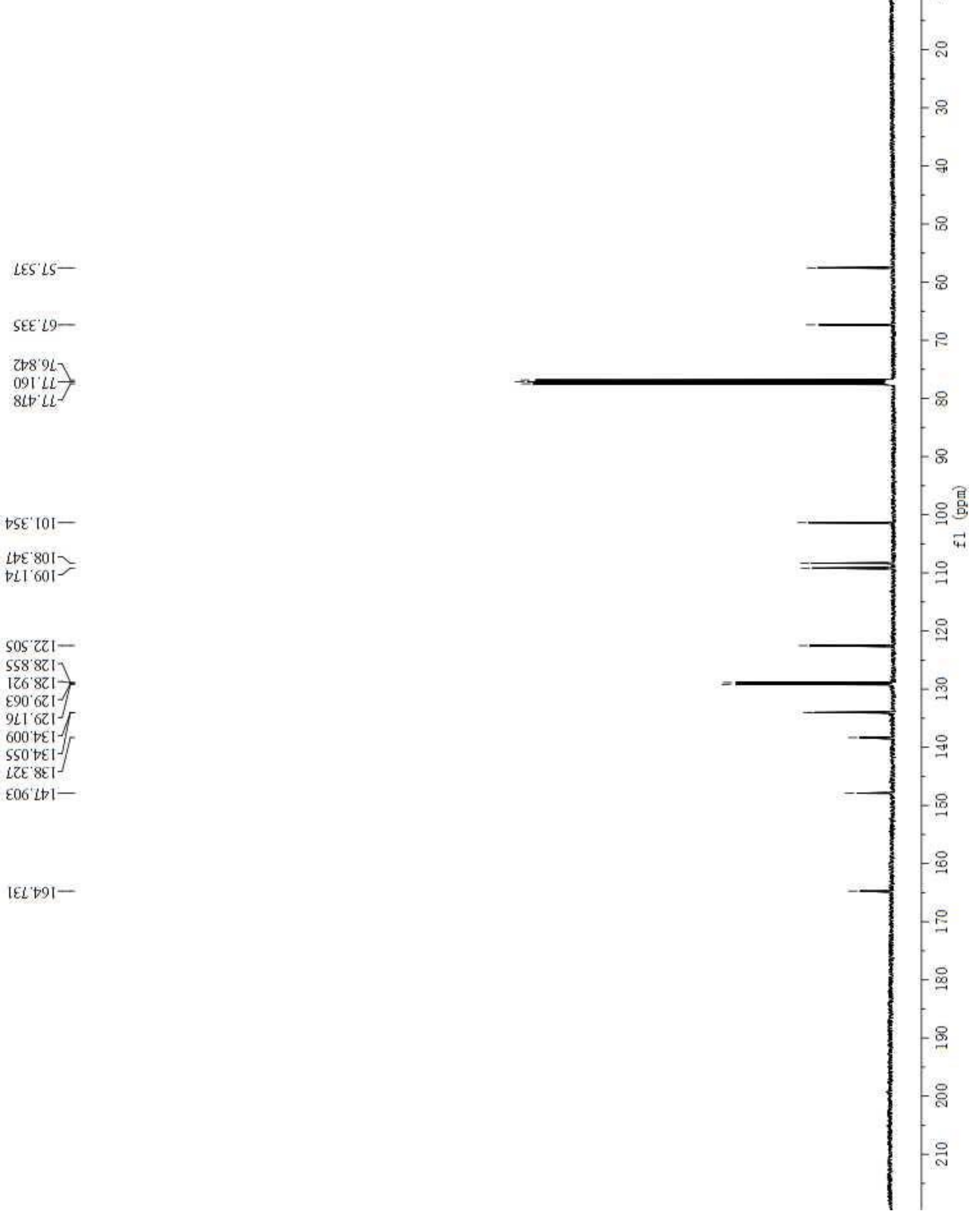

^{1}H NMR of **7u** ($CDCl_3$, 400 MHz, 25 °C)

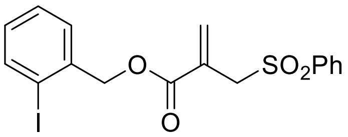

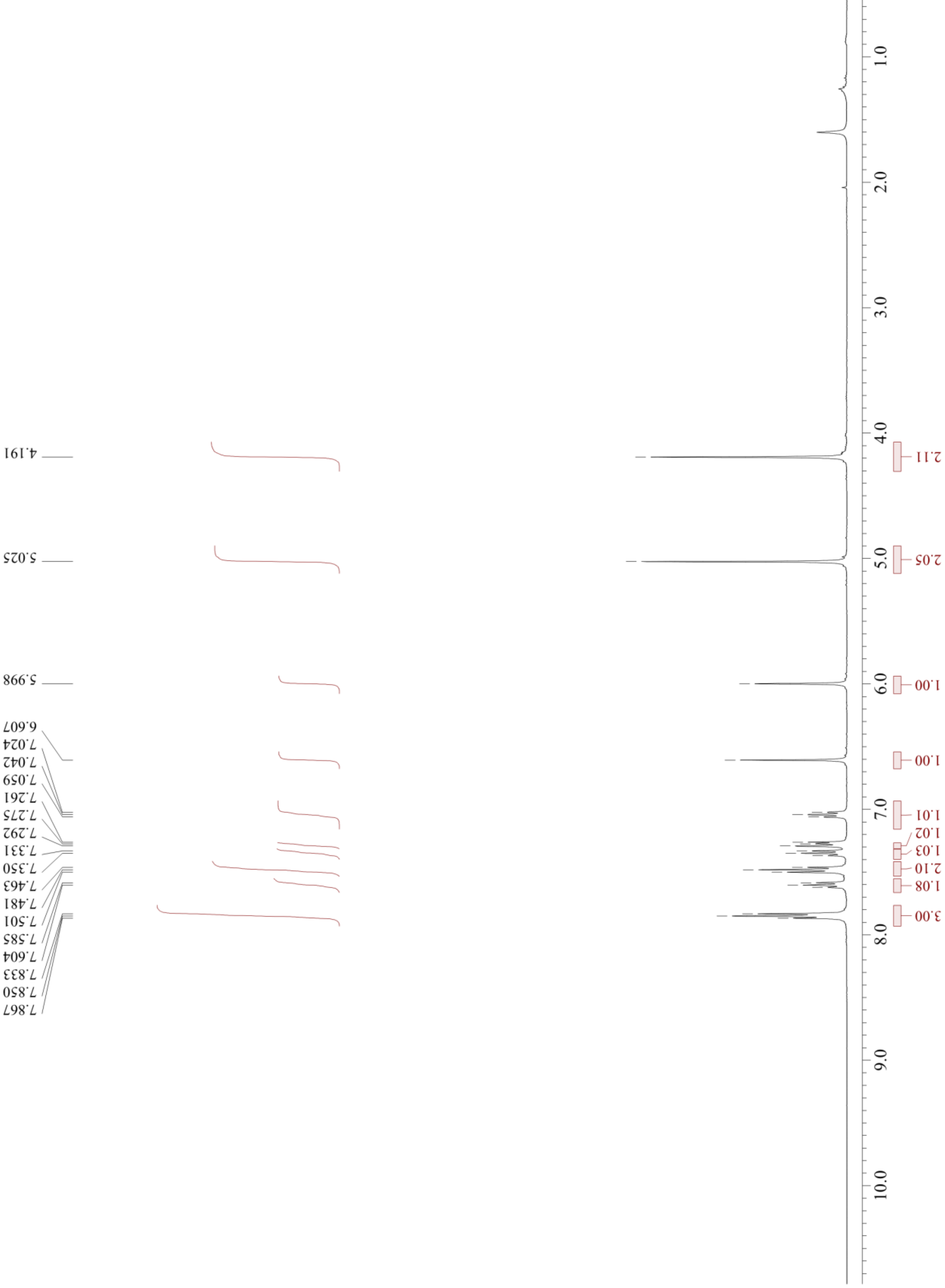

^{13}C NMR of **7u** ($CDCl_3$, 100 MHz, 25 ºC)

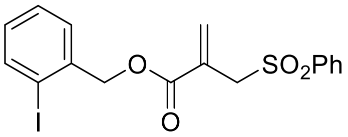

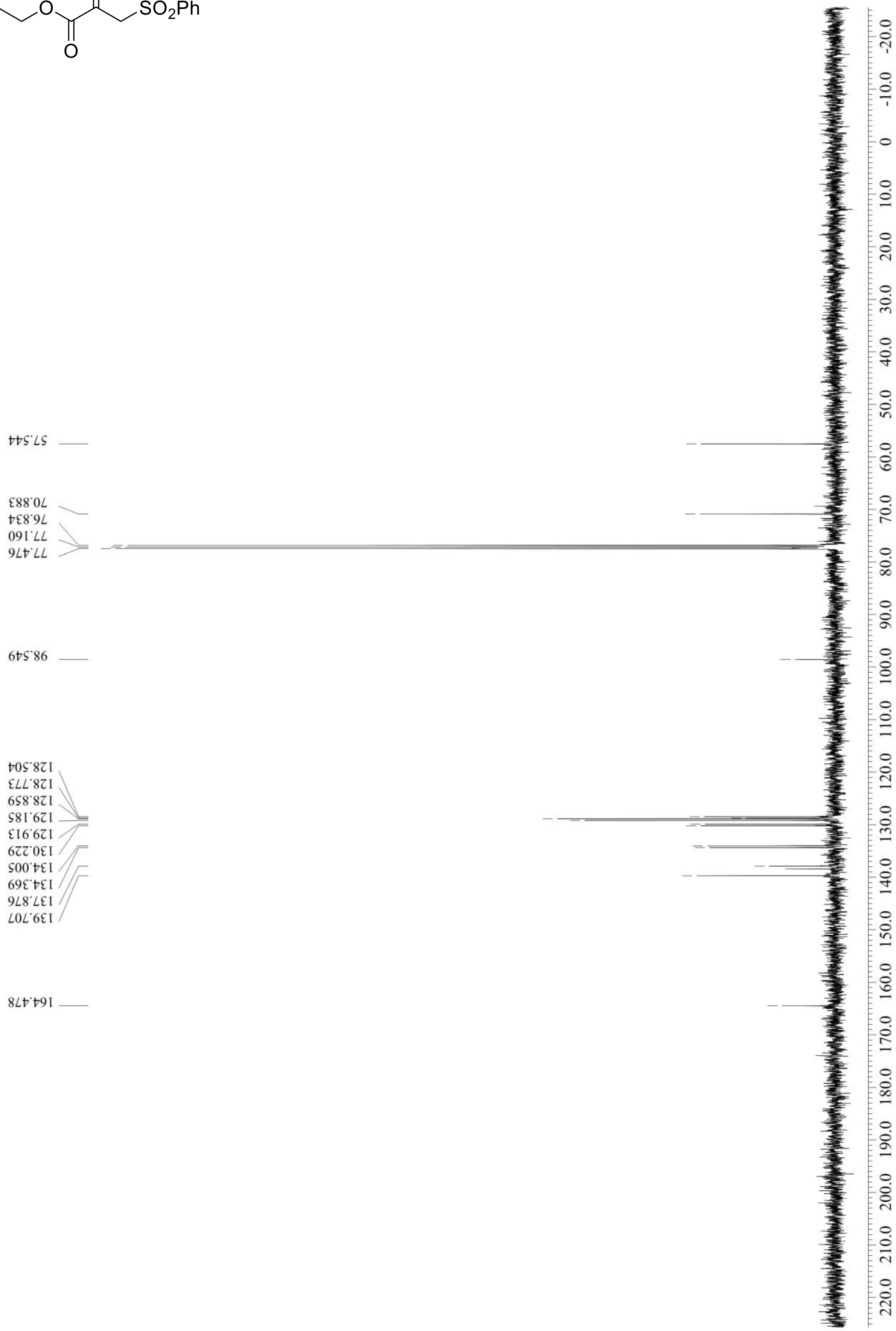

^{1}H NMR of **7v** ($CDCl_3$, 400 MHz, 25 ºC)

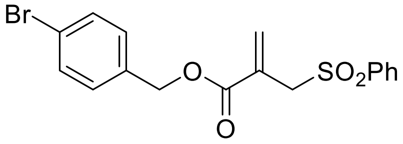

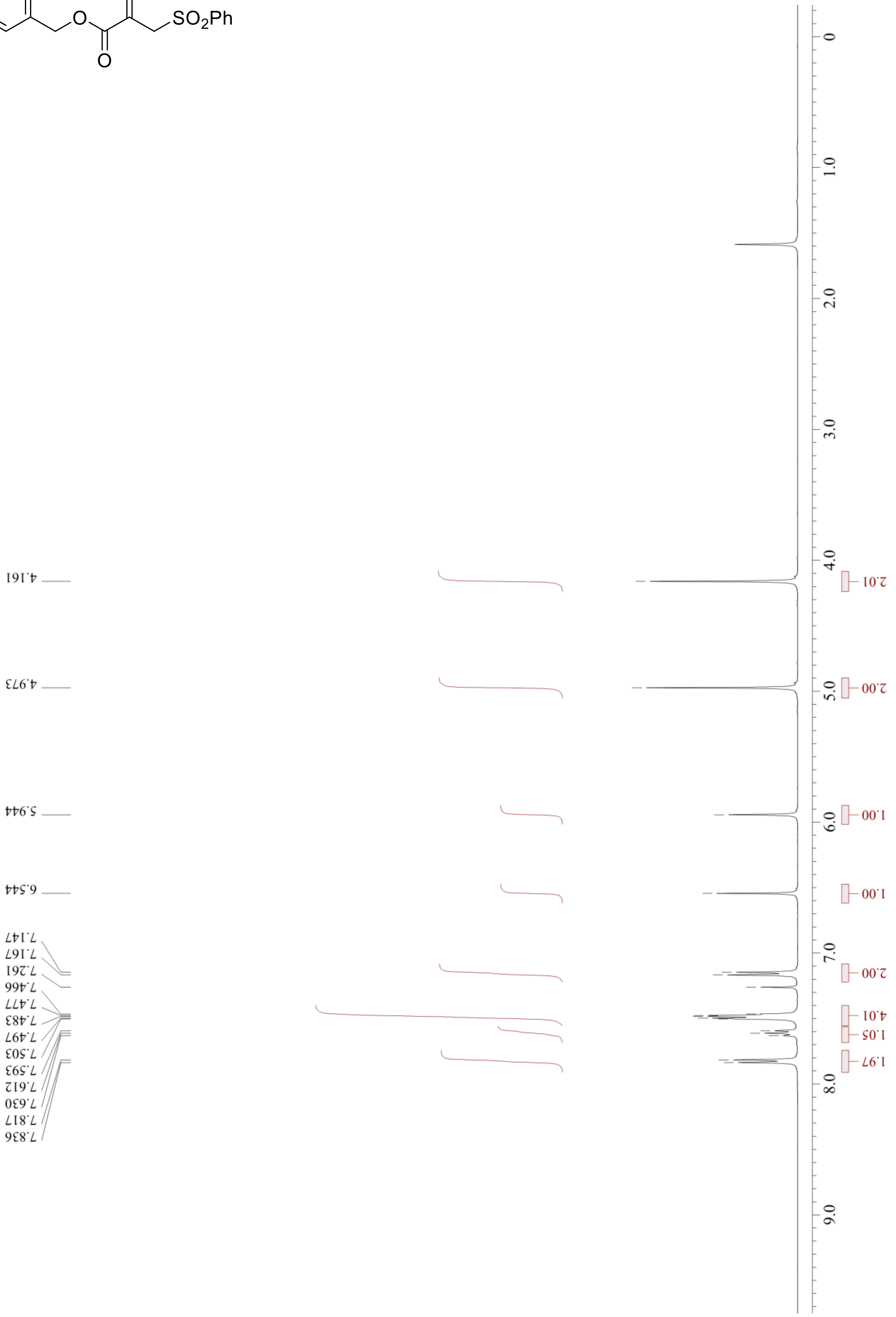

^{13}C NMR of **7v** ($CDCl_3$, 100 MHz, 25 ºC)

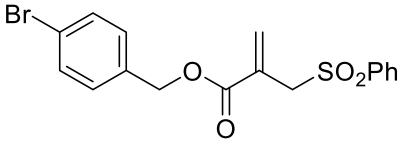

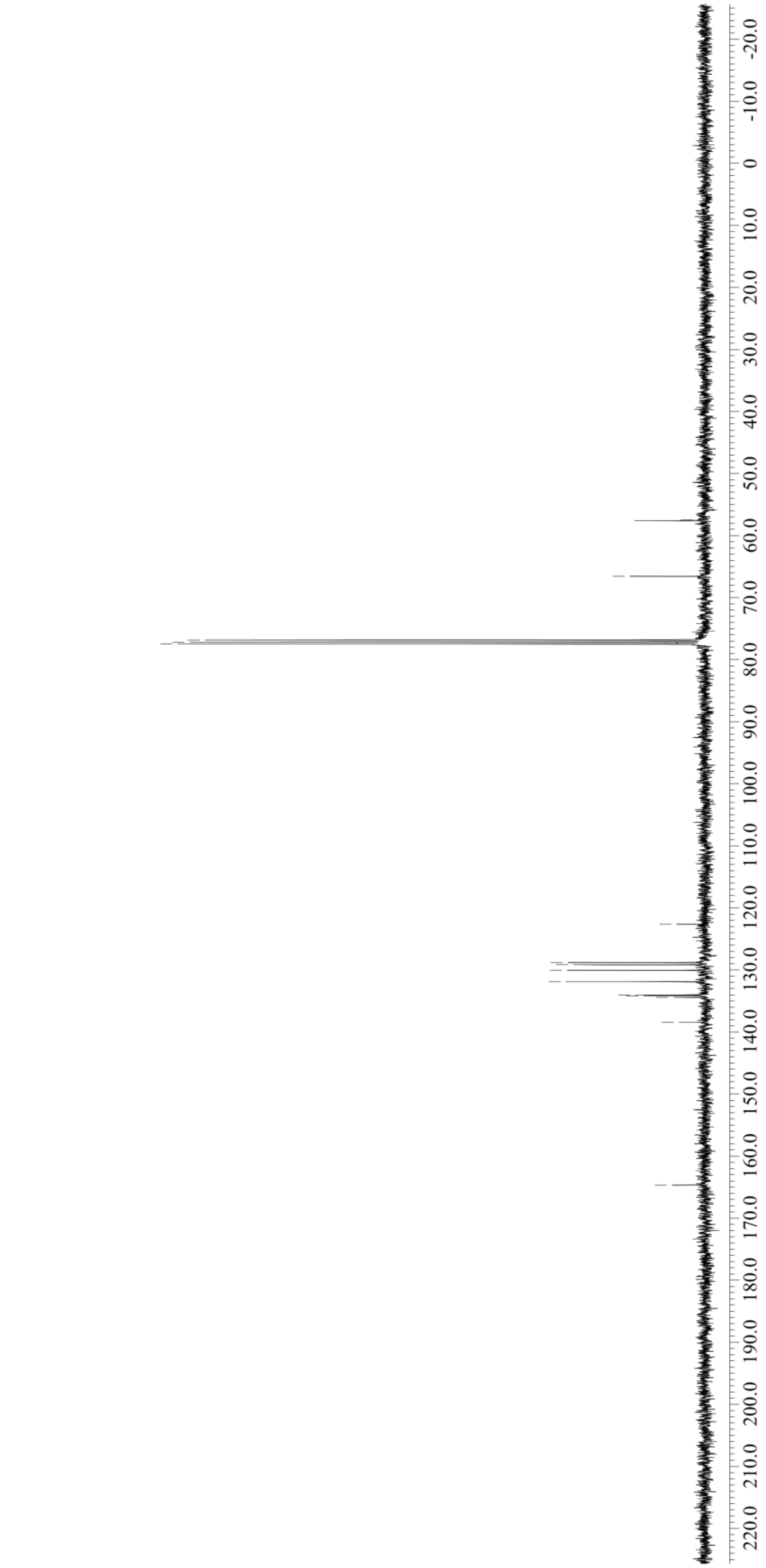

^{1}H NMR of **7x** ($CDCl_3$, 400 MHz, 25 ºC)

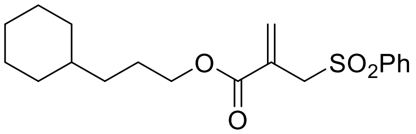

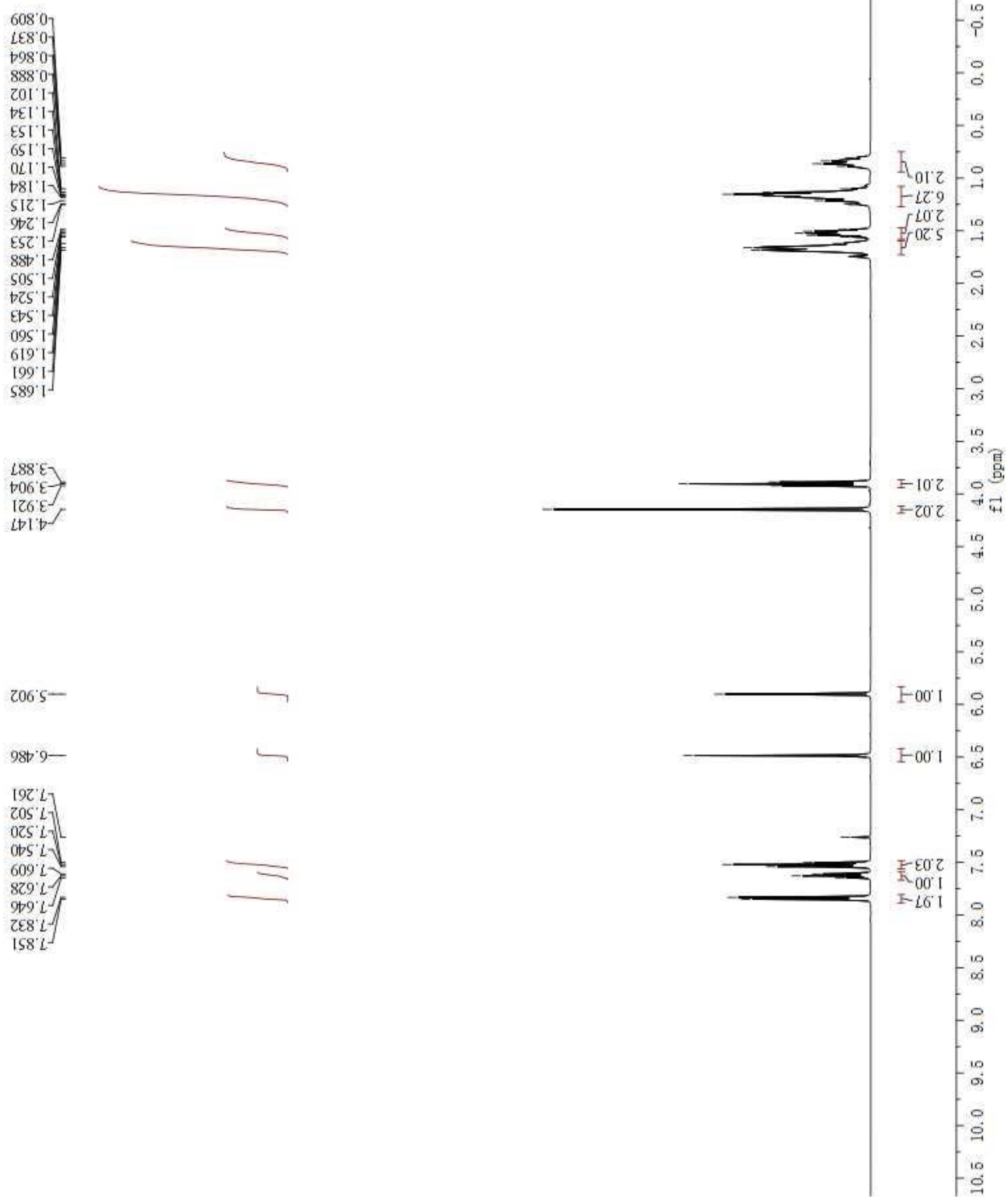

^{13}C NMR of **7x** ($CDCl_3$, 100 MHz, 25 ºC)

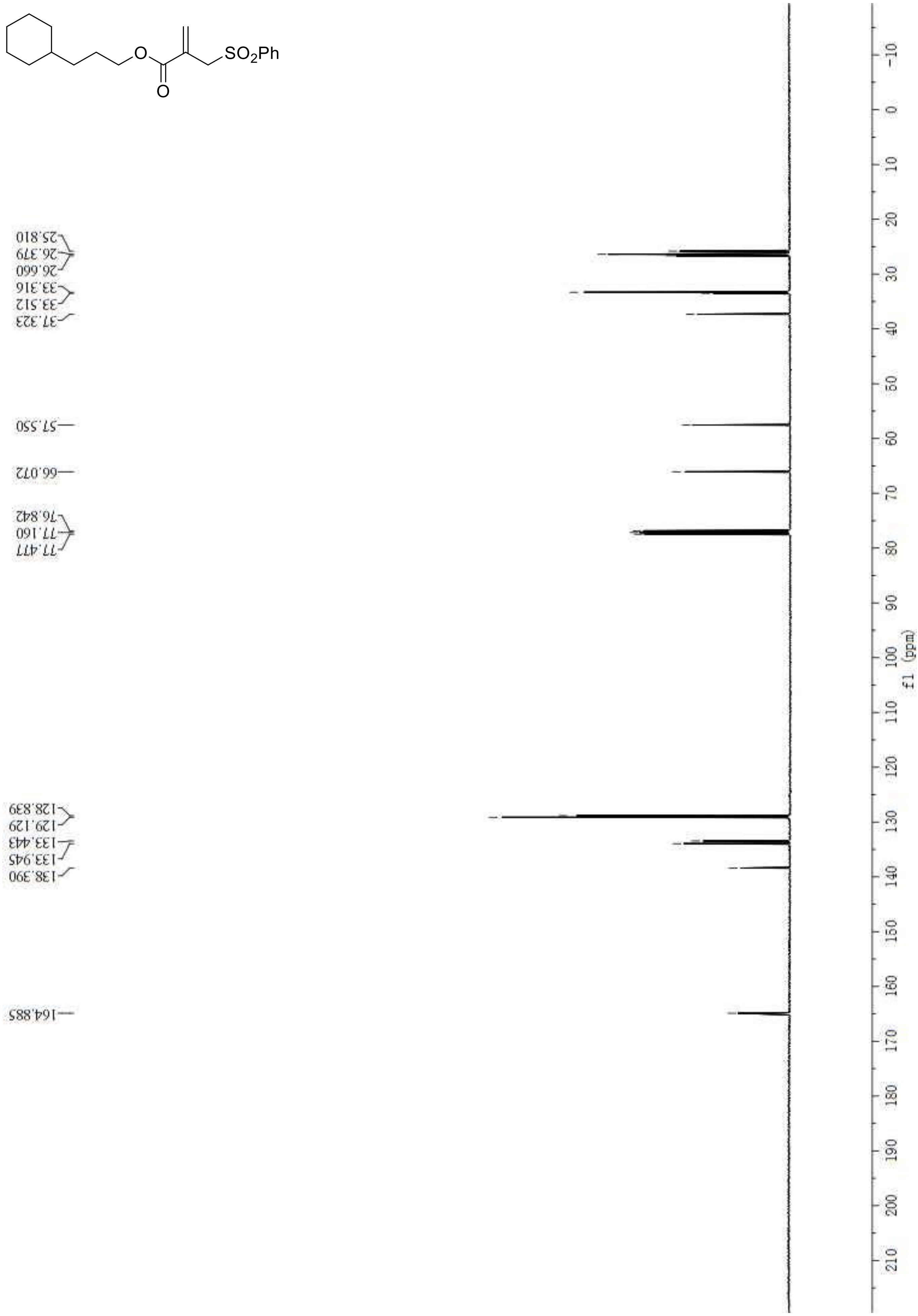

^{1}H NMR of **7y** ($CDCl_3$, 400 MHz, 25 °C)

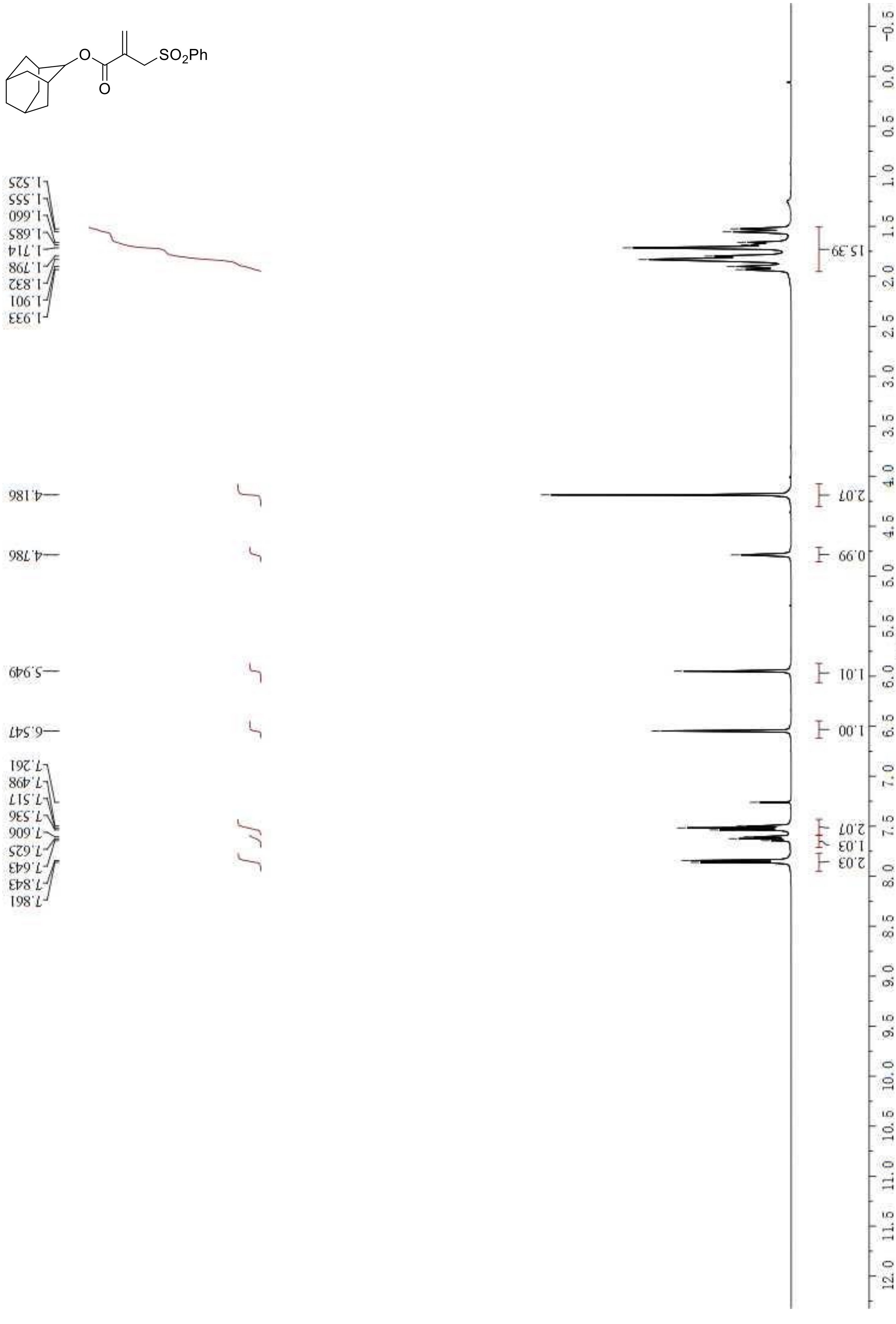

^{13}C NMR of **7y** ($CDCl_3$, 100 MHz, 25 ºC)

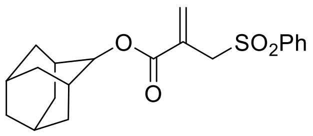

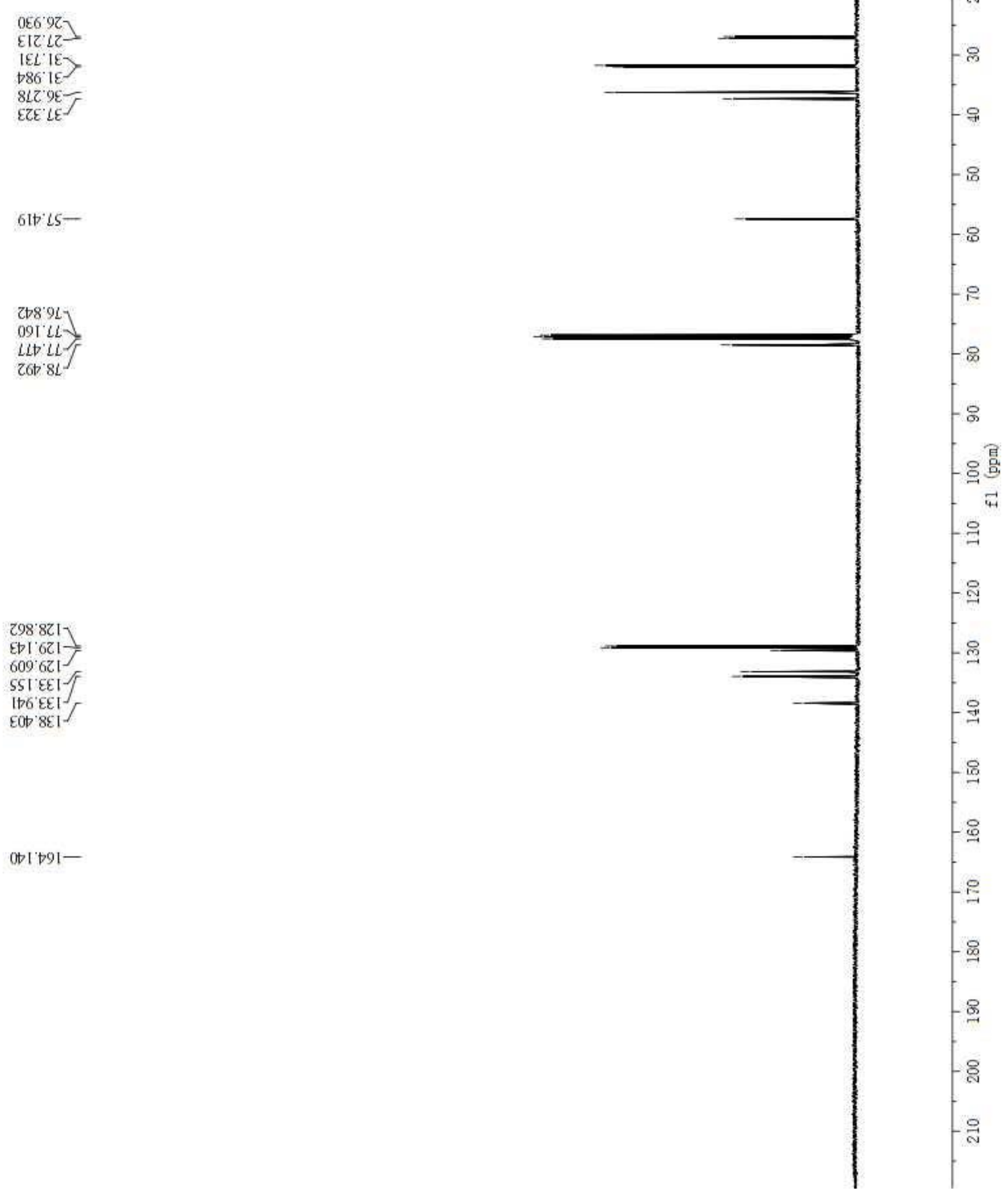

^{1}H NMR of **7z**($CDCl_3$, 400 MHz, 25 ºC)

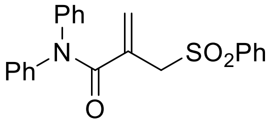

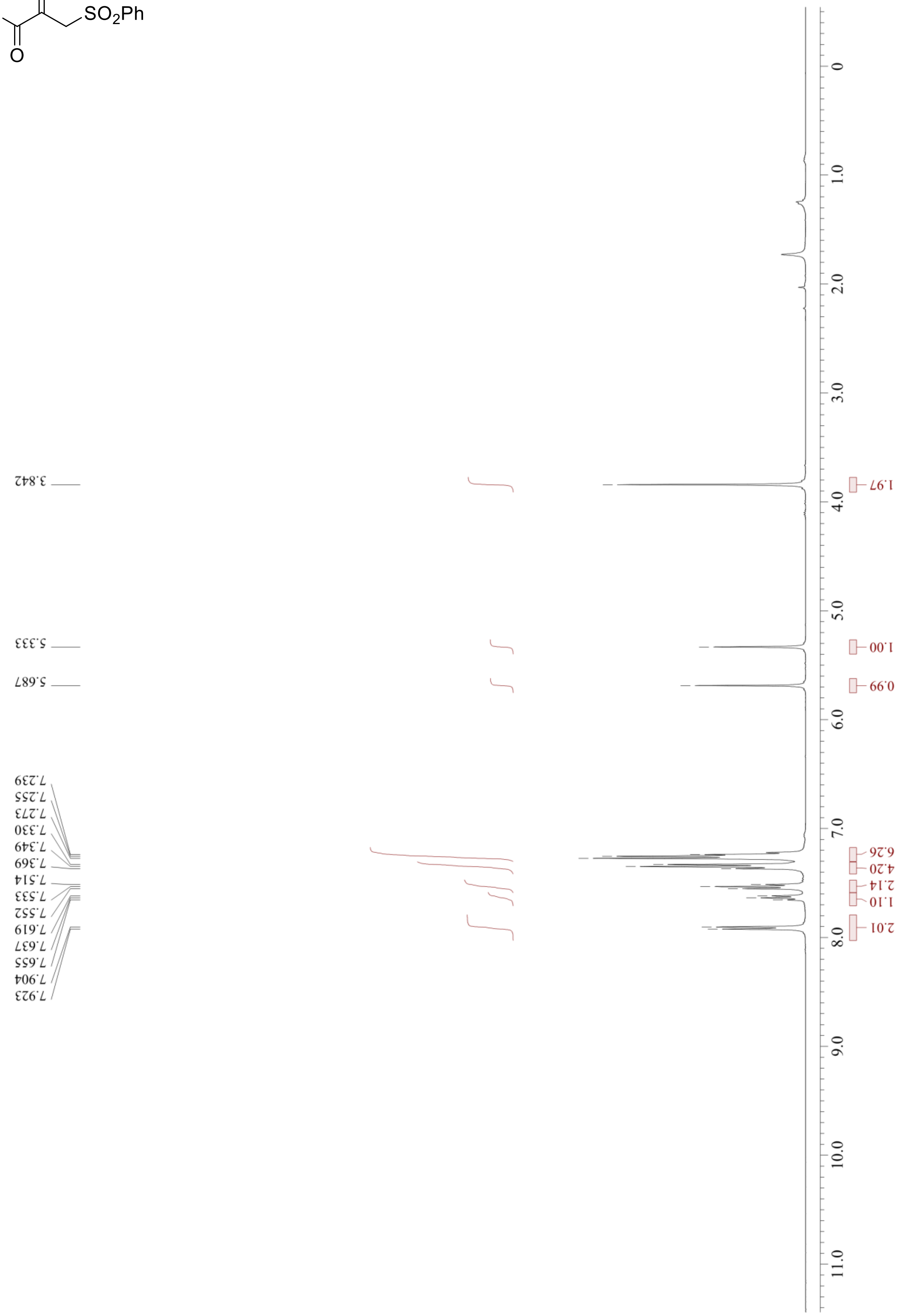

^{13}C NMR of **7z**(CDCl$_3$, 100 MHz, 25 ºC)

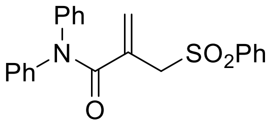

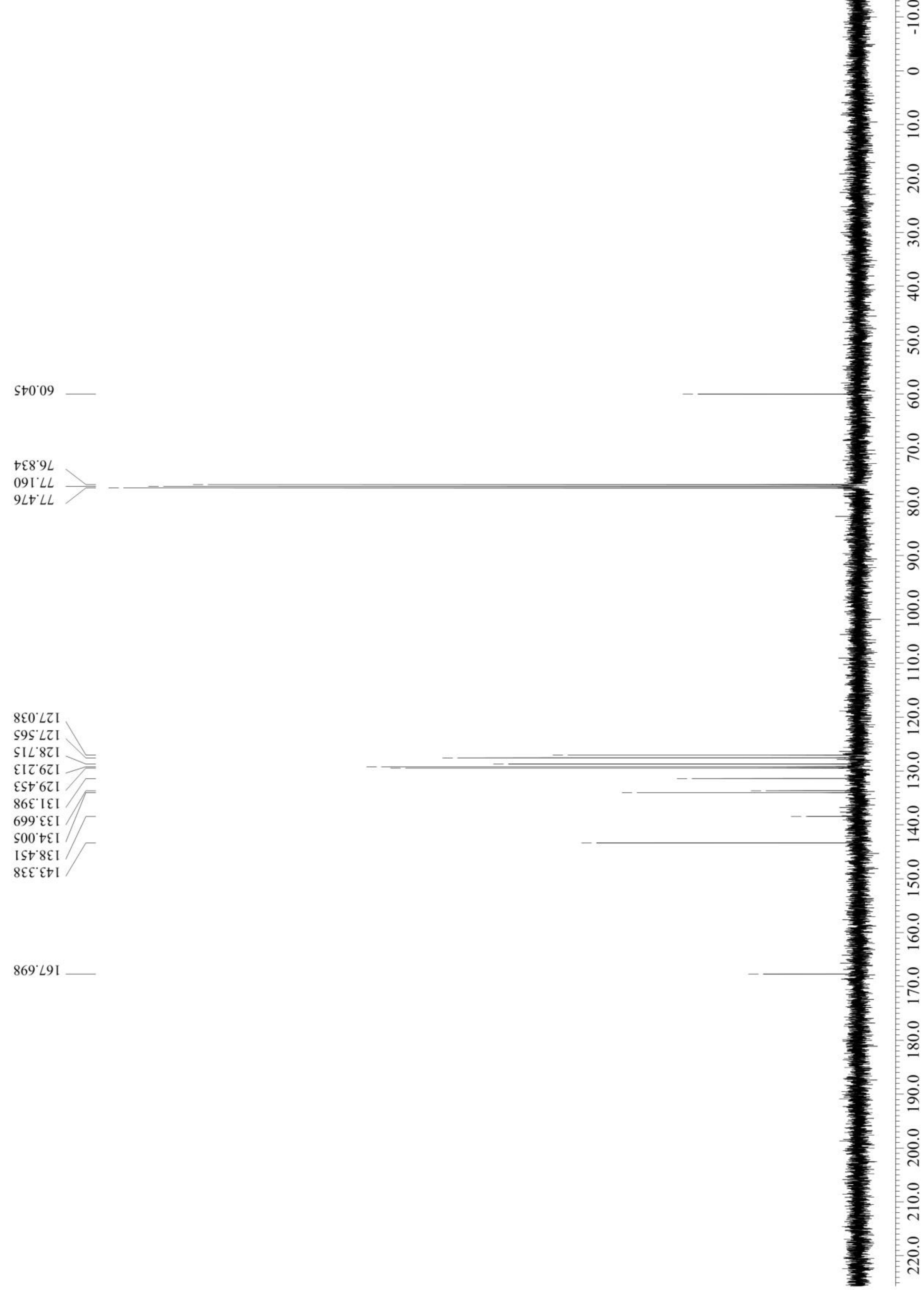

^{1}H NMR of **7ae** ($CDCl_3$, 400 MHz, 25 ºC)

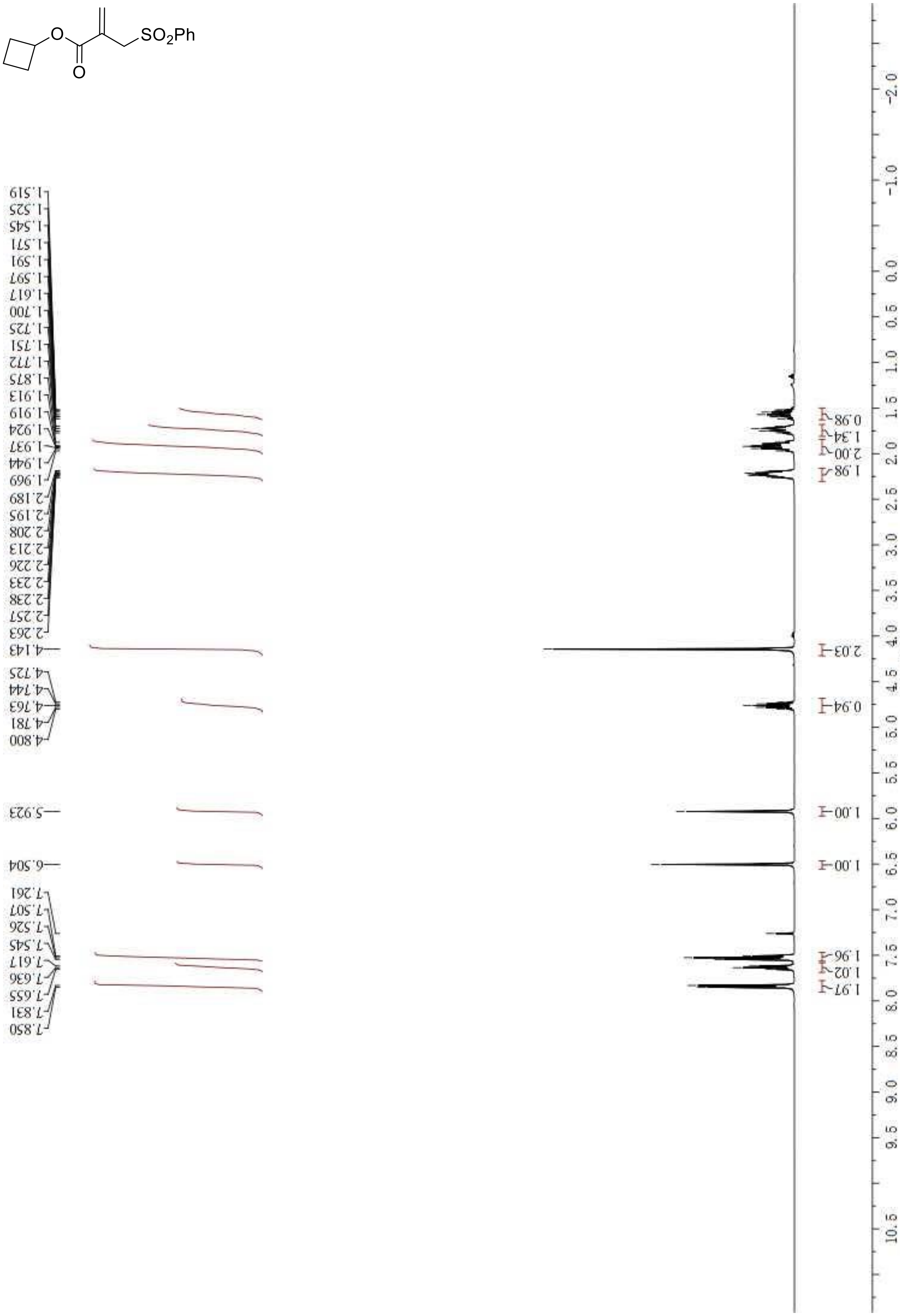

^{13}C NMR of **7ae** ($CDCl_3$, 100 MHz, 25 ºC)

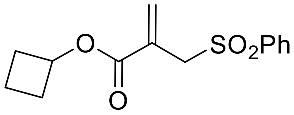

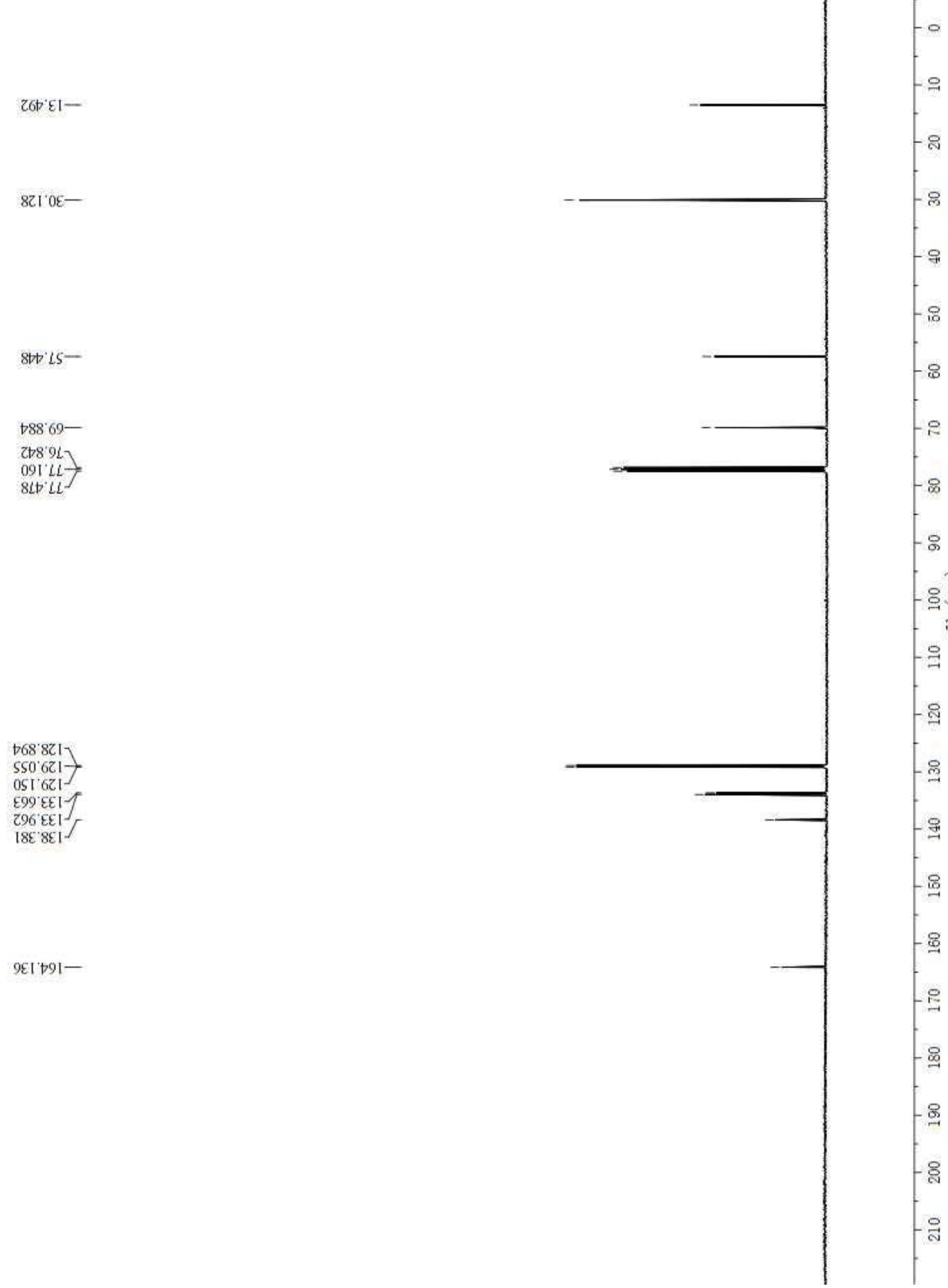

^{1}H NMR of **7ai** ($CDCl_3$, 400 MHz, 25 ºC)

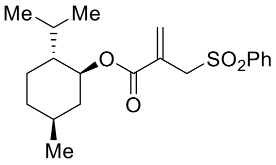

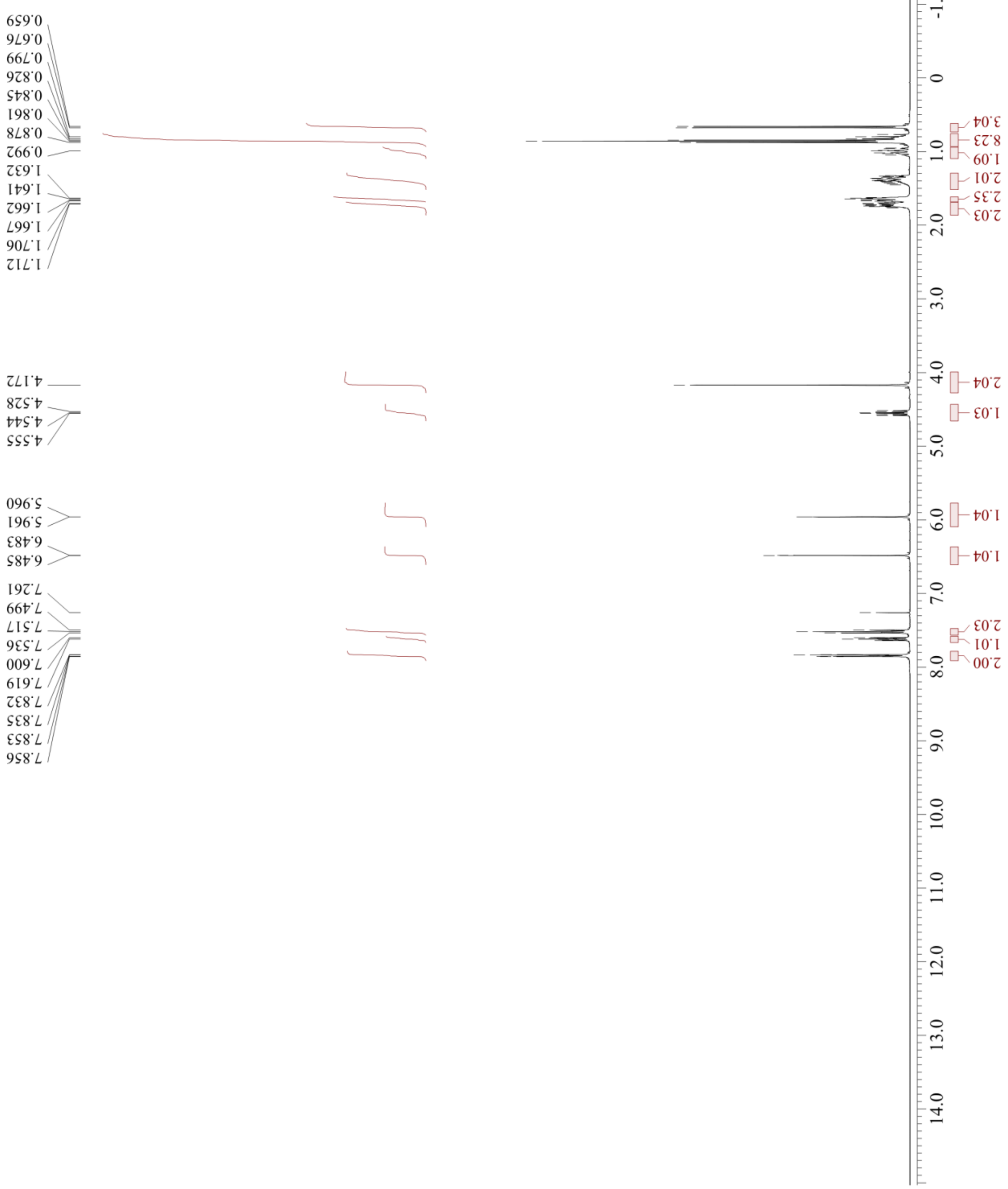

^{13}C NMR of **7ai** ($CDCl_3$, 100 MHz, 25 ºC)

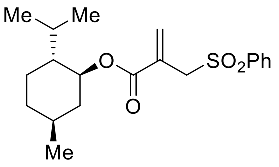

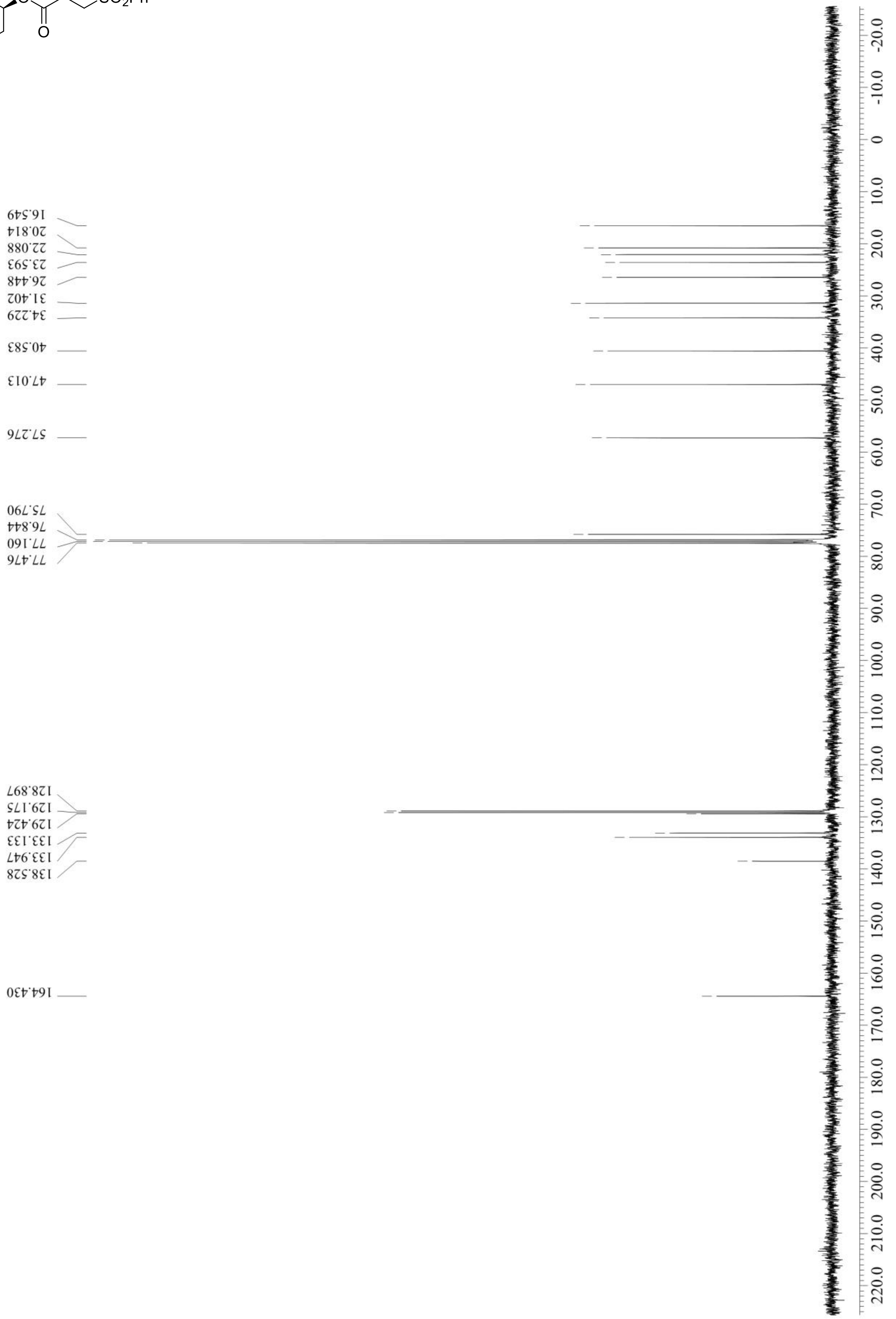

^{1}H NMR of **7aj** ($CDCl_3$, 400 MHz, 25 ºC)

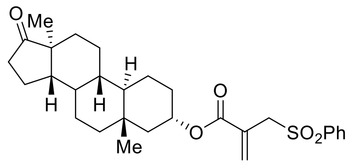

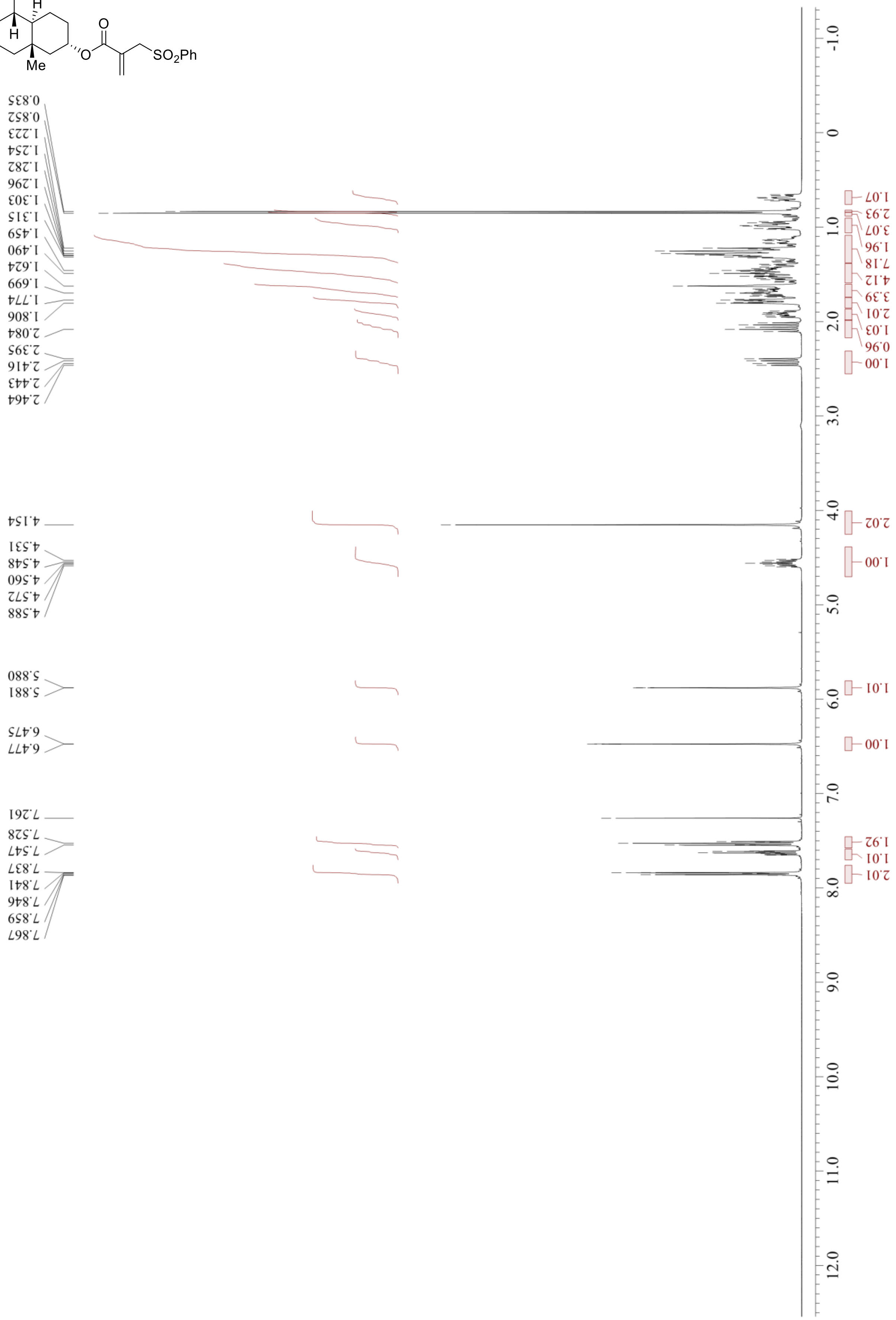

^{13}C NMR of **7aj** (CDCl$_3$, 100 MHz, 25 ºC)

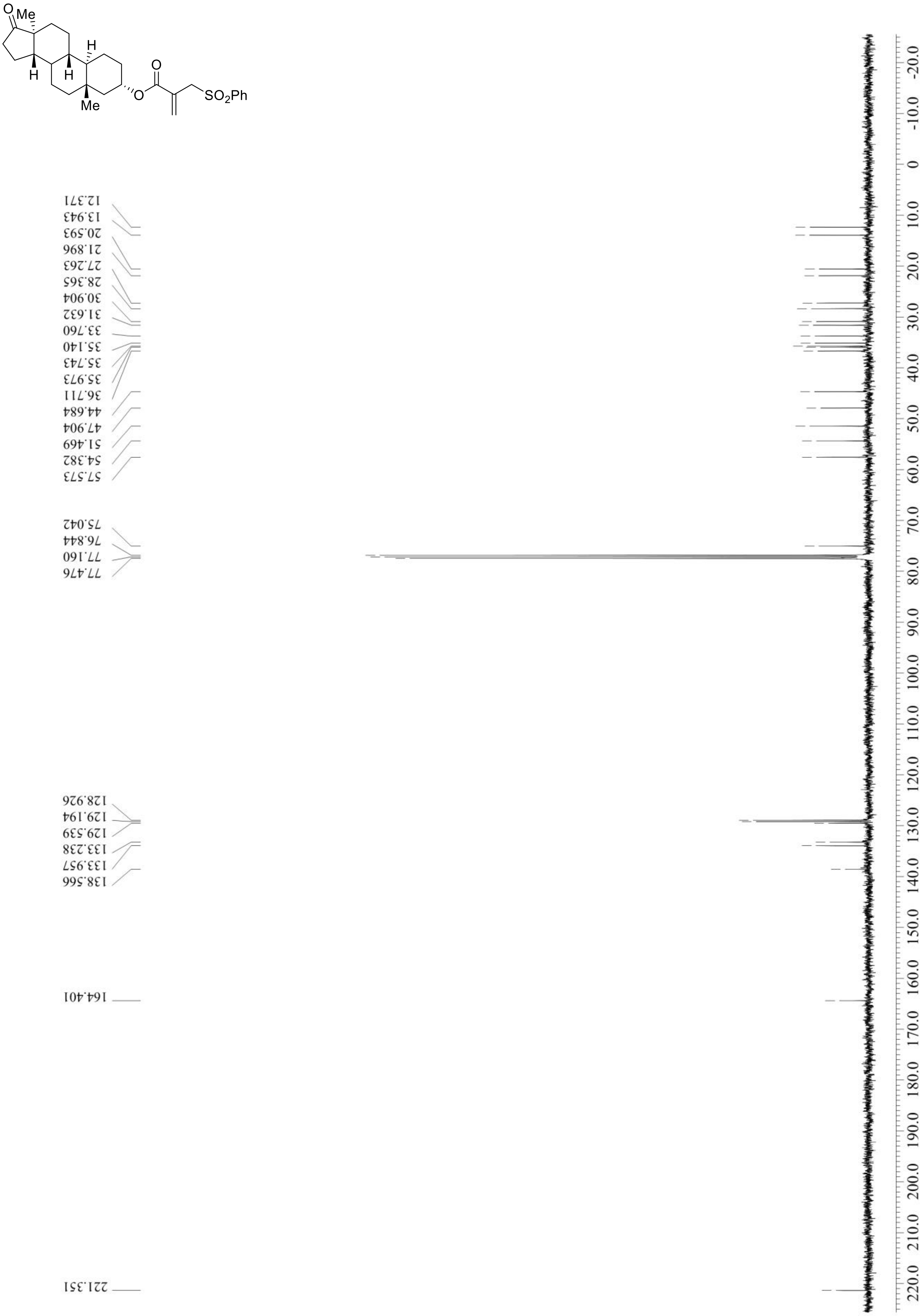

^{1}H NMR of **7al** ($CDCl_3$, 400 MHz, 25 ºC)

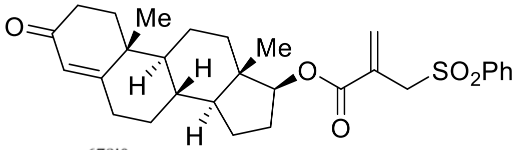

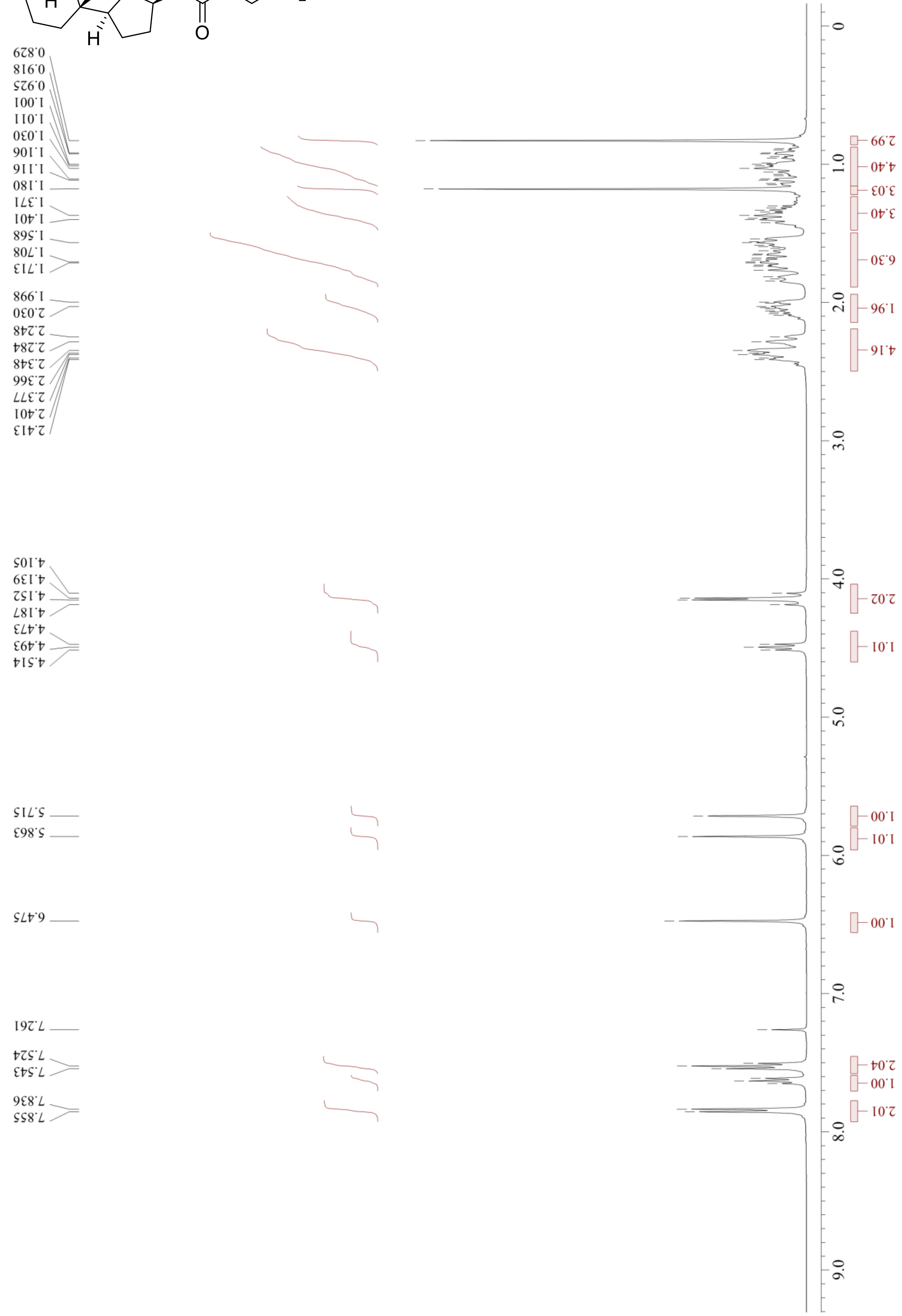

^{13}C NMR of **7al** ($CDCl_3$, 100 MHz, 25 °C)

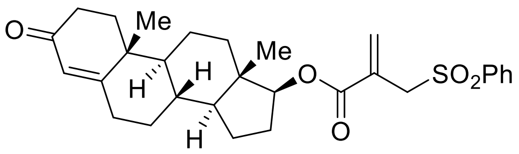

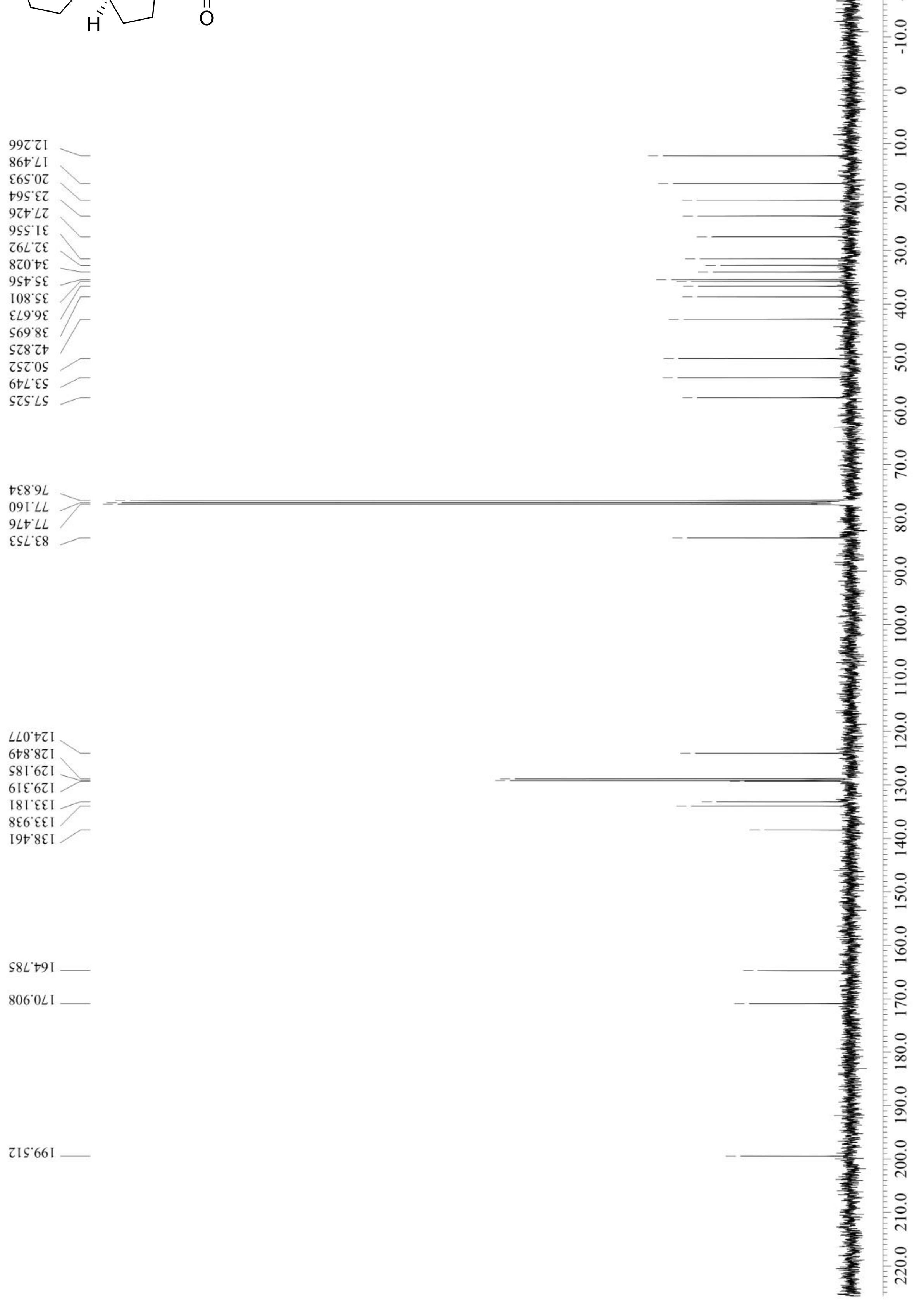

^{1}H NMR of **7an** ($CDCl_3$, 400 MHz, 25 ºC)

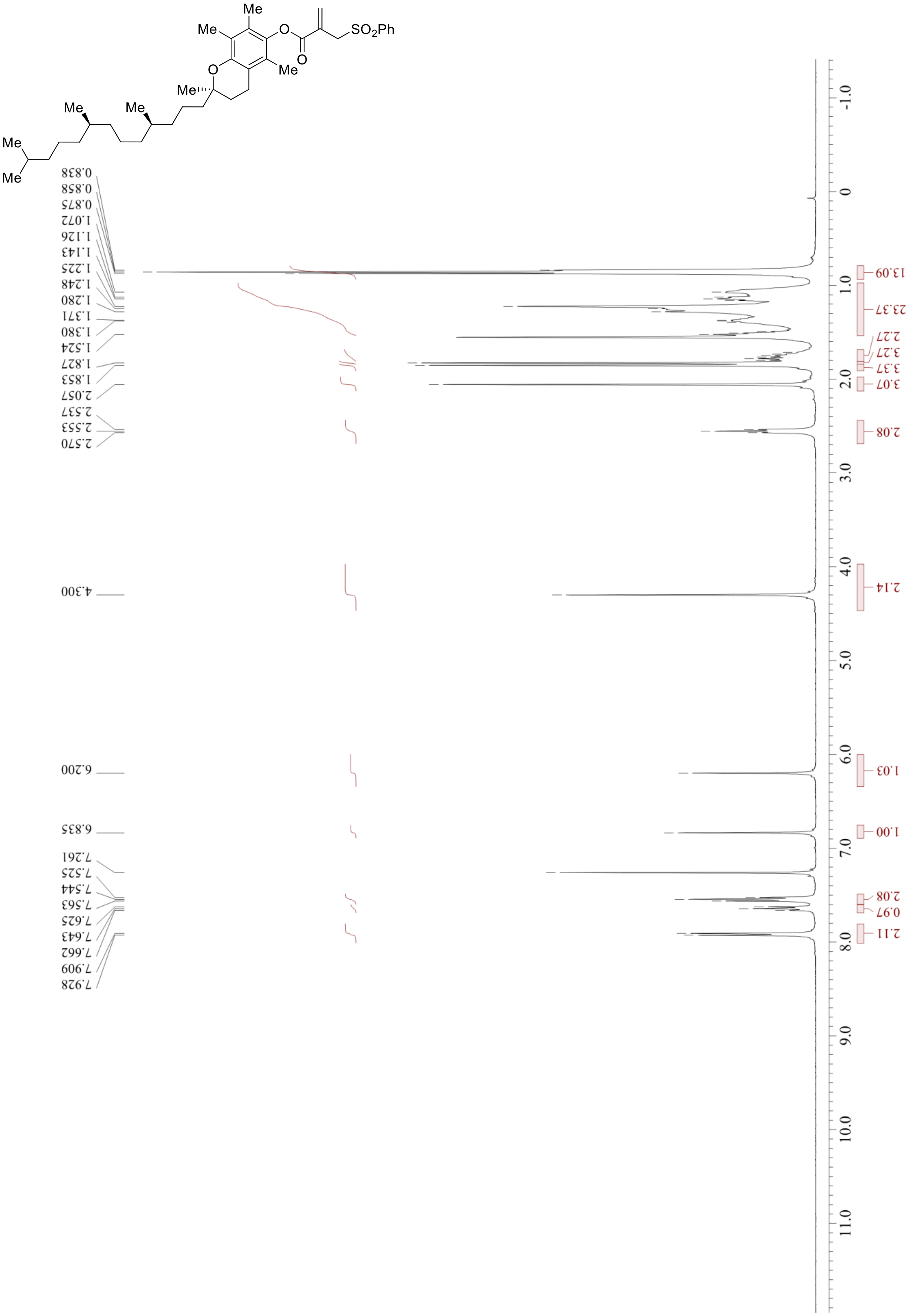

^{13}C NMR of **7an** ($CDCl_3$, 100 MHz, 25 ºC)

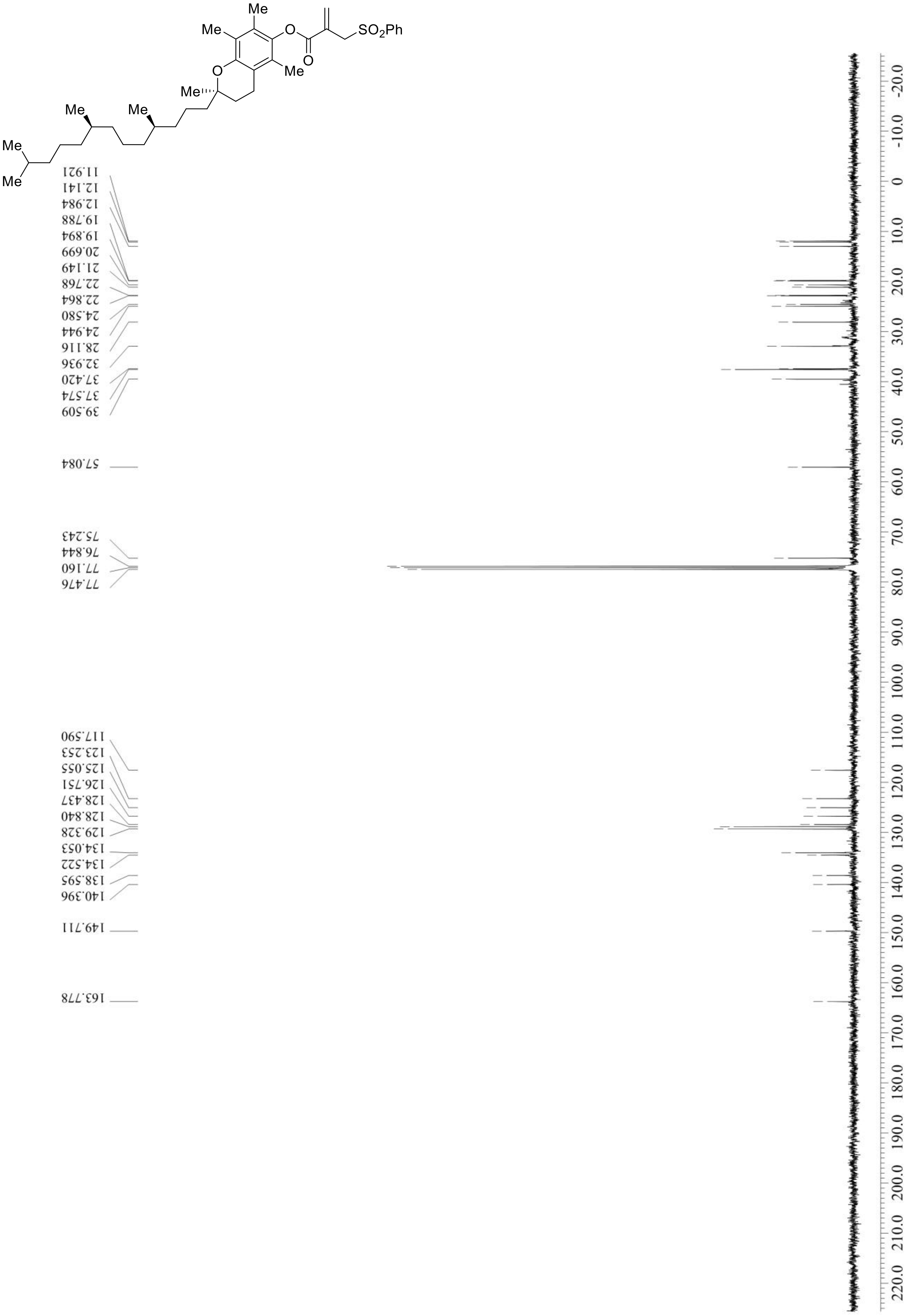

^{1}H NMR of **7ao** ($CDCl_3$, 400 MHz, 25 °C)

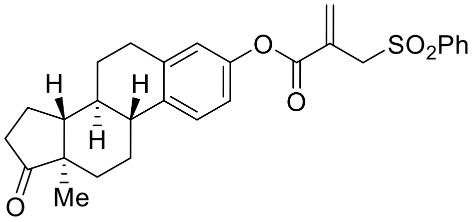

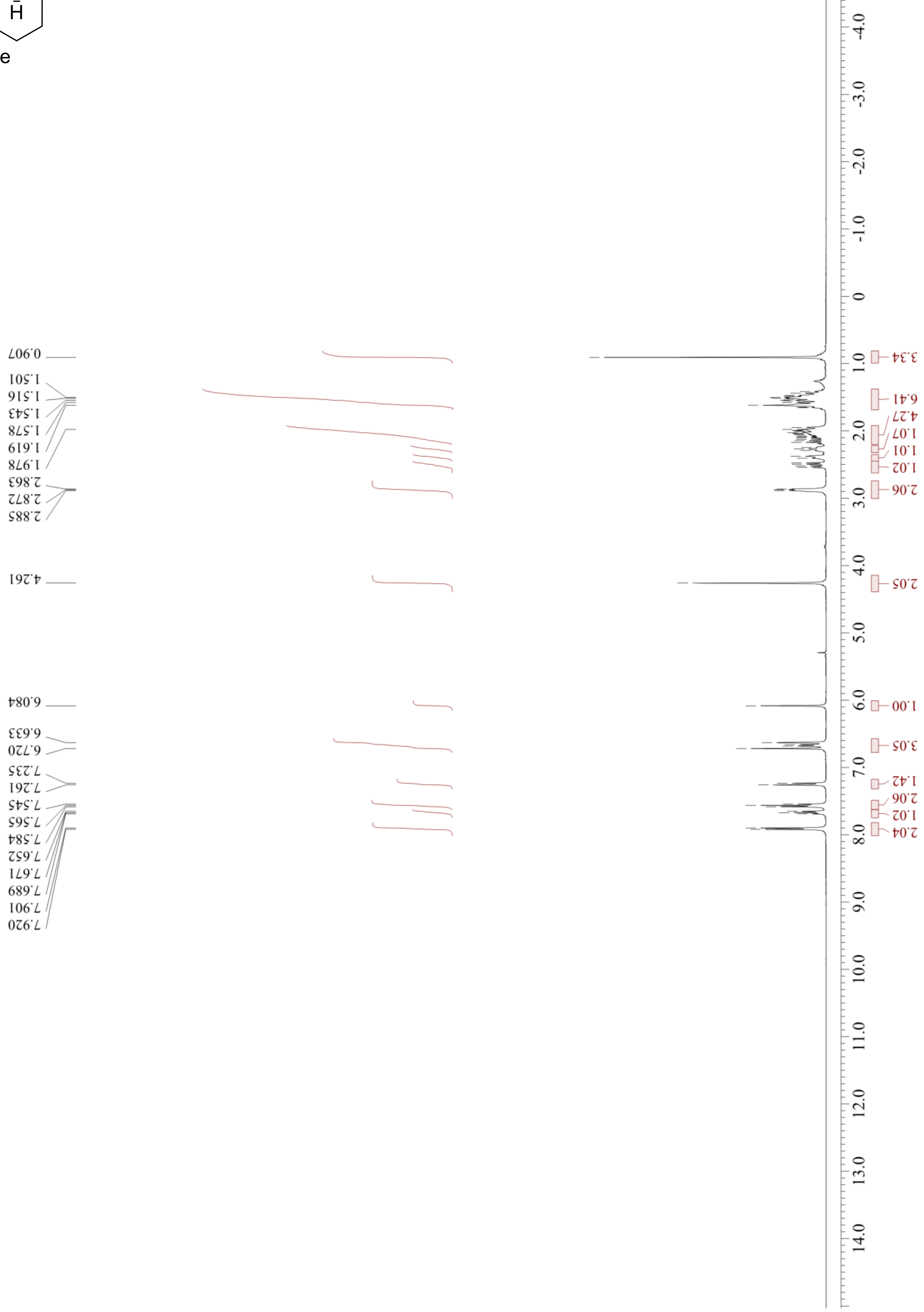

^{13}C NMR of **7ao** ($CDCl_3$, 100 MHz, 25 ºC)

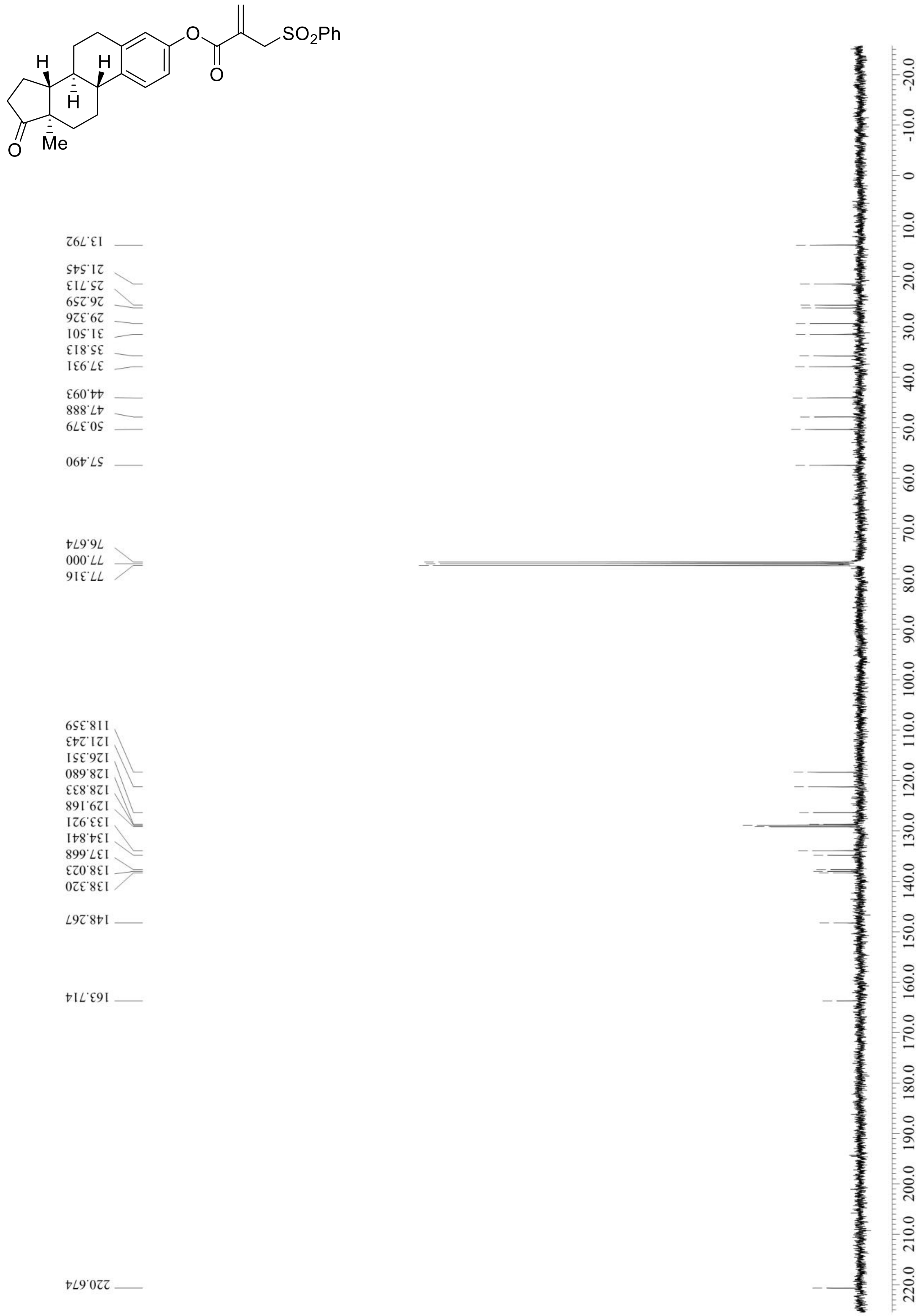

^{1}H NMR of **7ab** ($CDCl_3$, 400 MHz, 25 ºC)

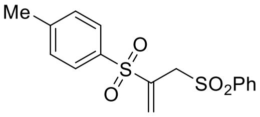

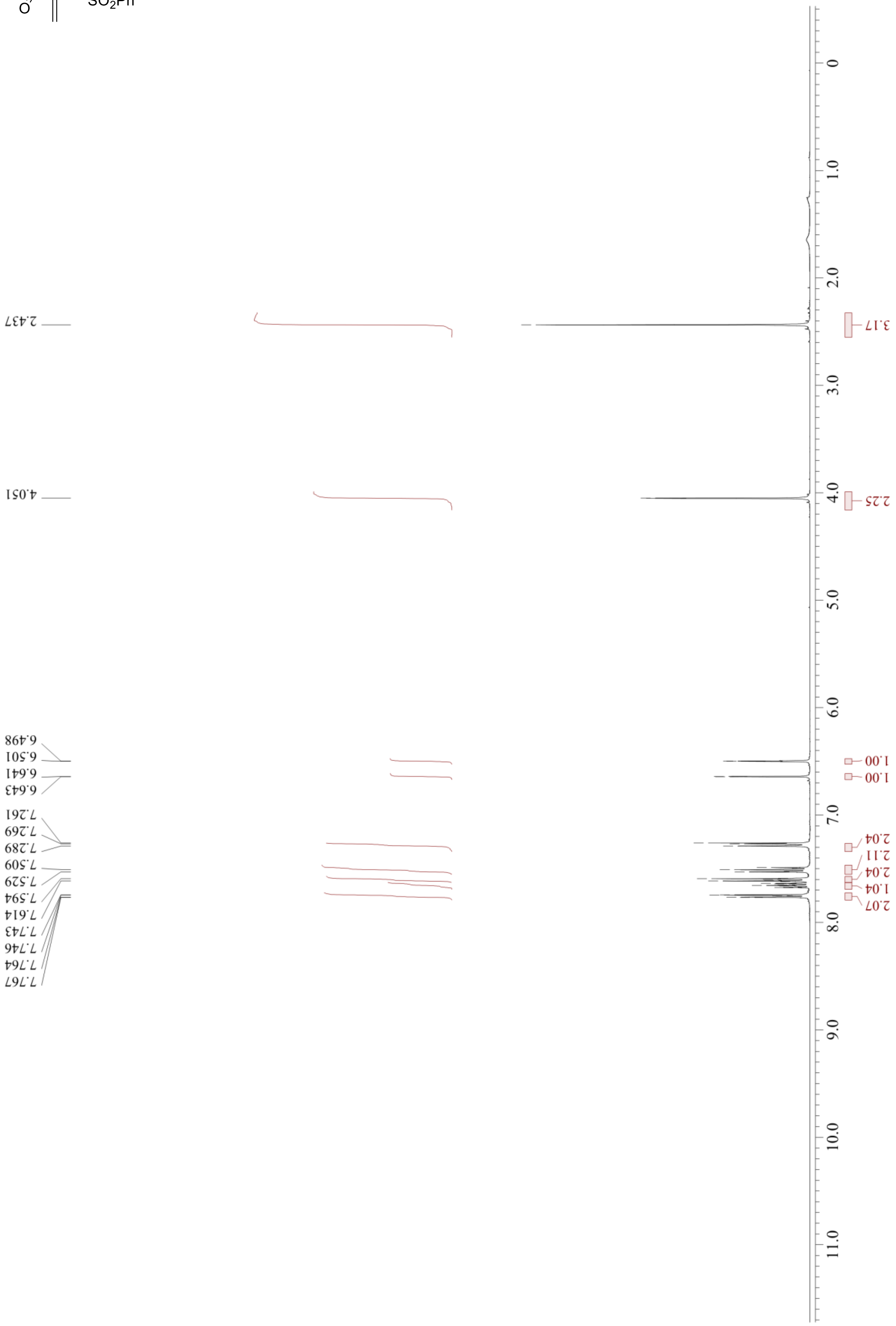

^{13}C NMR of **7ab** ($CDCl_3$, 100 MHz, 25 ºC)

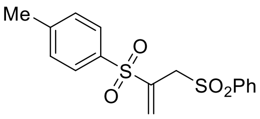

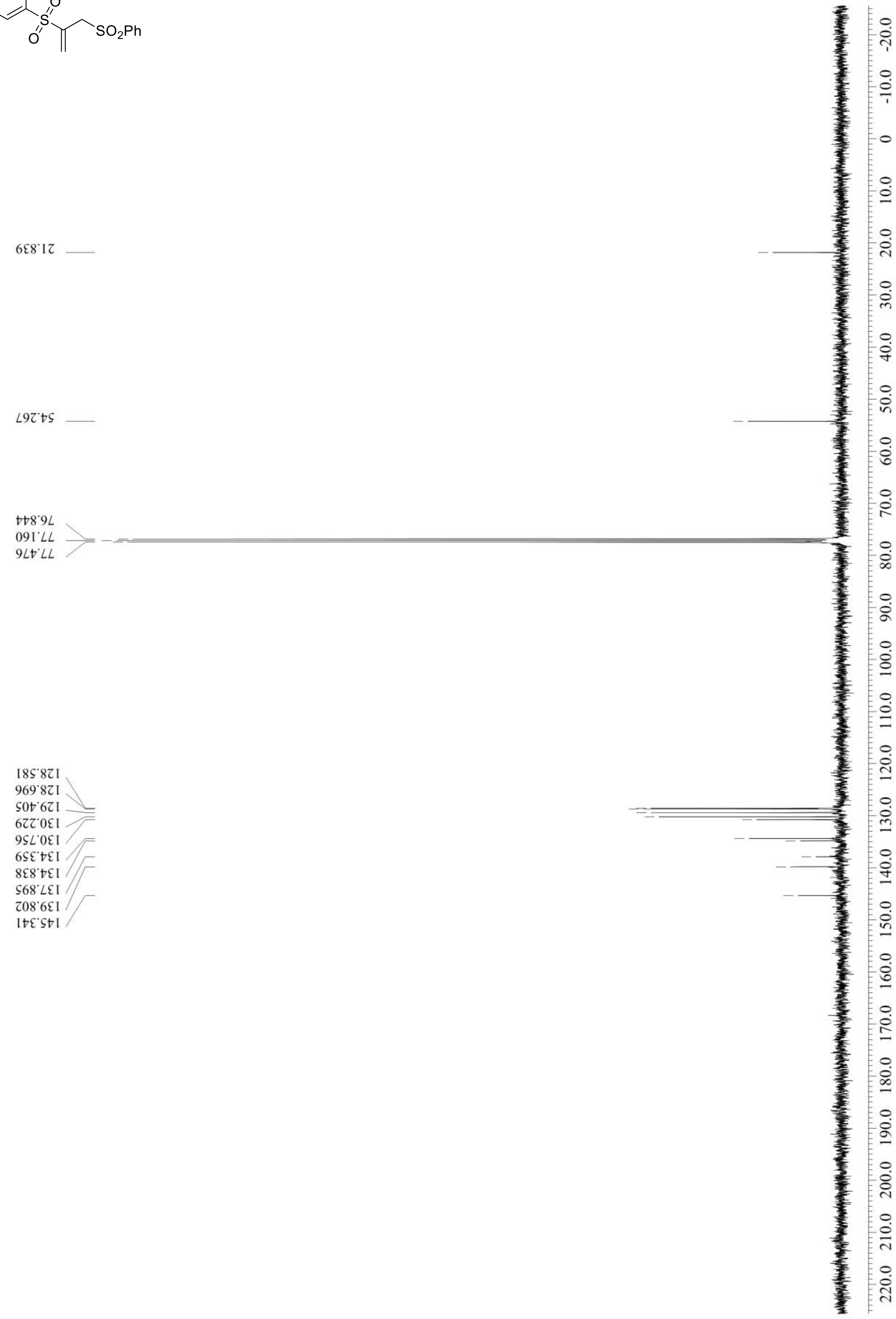

^{1}H NMR of **7ad** ($CDCl_3$, 400 MHz, 25 ºC)

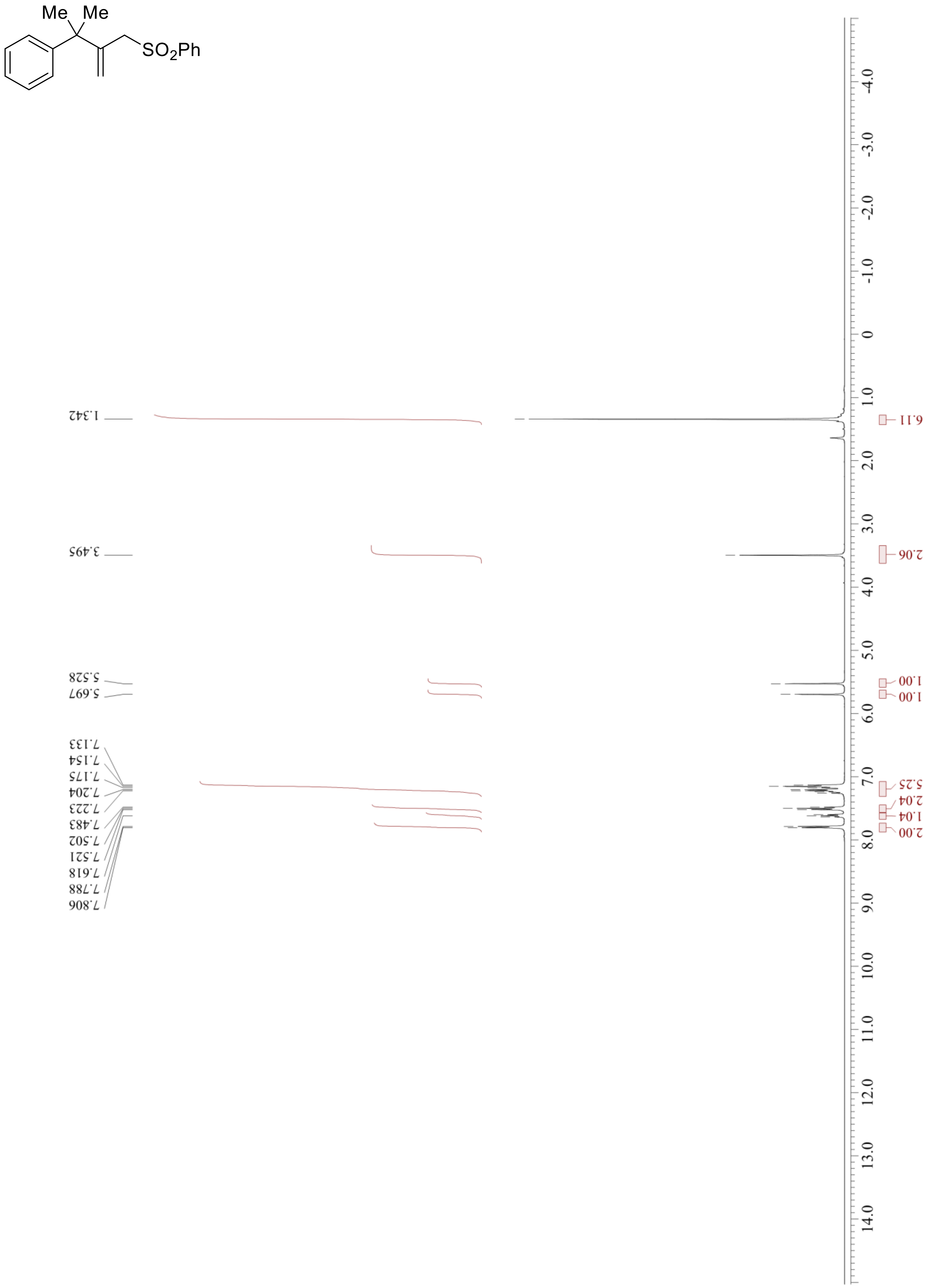

^{13}C NMR of **7ad** ($CDCl_3$, 100 MHz, 25 ºC)

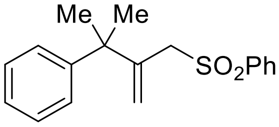

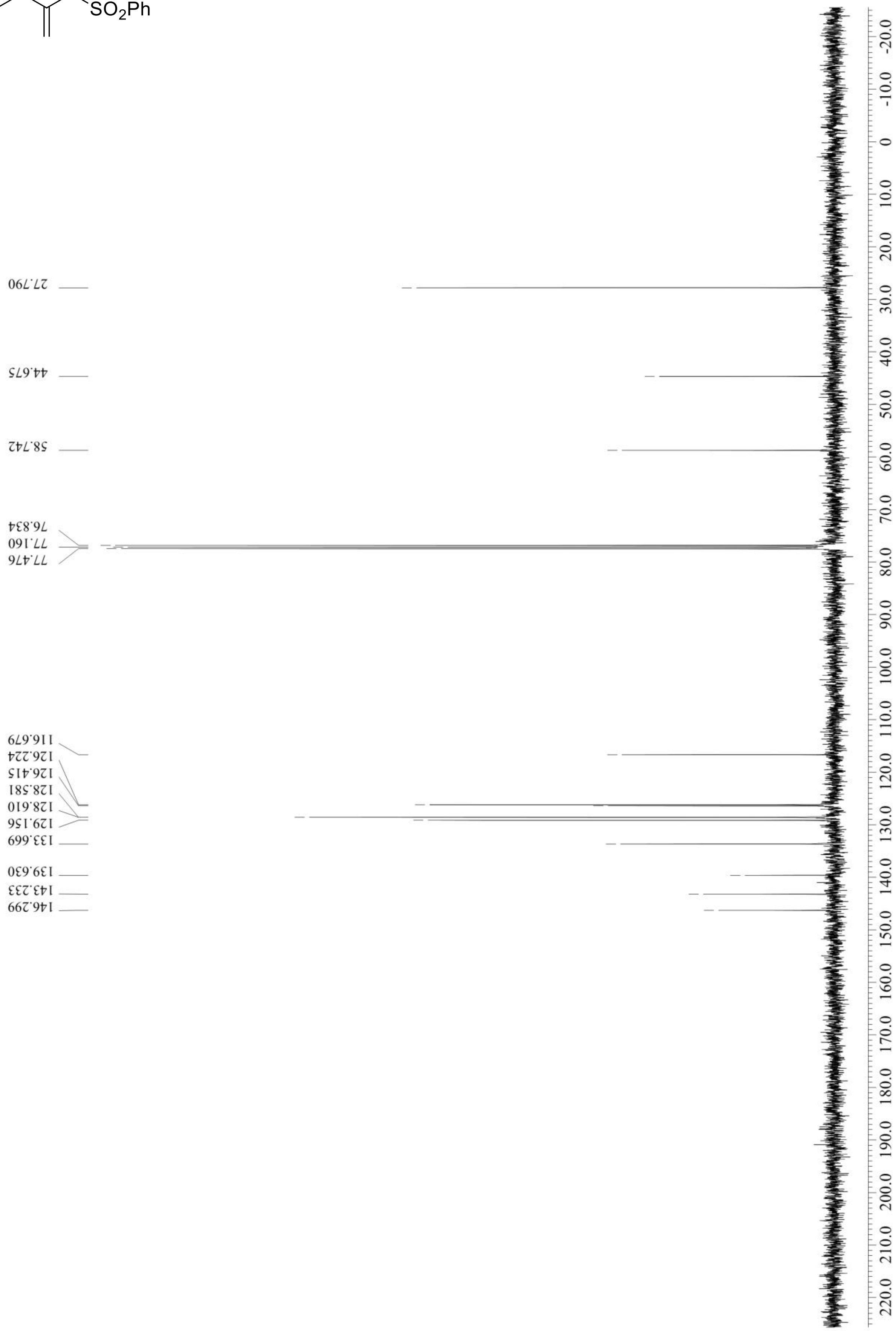

^{1}H NMR of **7af** ($CDCl_3$, 400 MHz, 25 ºC)

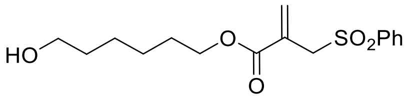

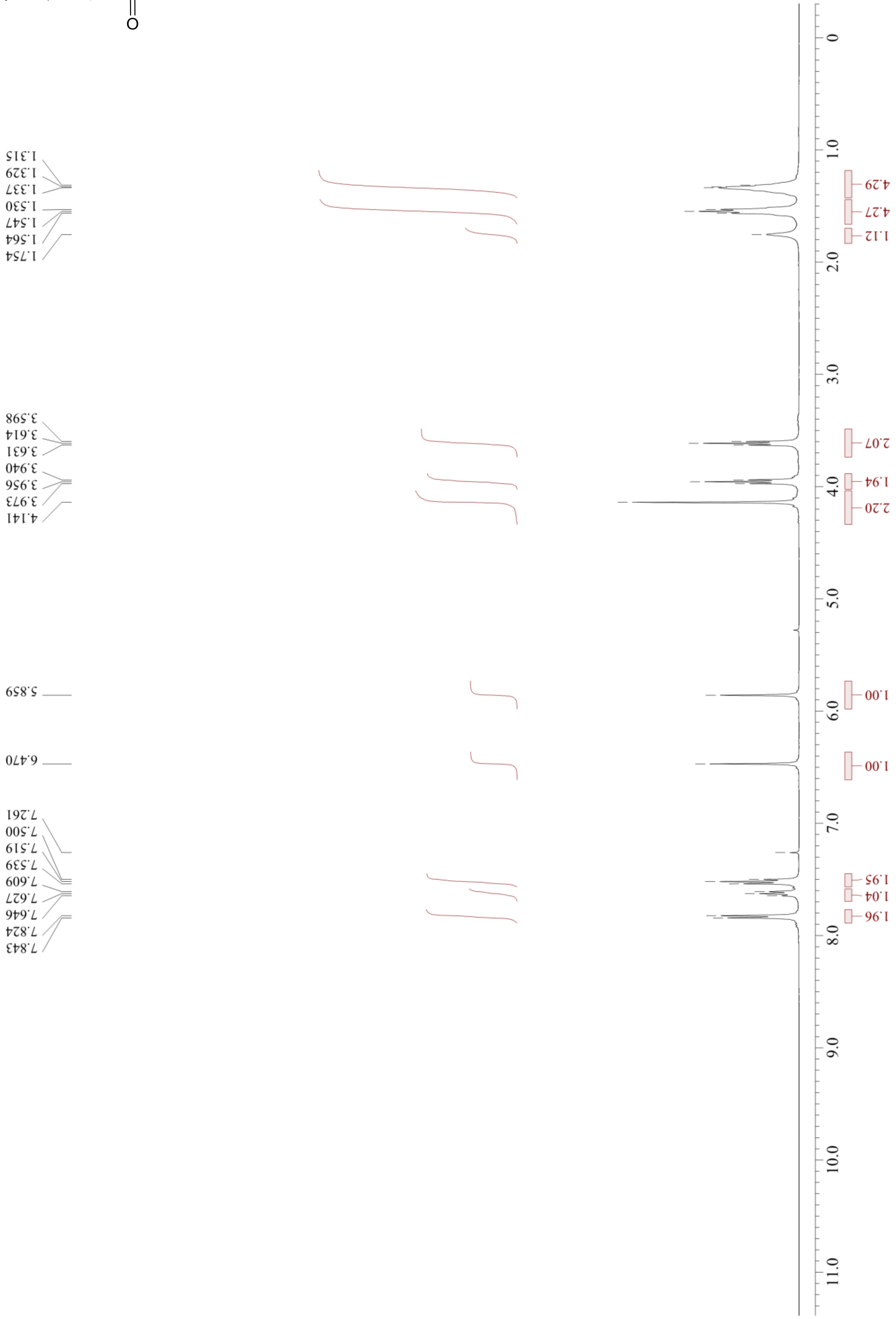

^{13}C NMR of **7af** ($CDCl_3$, 100 MHz, 25 ºC)

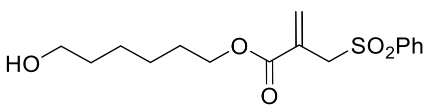

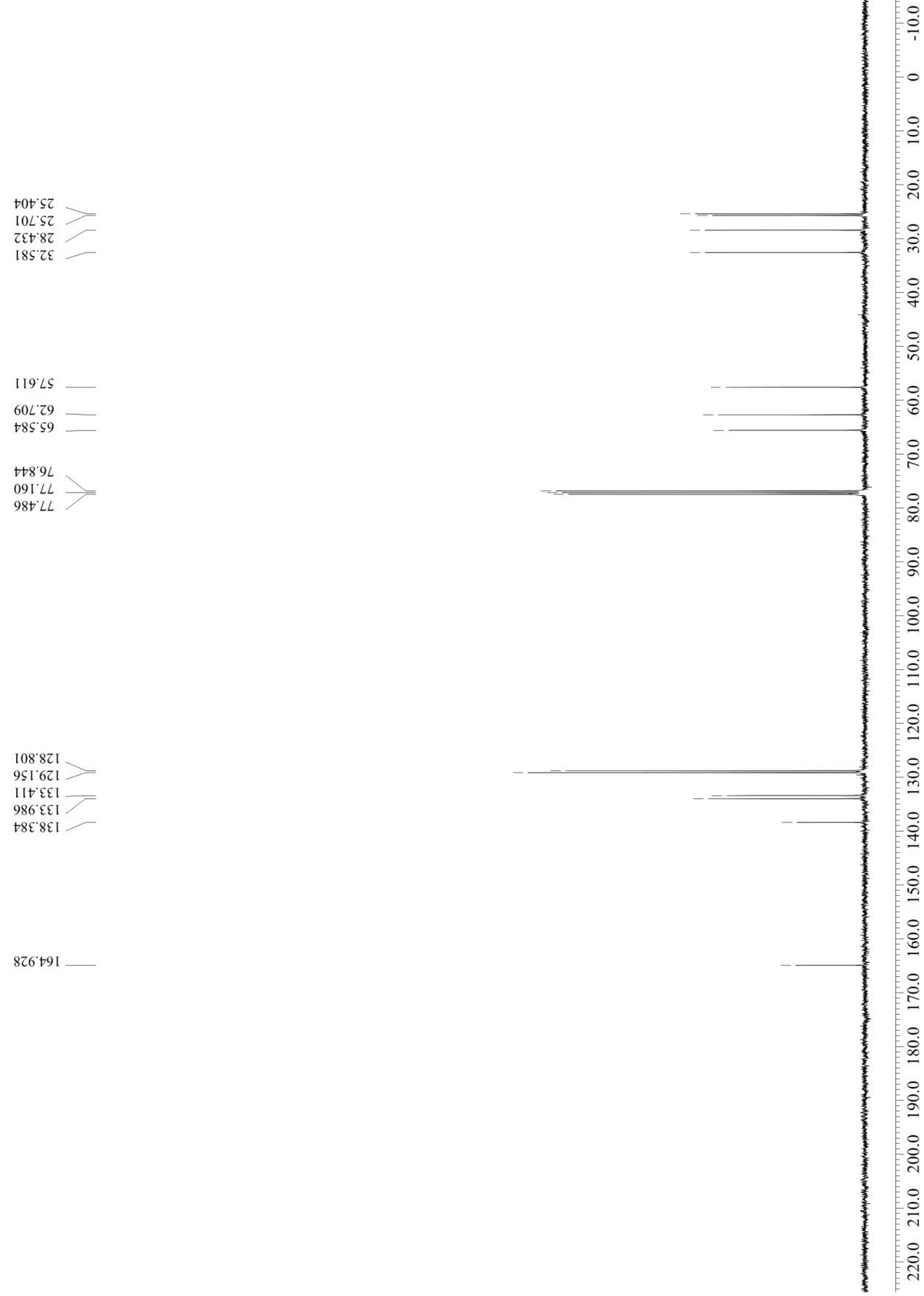

^{1}H NMR of **7ag** ($CDCl_3$, 400 MHz, 25 ºC)

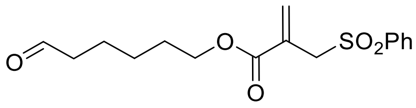

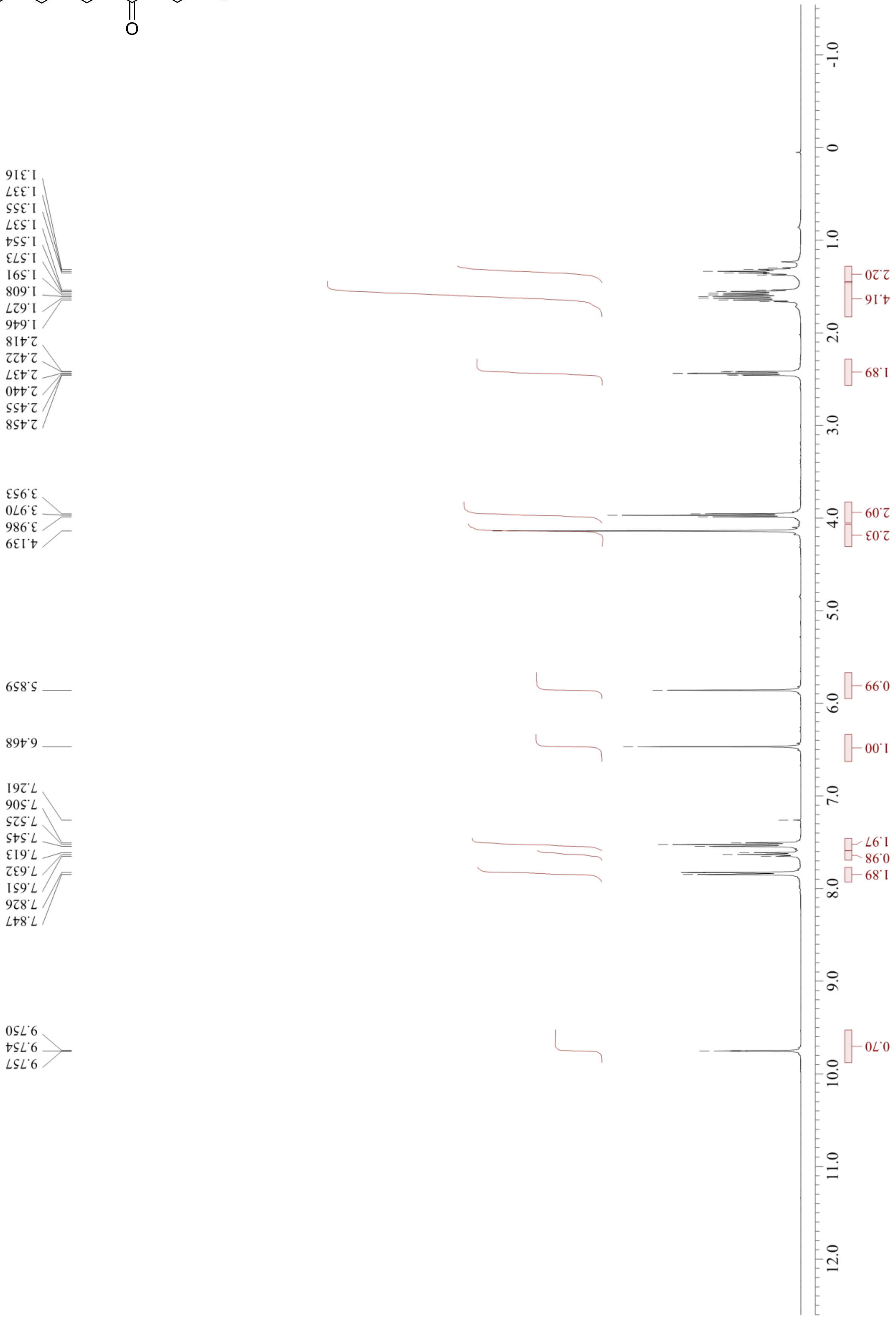

^{13}C NMR of **7ag** ($CDCl_3$, 100 MHz, 25 ºC)

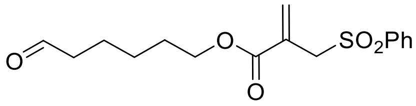

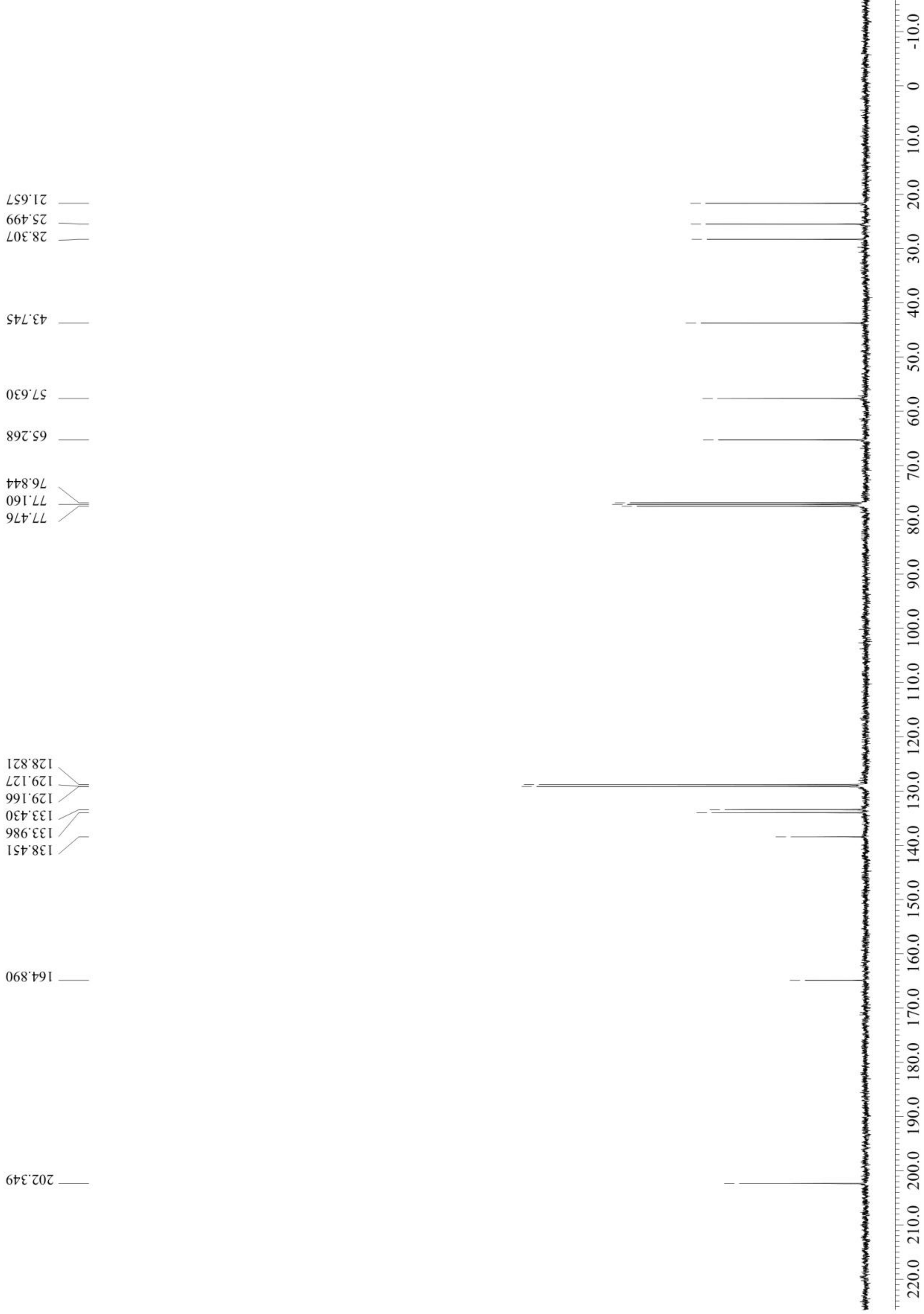

^{1}H NMR of **8a** ($CDCl_3$, 400 MHz, 25 ºC)

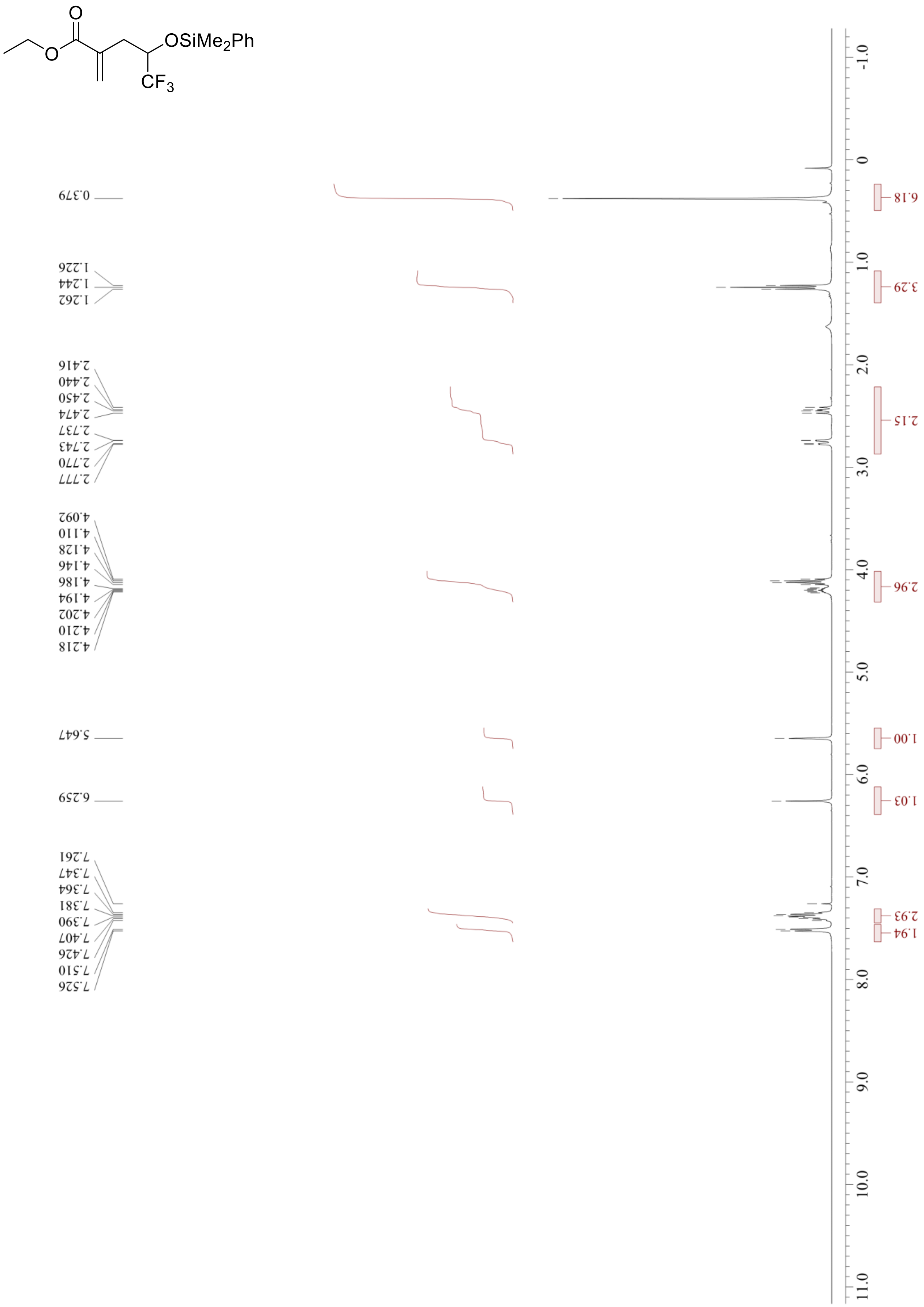

^{13}C NMR of **8a** ($CDCl_3$, 100 MHz, 25 ºC)

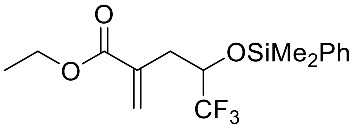

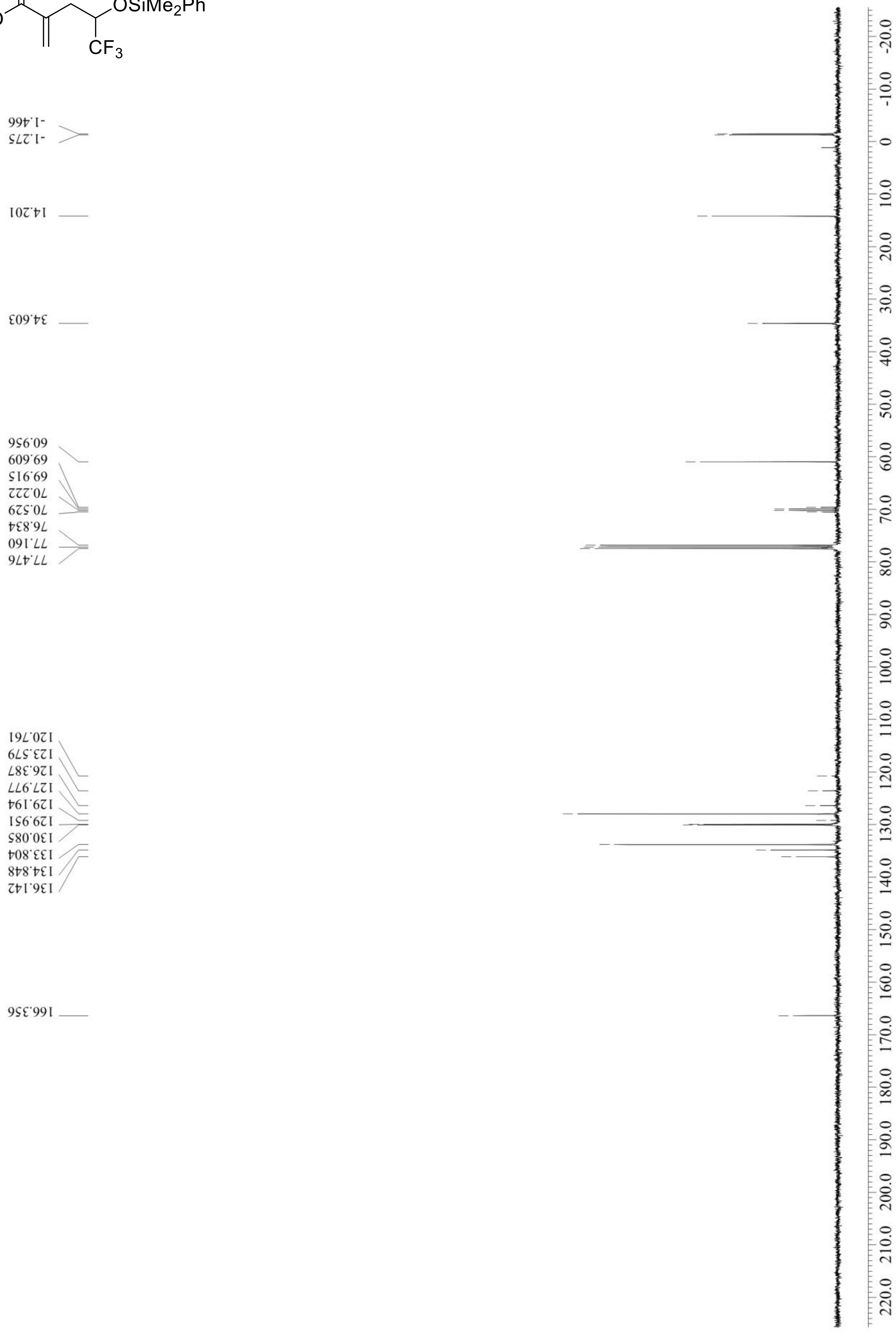

^{19}F NMR of **8a** ($CDCl_3$, 375 MHz, 25 ºC)

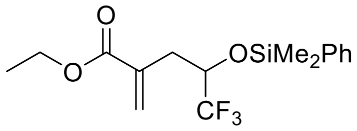

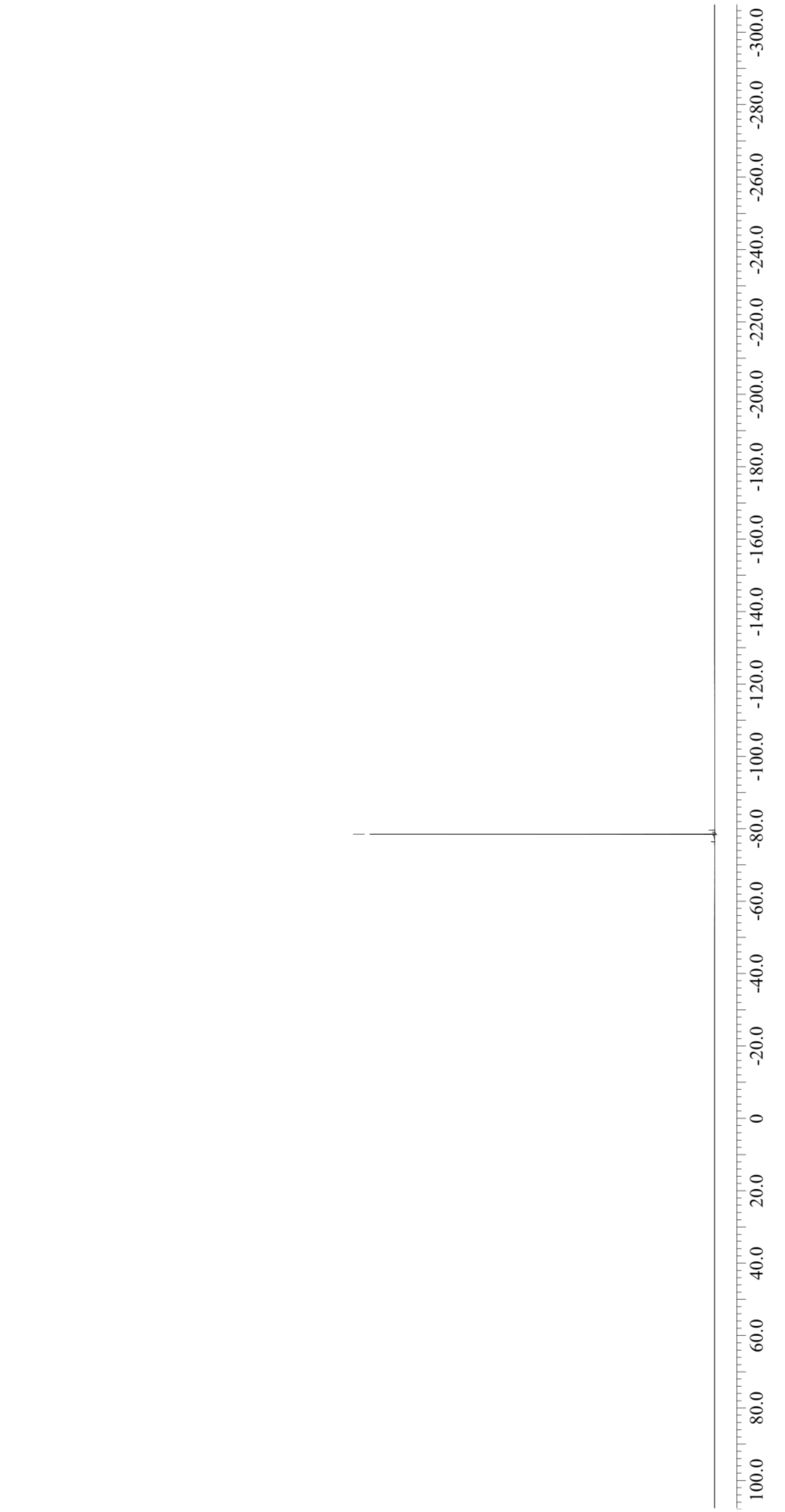

^{1}H NMR of **9b'** ($CDCl_3$, 400 MHz, 25 ºC)

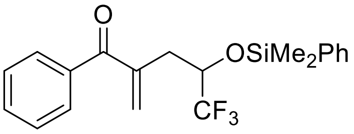

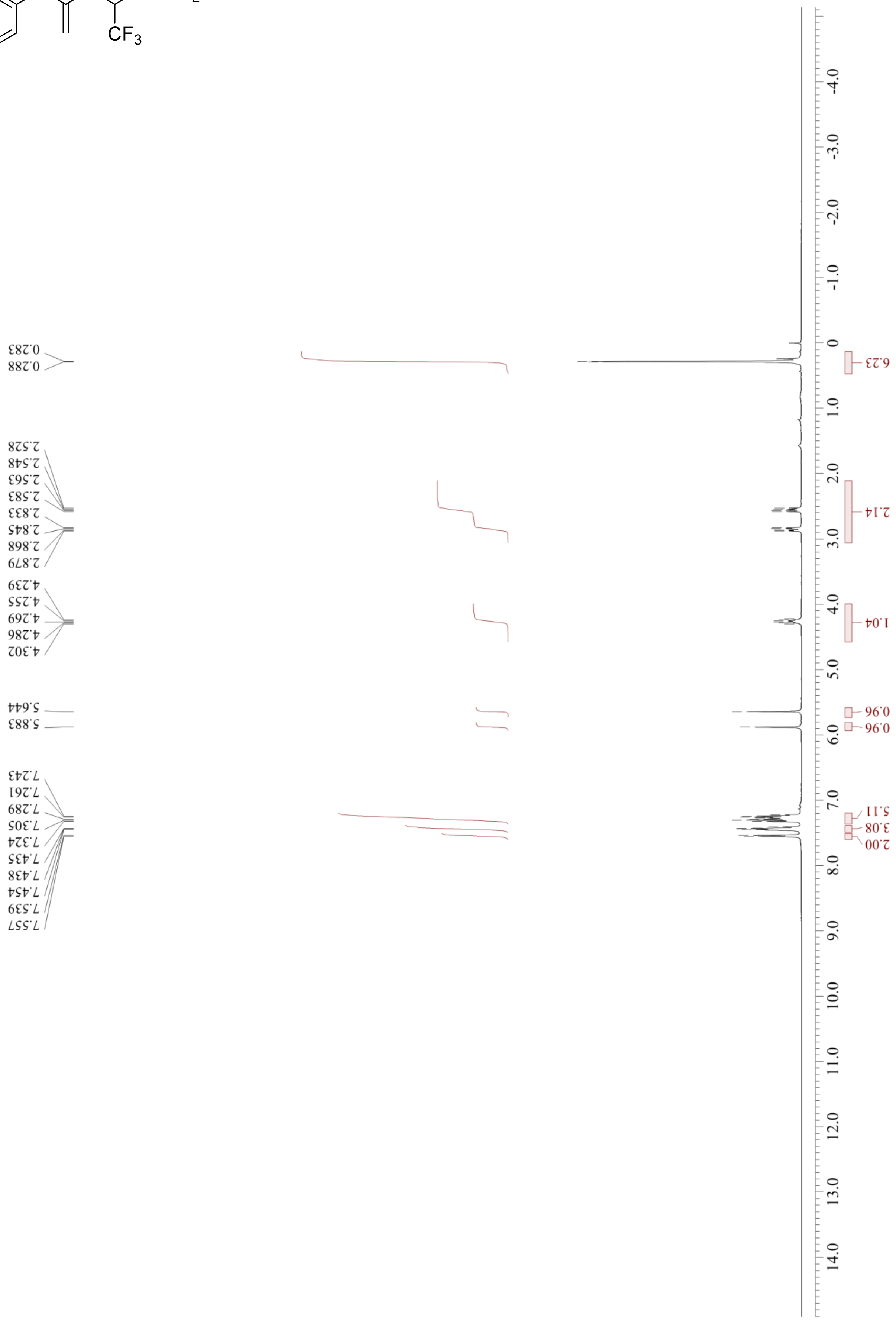

^{13}C NMR of **9b’** (CDCl$_3$, 100 MHz, 25 ºC)

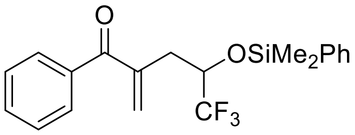

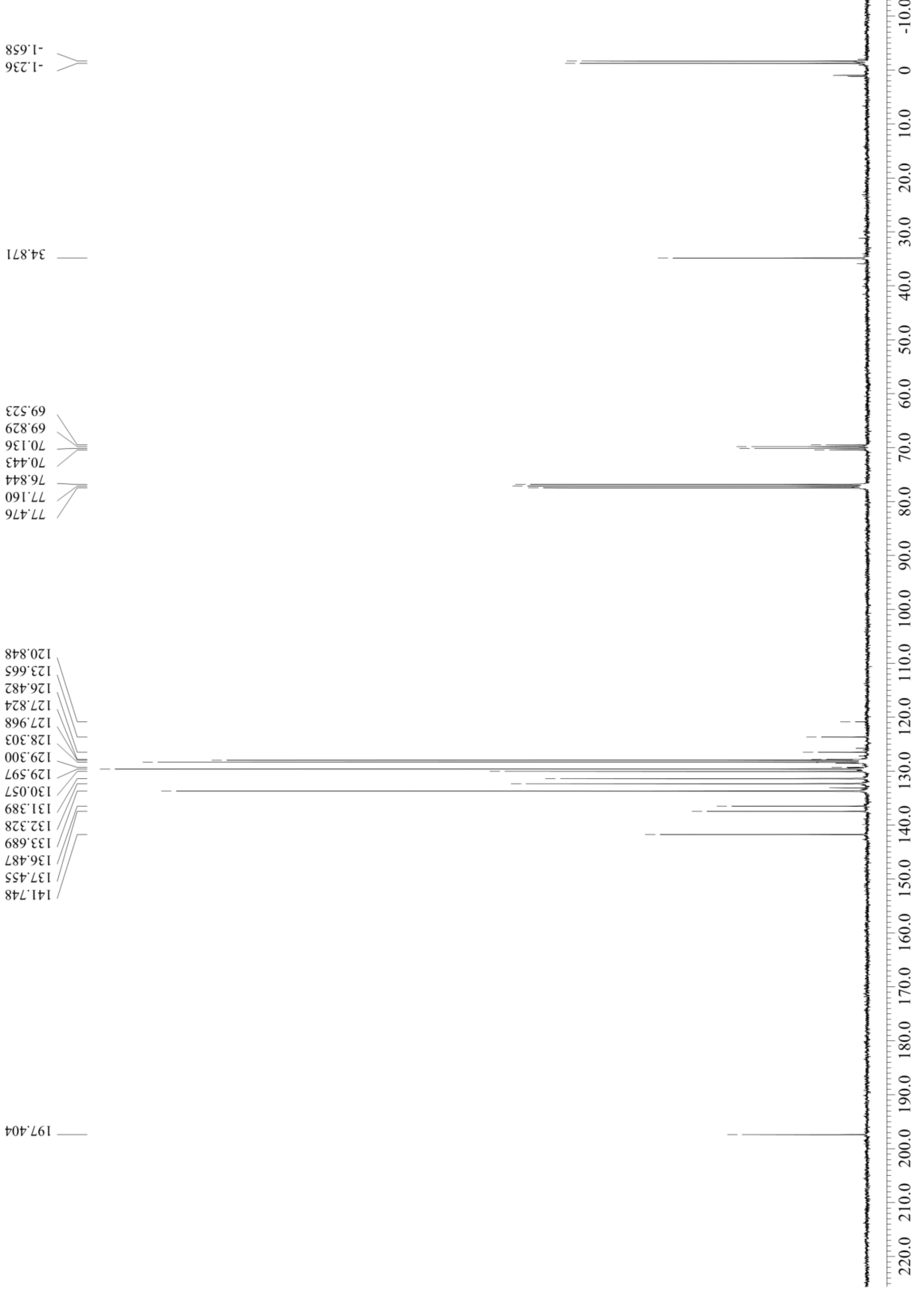

^{19}F NMR of **9b'** ($CDCl_3$, 100 MHz, 25 ºC)

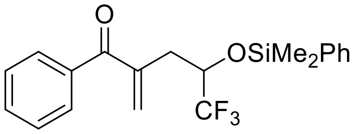

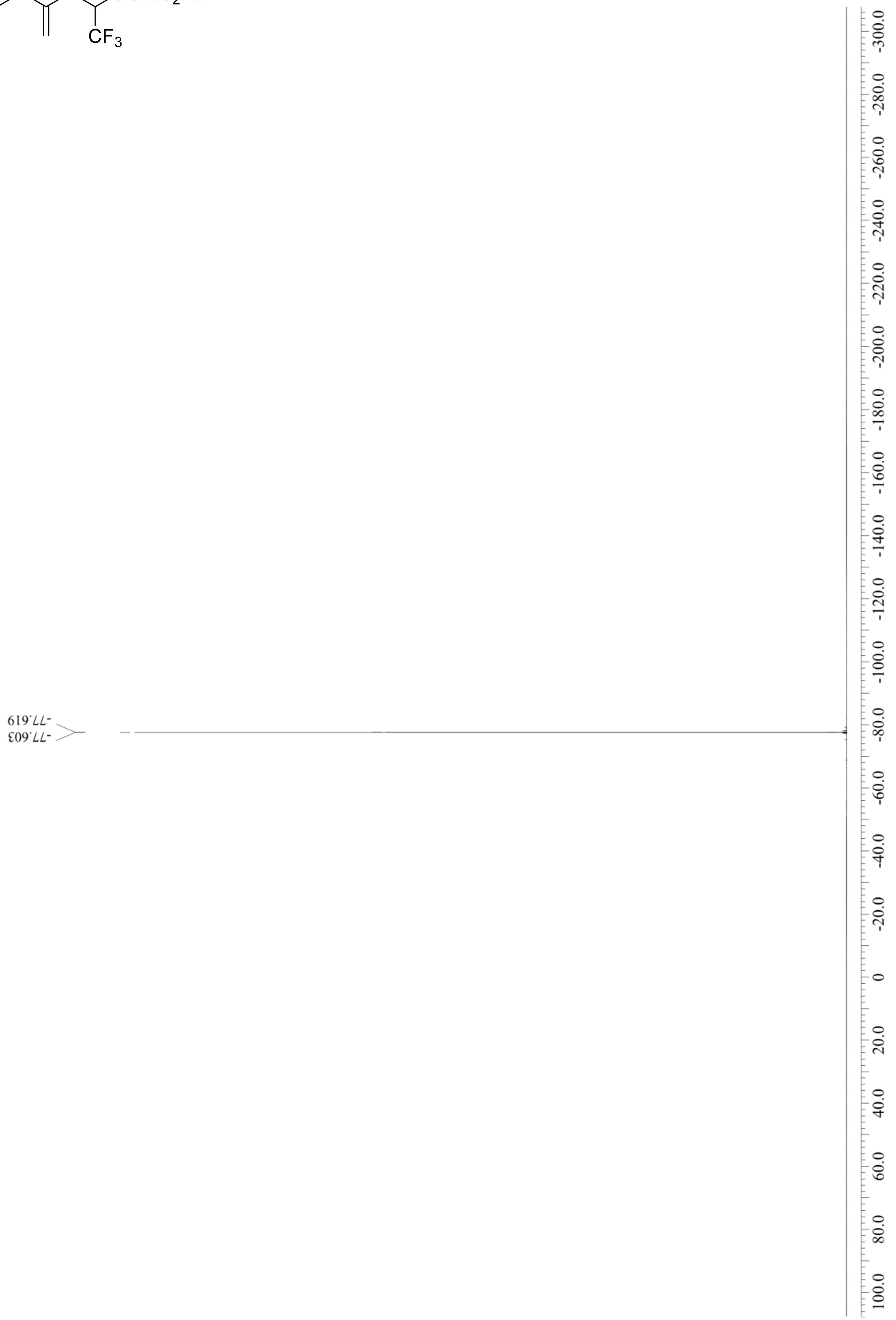

^{1}H NMR of **9a** ($CDCl_3$, 400 MHz, 25 °C)

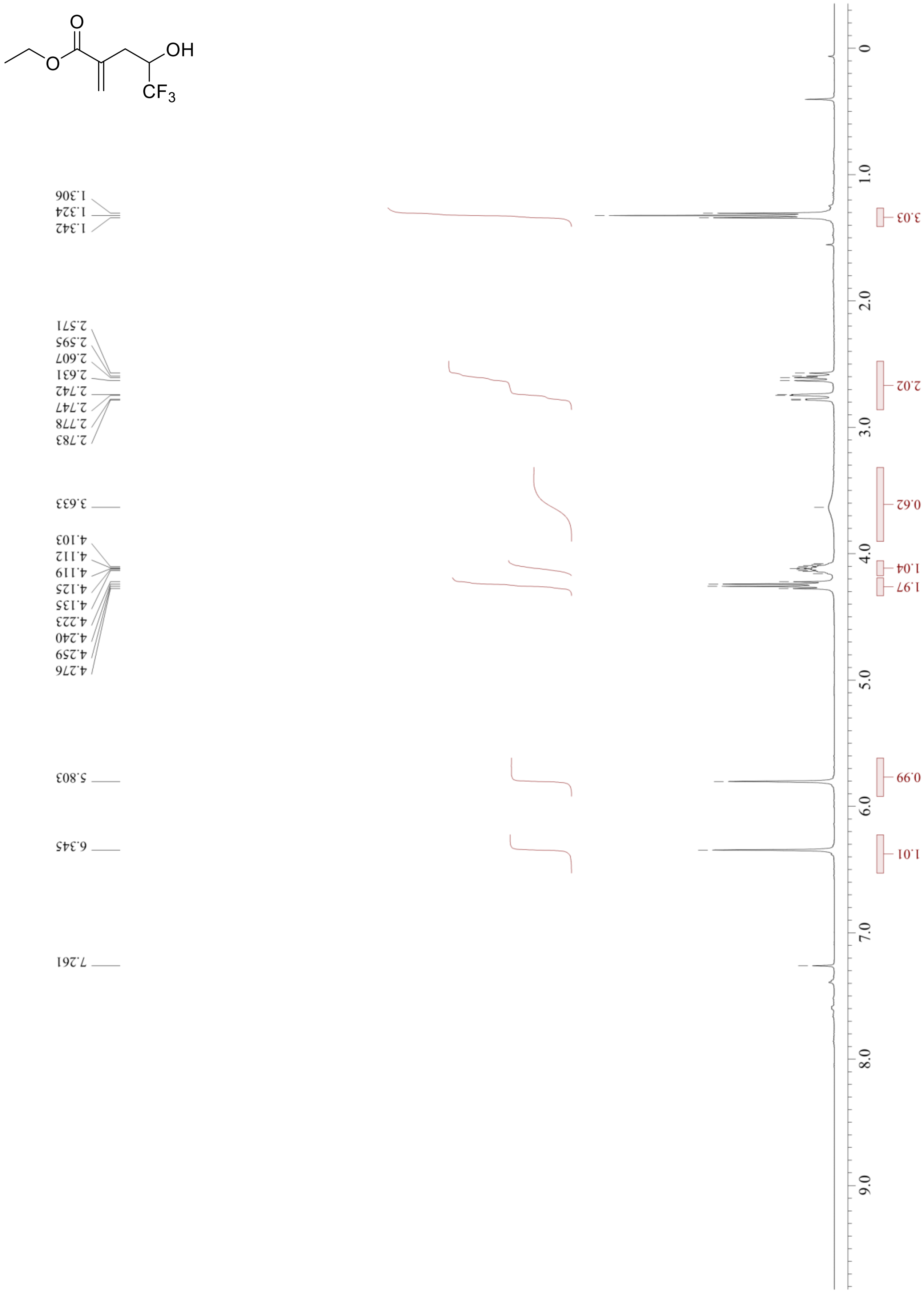

^{13}C NMR of **9a** ($CDCl_3$, 100 MHz, 25 ºC)

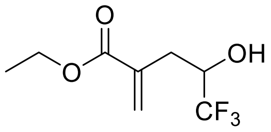

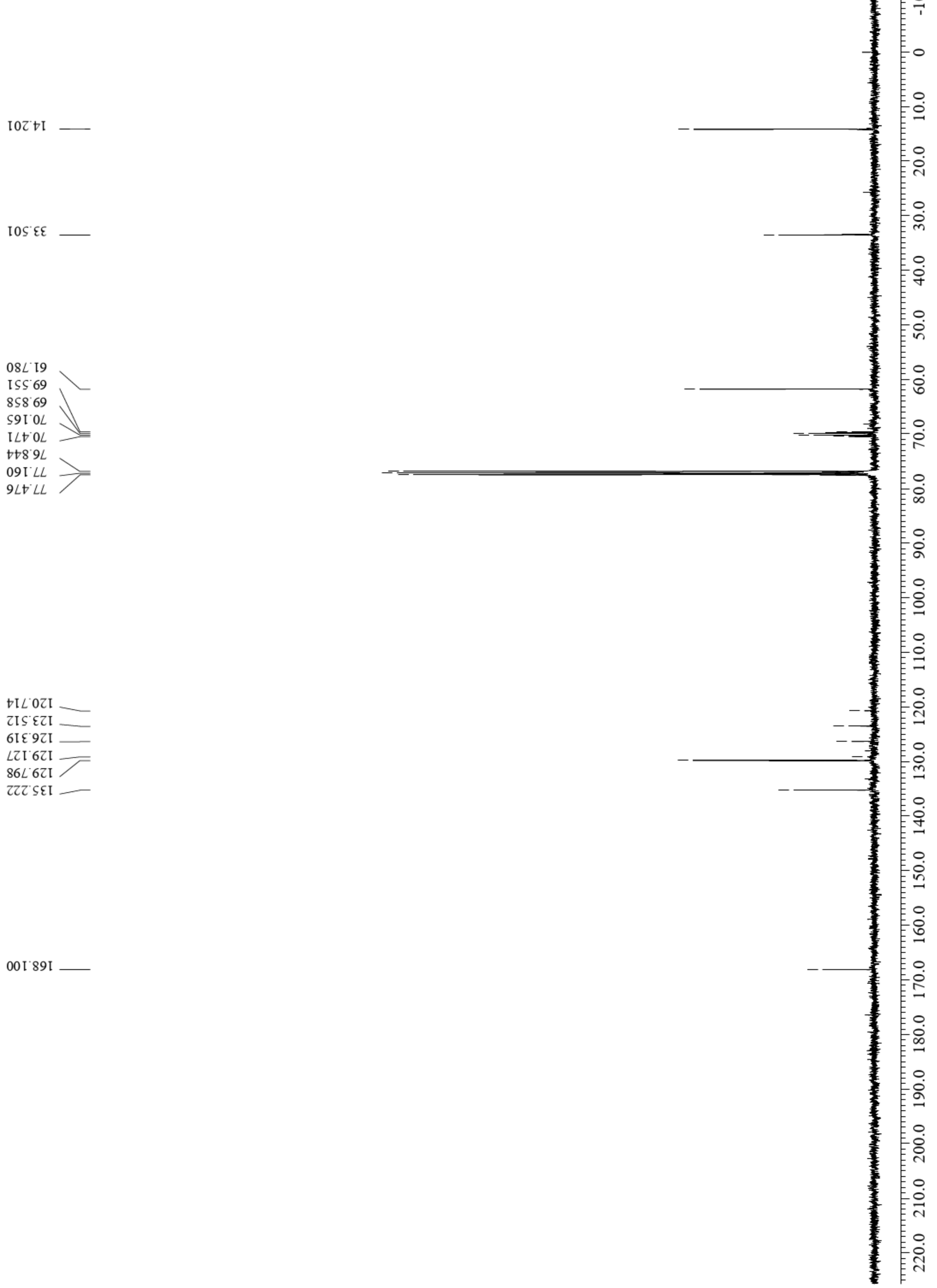

^{19}F NMR of **9a** ($CDCl_3$, 375 MHz, 25 ºC)

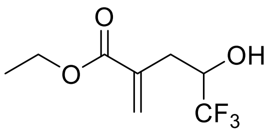

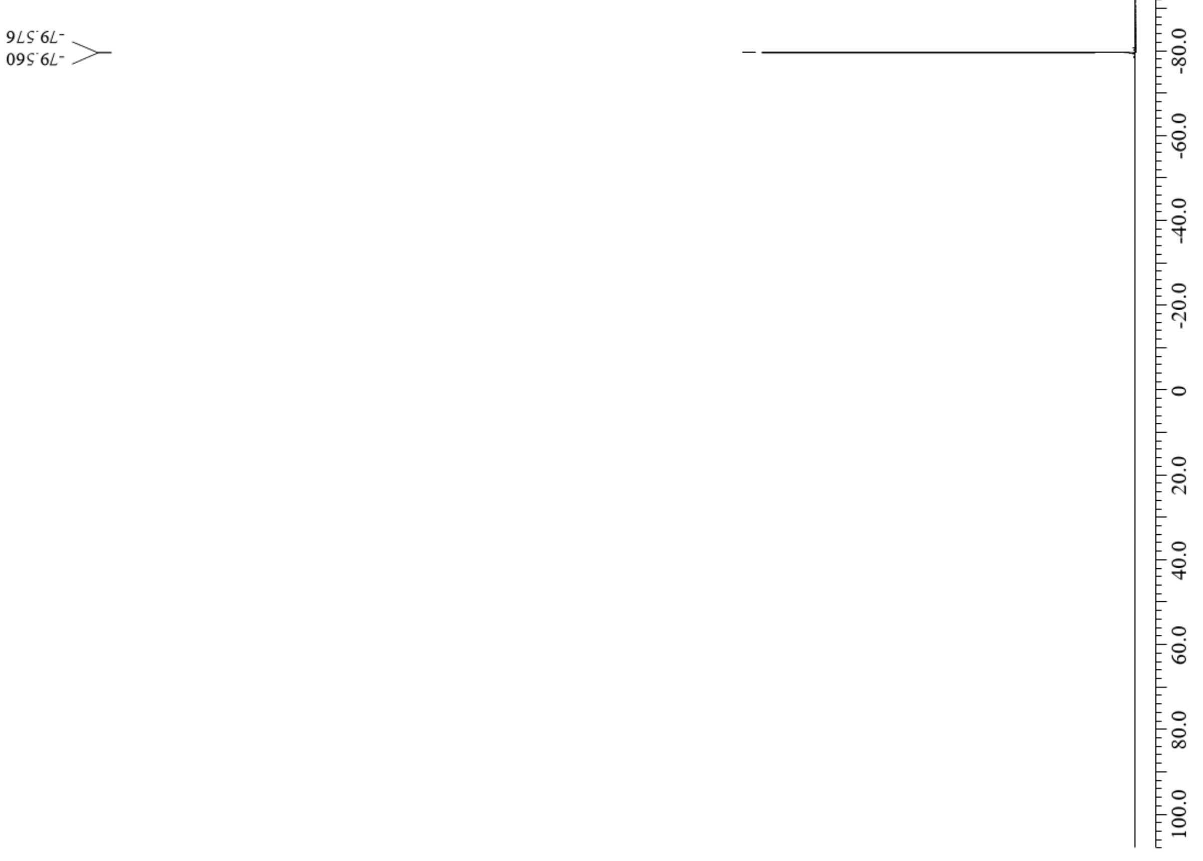

^{1}H NMR of **9b** ($CDCl_3$, 400 MHz, 25 °C)

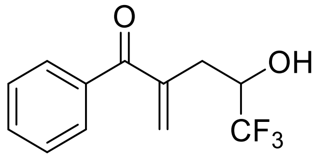

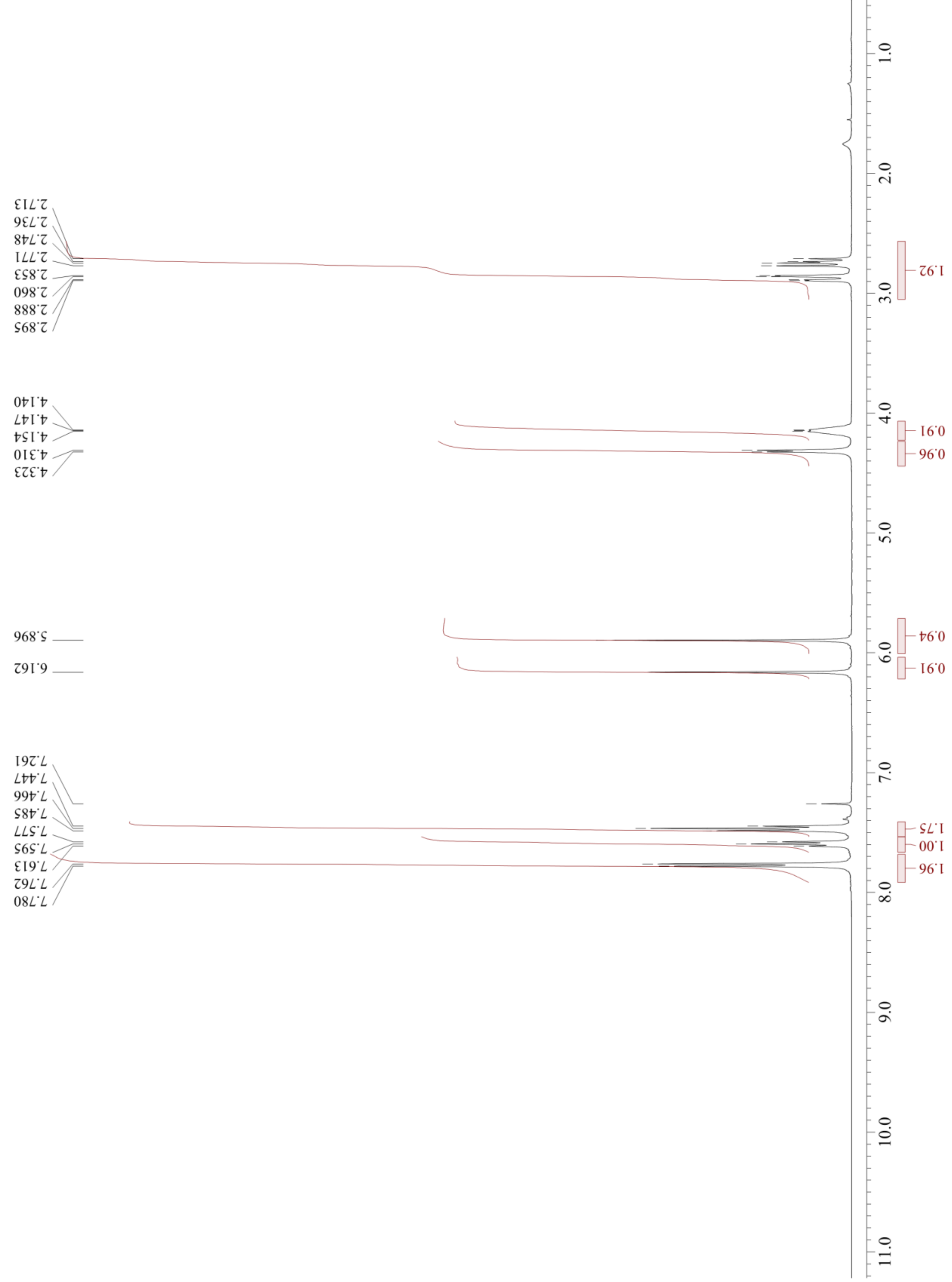

^{13}C NMR of **9b** ($CDCl_3$, 100 MHz, 25 ºC)

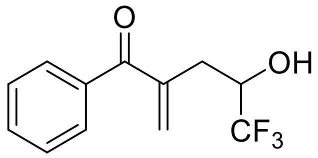

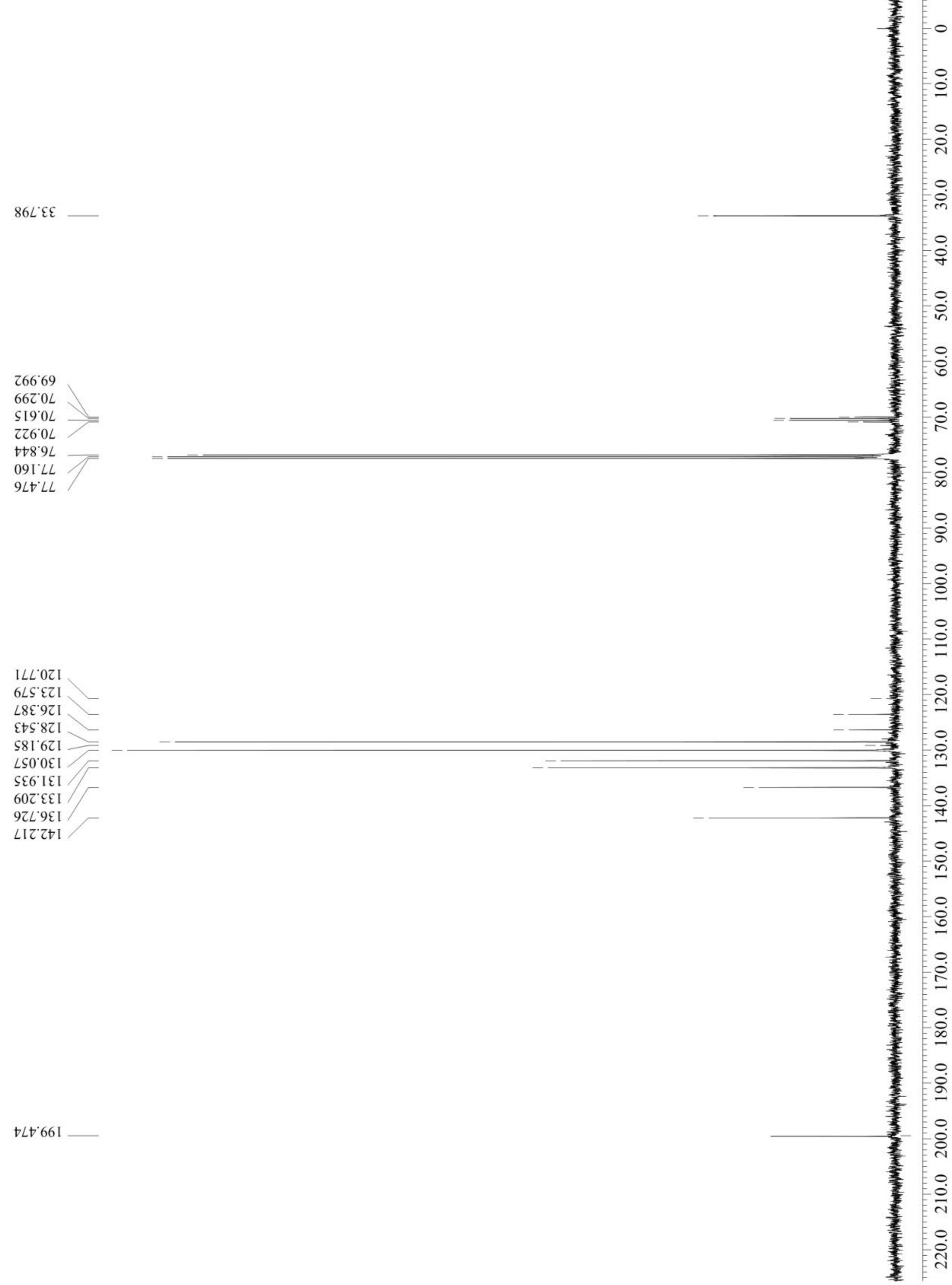

^{19}F NMR of **9b** ($CDCl_3$, 375 MHz, 25 ºC)

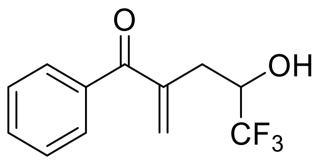

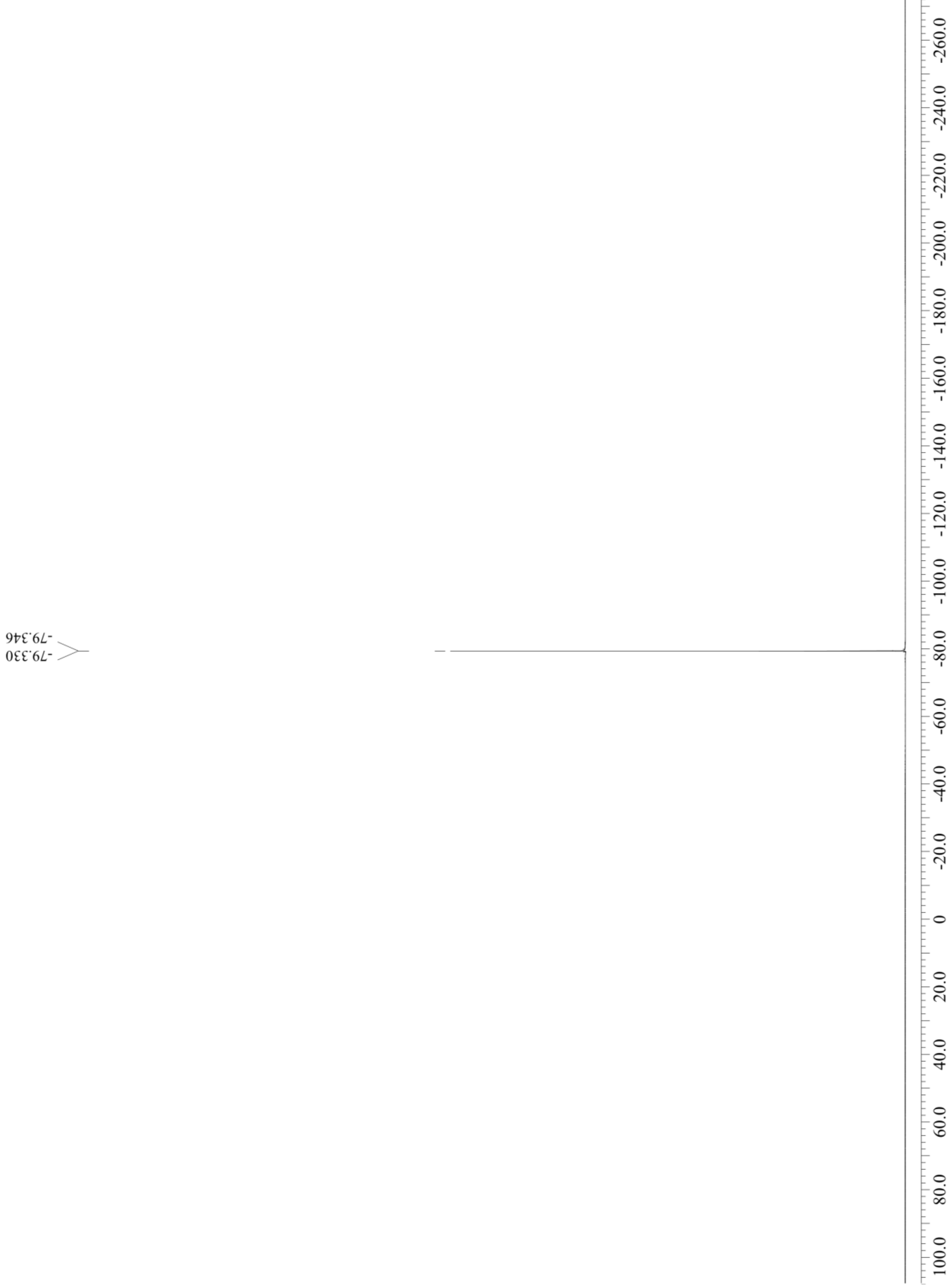

^{1}H NMR of **9c** ($CDCl_3$, 400 MHz, 25 ºC)

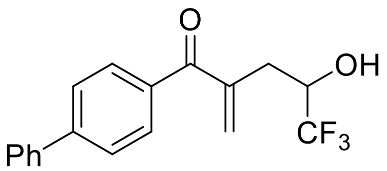

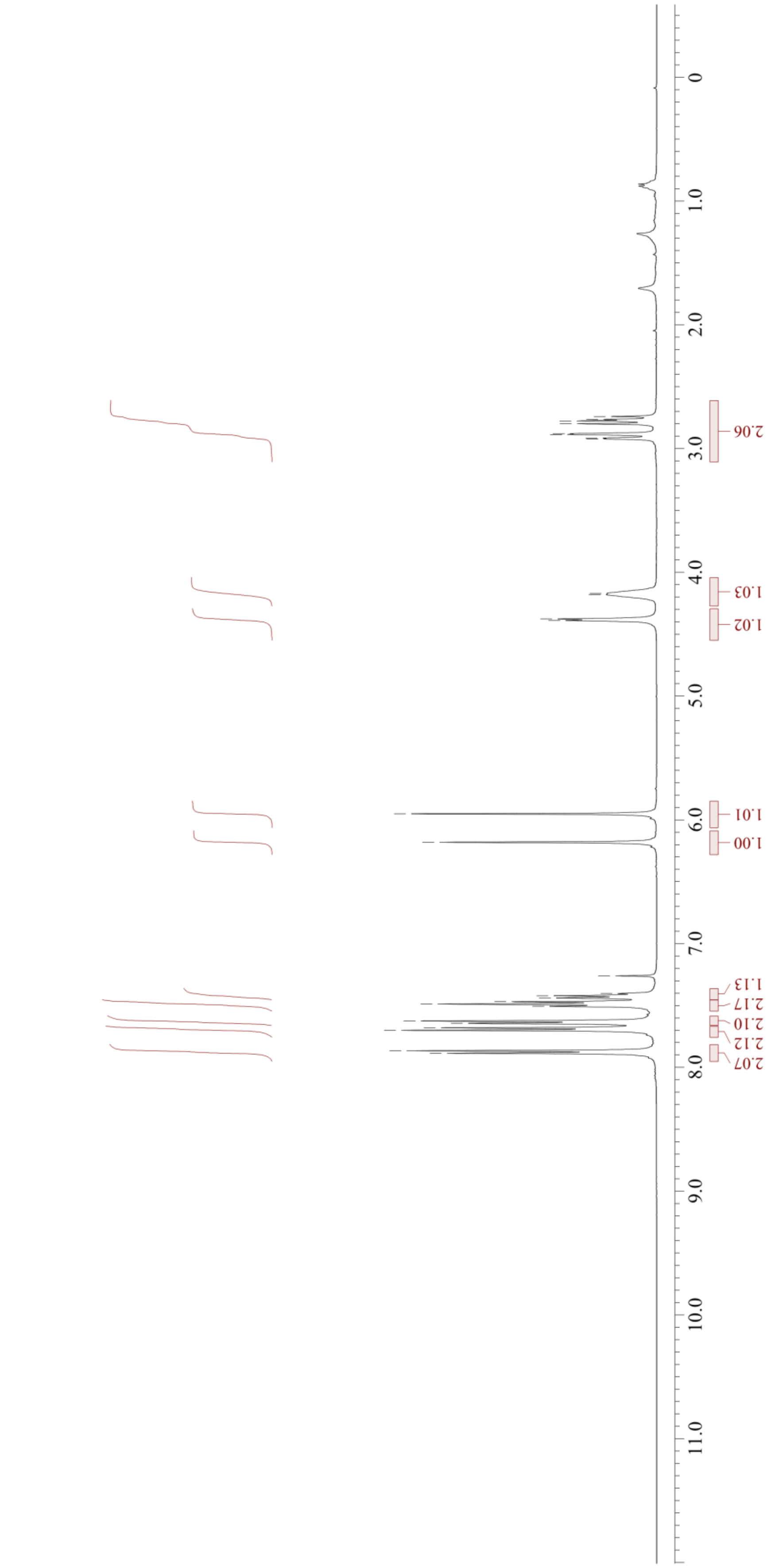

^{13}C NMR of **9c** ($CDCl_3$, 100 MHz, 25 ºC)

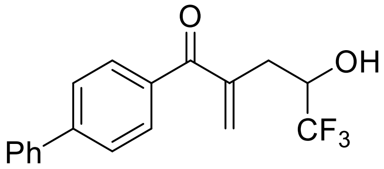

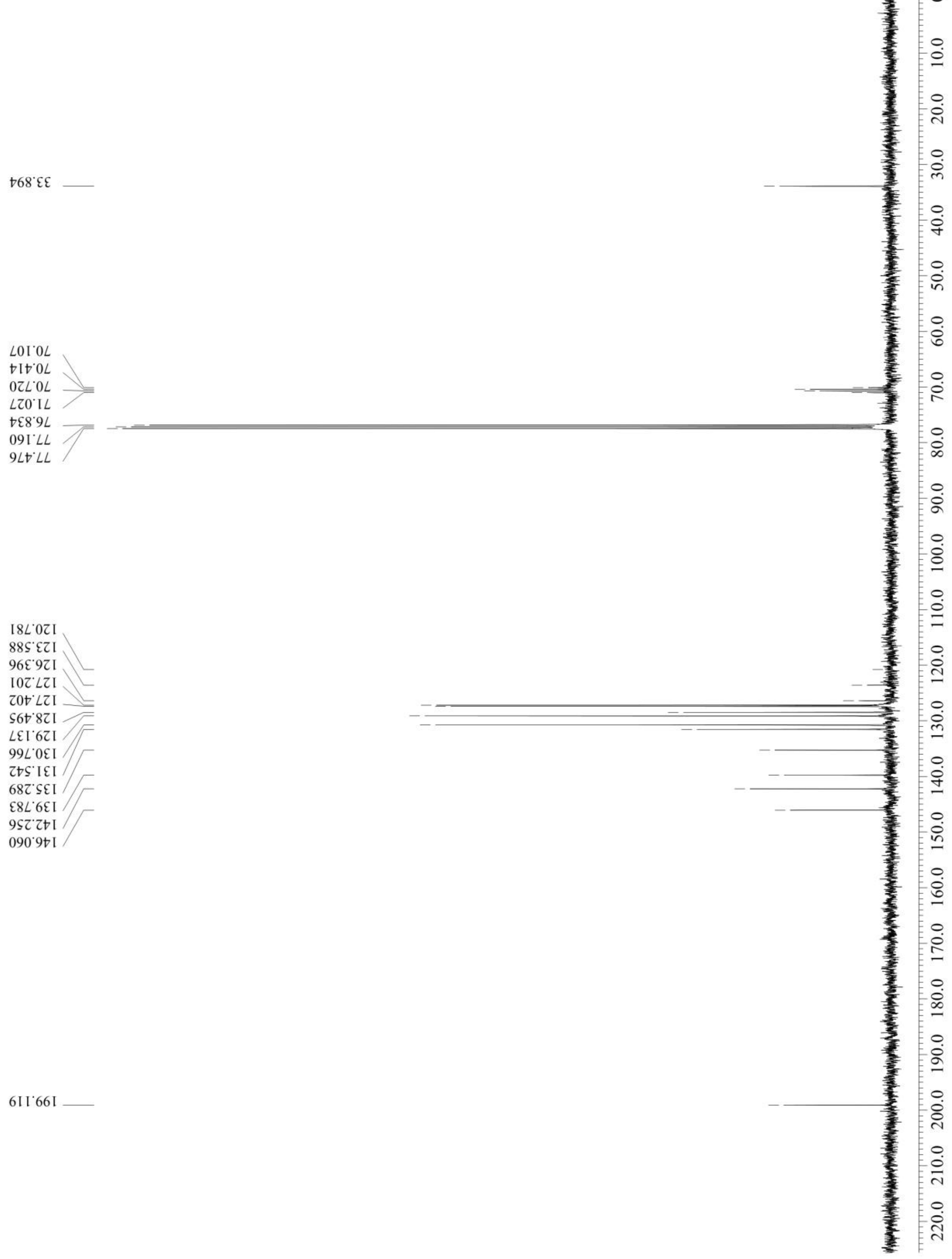

^{19}F NMR of **9c** ($CDCl_3$, 375 MHz, 25 ºC)

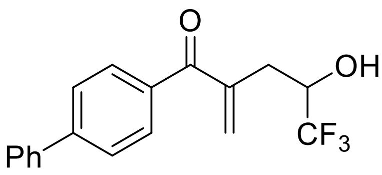

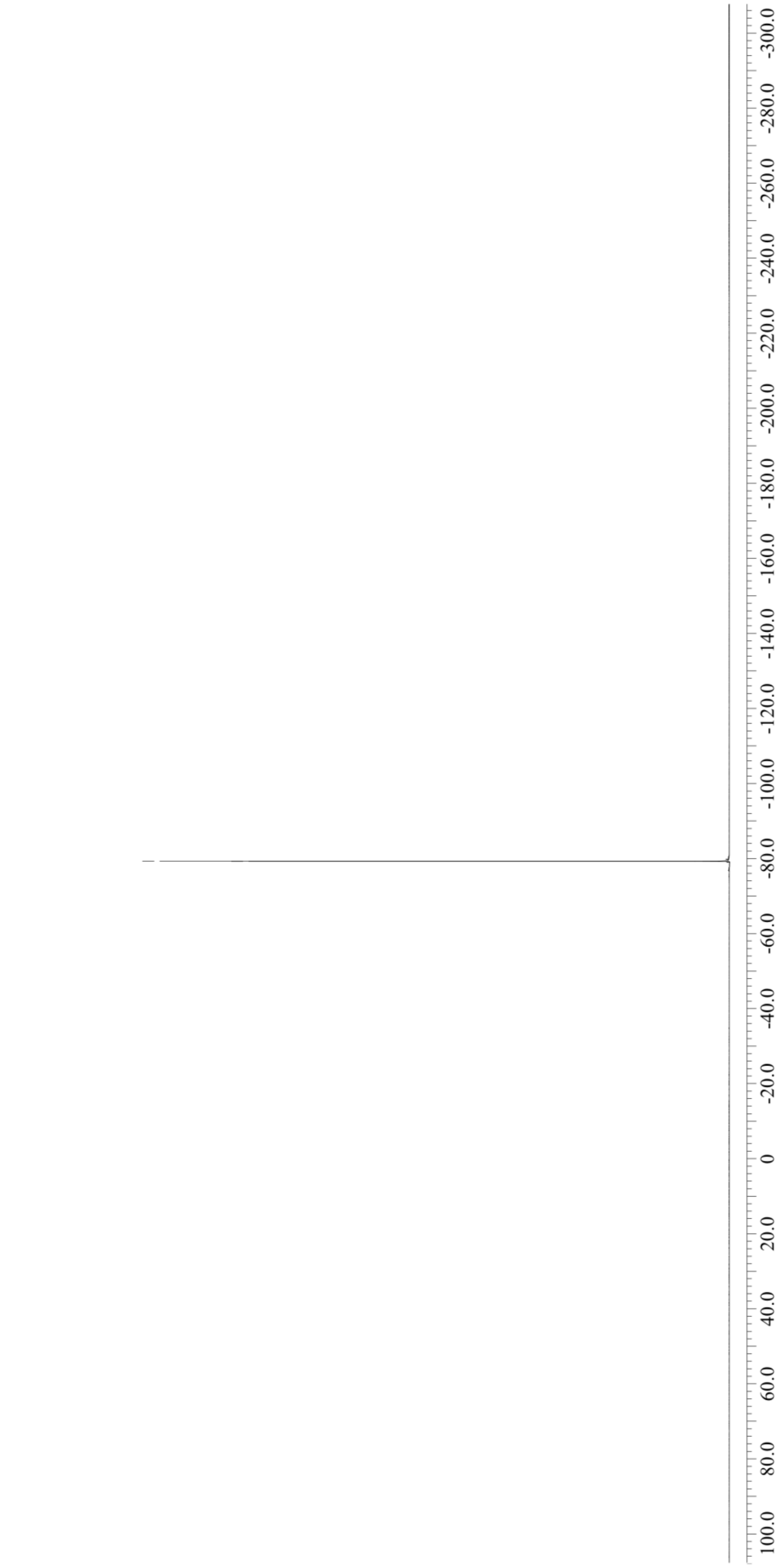

^{1}H NMR of **9d** ($CDCl_3$, 400 MHz, 25 °C)

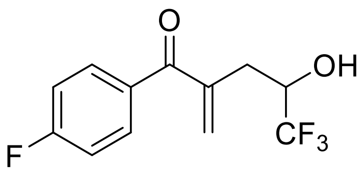

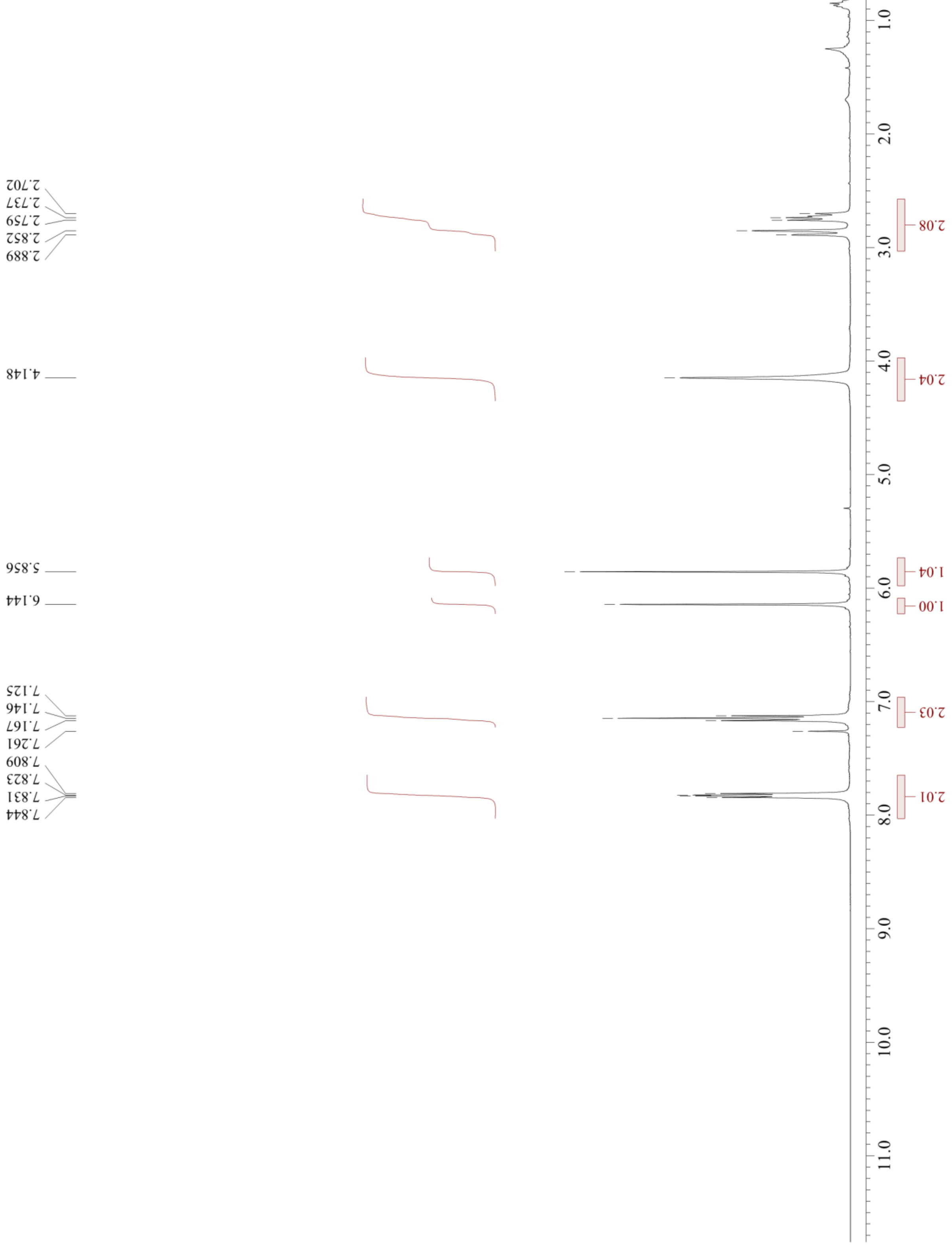

^{13}C NMR of **9d** ($CDCl_3$, 100 MHz, 25 ºC)

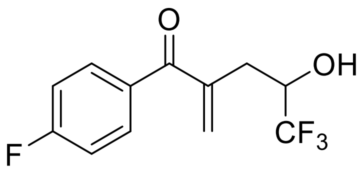

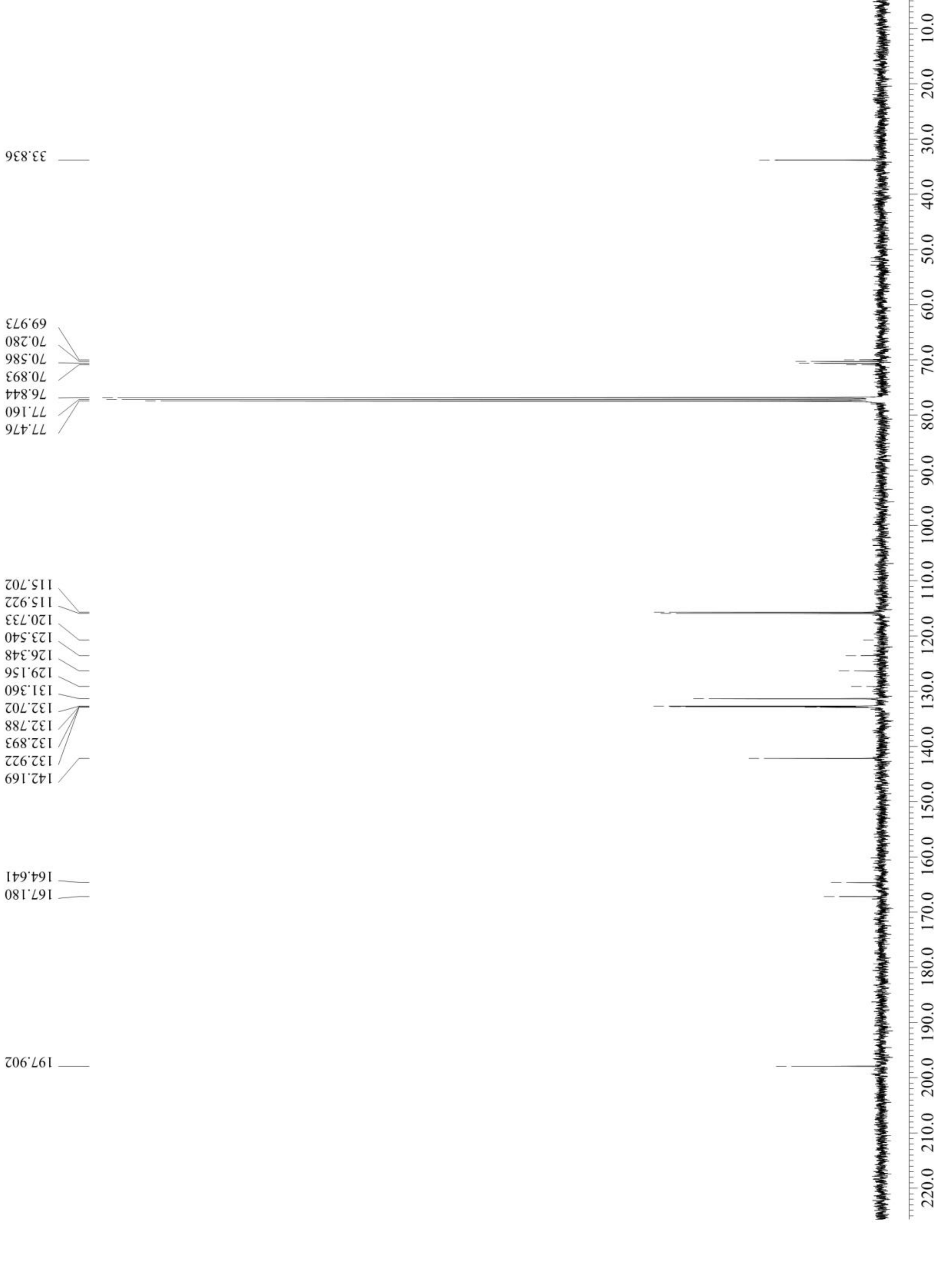

^{19}F NMR of **9d** ($CDCl_3$, 375 MHz, 25 ºC)

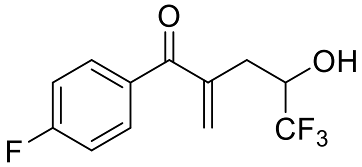

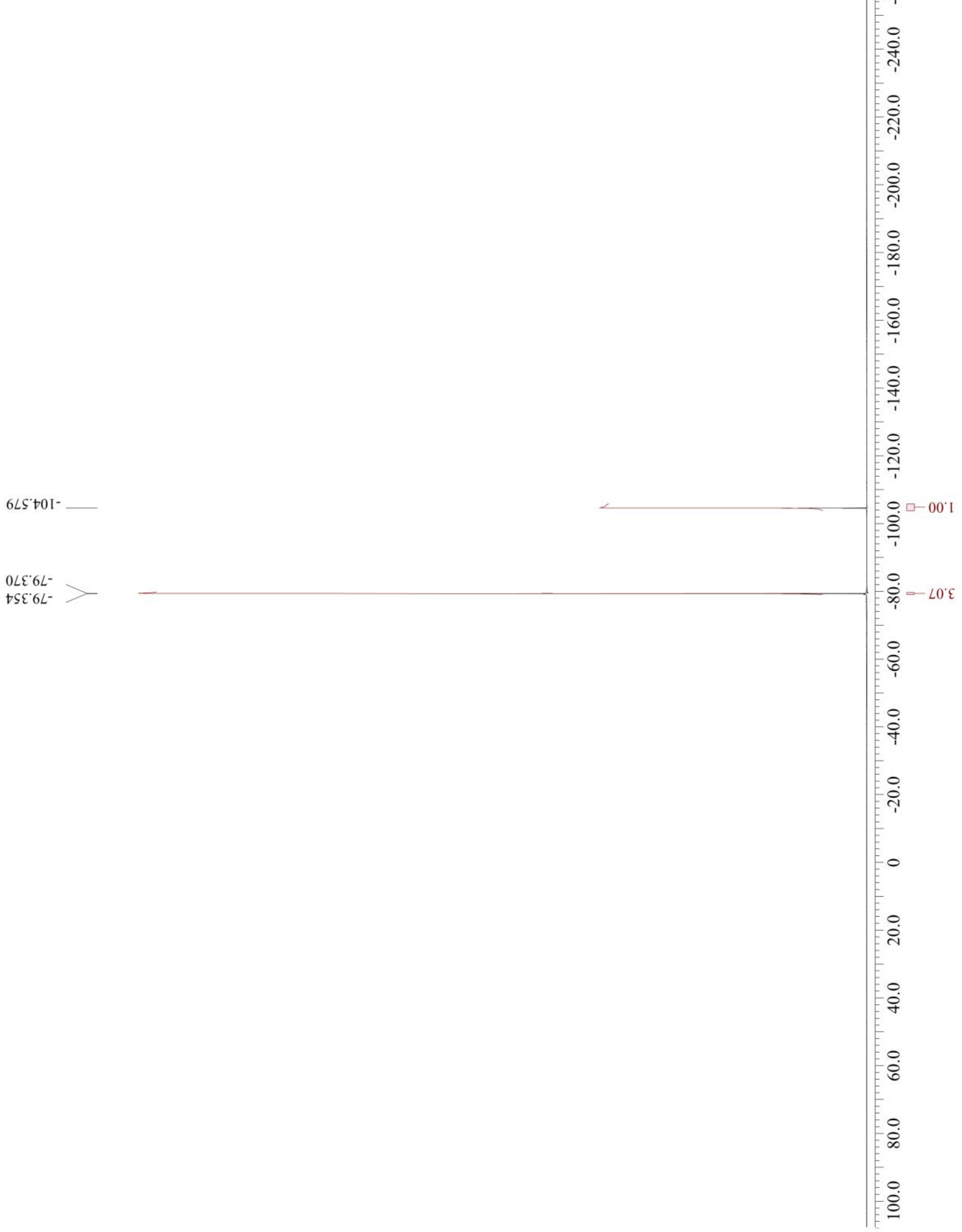

^{1}H NMR of **9e** ($CDCl_3$, 400 MHz, 25 ºC)

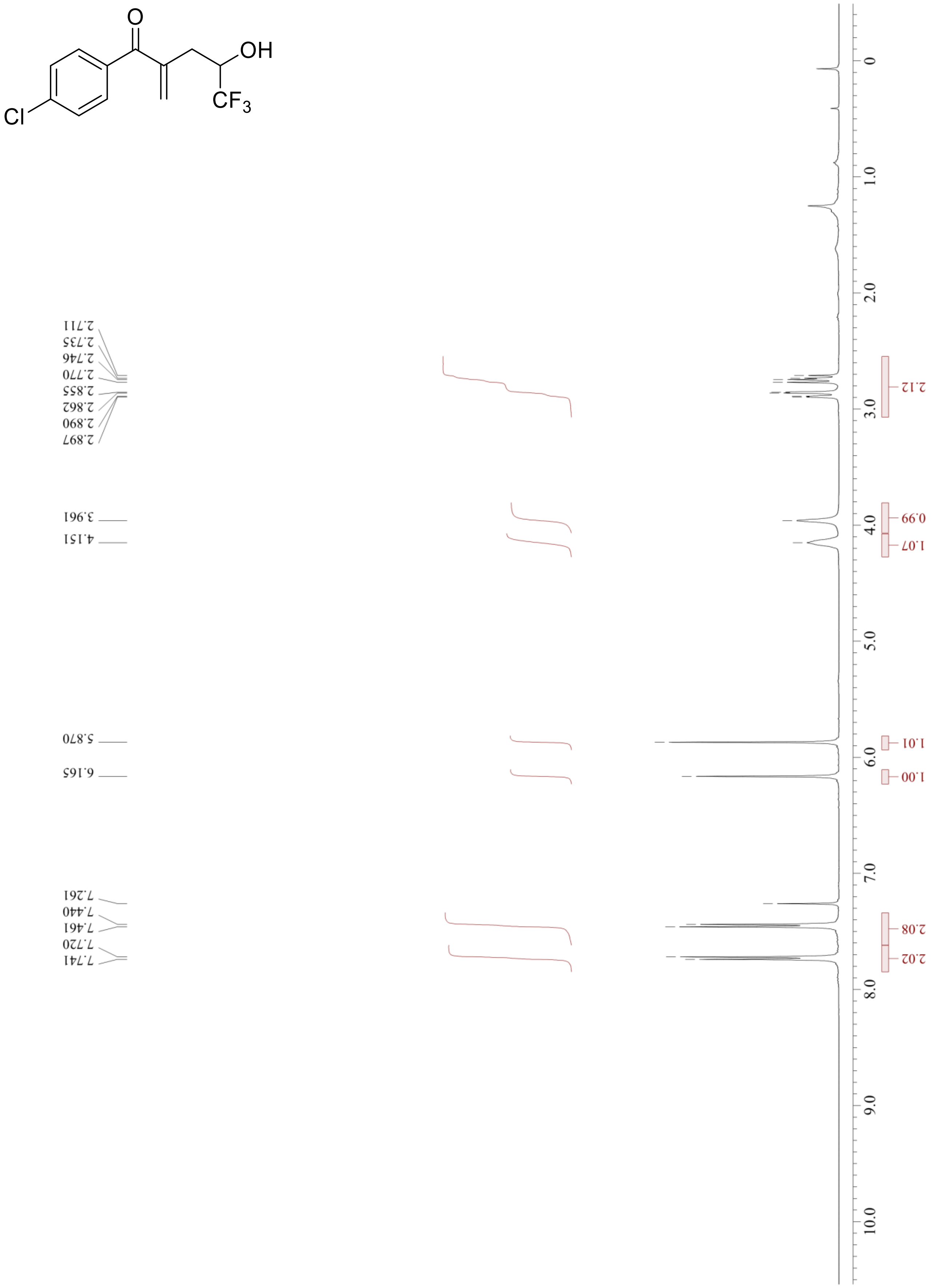

^{13}C NMR of **9e** ($CDCl_3$, 100 MHz, 25 ºC)

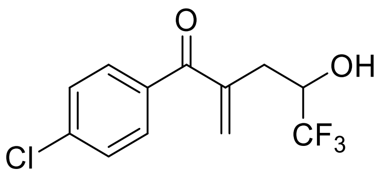

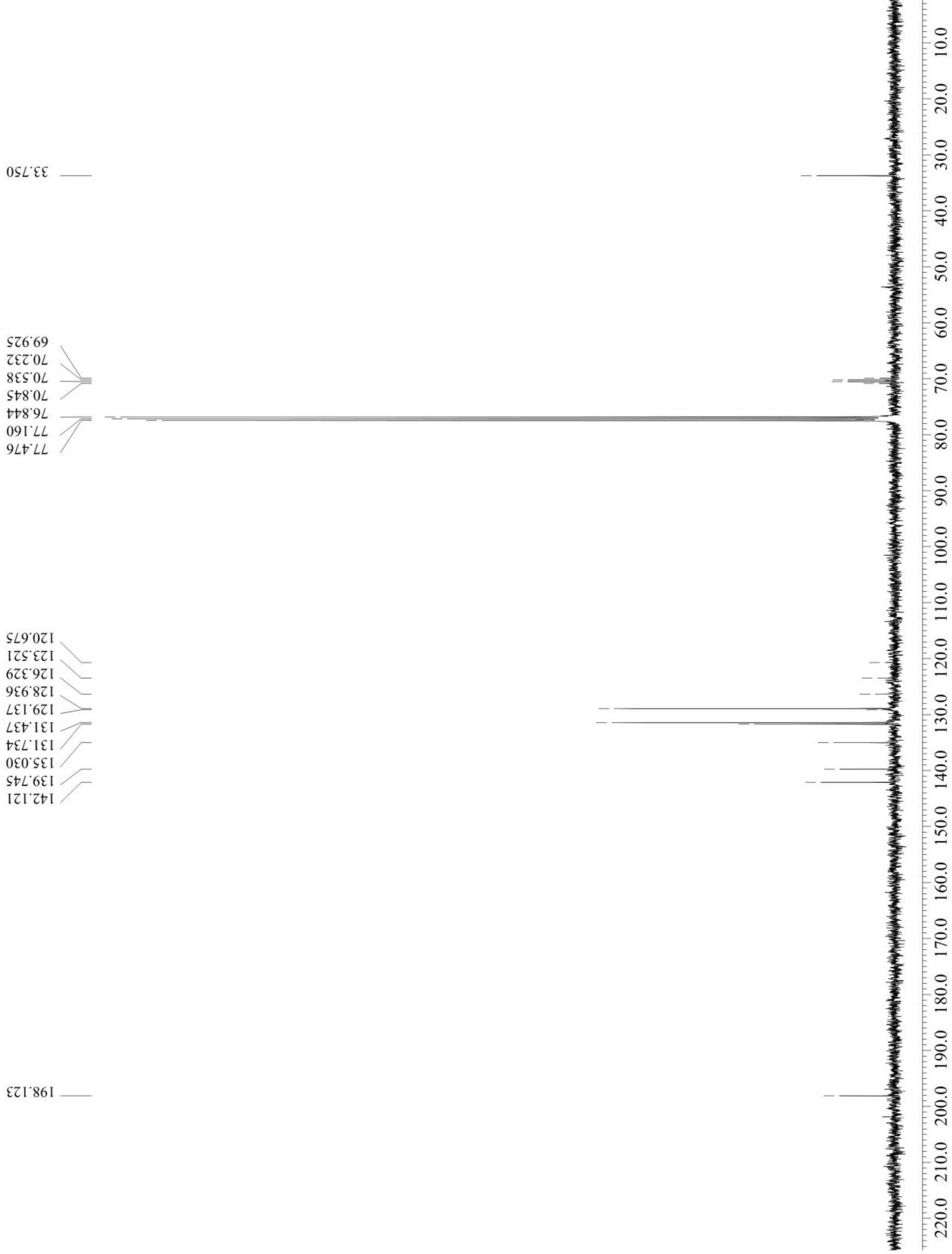

^{19}F NMR of **9e** ($CDCl_3$, 375 MHz, 25 ºC)

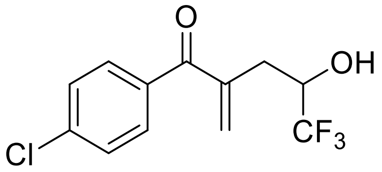

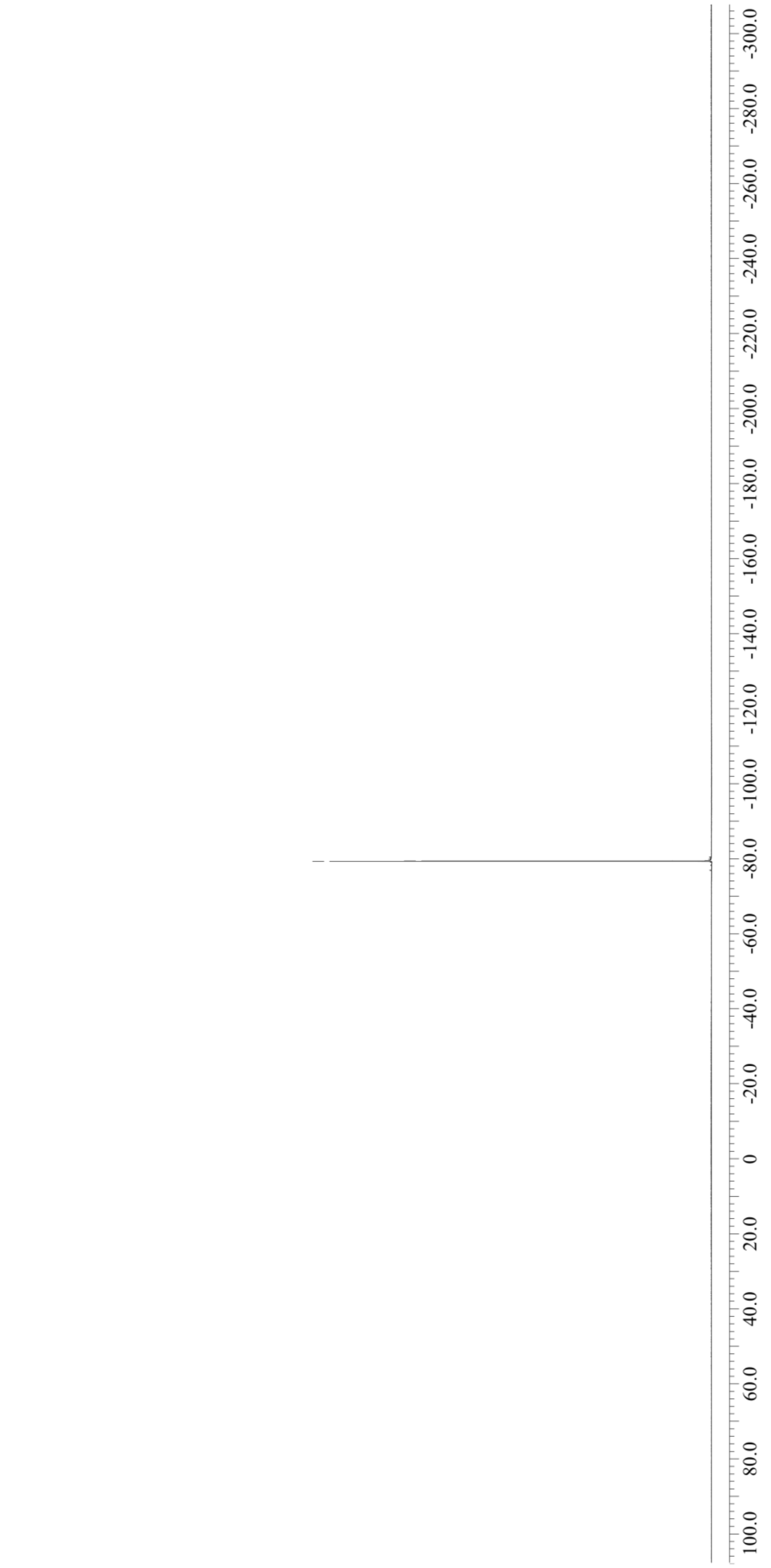

^{1}H NMR of **9f** ($CDCl_3$, 400 MHz, 25 ºC)

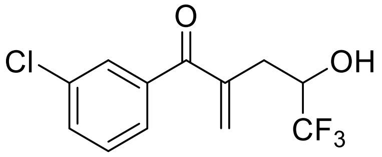

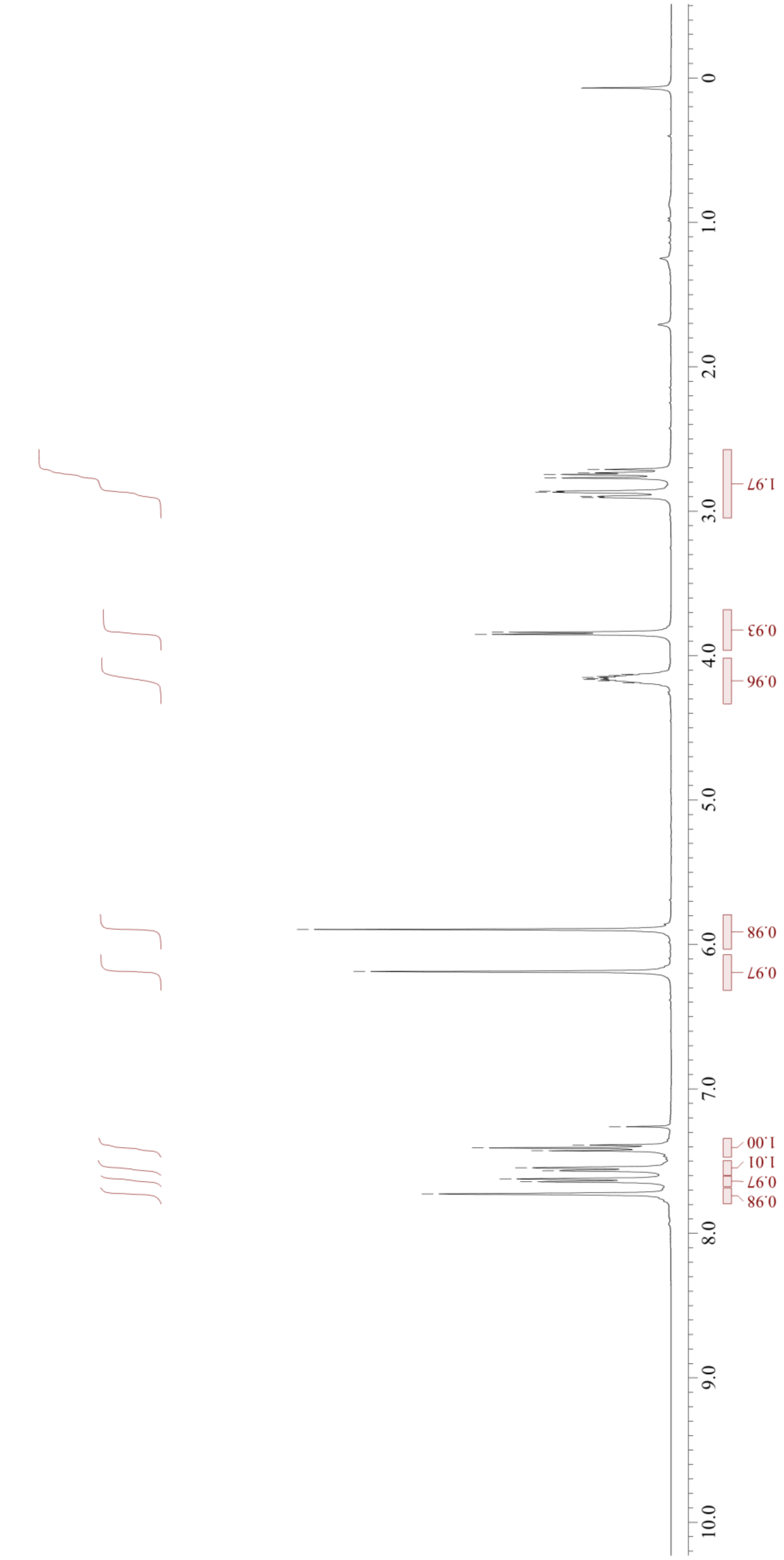

^{13}C NMR of **9f** ($CDCl_3$, 100 MHz, 25 ºC)

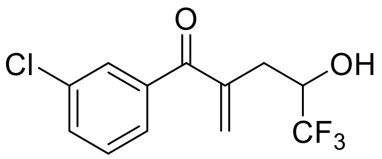

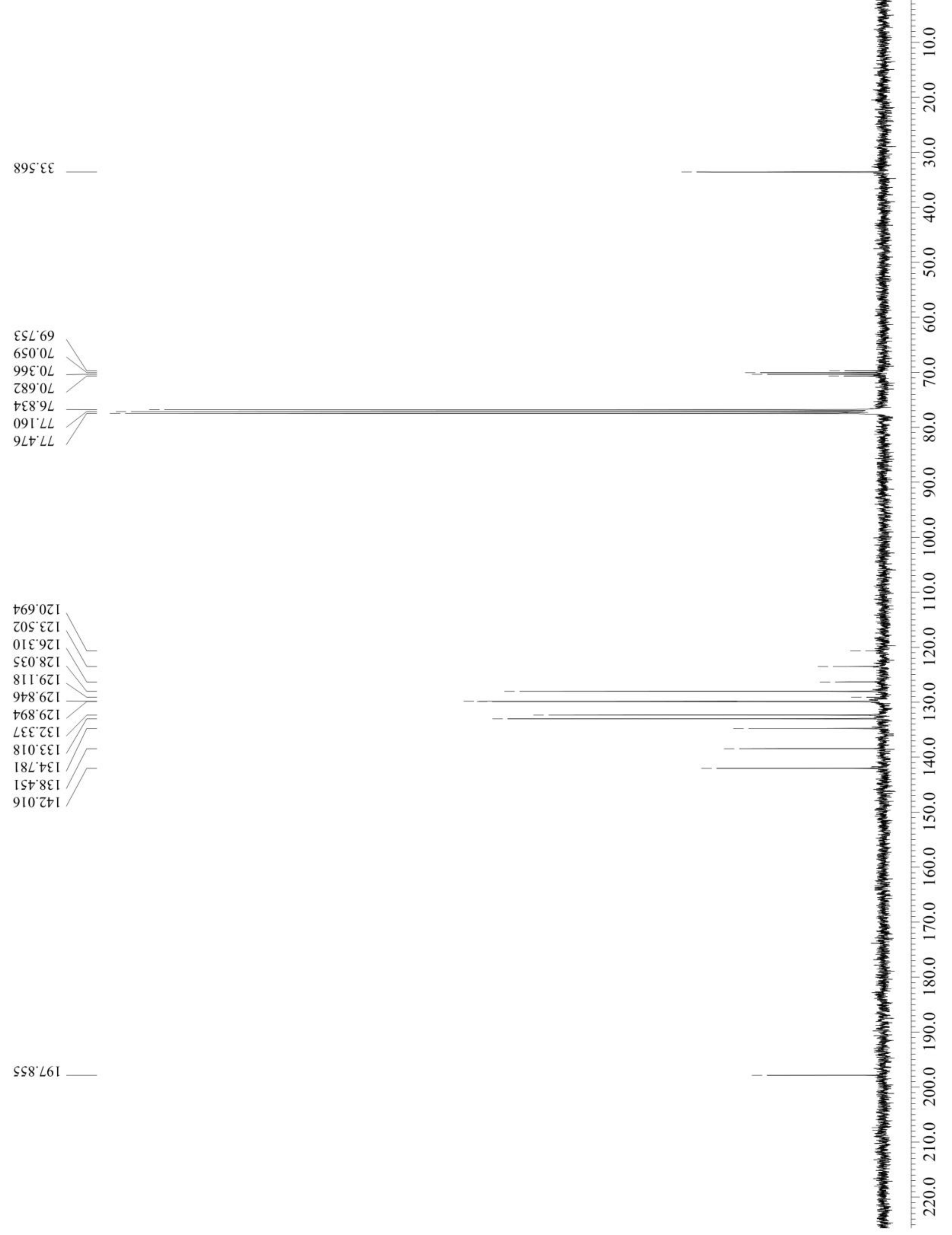

^{19}F NMR of **9f** ($CDCl_3$, 375 MHz, 25 ºC)

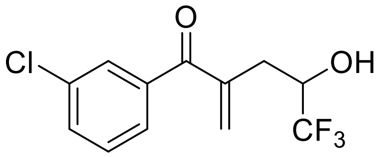

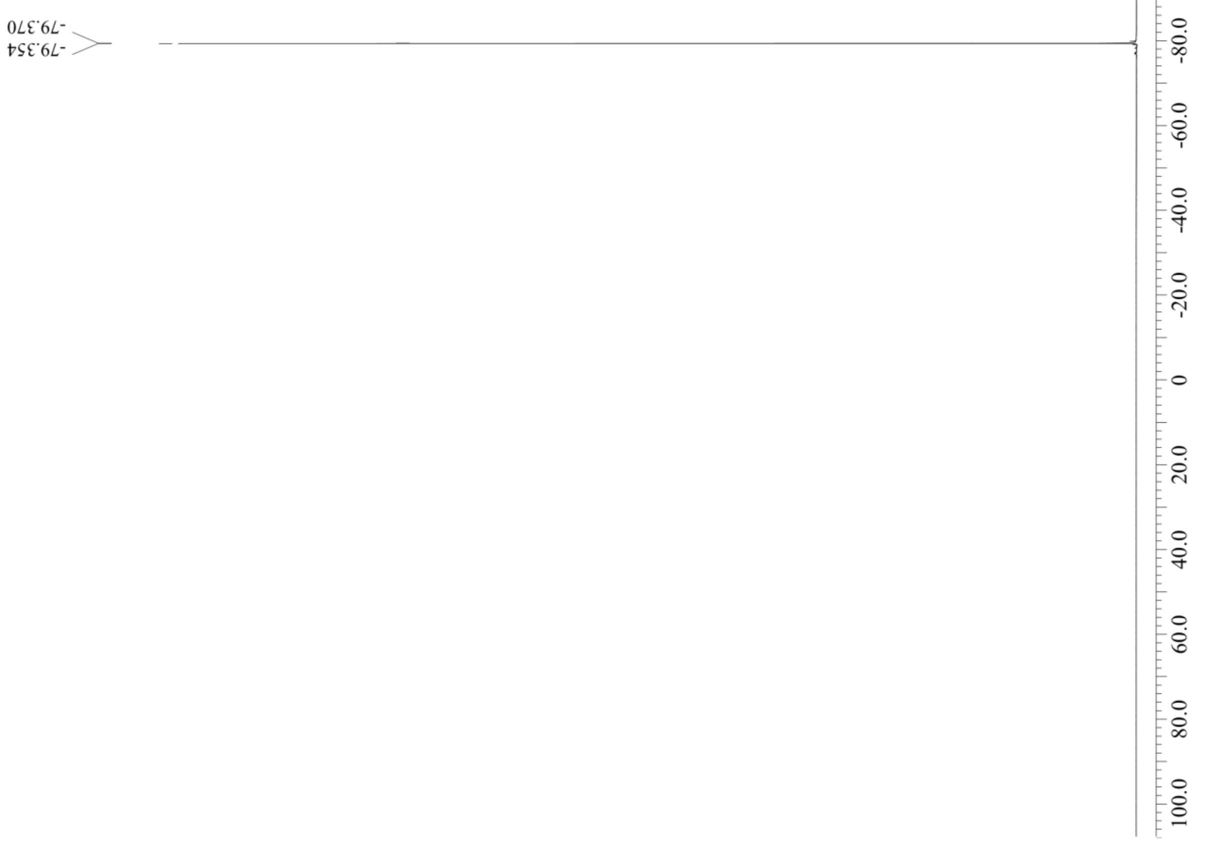

^{1}H NMR of **9g** ($CDCl_3$, 400 MHz, 25 °C)

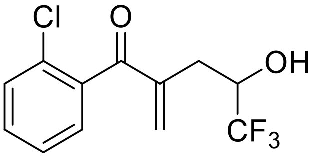

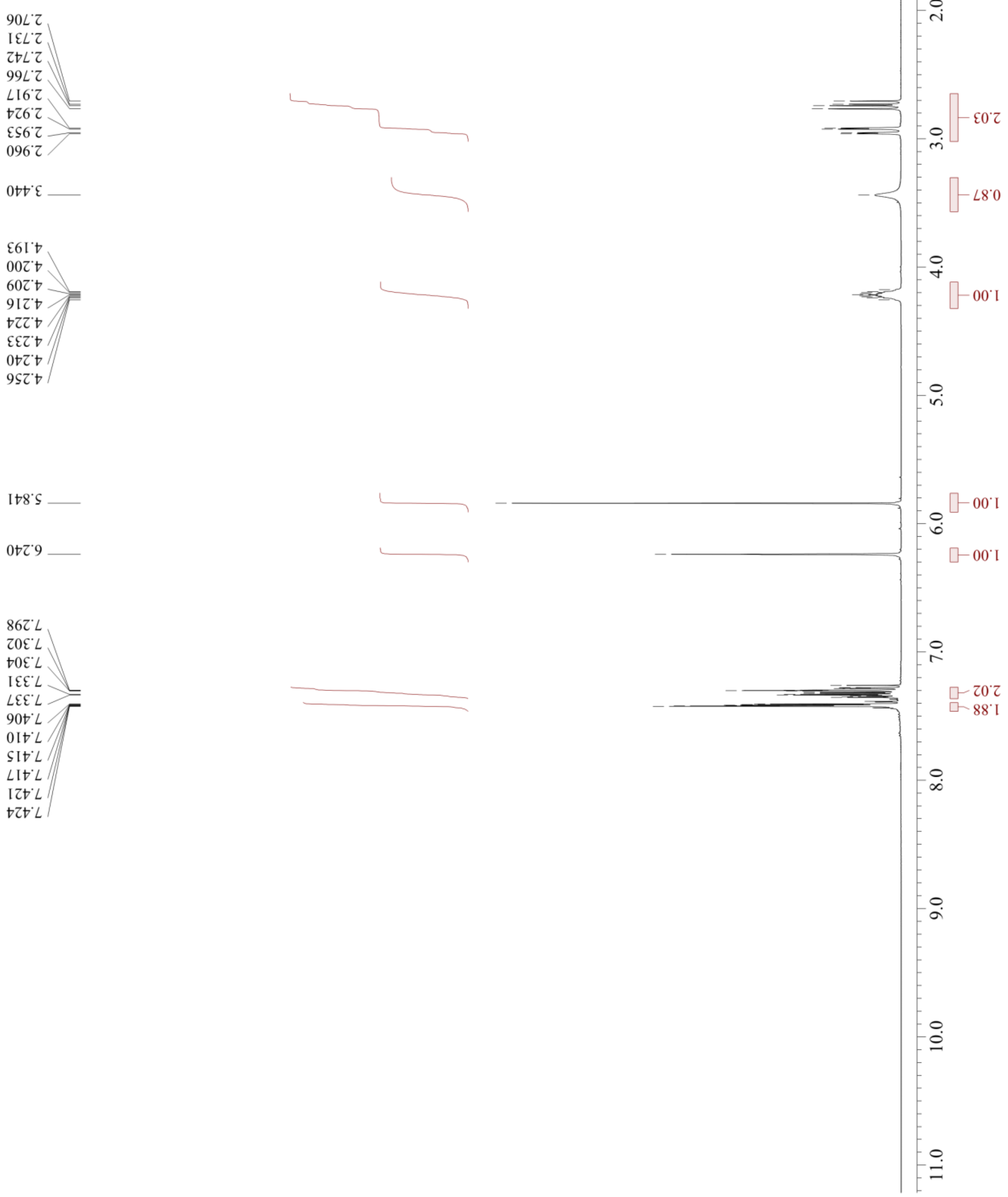

^{13}C NMR of **9g** ($CDCl_3$, 100 MHz, 25 ºC)

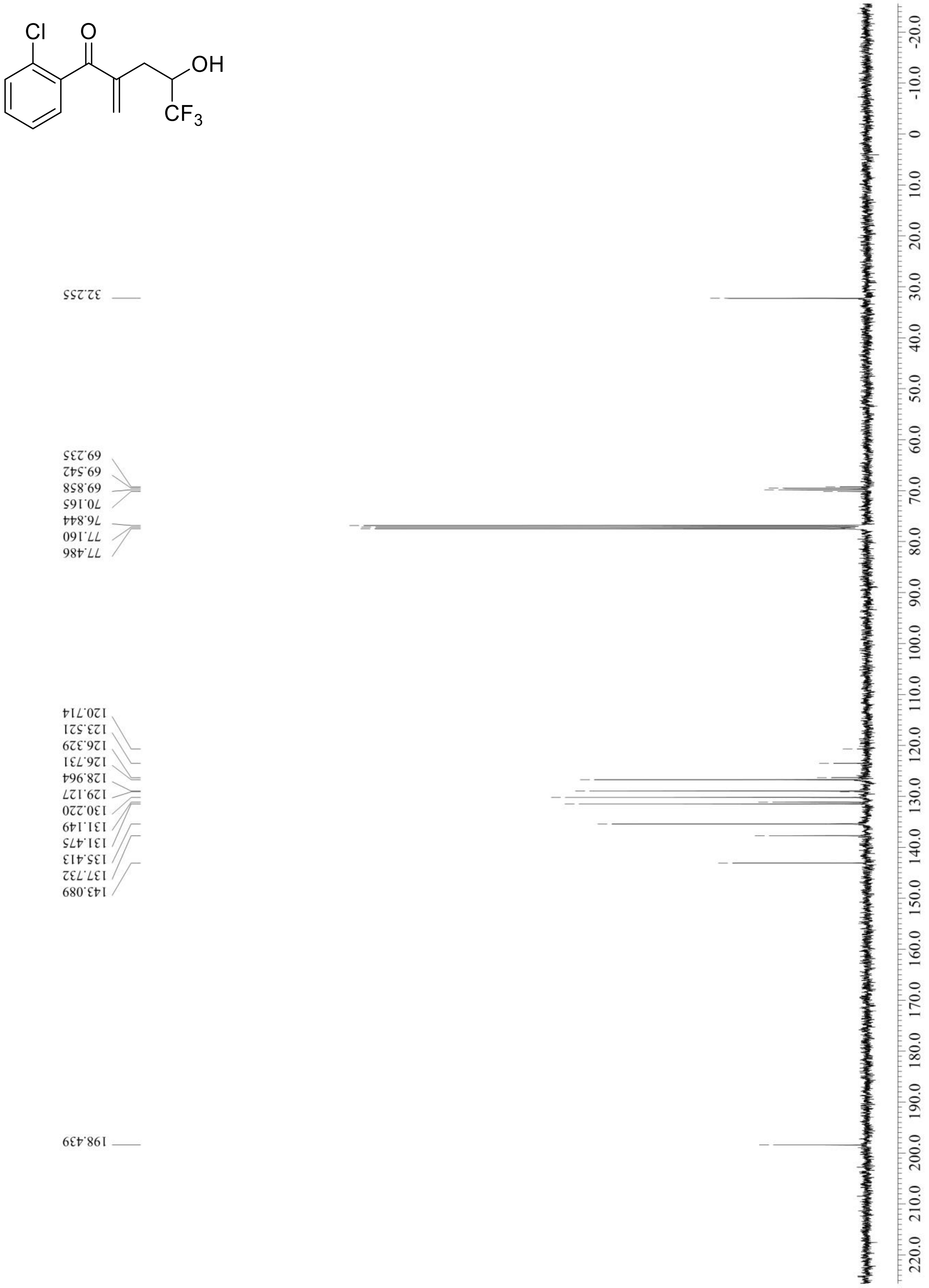

^{19}F NMR of **9g** ($CDCl_3$, 375 MHz, 25 ºC)

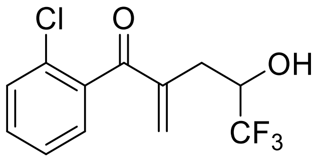

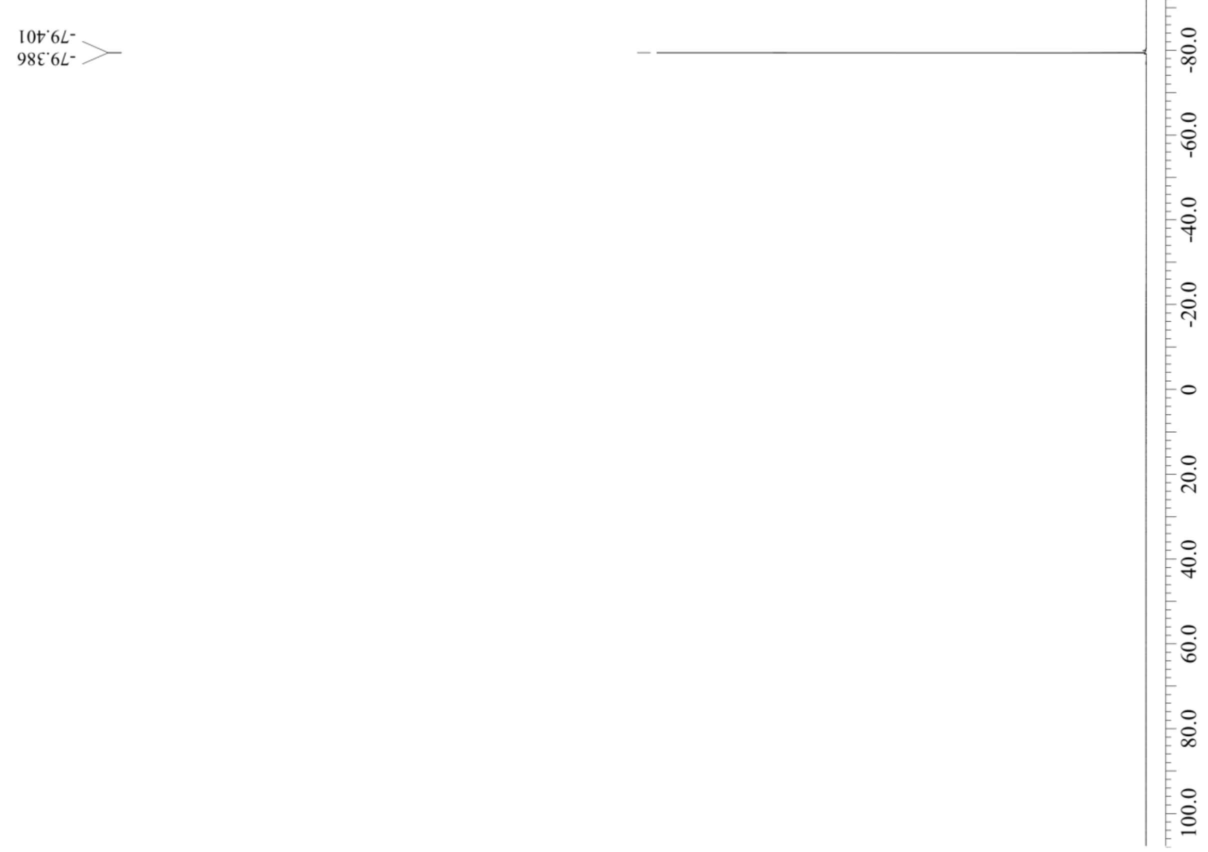

^{1}H NMR of **9h** ($CDCl_3$, 400 MHz, 25 ºC)

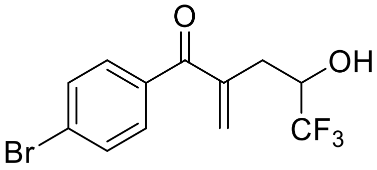

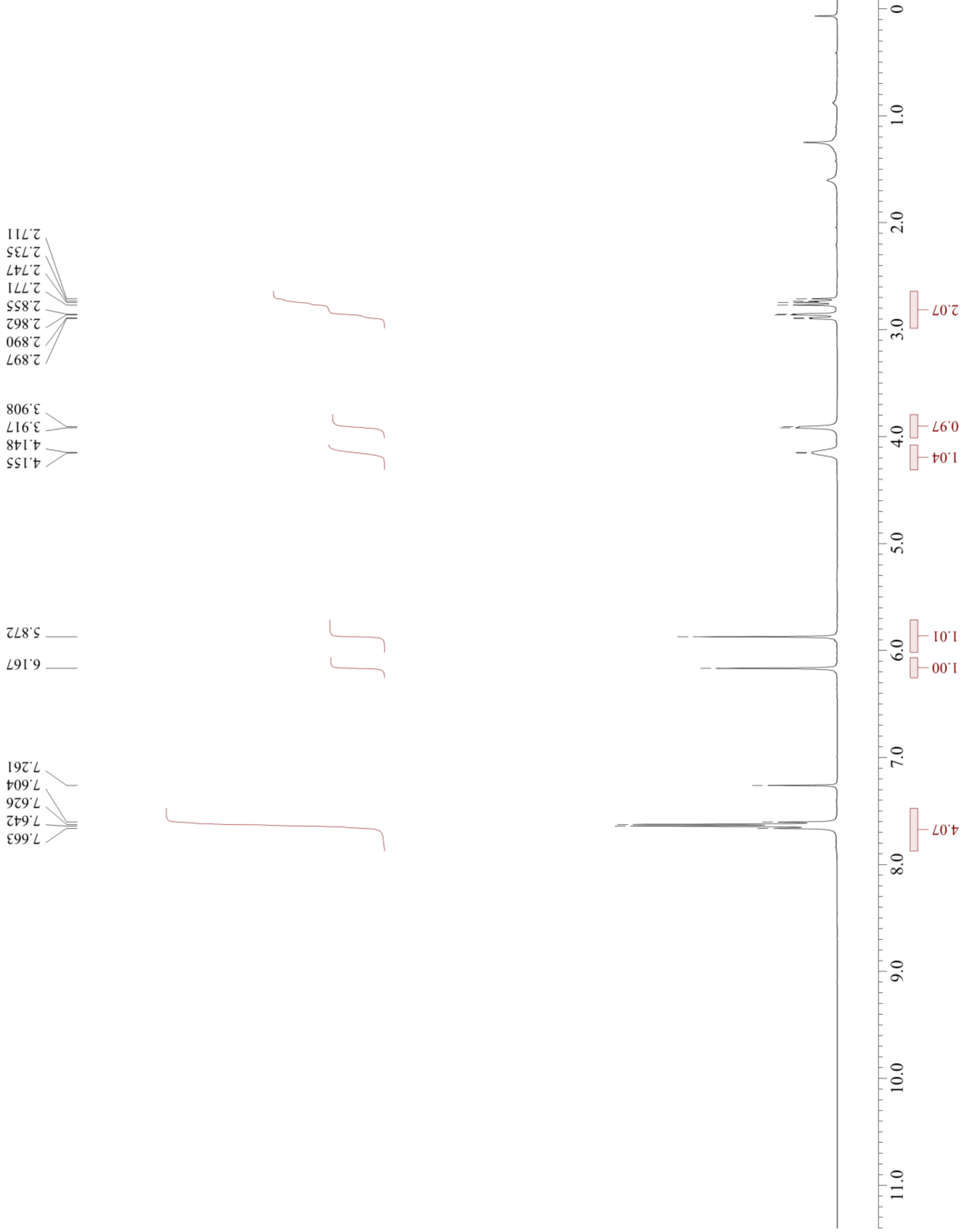

^{13}C NMR of **9h** ($CDCl_3$, 100 MHz, 25 ºC)

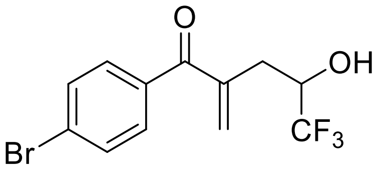

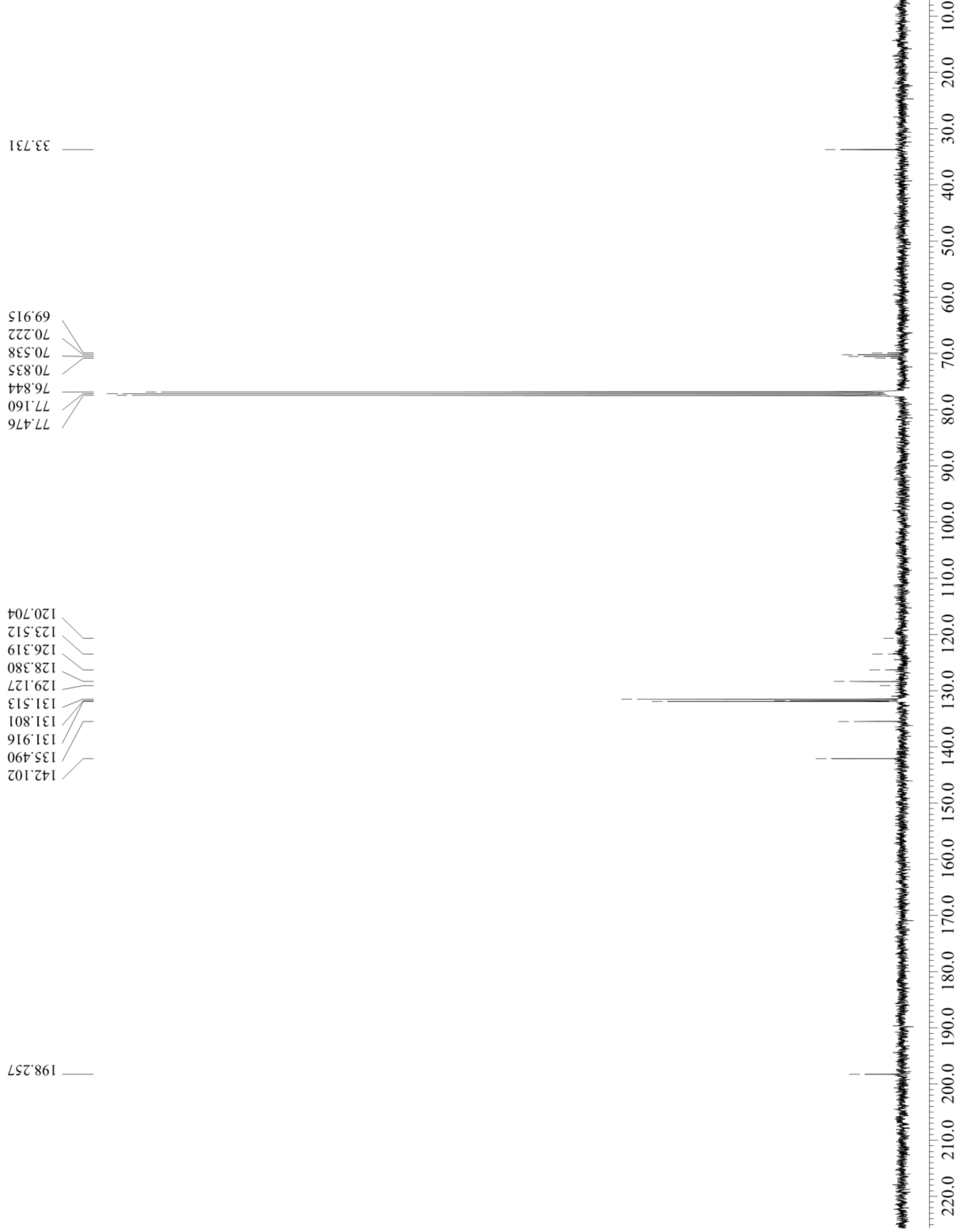

^{19}F NMR of **9h** ($CDCl_3$, 375 MHz, 25 ºC)

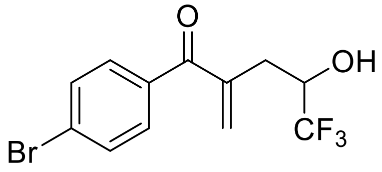

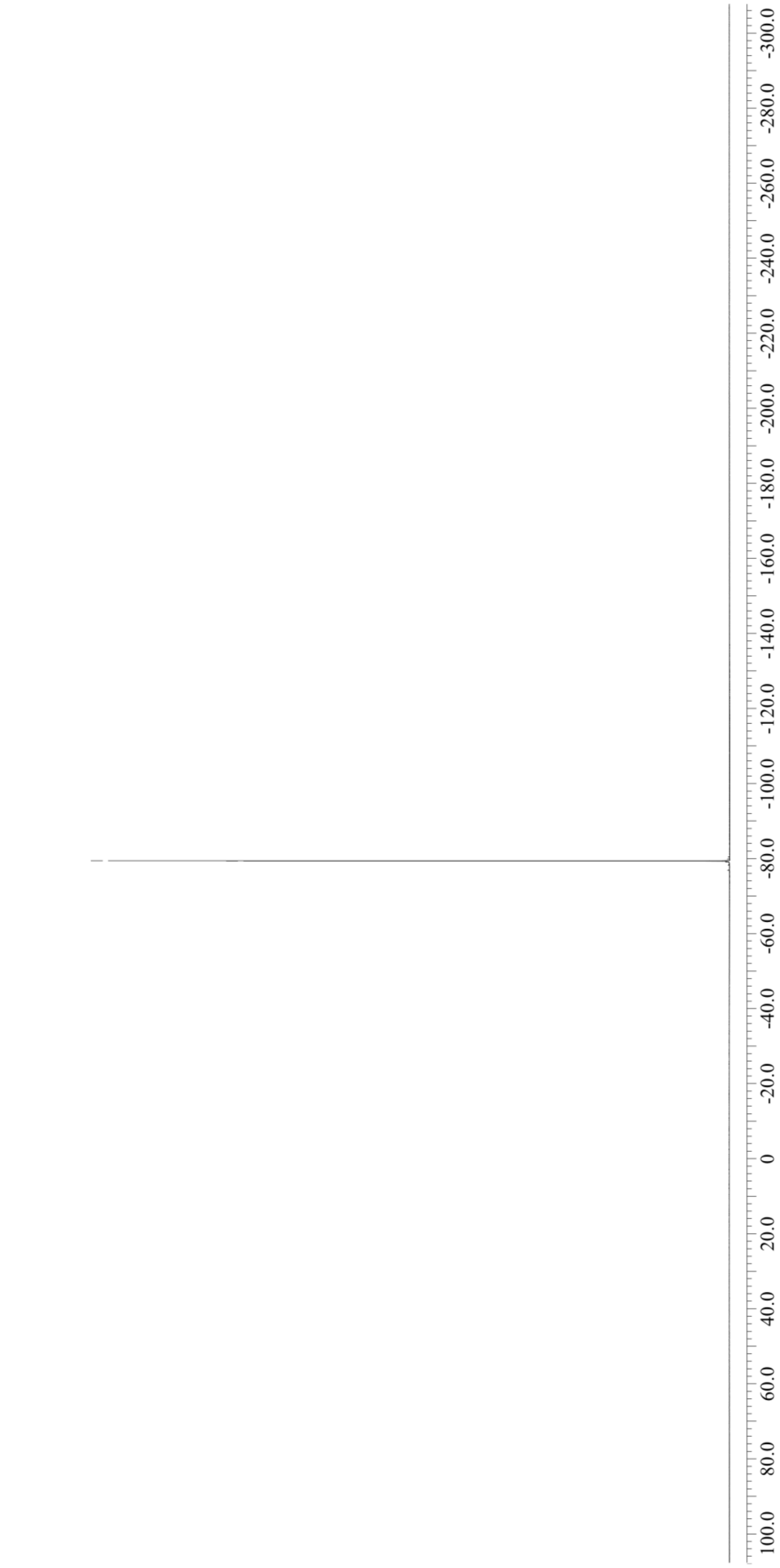

^{1}H NMR of **9i** ($CDCl_3$, 400 MHz, 25 ºC)

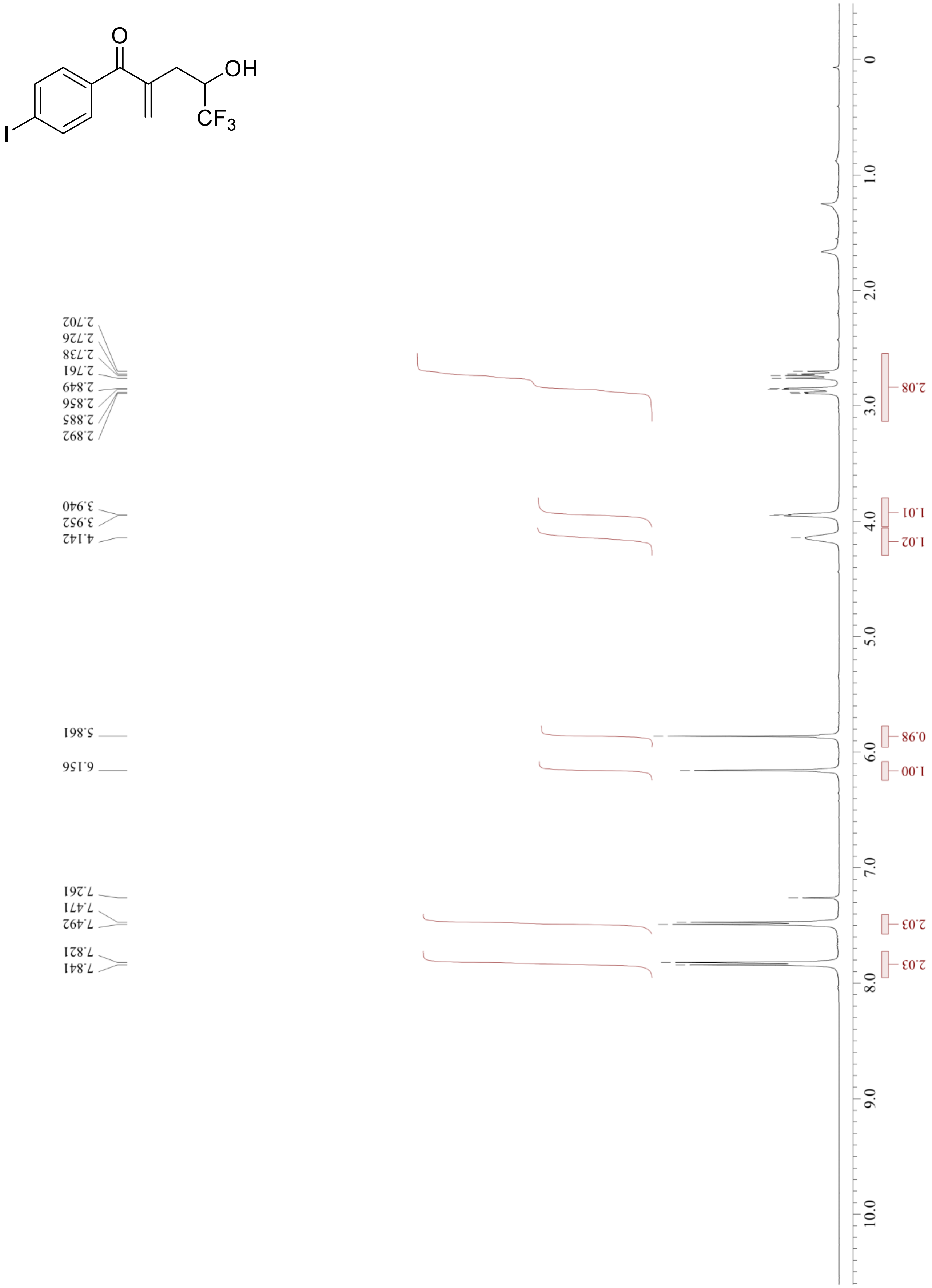

^{13}C NMR of **9i** ($CDCl_3$, 100 MHz, 25 ºC)

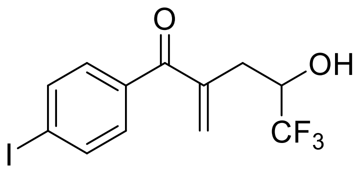

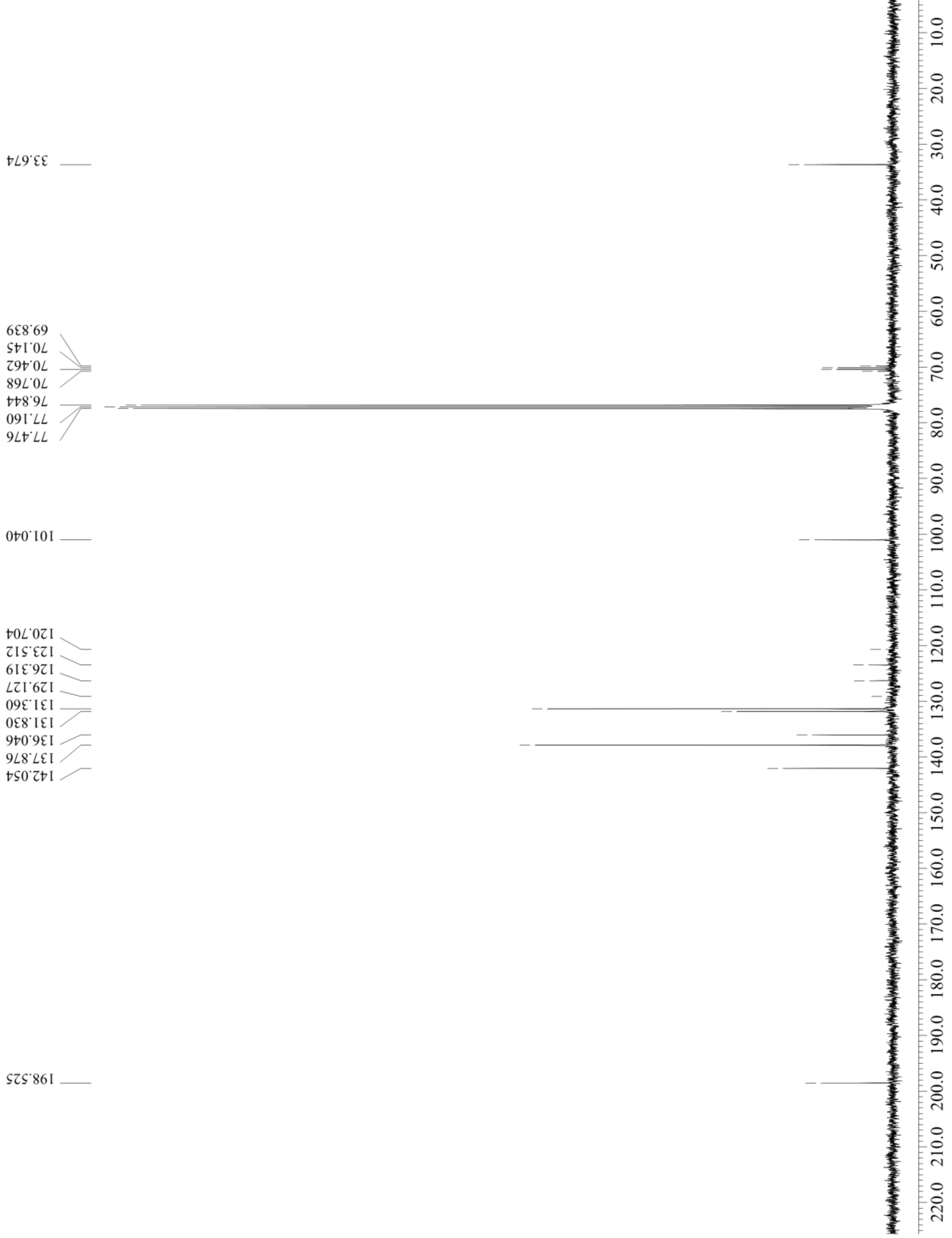

^{19}F NMR of **9i** ($CDCl_3$, 375 MHz, 25 °C)

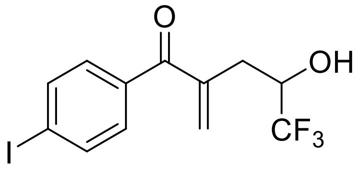

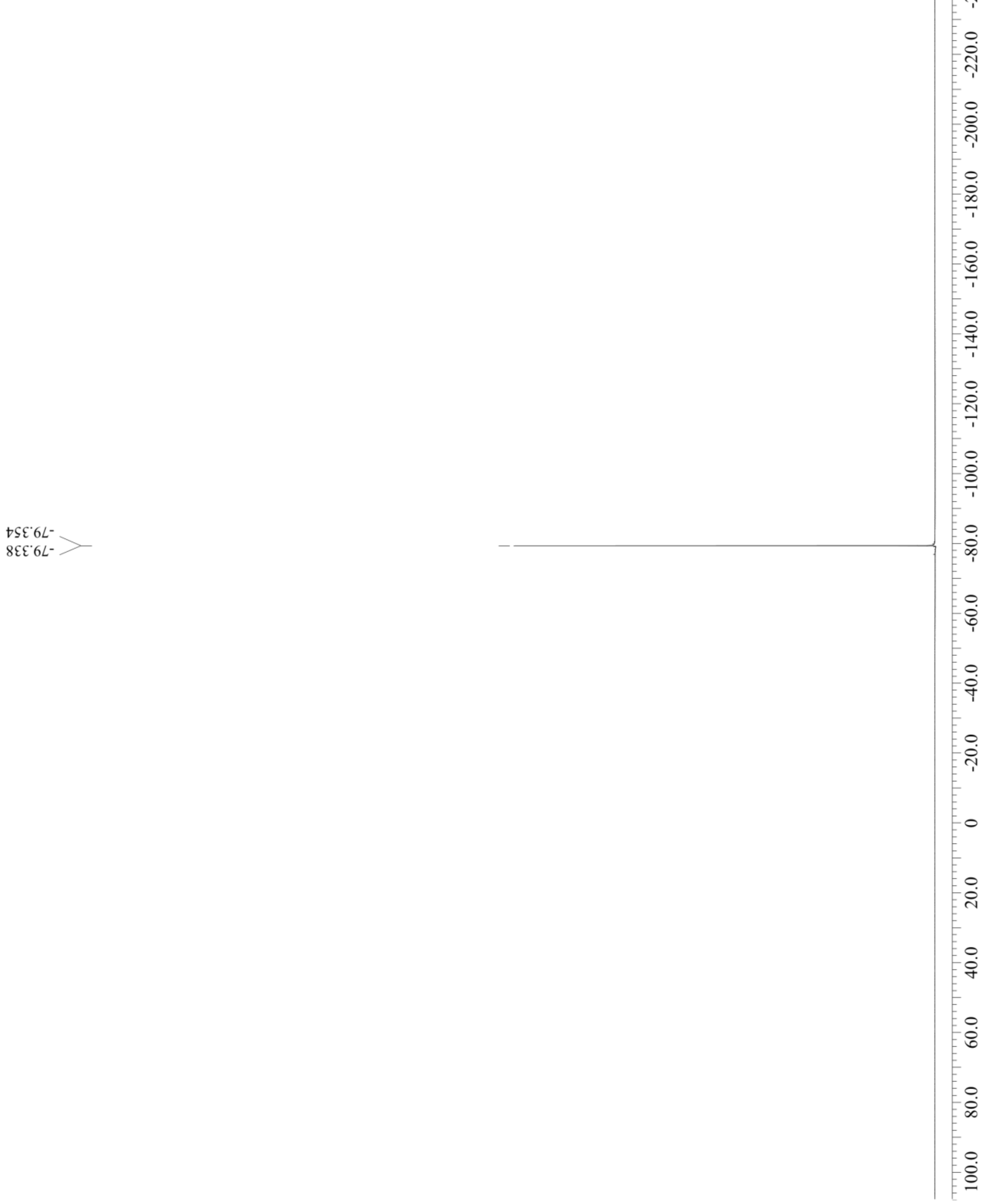

^{1}H NMR of **9j** ($CDCl_3$, 400 MHz, 25 ºC)

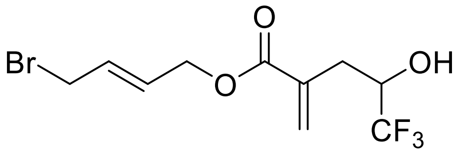

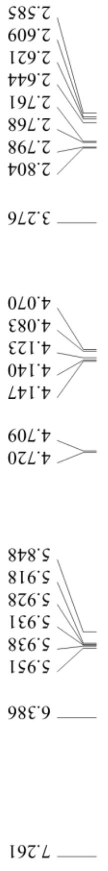

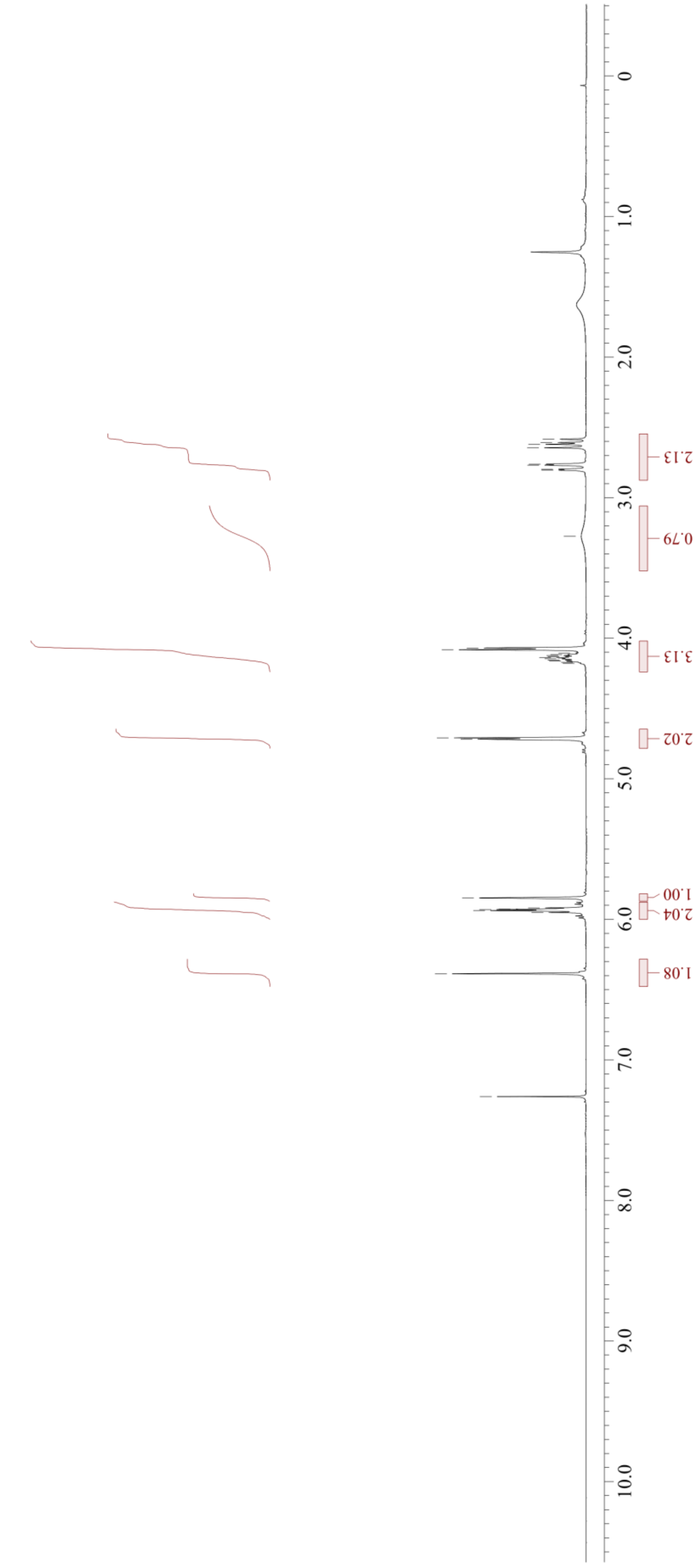

^{13}C NMR of **9j** ($CDCl_3$, 100 MHz, 25 ºC)

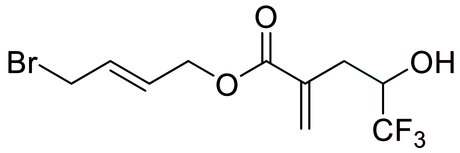

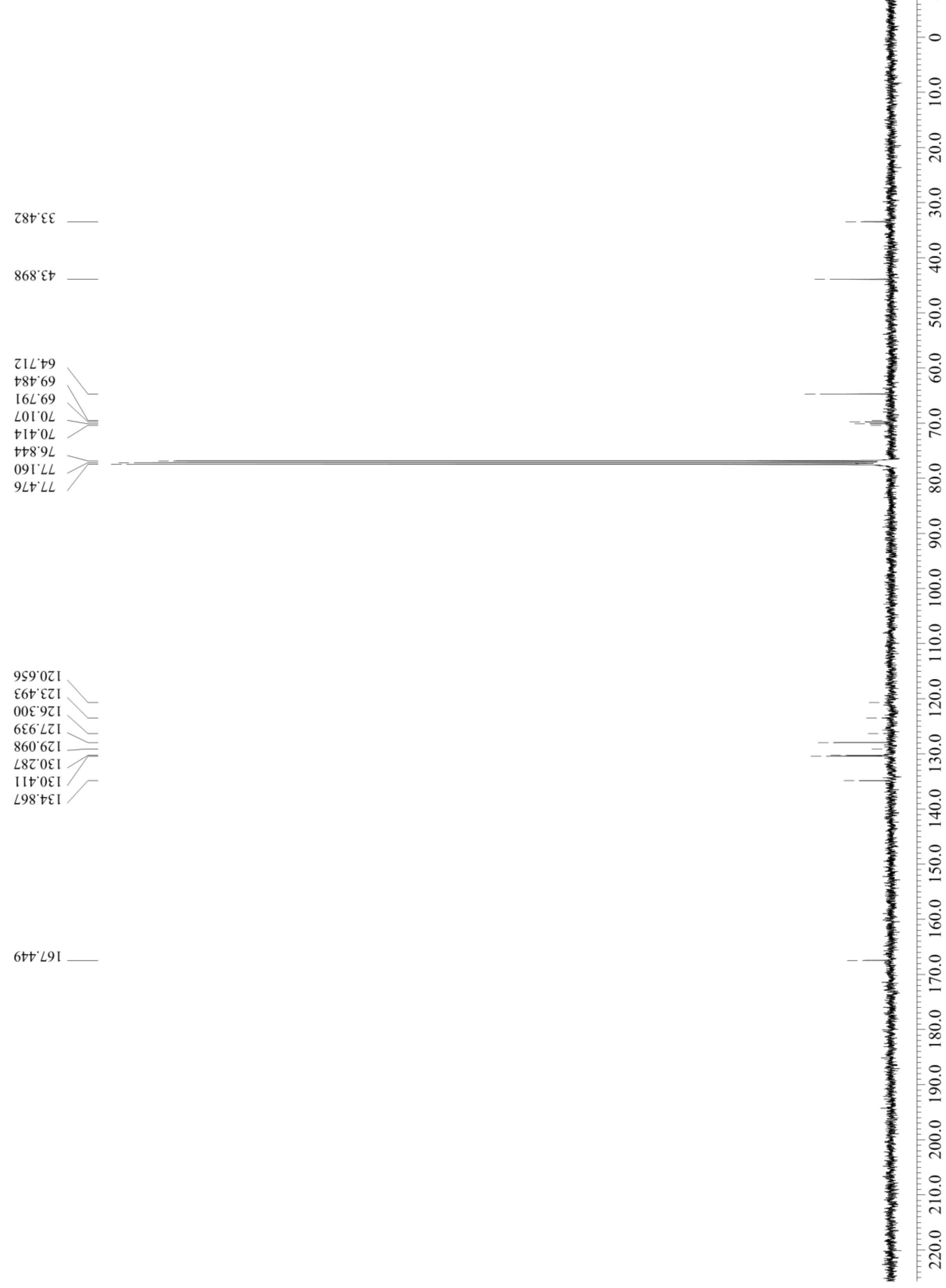

^{19}F NMR of **9j** ($CDCl_3$, 375 MHz, 25 ºC)

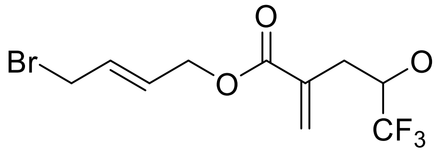

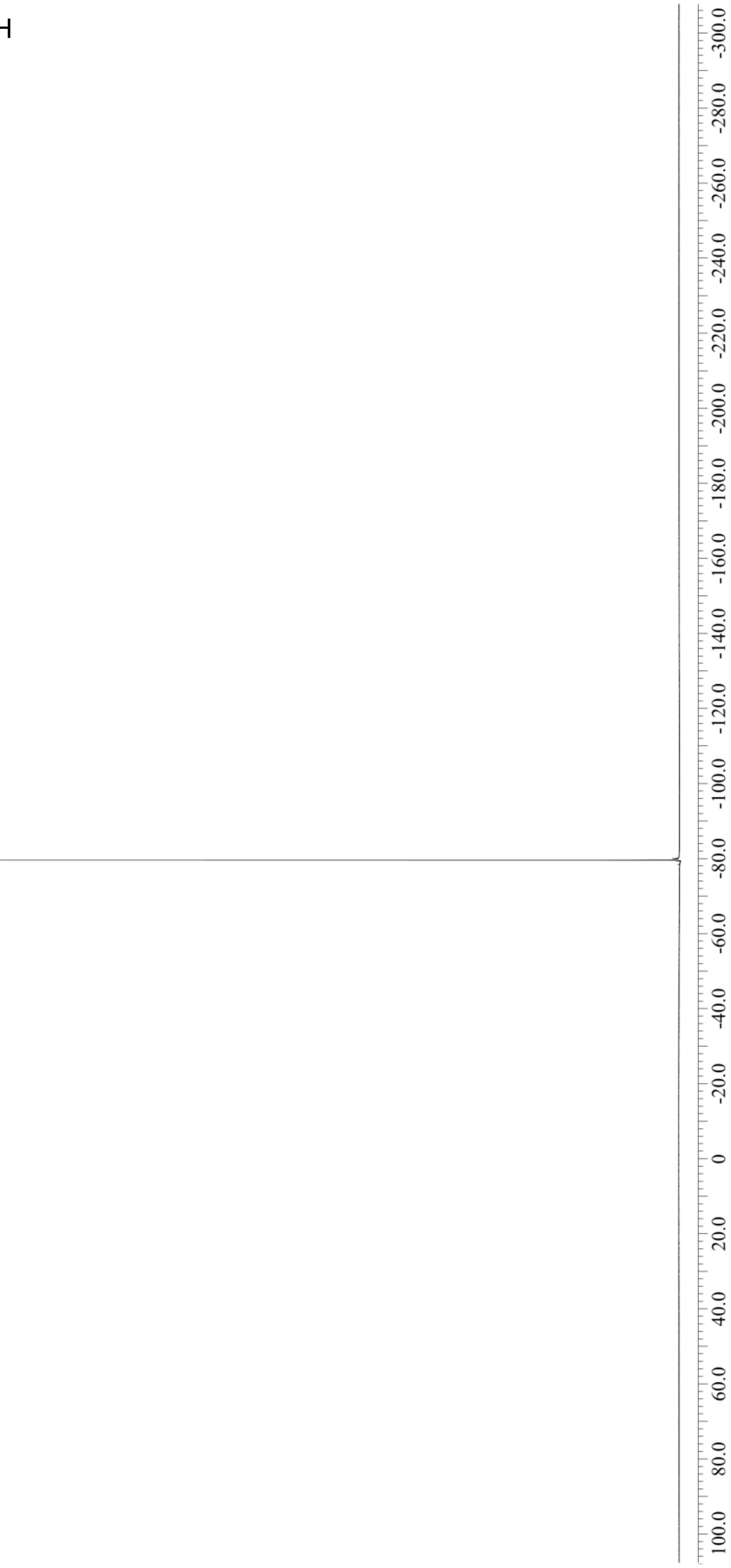

^{1}H NMR of **9k** ($CDCl_3$, 400 MHz, 25 °C)

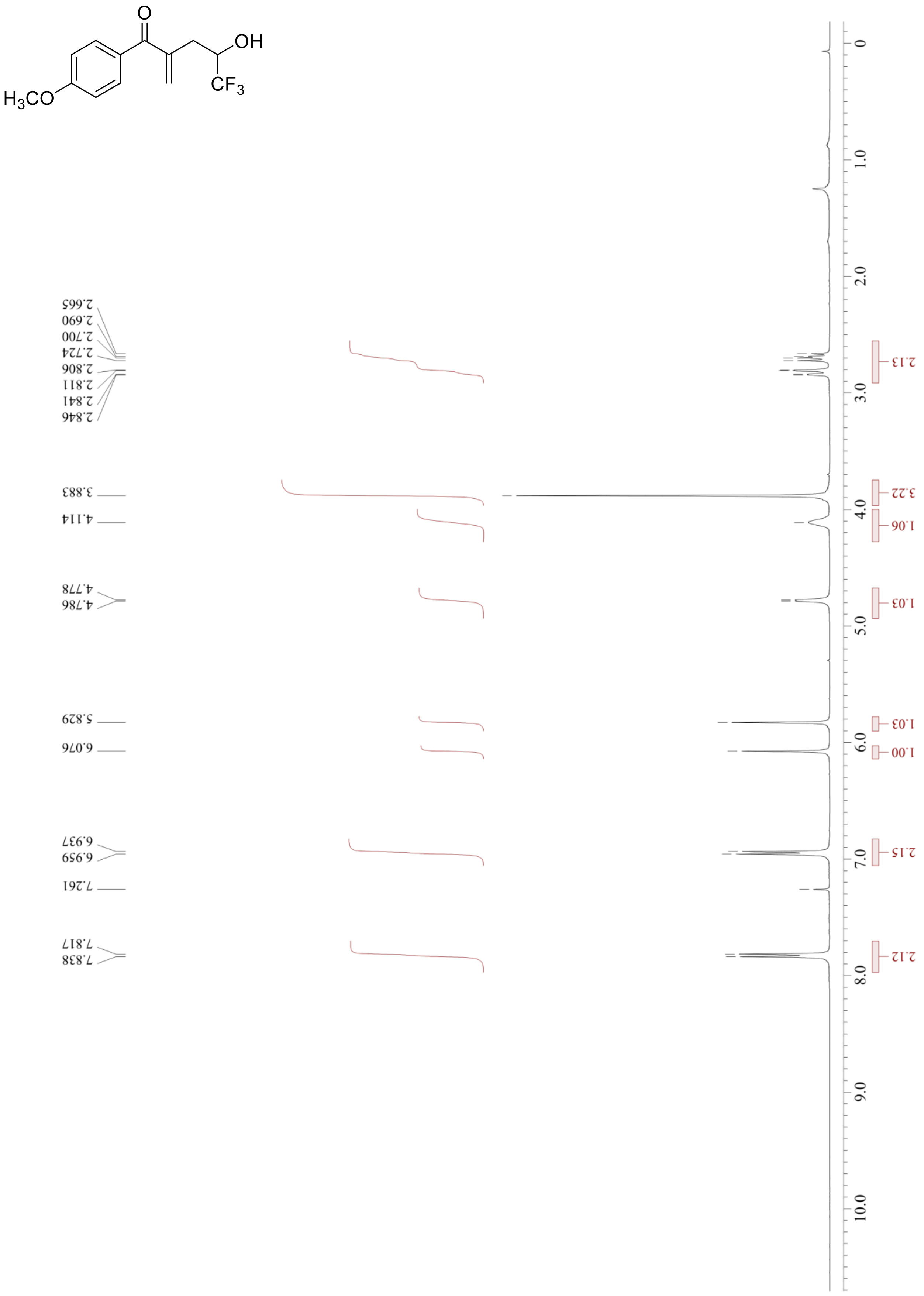

^{13}C NMR of **9k** ($CDCl_3$, 100 MHz, 25 ºC)

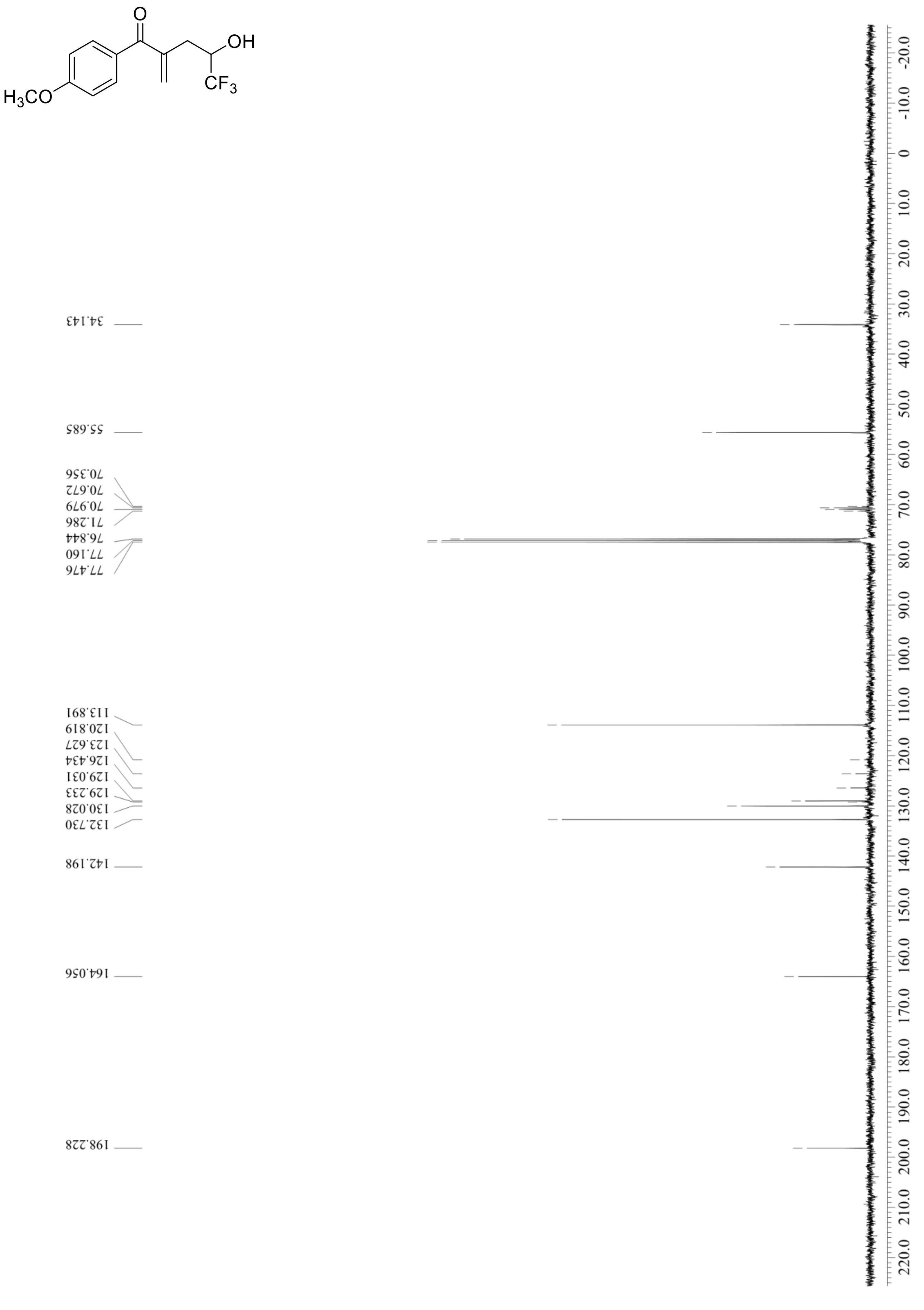

^{19}F NMR of **9k** ($CDCl_3$, 375 MHz, 25 ºC)

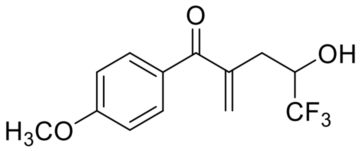

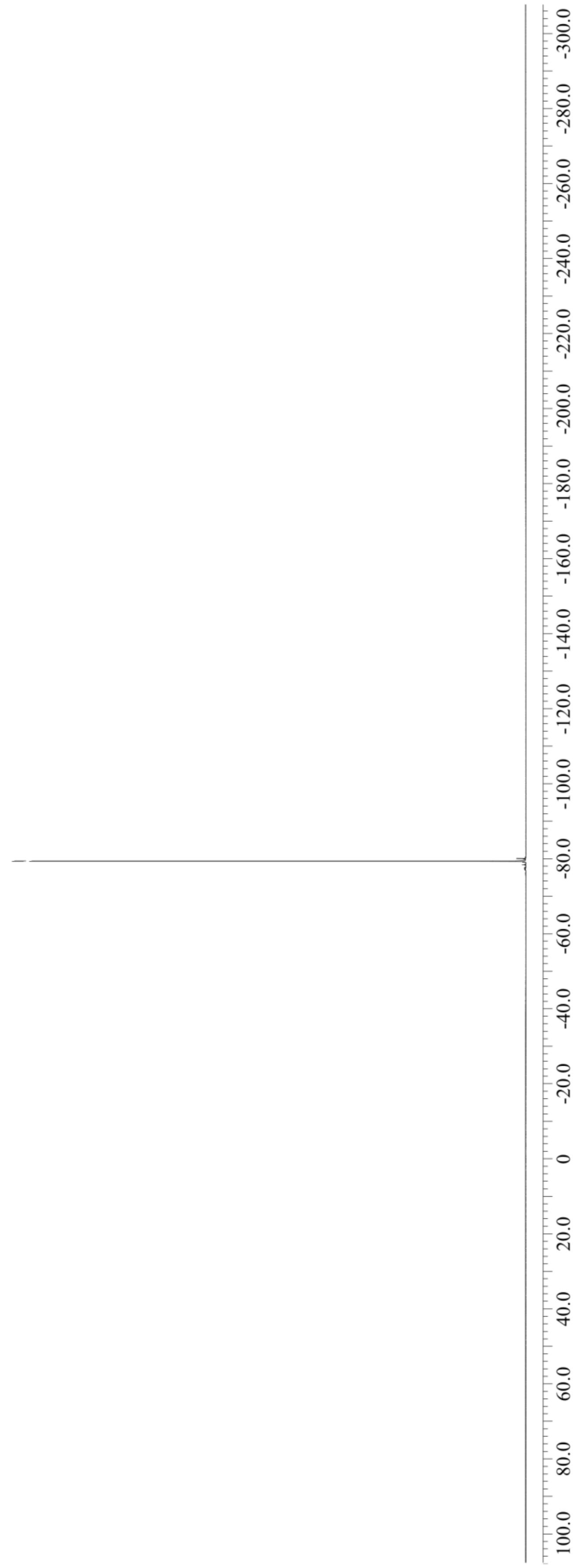

^{1}H NMR of **9l** ($CDCl_3$, 400 MHz, 25 ºC)

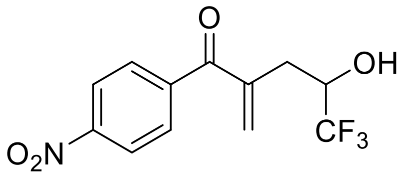

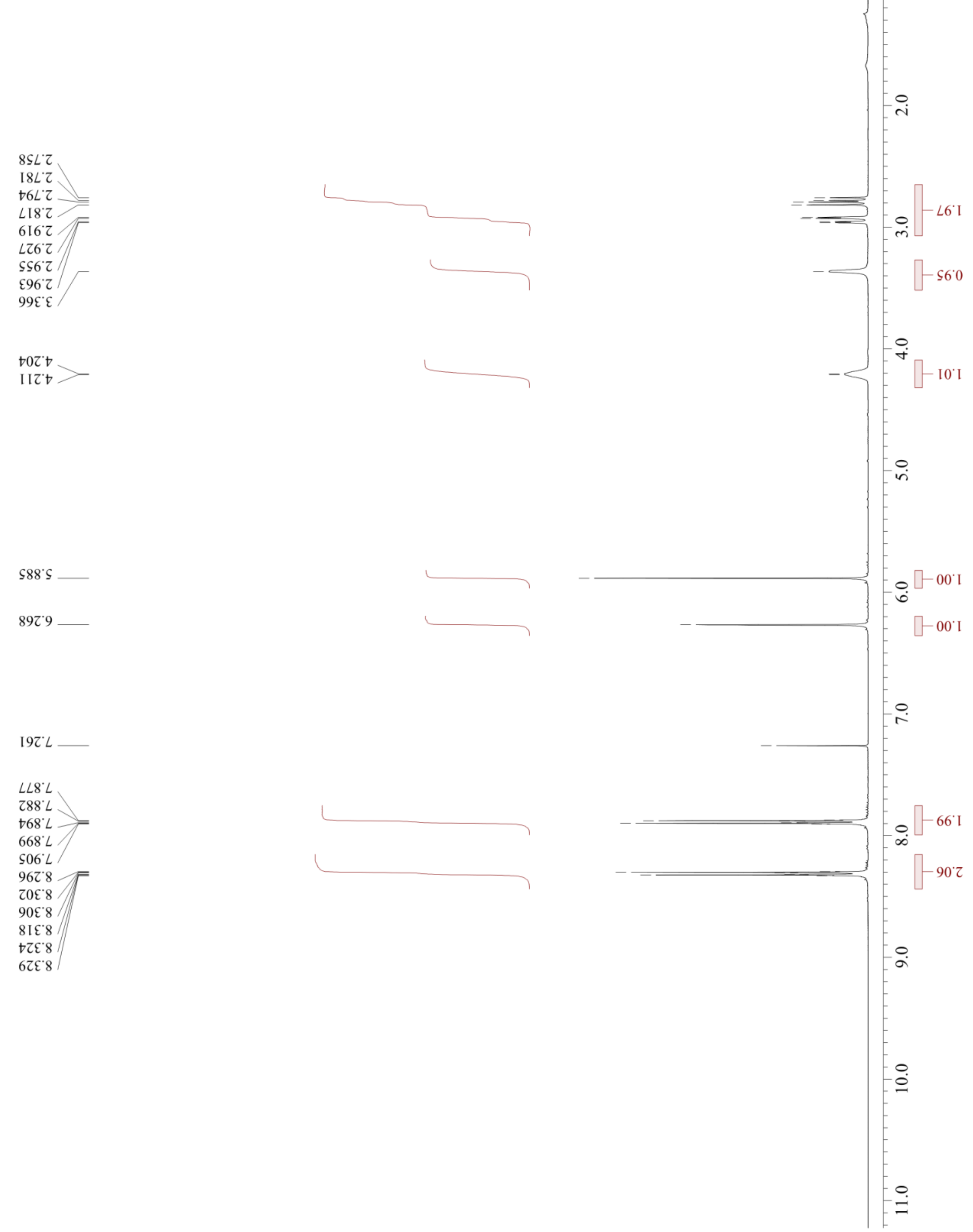

^{13}C NMR of **9l** ($CDCl_3$, 100 MHz, 25 °C)

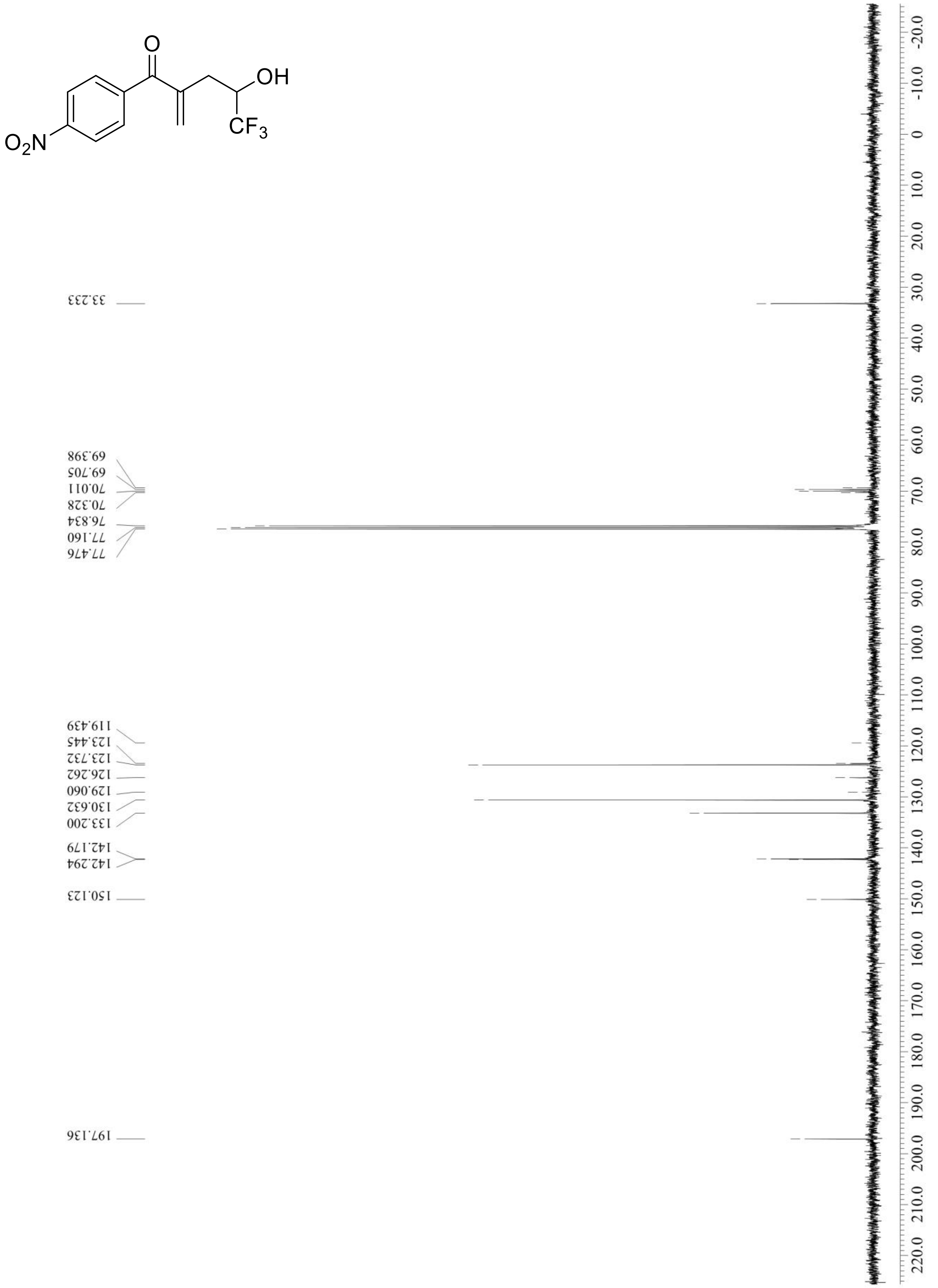

^{19}F NMR of **9l** ($CDCl_3$, 375 MHz, 25 ºC)

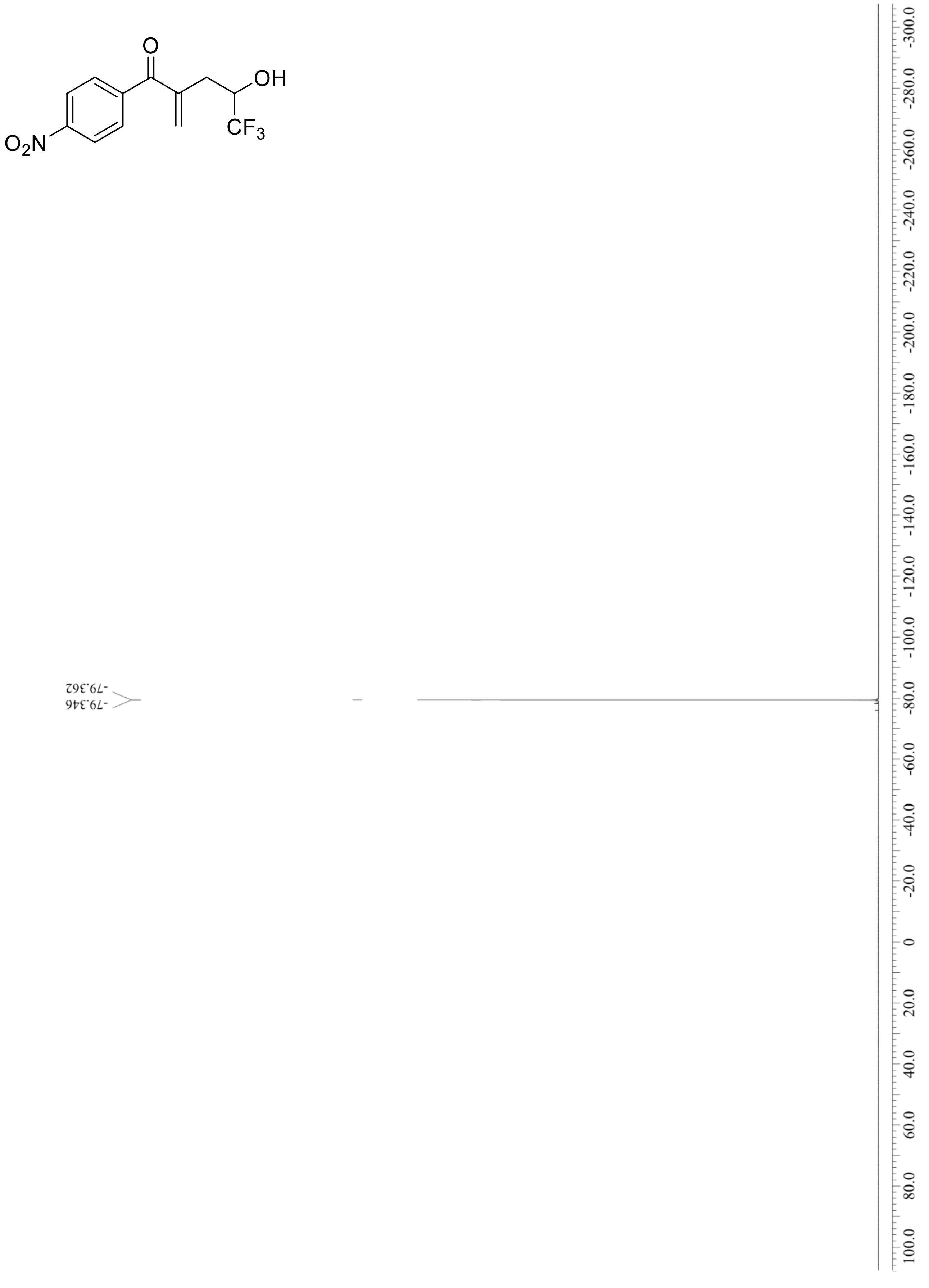

^{1}H NMR of **9m** ($CDCl_3$, 400 MHz, 25 ºC)

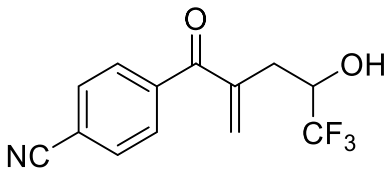

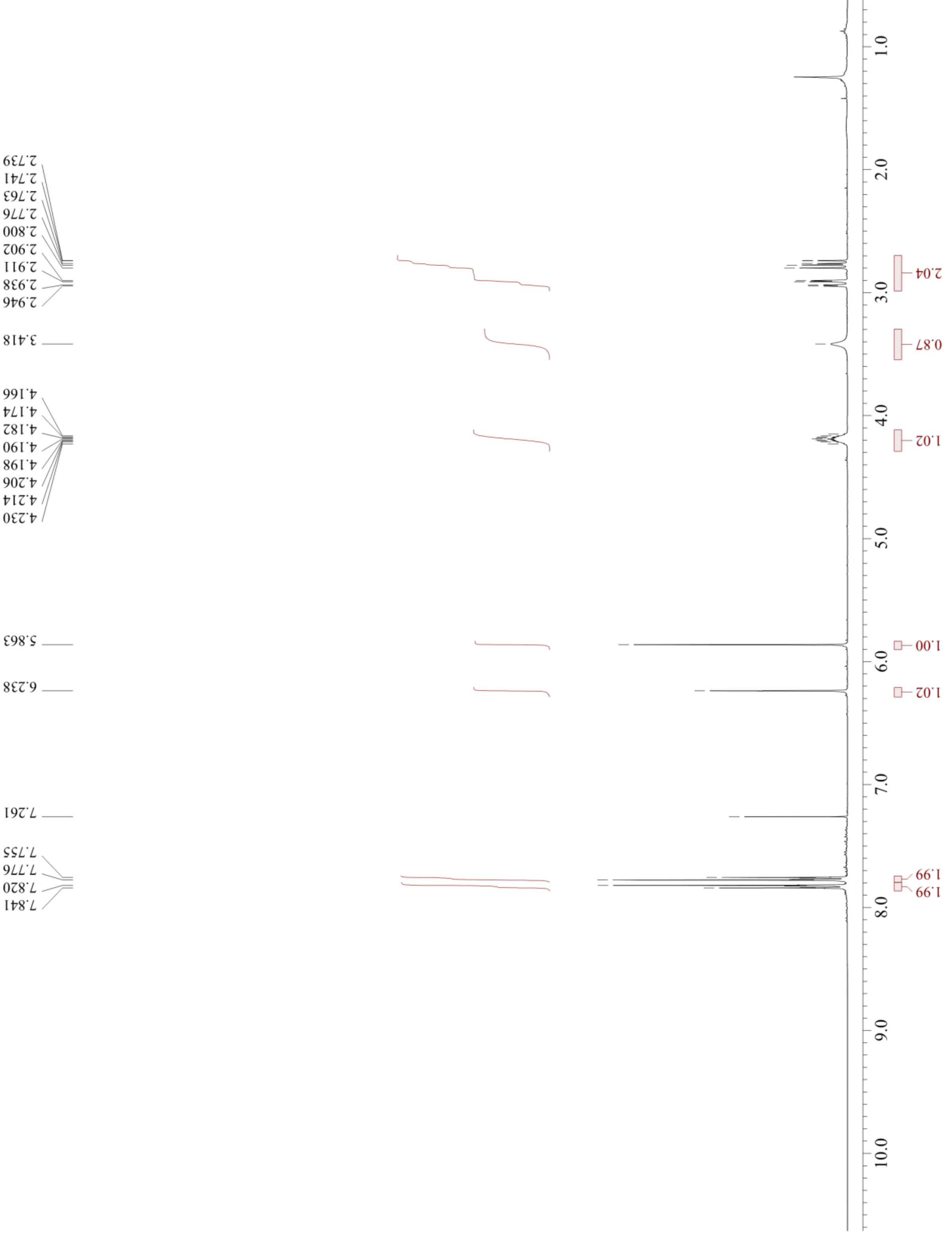

^{13}C NMR of **9m** ($CDCl_3$, 100 MHz, 25 ºC)

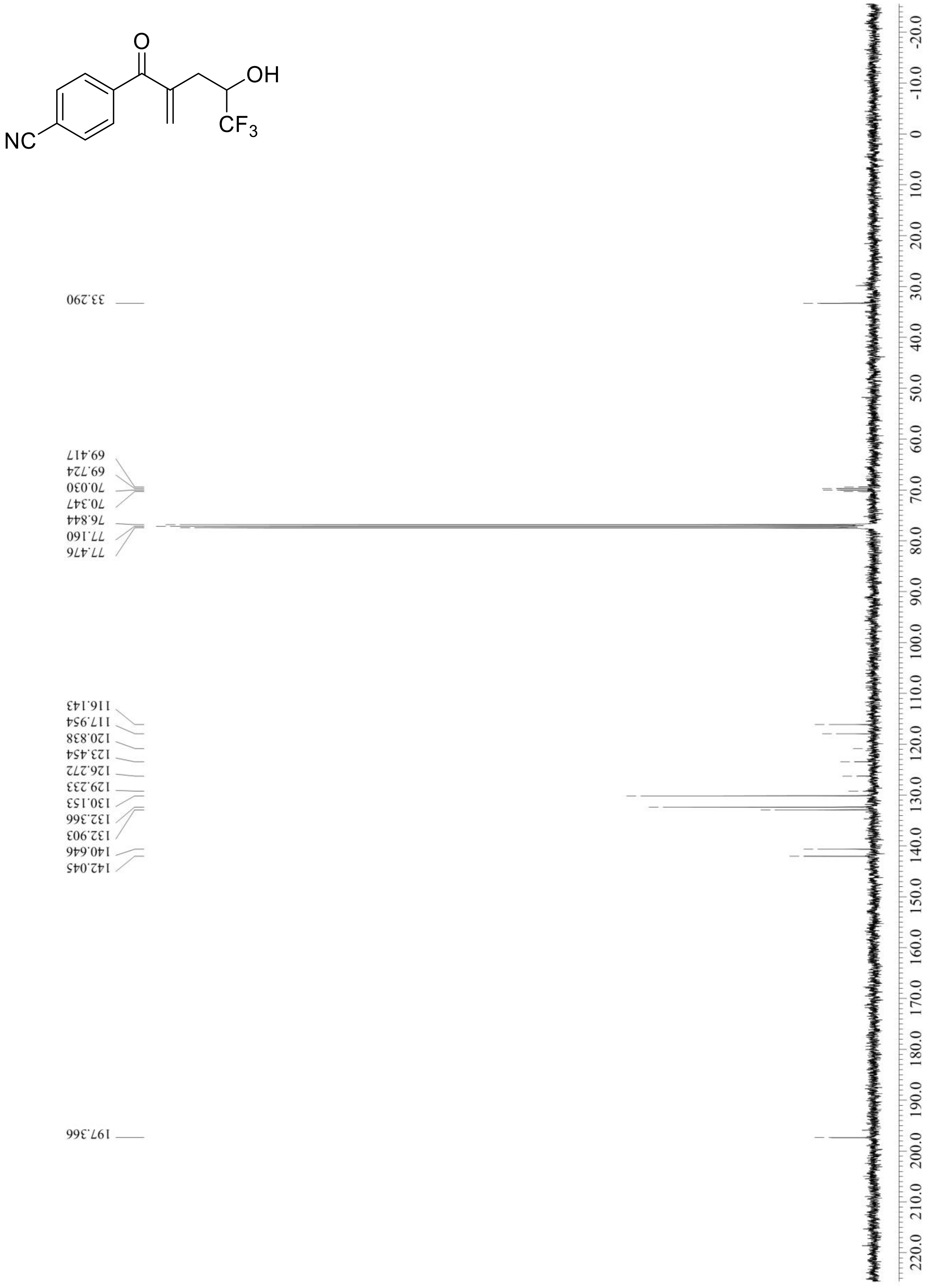

^{19}F NMR of **9m** ($CDCl_3$, 375 MHz, 25 ºC)

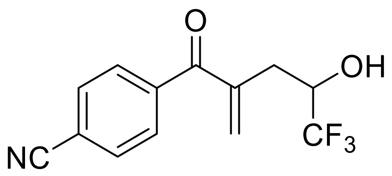

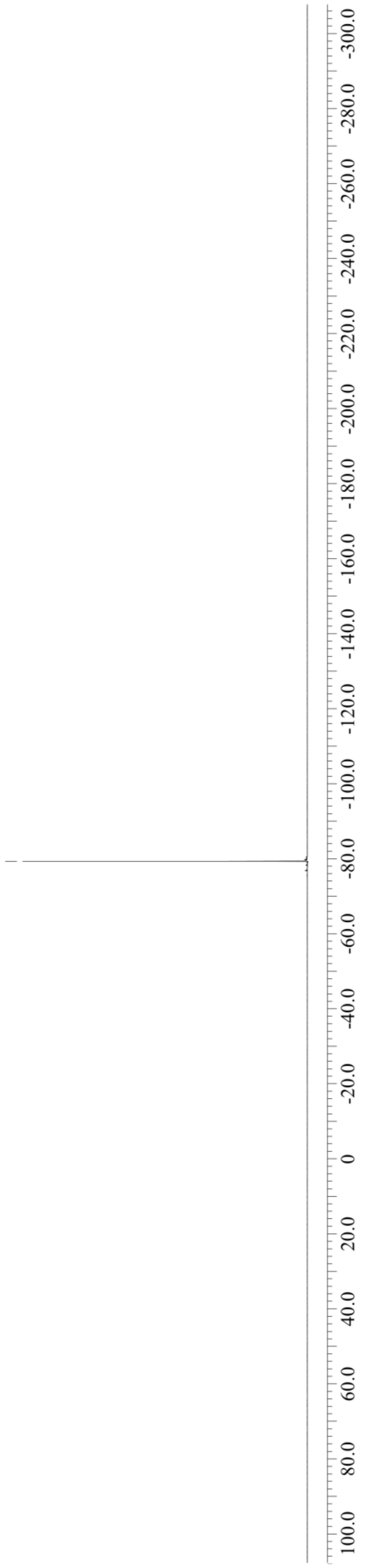

^1H NMR of **9n** ($CDCl_3$, 400 MHz, 25 ºC)

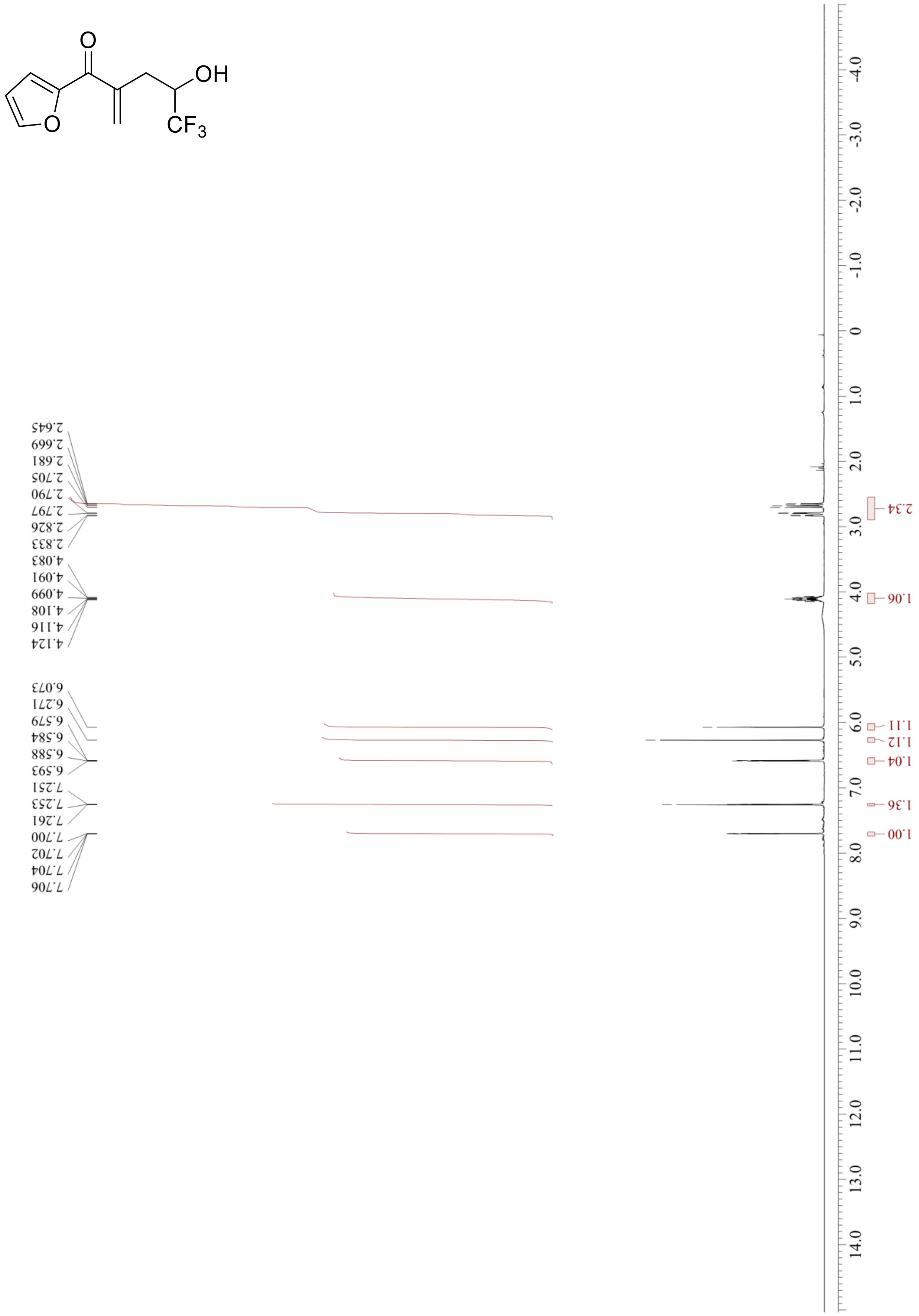

^{13}C NMR of **9n** ($CDCl_3$, 100 MHz, 25 ºC)

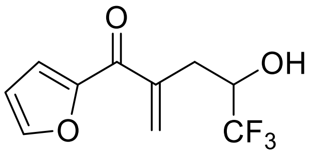

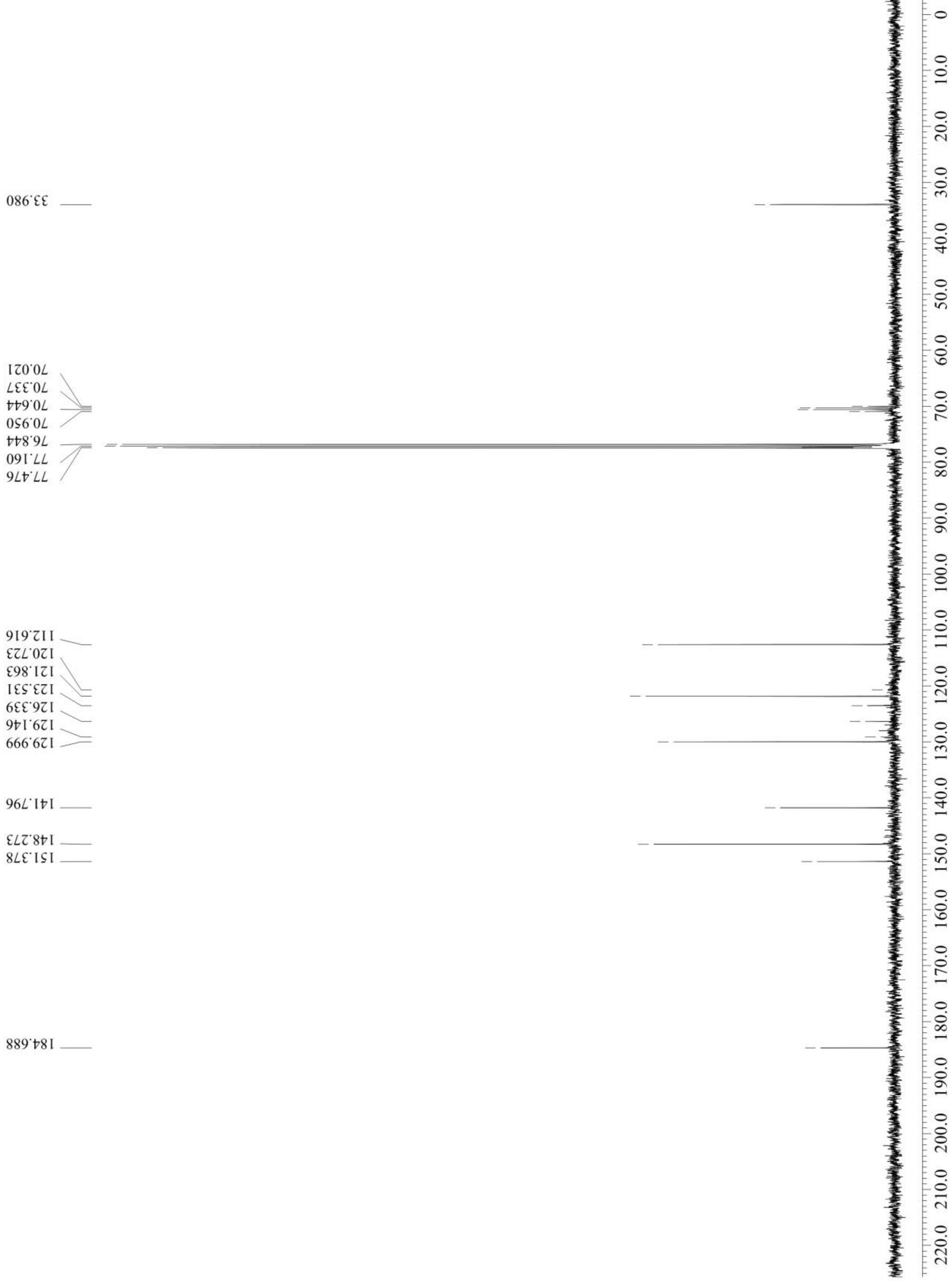

^{19}F NMR of **9n** ($CDCl_3$, 375 MHz, 25 ºC)

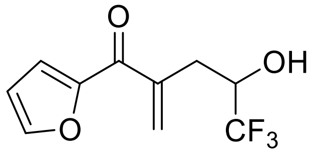

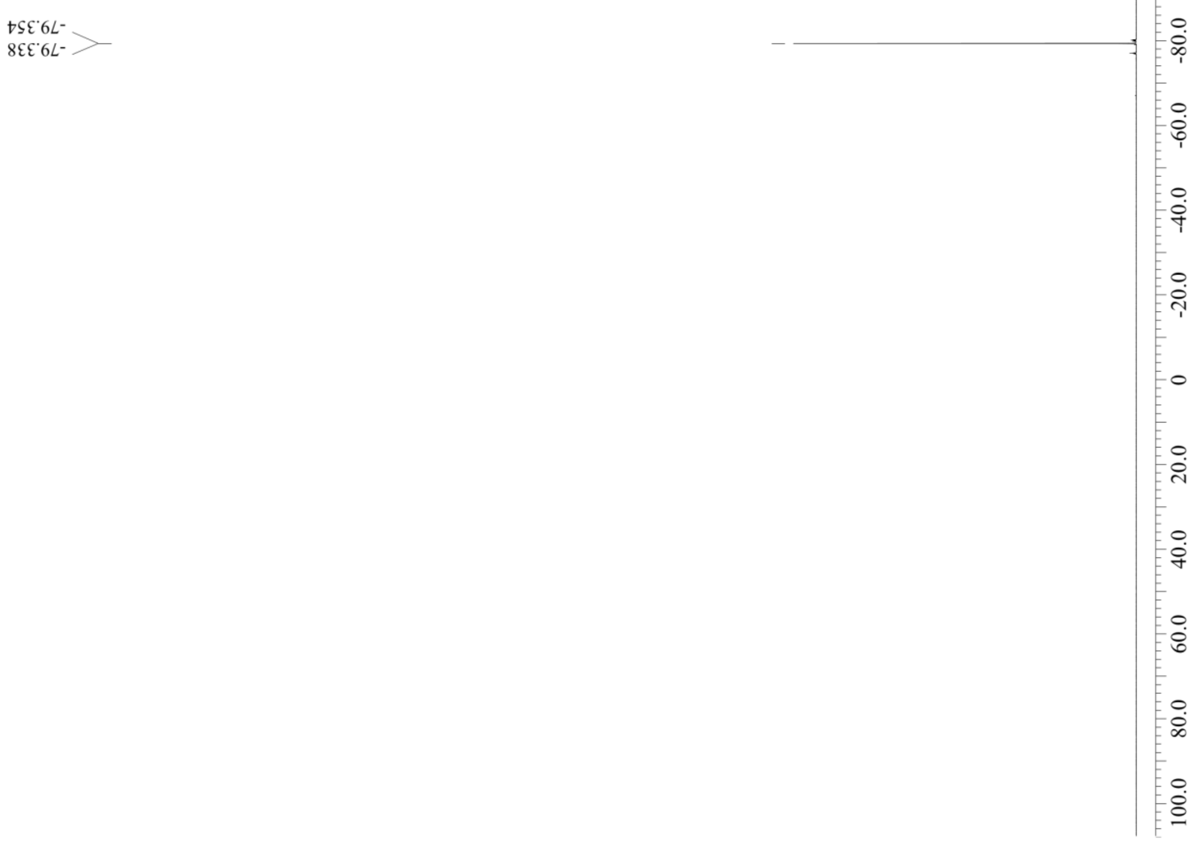

^{1}H NMR of **9o** ($CDCl_3$, 400 MHz, 25 ºC)

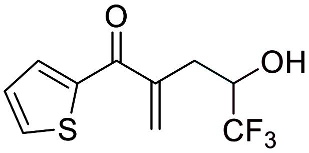

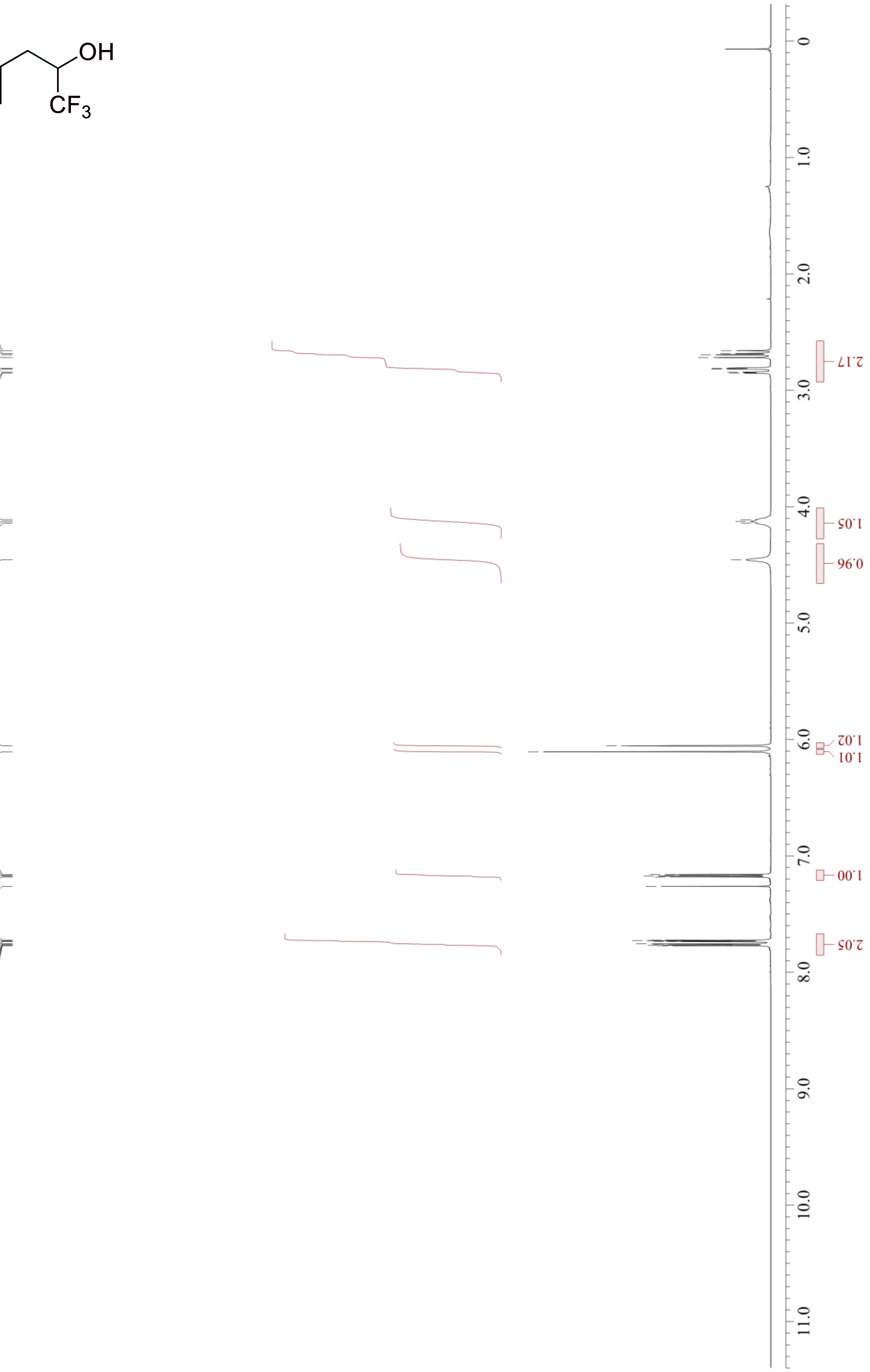

^{13}C NMR of **9o** ($CDCl_3$, 100 MHz, 25 ºC)

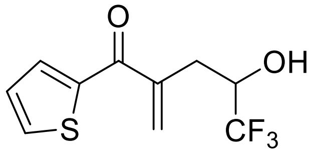

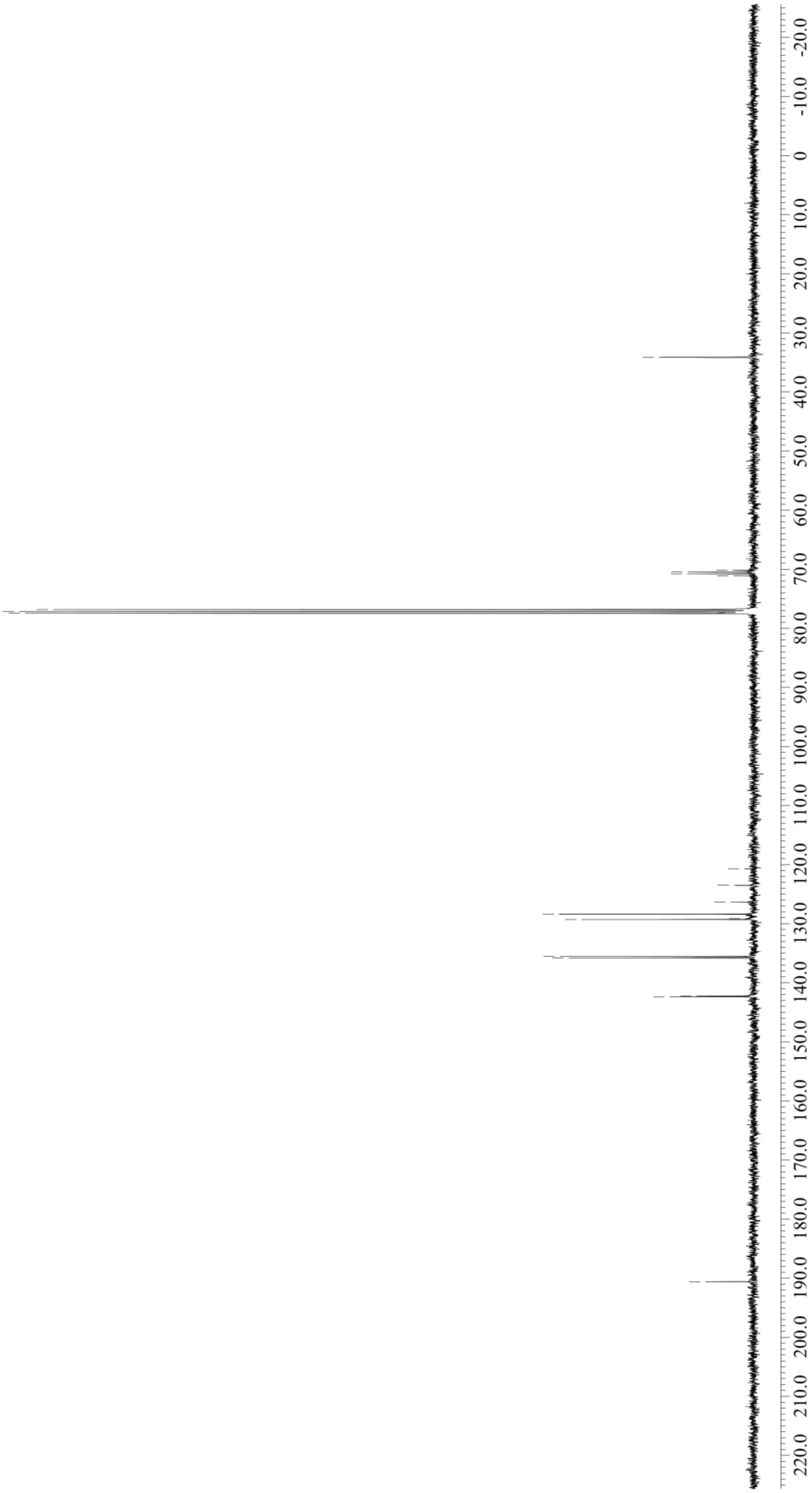

^{19}F NMR of **9o** ($CDCl_3$, 375 MHz, 25 ºC)

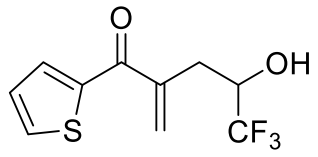

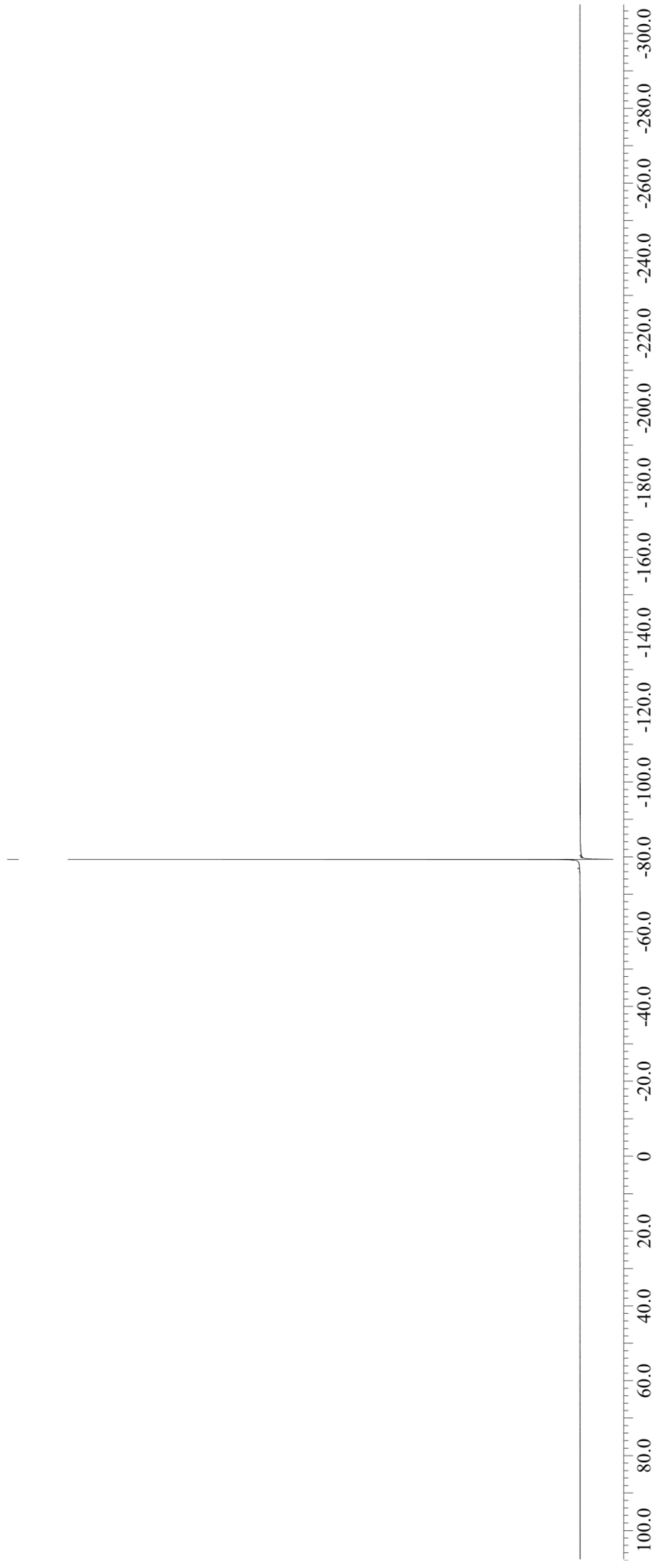

^{1}H NMR of **9p** ($CDCl_3$, 400 MHz, 25 ºC)

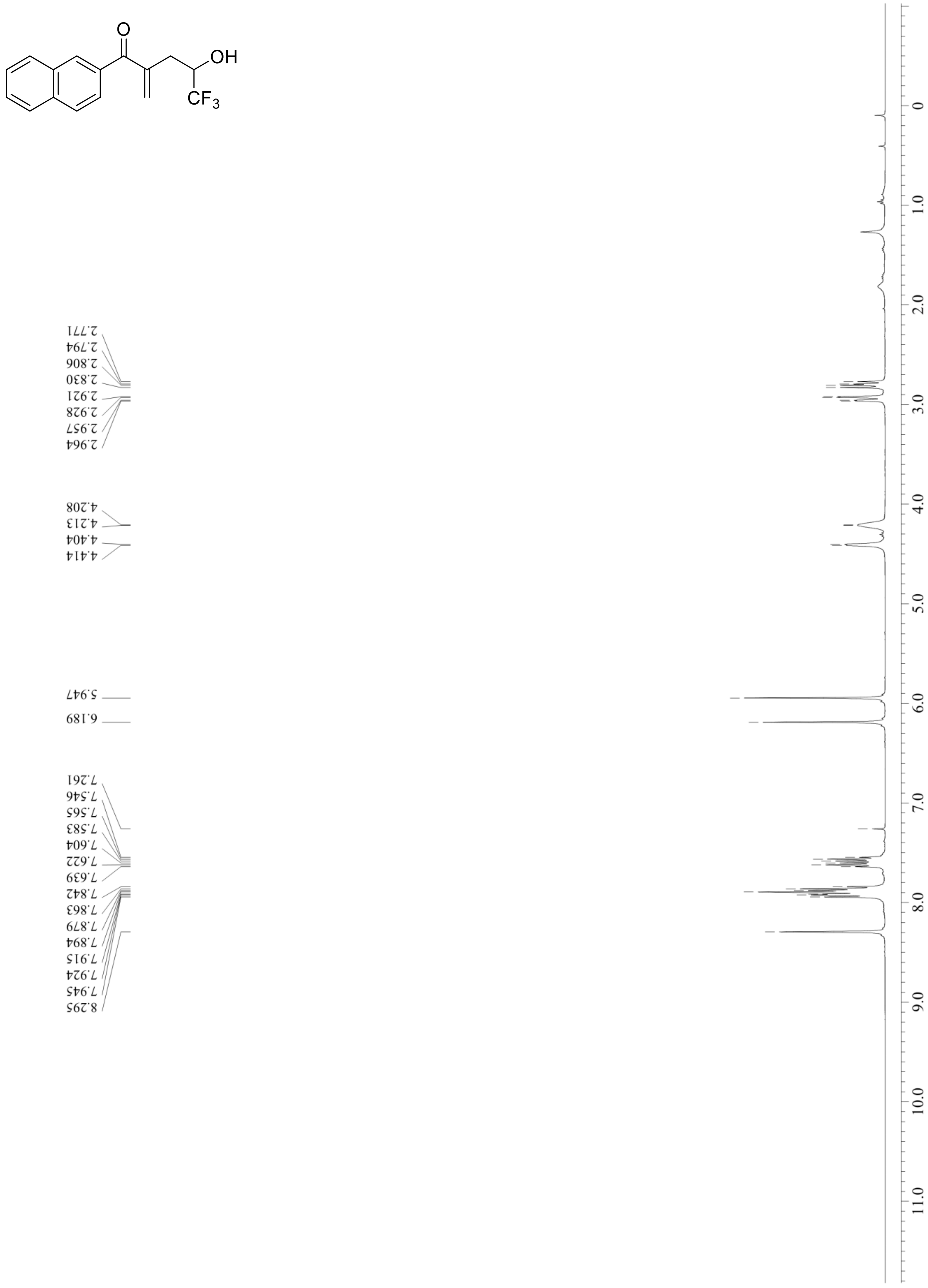

^{13}C NMR of **9p** ($CDCl_3$, 100 MHz, 25 ºC)

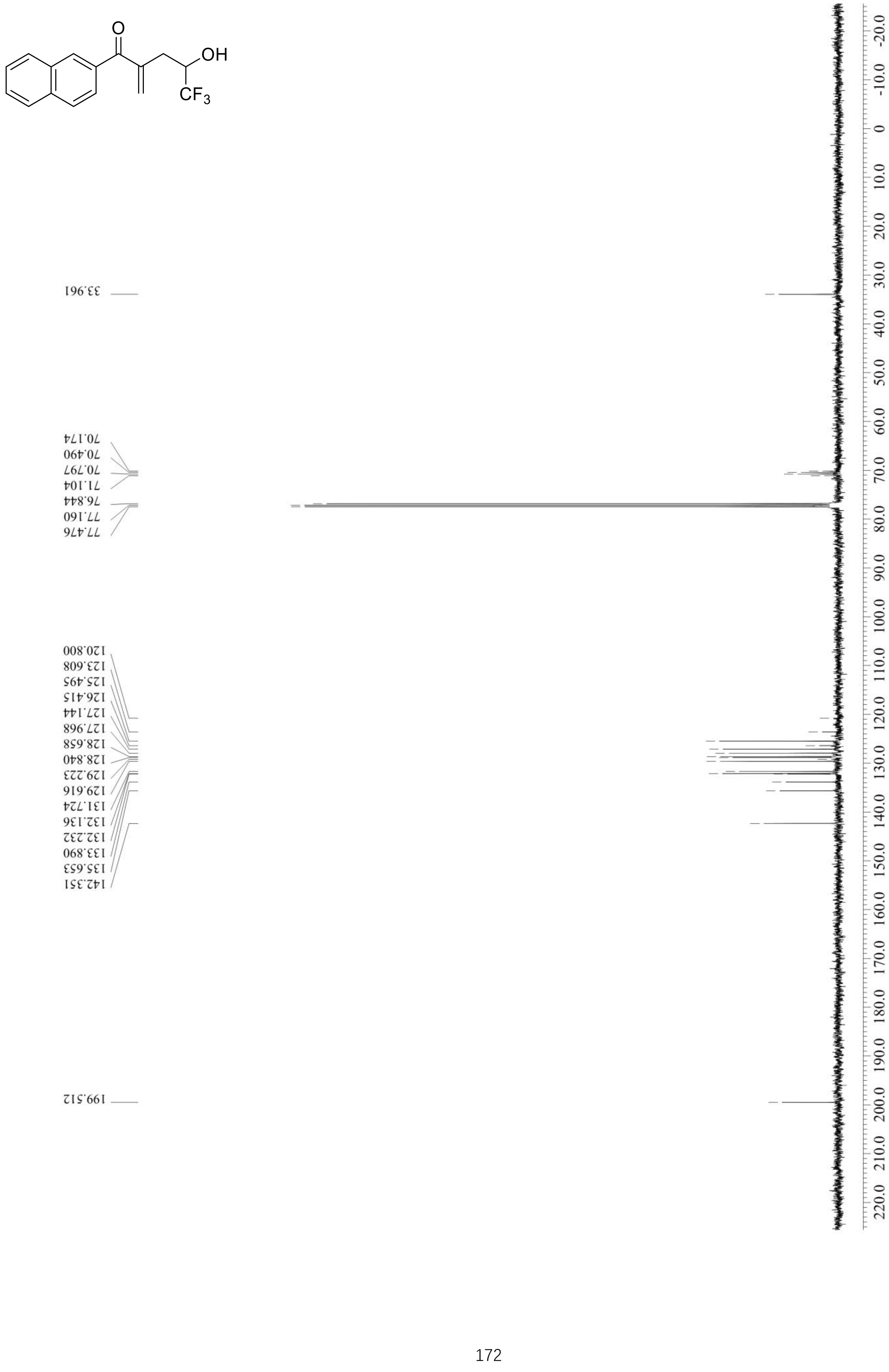

^{19}F NMR of **9p** ($CDCl_3$, 375 MHz, 25 ºC)

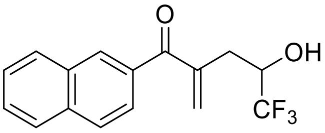

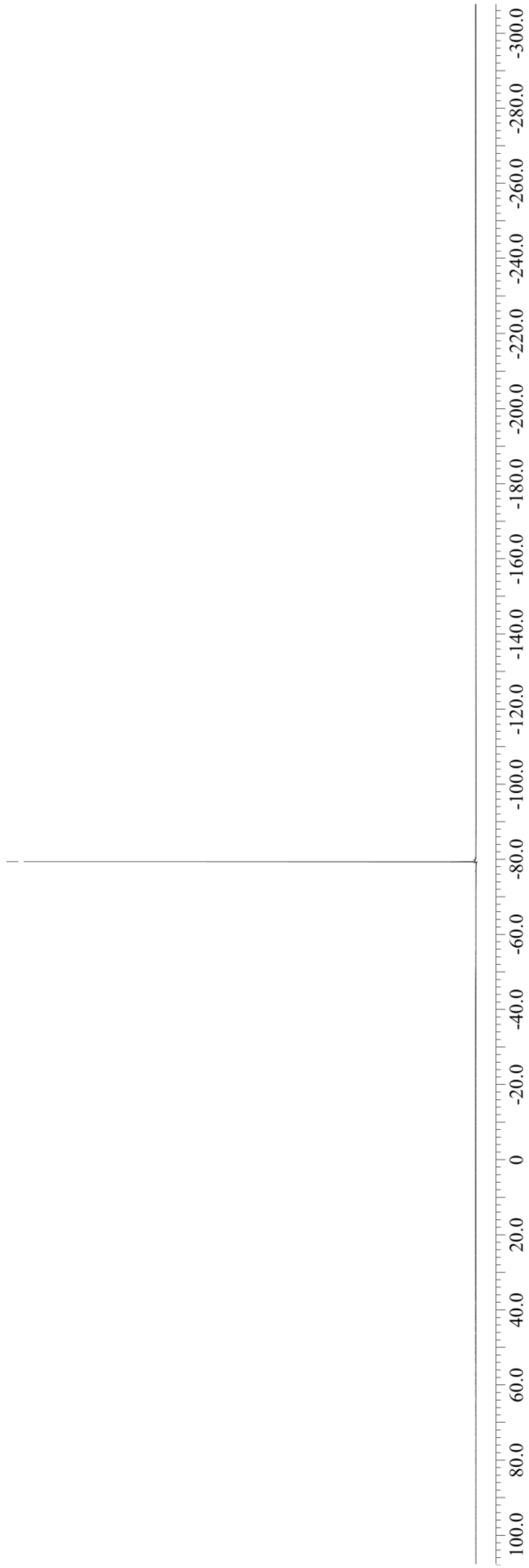

^{1}H NMR of **9q** ($CDCl_3$, 400 MHz, 25 °C)

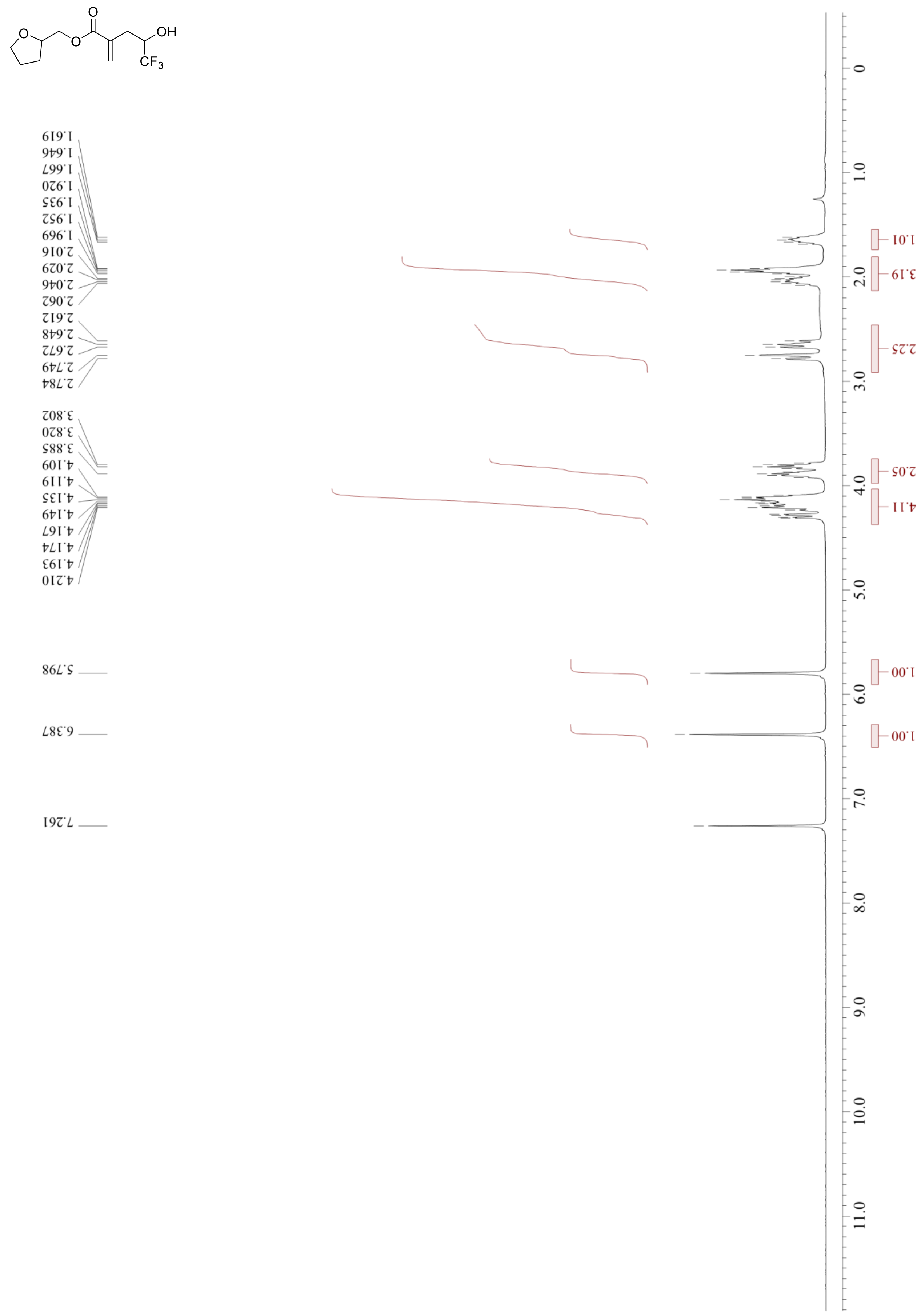

^{13}C NMR of **9q** ($CDCl_3$, 100 MHz, 25 ºC)

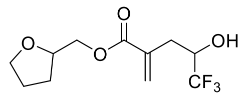

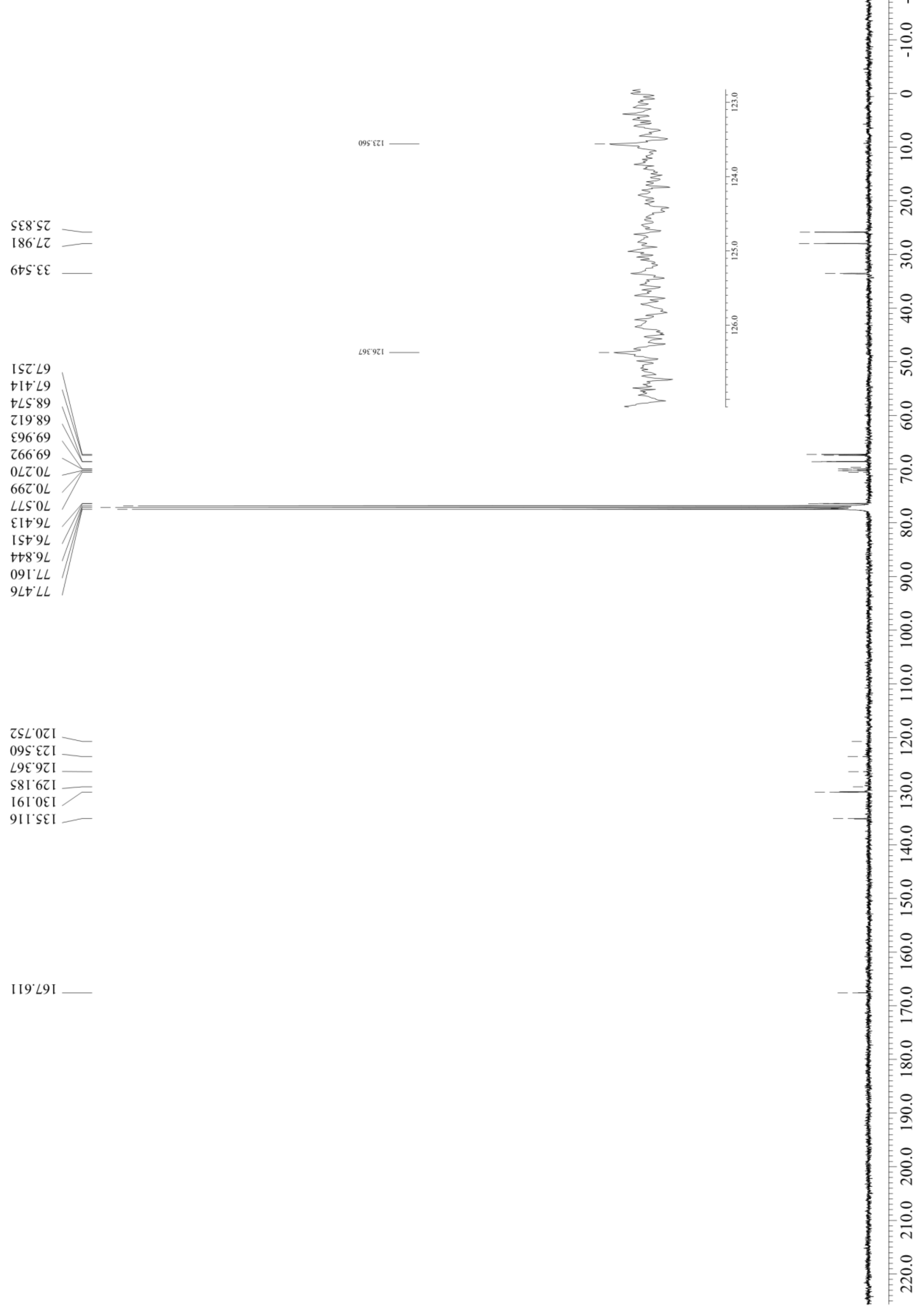

^{19}F NMR of **9q** ($CDCl_3$, 375 MHz, 25 ºC)

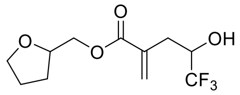

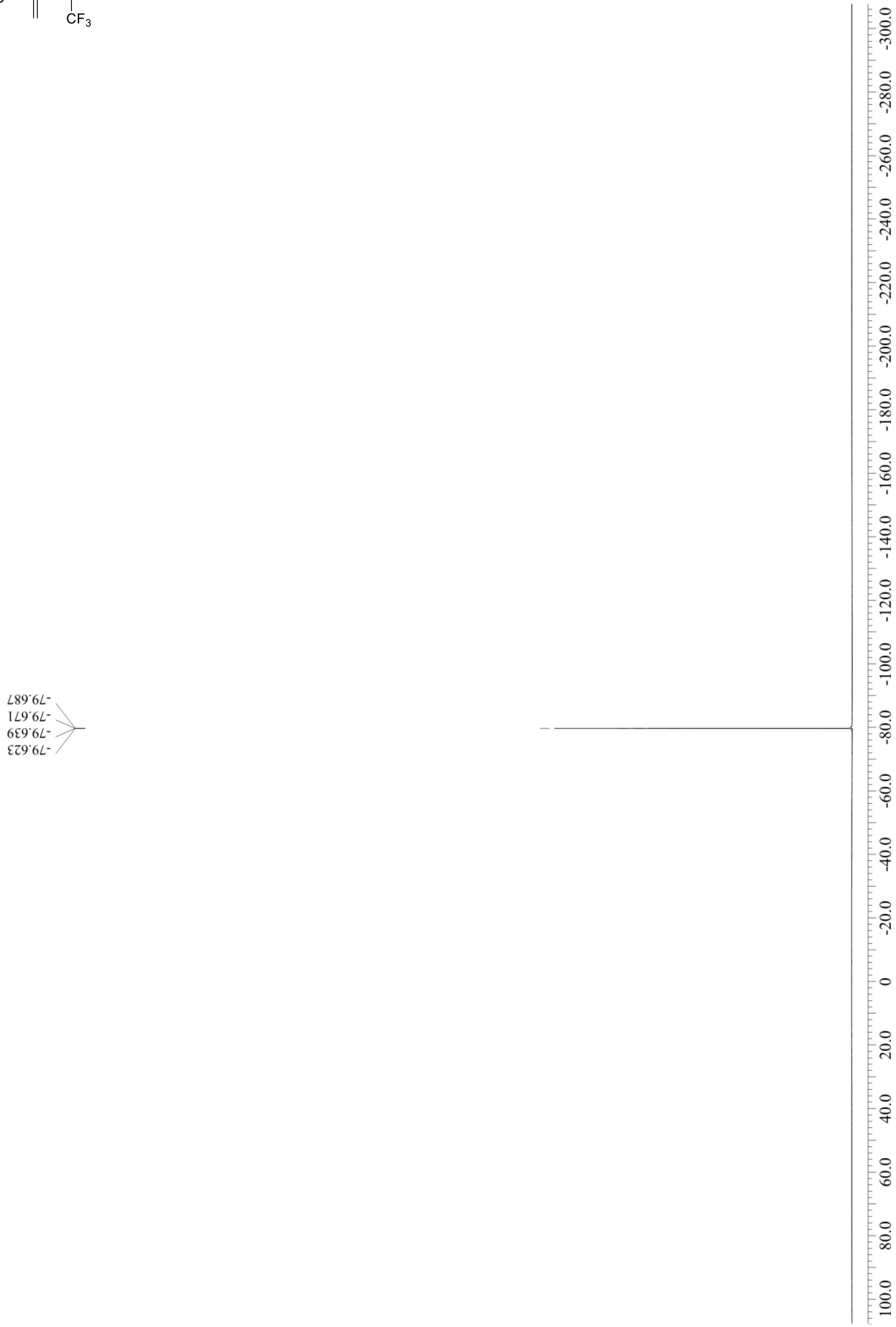

^{1}H NMR of **9r** ($CDCl_3$, 400 MHz, 25 ºC)

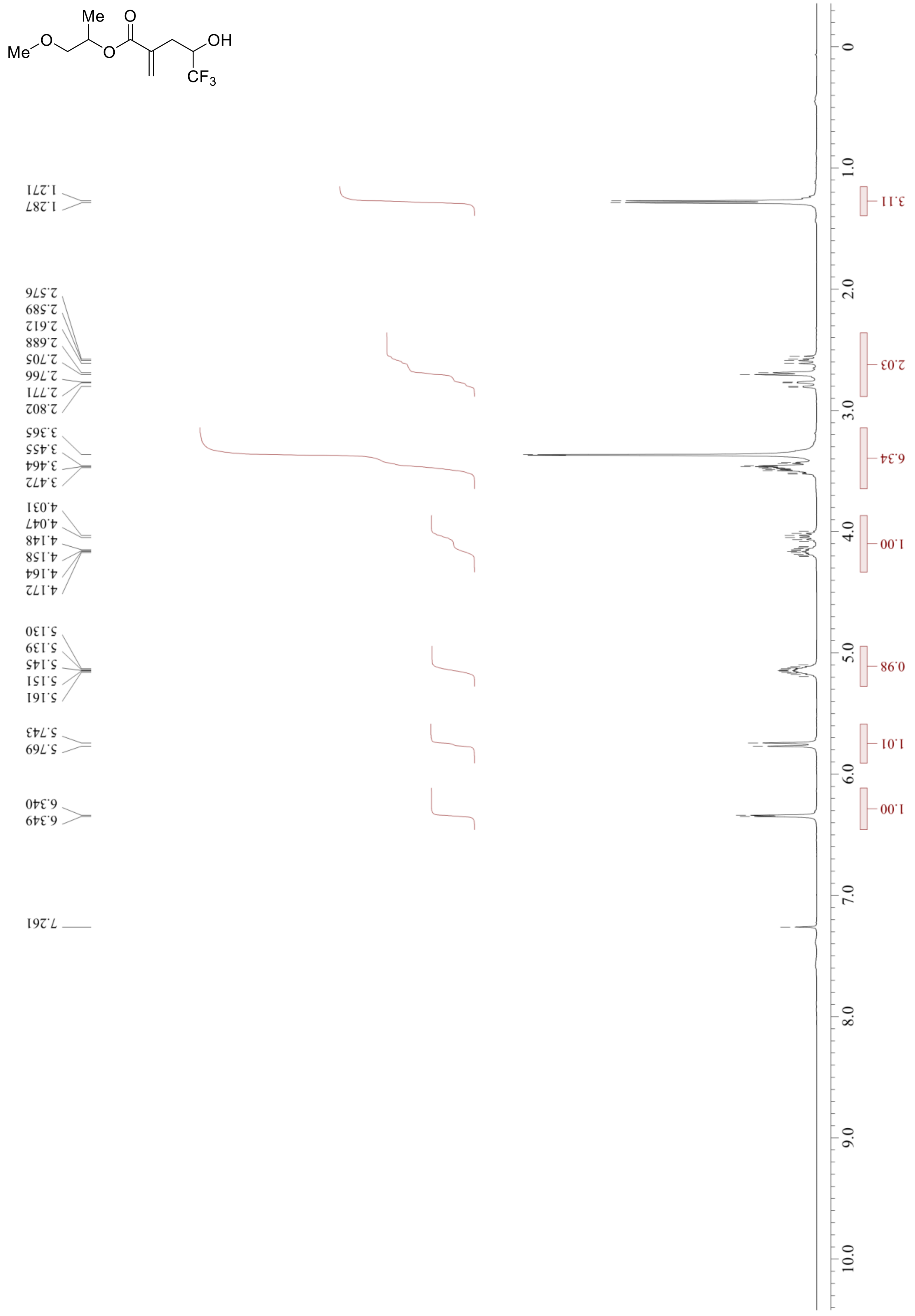

^{13}C NMR of **9r** ($CDCl_3$, 100 MHz, 25 ºC)

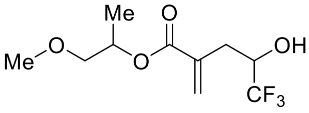

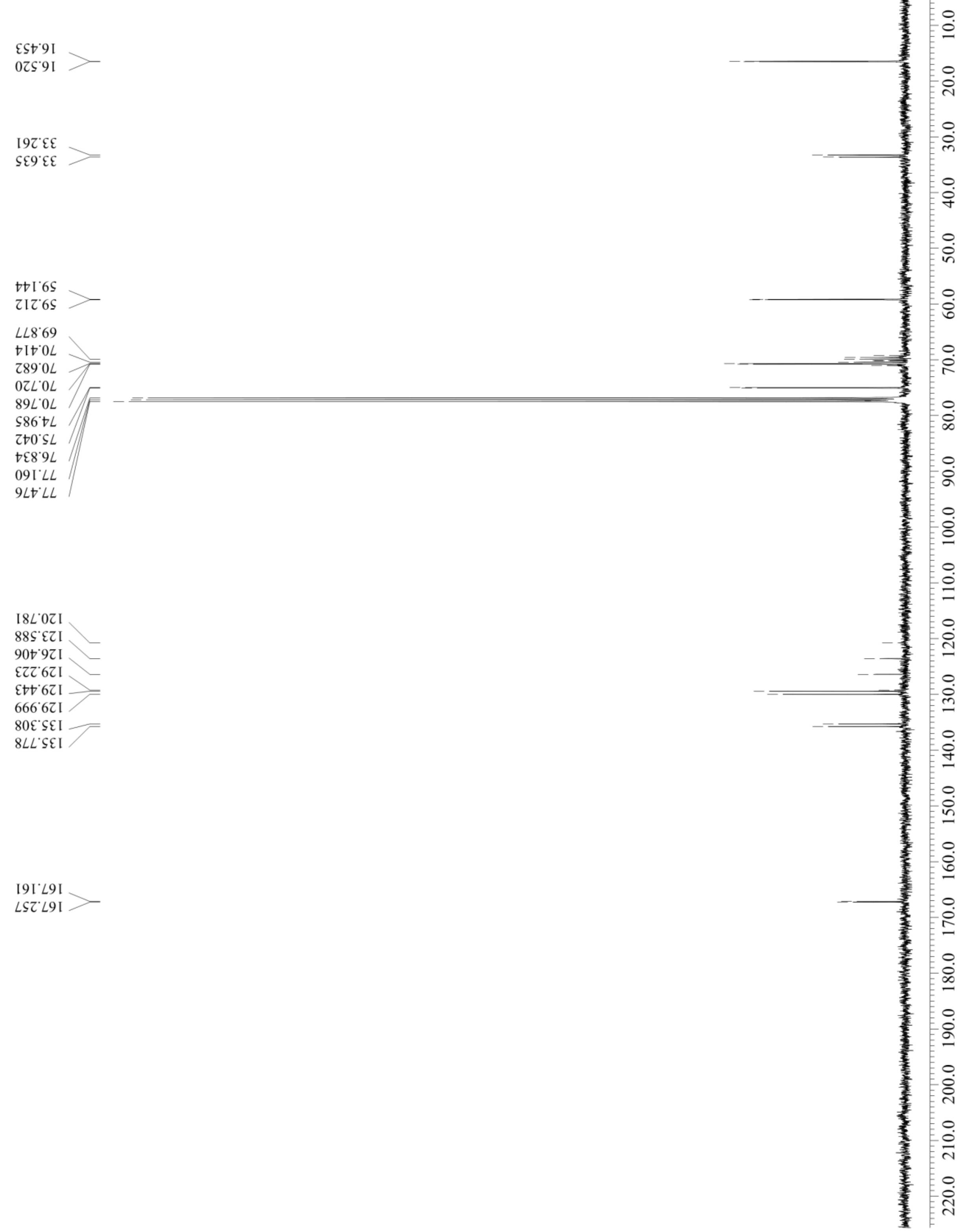

^{19}F NMR of **9r** ($CDCl_3$, 375 MHz, 25 ºC)

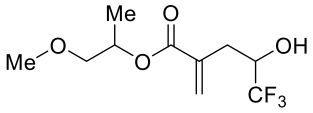

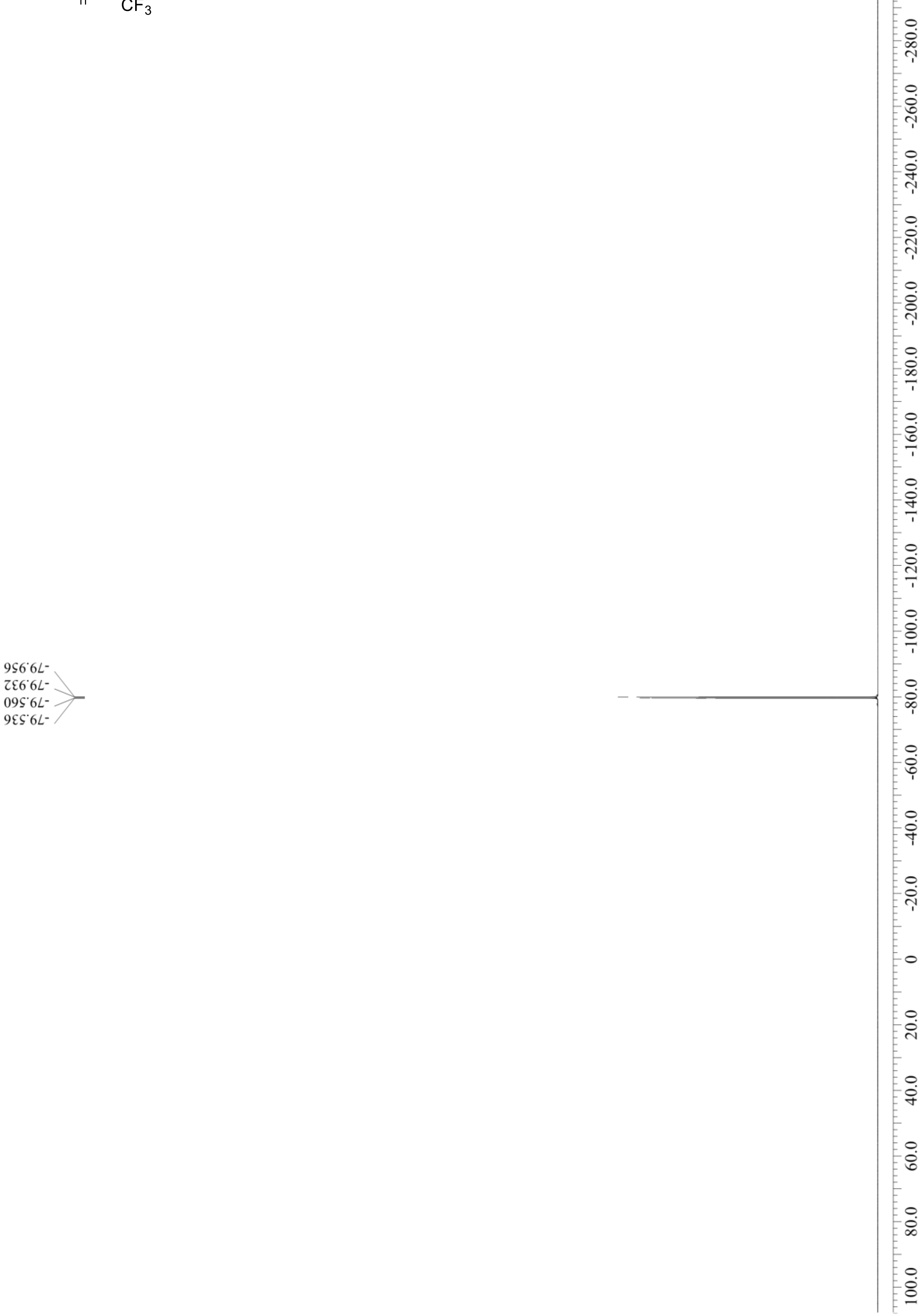

^{1}H NMR of **9s** ($CDCl_3$, 400 MHz, 25 ºC)

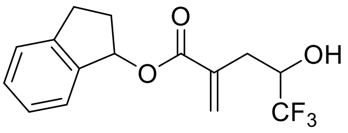

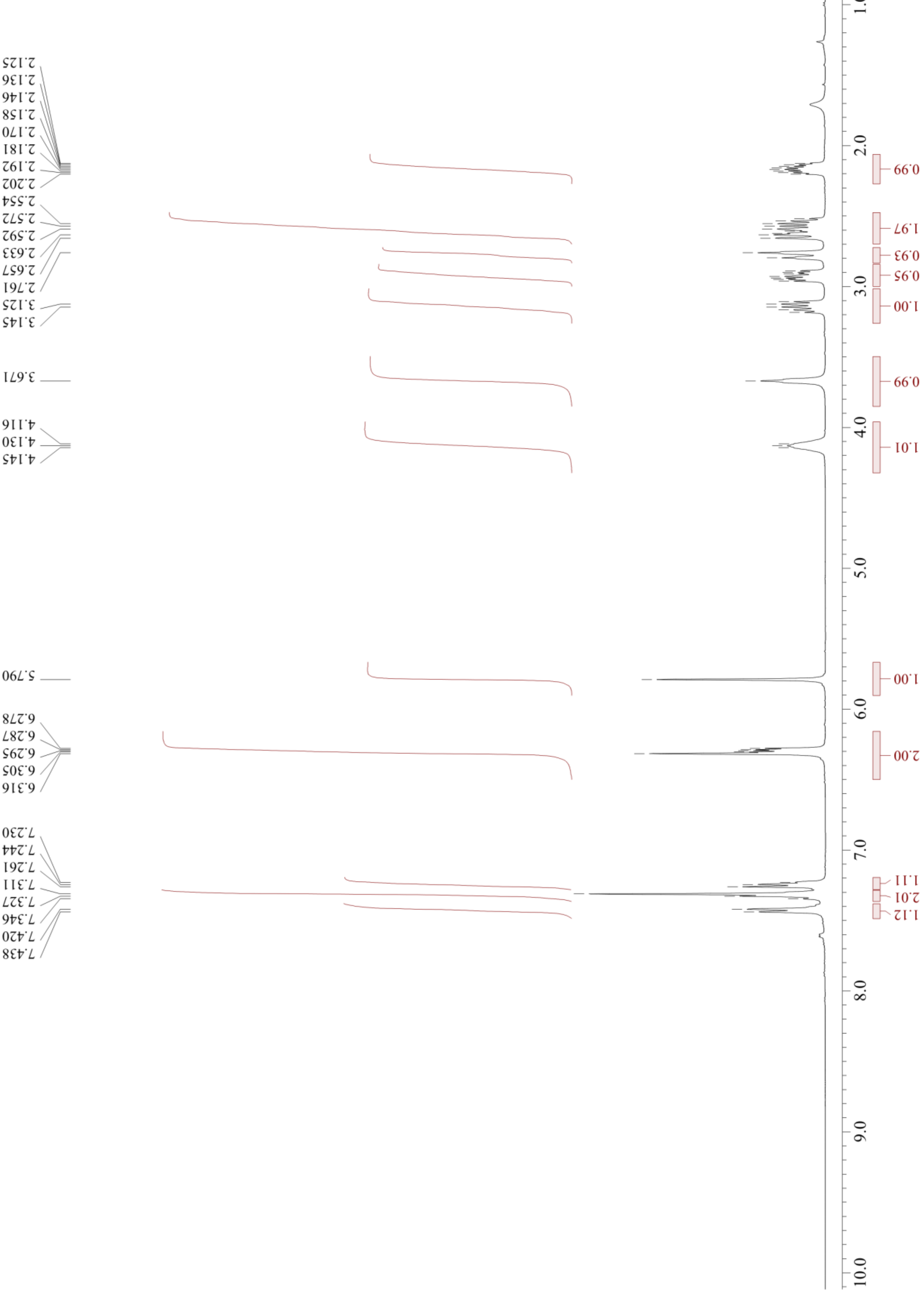

^{13}C NMR of **9s** ($CDCl_3$, 100 MHz, 25 ºC)

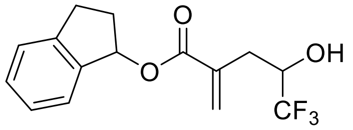

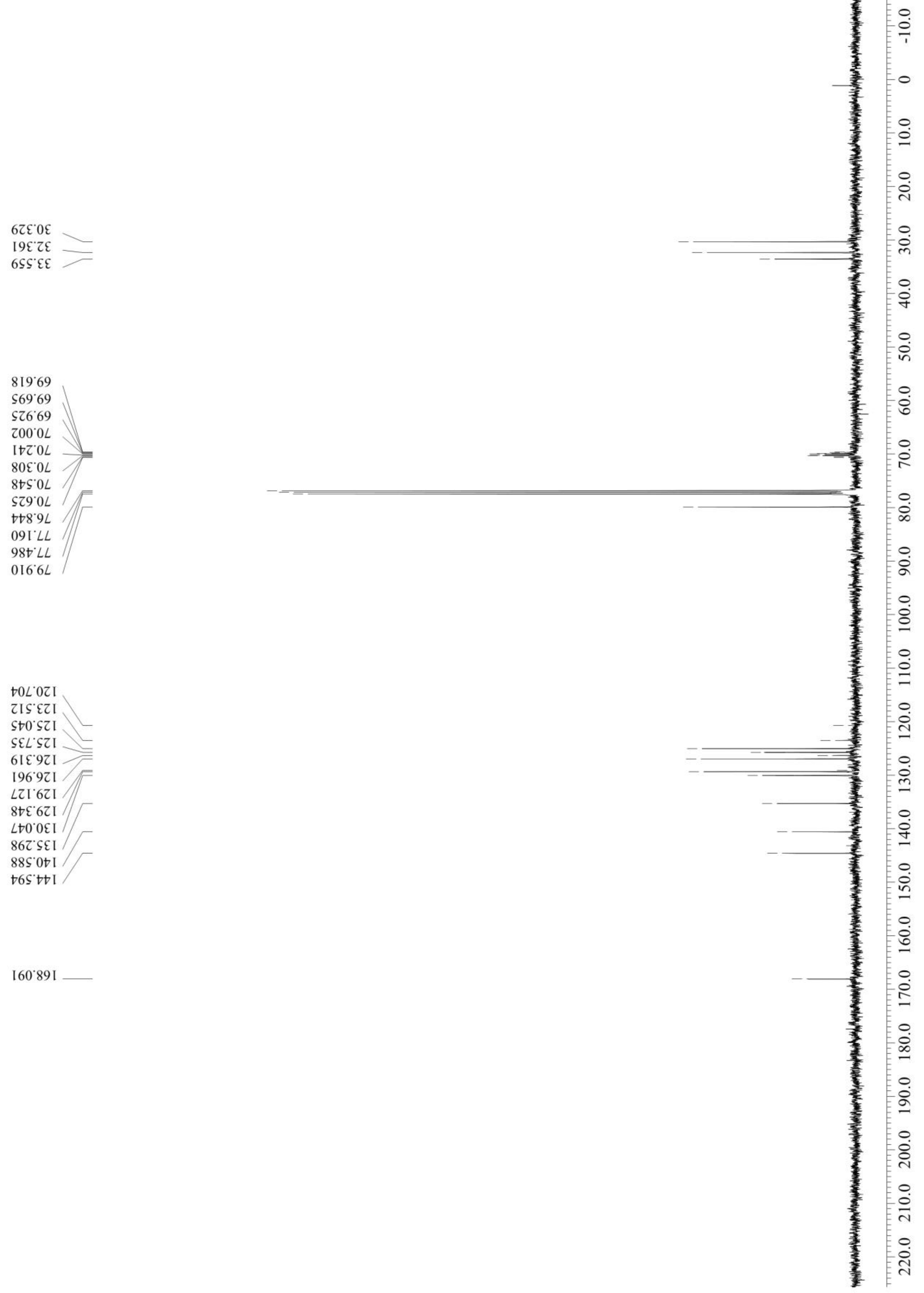

^{19}F NMR of **9s** ($CDCl_3$, 375 MHz, 25 ºC)

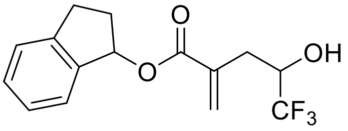

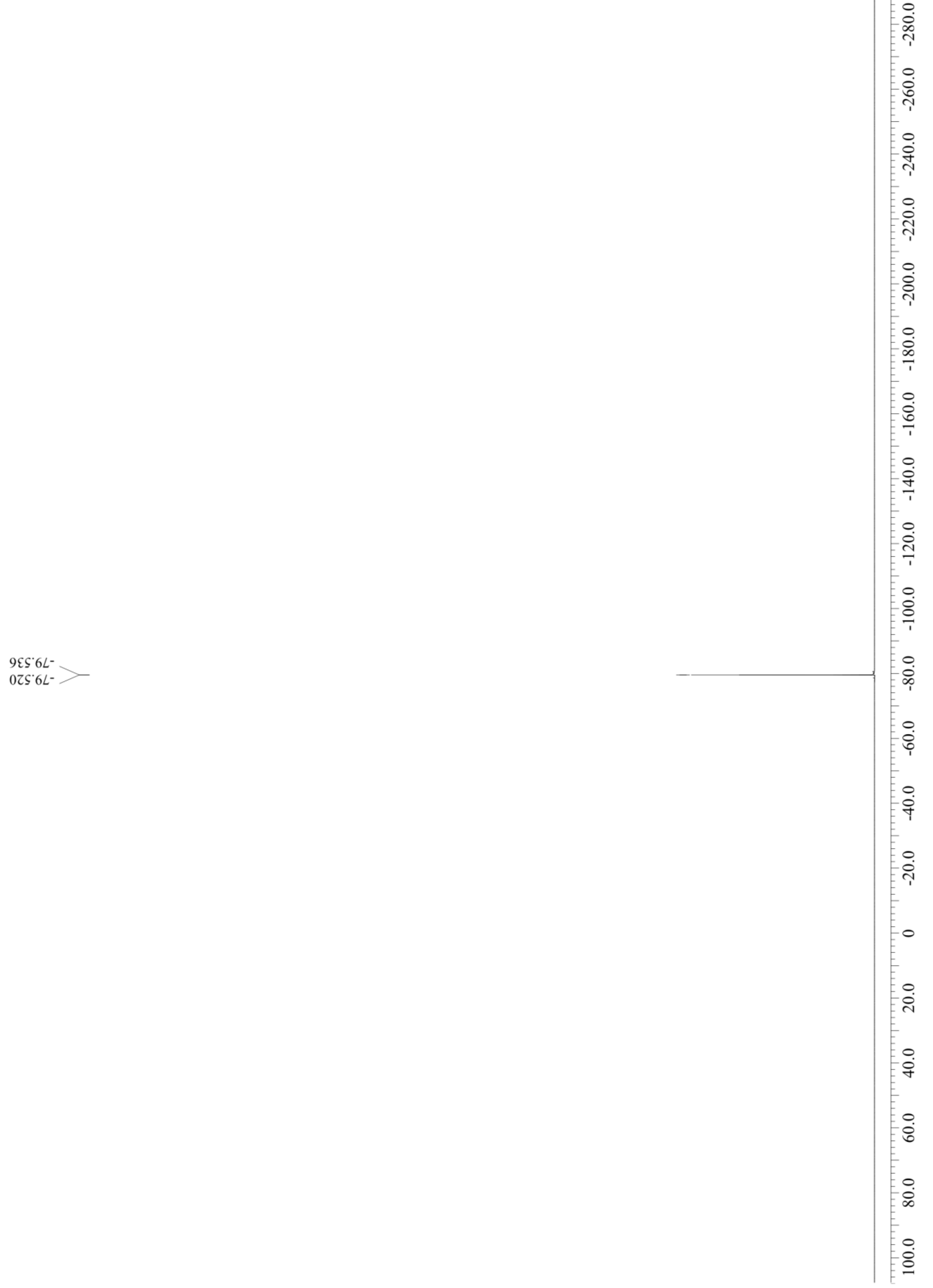

^{1}H NMR of **9t** ($CDCl_3$, 400 MHz, 25 ºC)

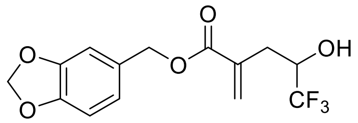

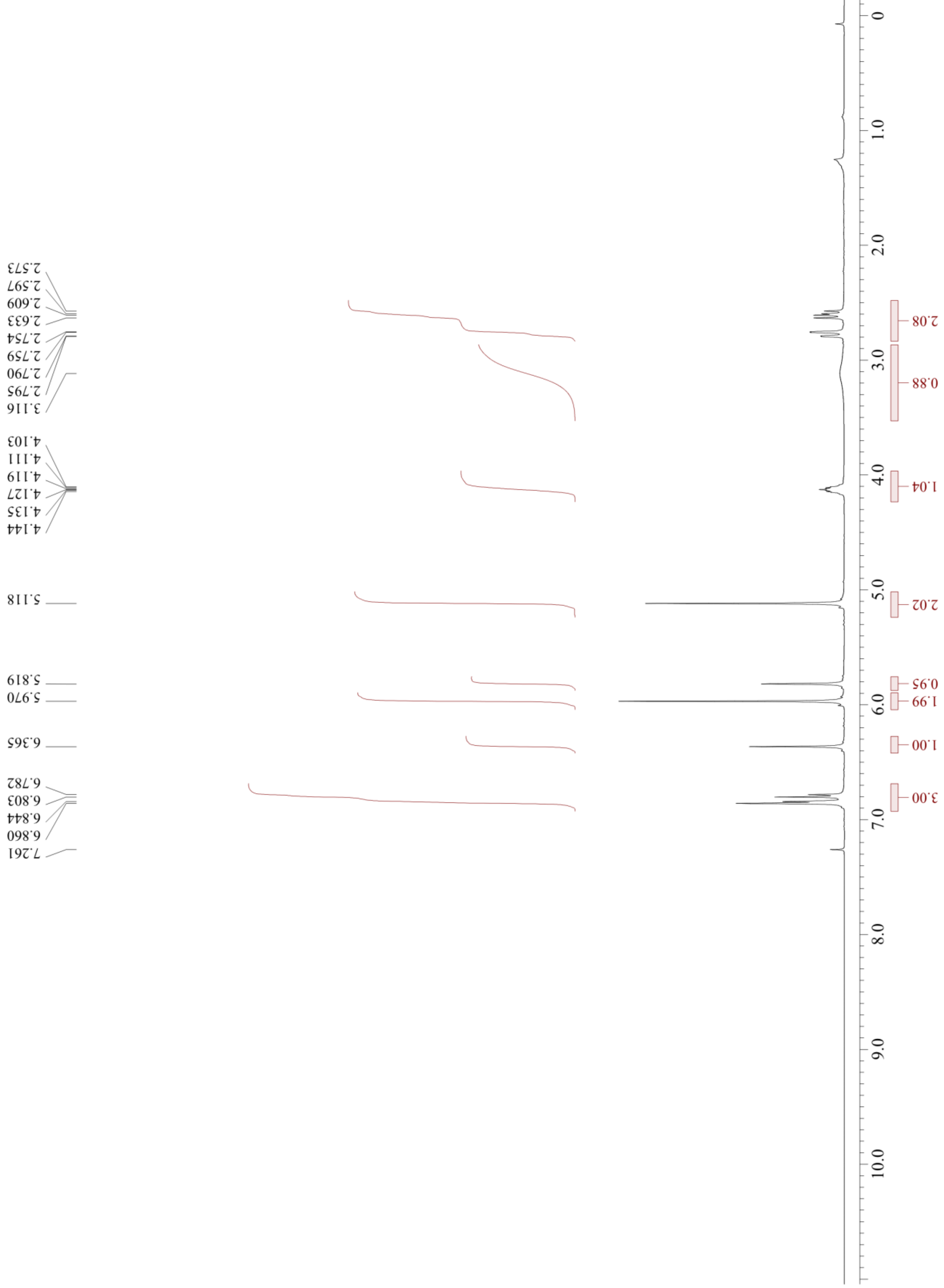

^{13}C NMR of **9t** ($CDCl_3$, 100 MHz, 25 ºC)

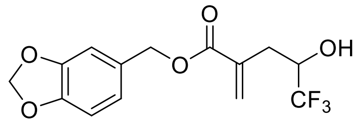

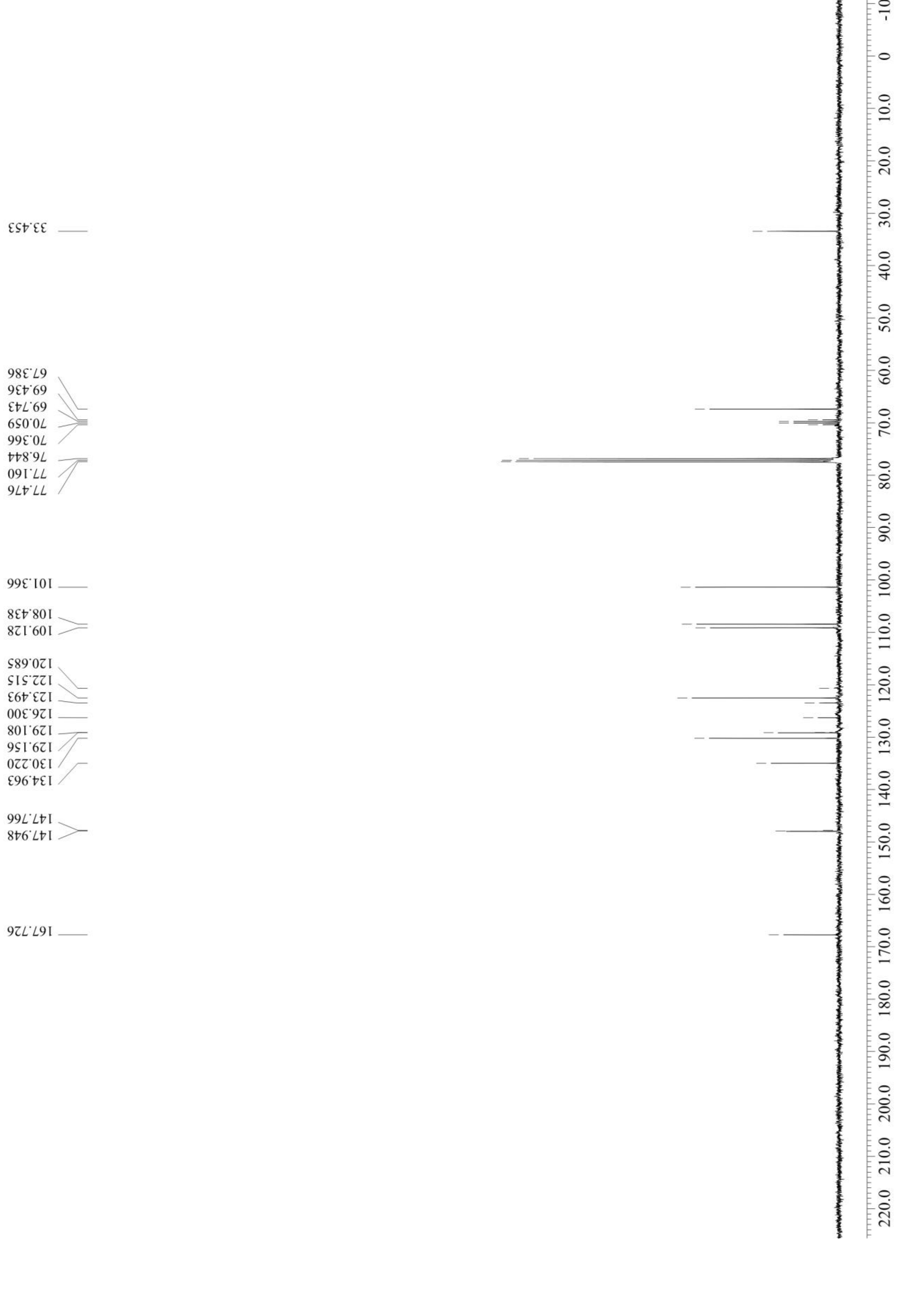

^{19}F NMR of **9t** ($CDCl_3$, 375 MHz, 25 ºC)

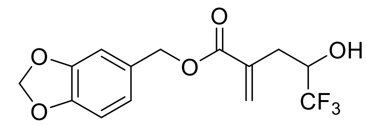

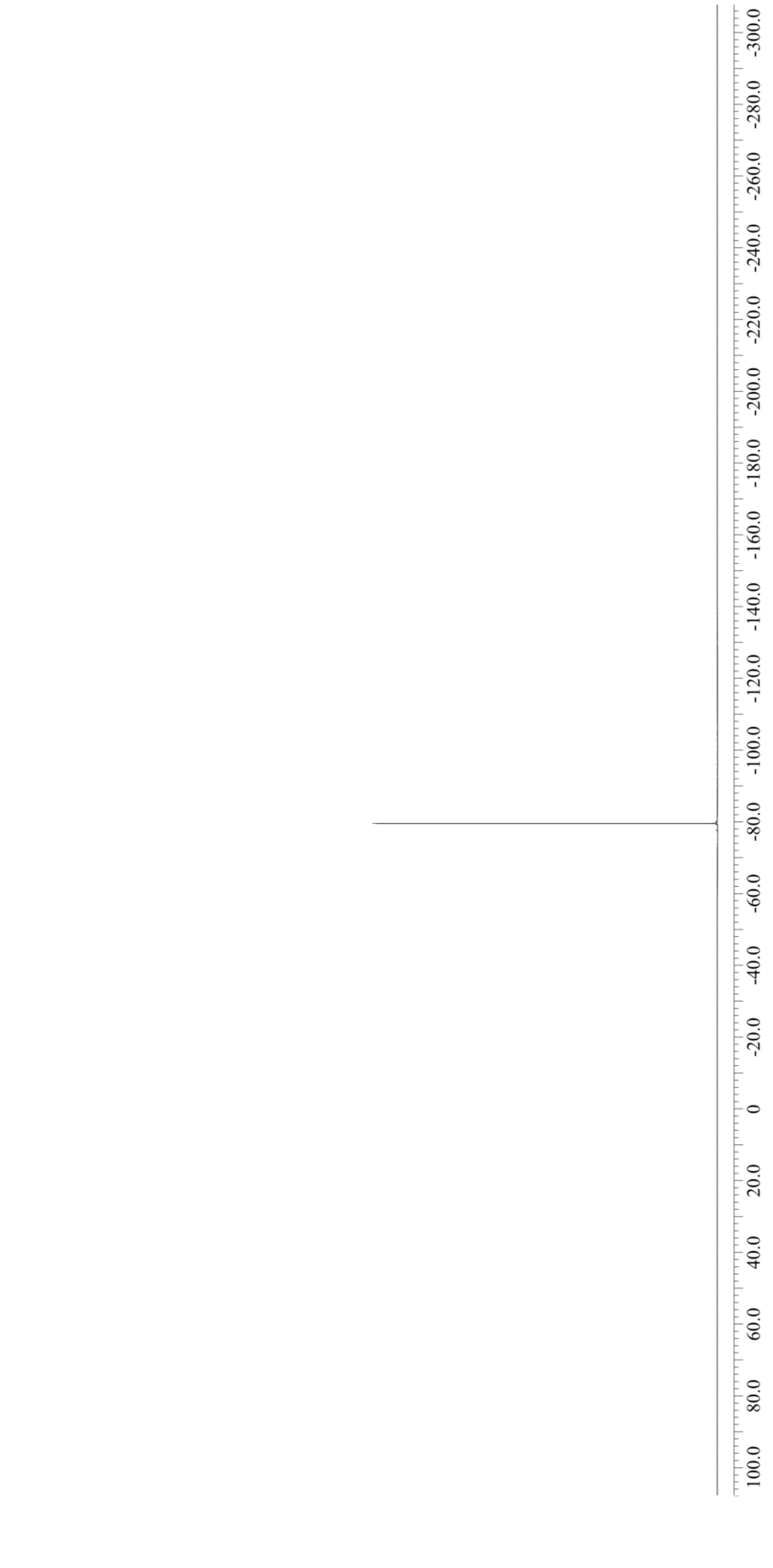

^{1}H NMR of **9u** ($CDCl_3$, 400 MHz, 25 ºC)

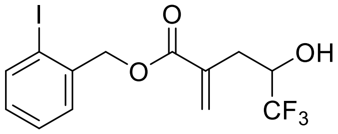

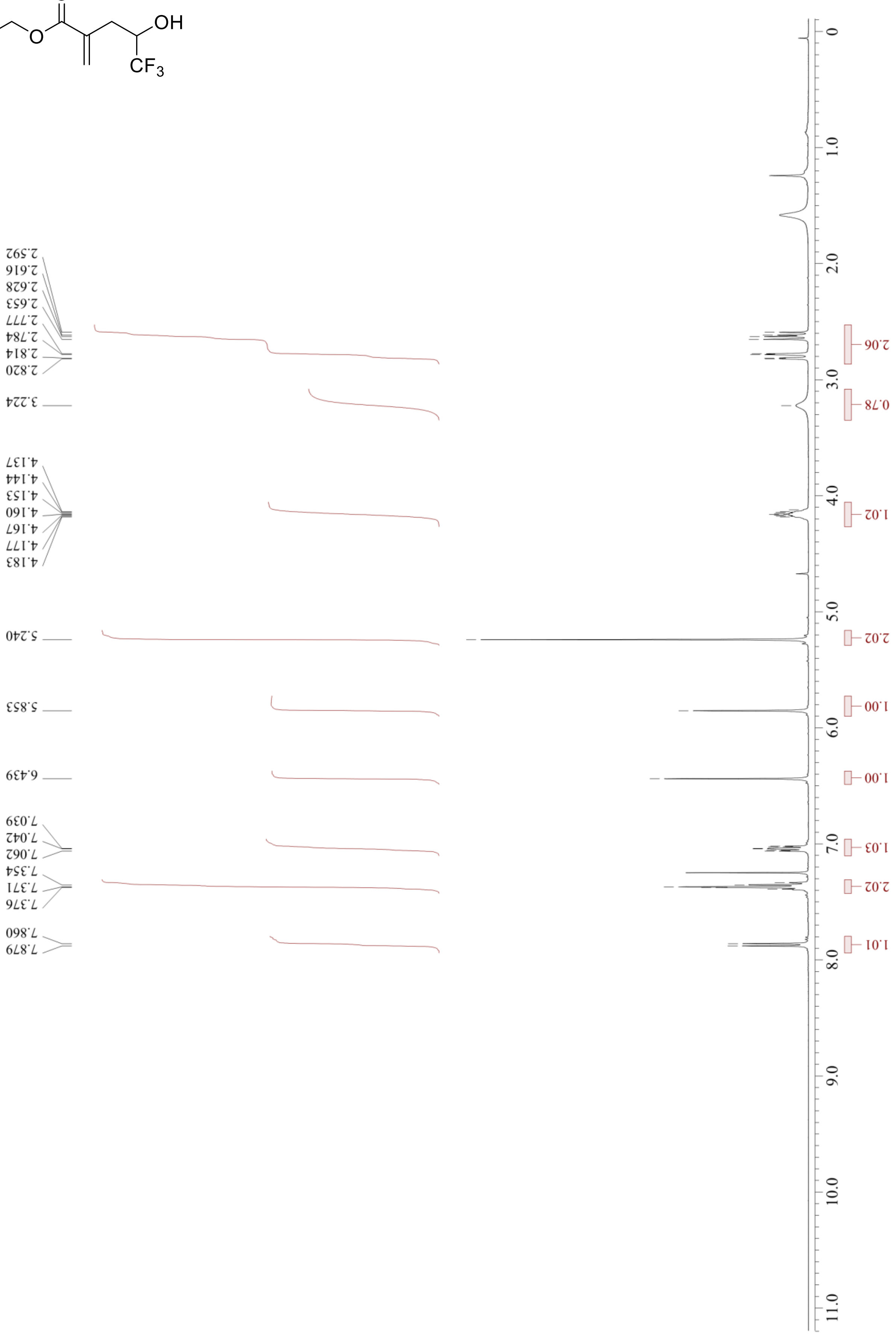

^{13}C NMR of **9u** ($CDCl_3$, 100 MHz, 25 ºC)

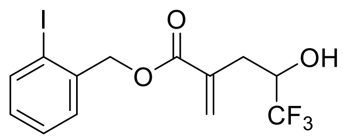

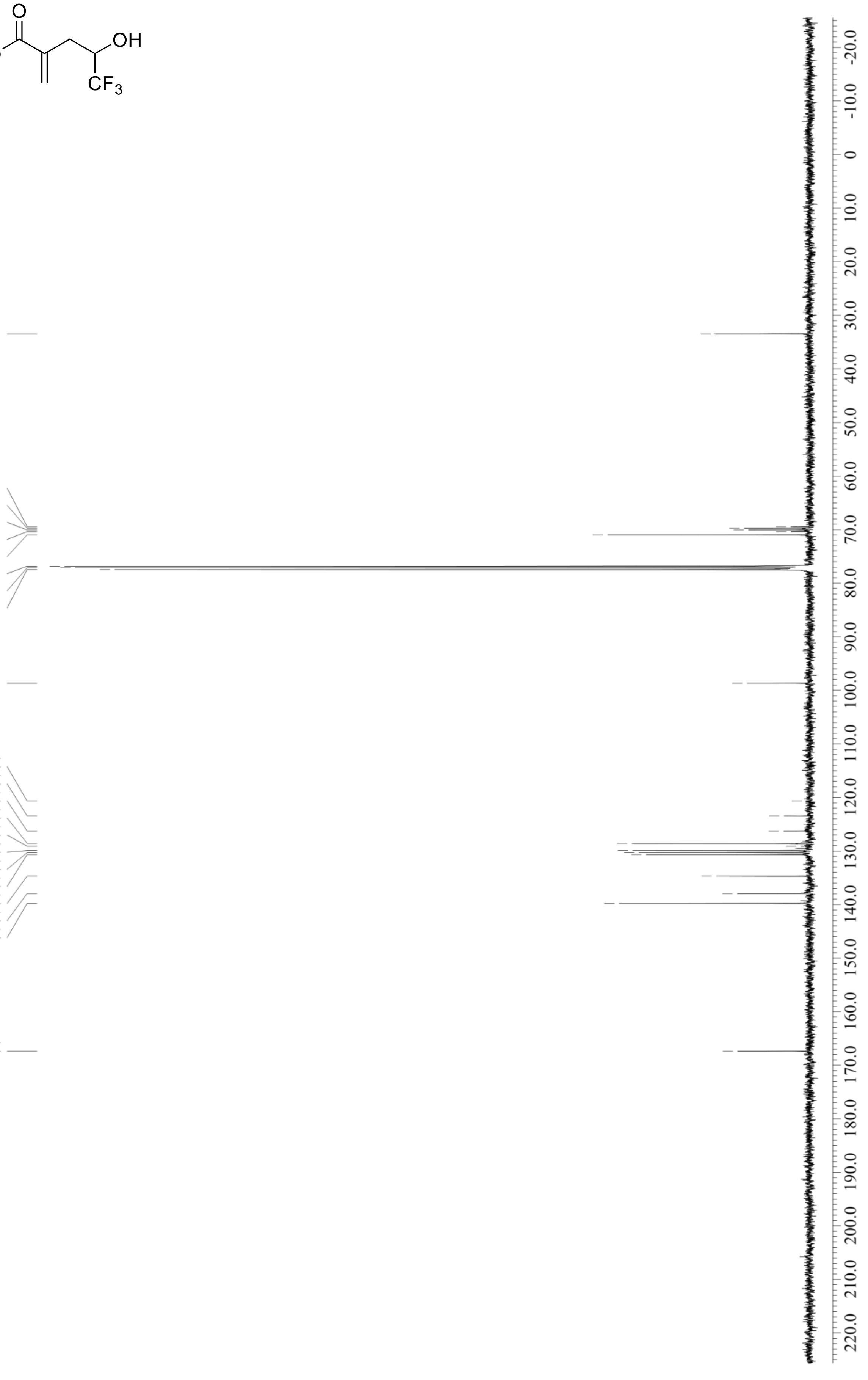

^{19}F NMR of **9u** ($CDCl_3$, 375 MHz, 25 ºC)

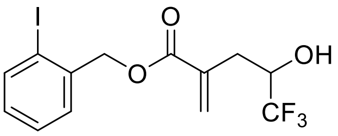

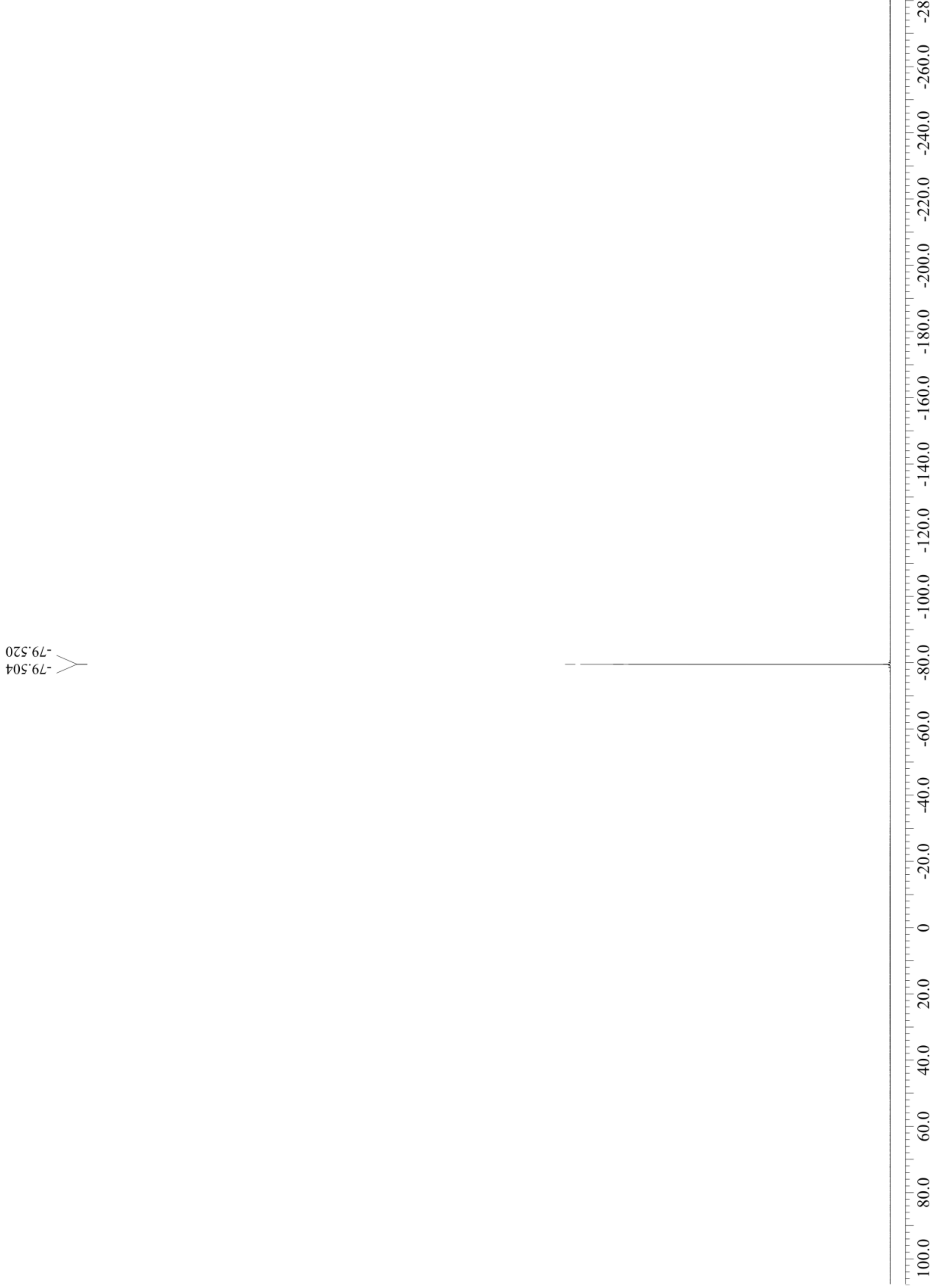

^{1}H NMR of **9v** ($CDCl_3$, 400 MHz, 25 °C)

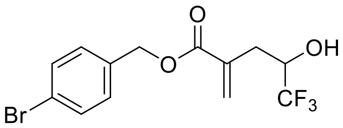

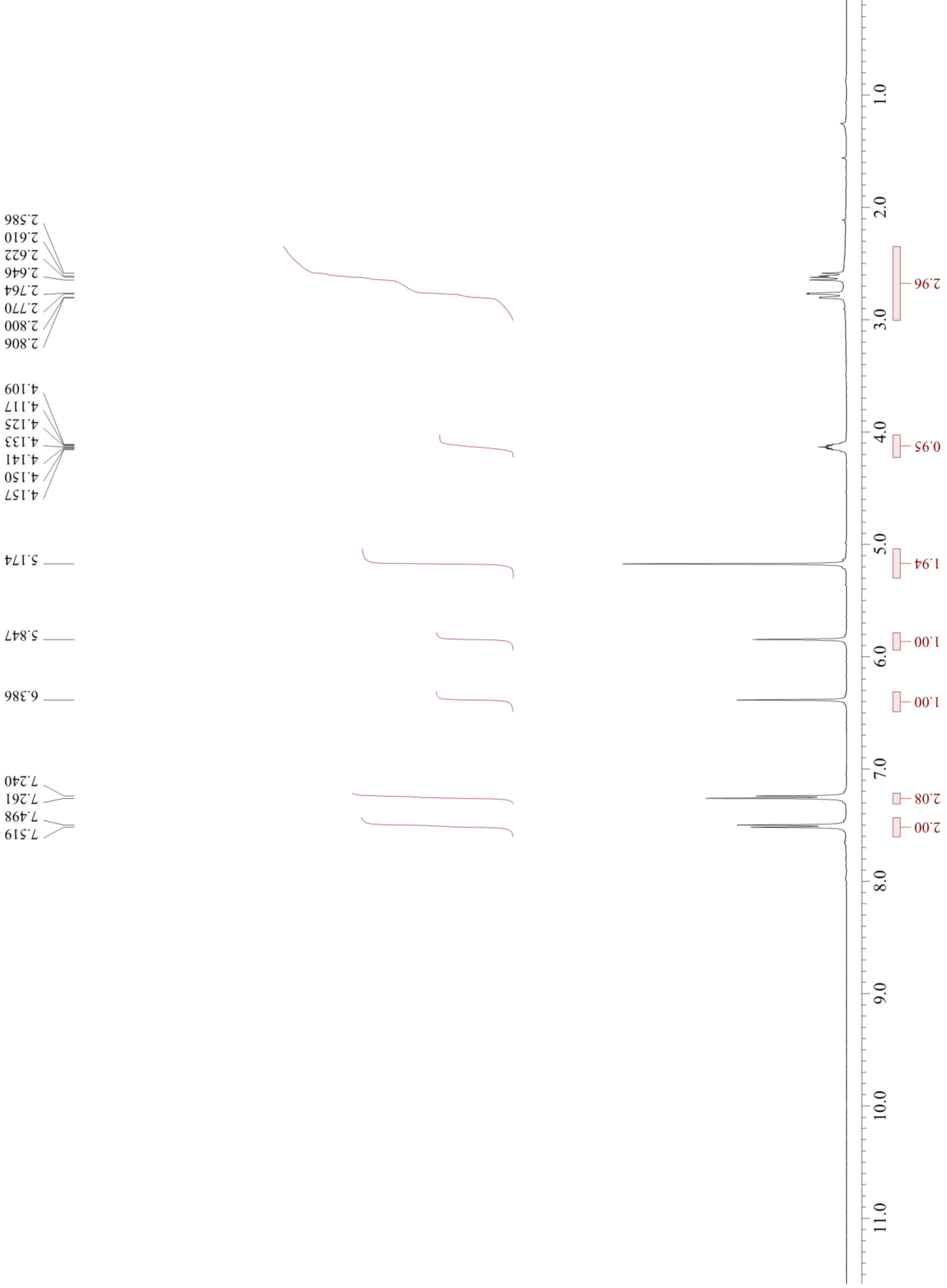

^{13}C NMR of **9v** ($CDCl_3$, 100 MHz, 25 ºC)

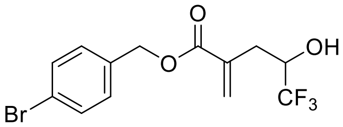

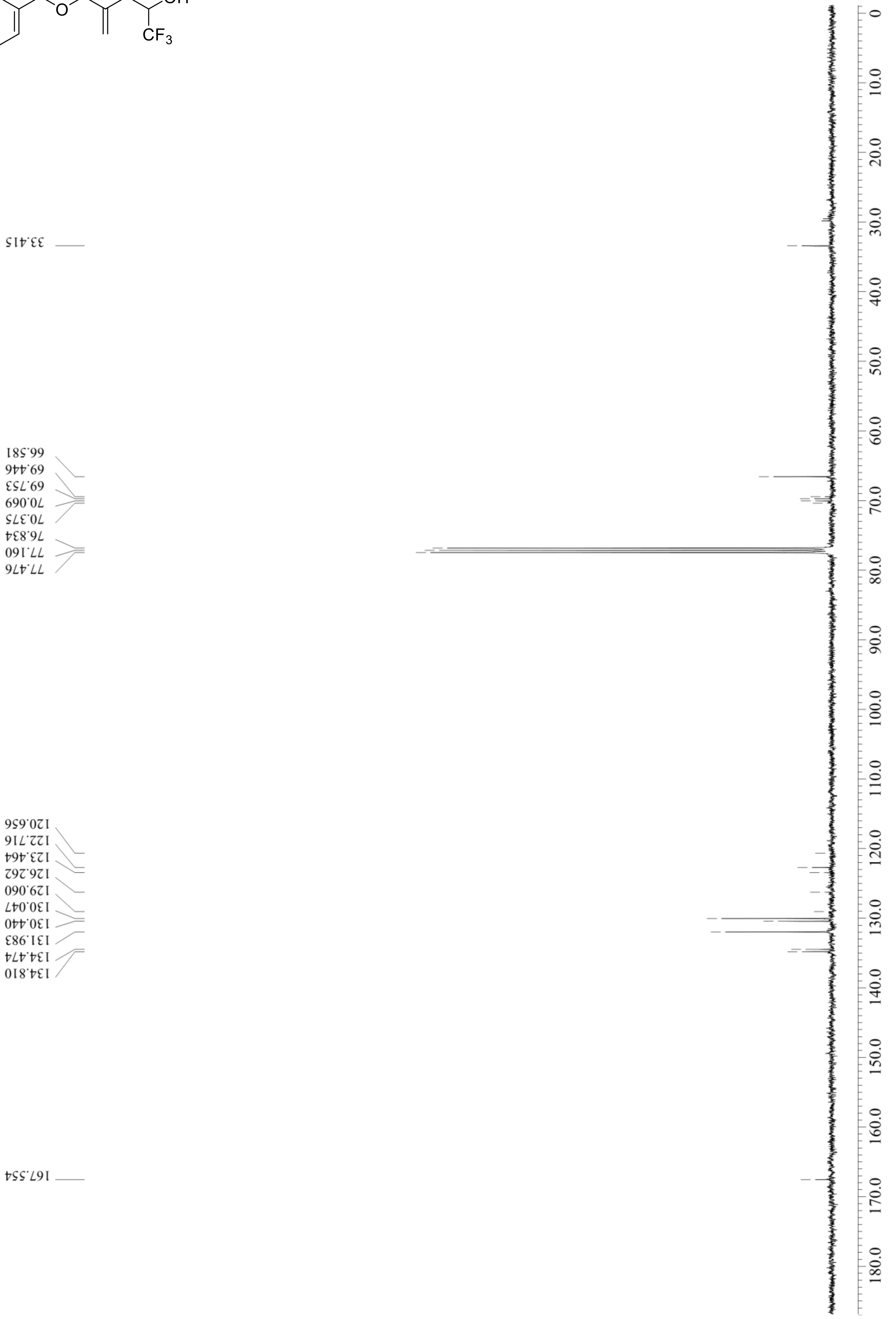

^{19}F NMR of **9v** ($CDCl_3$, 375 MHz, 25 ºC)

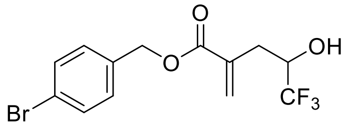

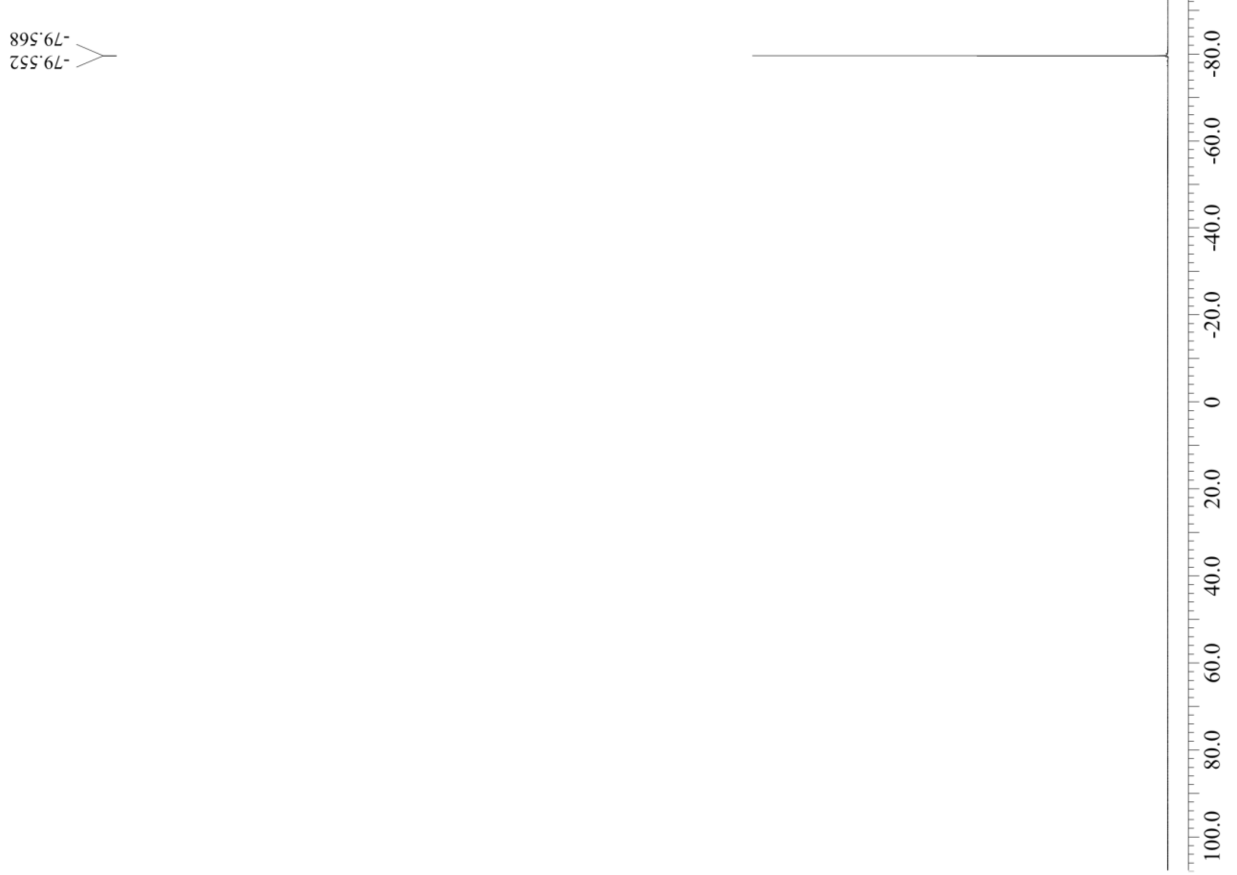

^{1}H NMR of **9w** ($CDCl_3$, 400 MHz, 25 ºC)

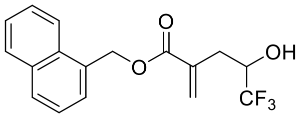

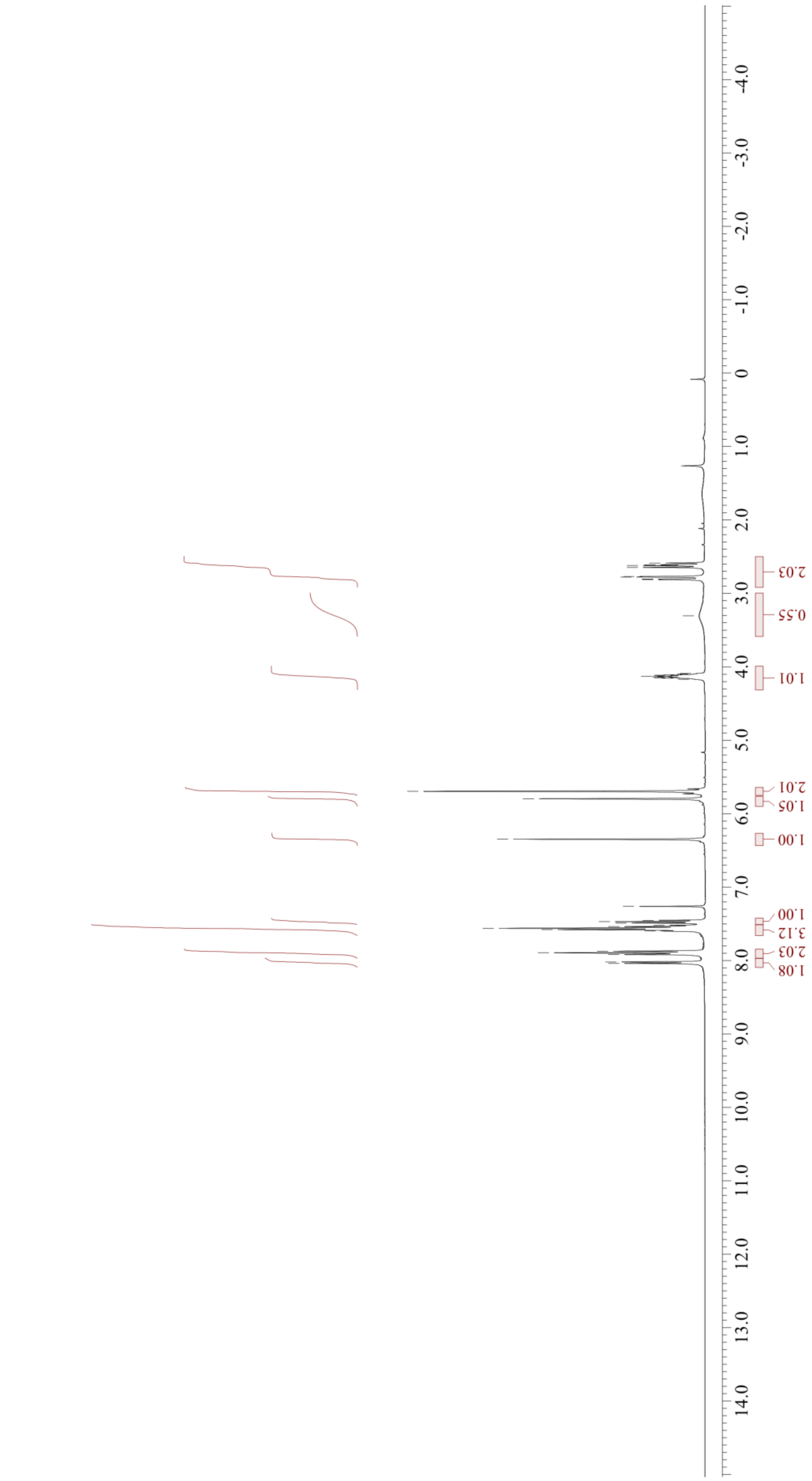

^{13}C NMR of **9w** ($CDCl_3$, 100 MHz, 25 ºC)

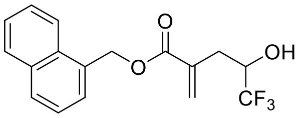

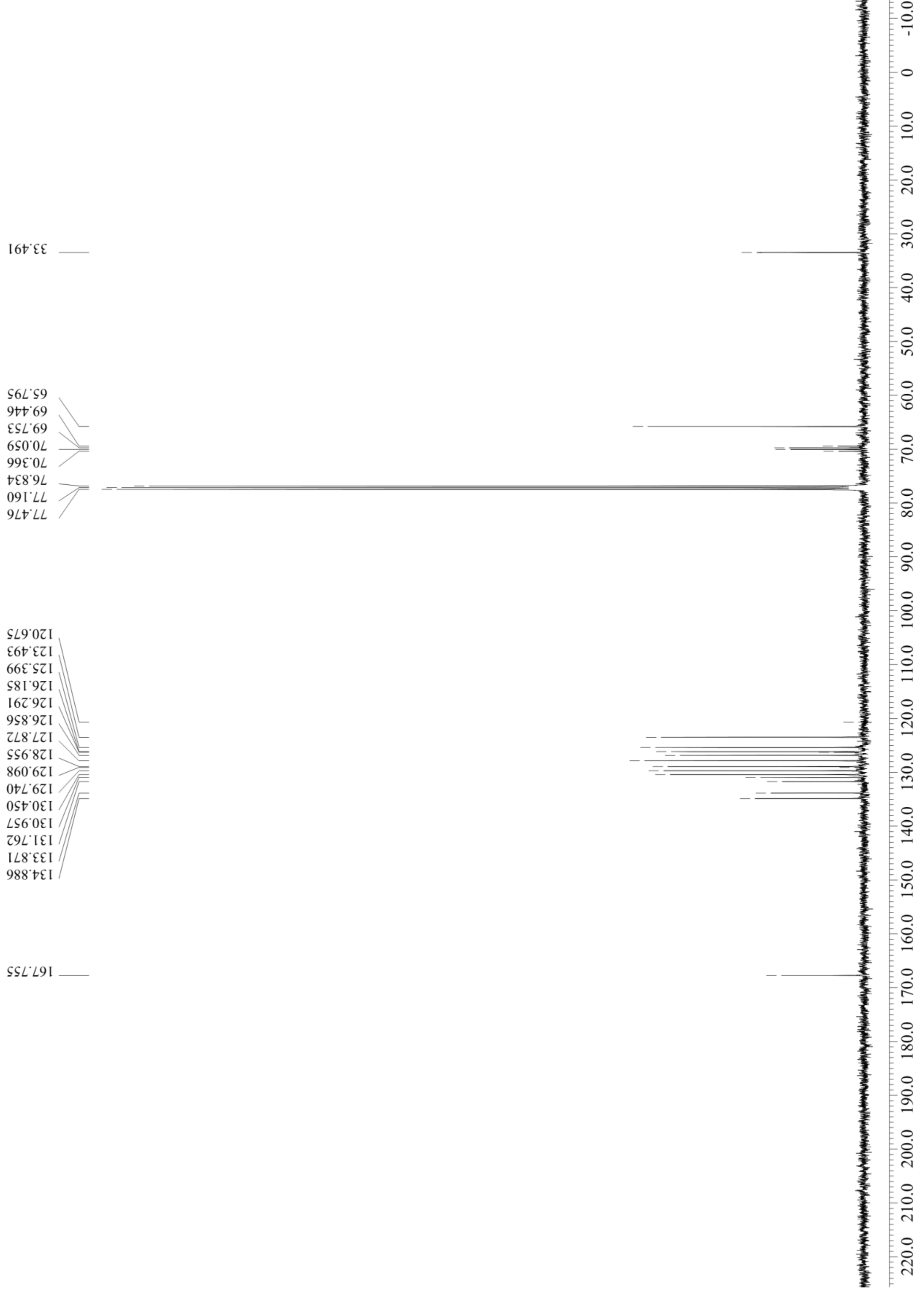

^{19}F NMR of **9w** ($CDCl_3$, 375 MHz, 25 ºC)

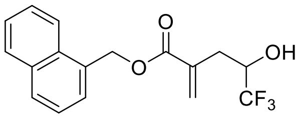

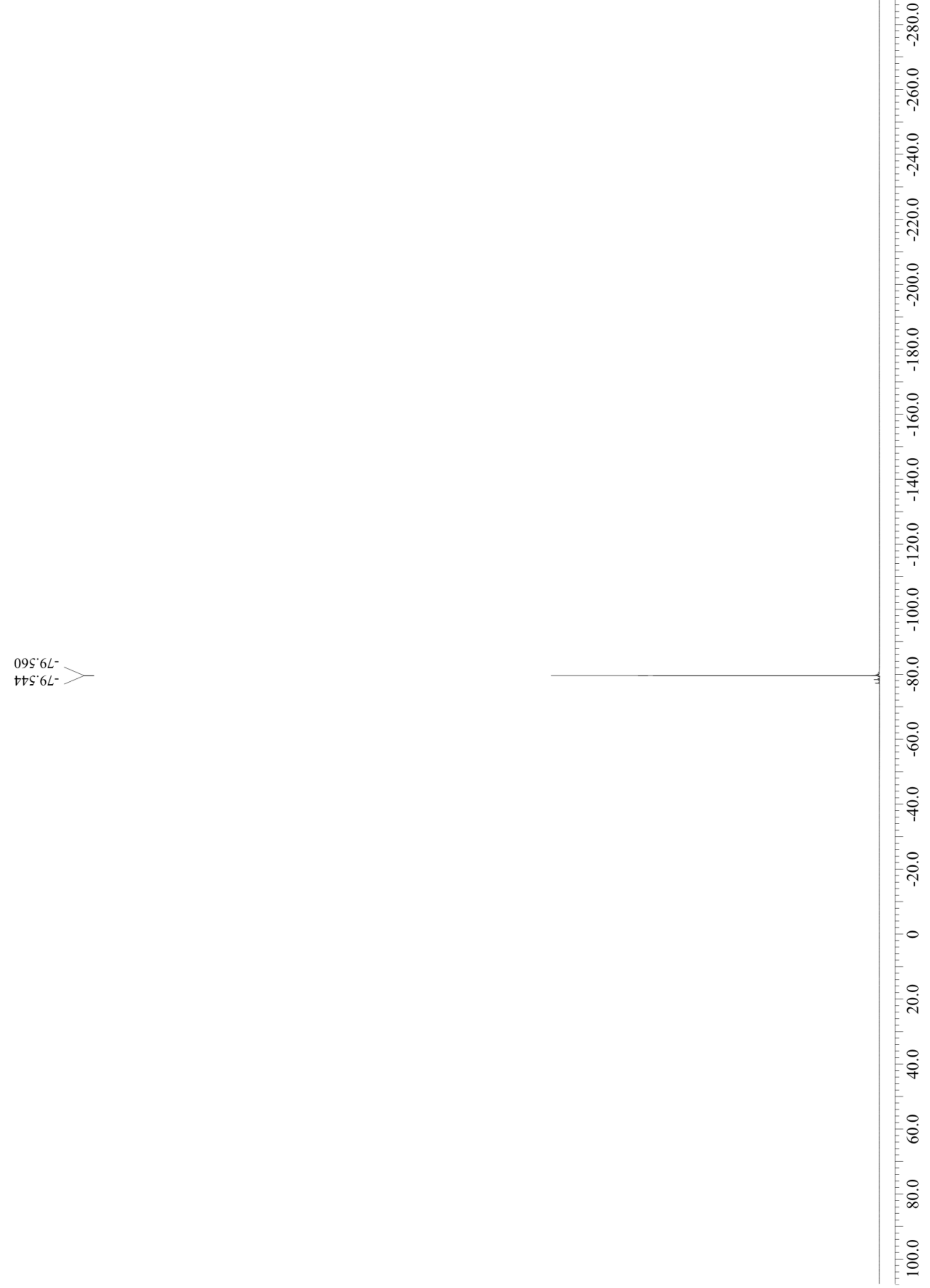

^{1}H NMR of **9x** ($CDCl_3$, 400 MHz, 25 ºC)

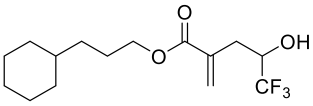

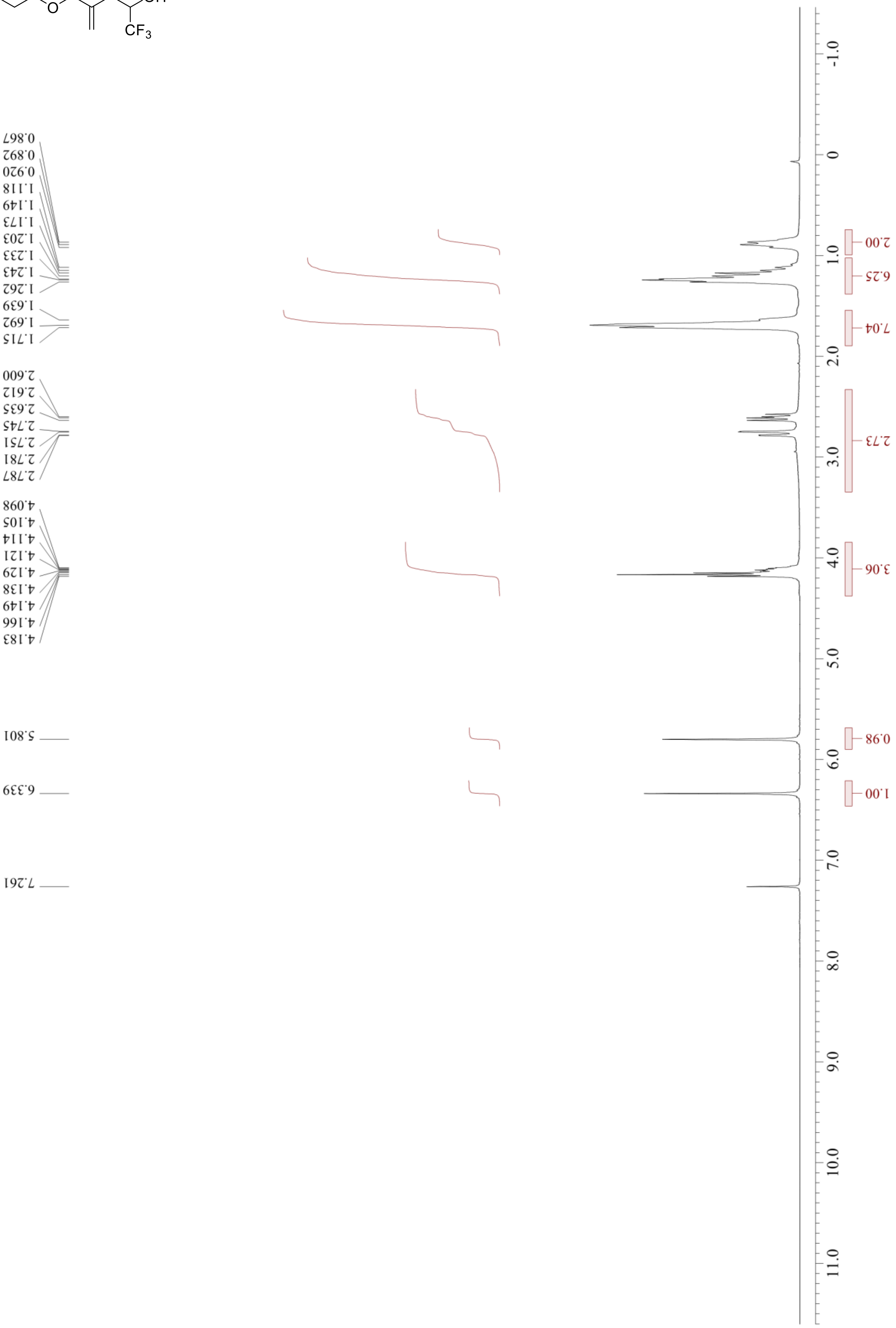

^{13}C NMR of **9x** ($CDCl_3$, 100 MHz, 25 ºC)

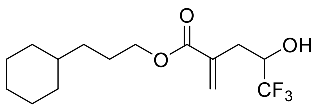

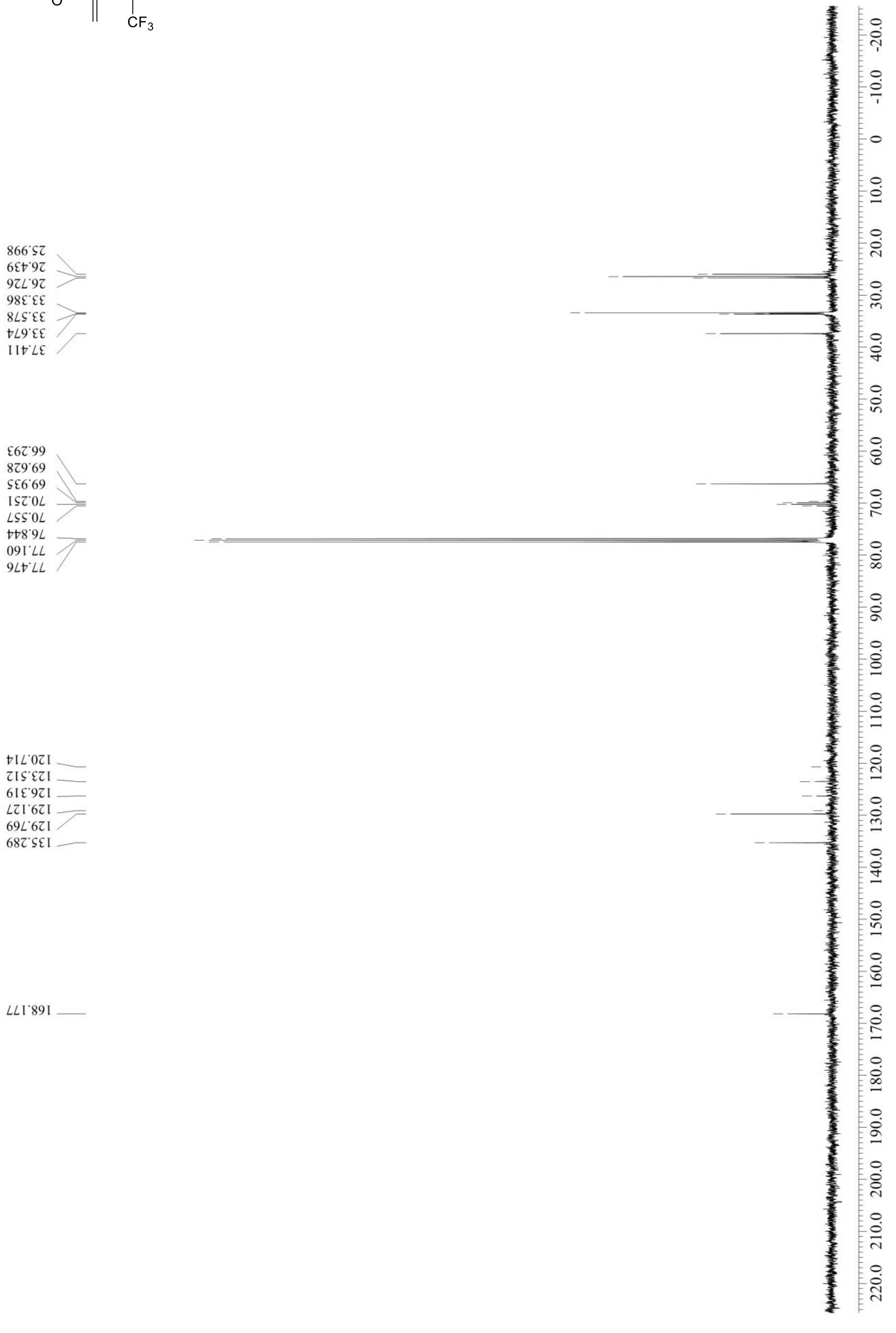

^{19}F NMR of **9x** ($CDCl_3$, 375 MHz, 25 ºC)

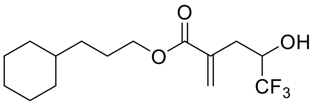

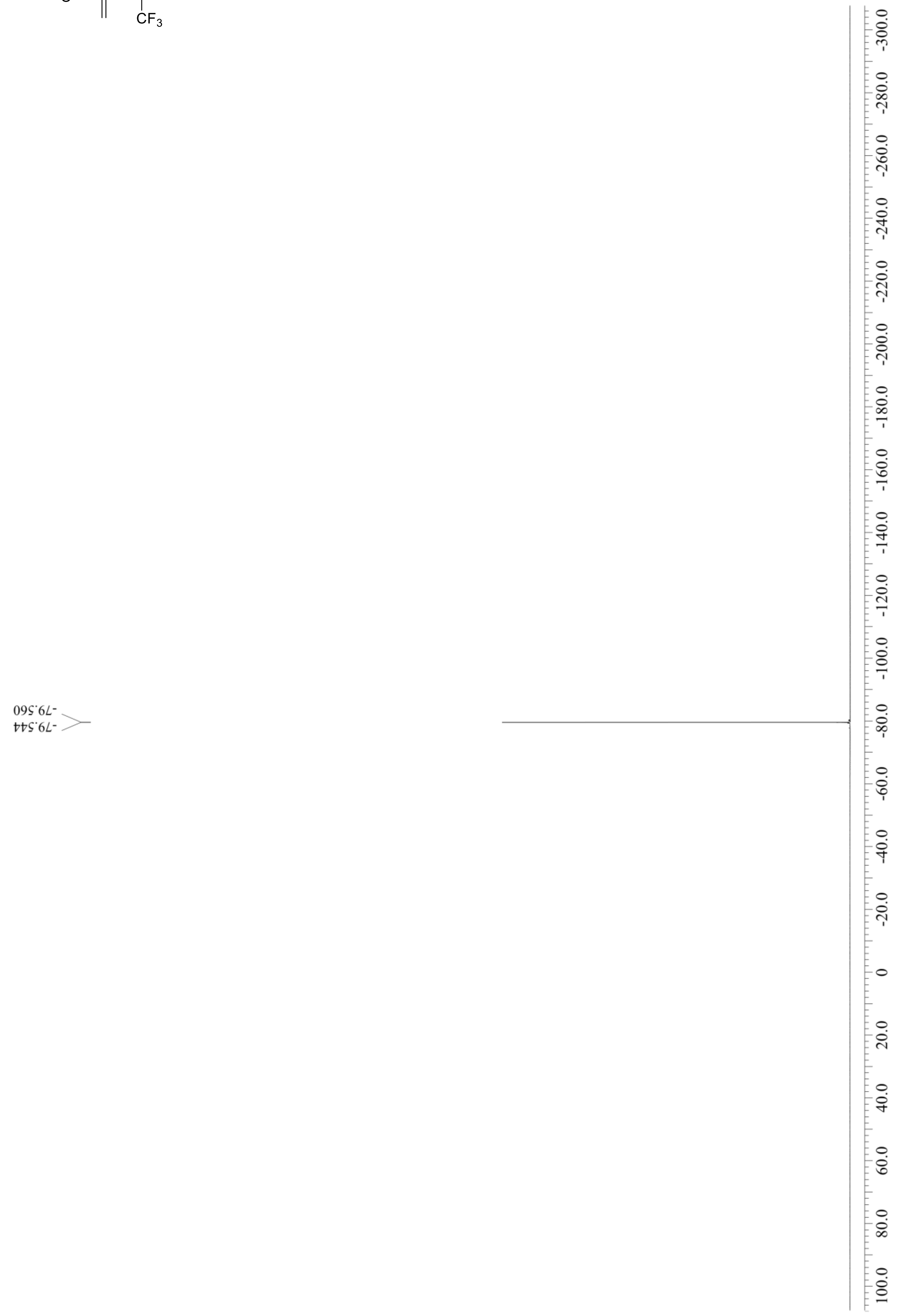

^{1}H NMR of **9y** ($CDCl_3$, 400 MHz, 25 ºC)

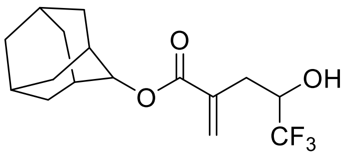

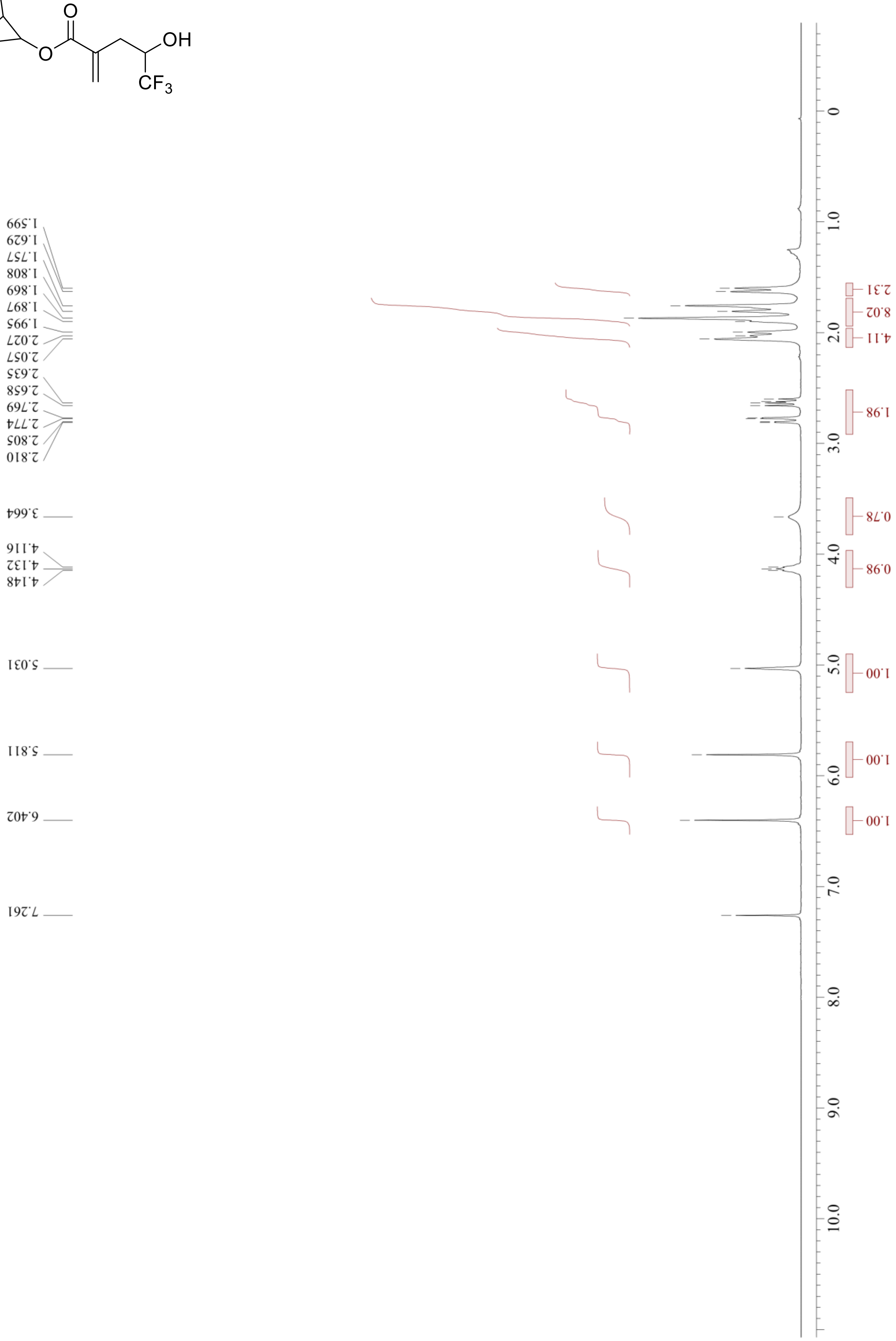

^{13}C NMR of **9y** ($CDCl_3$, 100 MHz, 25 ºC)

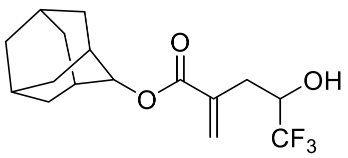

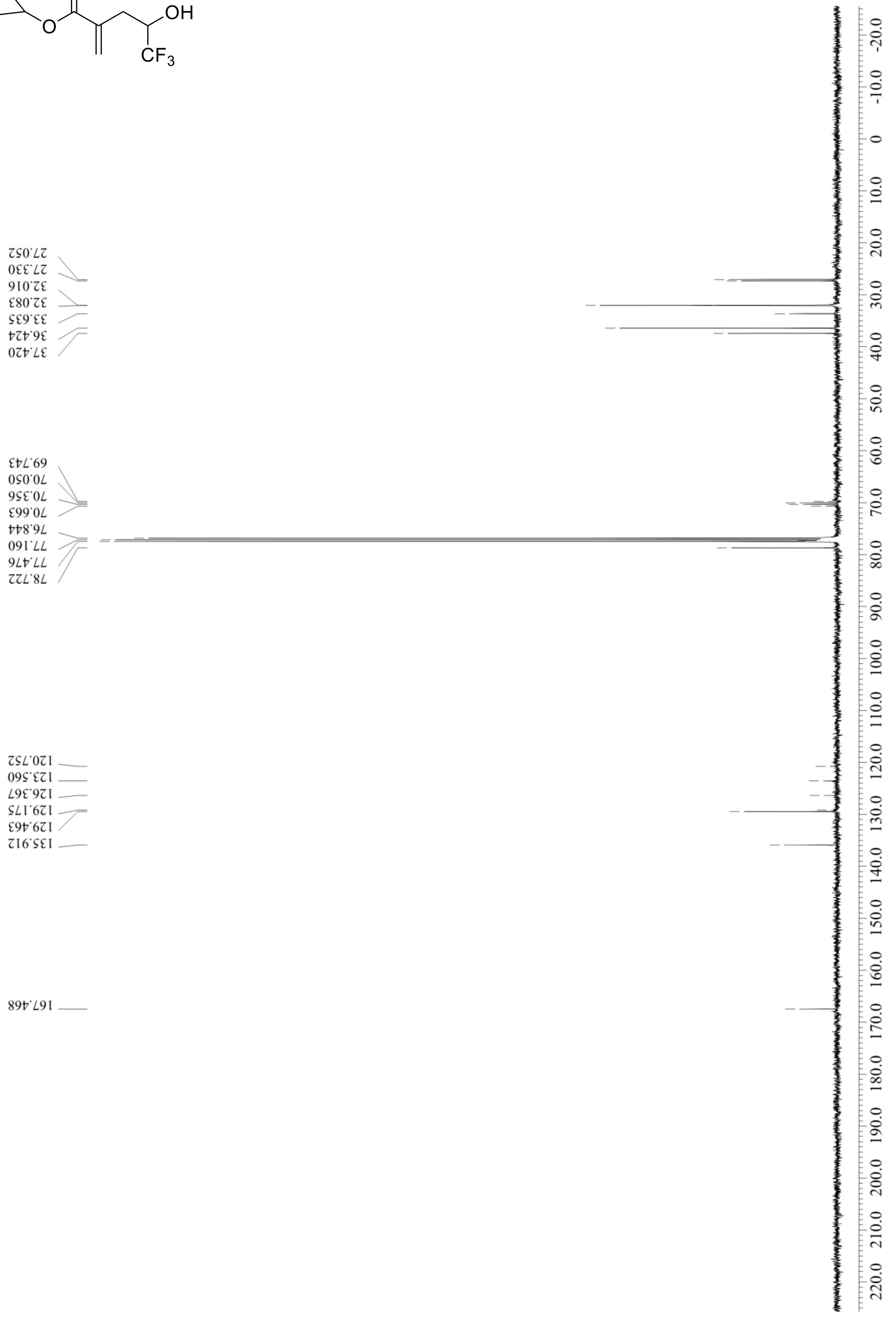

^{19}F NMR of **9y** ($CDCl_3$, 375 MHz, 25 ºC)

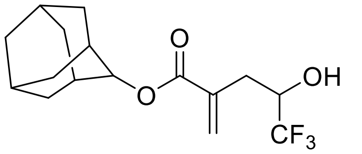

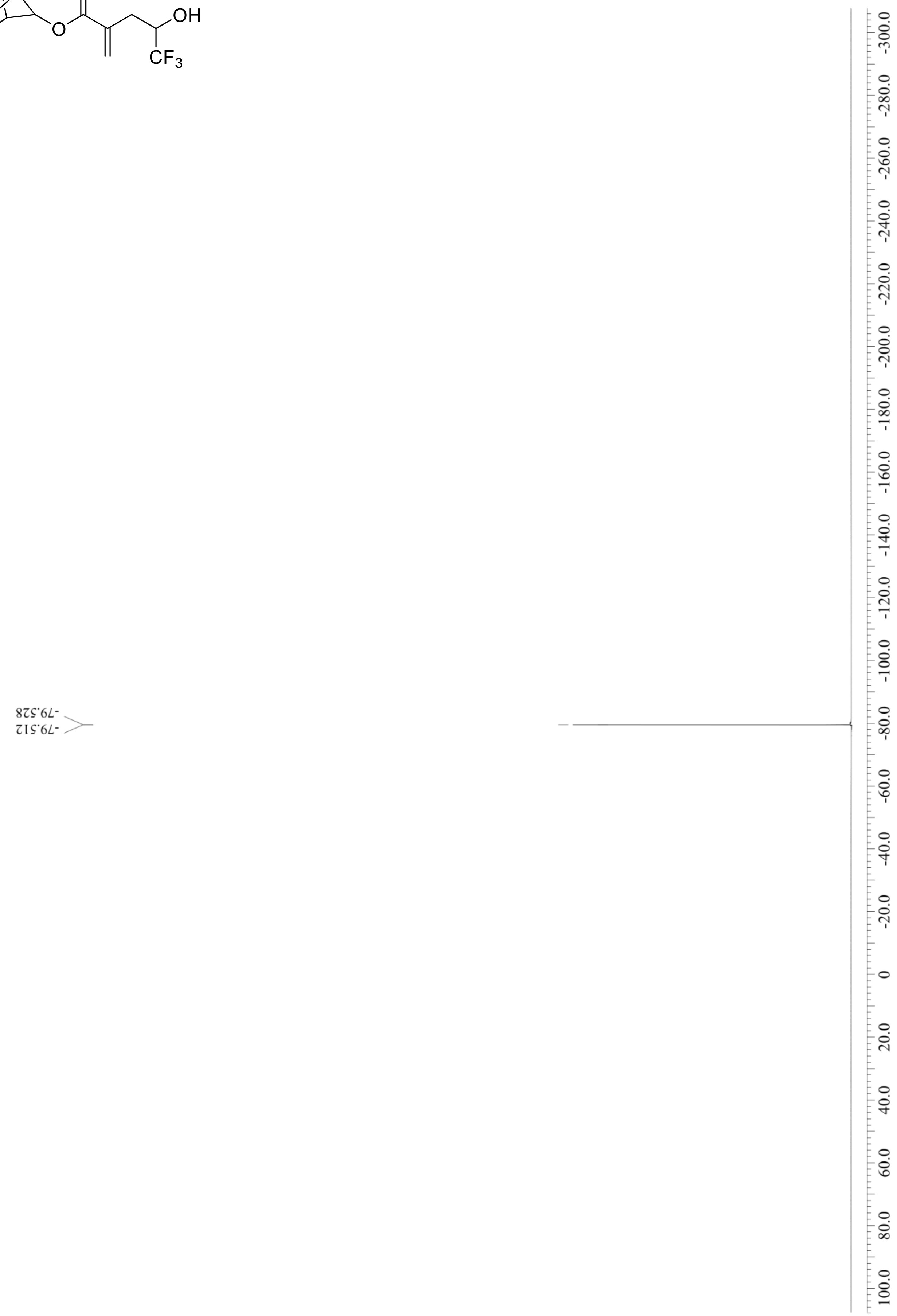

^{1}H NMR of **9z** ($CDCl_3$, 400 MHz, 25 ºC)

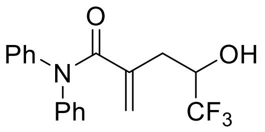

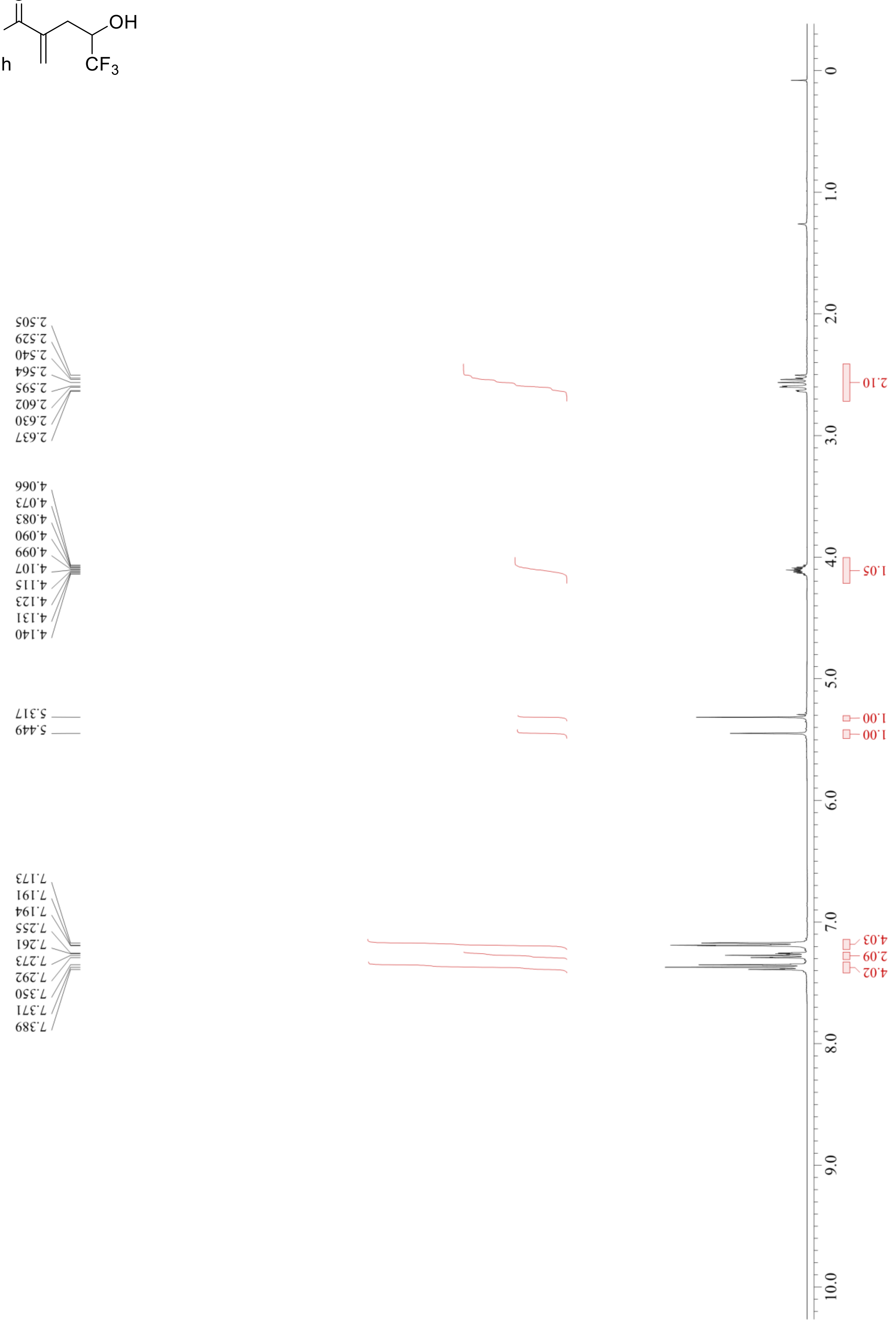

^{13}C NMR of **9z** ($CDCl_3$, 100 MHz, 25 ºC)

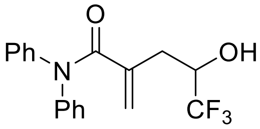

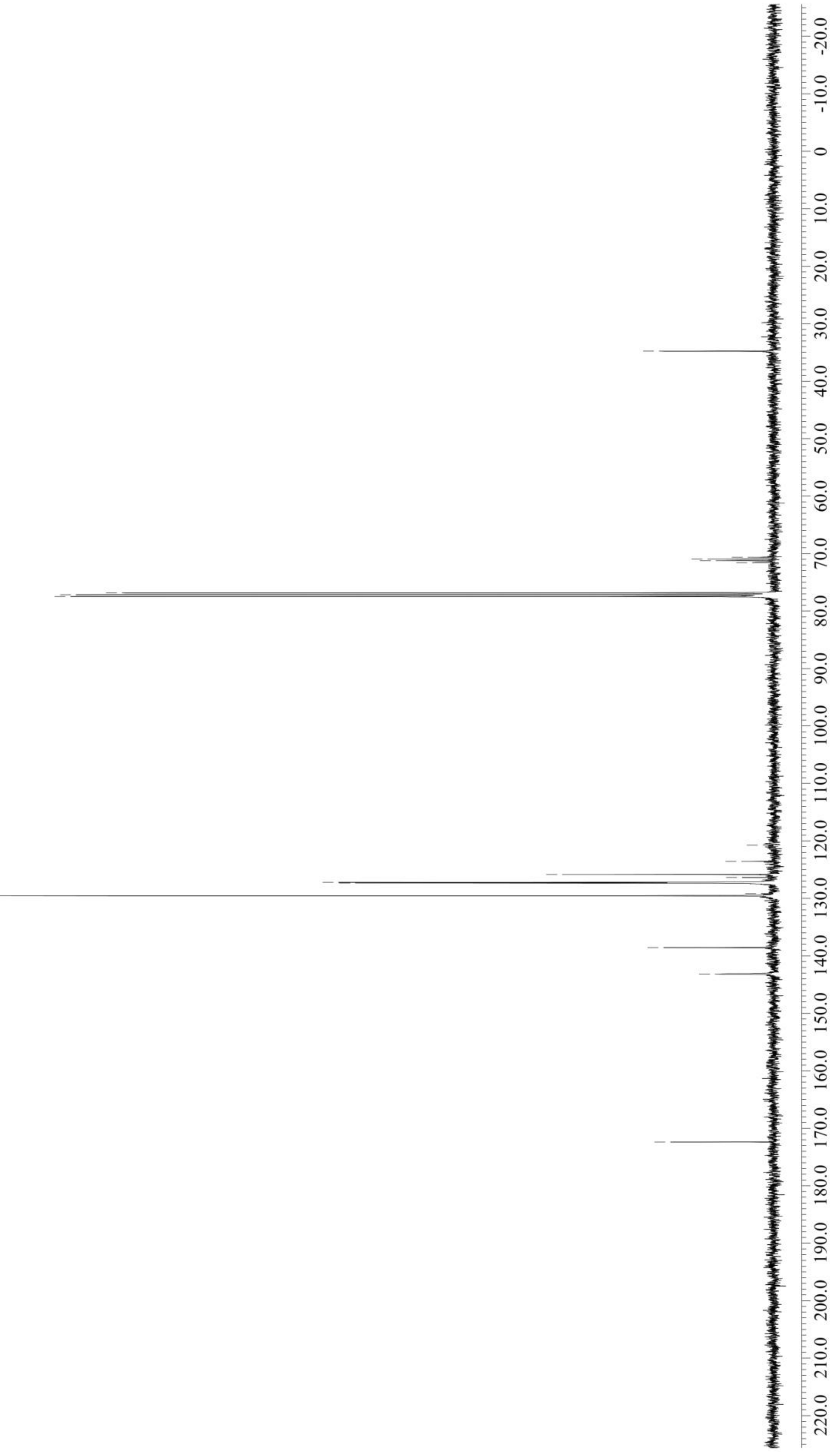

^{19}F NMR of **9z** ($CDCl_3$, 375 MHz, 25 ºC)

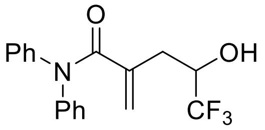

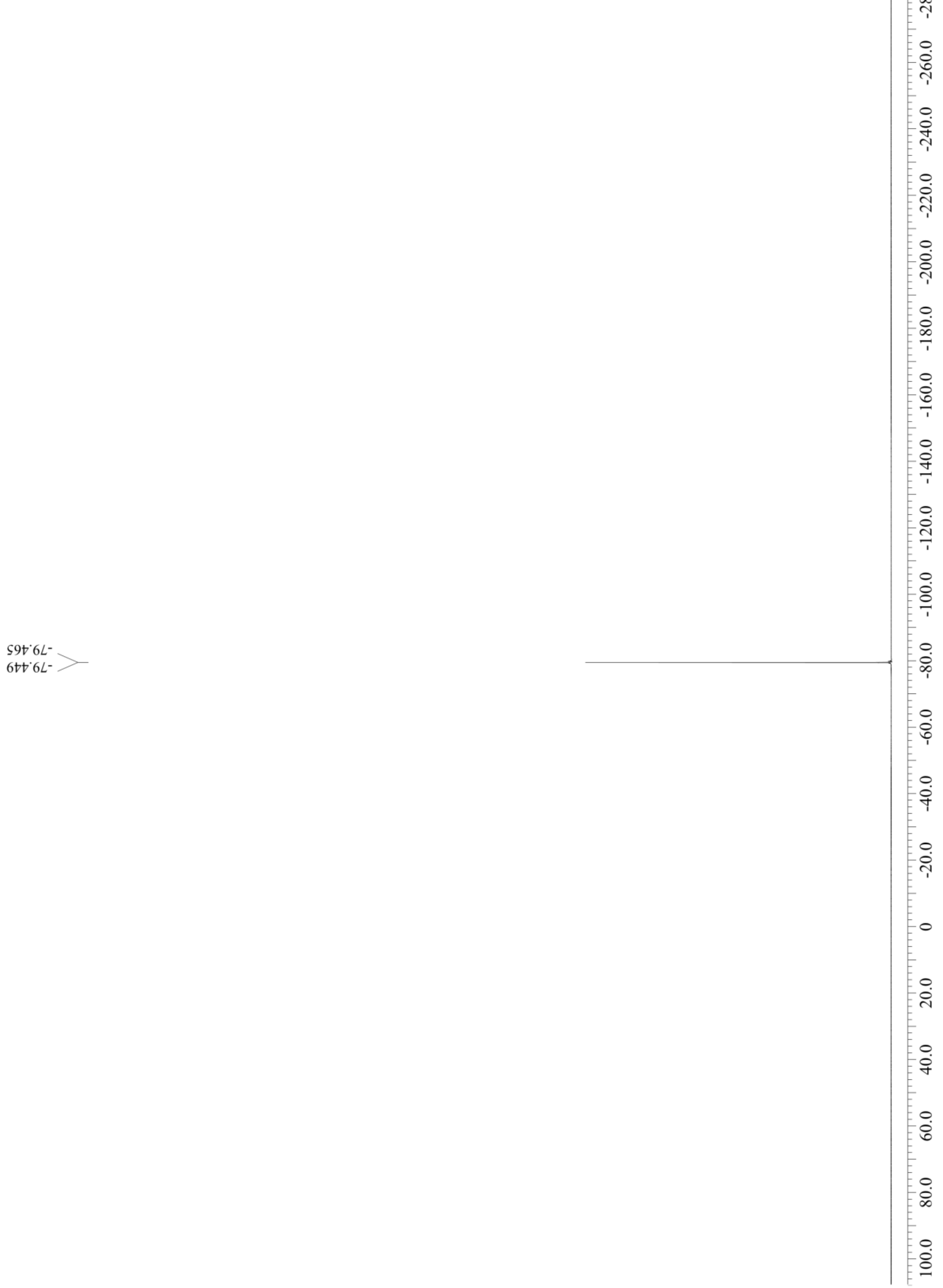

^{1}H NMR of **9aa** ($CDCl_3$, 400 MHz, 25 ºC)

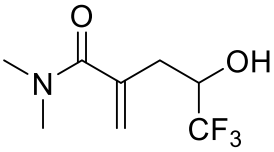

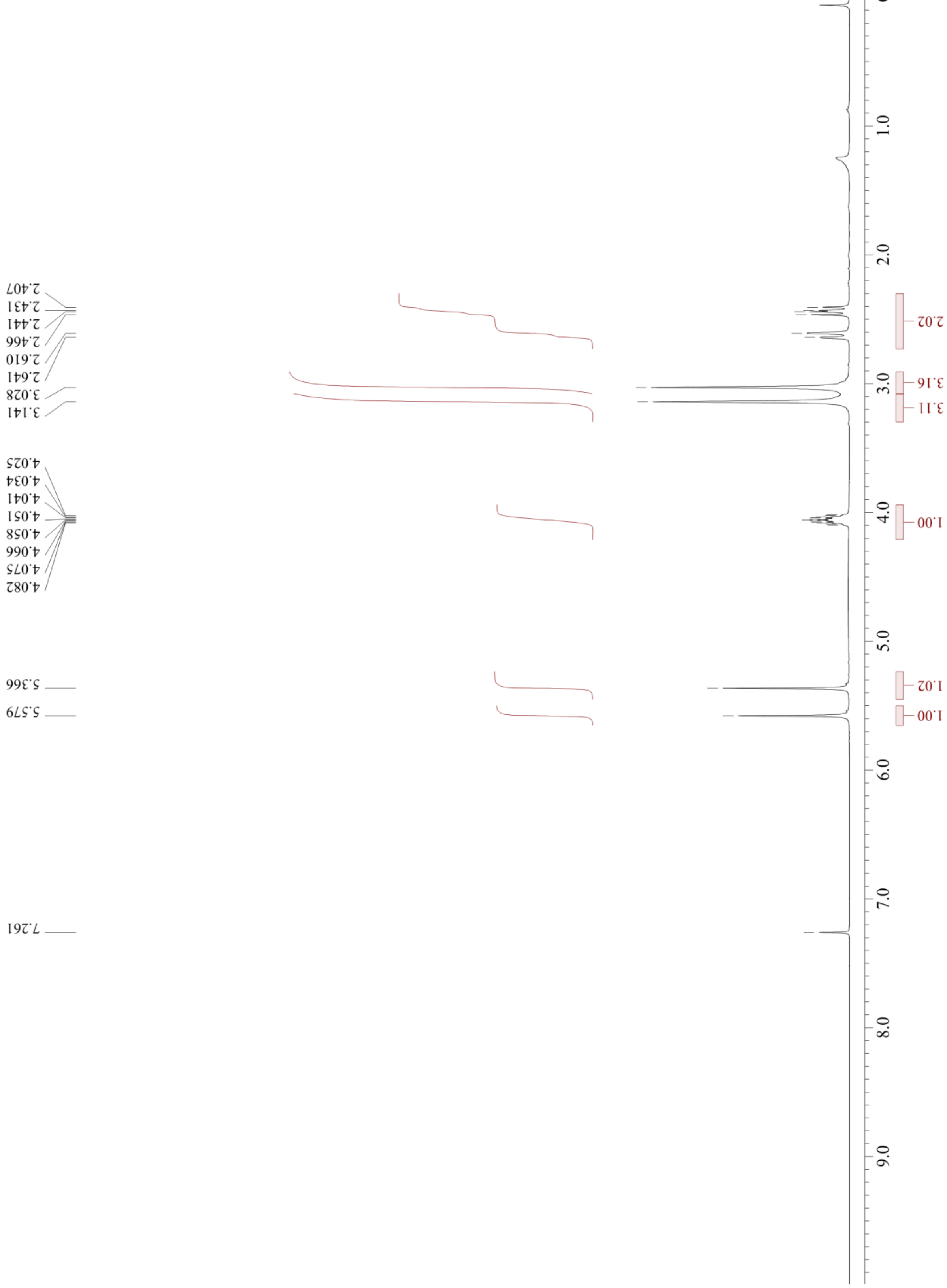

^{13}C NMR of **9aa** ($CDCl_3$, 100 MHz, 25 ºC)

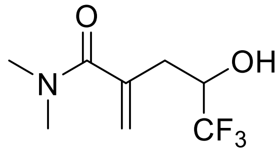

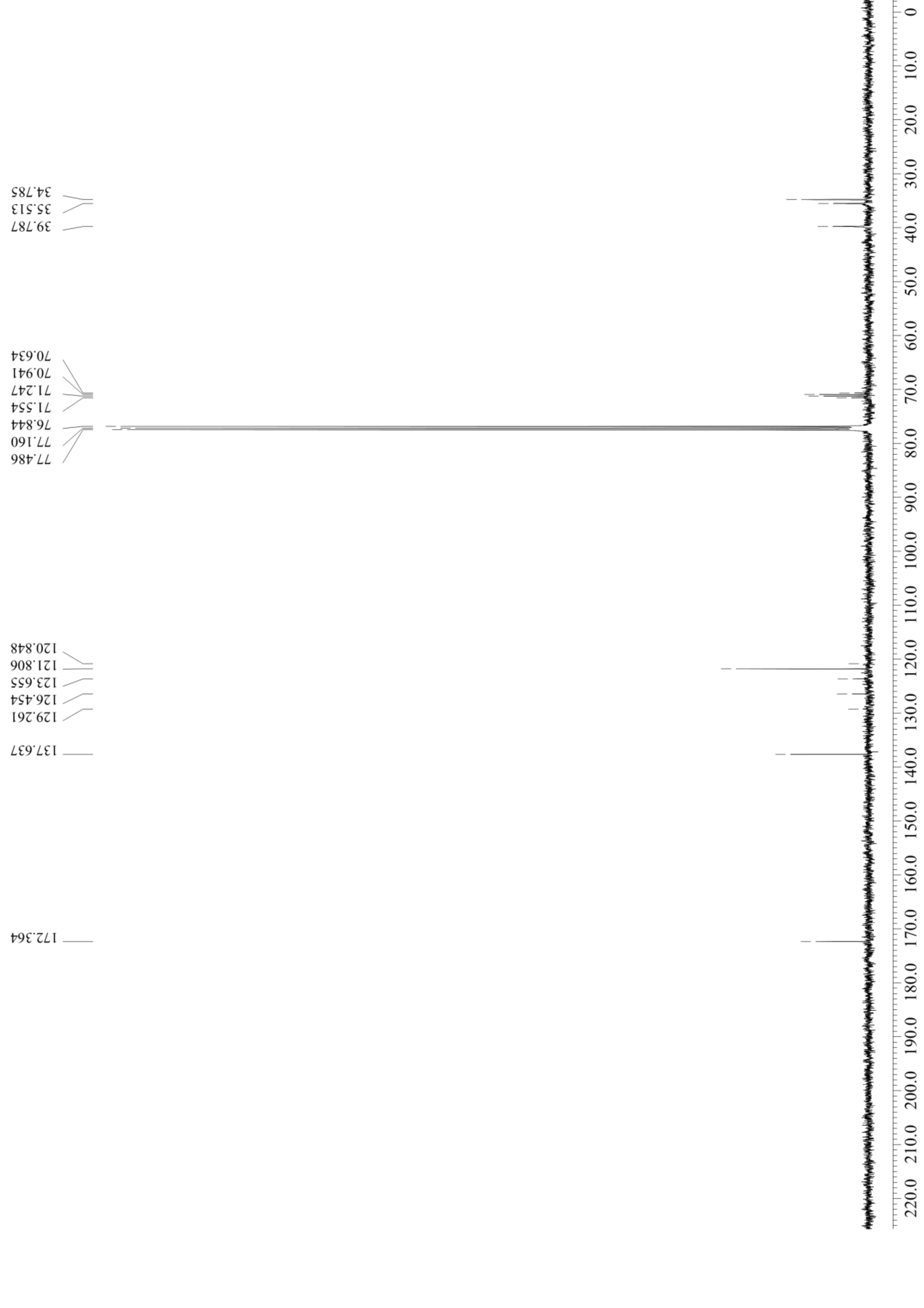

^{19}F NMR of **9aa** ($CDCl_3$, 375 MHz, 25 ºC)

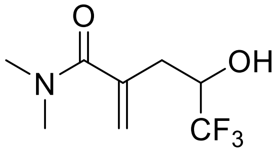

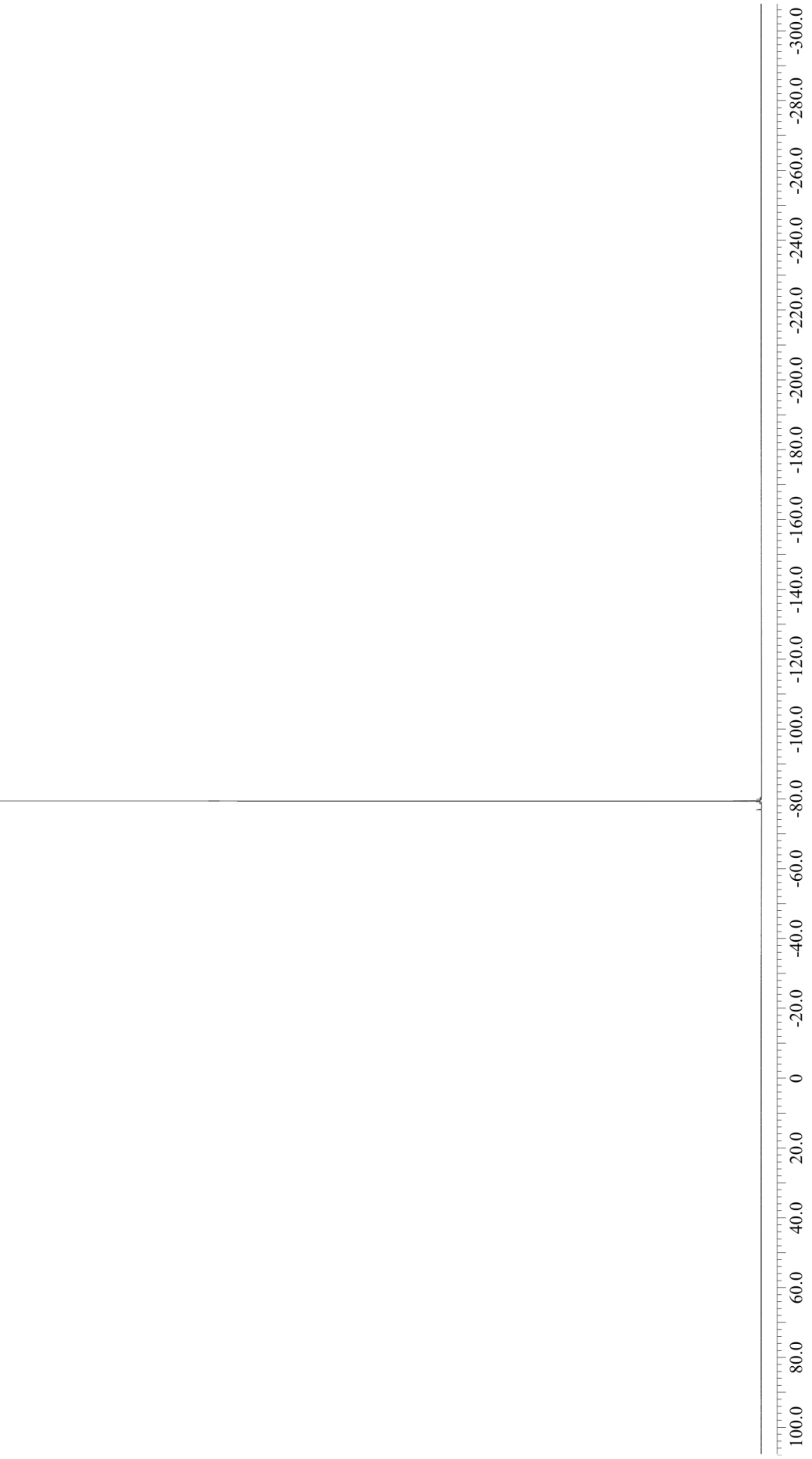

^1H NMR of **9ab** ($CDCl_3$, 400 MHz, 25 °C)

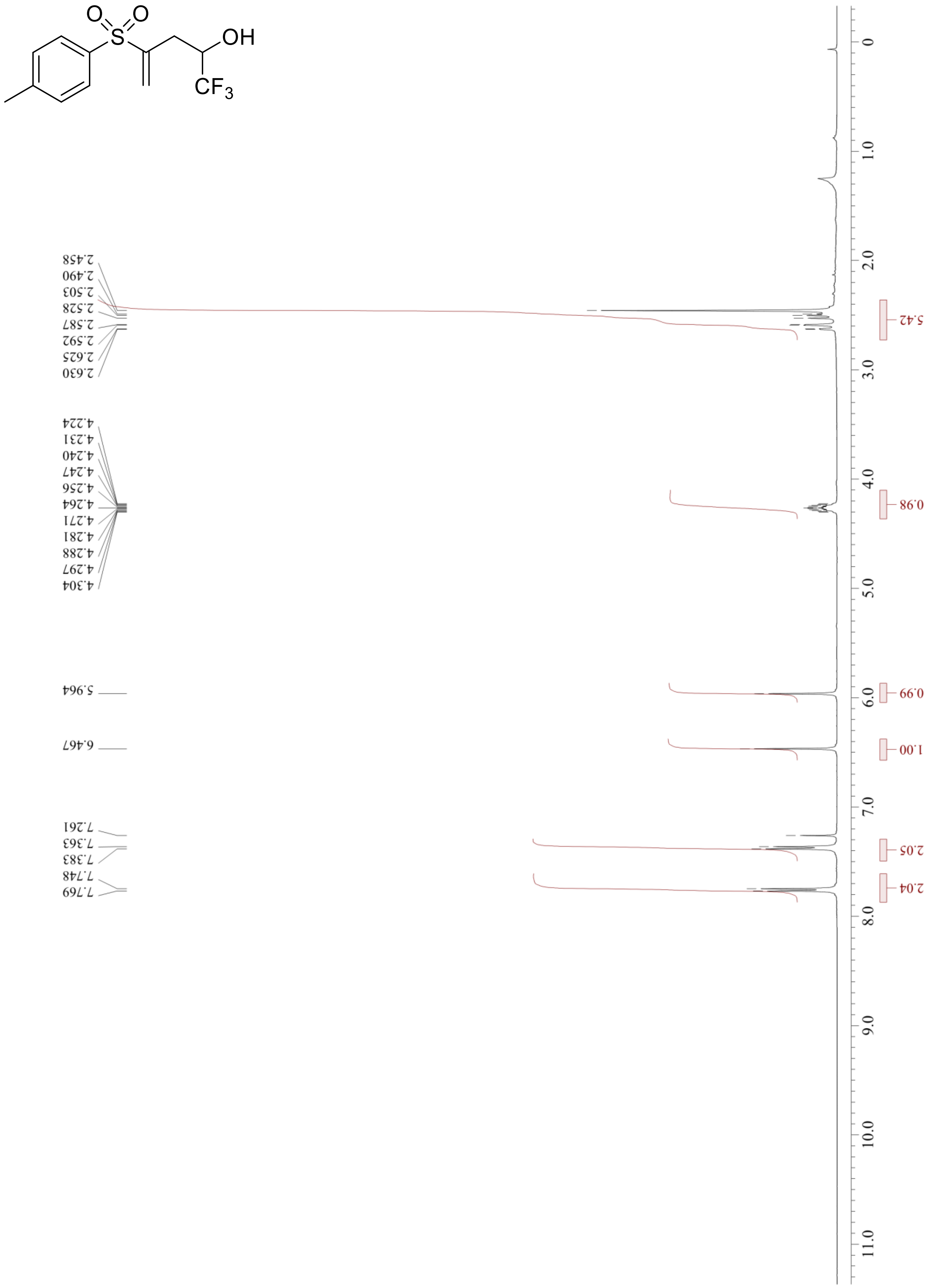

^{13}C NMR of **9ab** ($CDCl_3$, 100 MHz, 25 ºC)

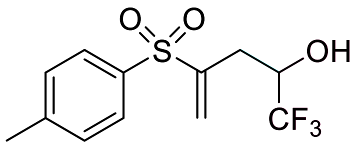

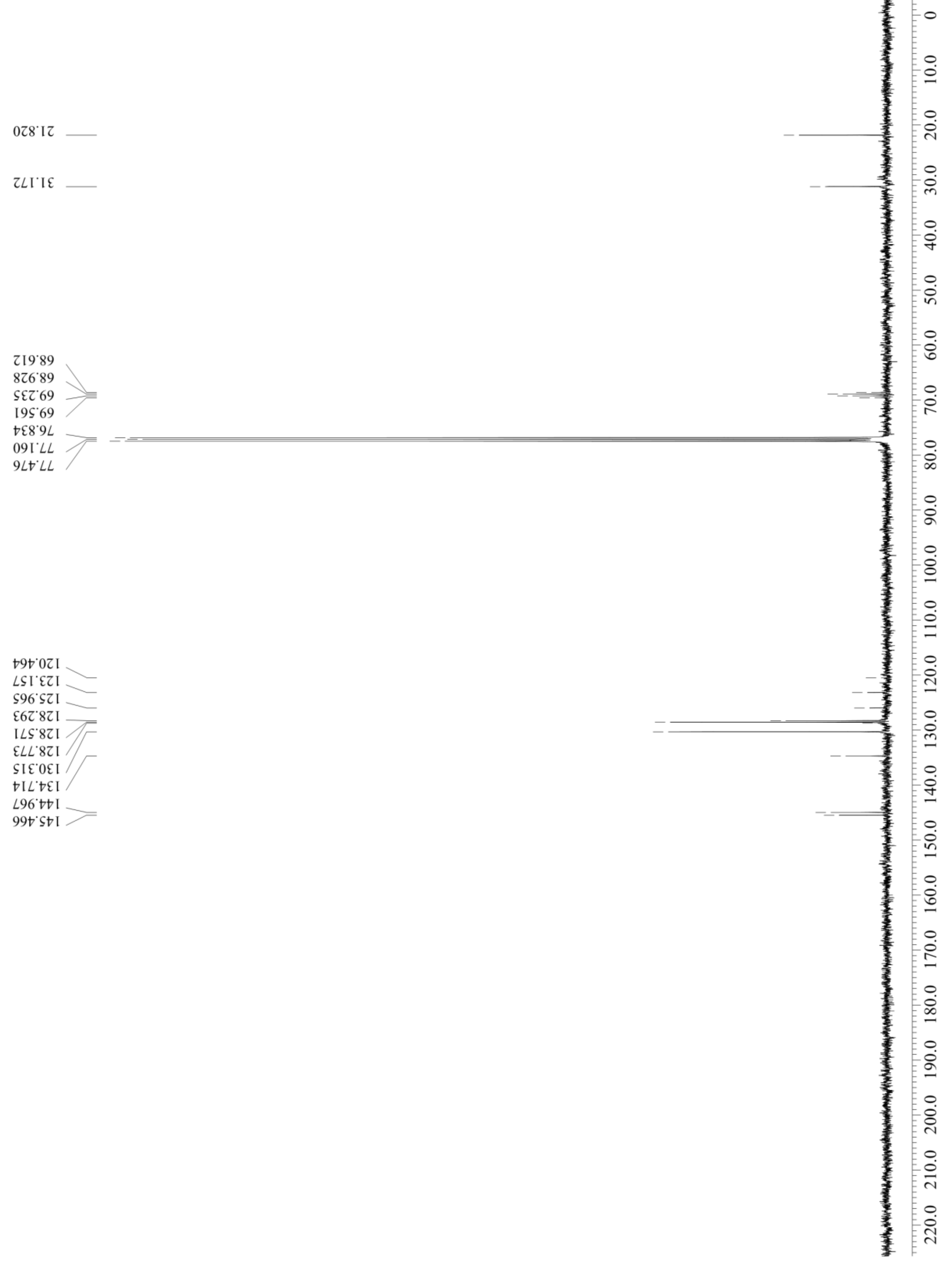

^{19}F NMR of **9ab** ($CDCl_3$, 375 MHz, 25 ºC)

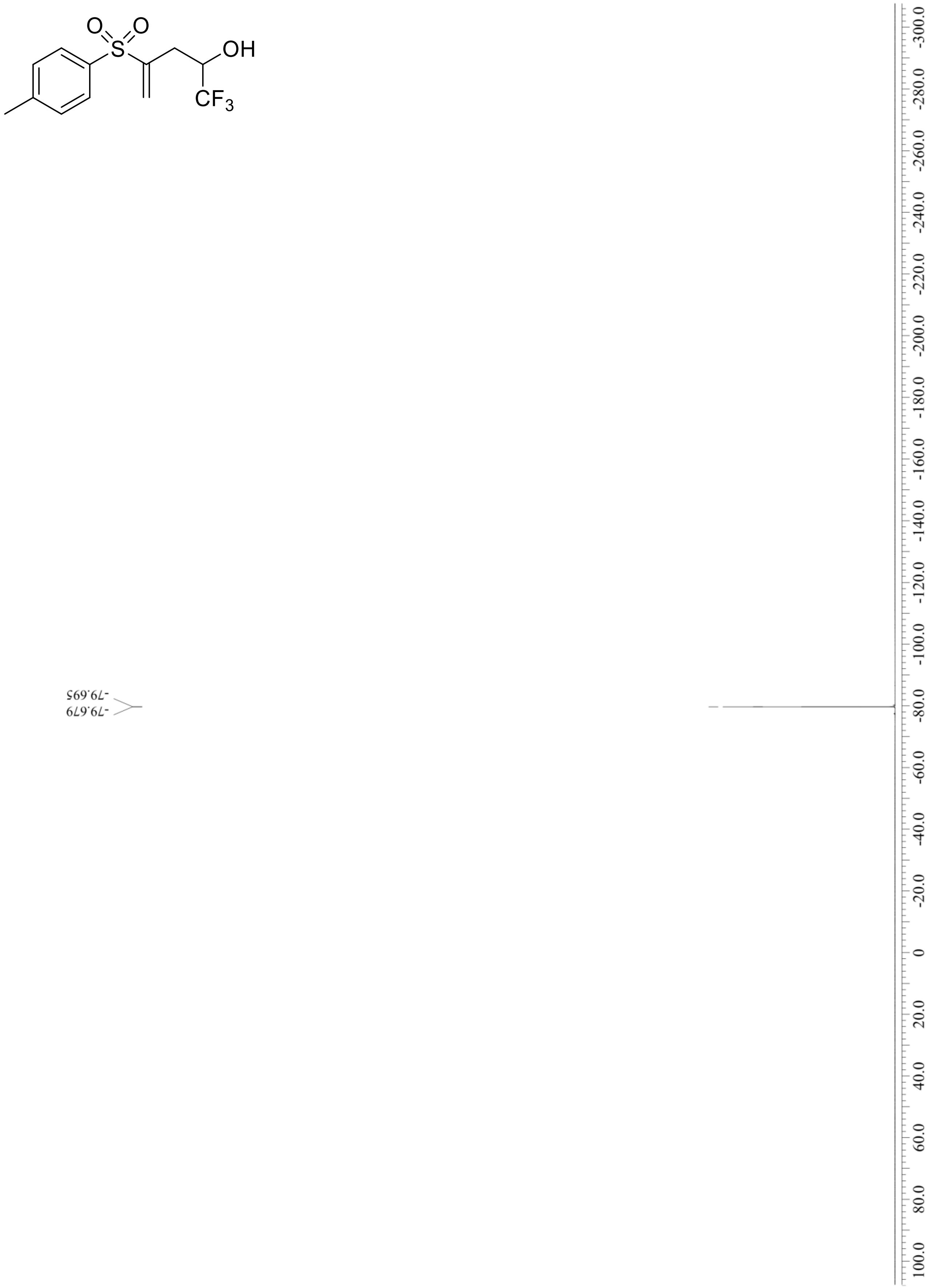

^{1}H NMR of **9ac** ($CDCl_3$, 400 MHz, 25 ºC)

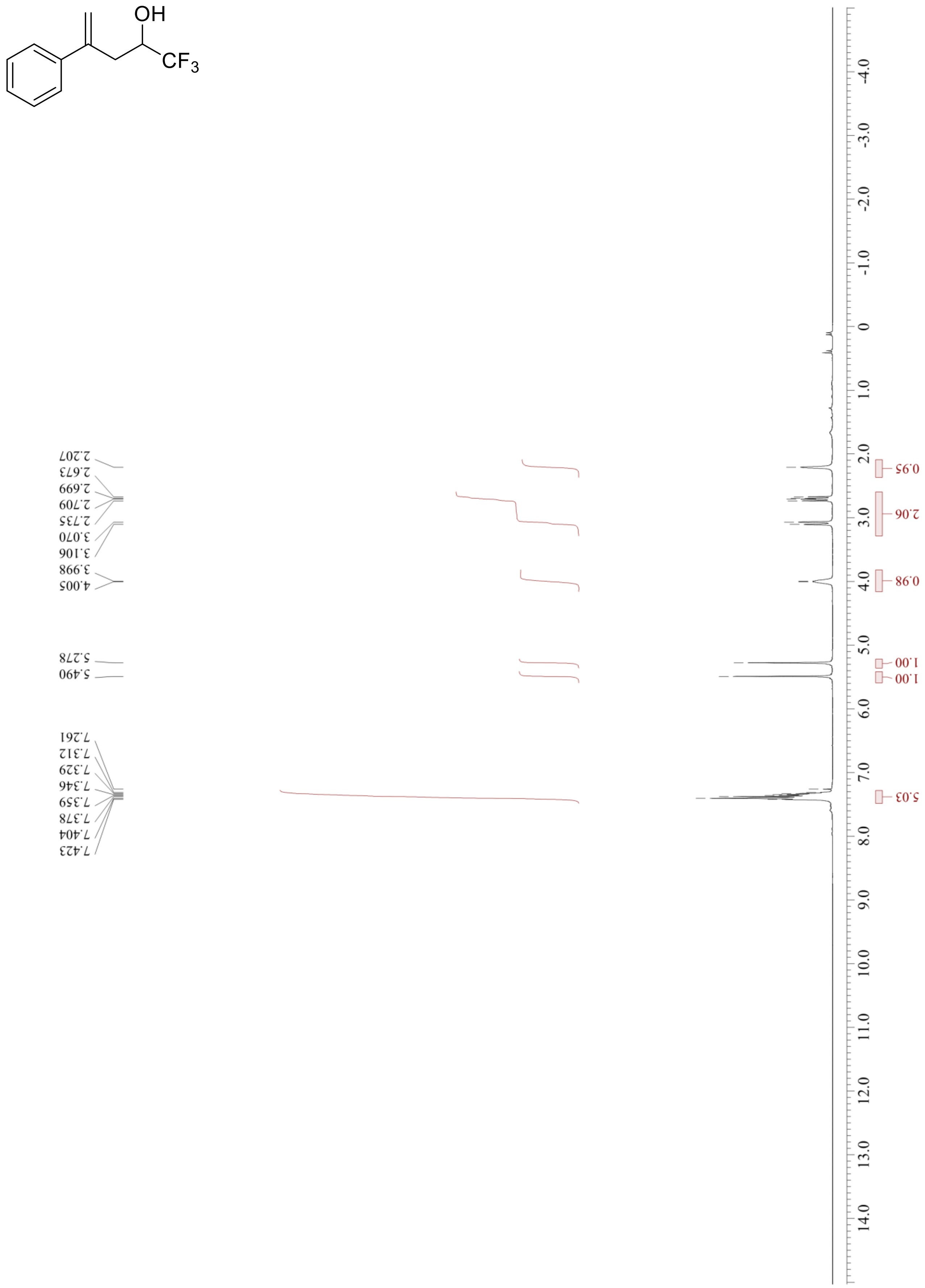

^{13}C NMR of **9ac** ($CDCl_3$, 100 MHz, 25 ºC)

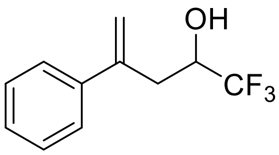

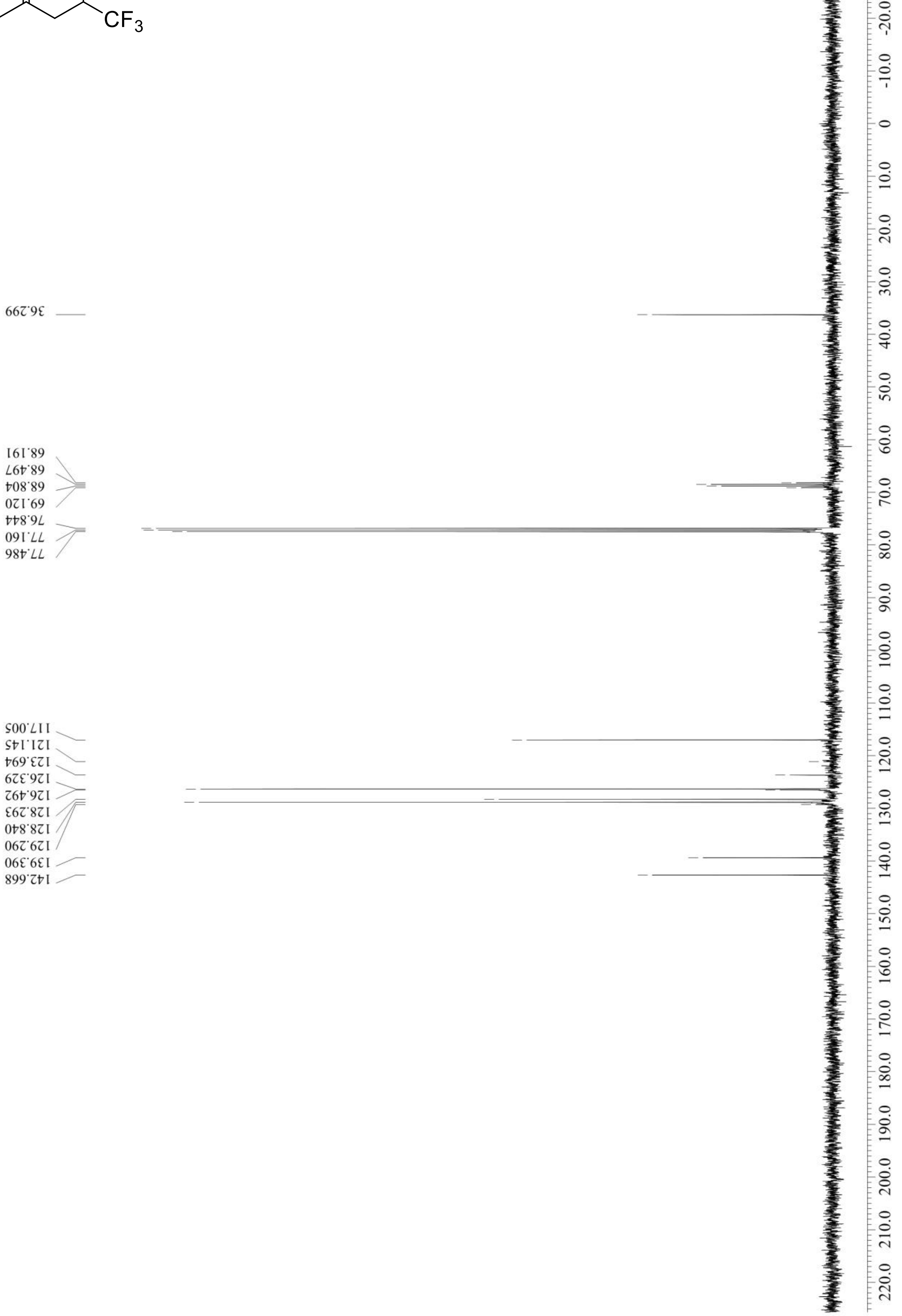

^{19}F NMR of **9ac** ($CDCl_3$, 375 MHz, 25 ºC)

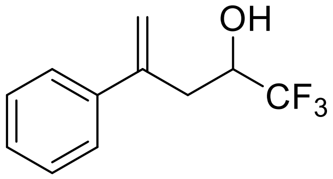

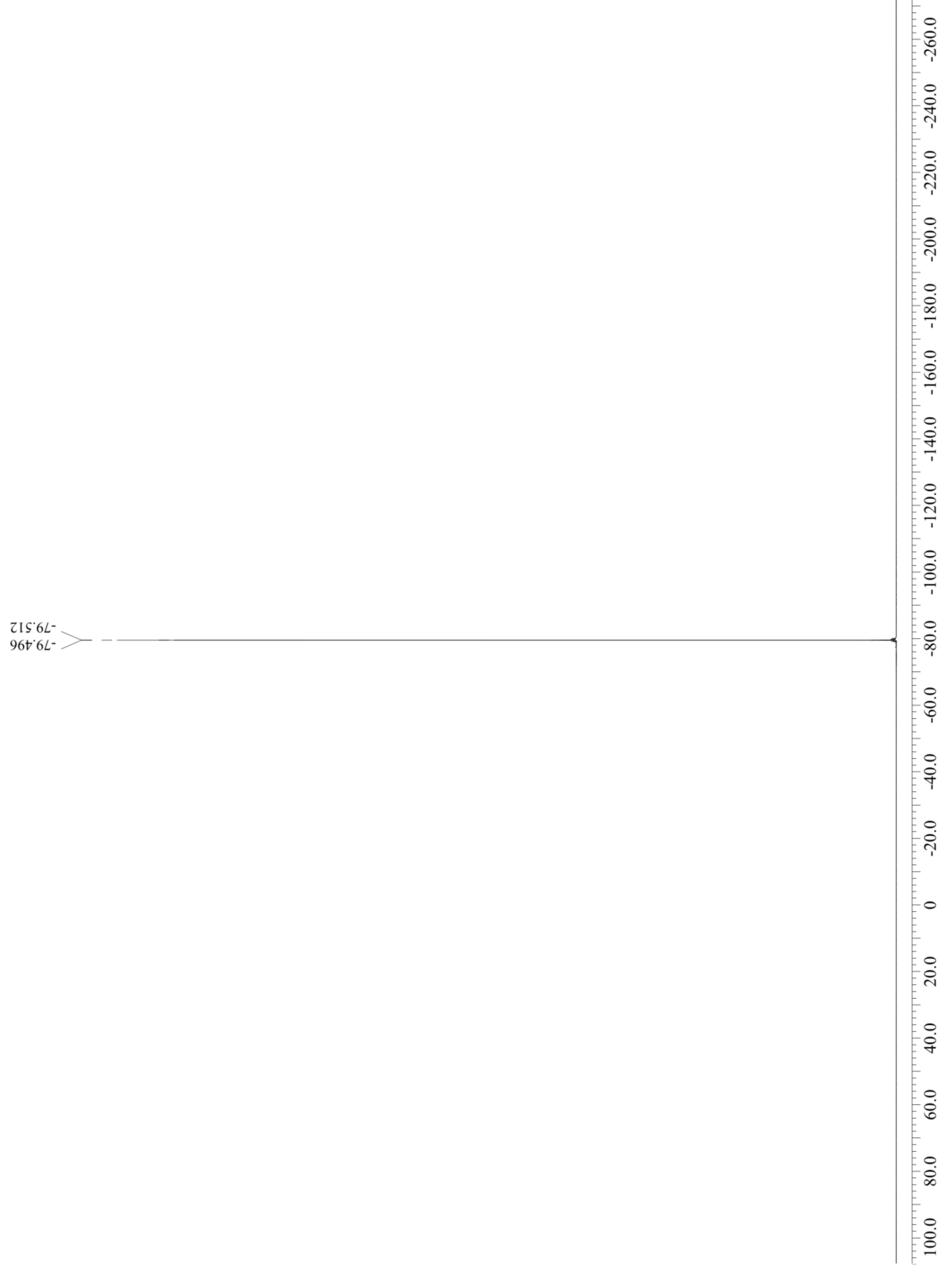

^{1}H NMR of **9ad** ($CDCl_3$, 400 MHz, 25 °C)

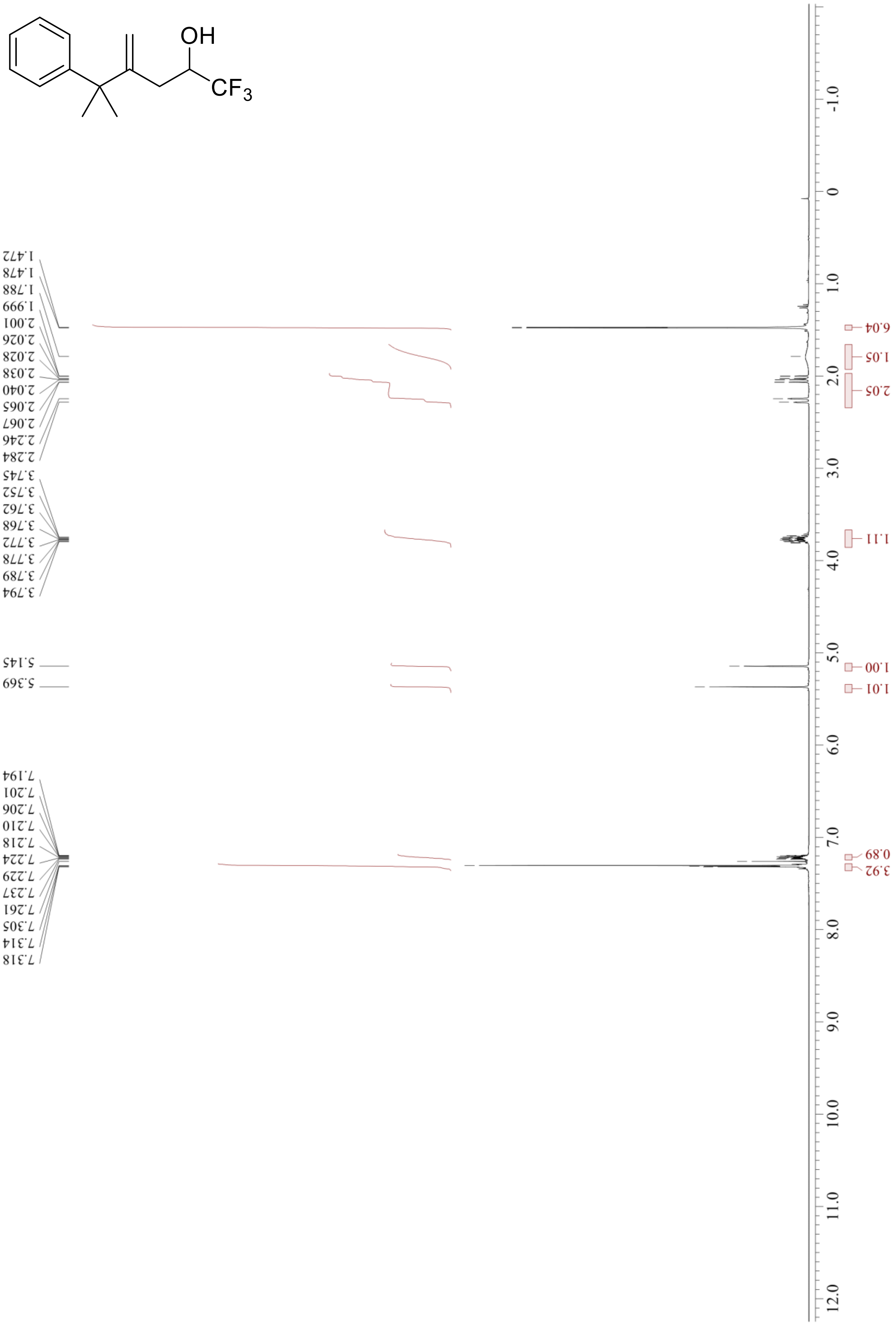

^{13}C NMR of **9ad** ($CDCl_3$, 100 MHz, 25 ºC)

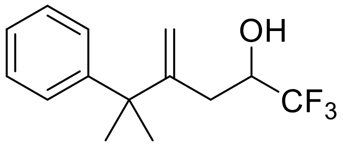

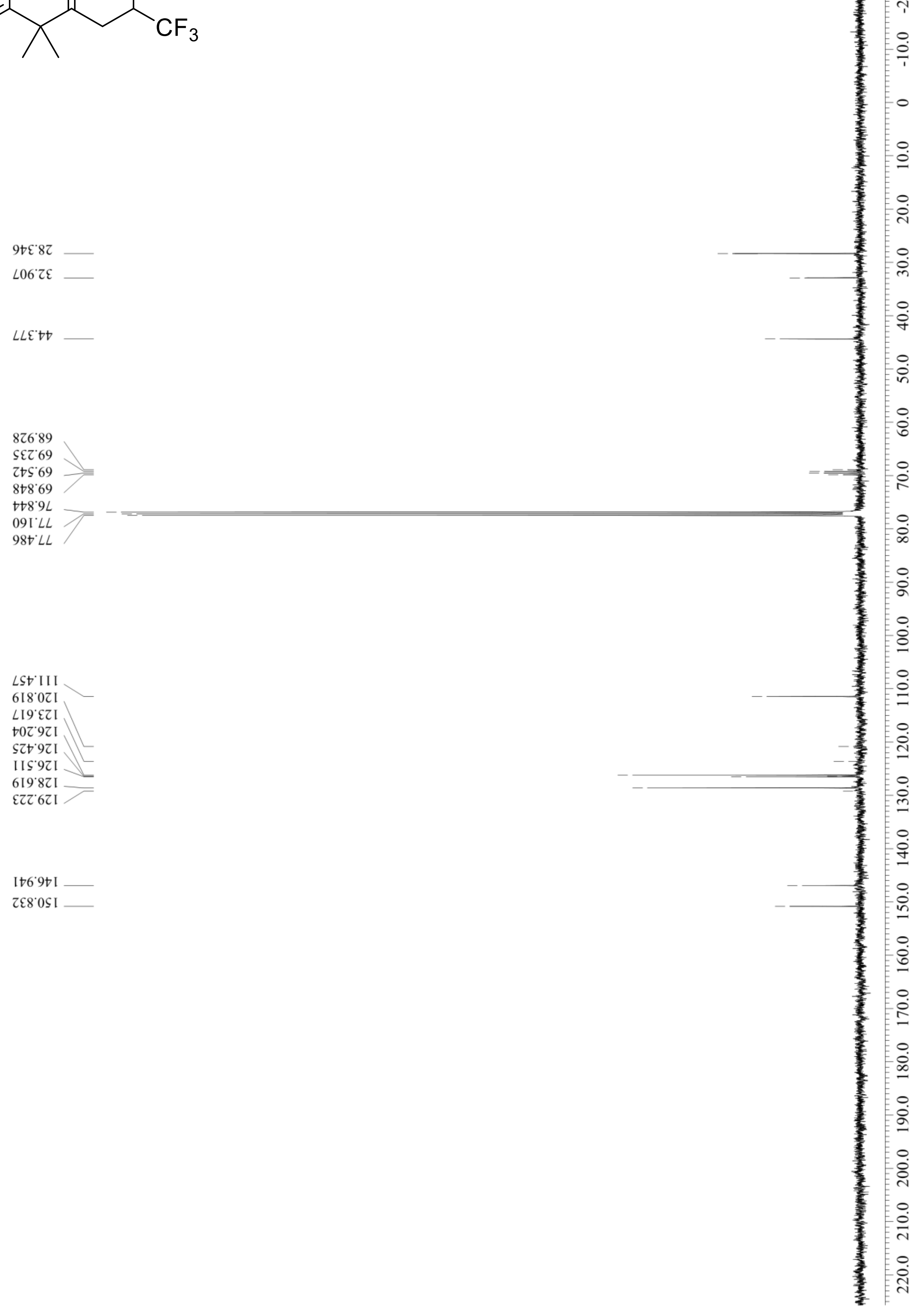

^{19}F NMR of **9ad** ($CDCl_3$, 375 MHz, 25 ºC)

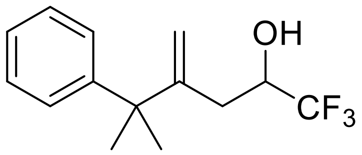

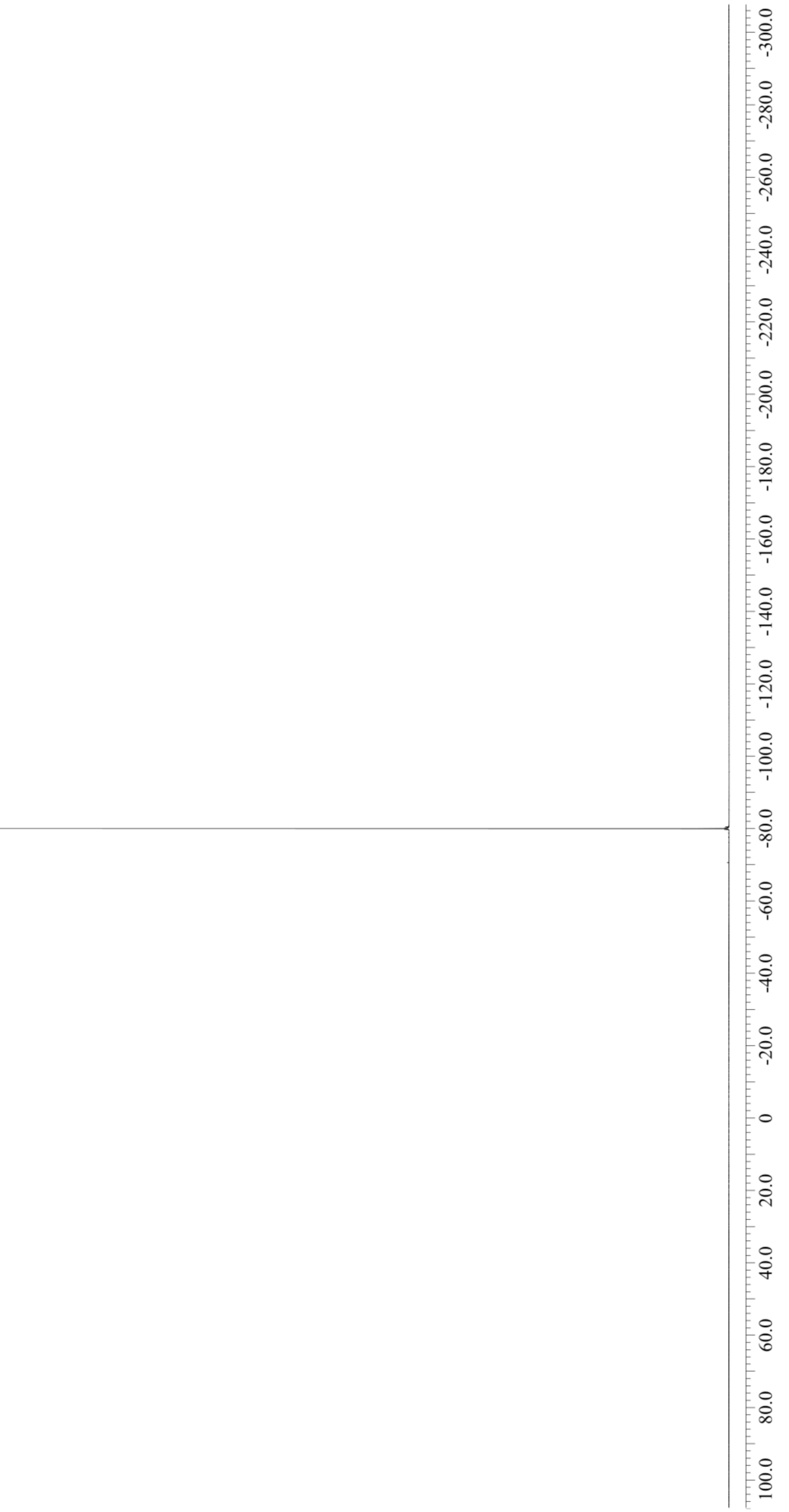

^{1}H NMR of **9ae** ($CDCl_3$, 400 MHz, 25 ºC)

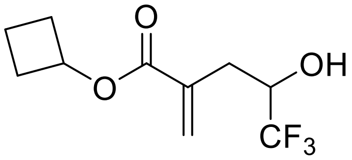

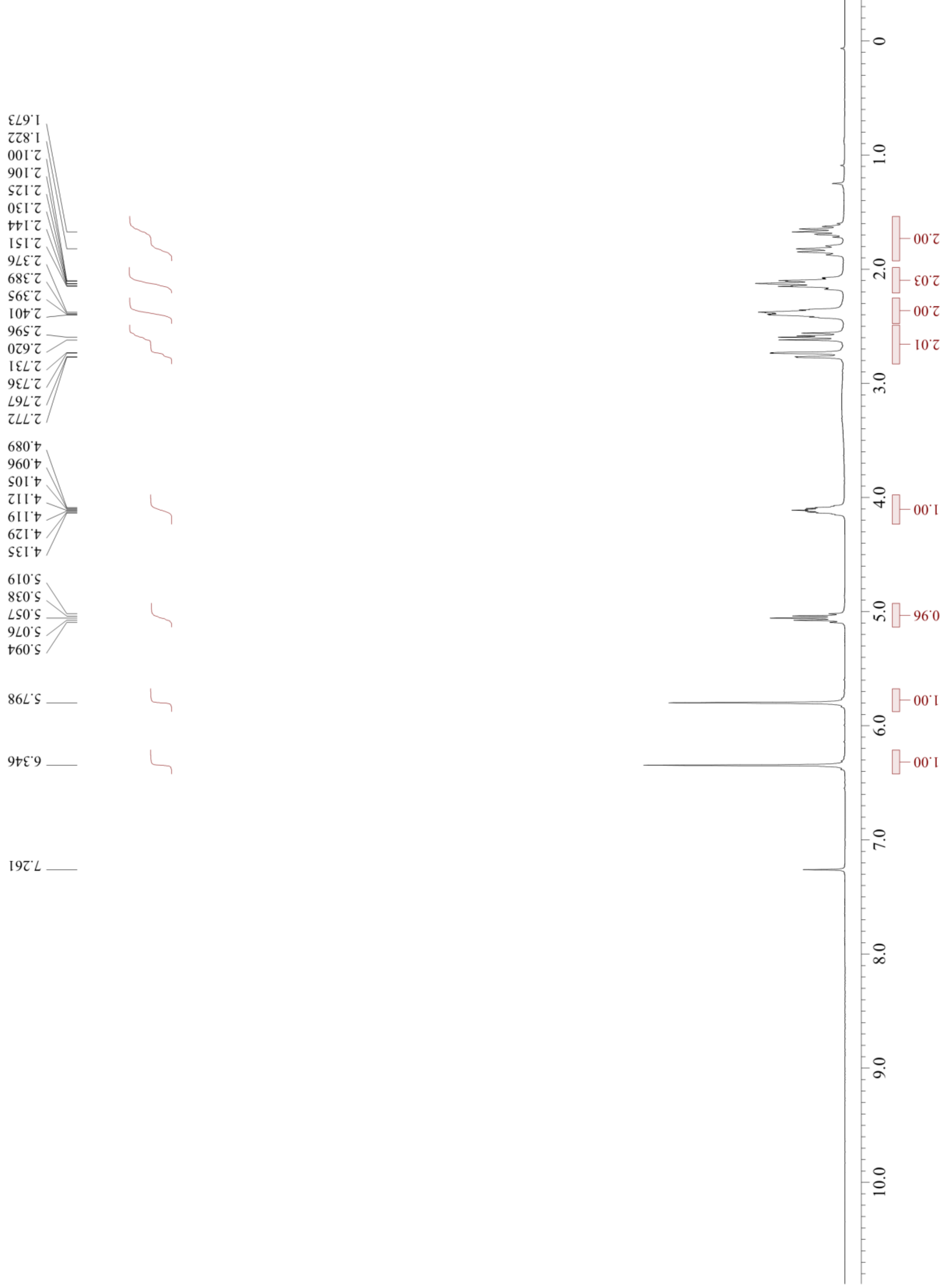

^{13}C NMR of **9ae** ($CDCl_3$, 100 MHz, 25 ºC)

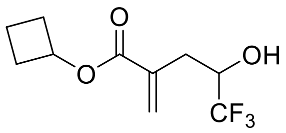

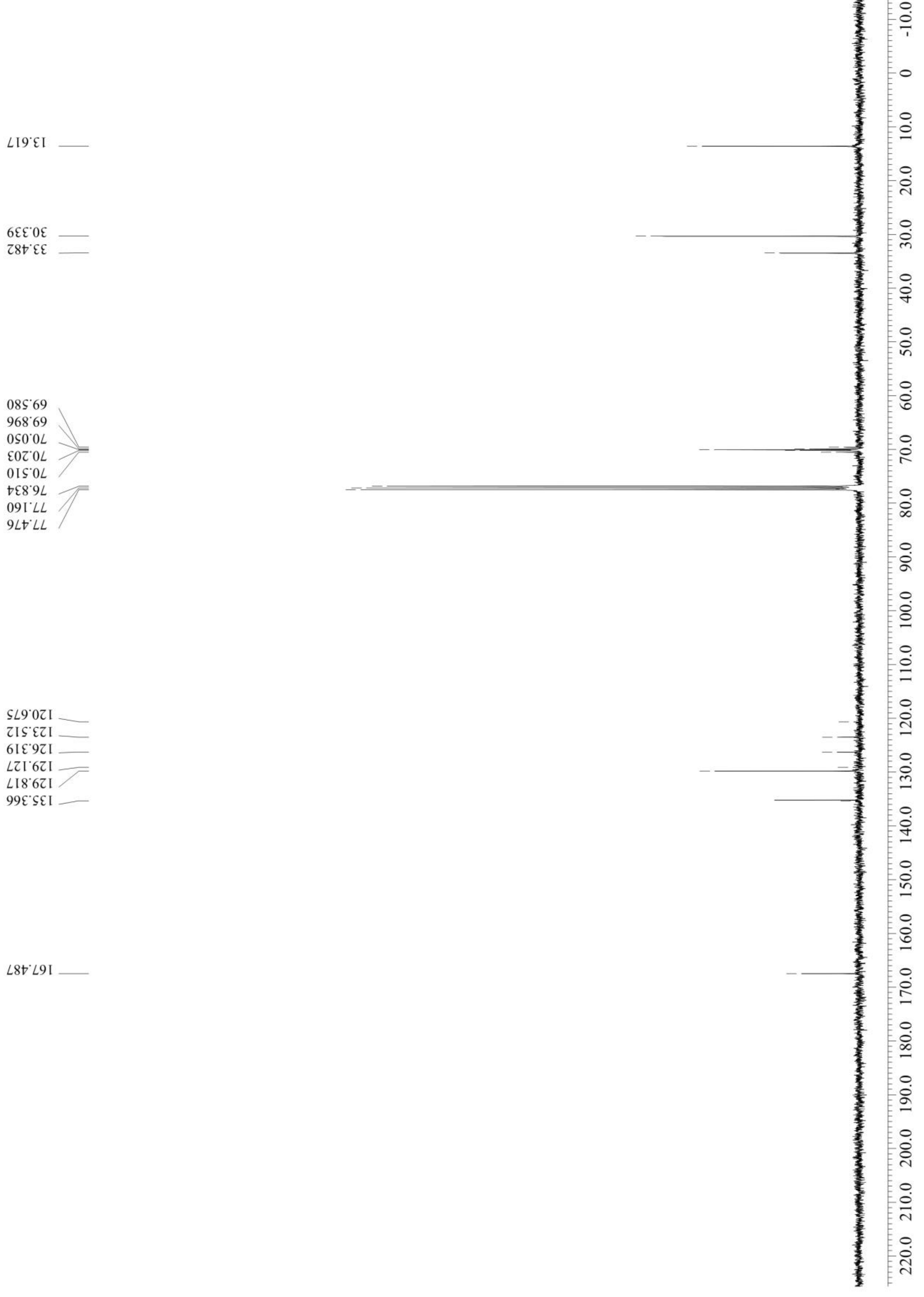

^{19}F NMR of **9ae** ($CDCl_3$, 375 MHz, 25 ºC)

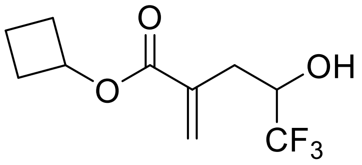

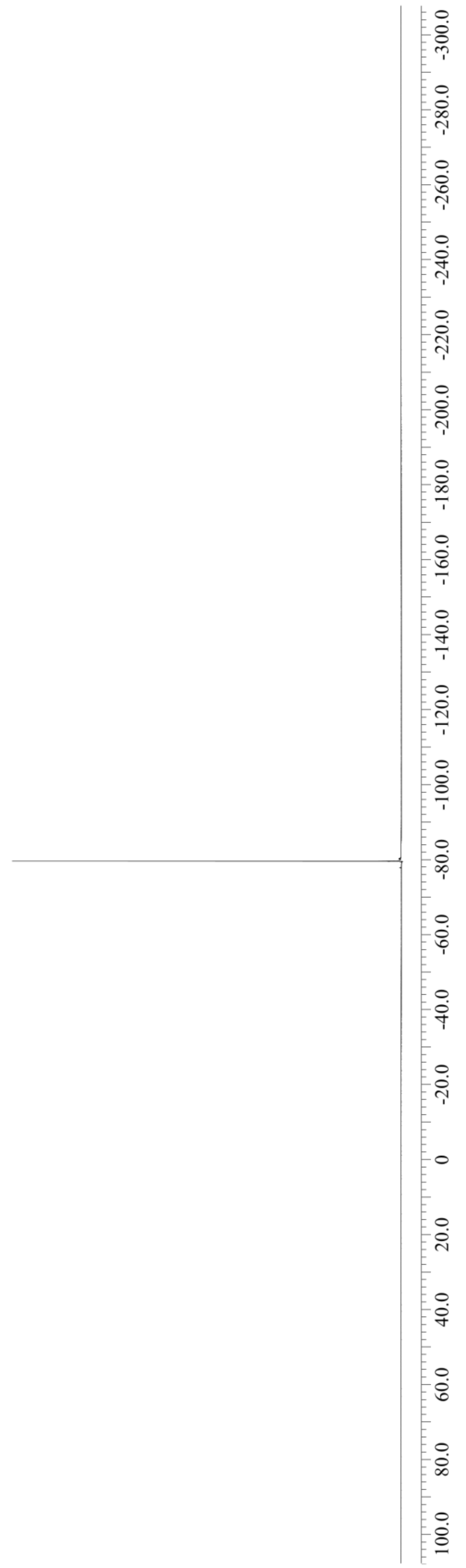

^{1}H NMR of **9af** ($CDCl_3$, 400 MHz, 25 ºC)

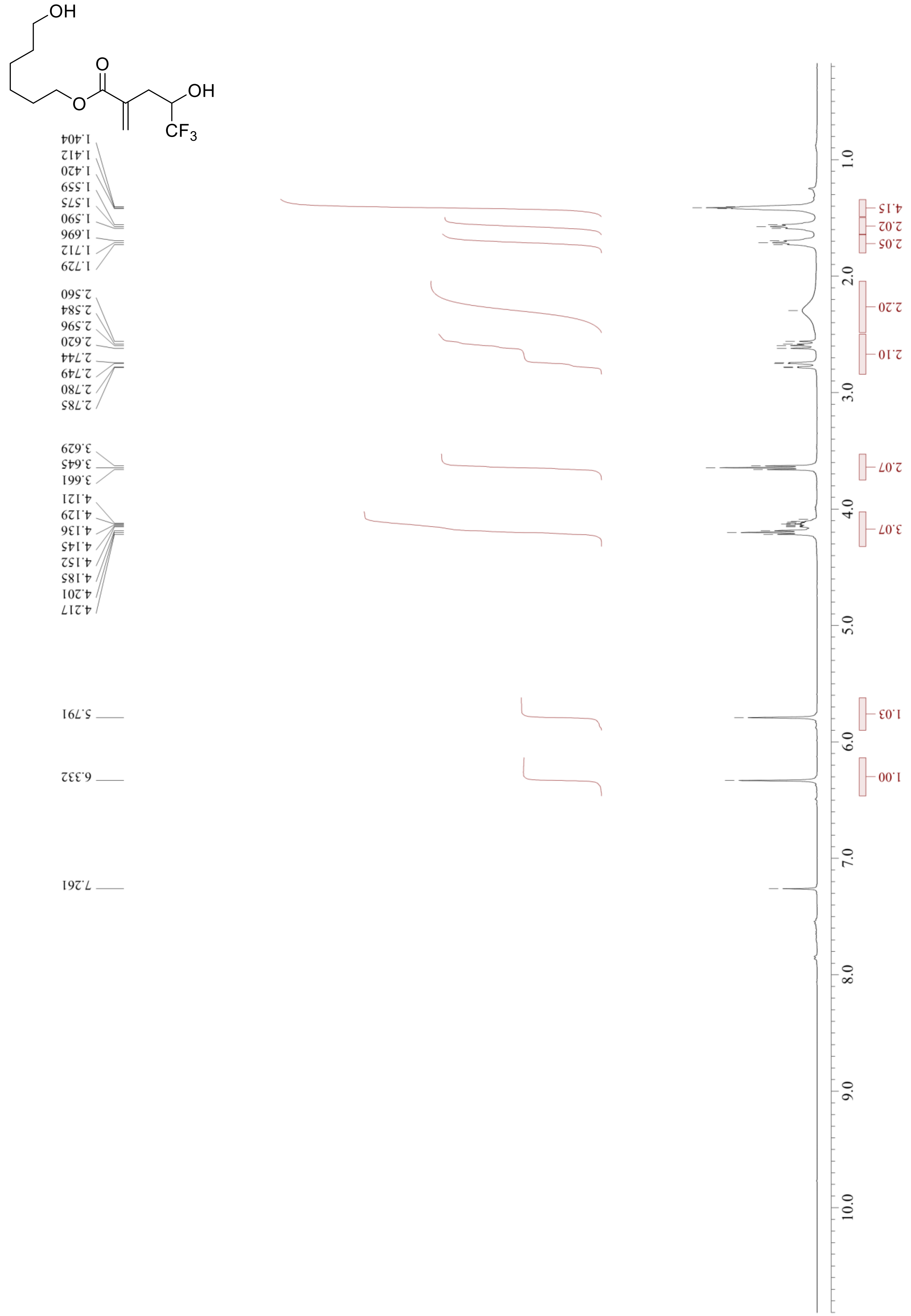

^{13}C NMR of **9af** ($CDCl_3$, 100 MHz, 25 ºC)

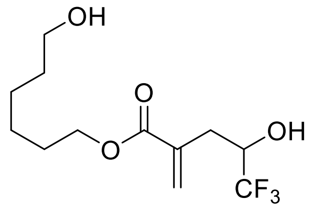

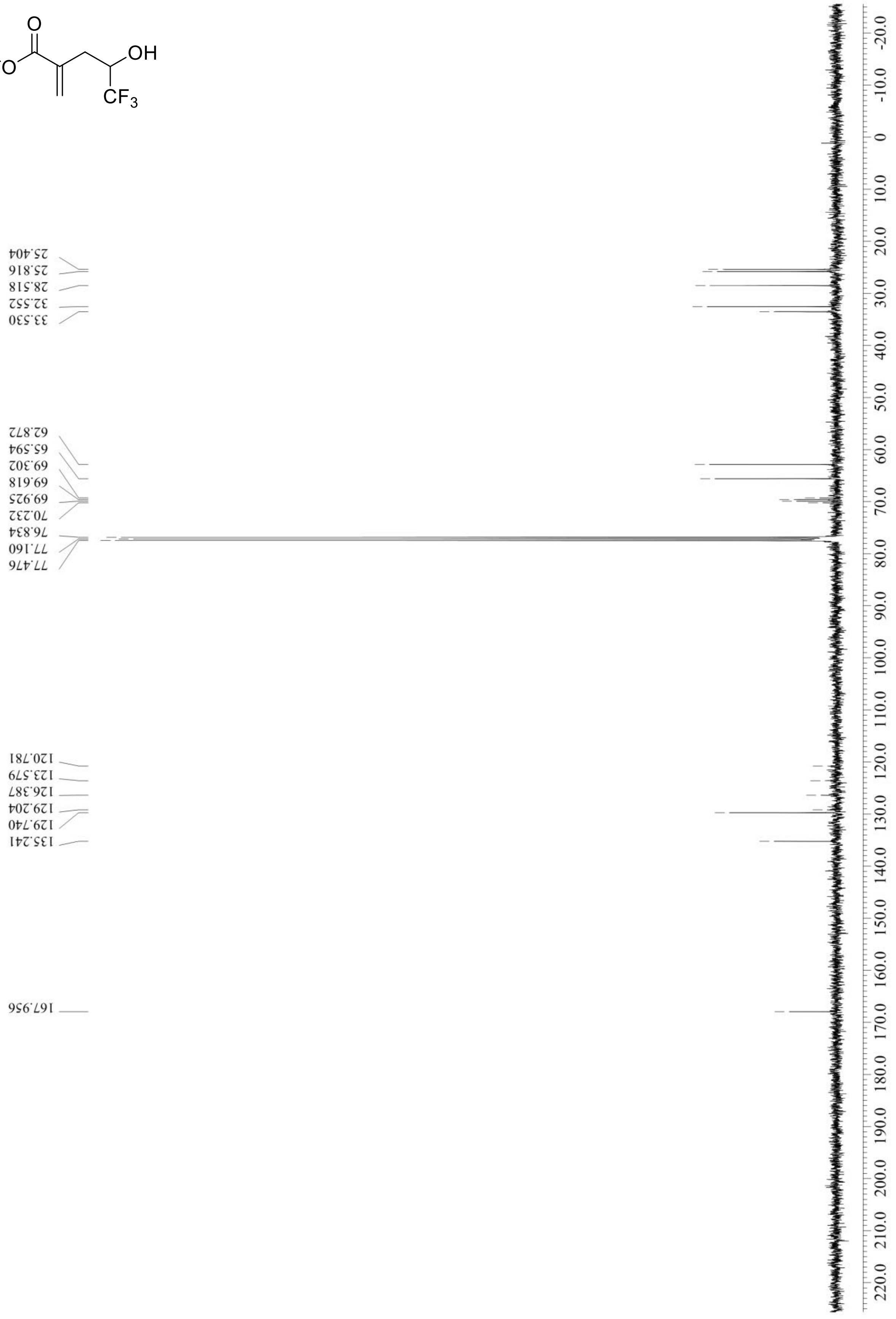

^{19}F NMR of **9af** ($CDCl_3$, 375 MHz, 25 ºC)

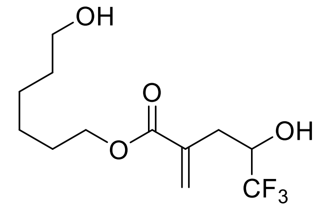

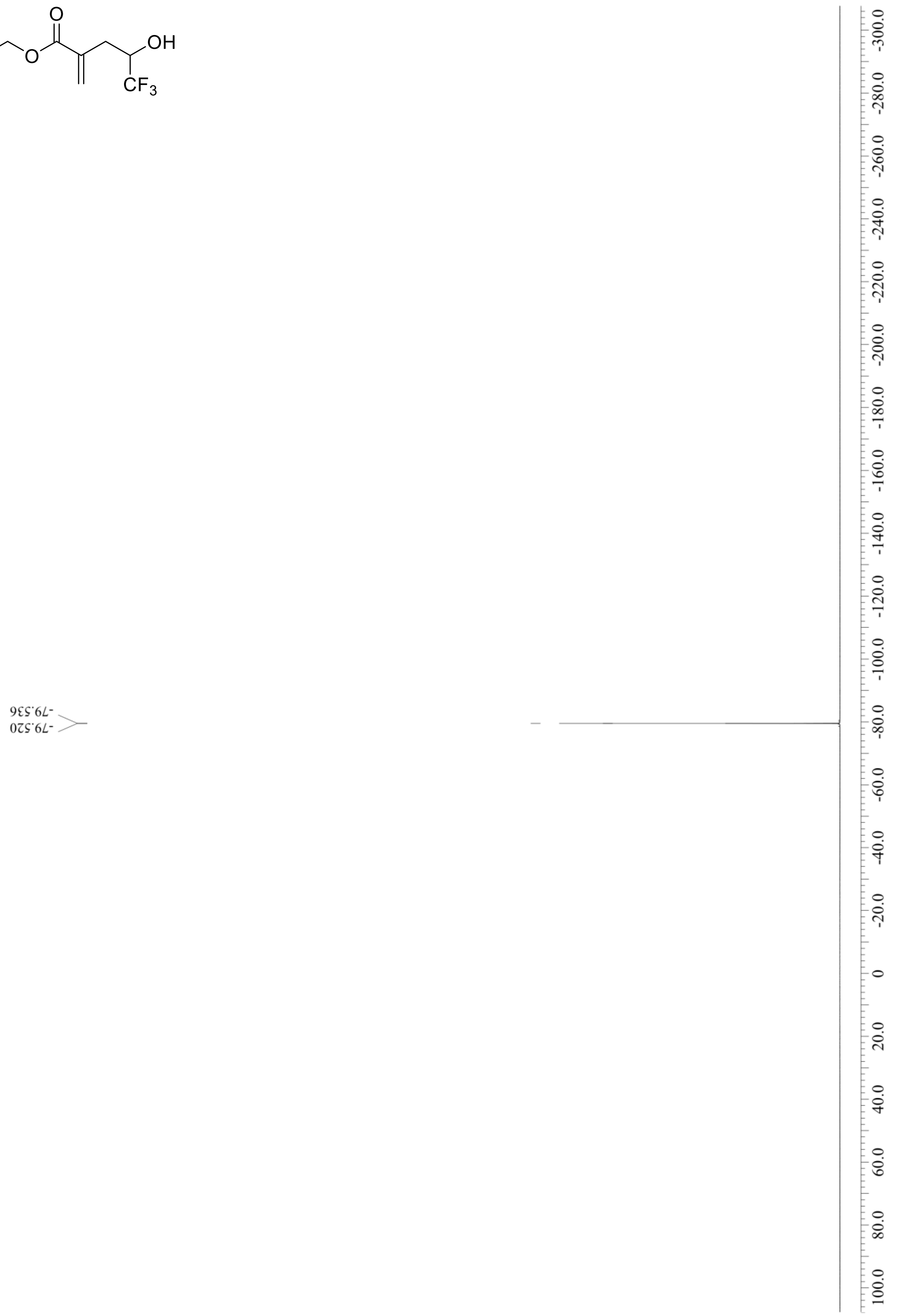

^{1}H NMR of **9ag** ($CDCl_3$, 400 MHz, 25 ºC)

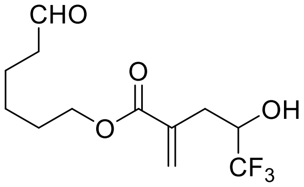

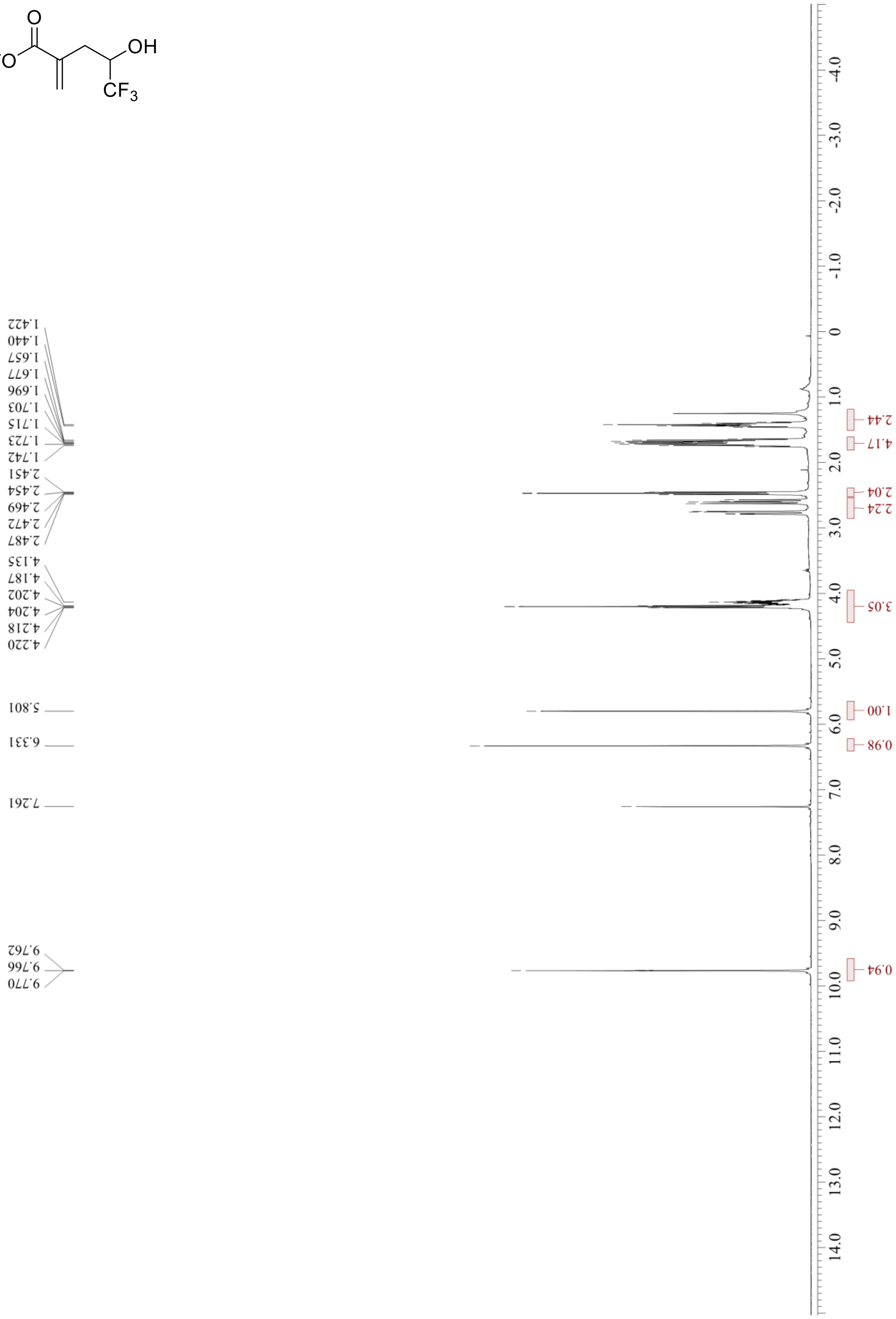

^{13}C NMR of **9ag** ($CDCl_3$, 100 MHz, 25 ºC)

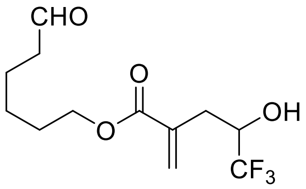

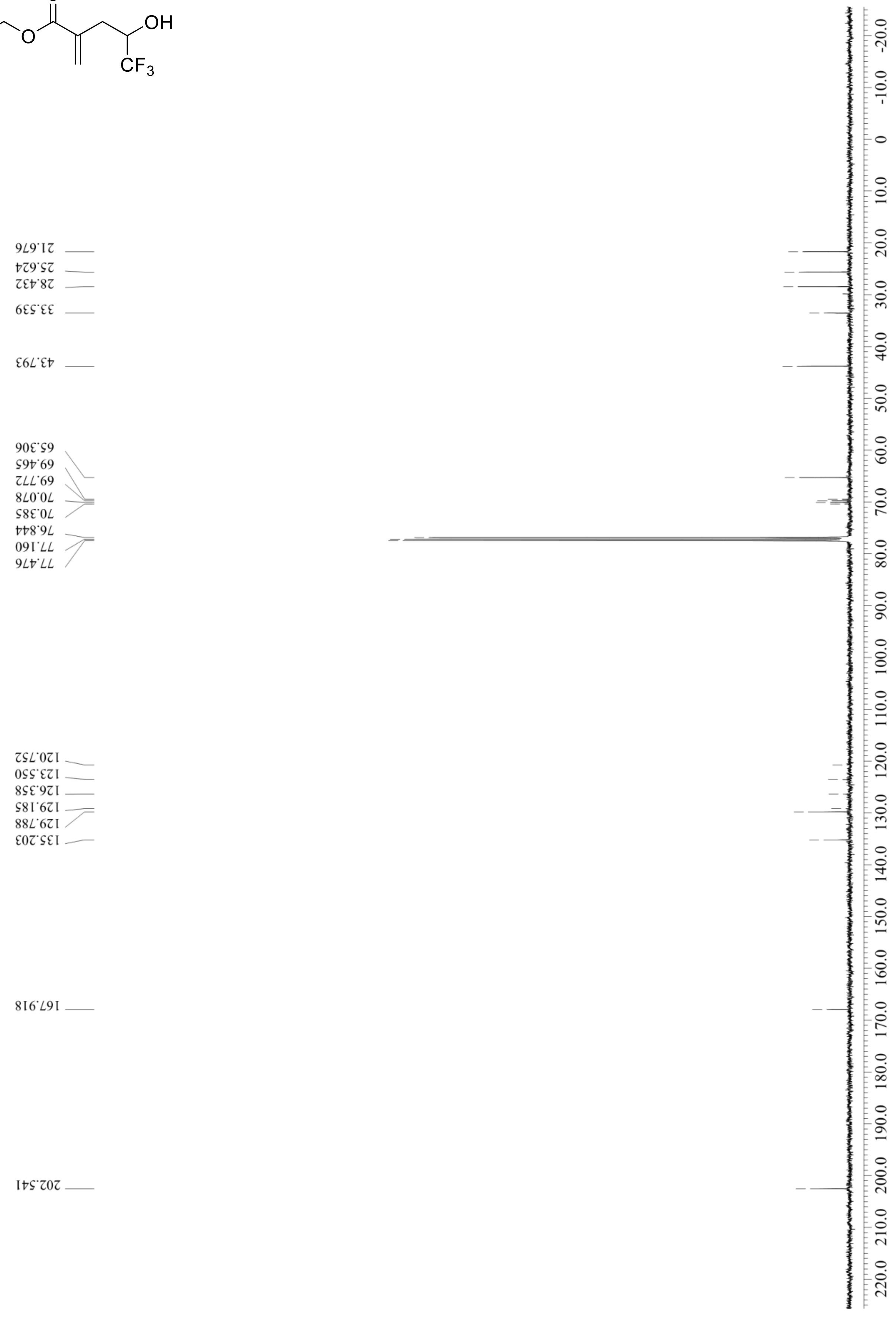

^{19}F NMR of **9ag** ($CDCl_3$, 375 MHz, 25 ºC)

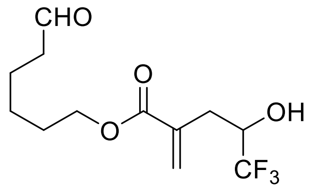

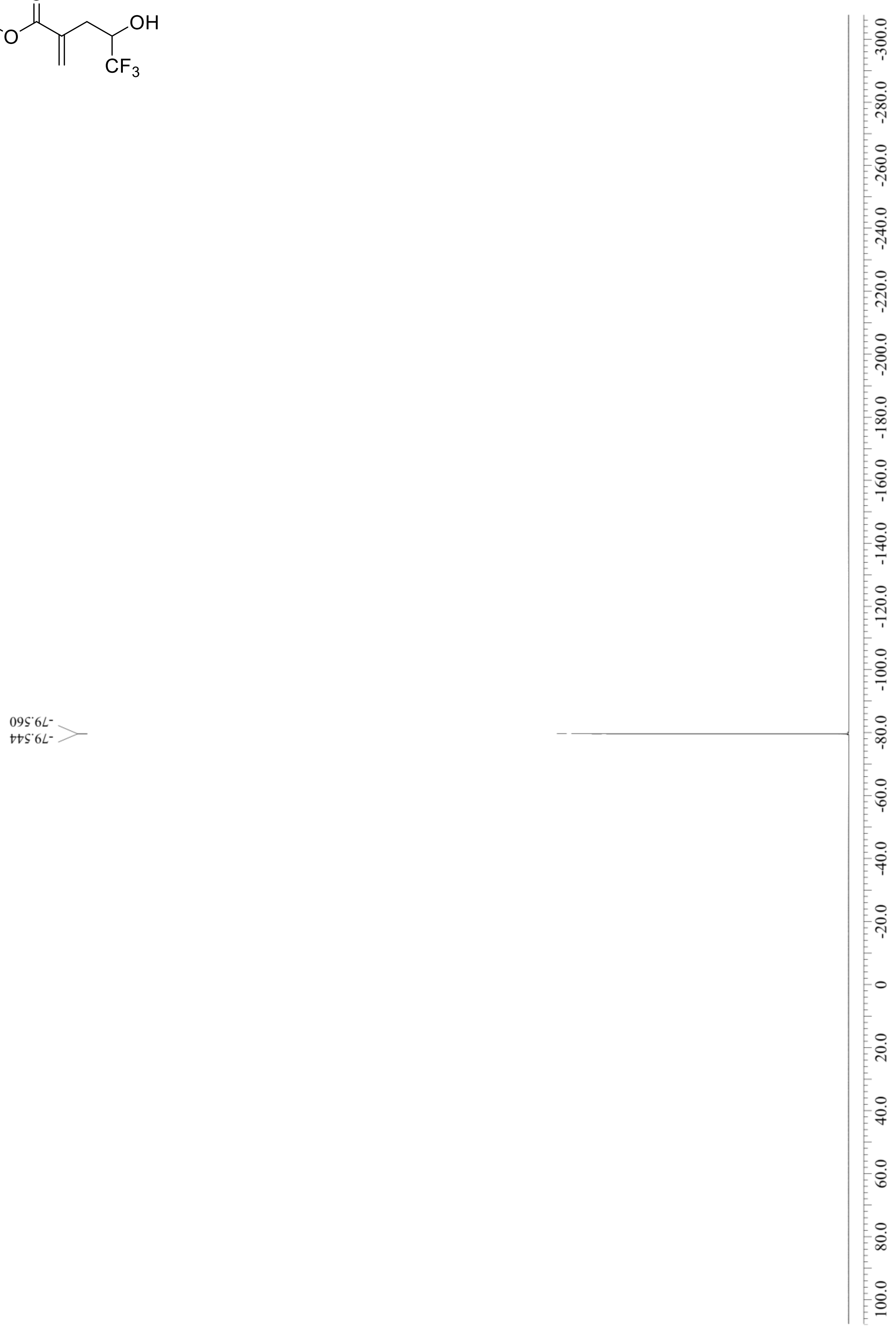

^{1}H NMR of **9ah** ($CDCl_3$, 400 MHz, 25 ºC)

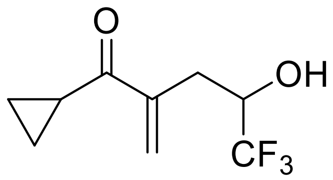

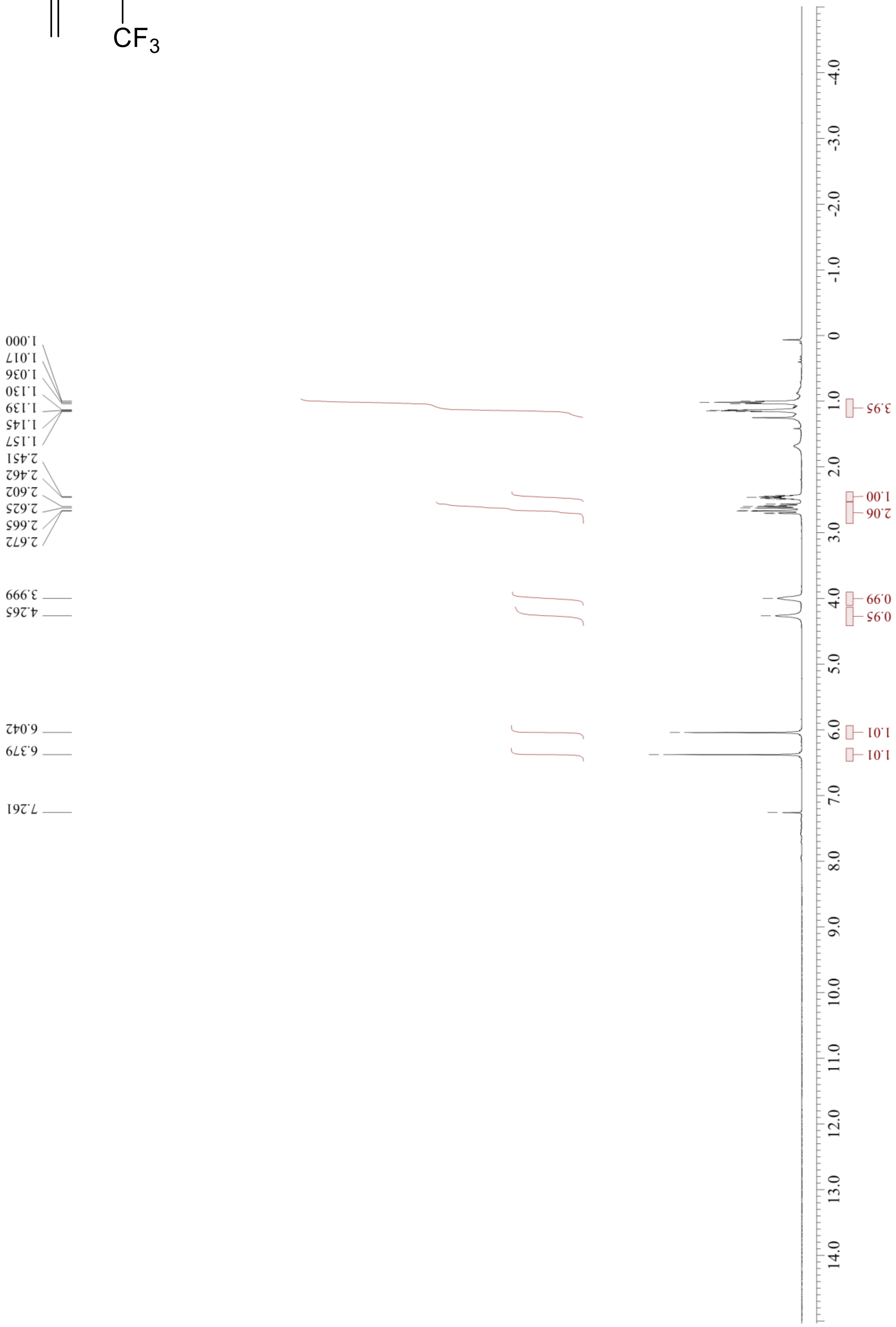

^{13}C NMR of **9ah** ($CDCl_3$, 100 MHz, 25 ºC)

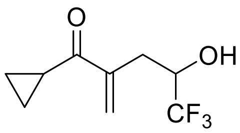

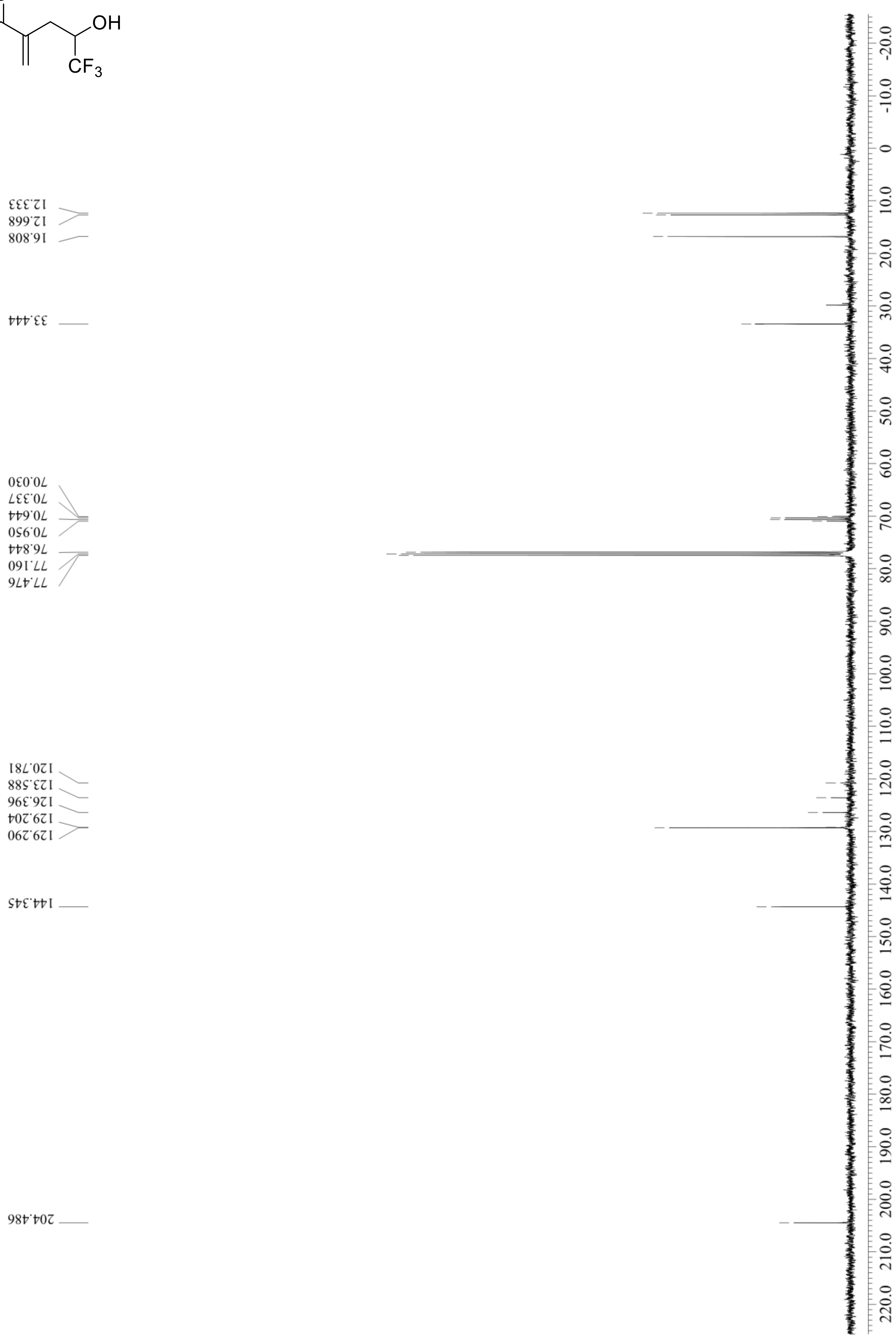

^{19}F NMR of **9ah** ($CDCl_3$, 375 MHz, 25 ºC)

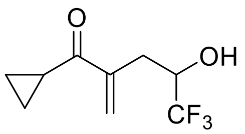

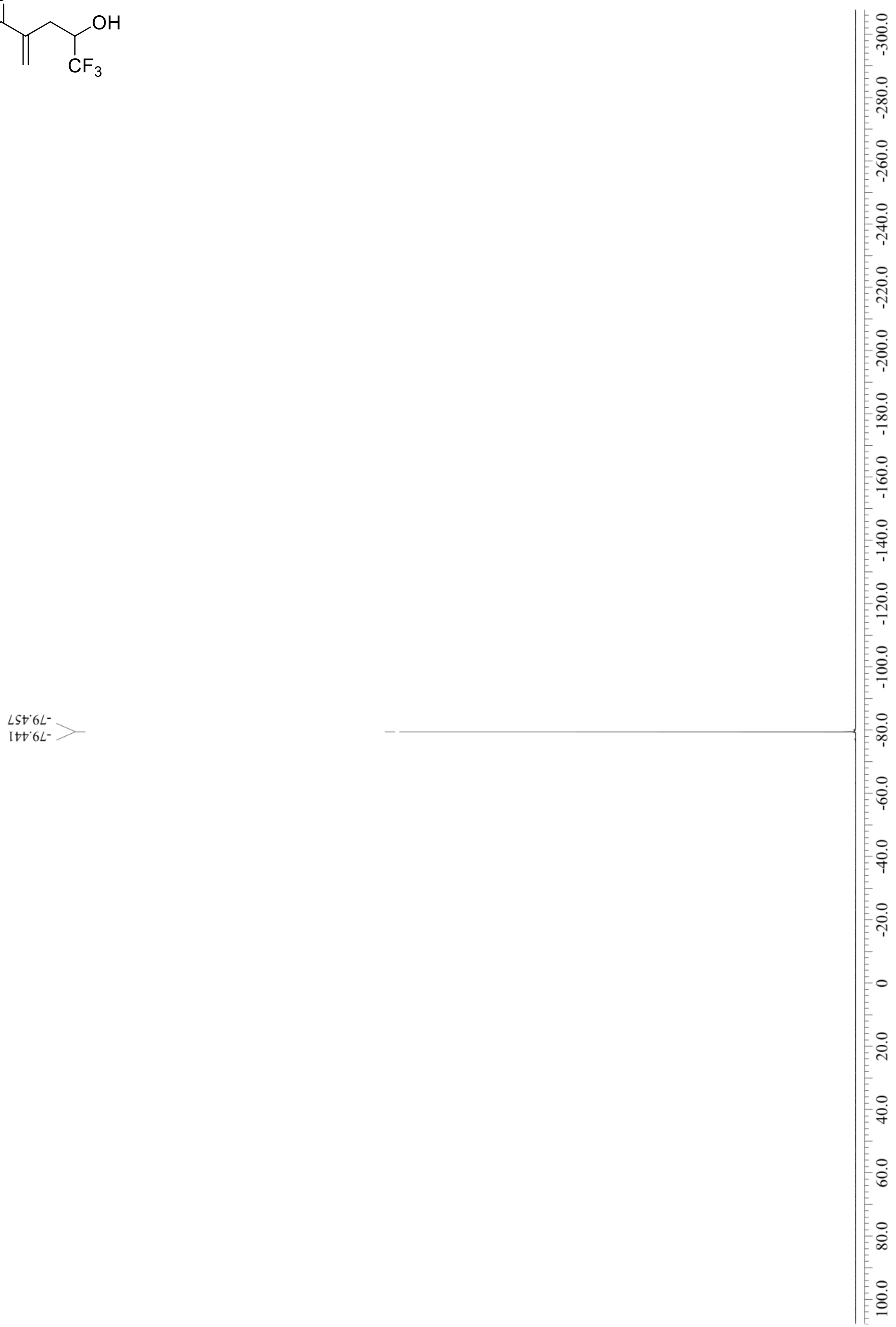

^{1}H NMR of **9ai** ($CDCl_3$, 400 MHz, 25 ºC)

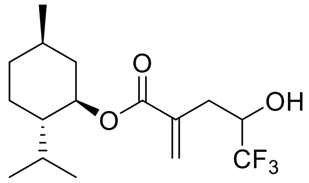

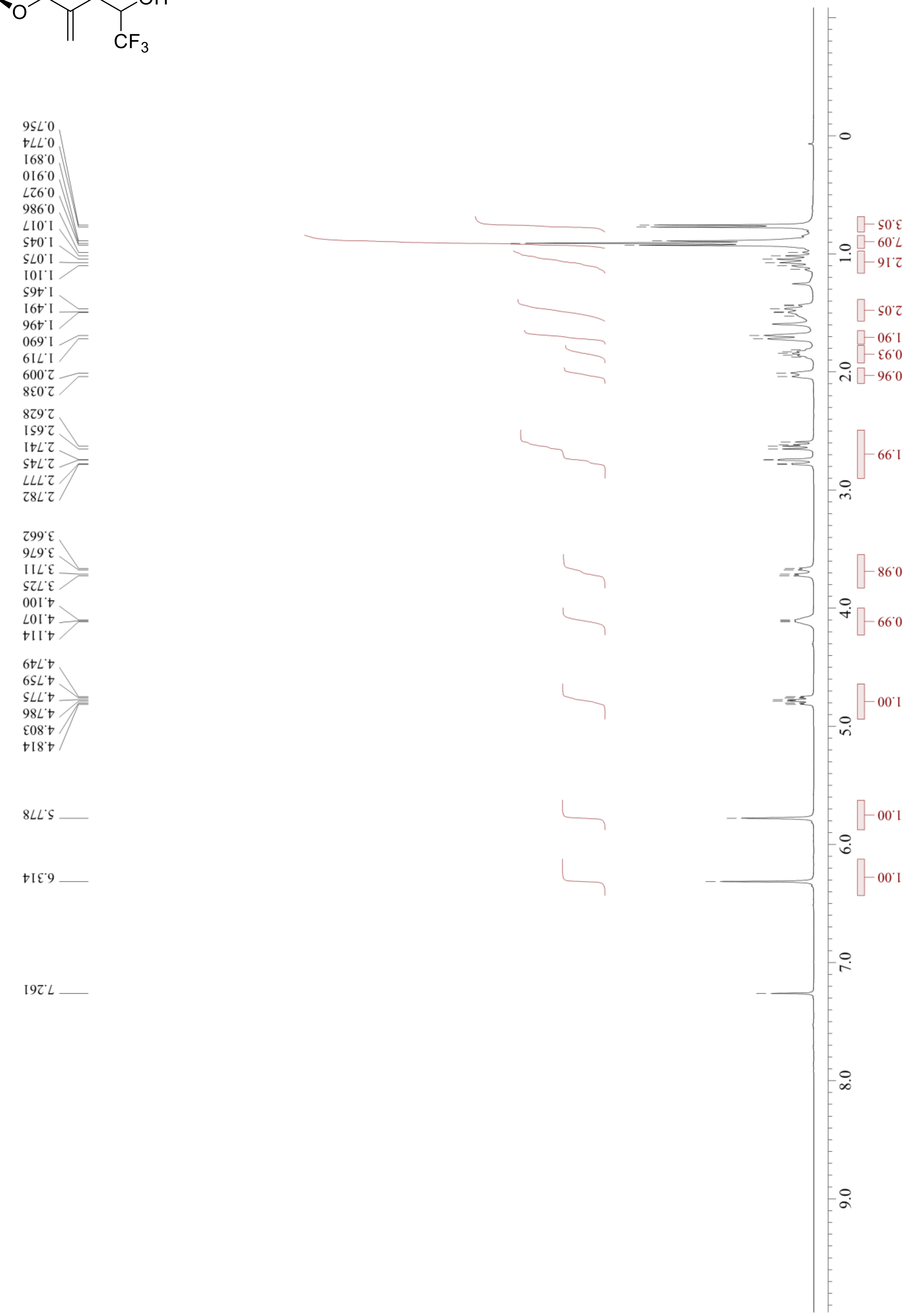

^{13}C NMR of **9ai** ($CDCl_3$, 100 MHz, 25 °C)

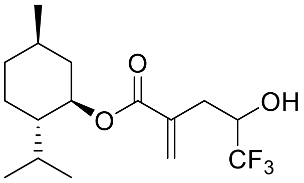

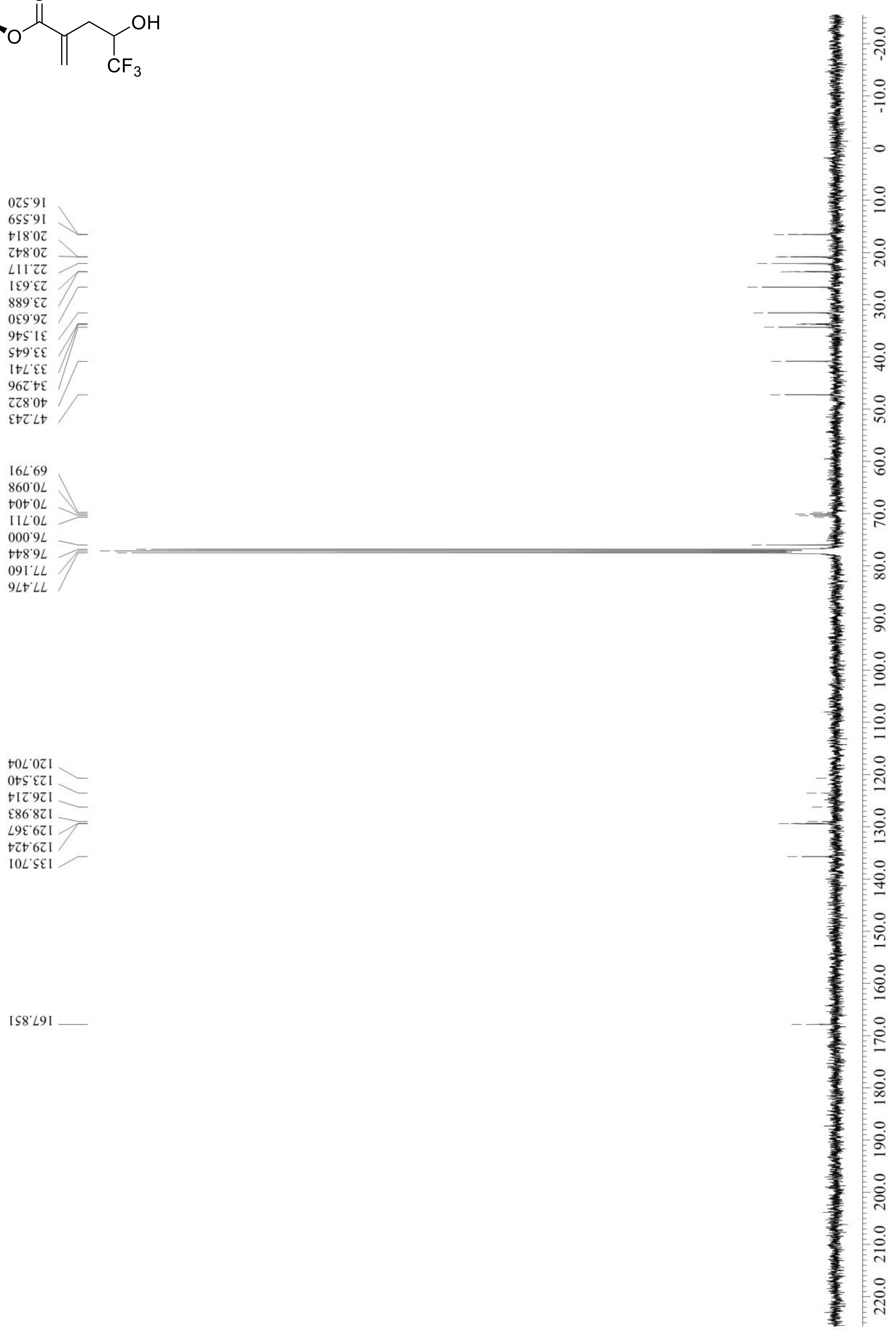

^{19}F NMR of **9ai** ($CDCl_3$, 375 MHz, 25 ºC)

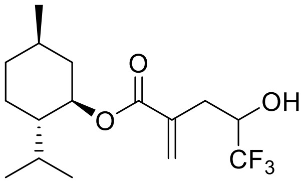

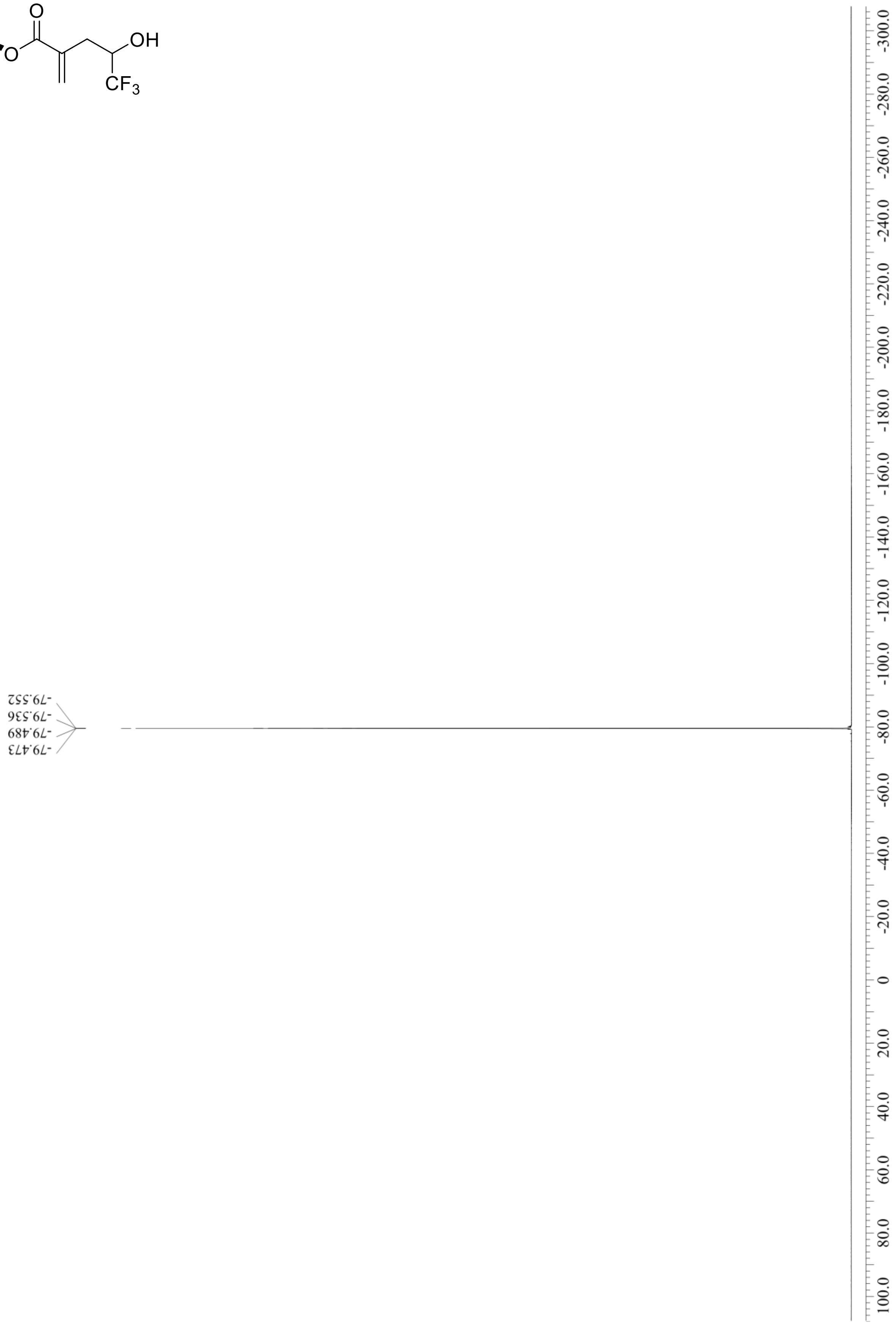

^{1}H NMR of **9aj** ($CDCl_3$, 400 MHz, 25 ºC)

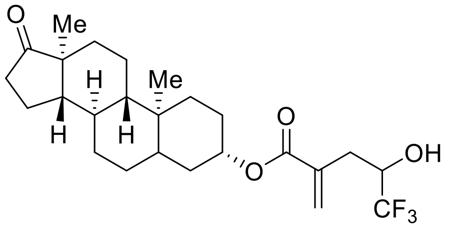

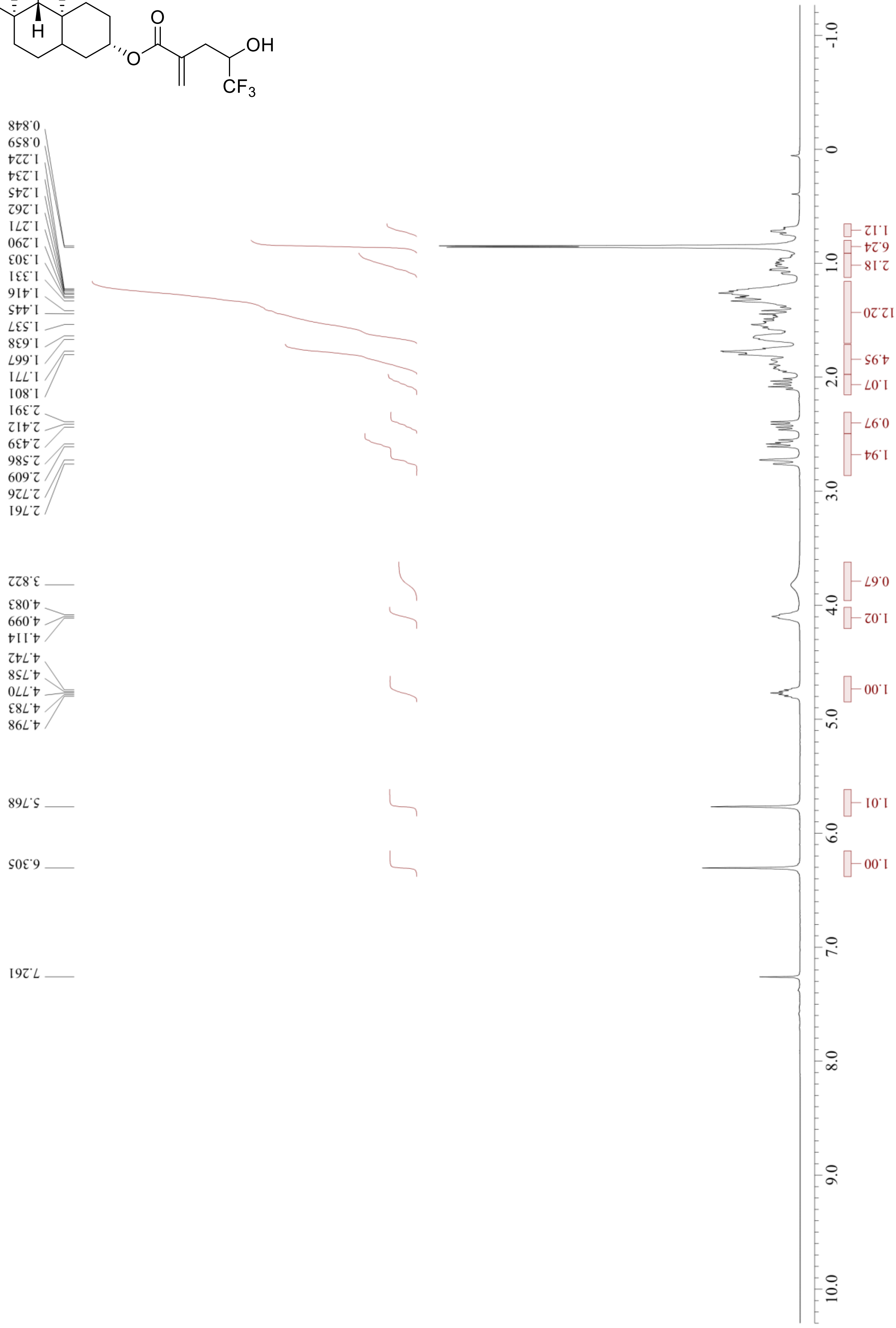

^{13}C NMR of **9aj** ($CDCl_3$, 100 MHz, 25 ºC)

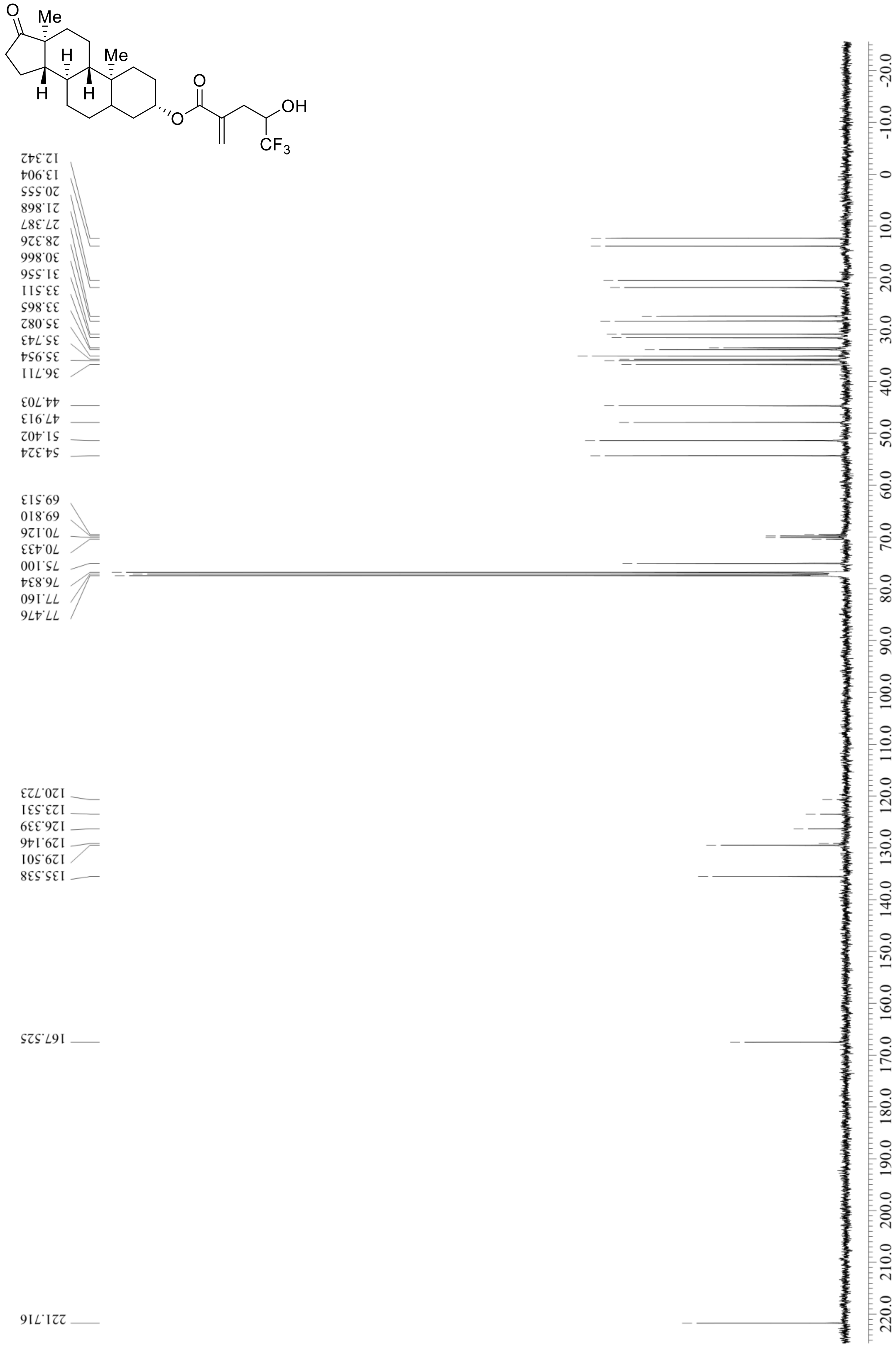

^{19}F NMR of **9aj** ($CDCl_3$, 375 MHz, 25 ºC)

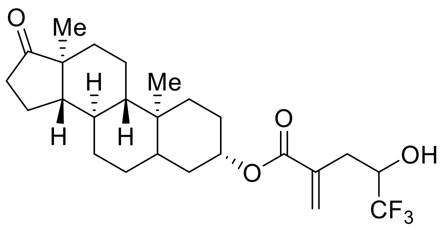

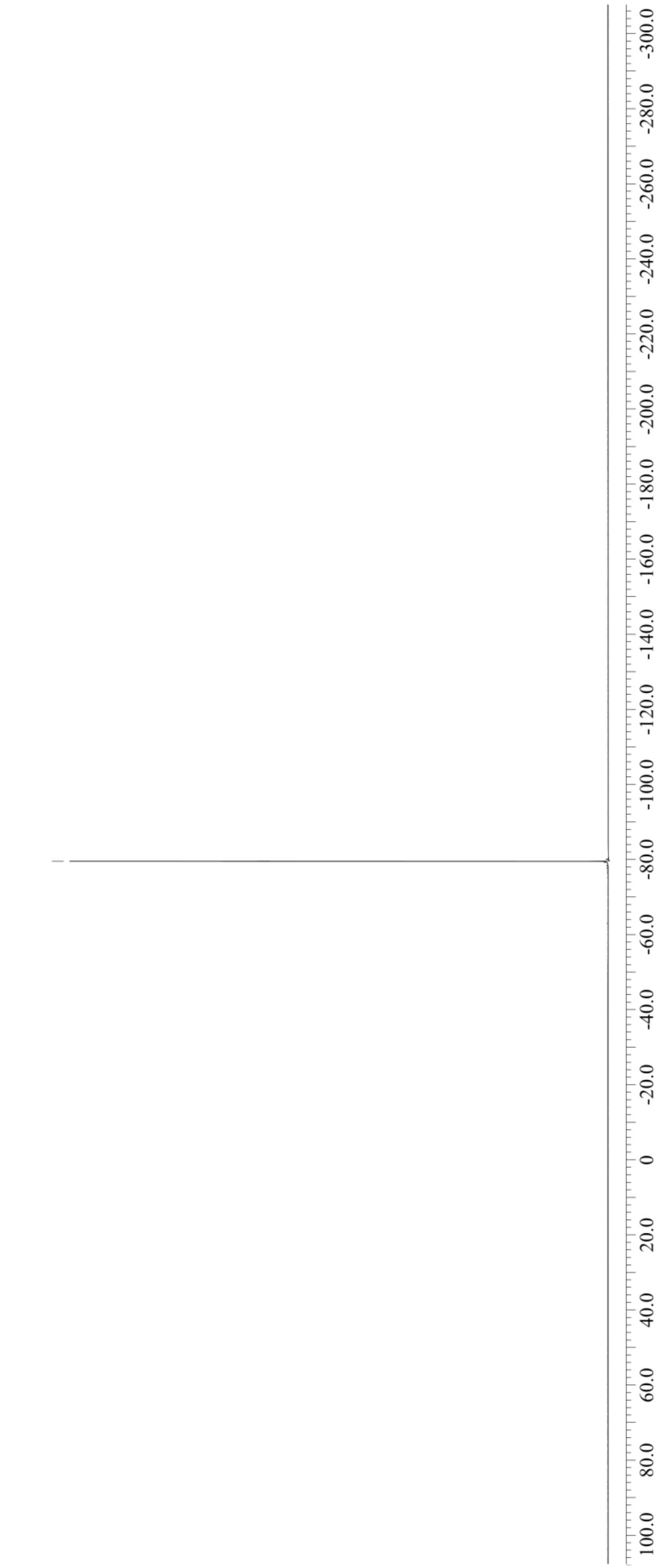

^{1}H NMR of **9ak** ($CDCl_3$, 400 MHz, 25 ºC)

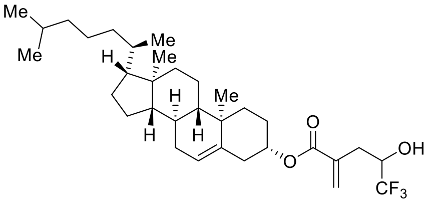

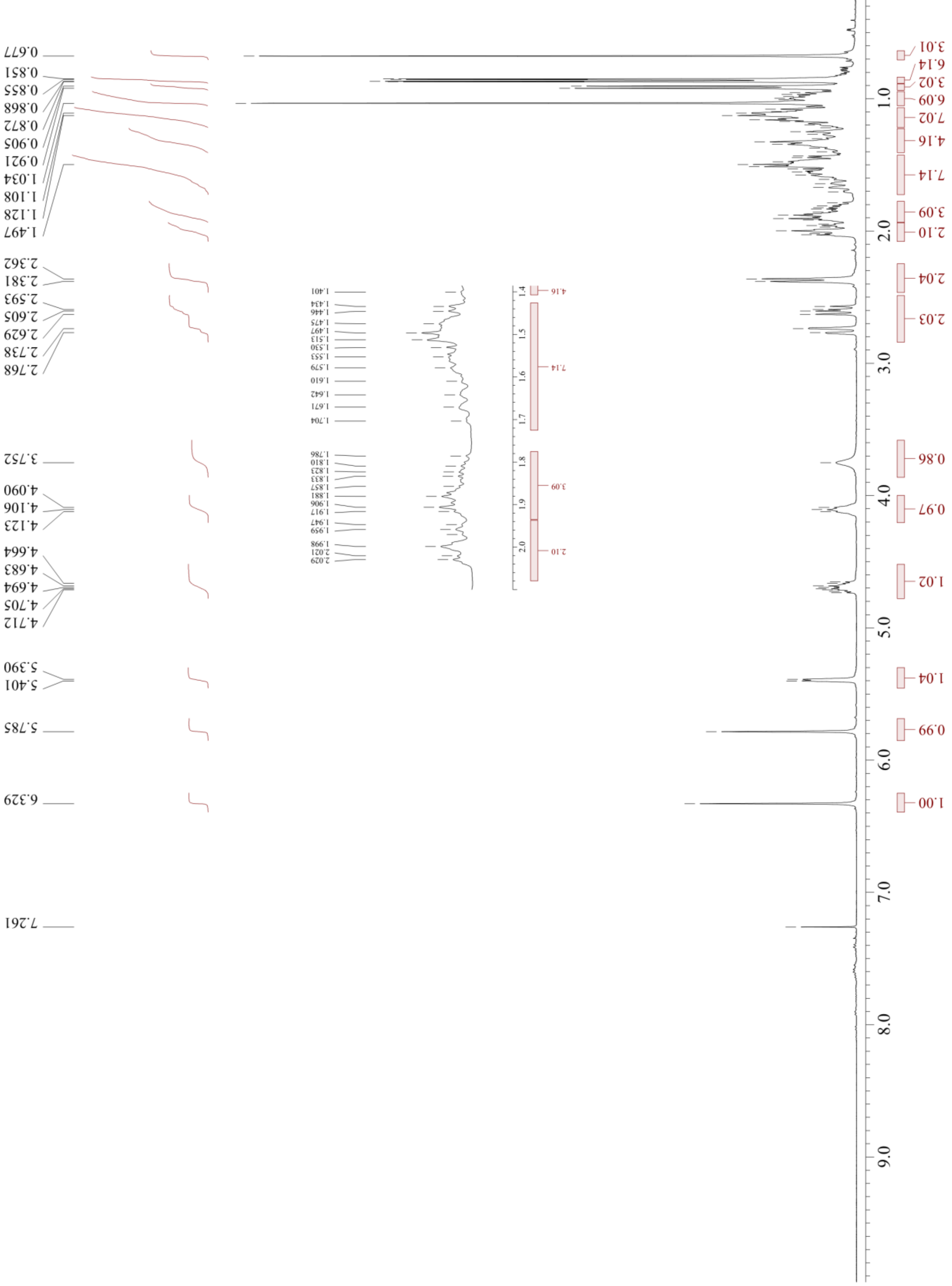

^{13}C NMR of **9ak** ($CDCl_3$, 100 MHz, 25 ºC)

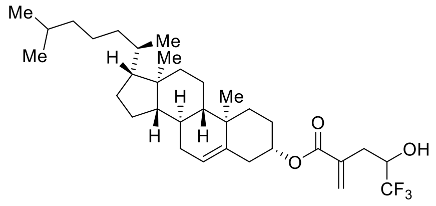

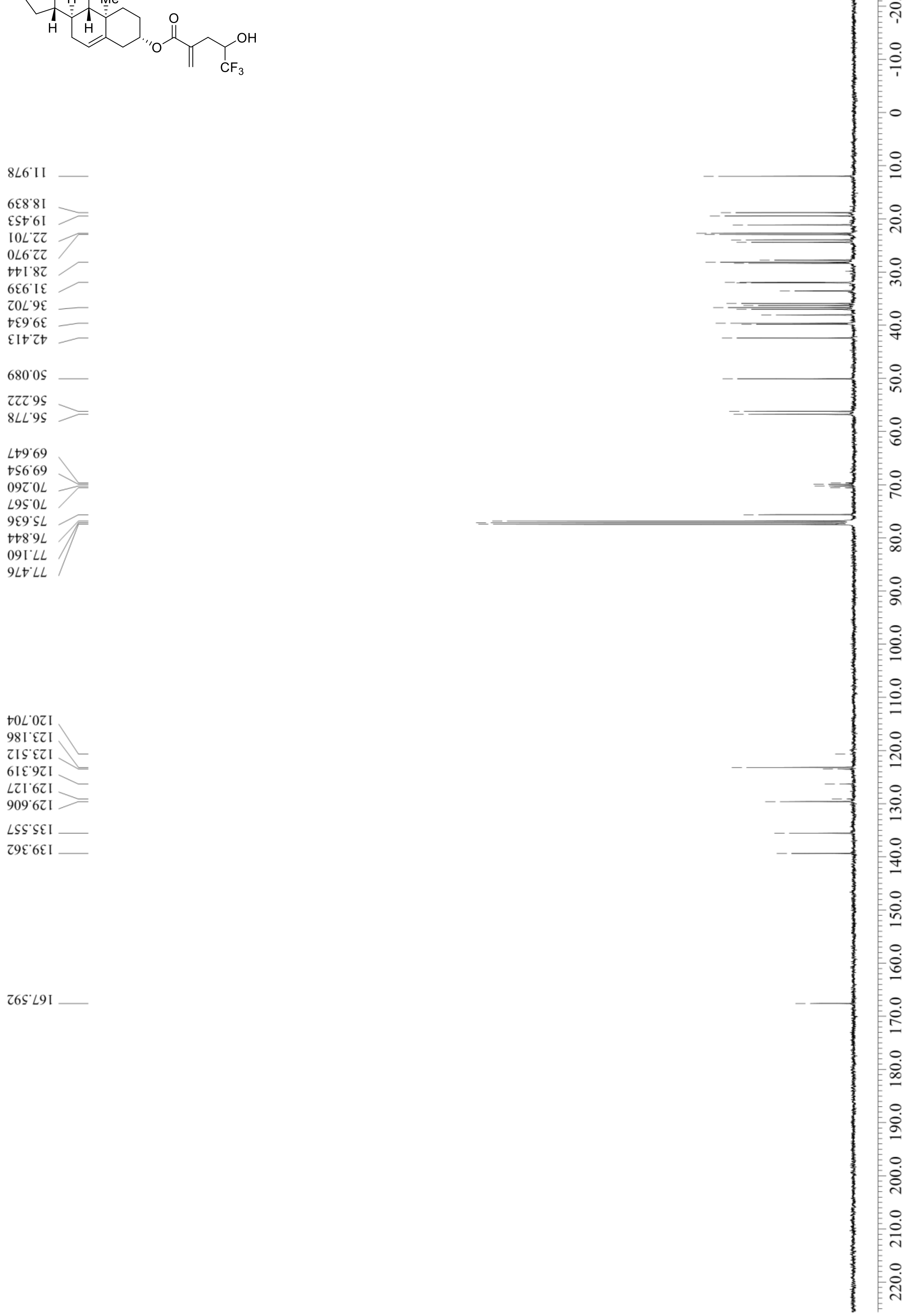

^{19}F NMR of **9ak** ($CDCl_3$, 375 MHz, 25 ºC)

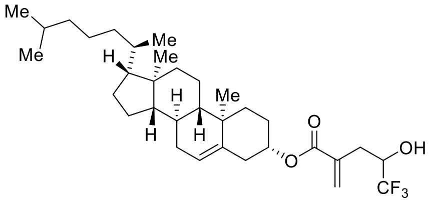

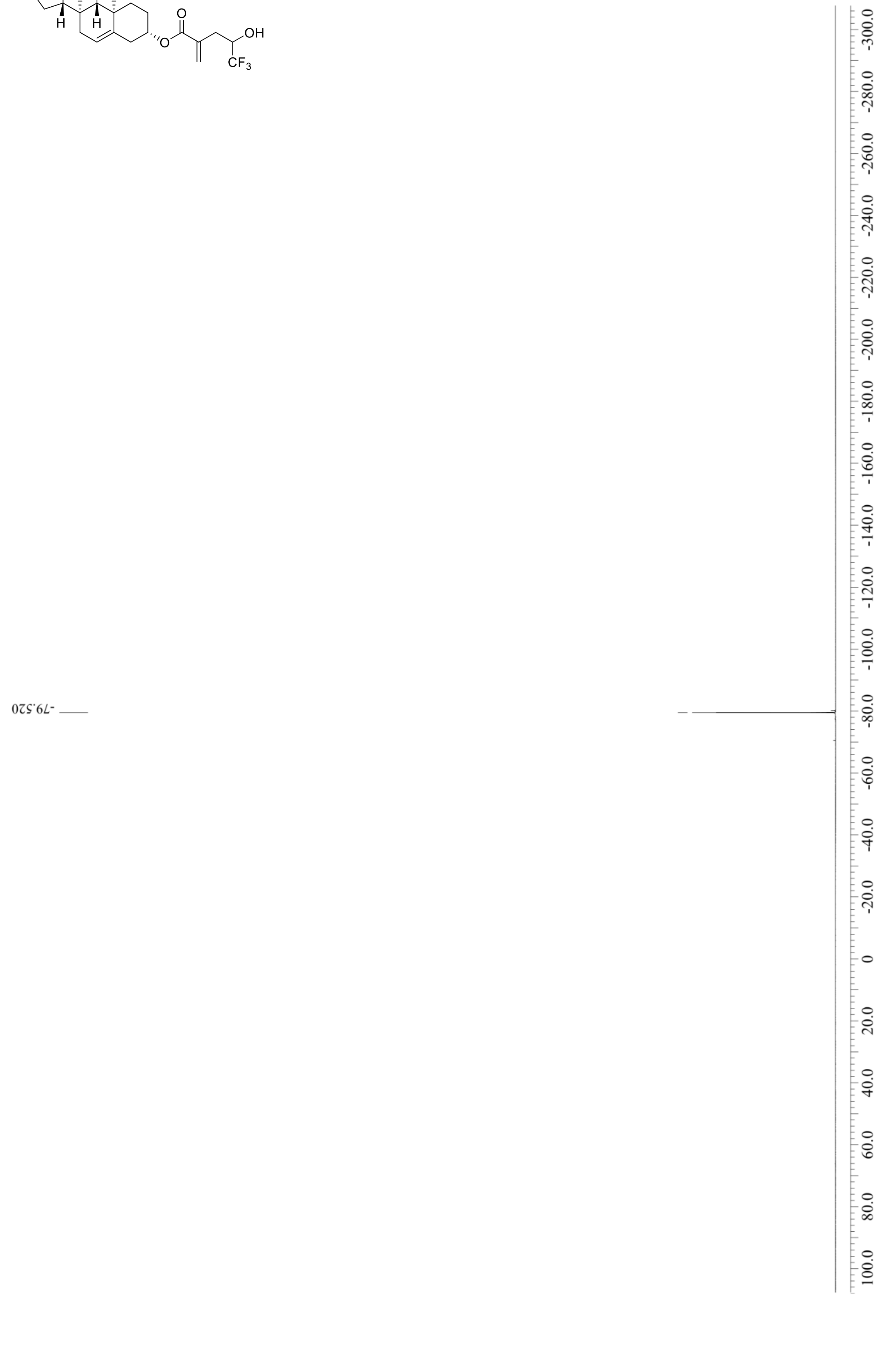

^{1}H NMR of **9al** ($CDCl_3$, 400 MHz, 25 ºC)

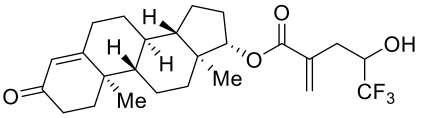

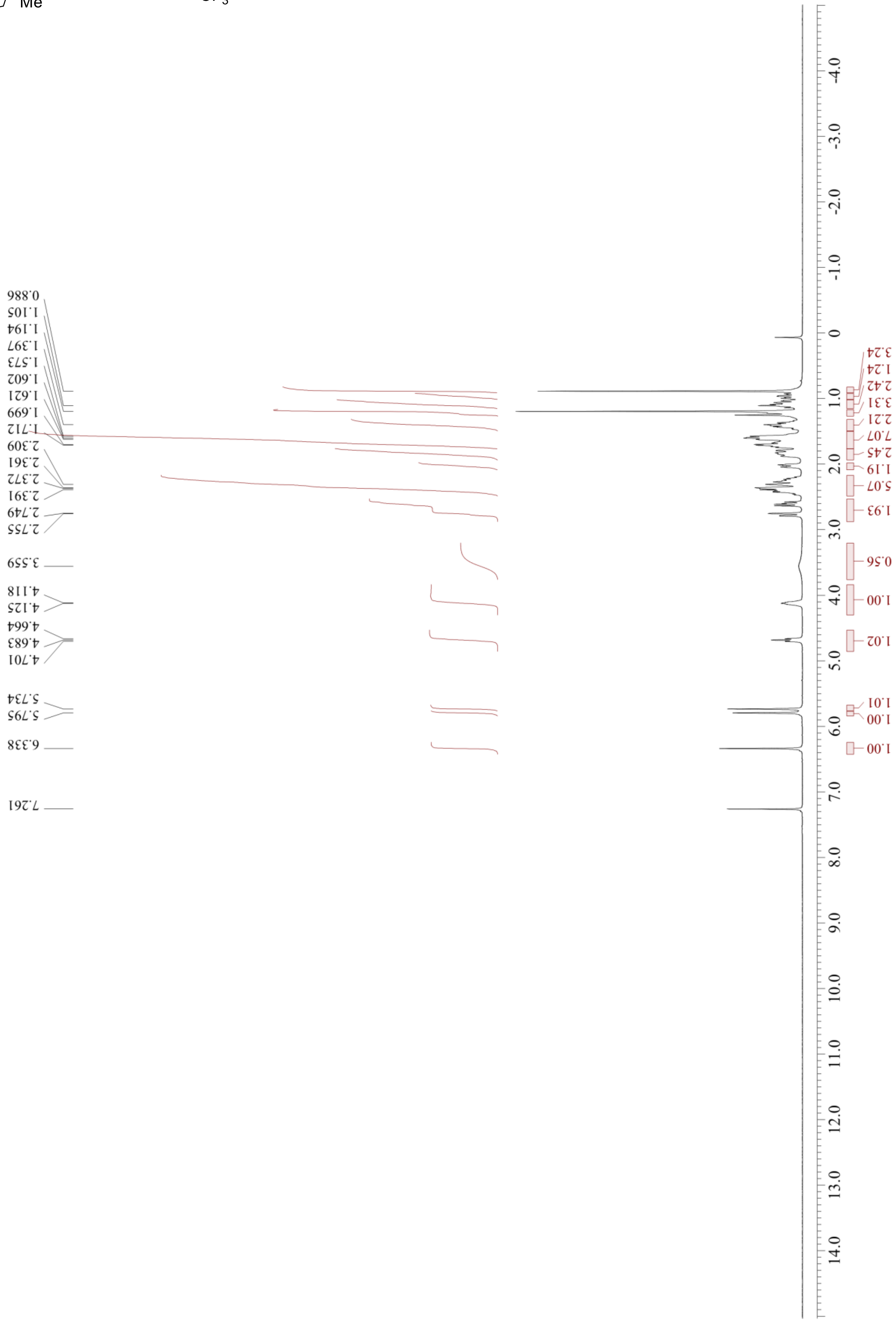

^{13}C NMR of **9al** ($CDCl_3$, 100 MHz, 25 °C)

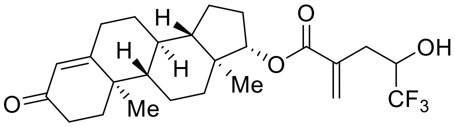

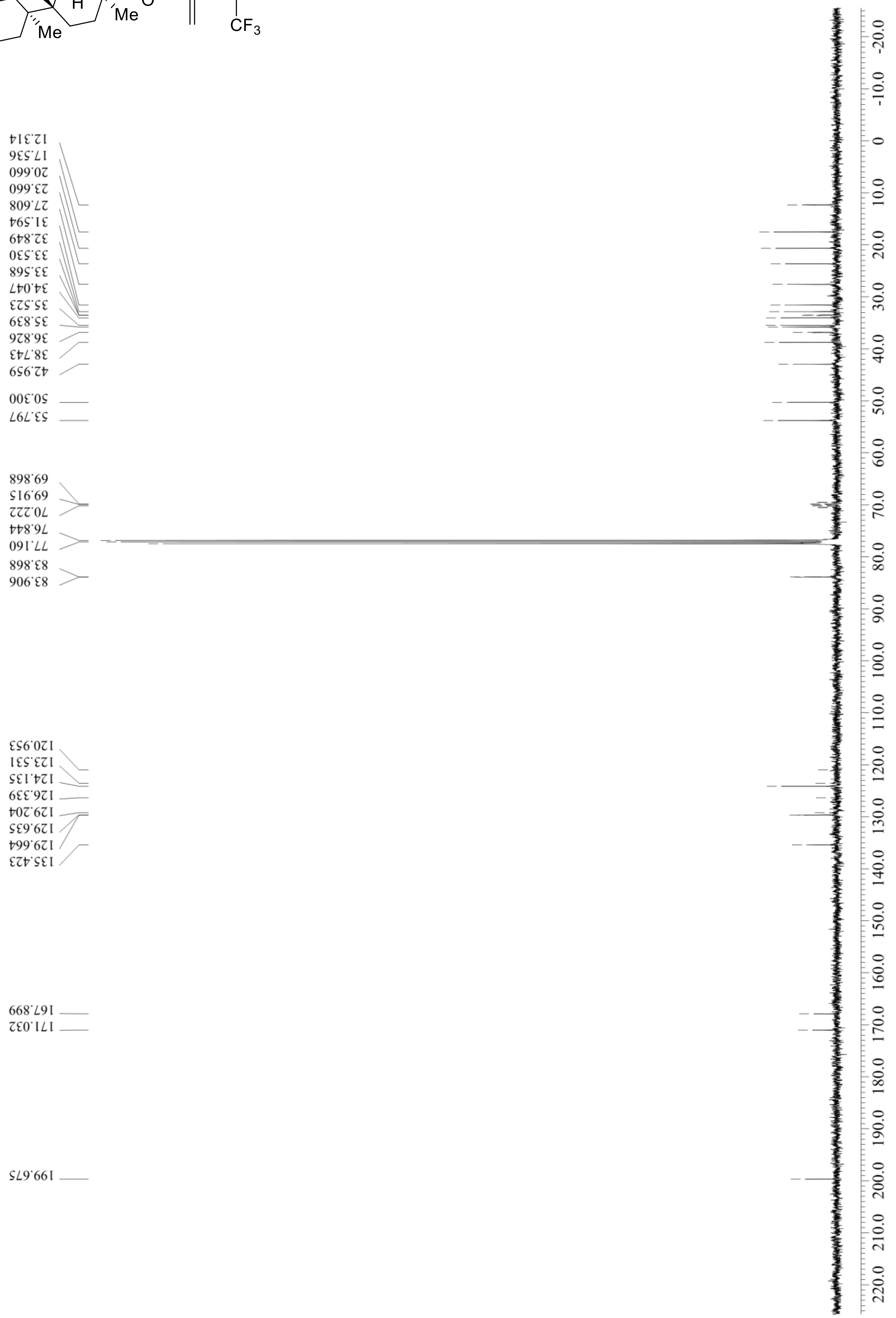

^{19}F NMR of **9al** ($CDCl_3$, 375 MHz, 25 ºC)

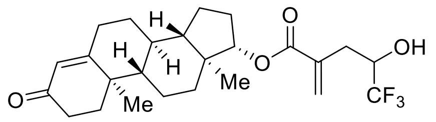

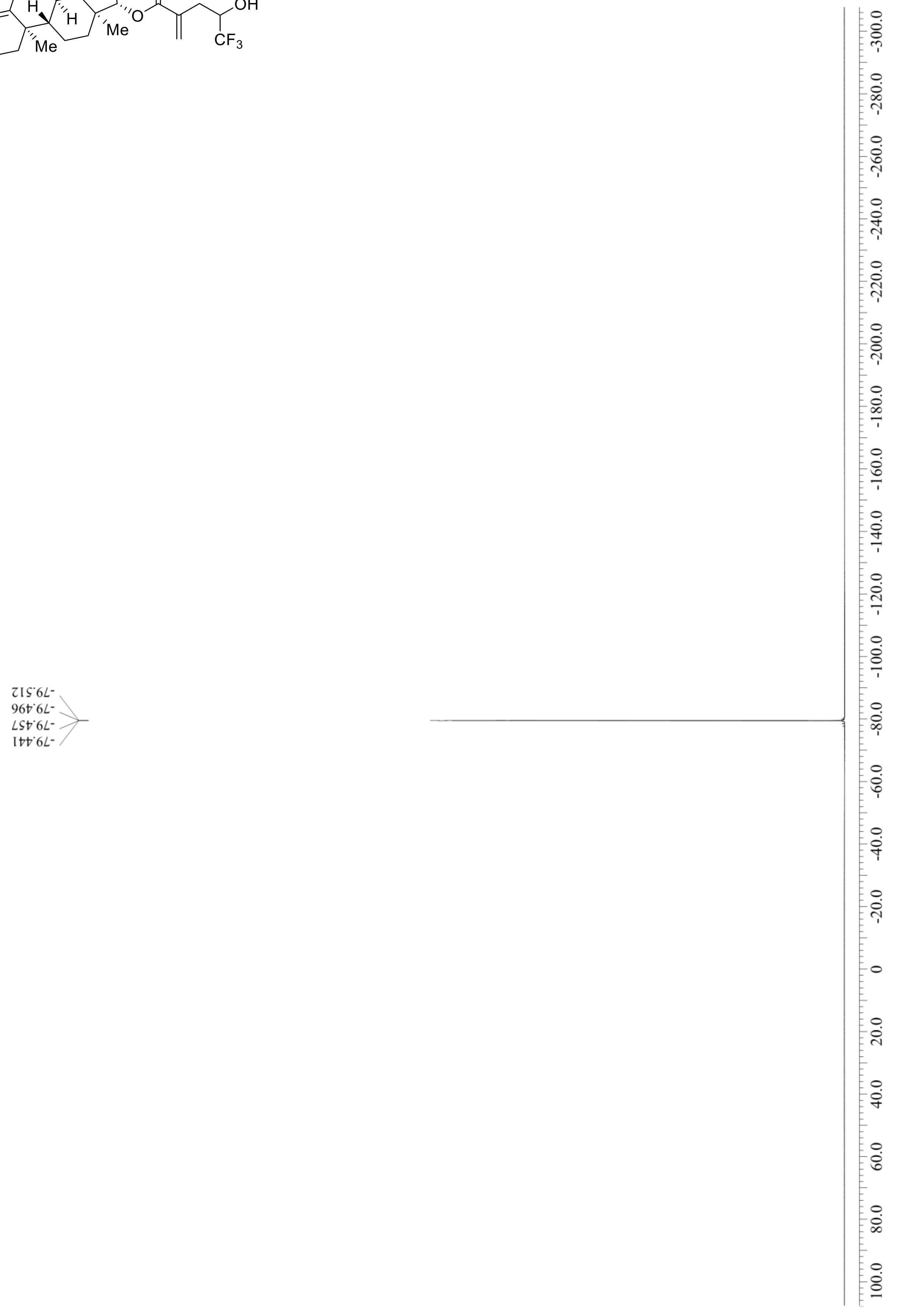

^{1}H NMR of **9am** ($CDCl_3$, 400 MHz, 25 ºC)

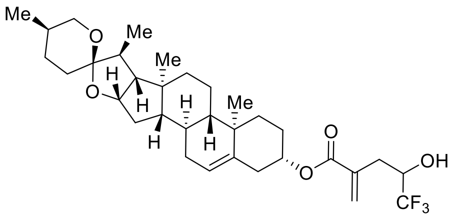

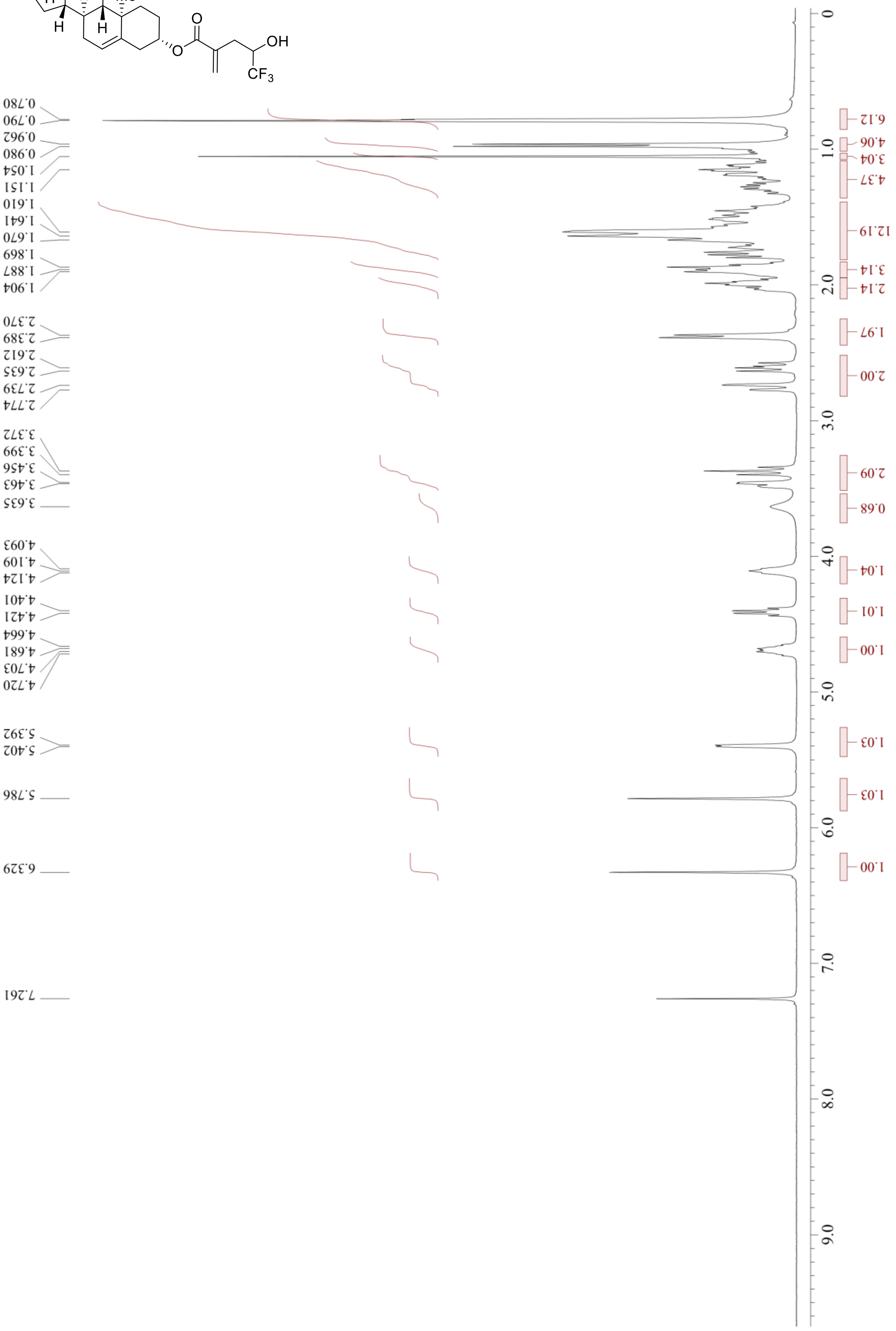

^{13}C NMR of **9am** ($CDCl_3$, 100 MHz, 25 ºC)

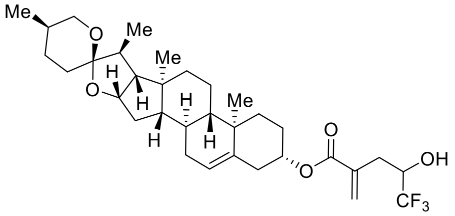

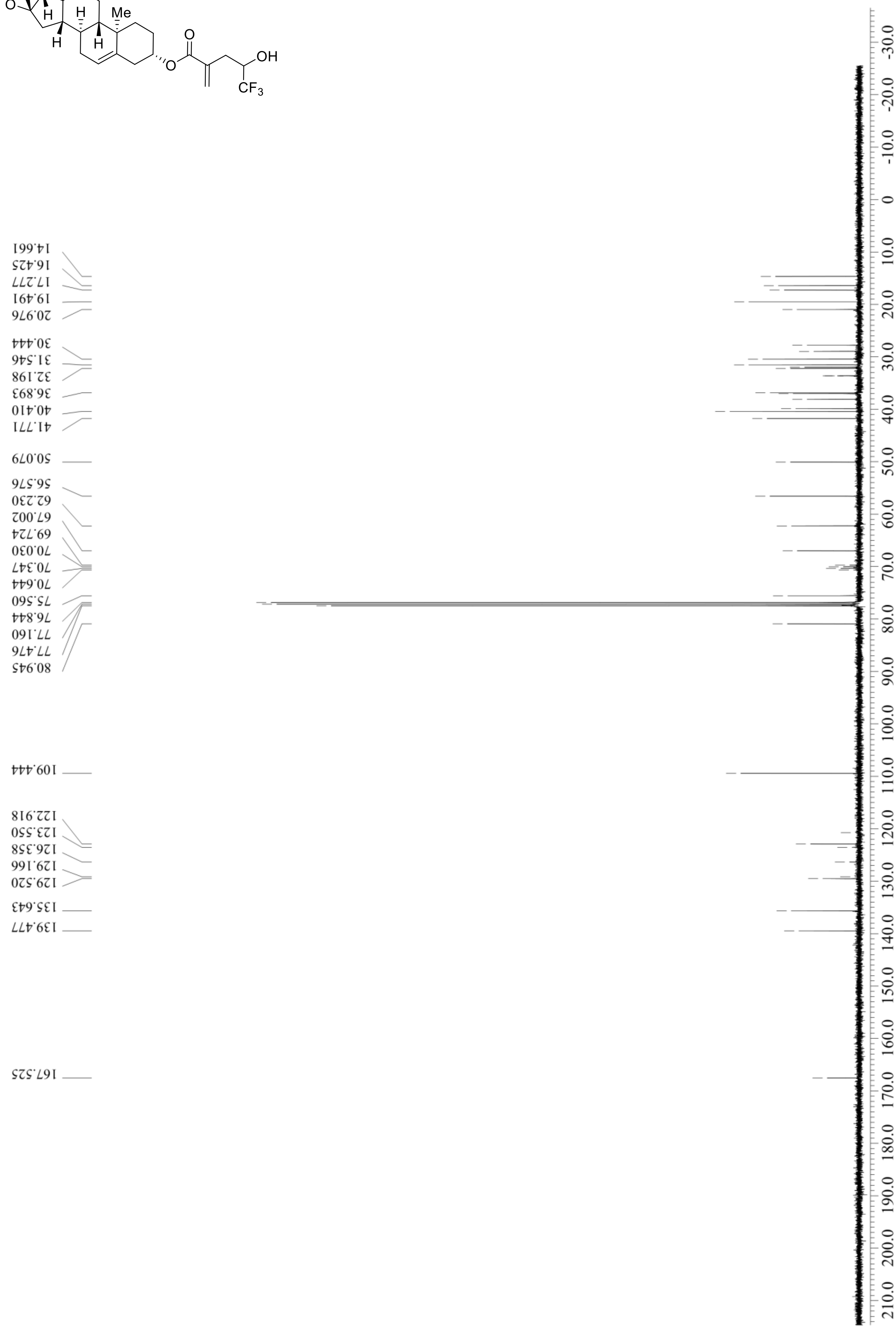

^{19}F NMR of **9am** ($CDCl_3$, 375 MHz, 25 ºC)

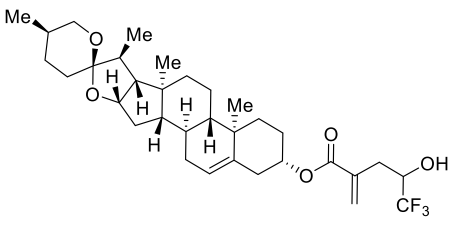

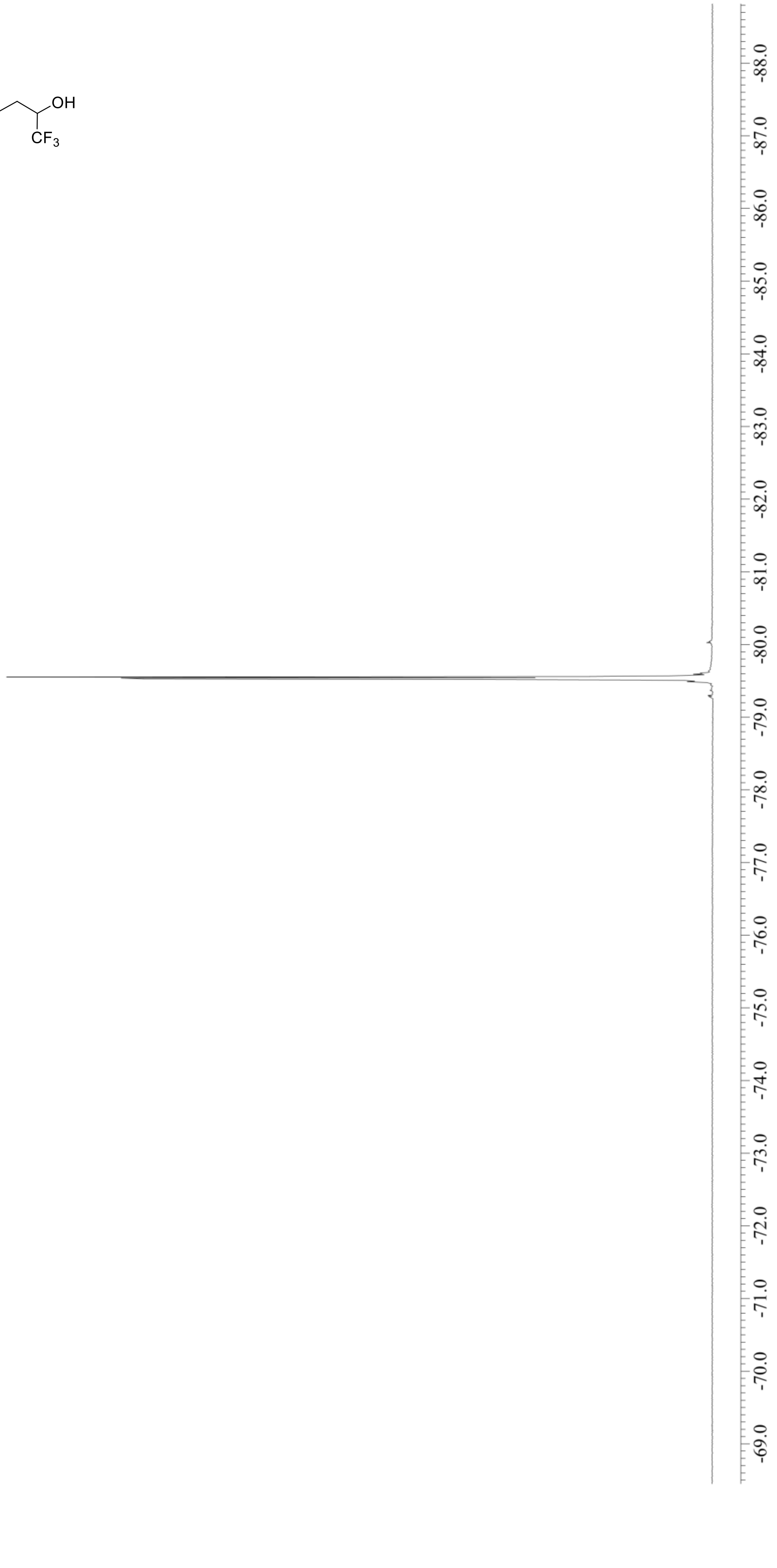

^{1}H NMR of **9an** ($CDCl_3$, 400 MHz, 25 °C)

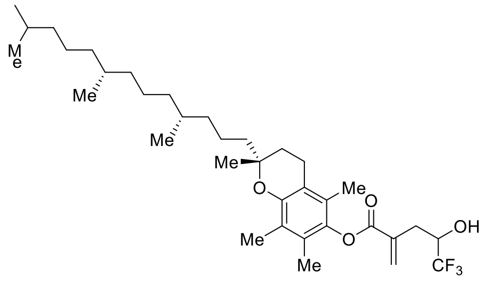

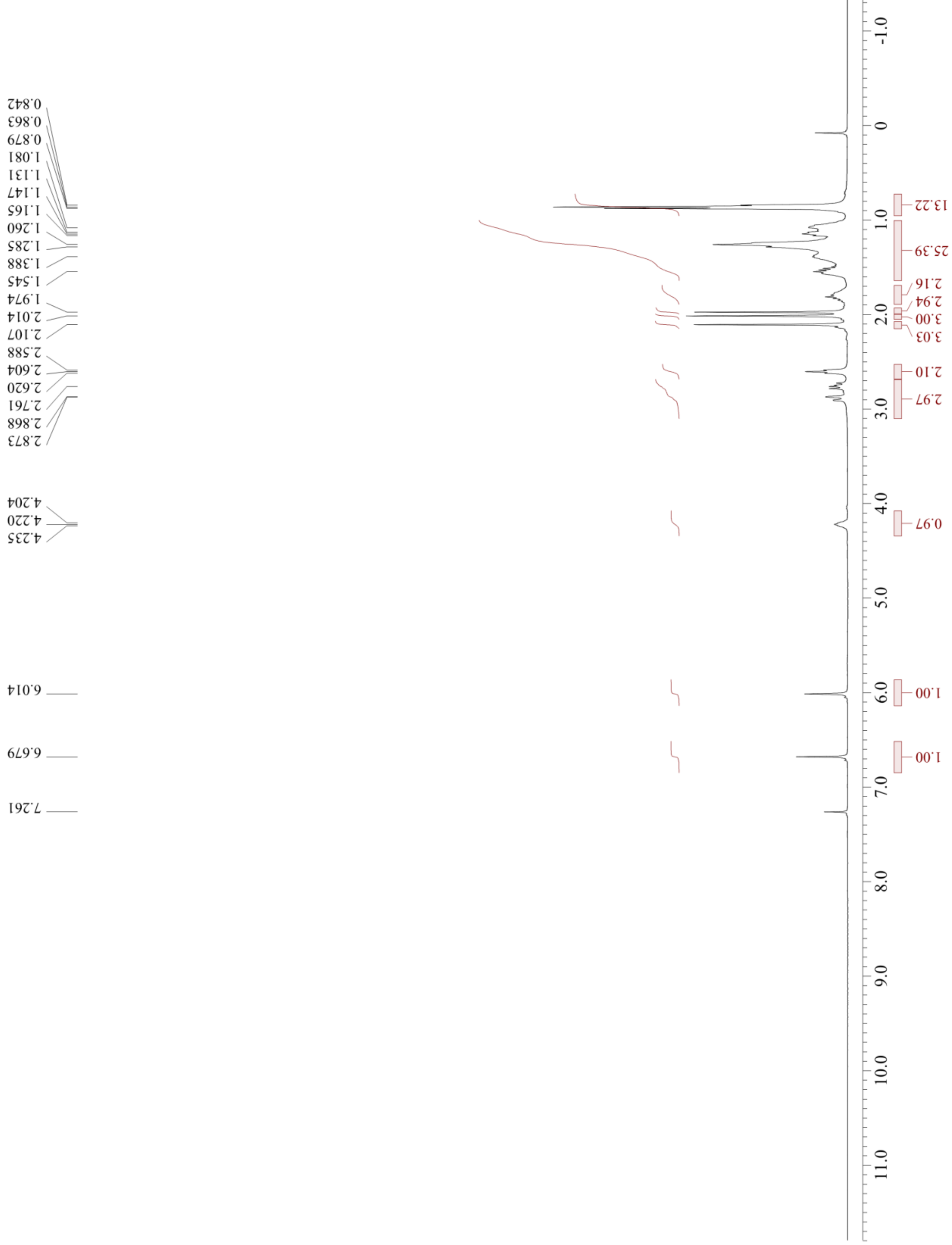

^{13}C NMR of **9an** ($CDCl_3$, 100 MHz, 25 ºC)

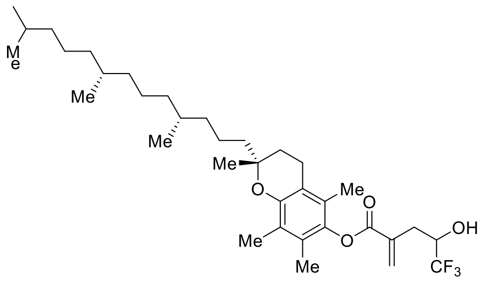

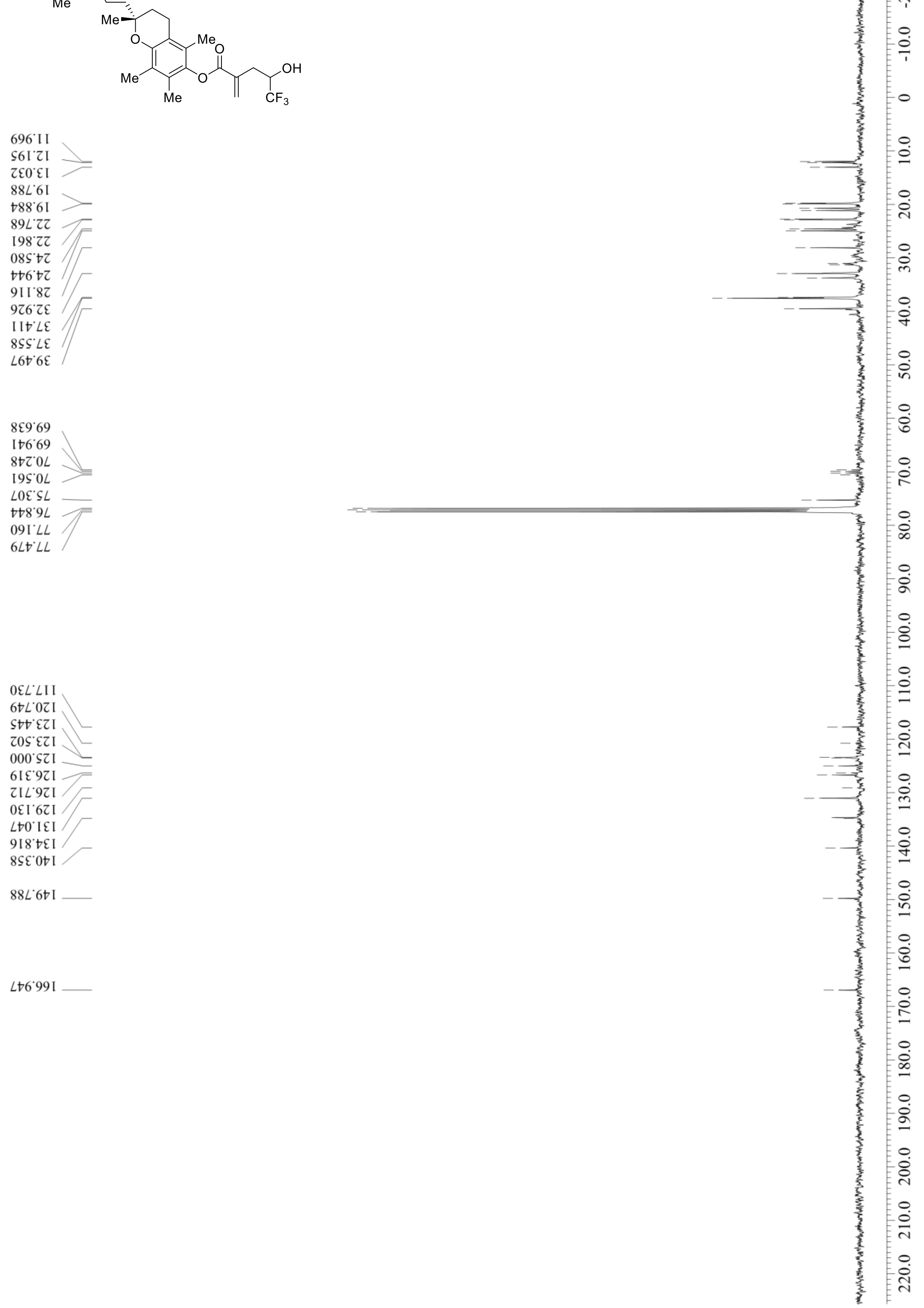

^{19}F NMR of **9an** ($CDCl_3$, 375 MHz, 25 ºC)

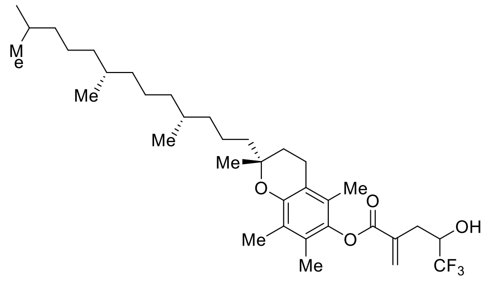

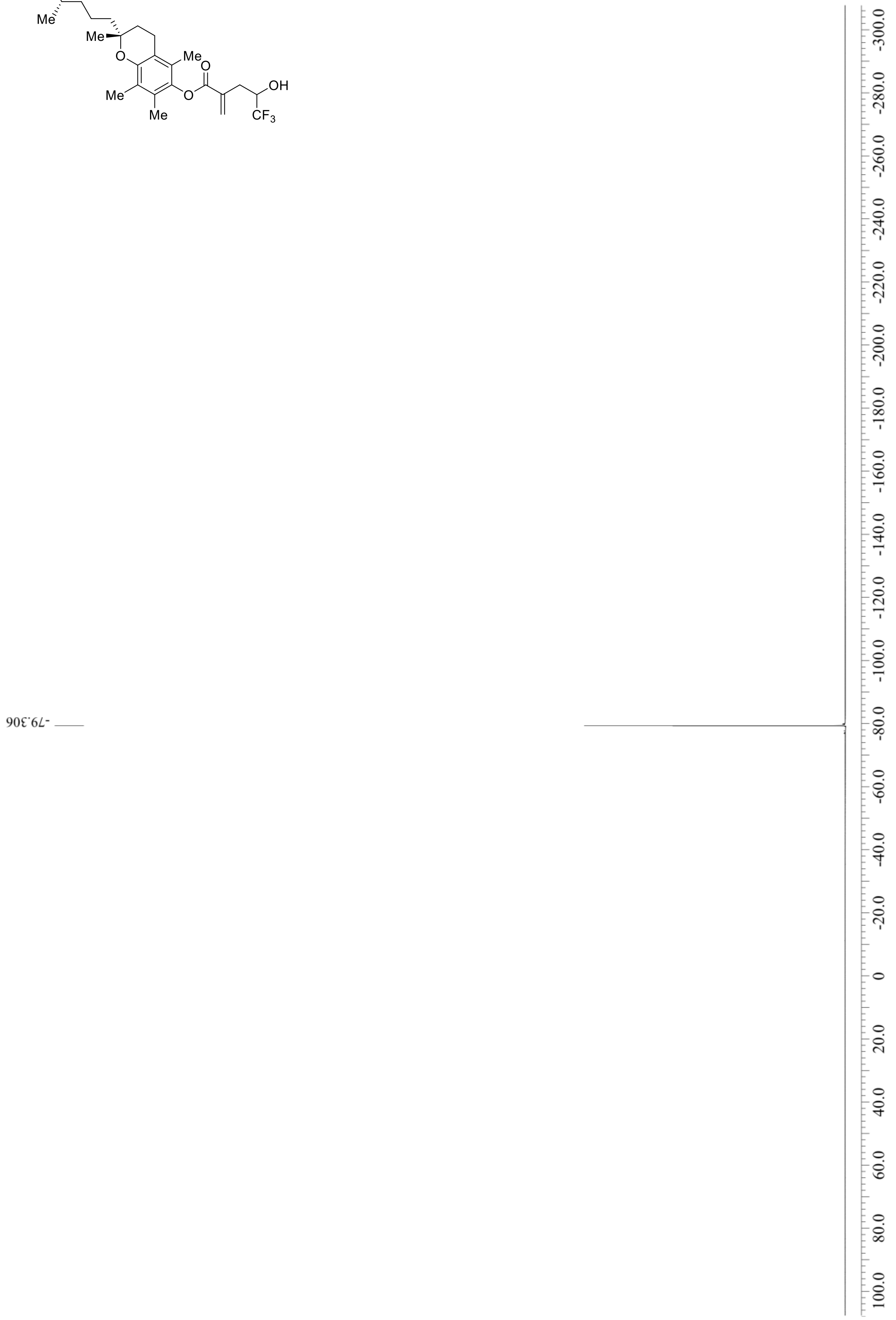

^{1}H NMR of **9ao** ($CDCl_3$, 400 MHz, 25 ºC)

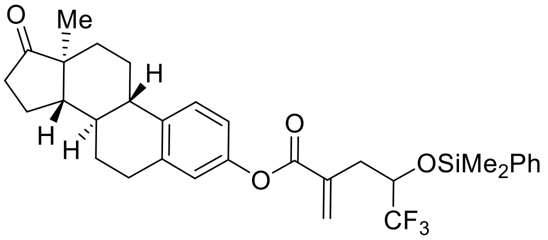

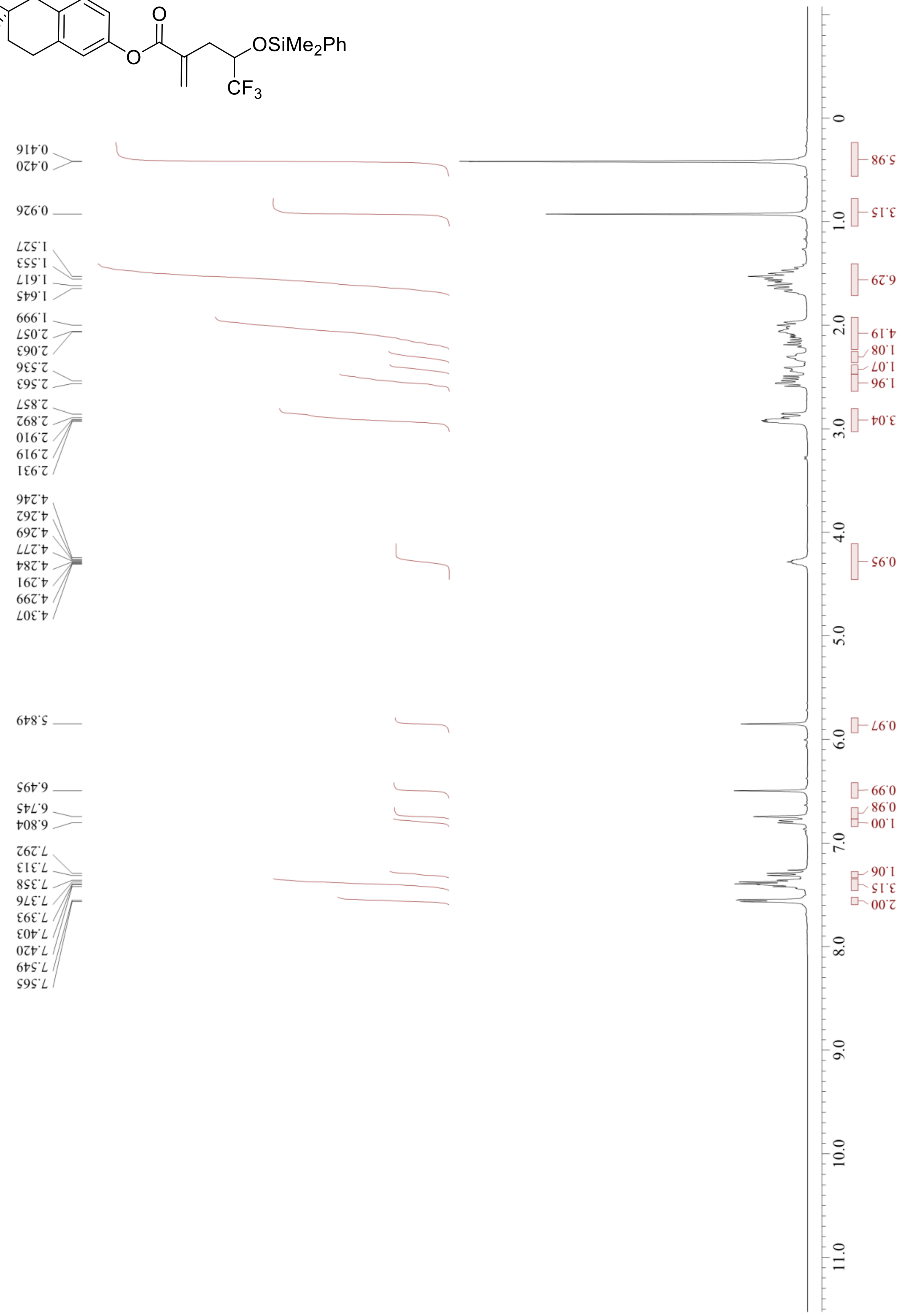

^{13}C NMR of **9ao** ($CDCl_3$, 100 MHz, 25 ºC)

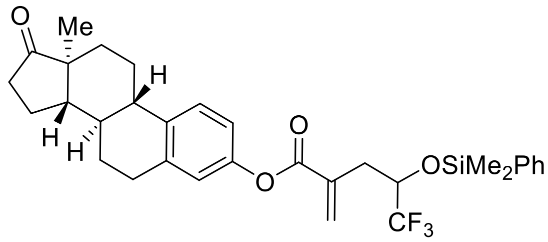

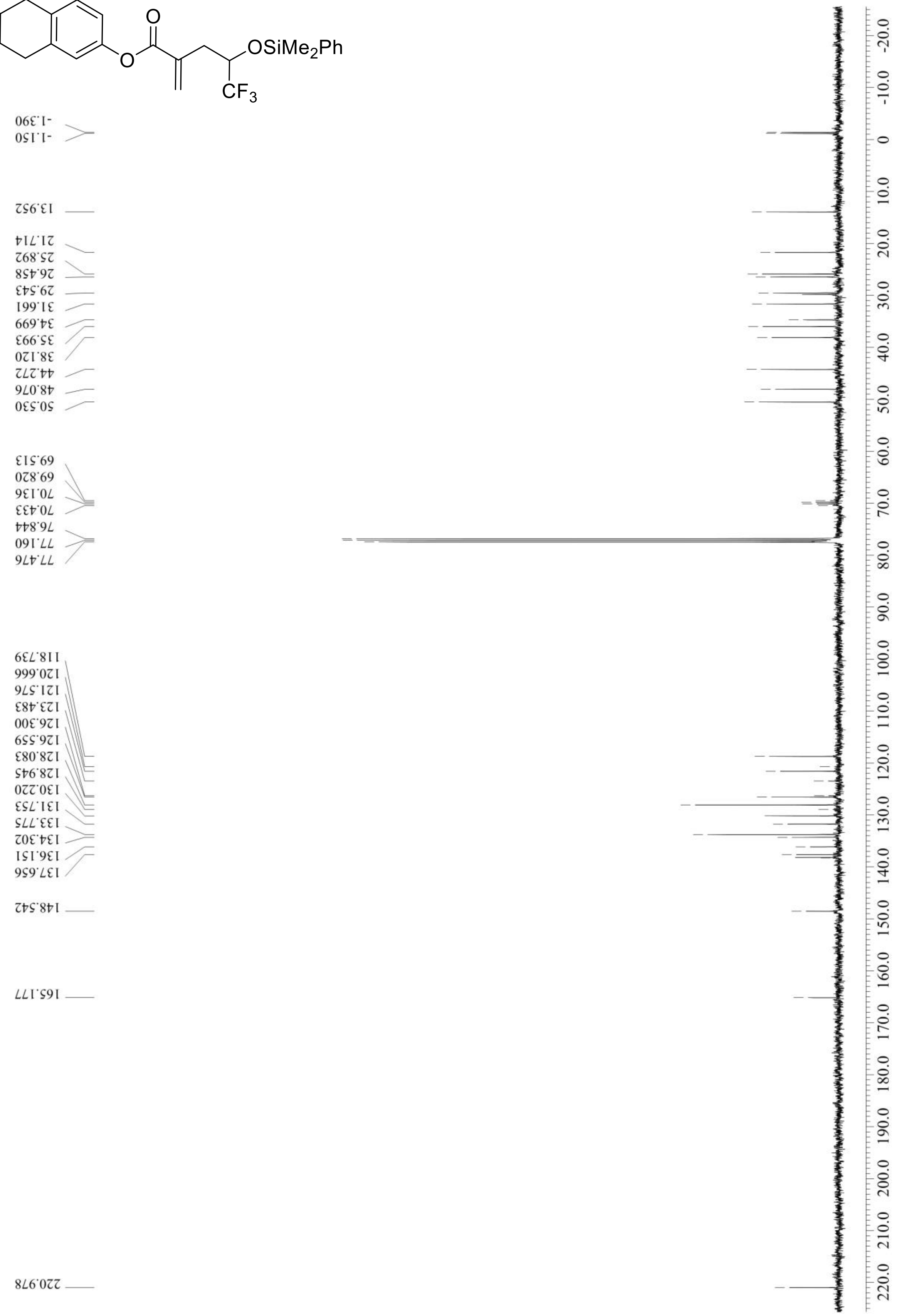

^{19}F NMR of **9ao** ($CDCl_3$, 375 MHz, 25 ºC)

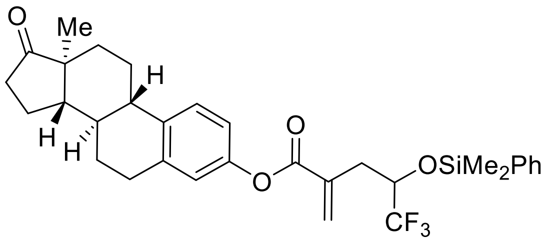

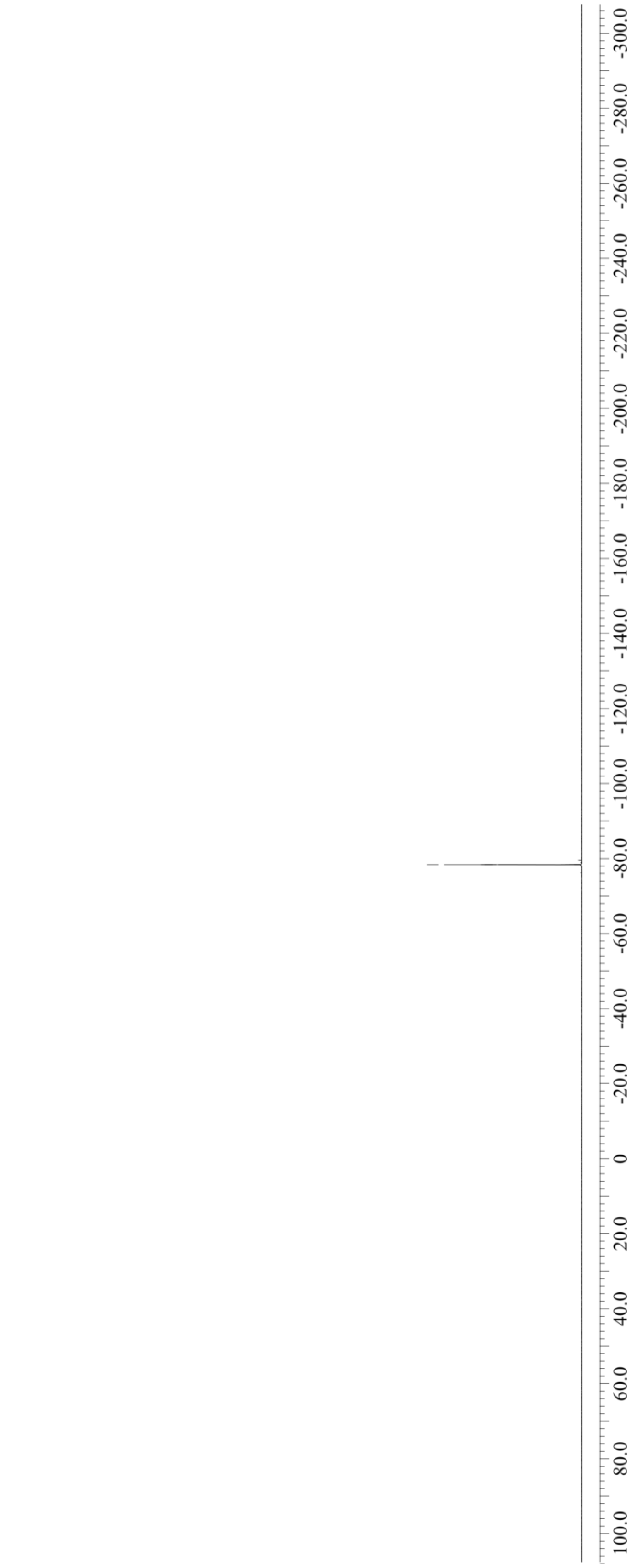

^{1}H NMR of **9ap** ($CDCl_3$, 400 MHz, 25 ºC)

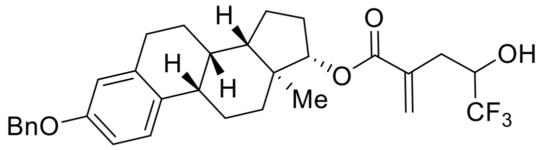

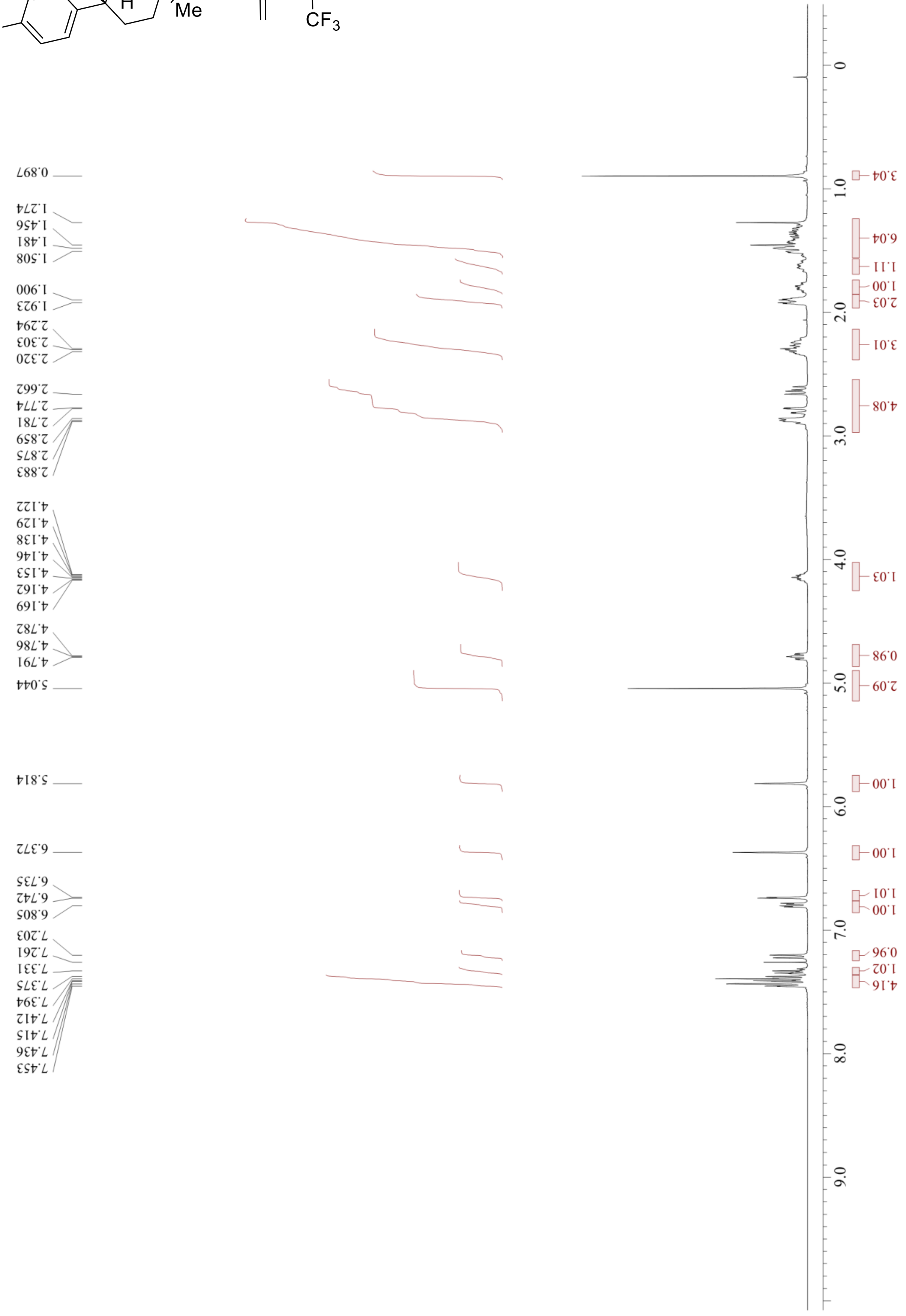

^{13}C NMR of **9ap** ($CDCl_3$, 100 MHz, 25 ºC)

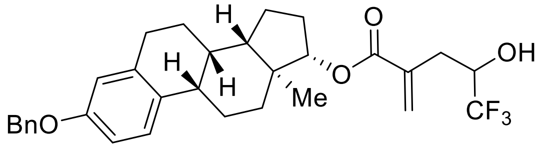

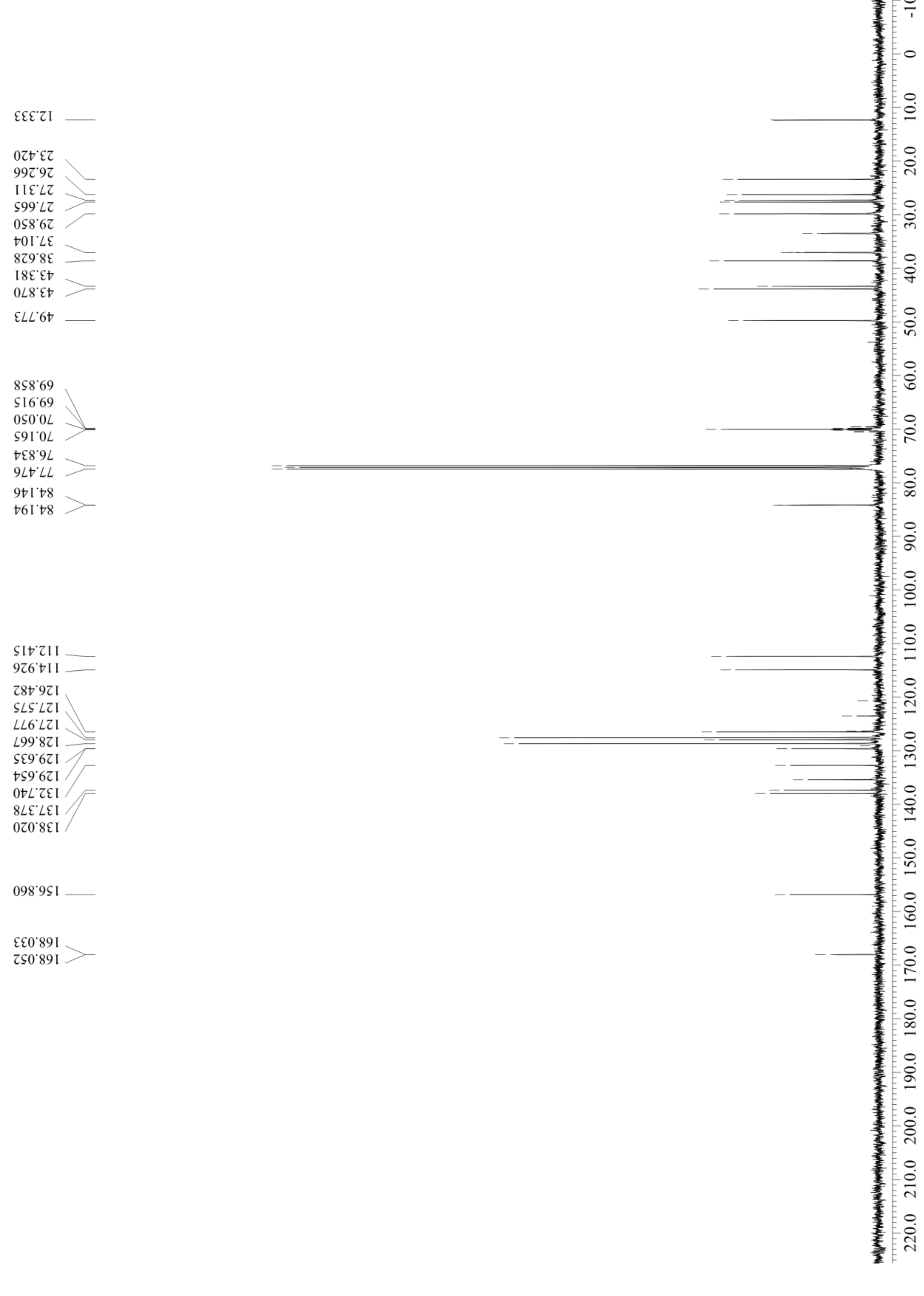

^{19}F NMR of **9ap** ($CDCl_3$, 375 MHz, 25 ºC)

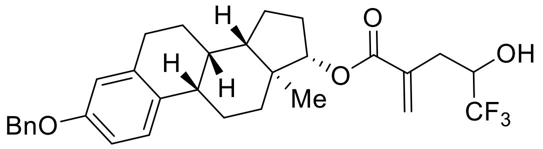

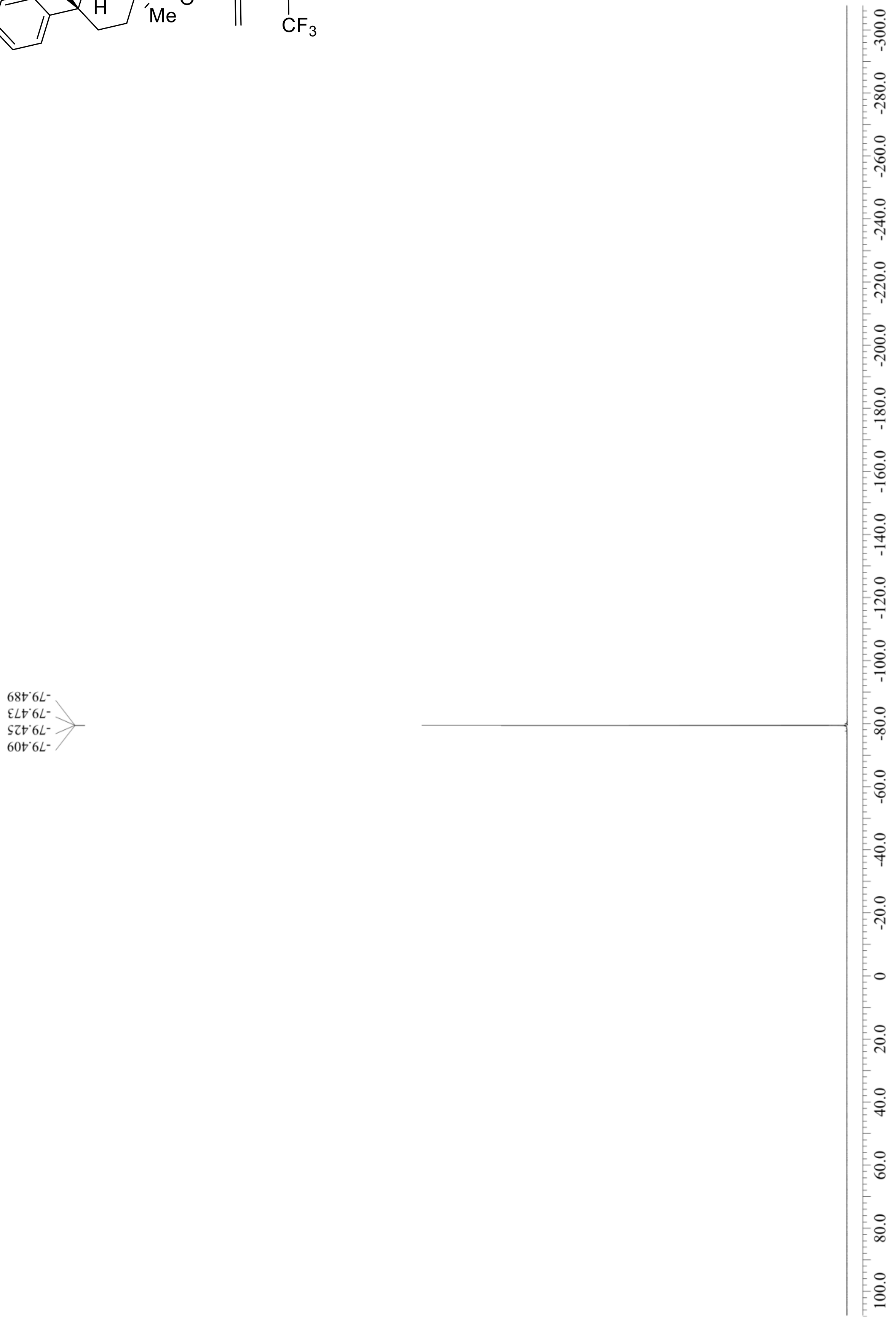

^{1}H NMR of **11a** ($CDCl_3$, 400 MHz, 25 ºC)

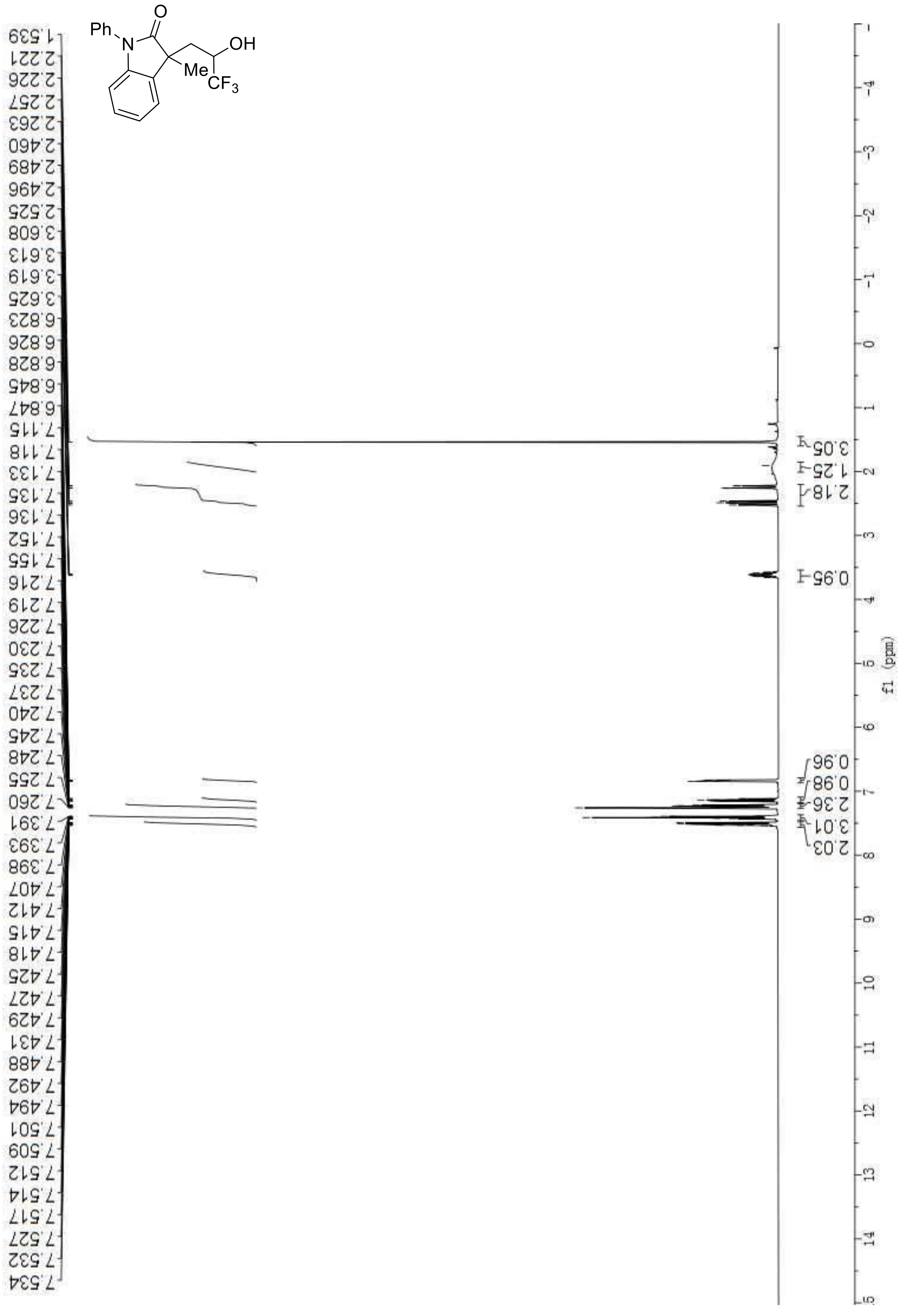

^{13}C NMR of **11a** ($CDCl_3$, 150 MHz, 25 ºC)

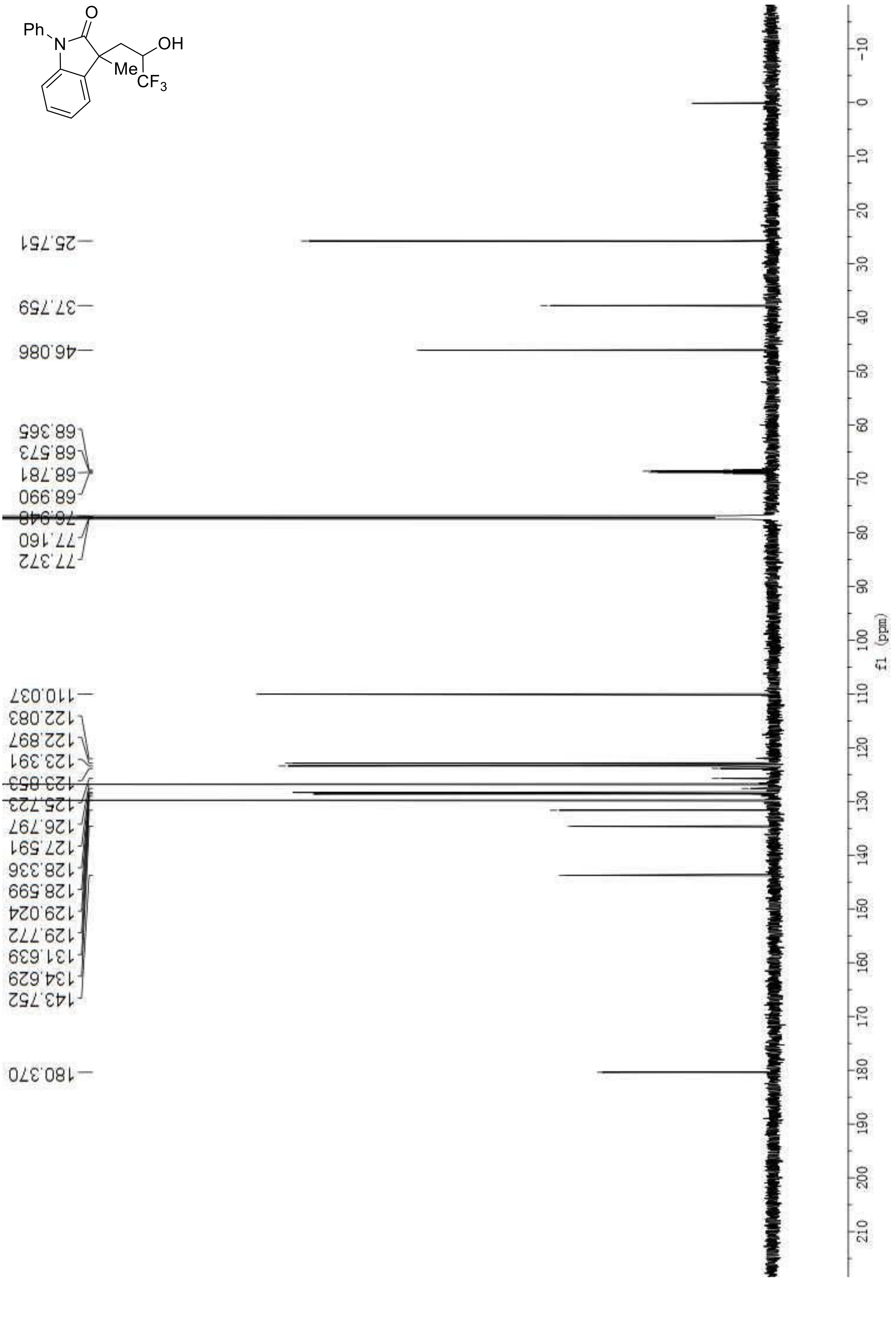

^{19}F NMR of **11a** ($CDCl_3$, 375 MHz, 25 ºC)

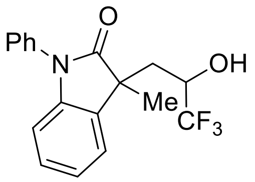

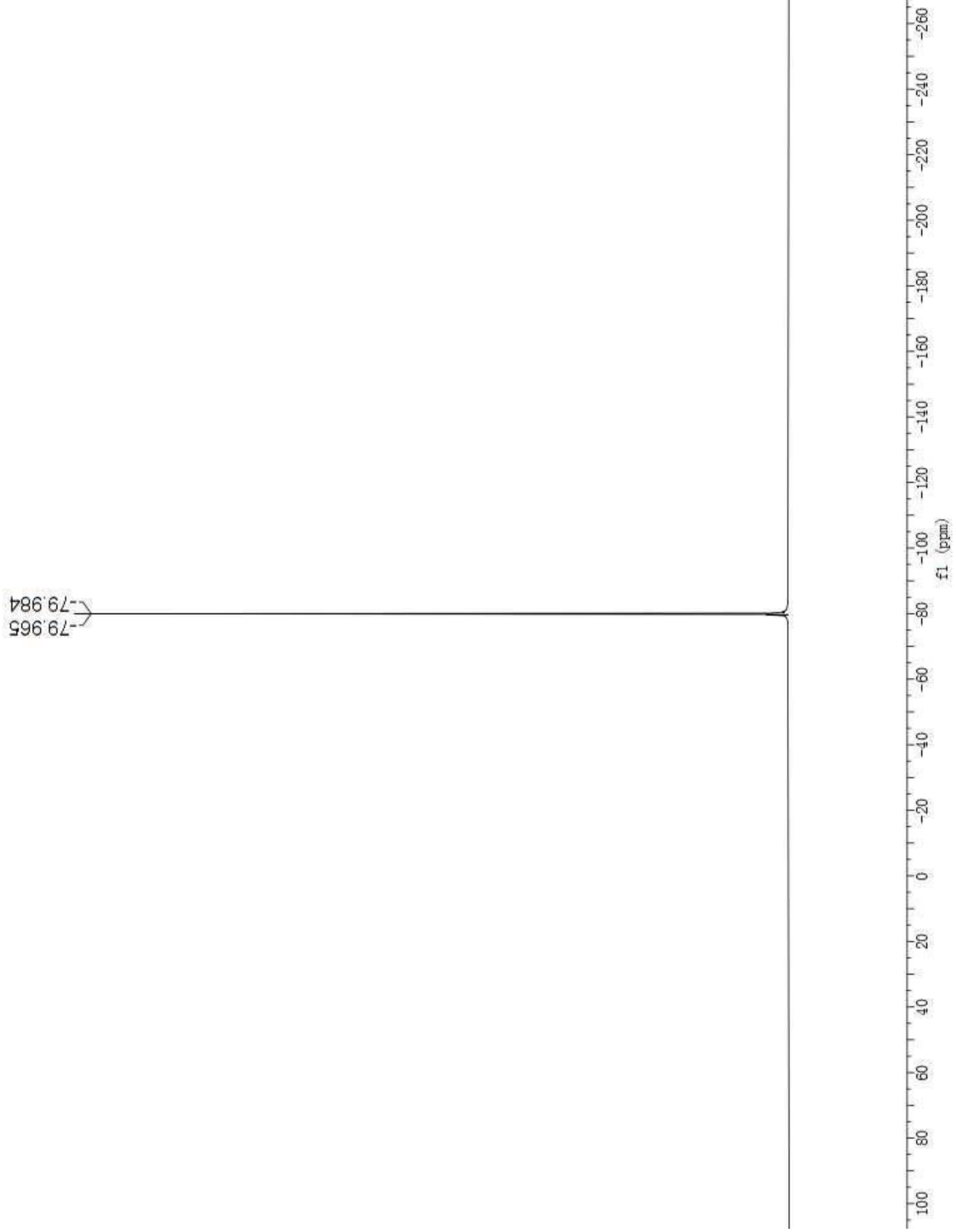

^{1}H NMR of **11b** ($CDCl_3$, 400 MHz, 25 ºC)

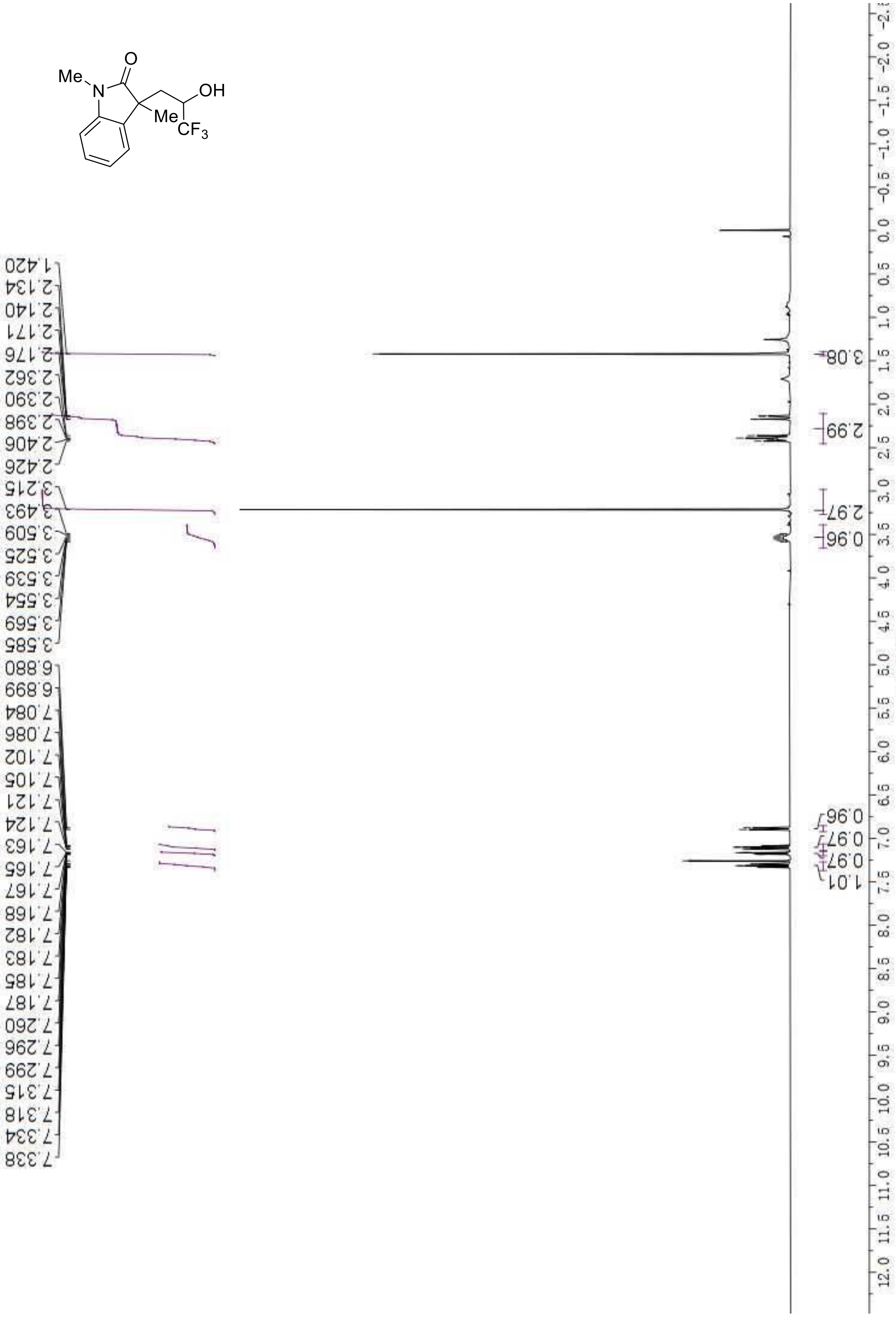

^{13}C NMR of **11b** ($CDCl_3$, 150 MHz, 25 ºC)

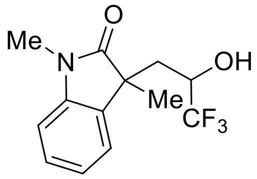

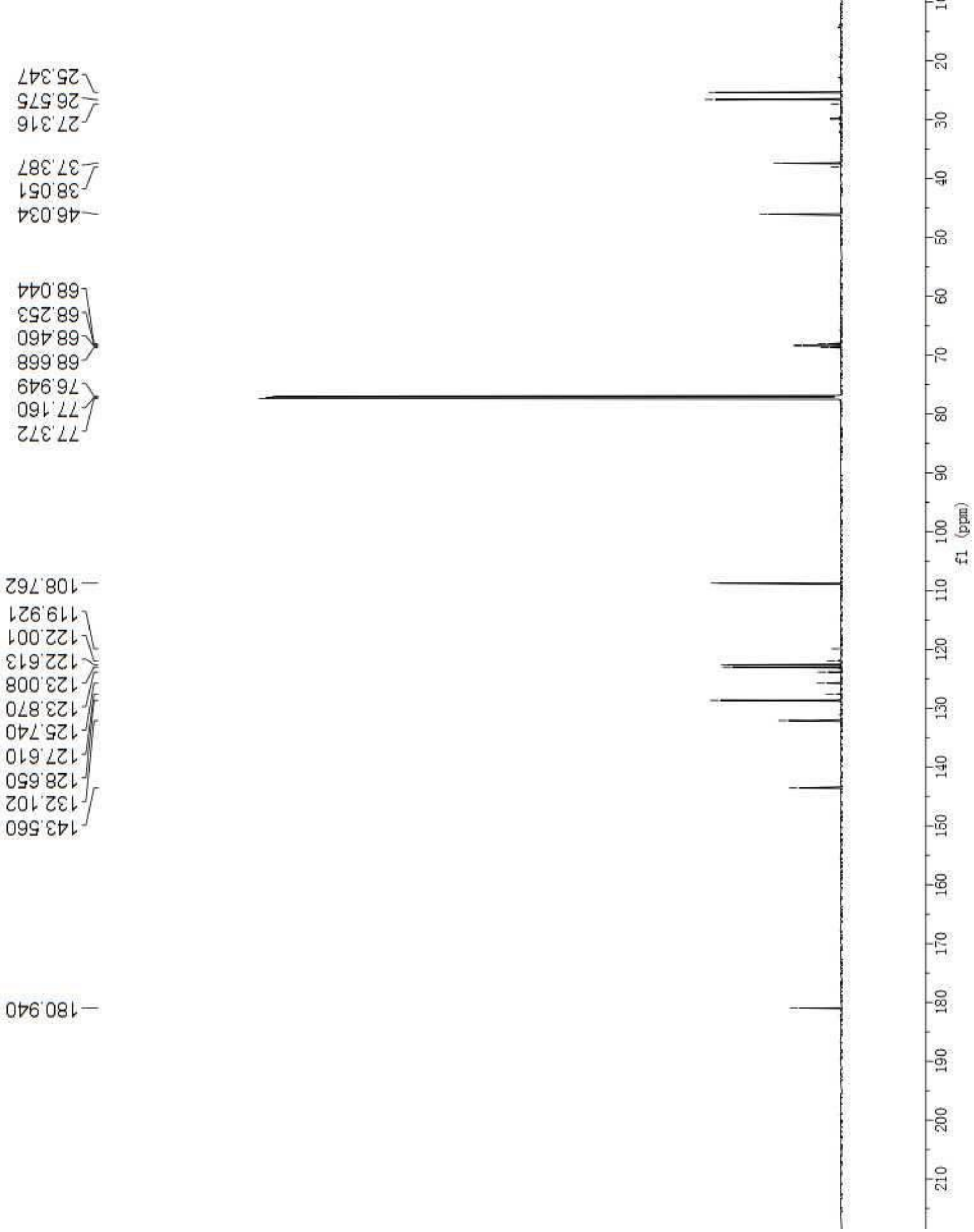

^{19}F NMR of **11b** ($CDCl_3$, 375 MHz, 25 ºC)

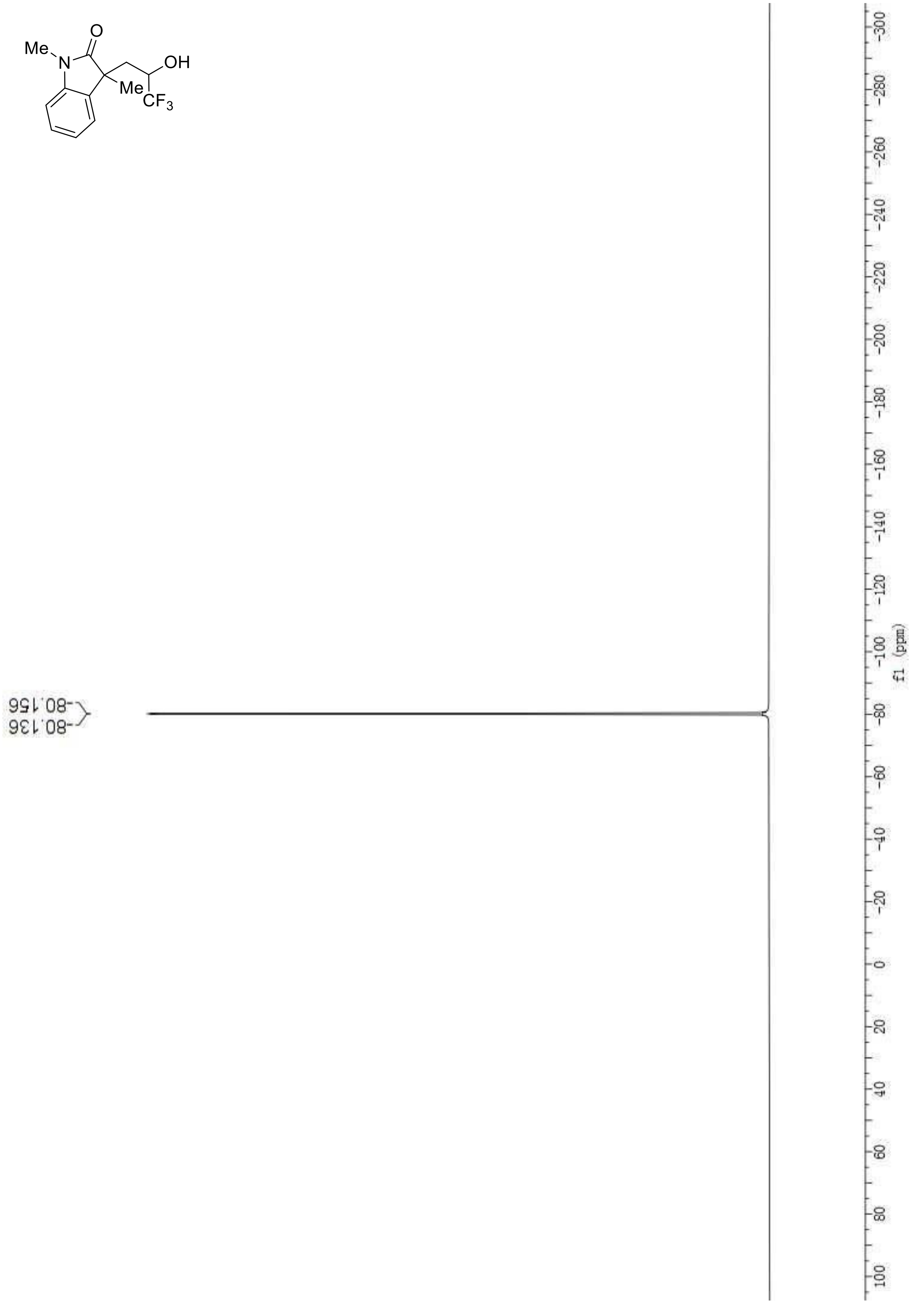

^{1}H NMR of **11c** ($CDCl_3$, 400 MHz, 25 ºC)

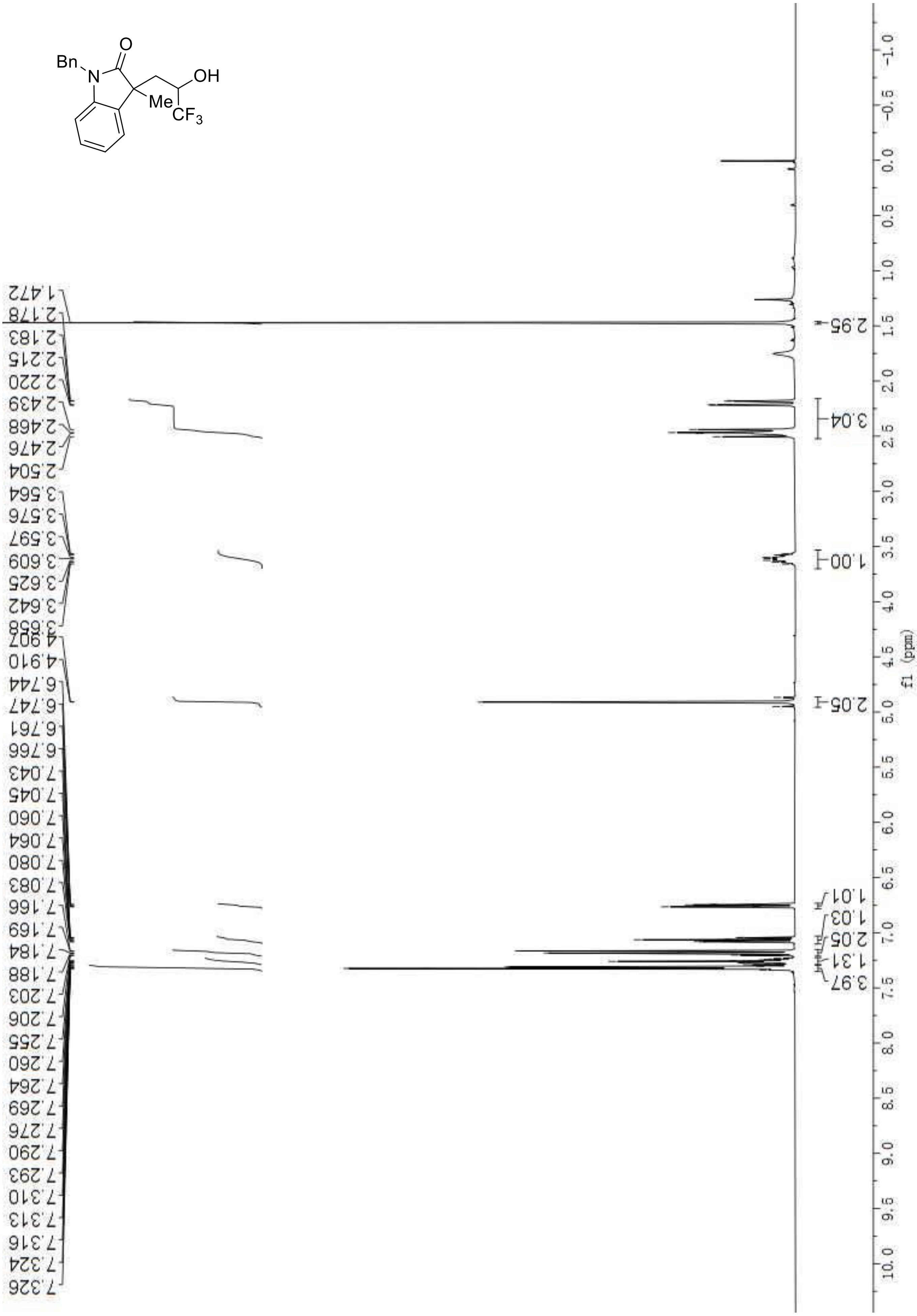

^{13}C NMR of **11c** ($CDCl_3$, 150 MHz, 25 ºC)

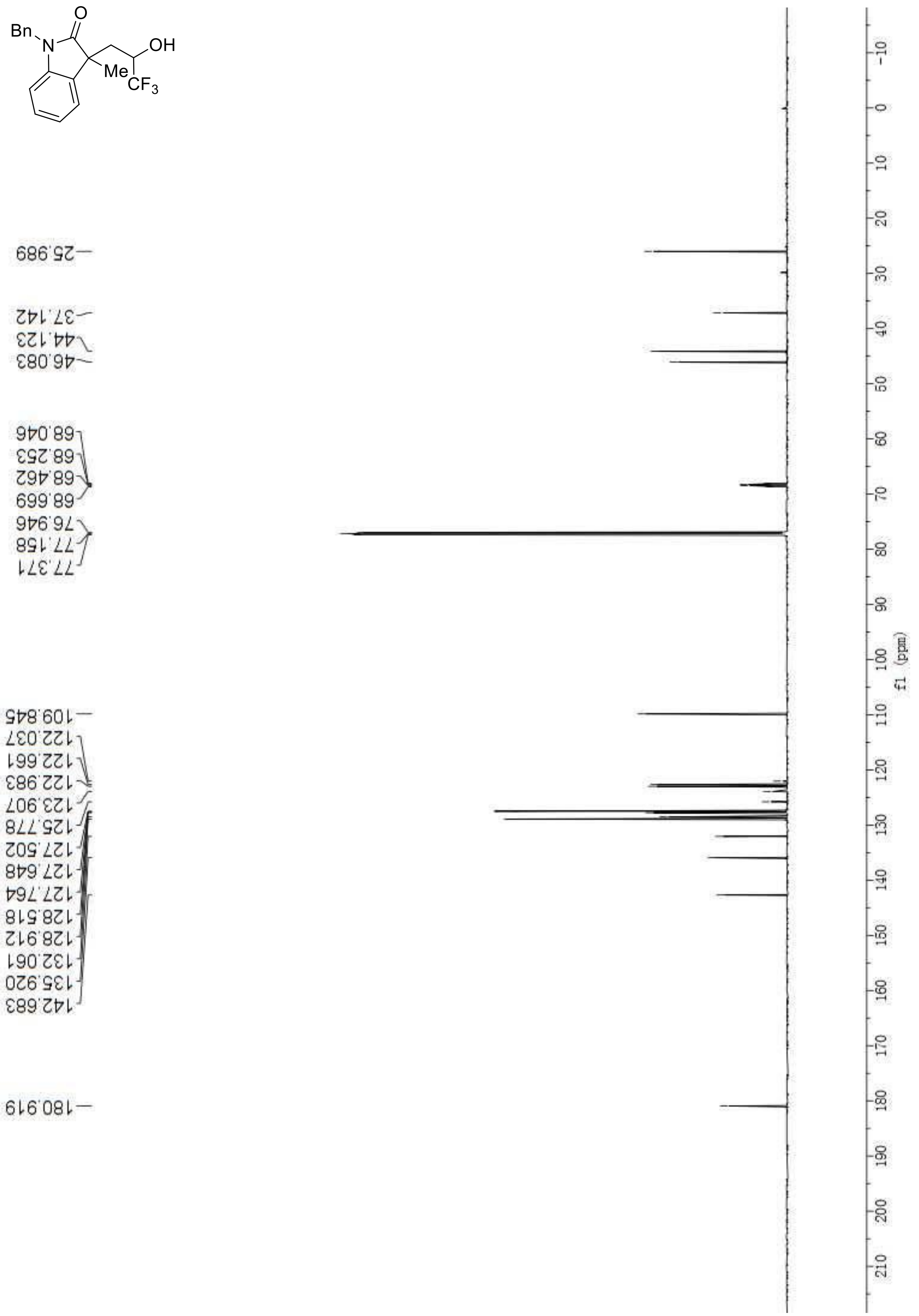

^{19}F NMR of **11c** ($CDCl_3$, 375 MHz, 25 ºC)

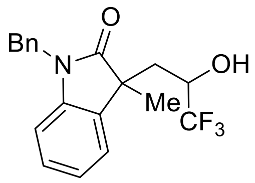

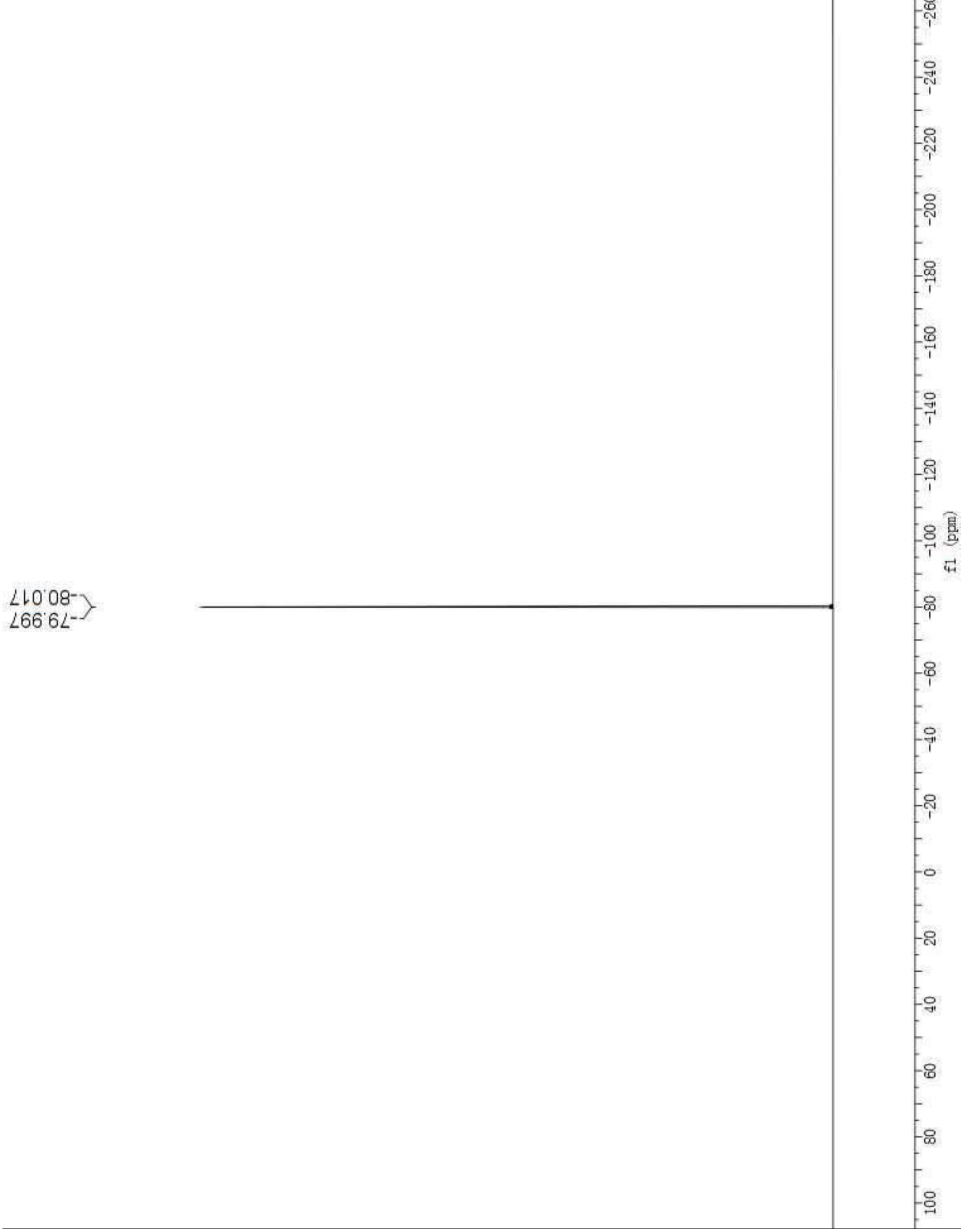

^{1}H NMR of **11d** ($CDCl_3$, 400 MHz, 25 ºC)

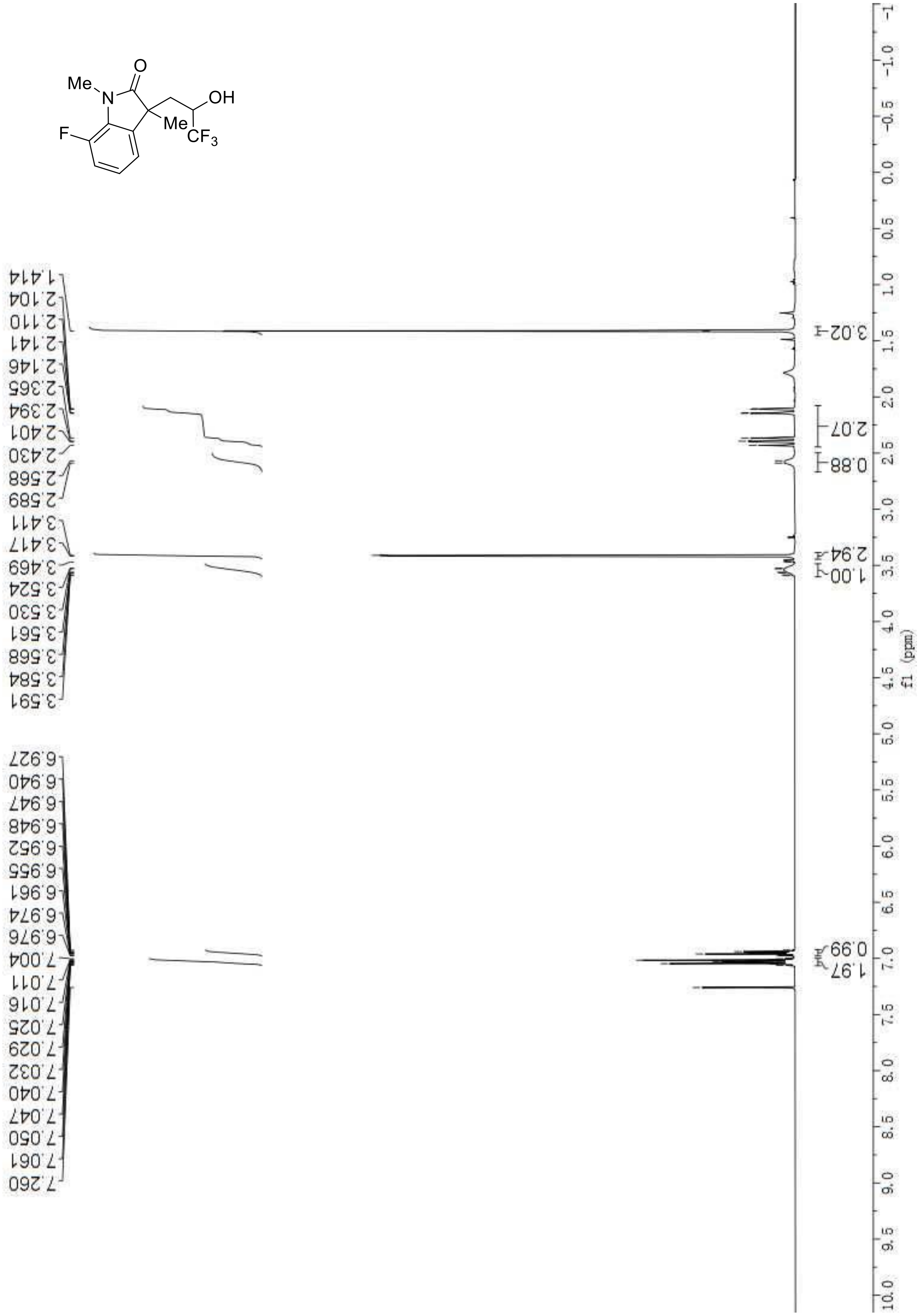

^{13}C NMR of **11d** ($CDCl_3$, 150 MHz, 25 ºC)

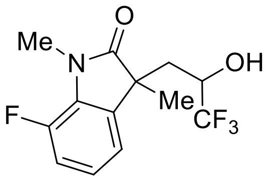

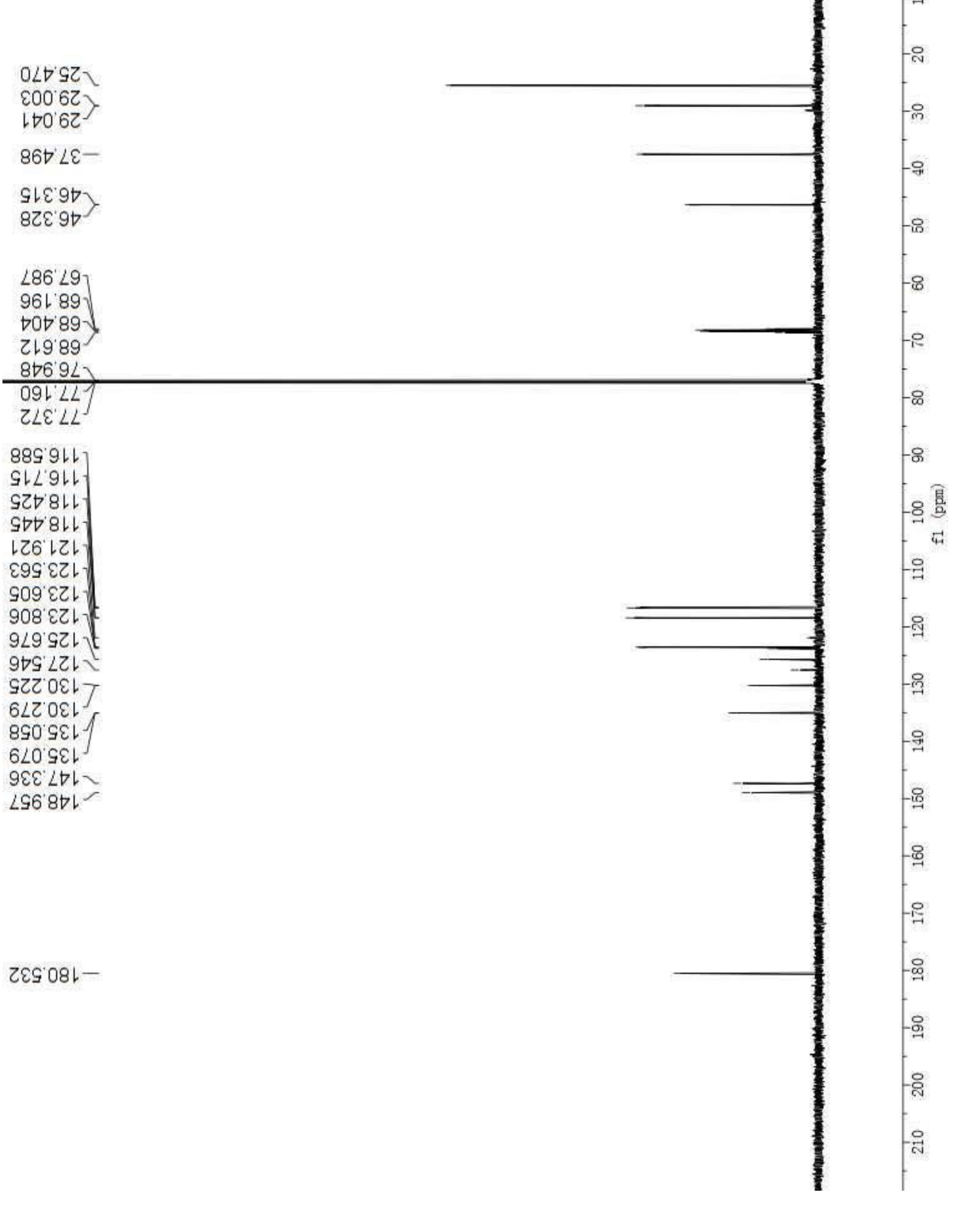

^{19}F NMR of **11d** ($CDCl_3$, 375 MHz, 25 ºC)

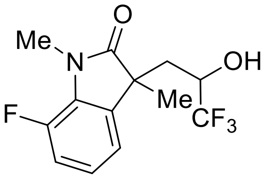

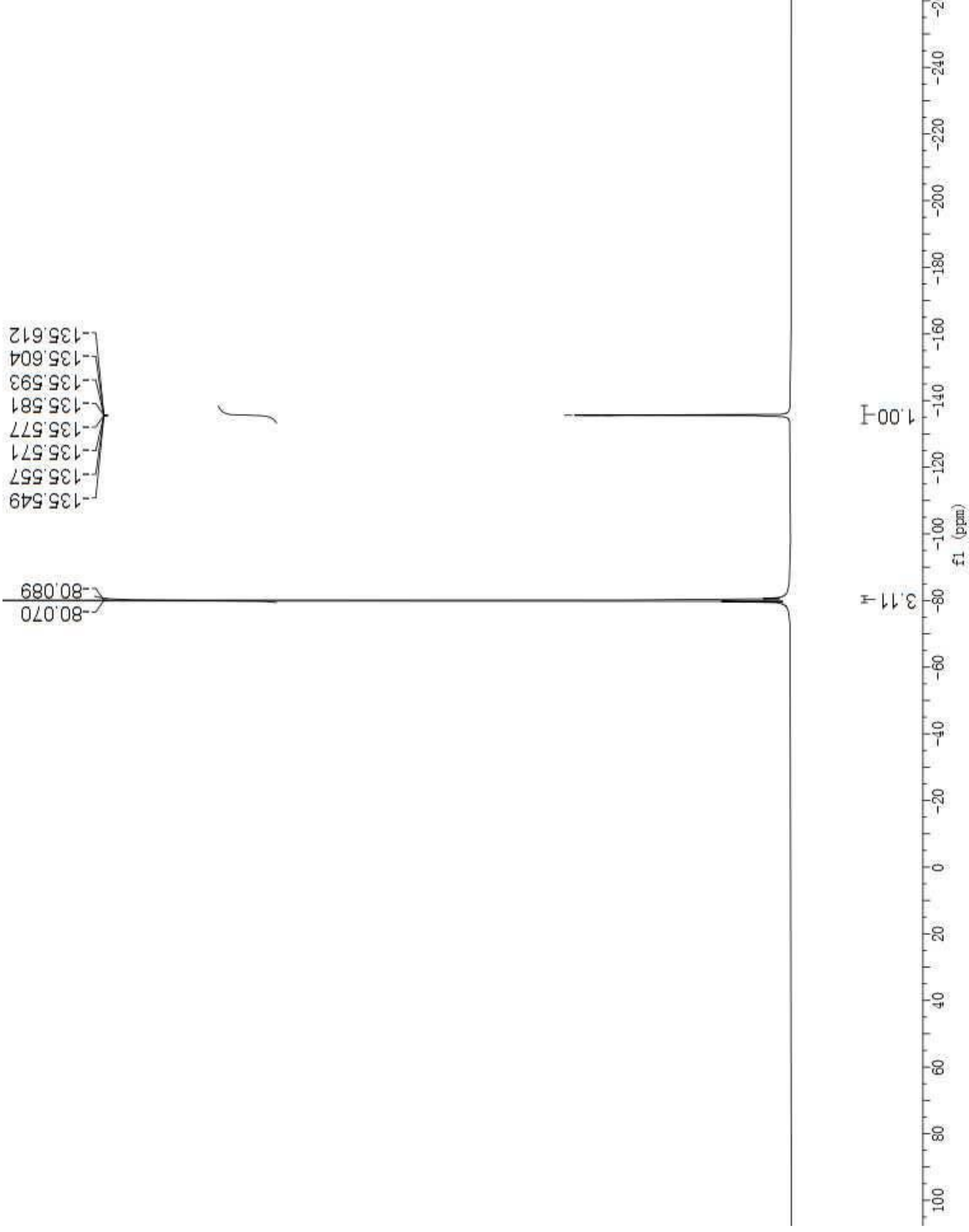

^{1}H NMR of **11e**($CDCl_3$, 400 MHz, 25 ºC)

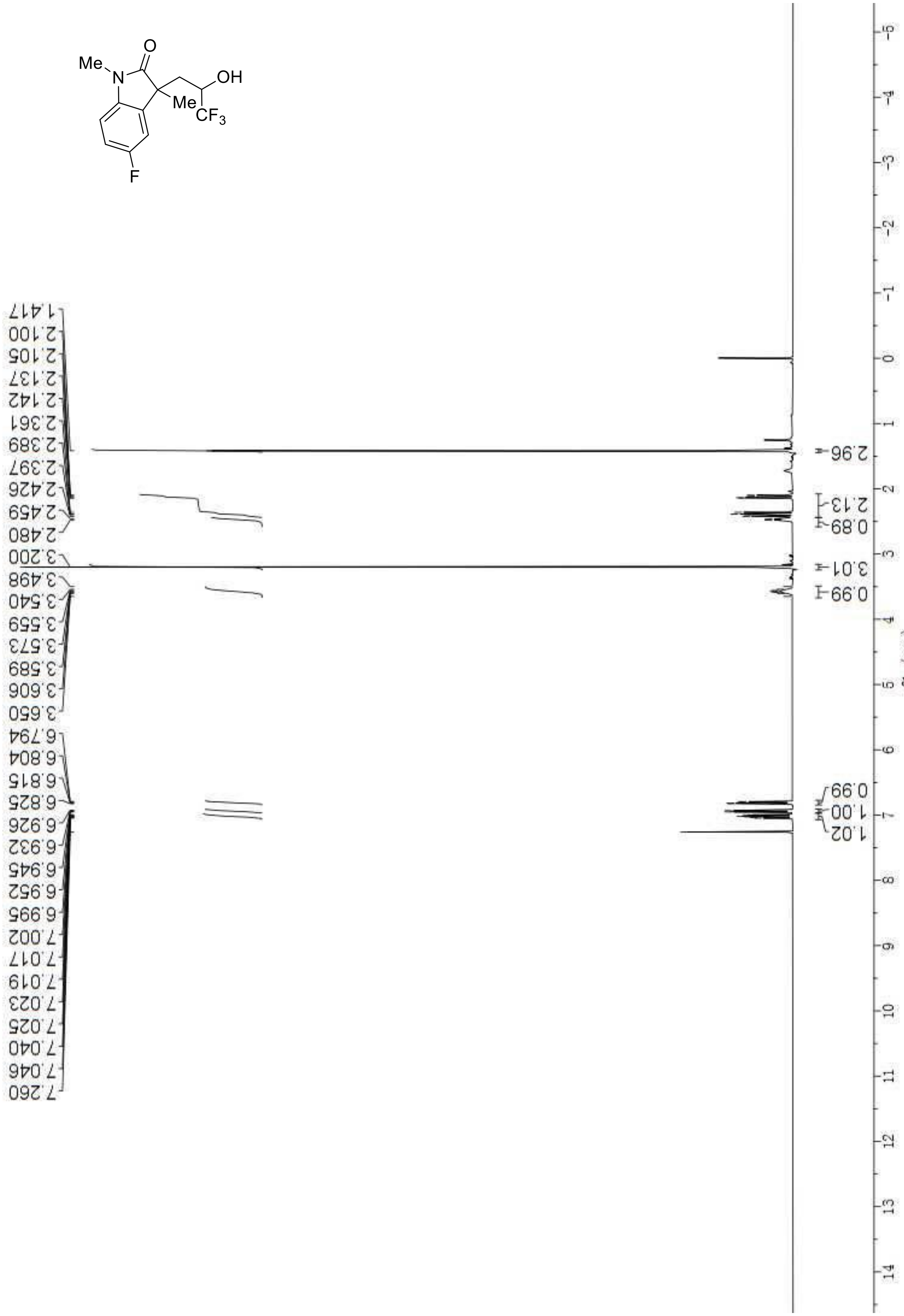

^{13}C NMR of **11e** ($CDCl_3$, 150 MHz, 25 ºC)

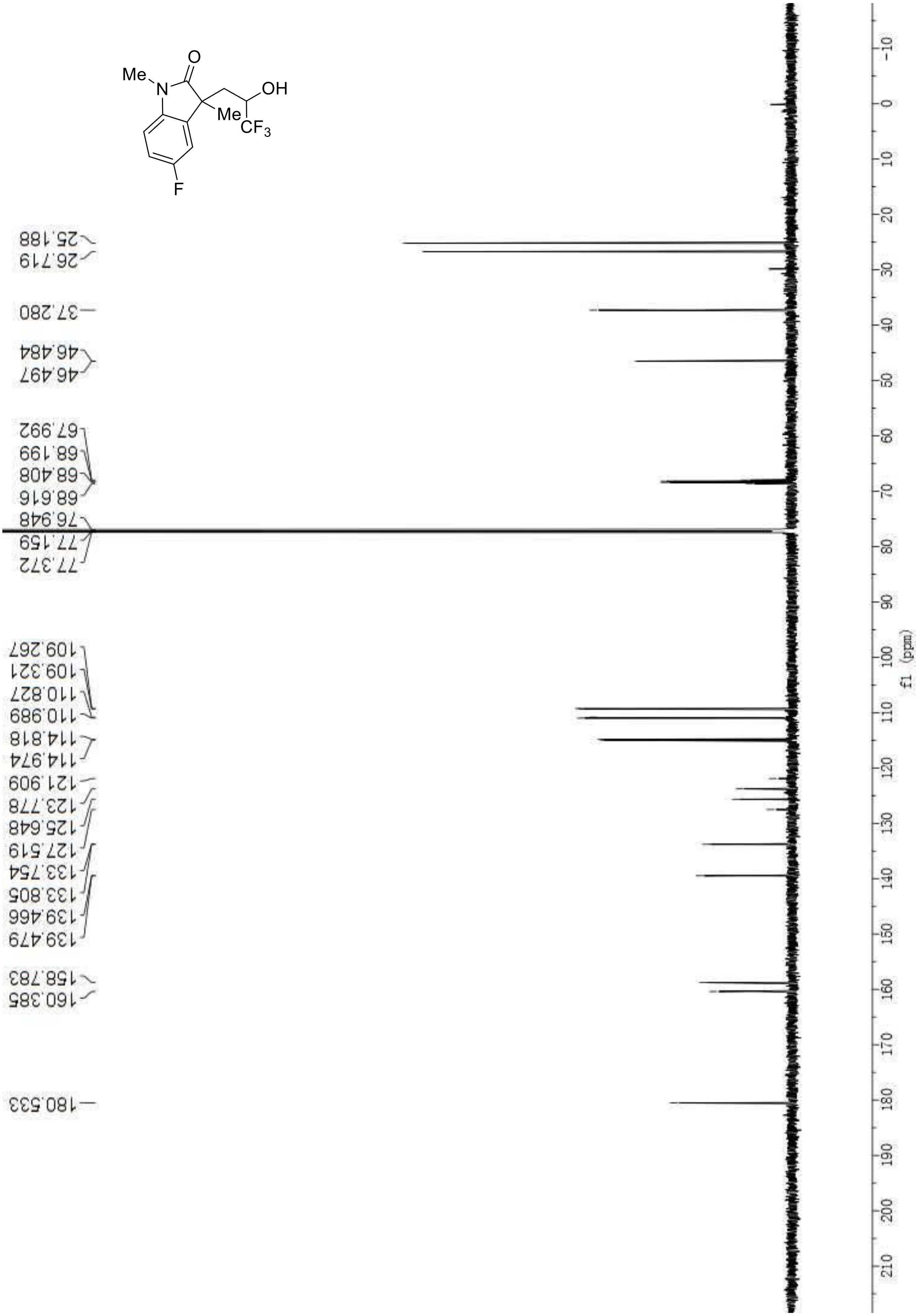

^{19}F NMR of **11e** ($CDCl_3$, 375 MHz, 25 °C)

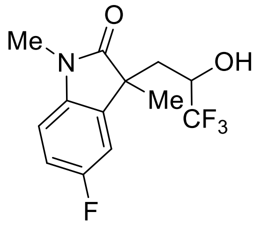

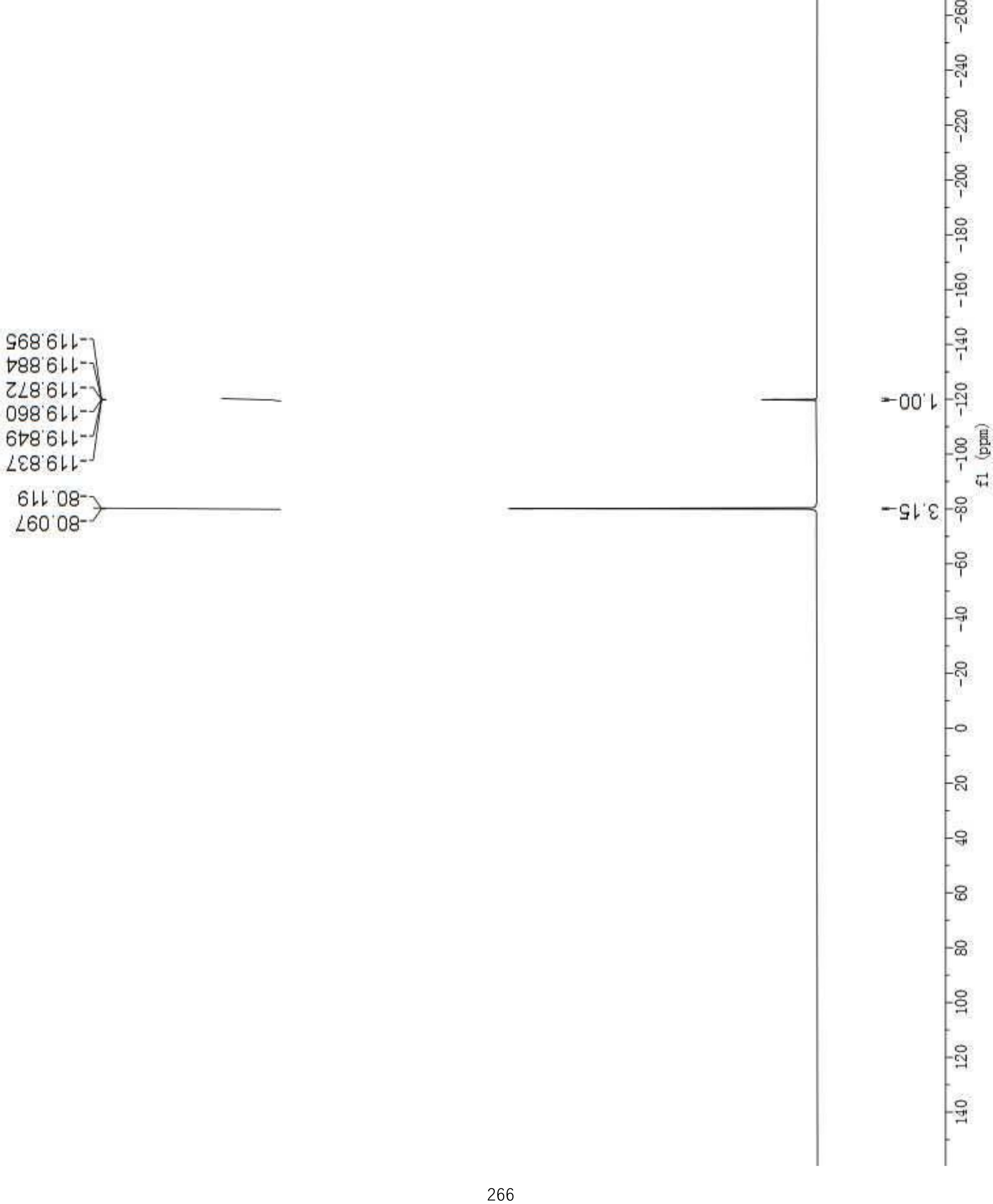

^{1}H NMR of **11f** ($CDCl_3$, 400 MHz, 25 ºC)

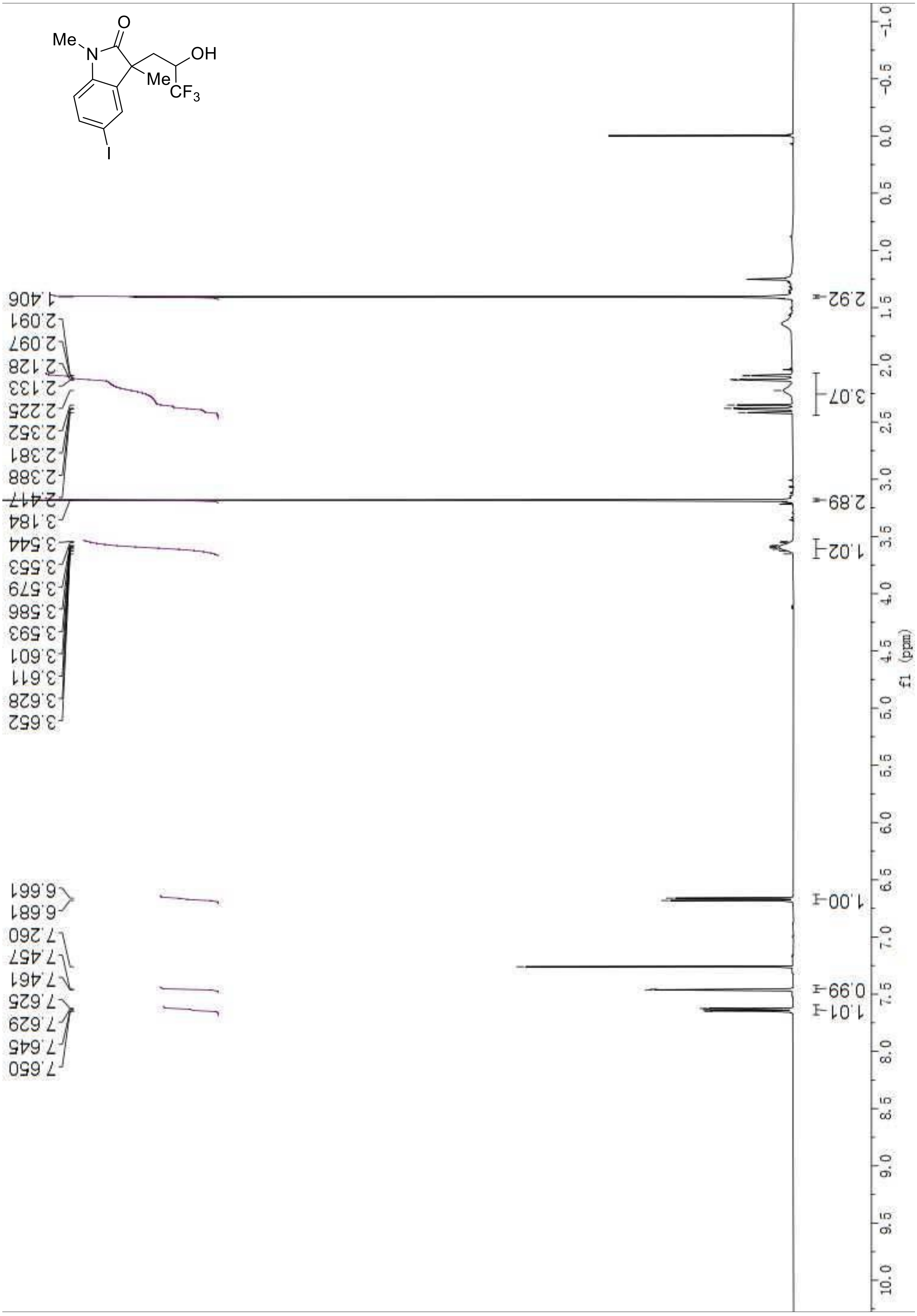

^{13}C NMR of **11f** ($CDCl_3$, 150 MHz, 25 °C)

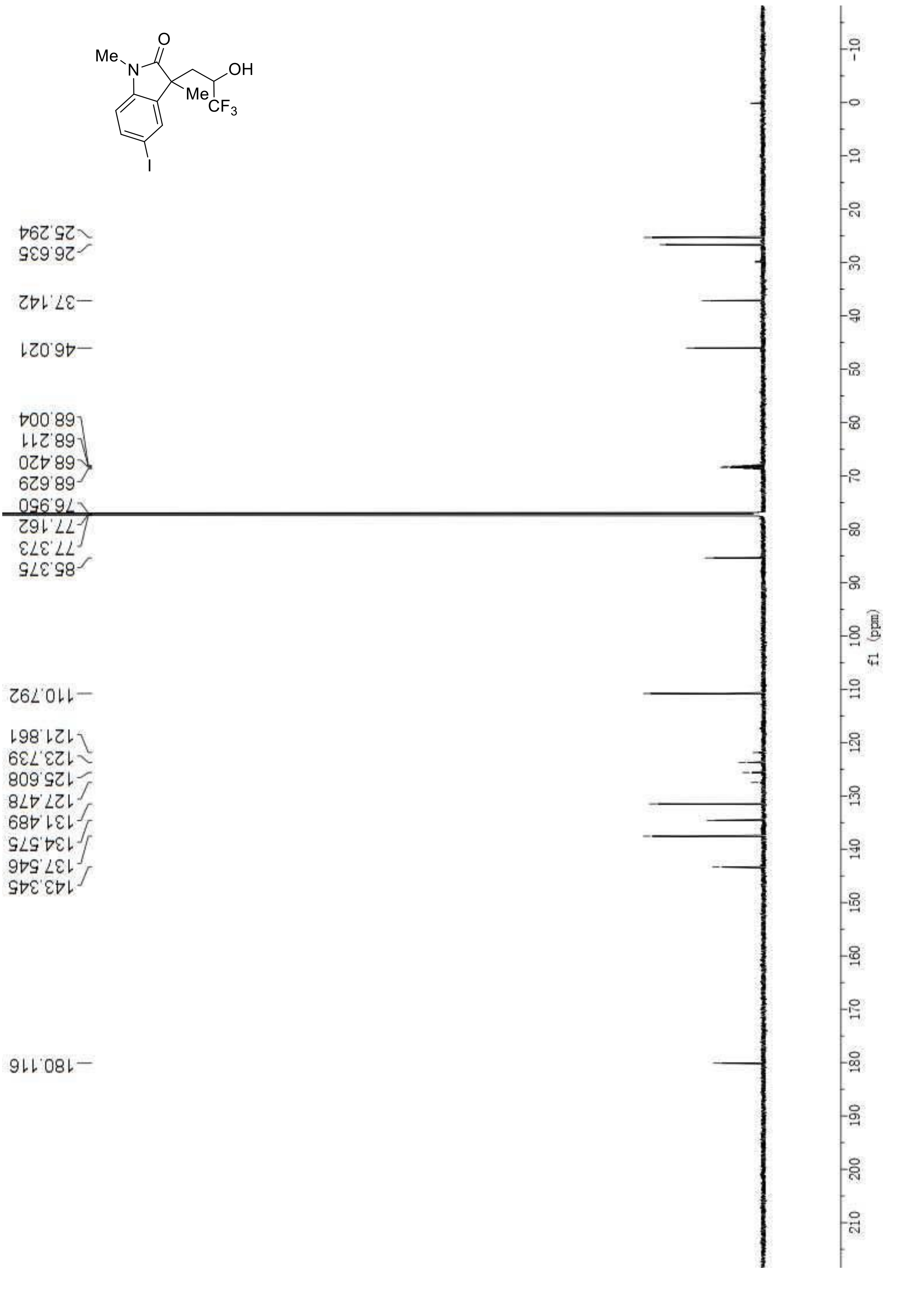

^{19}F NMR of **11f** ($CDCl_3$, 375 MHz, 25 ºC)

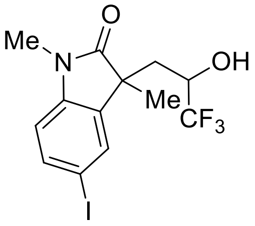

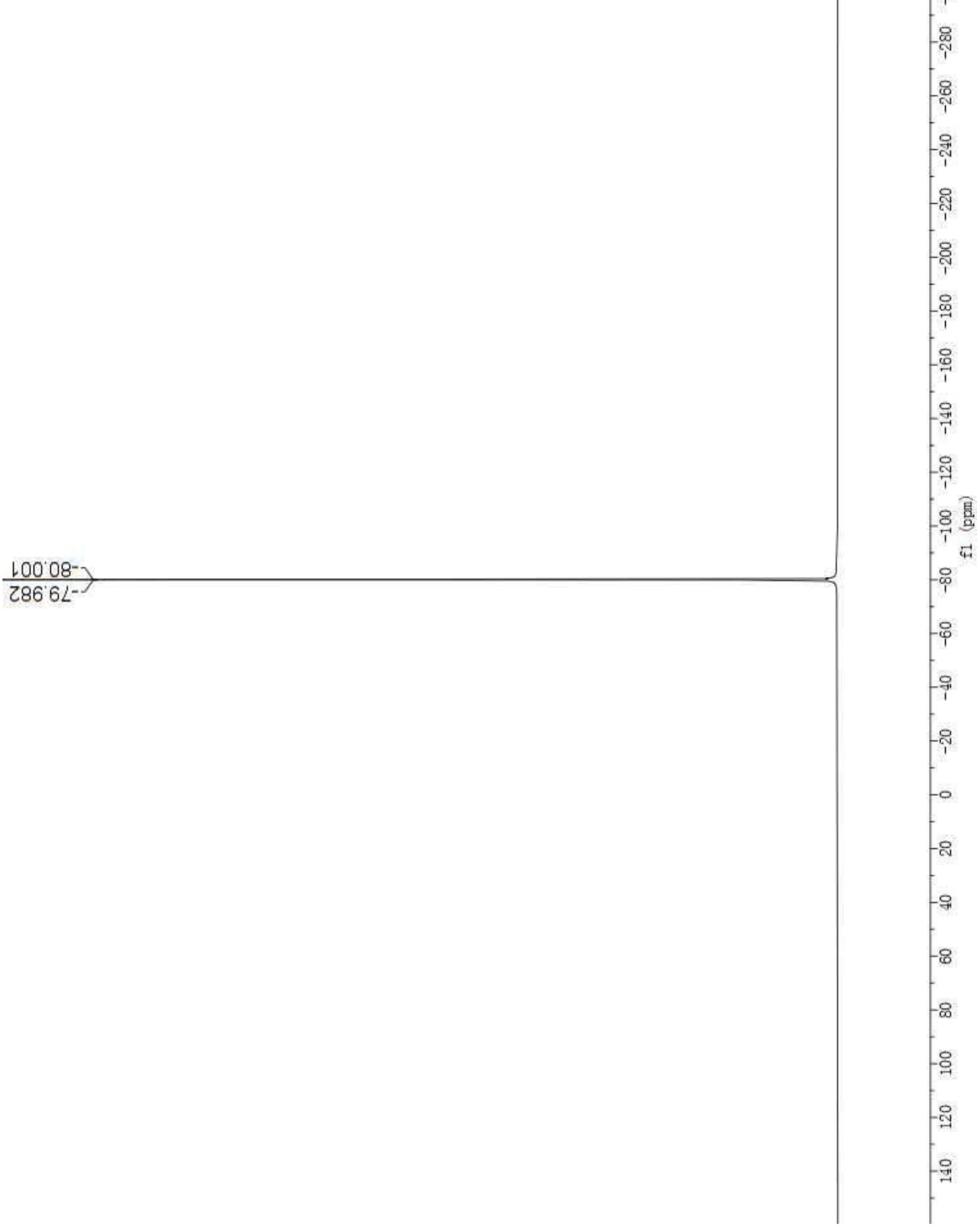

^{1}H NMR of **11g** ($CDCl_3$, 400 MHz, 25 ºC)

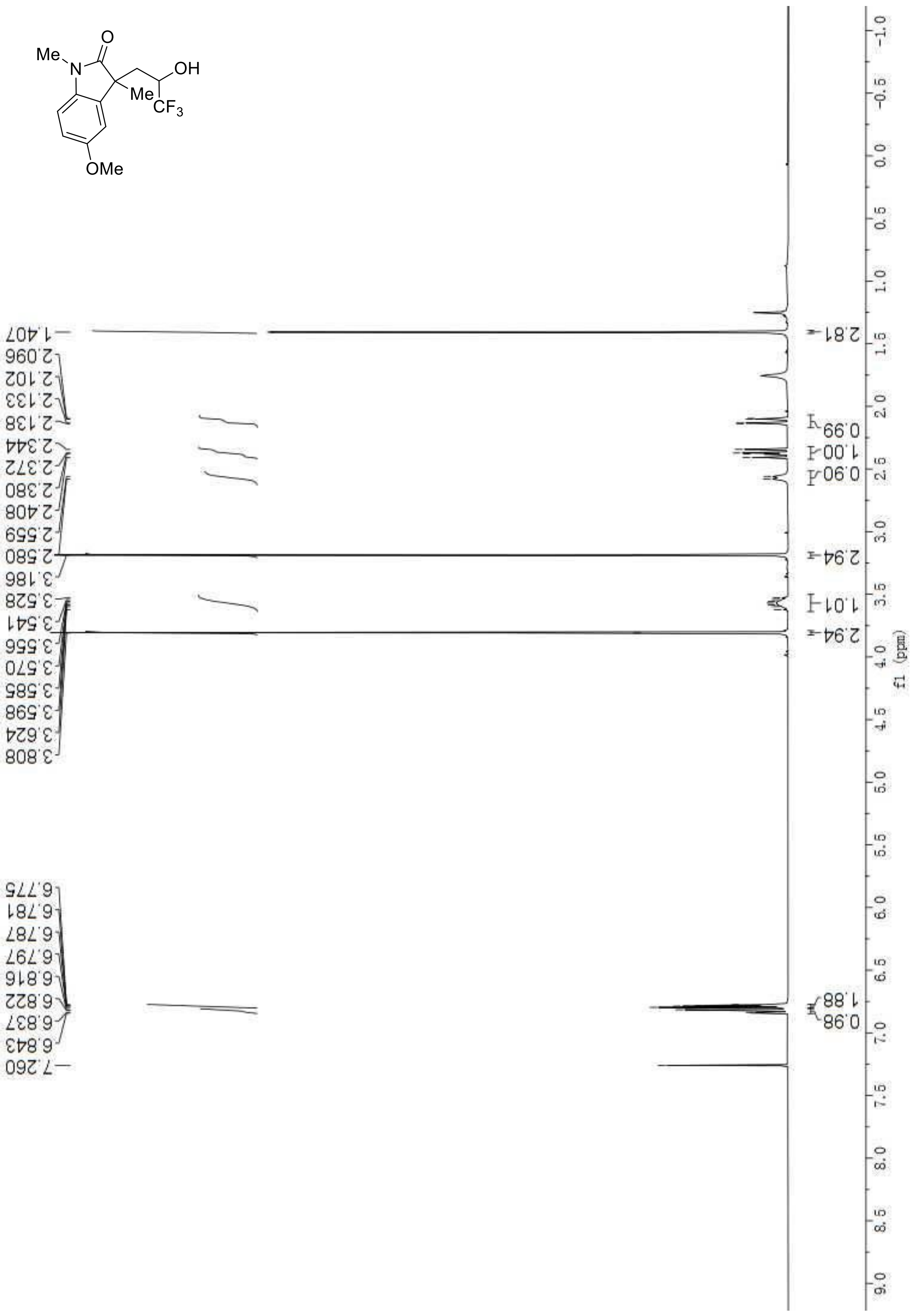

^{13}C NMR of **11g** ($CDCl_3$, 150 MHz, 25 ºC)

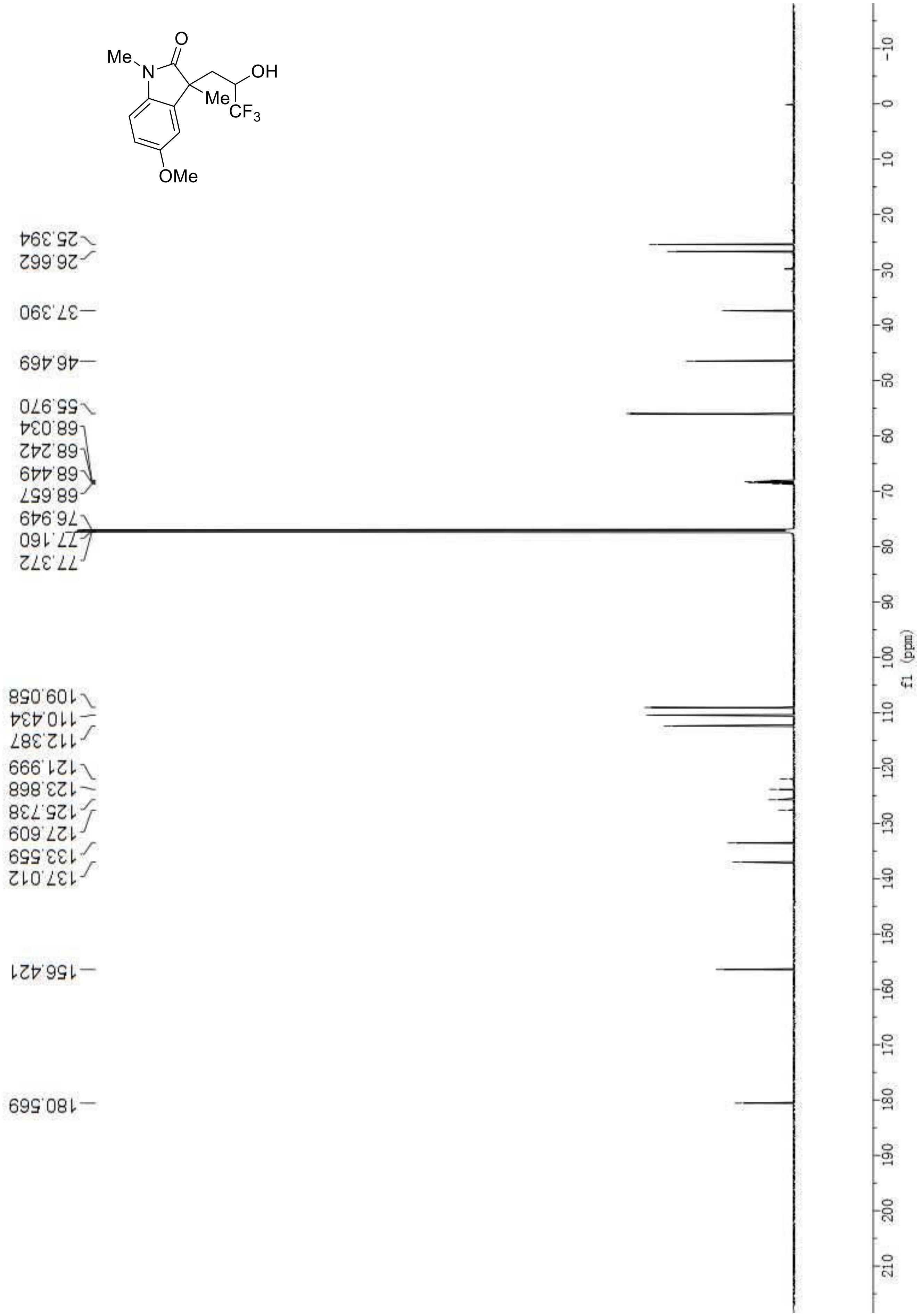

^{19}F NMR of **11g** ($CDCl_3$, 375 MHz, 25 ºC)

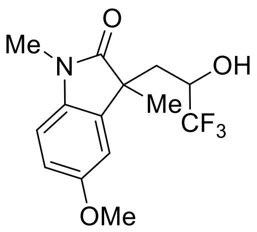

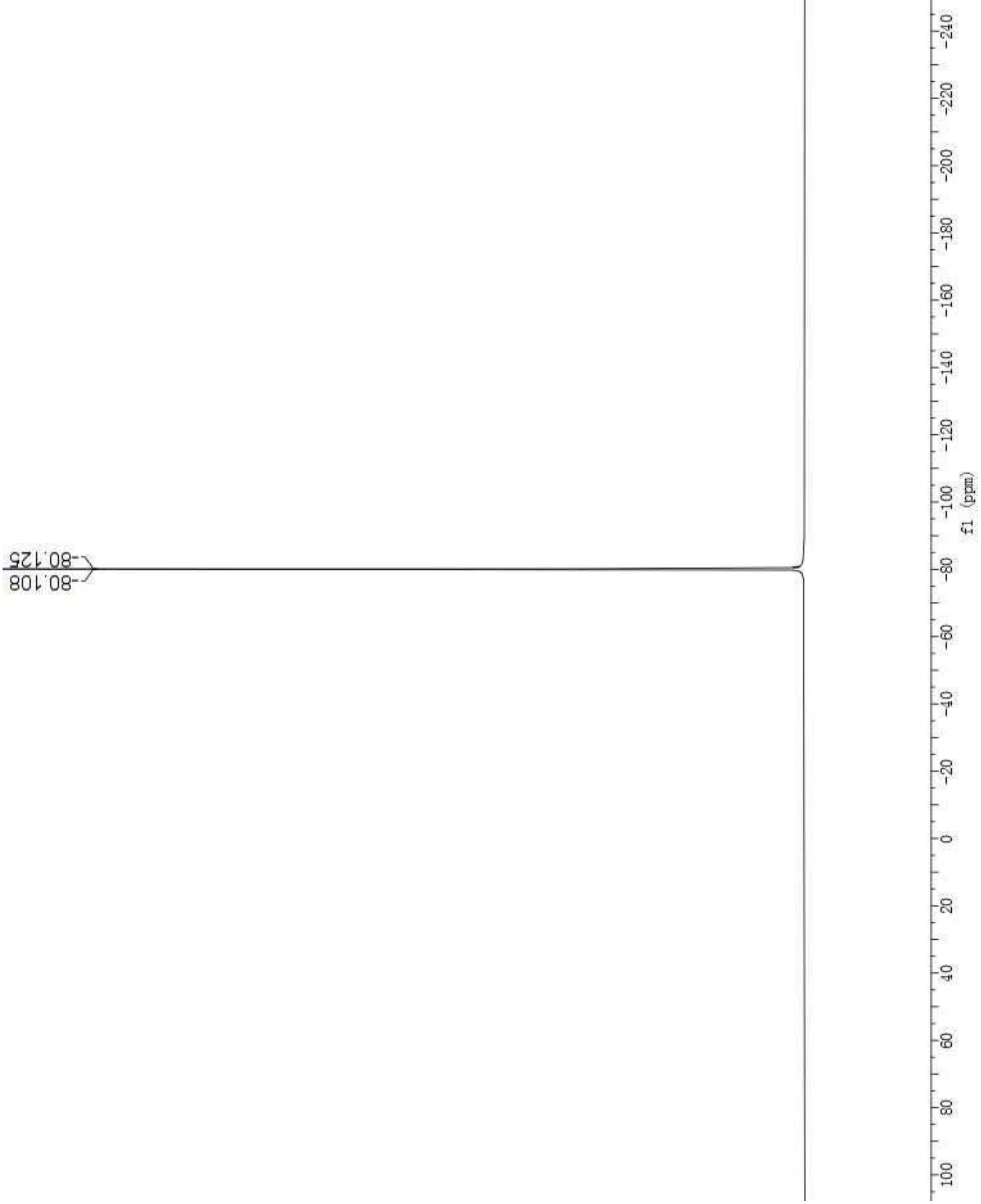

^{1}H NMR of **11h** ($CDCl_3$, 400 MHz, 25 ºC)

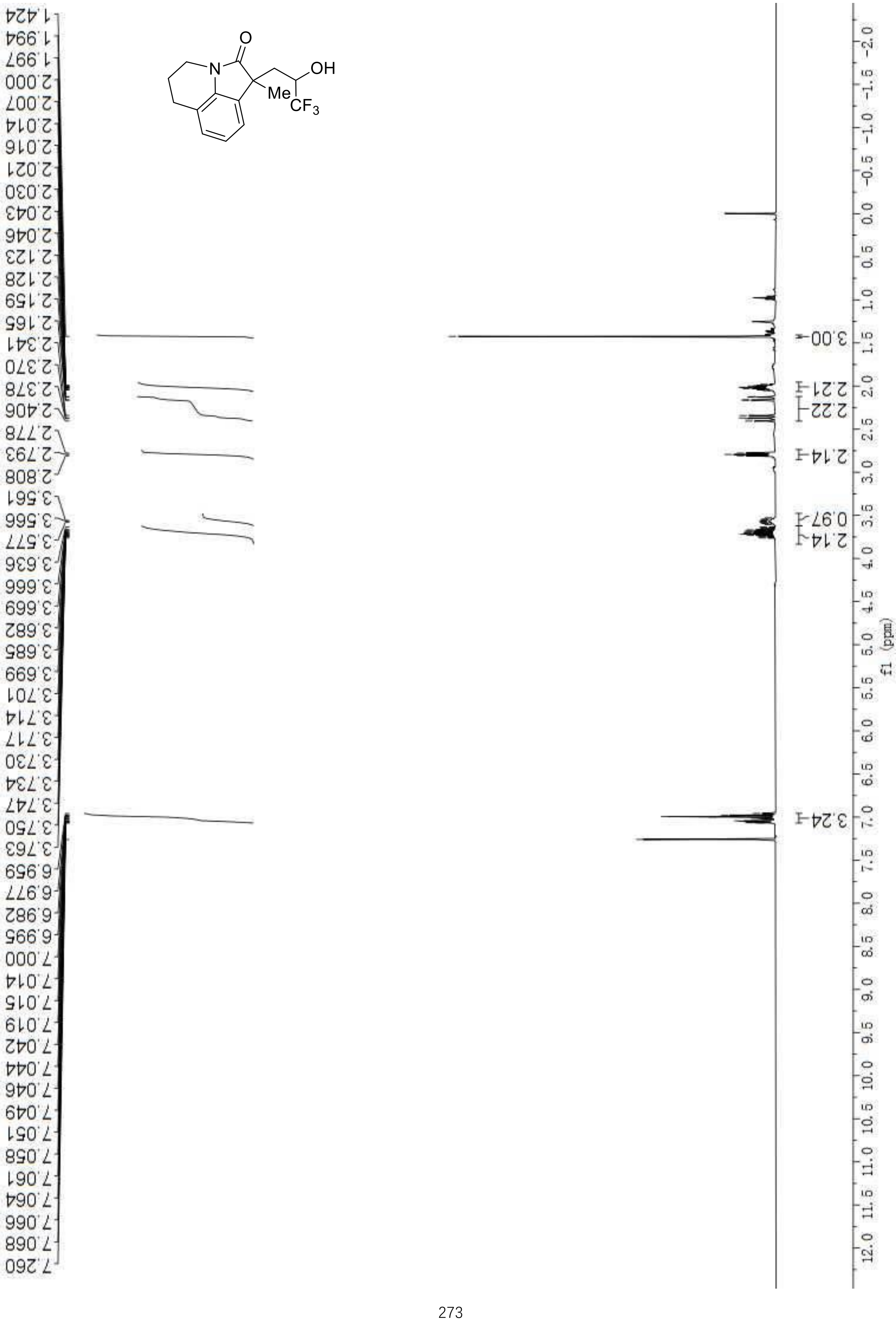

^{13}C NMR of **11h** ($CDCl_3$, 150 MHz, 25 ºC)

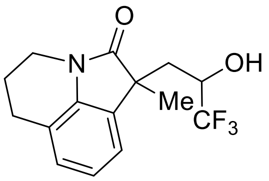

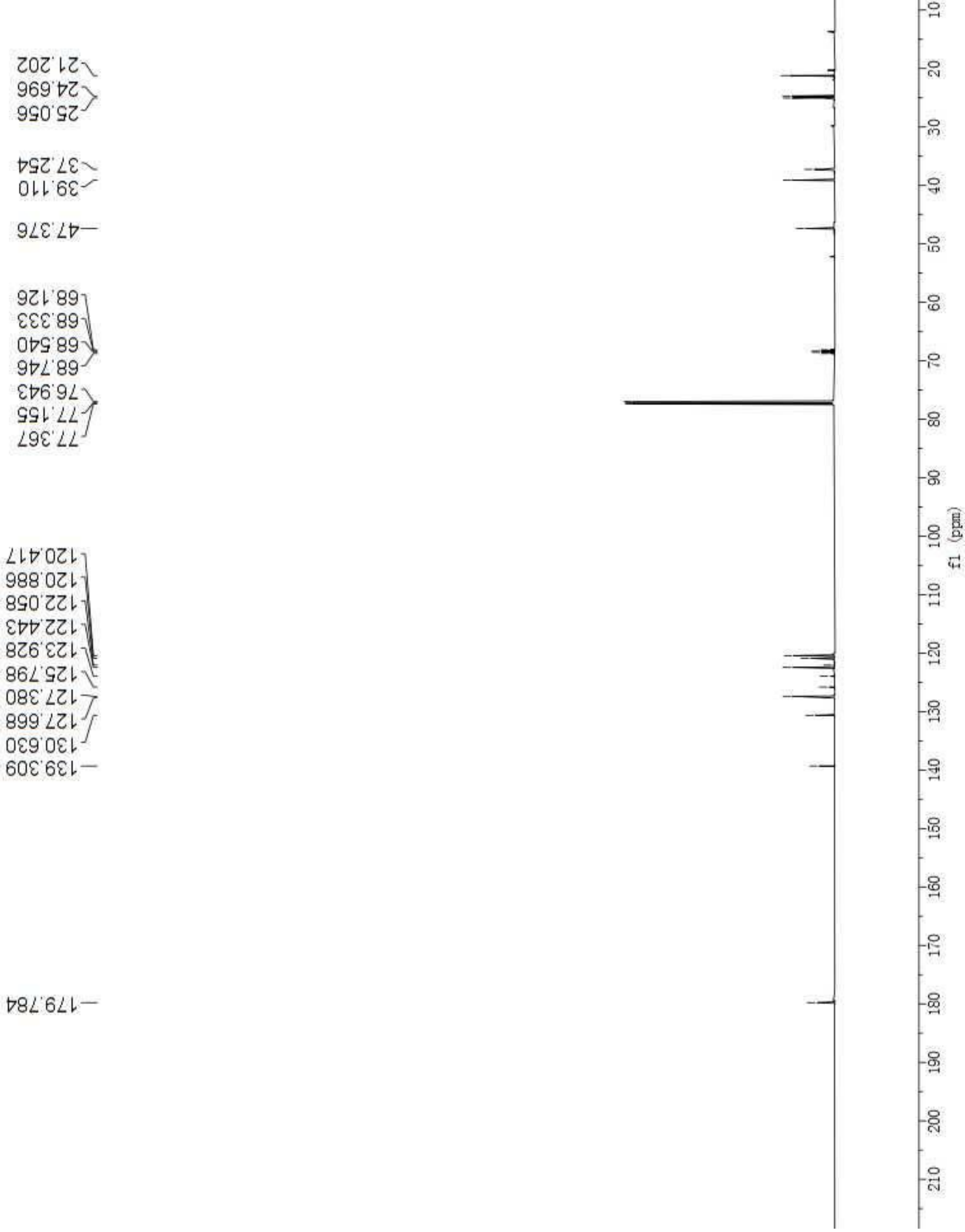

^{19}F NMR of **11h** ($CDCl_3$, 375 MHz, 25 ºC)

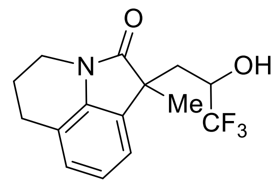

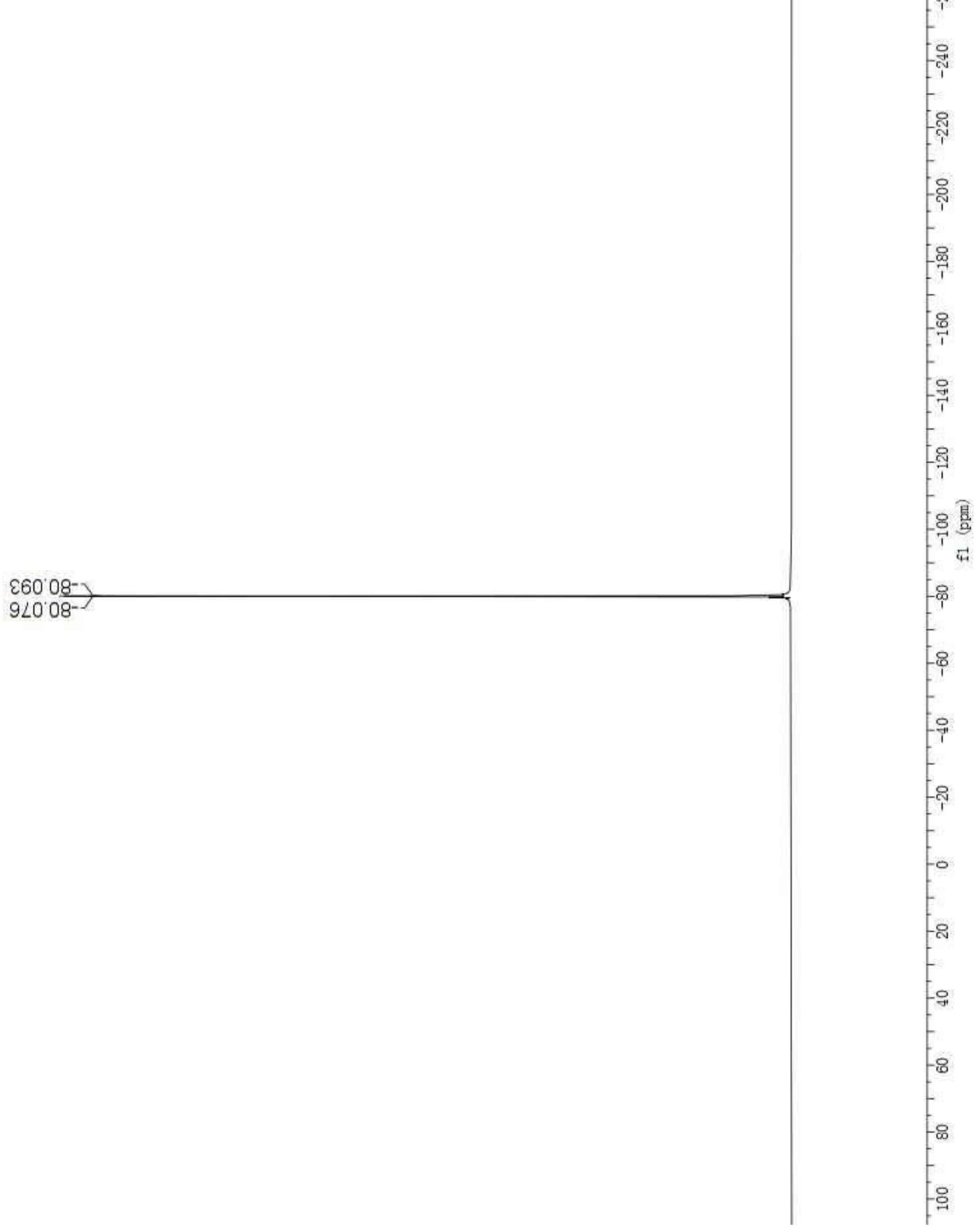

^{1}H NMR of **11i** ($CDCl_3$, 400 MHz, 25 ºC)

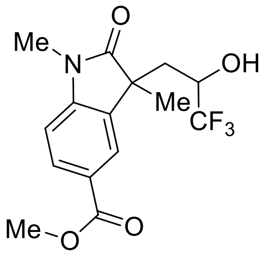

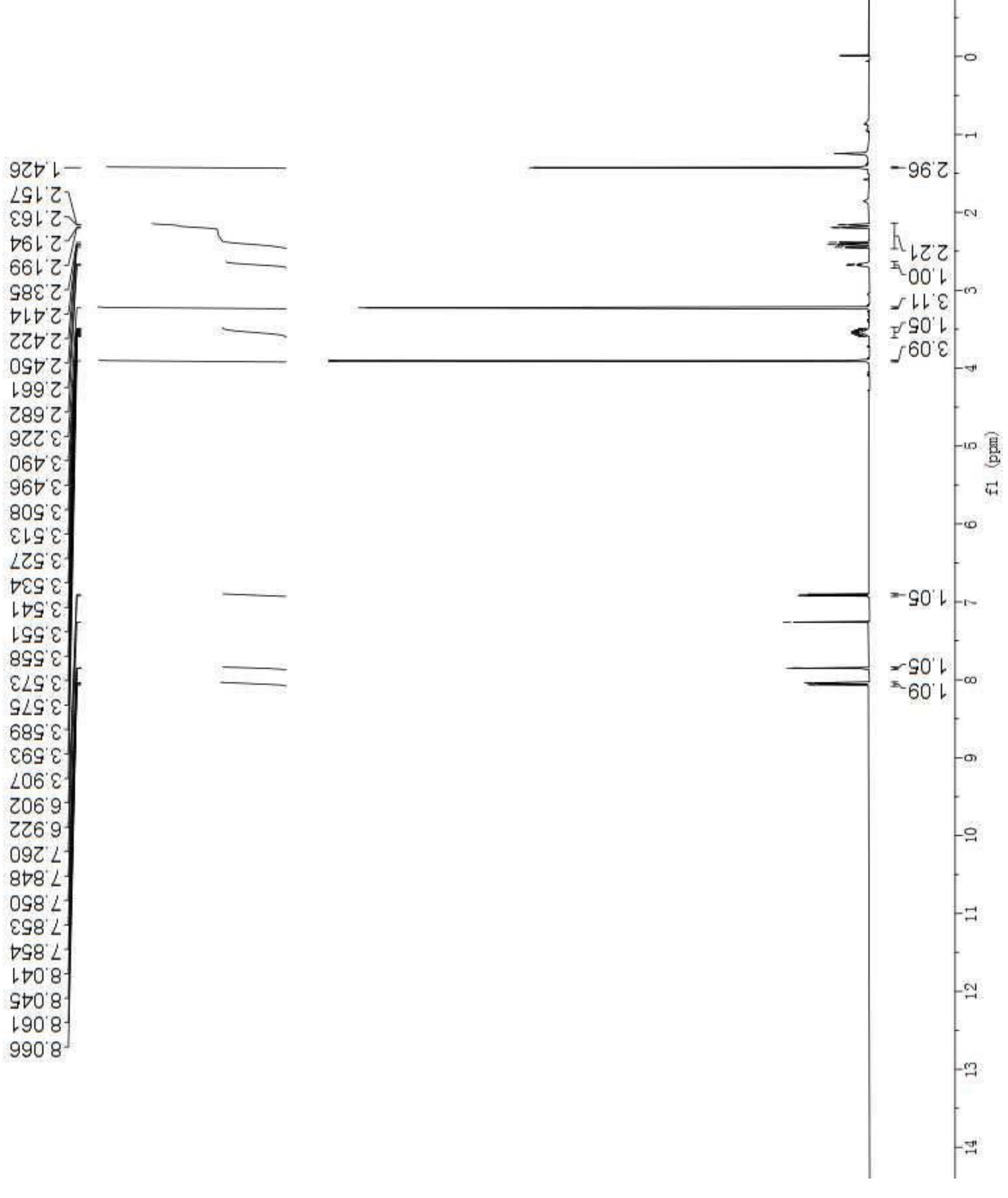

^{13}C NMR of **11i** ($CDCl_3$, 150 MHz, 25 ºC)

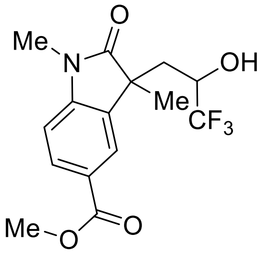

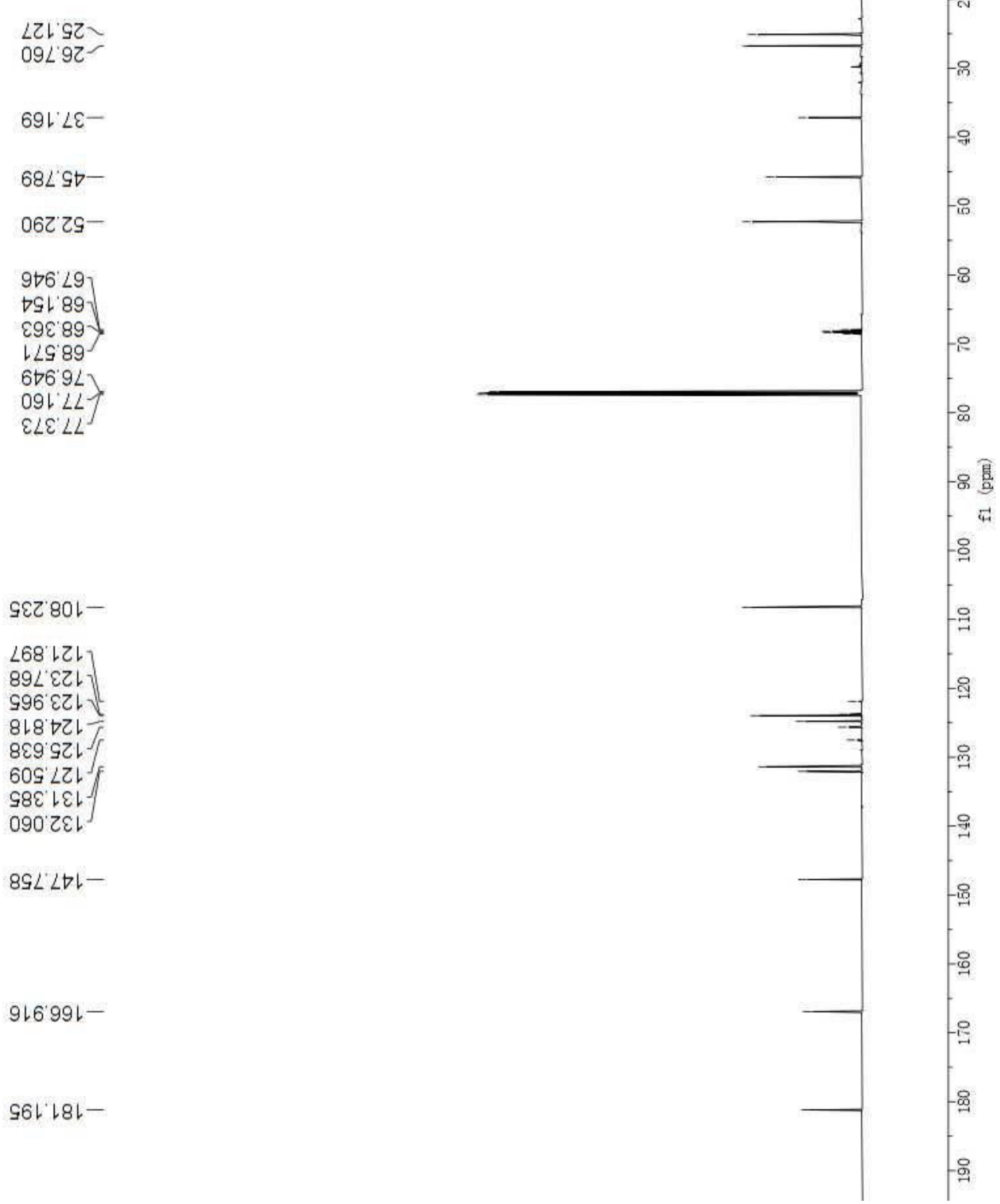

^{19}F NMR of **11i** ($CDCl_3$, 375 MHz, 25 ºC)

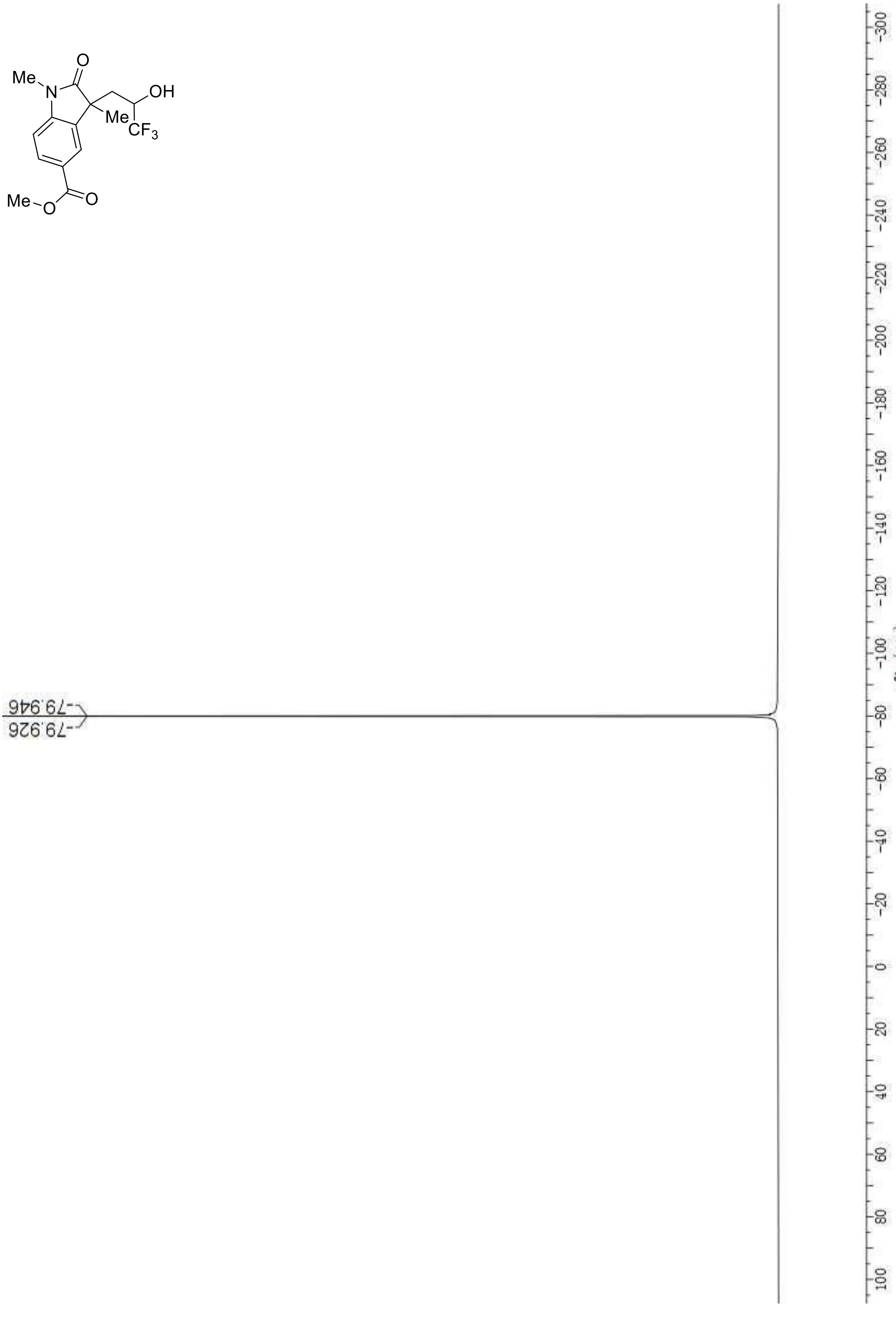

^{1}H NMR of **13a** ($CDCl_3$, 400 MHz, 25 ºC)

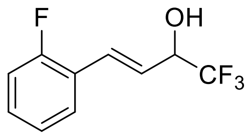

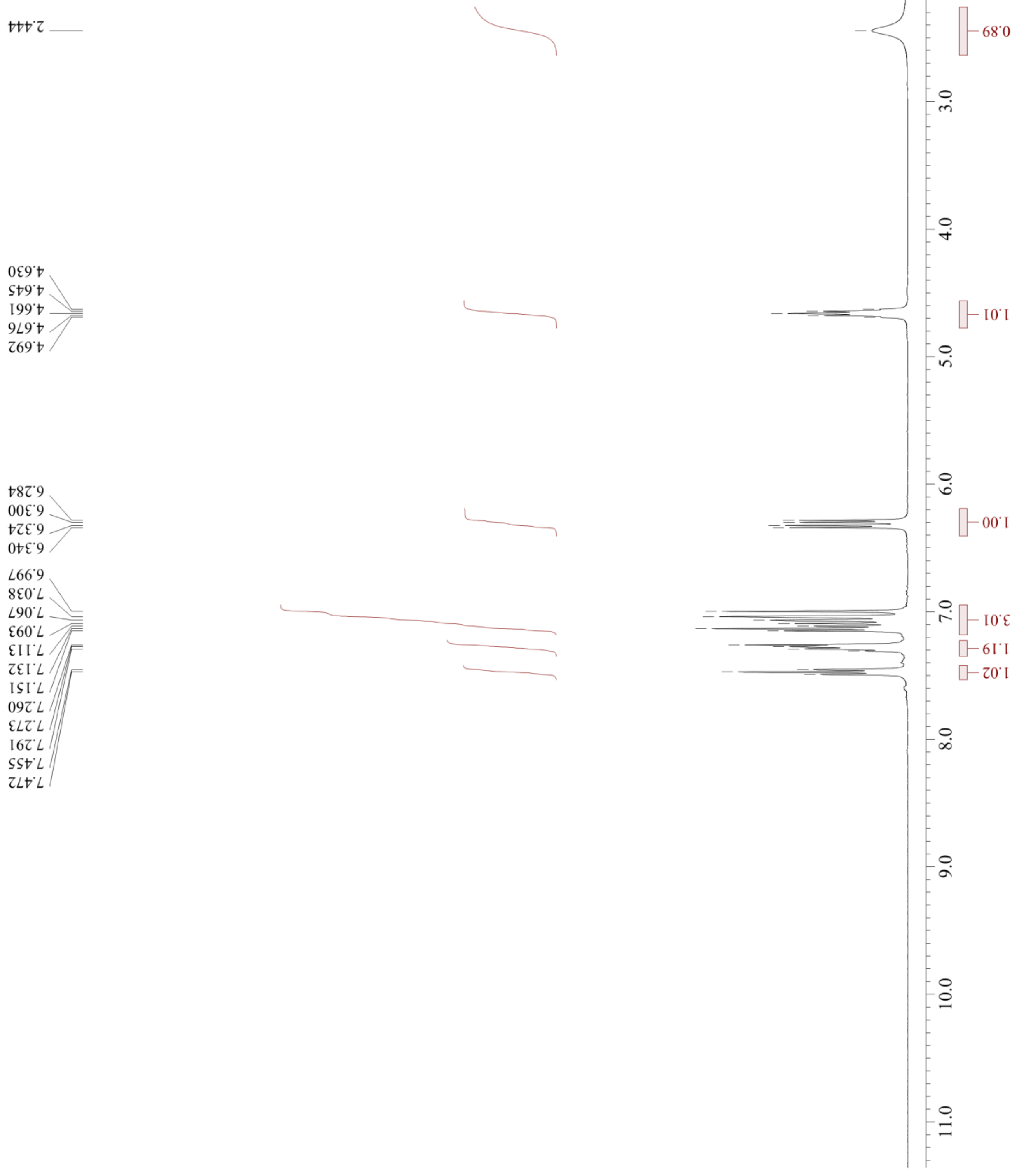

^{13}C NMR of **13a** ($CDCl_3$, 100 MHz, 25 ºC)

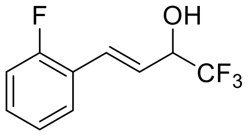

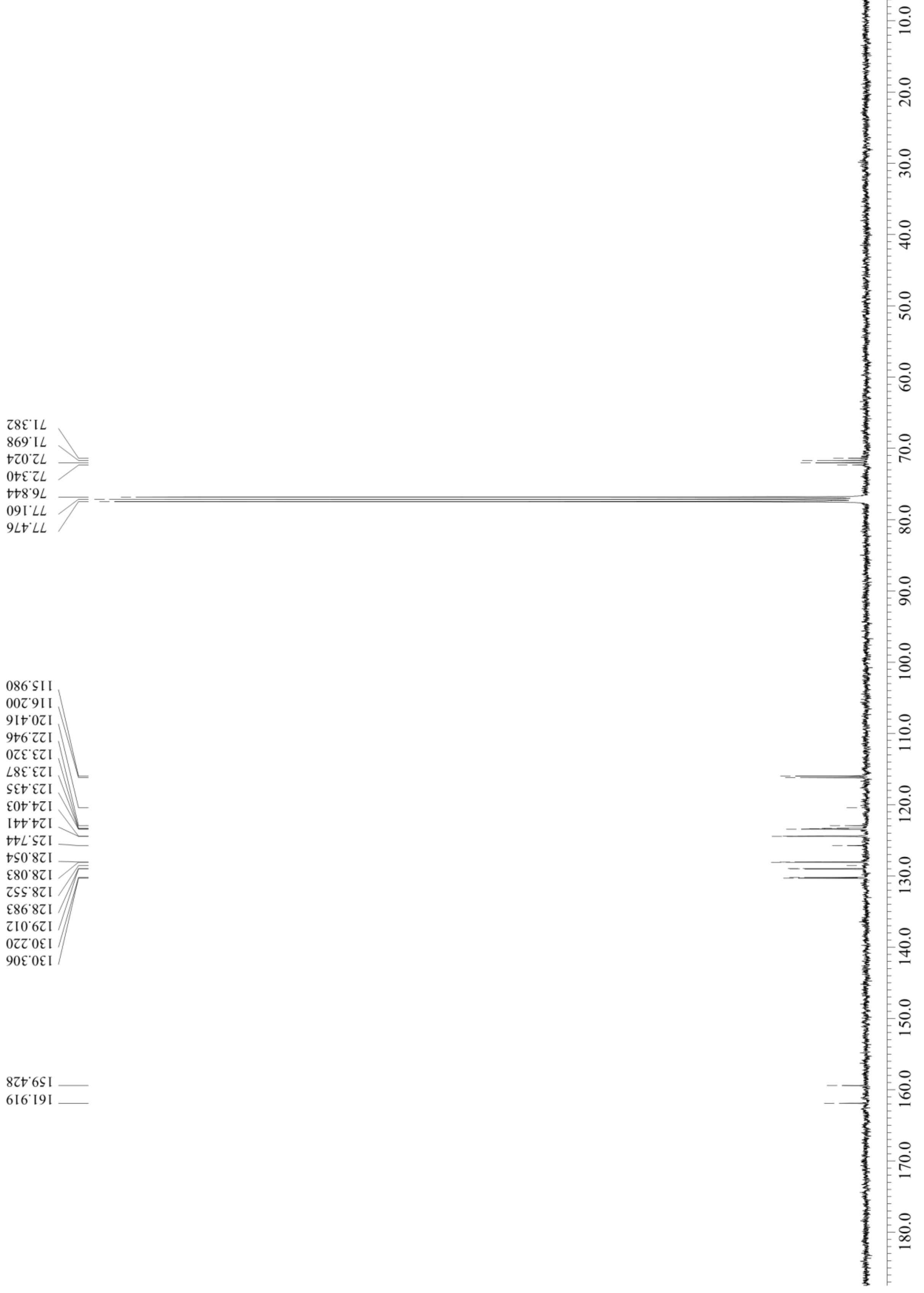

^{19}F NMR of **13a** ($CDCl_3$, 375 MHz, 25 ºC)

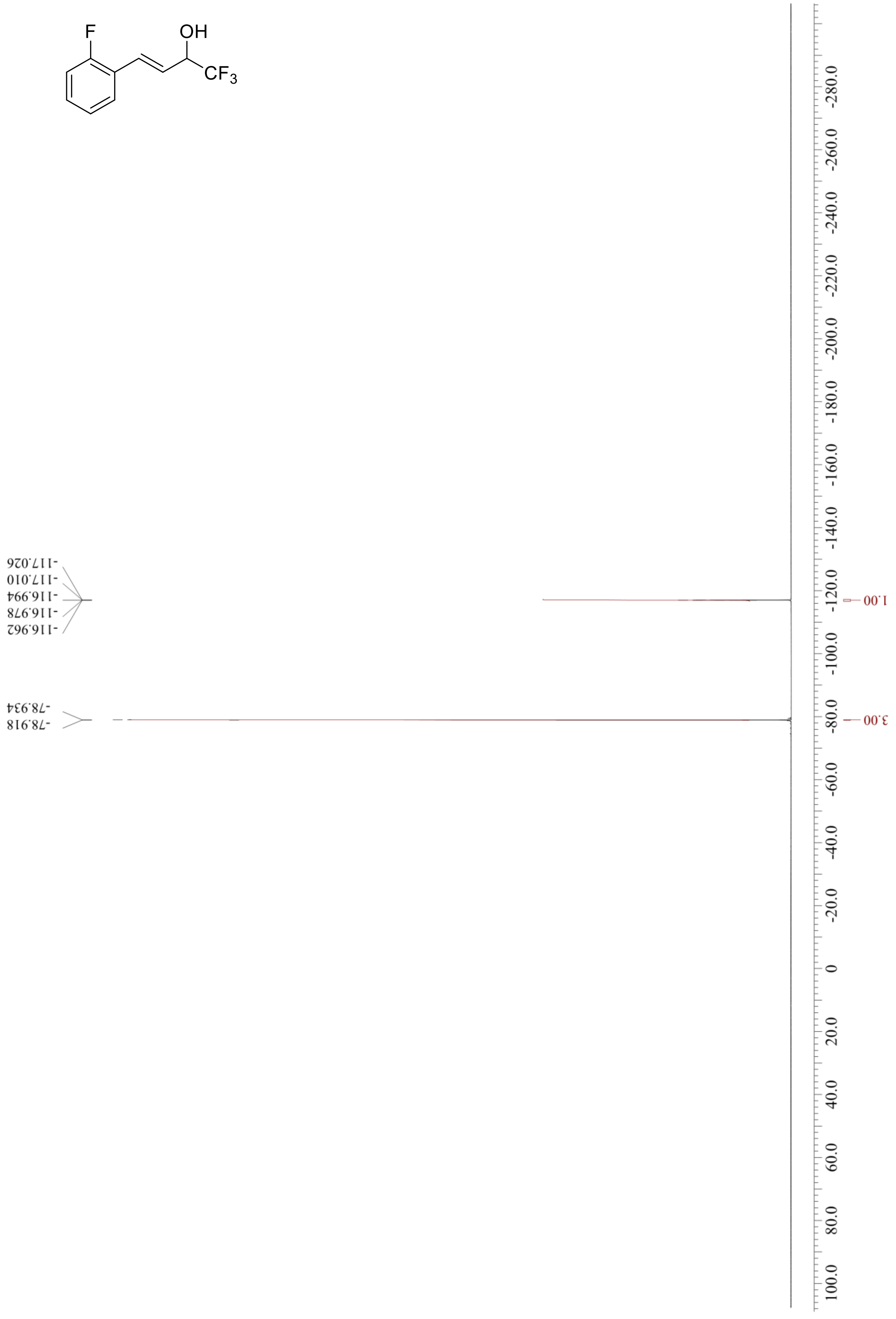

^{1}H NMR of **13b** ($CDCl_3$, 400 MHz, 25 °C)

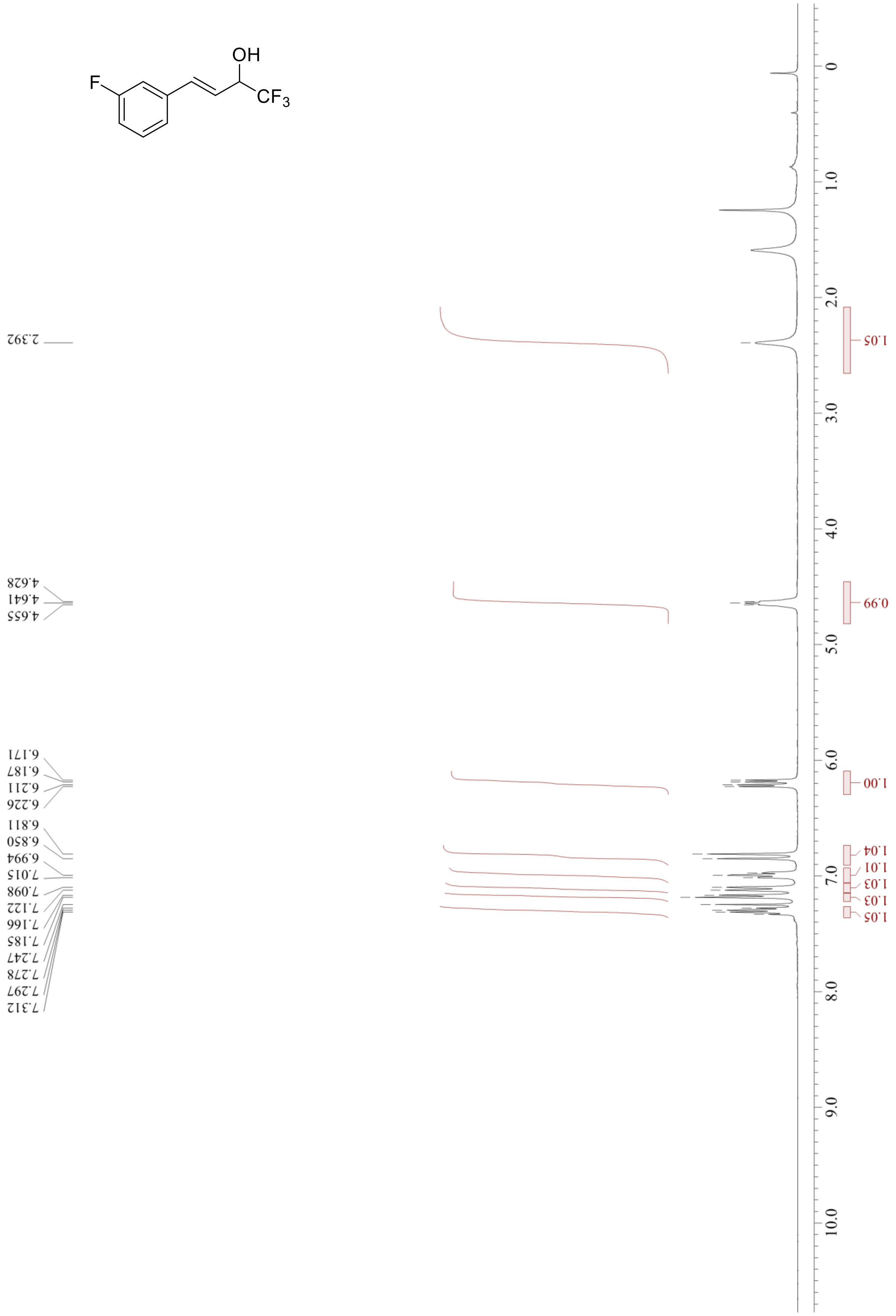

^{13}C NMR of **13b** ($CDCl_3$, 150 MHz, 25 ºC)

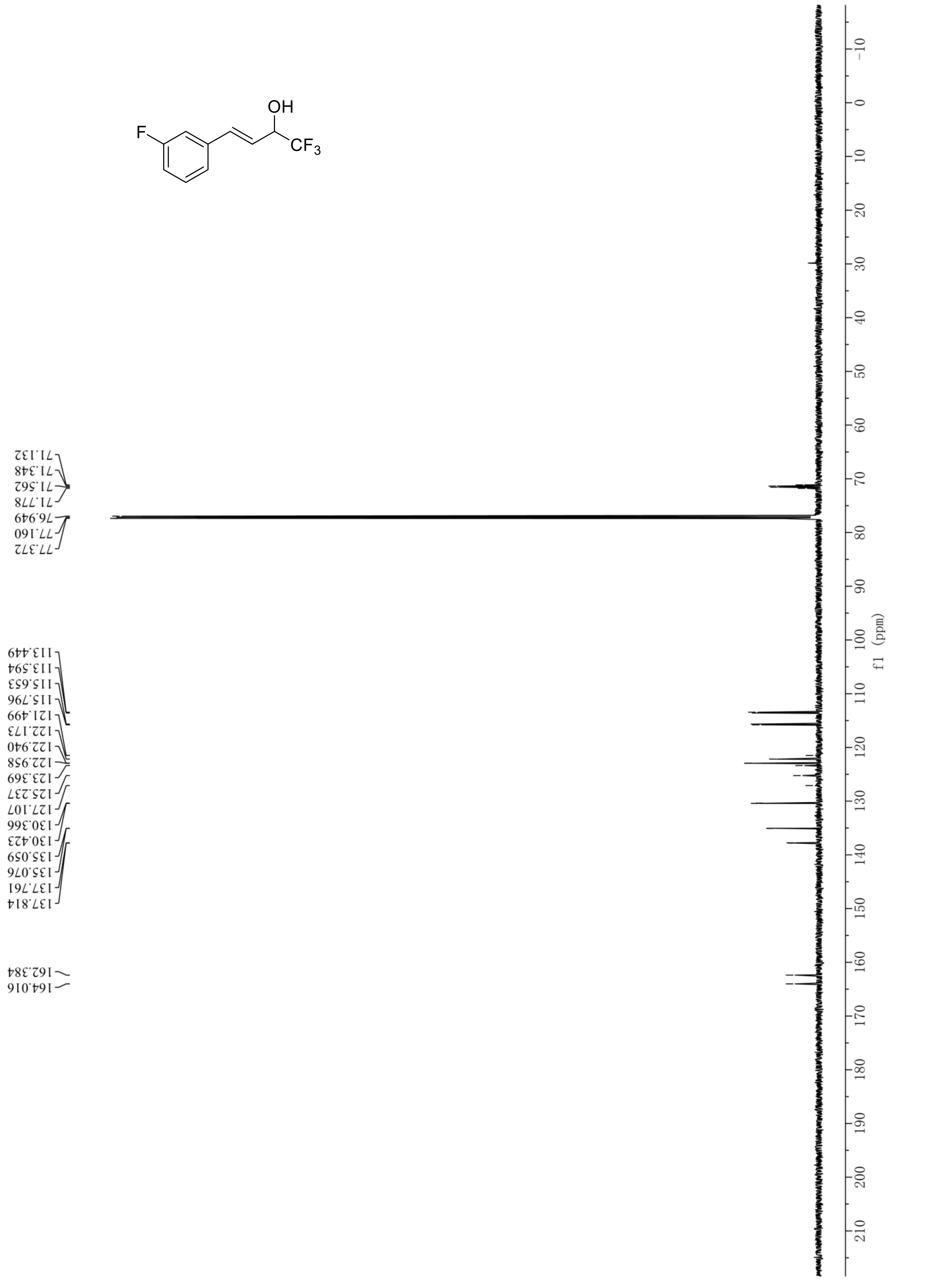

^{19}F NMR of **13b** ($CDCl_3$, 375 MHz, 25 ºC)

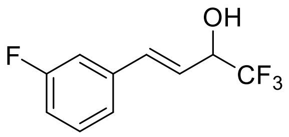

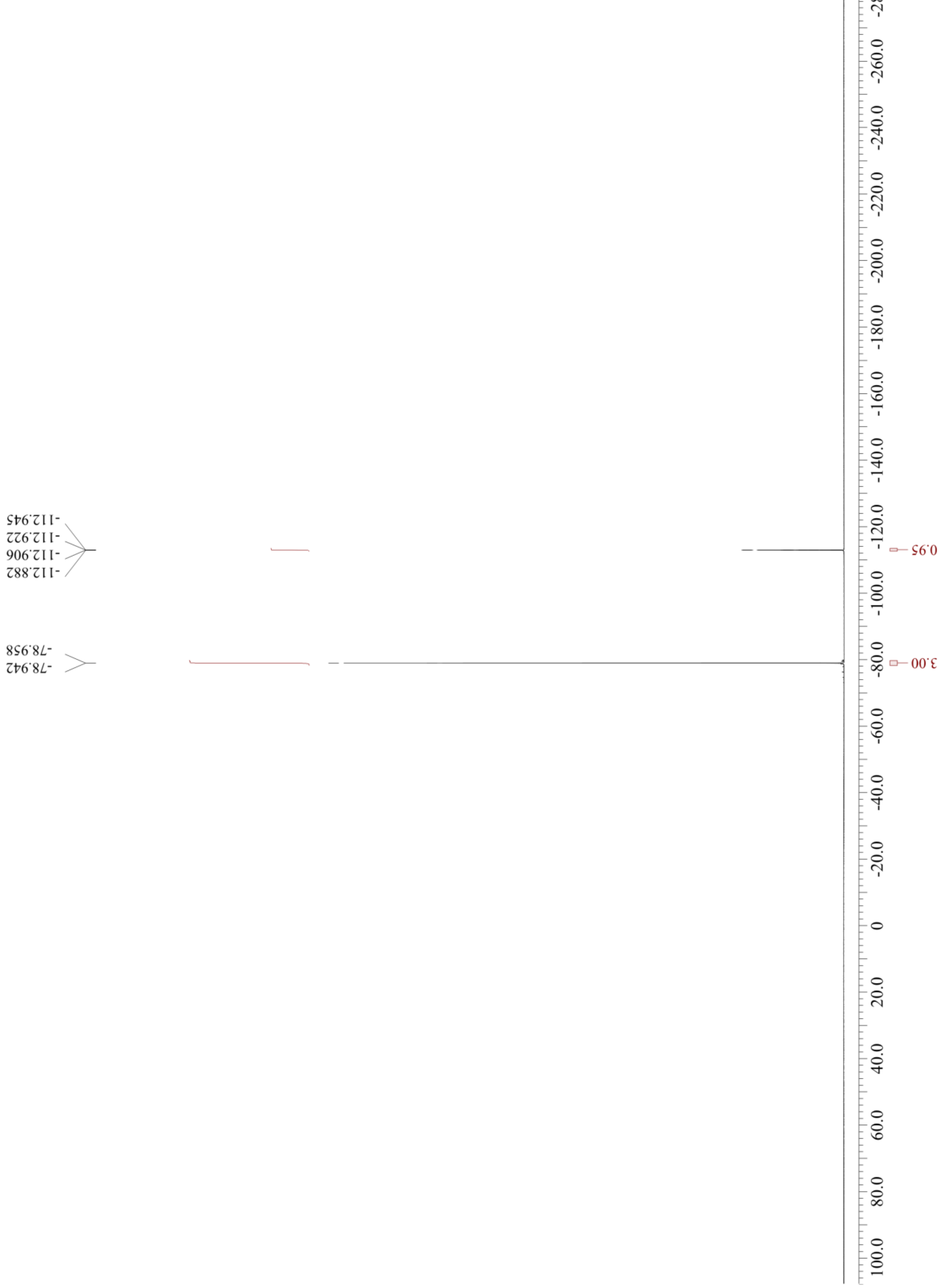

^{1}H NMR of **13c** ($CDCl_3$, 400 MHz, 25 ºC)

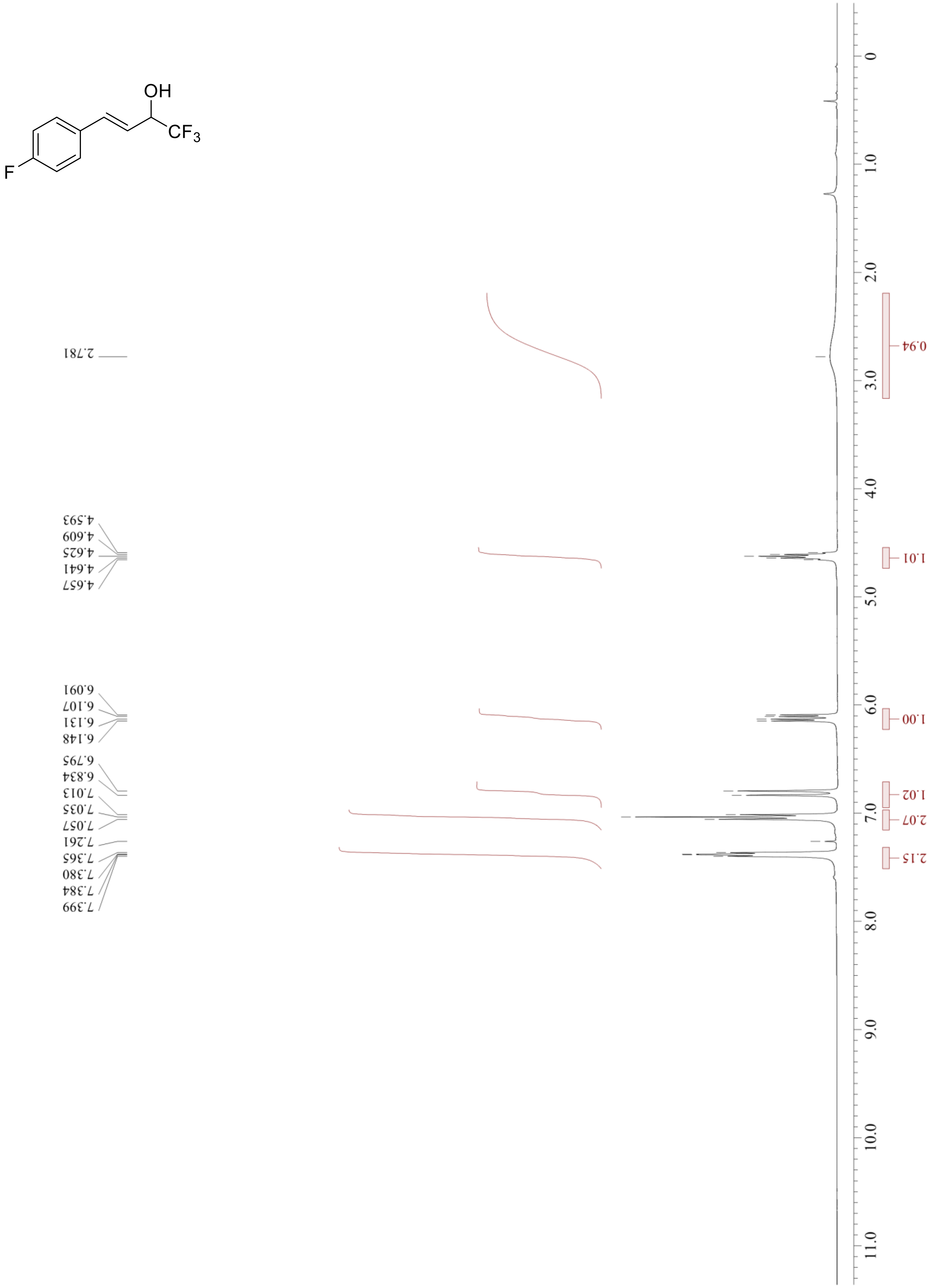

^{13}C NMR of **13c** ($CDCl_3$, 100 MHz, 25 ºC)

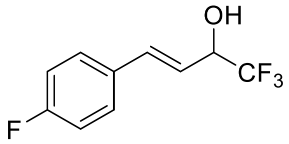

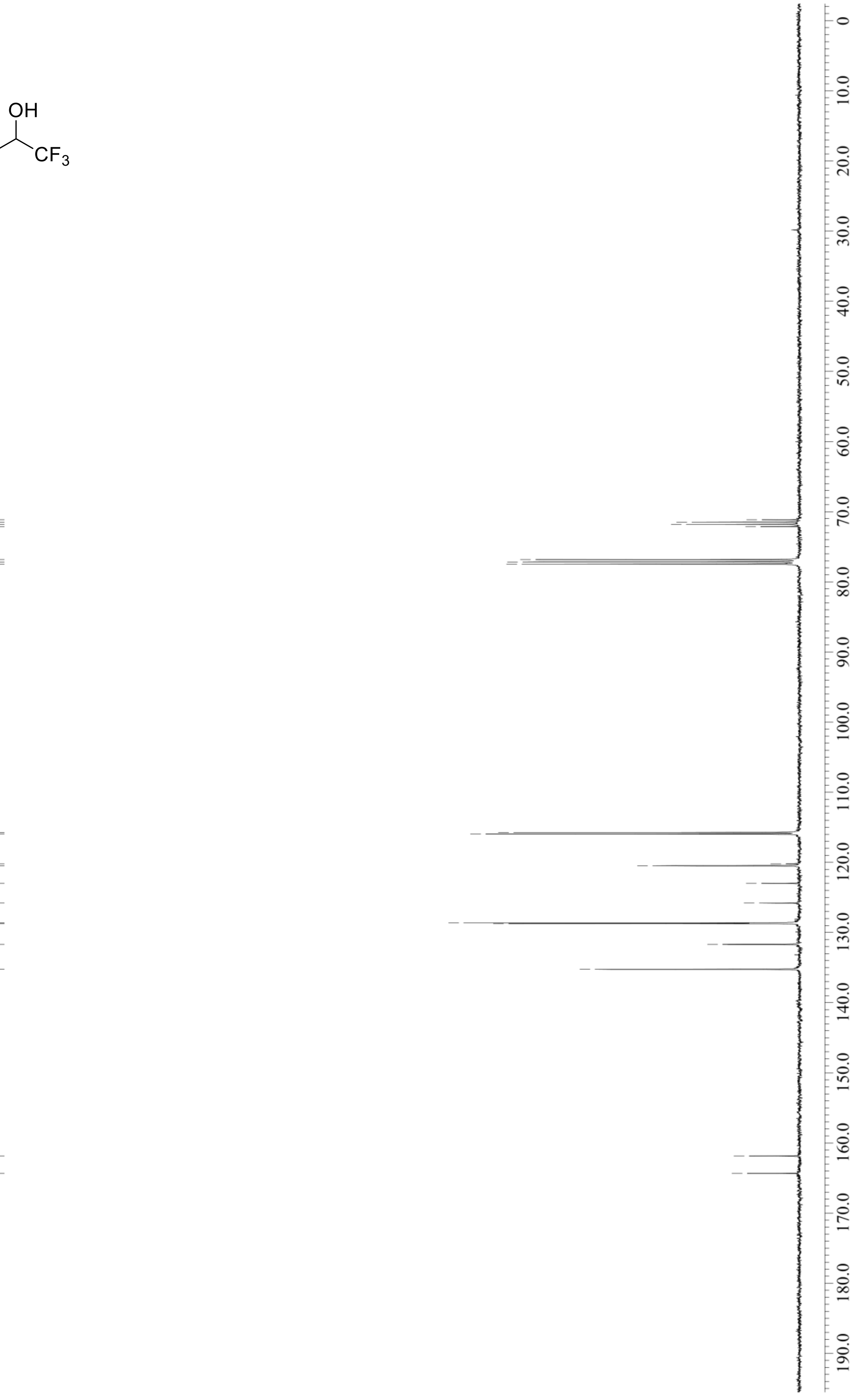

^{19}F NMR of **13c** ($CDCl_3$, 375 MHz, 25 ºC)

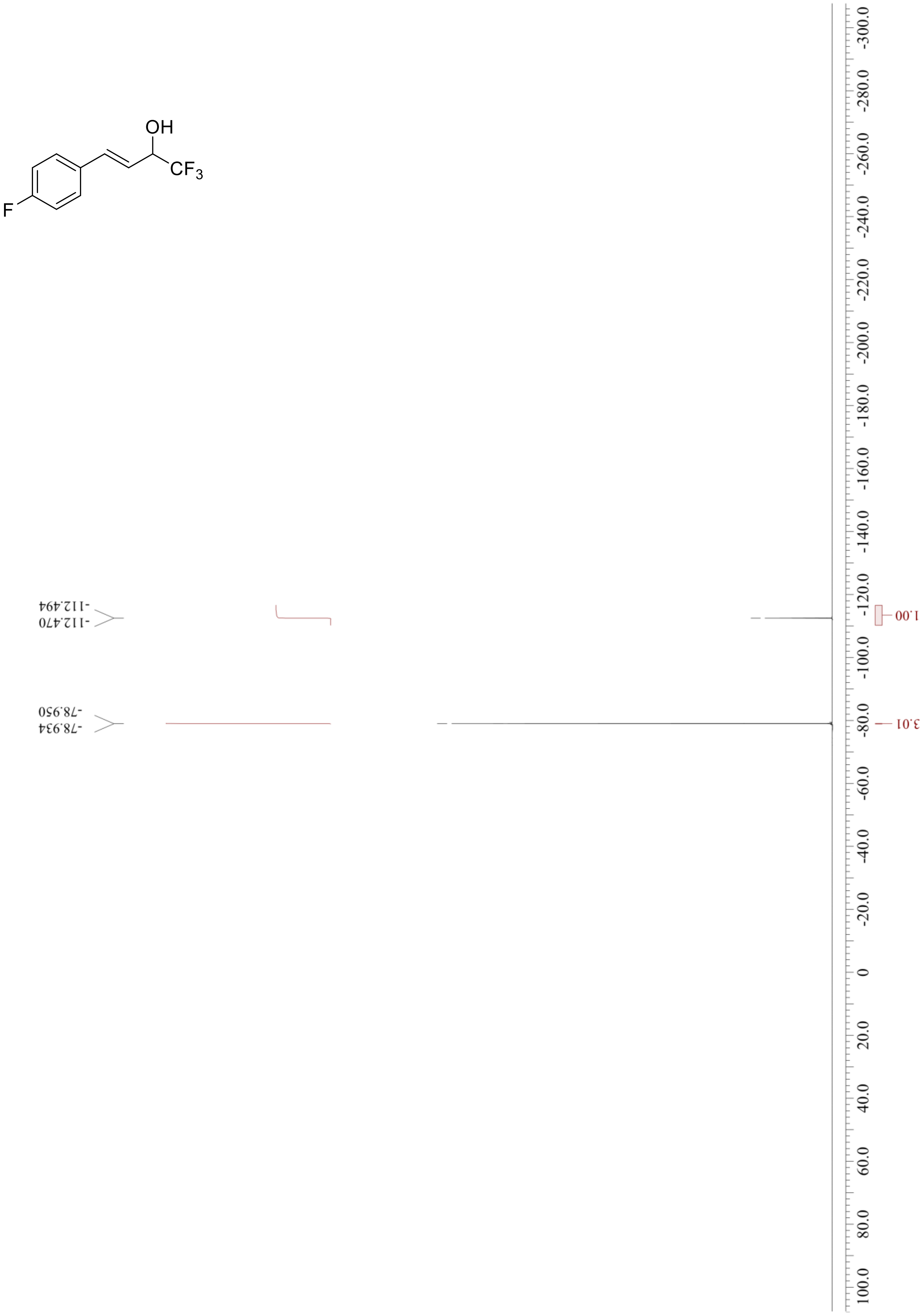

^{1}H NMR of **13d** ($CDCl_3$, 400 MHz, 25 ºC)

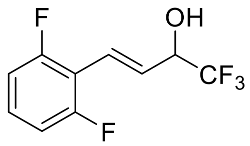

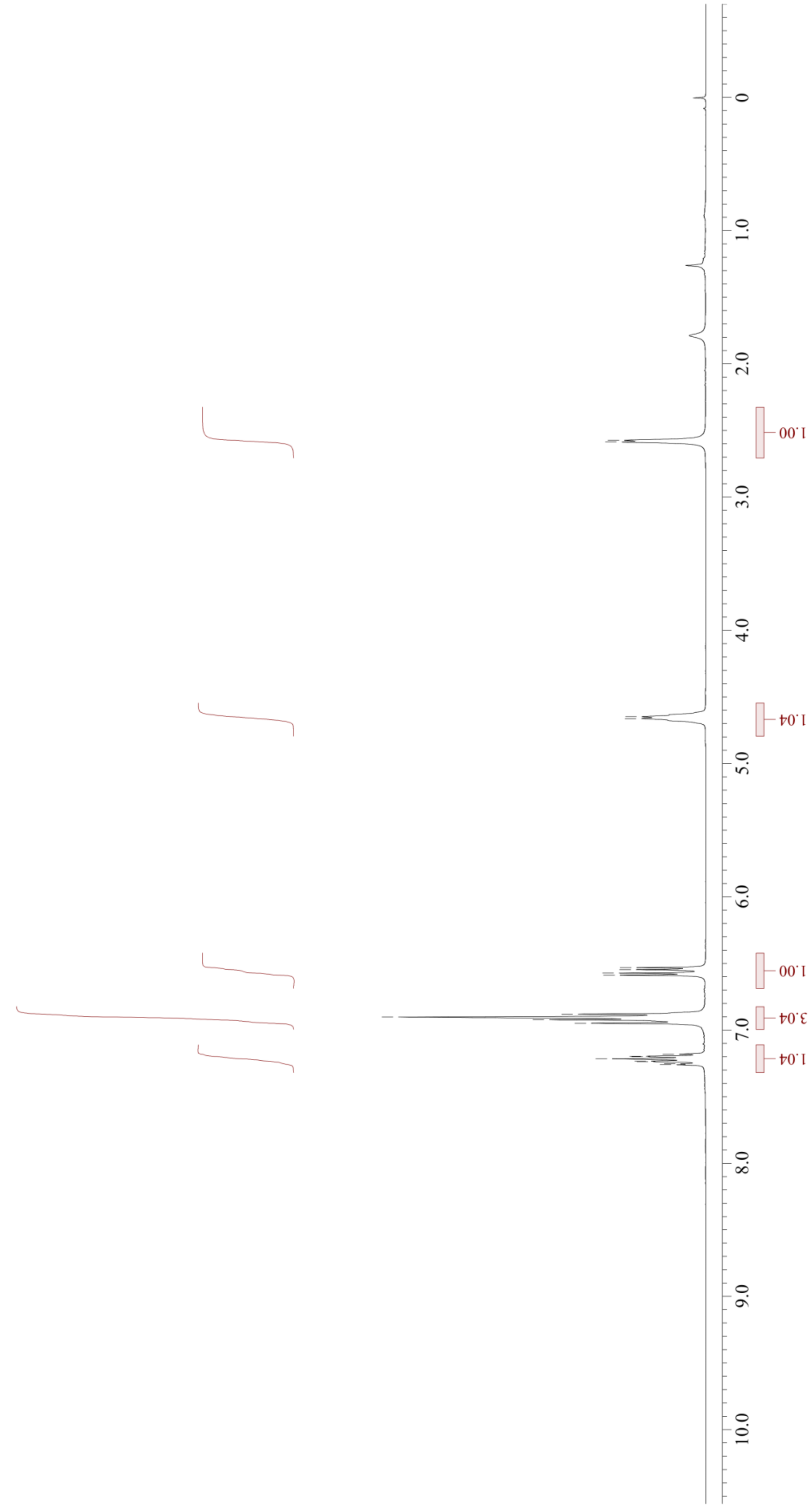

^{13}C NMR of **13d** ($CDCl_3$, 100 MHz, 25 ºC)

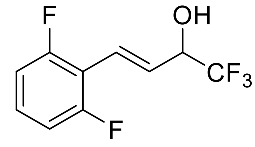

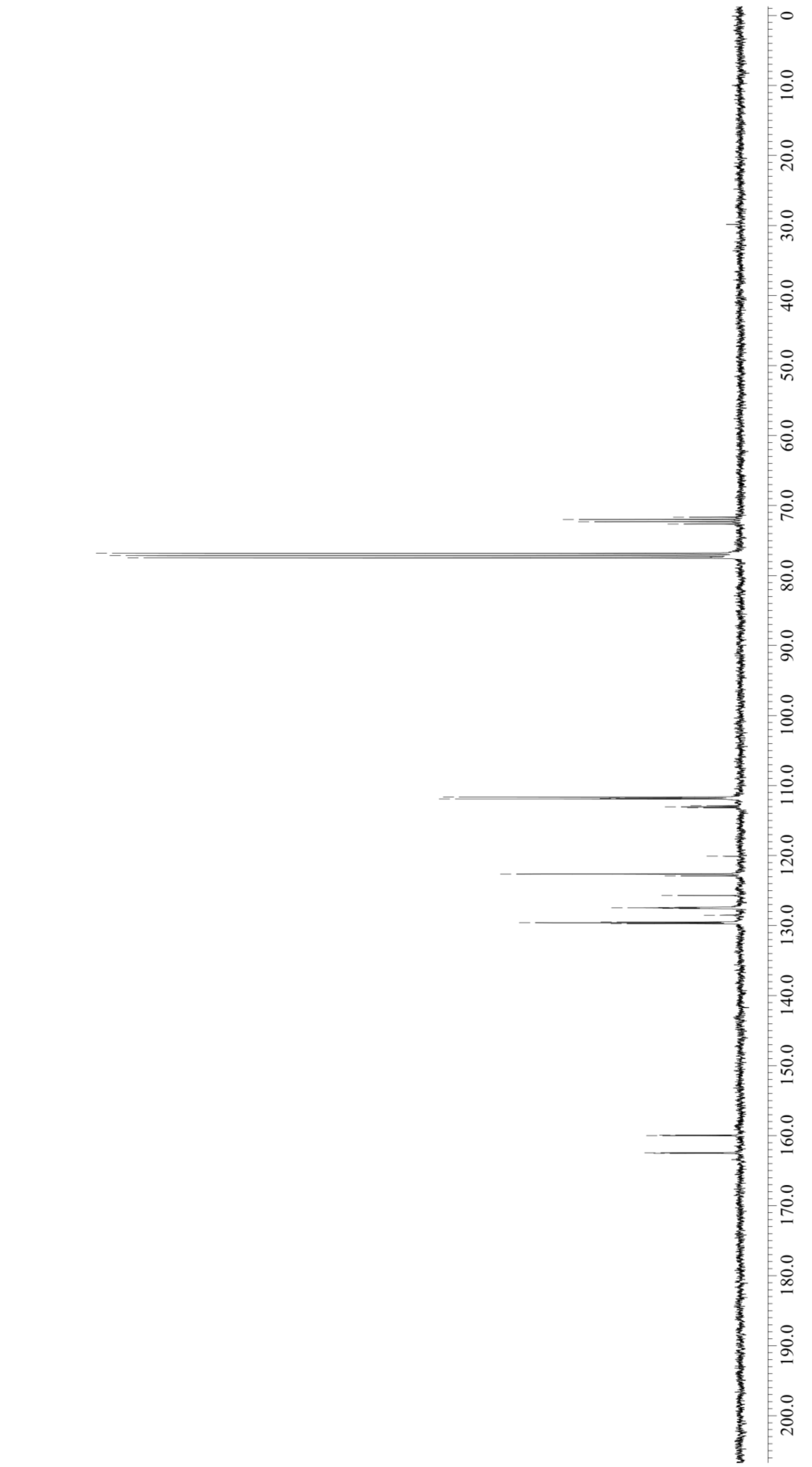

^{19}F NMR of **13d** ($CDCl_3$, 375 MHz, 25 ºC)

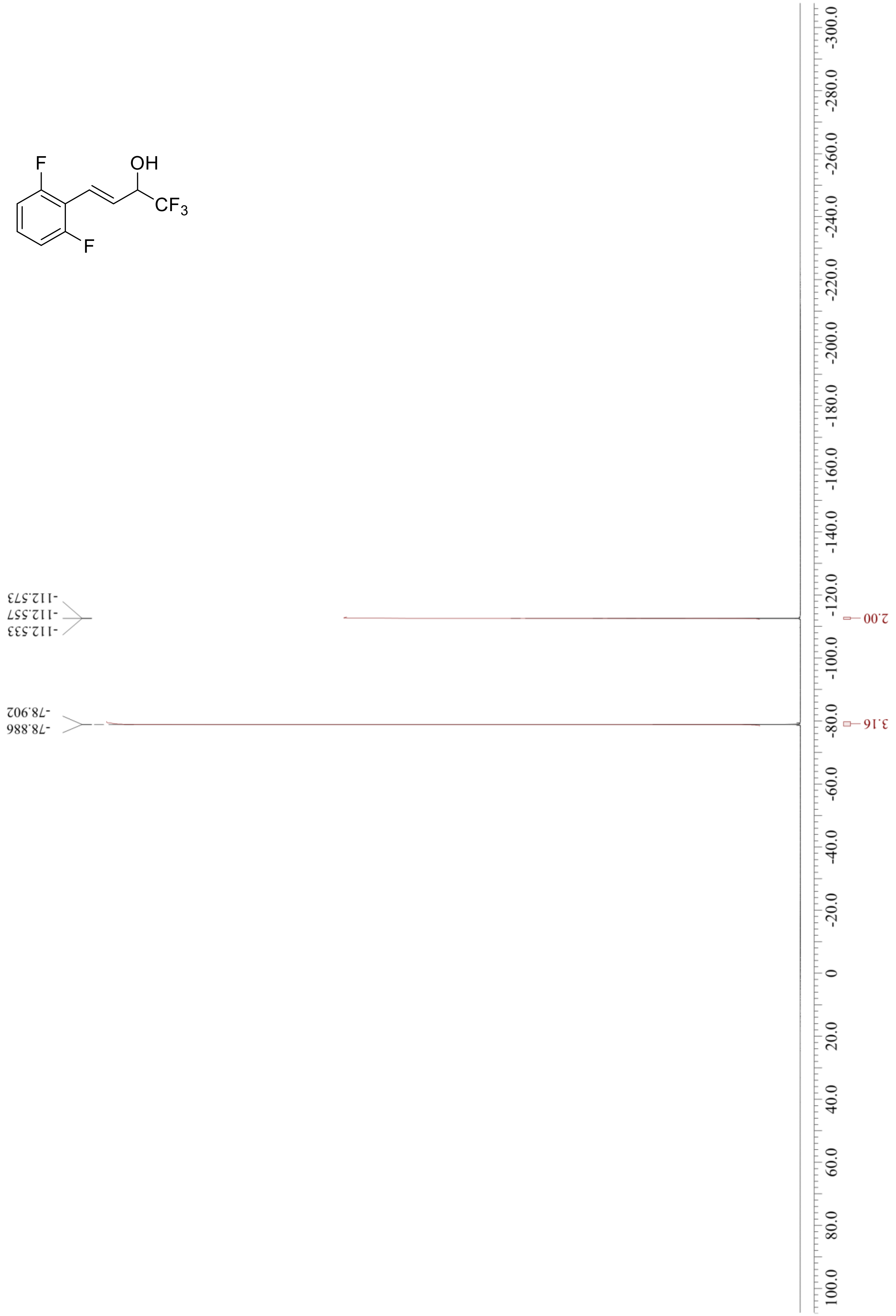

^{1}H NMR of **13e** ($CDCl_3$, 600 MHz, 25 ºC)

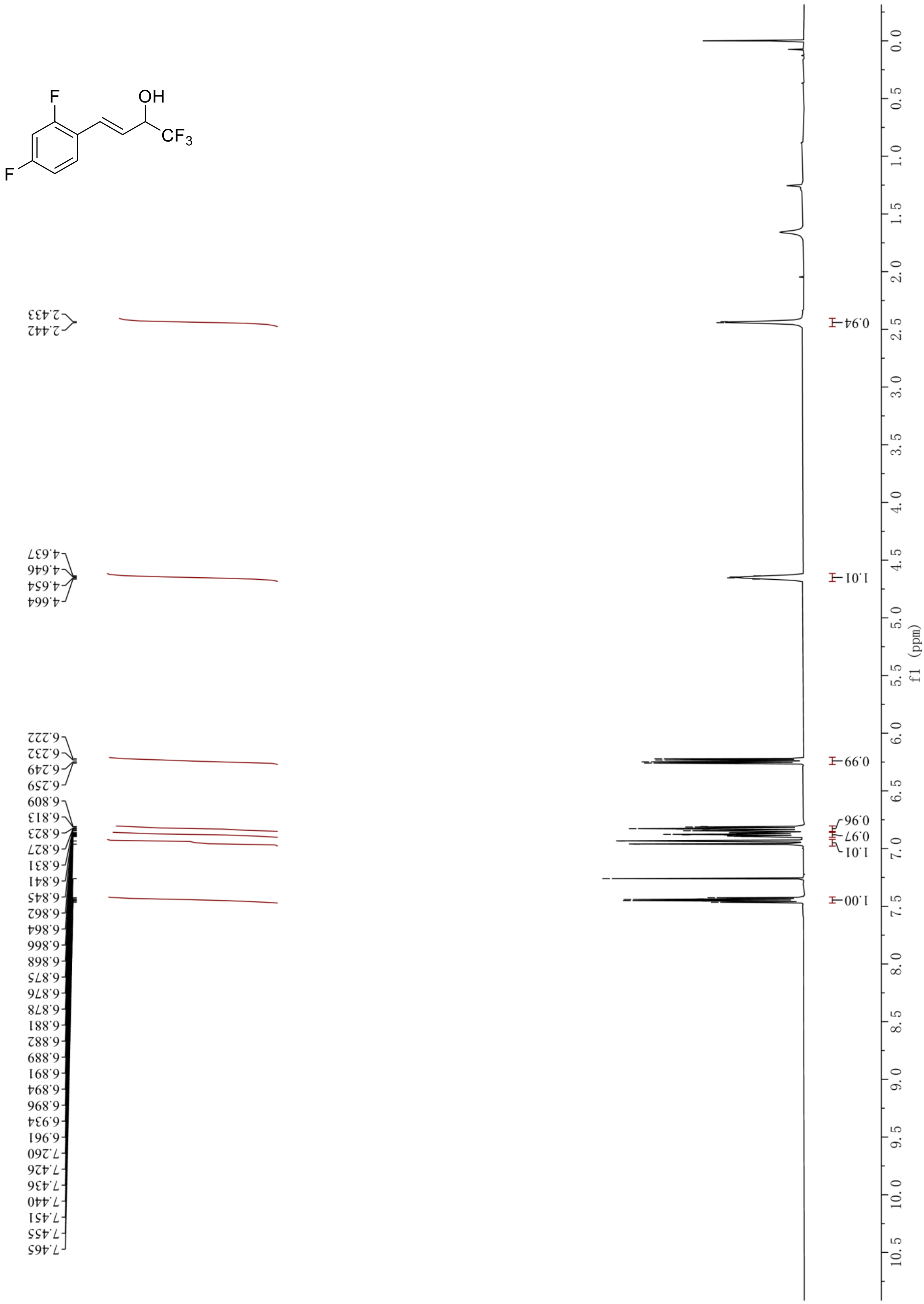

^{13}C NMR of **13e** ($CDCl_3$, 150 MHz, 25 ºC)

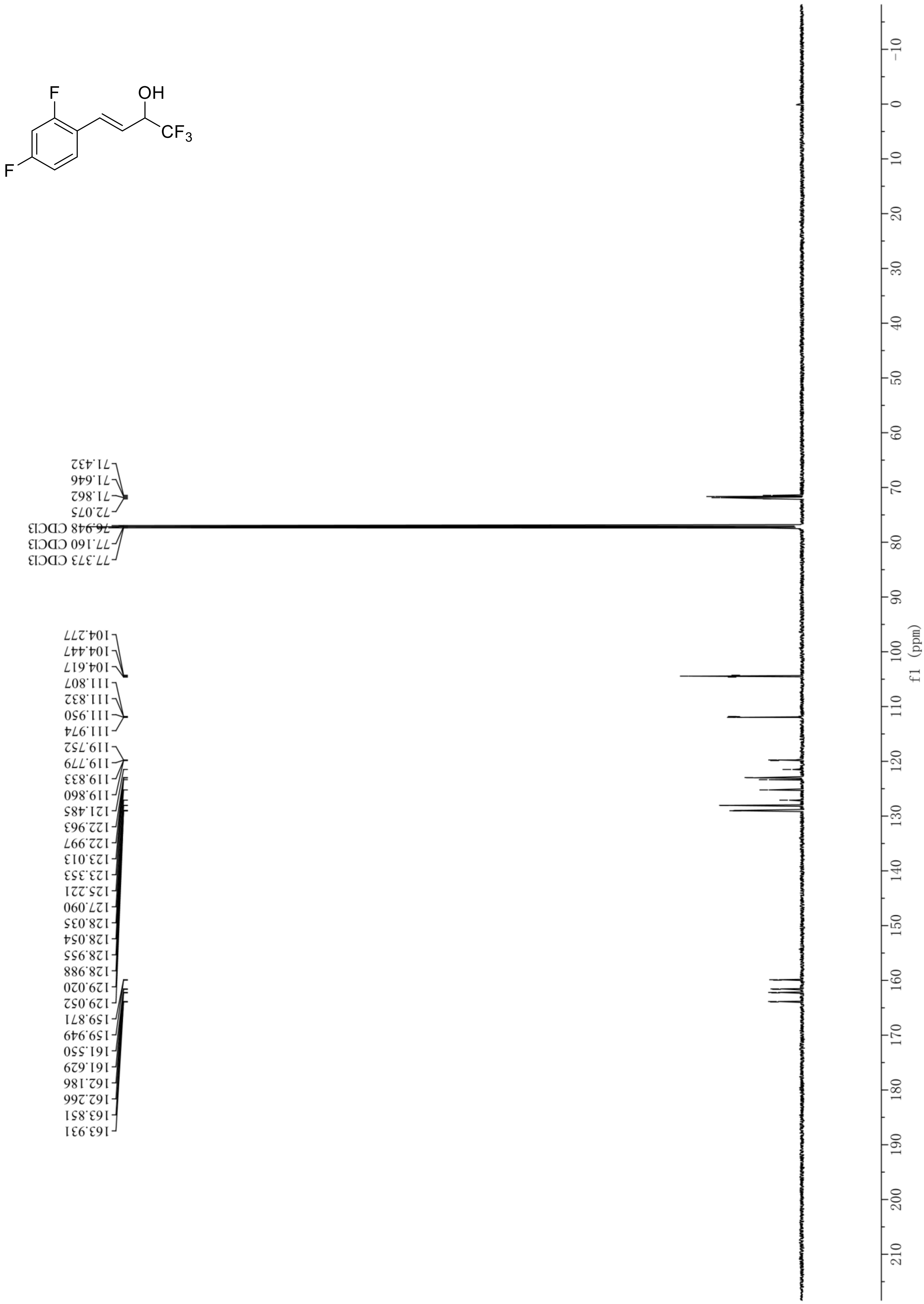

^{19}F NMR of **13e** ($CDCl_3$, 375 MHz, 25 ºC)

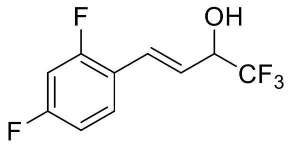

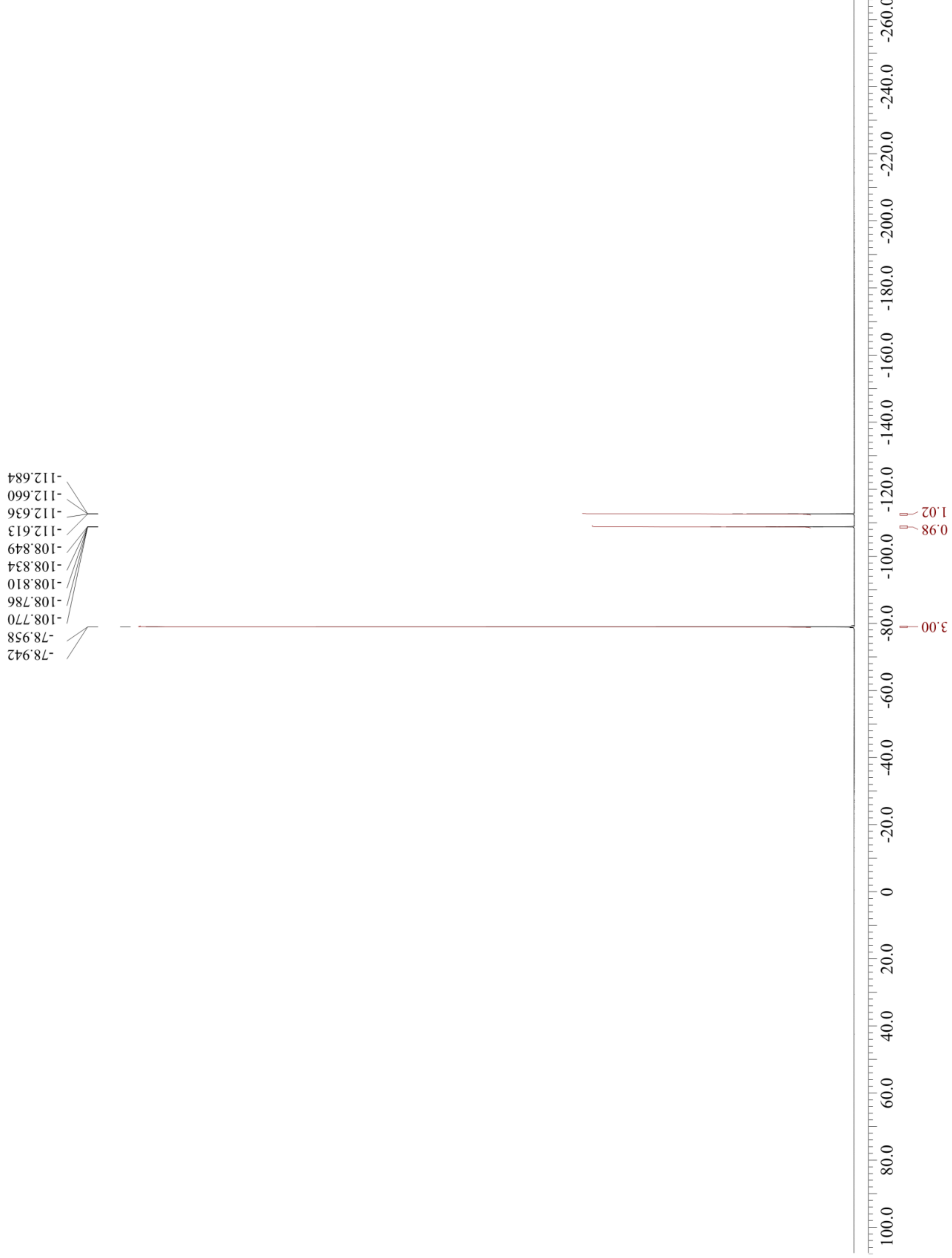

^{1}H NMR of **13f** ($CDCl_3$, 600 MHz, 25 ºC)

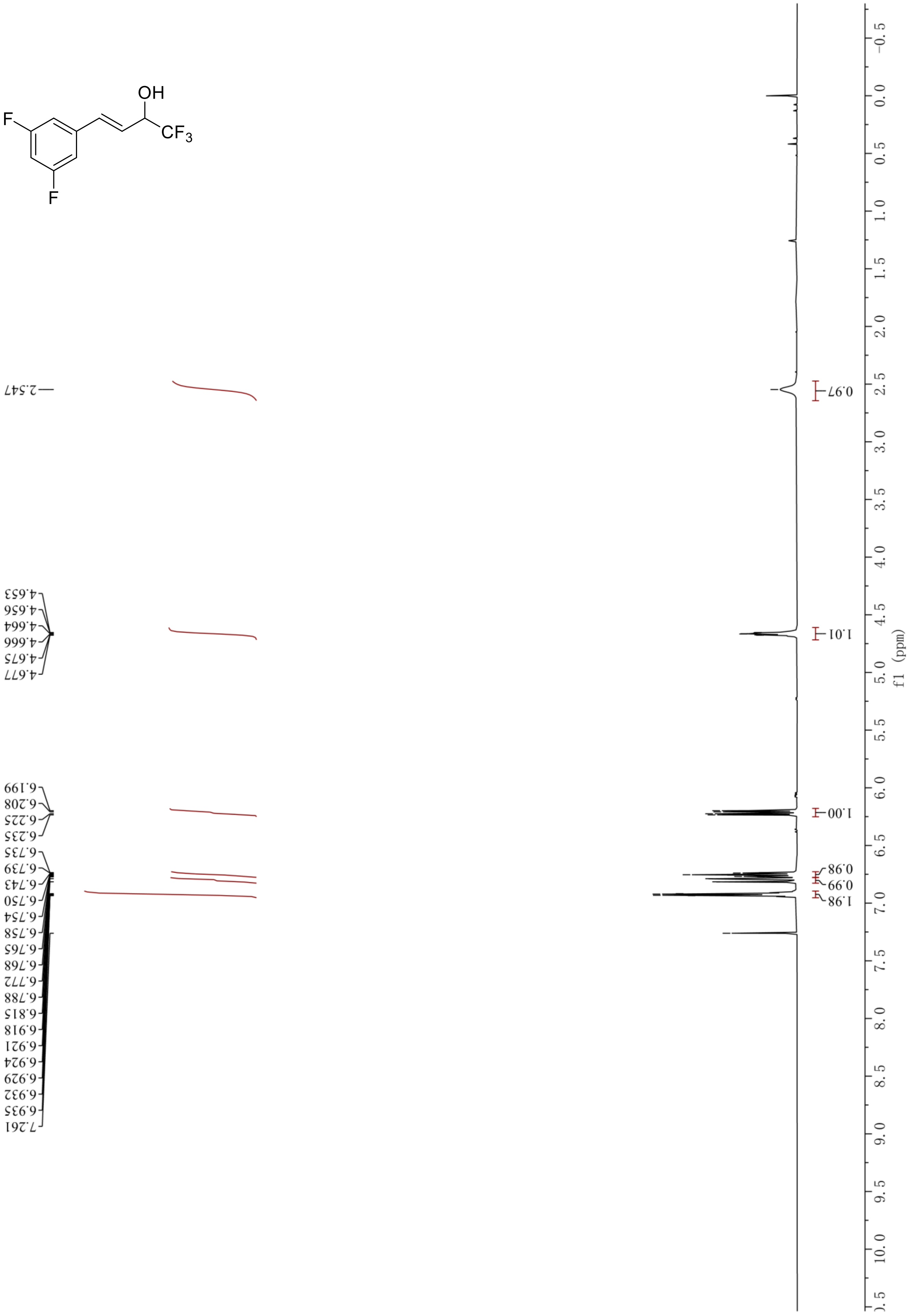

^{13}C NMR of **13f** ($CDCl_3$, 150 MHz, 25 ºC)

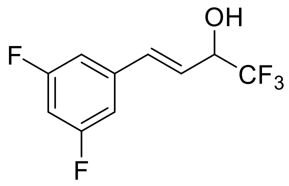

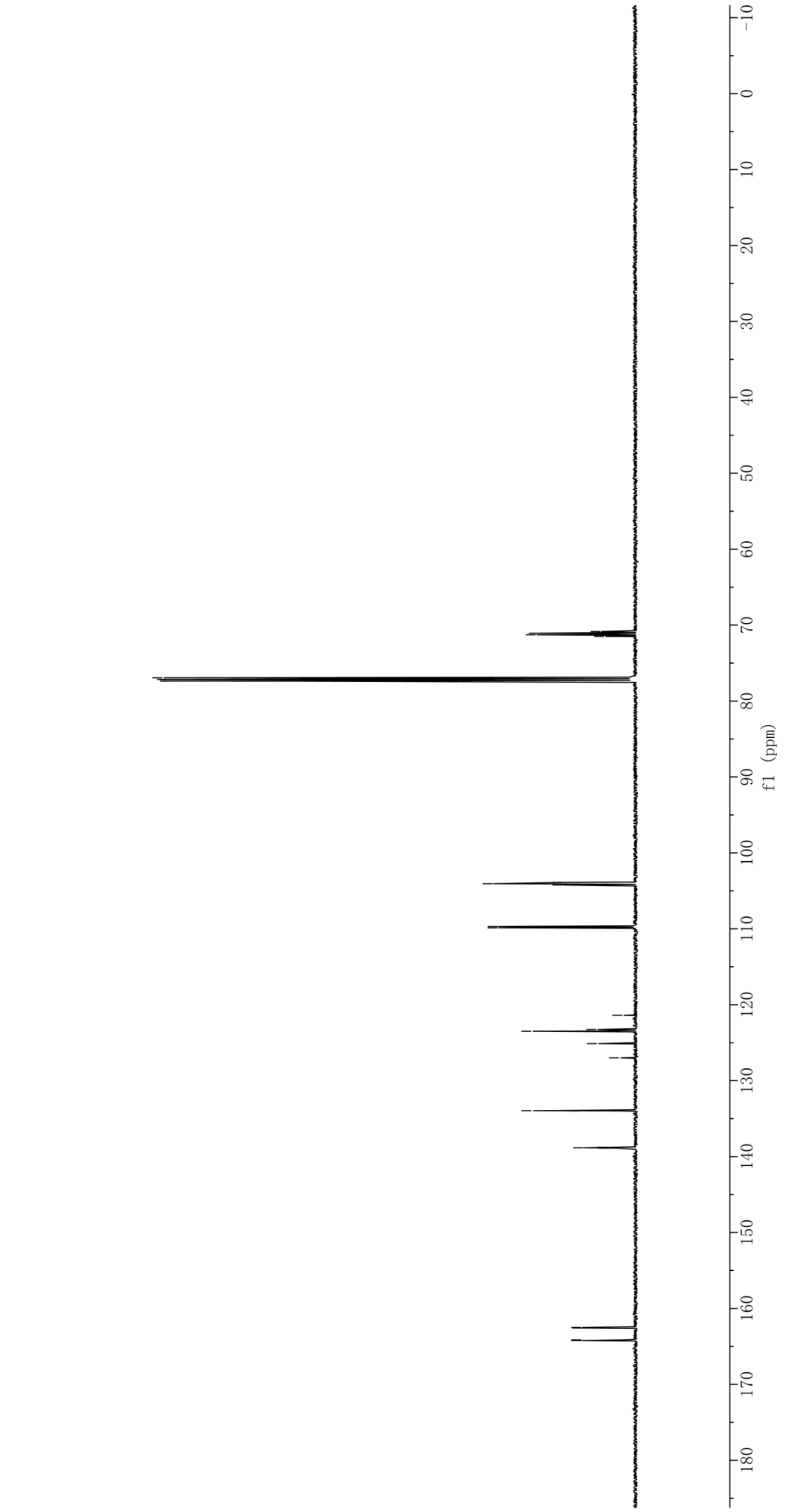

^{19}F NMR of **13f** ($CDCl_3$, 375 MHz, 25 ºC)

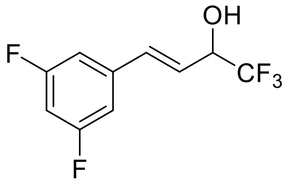

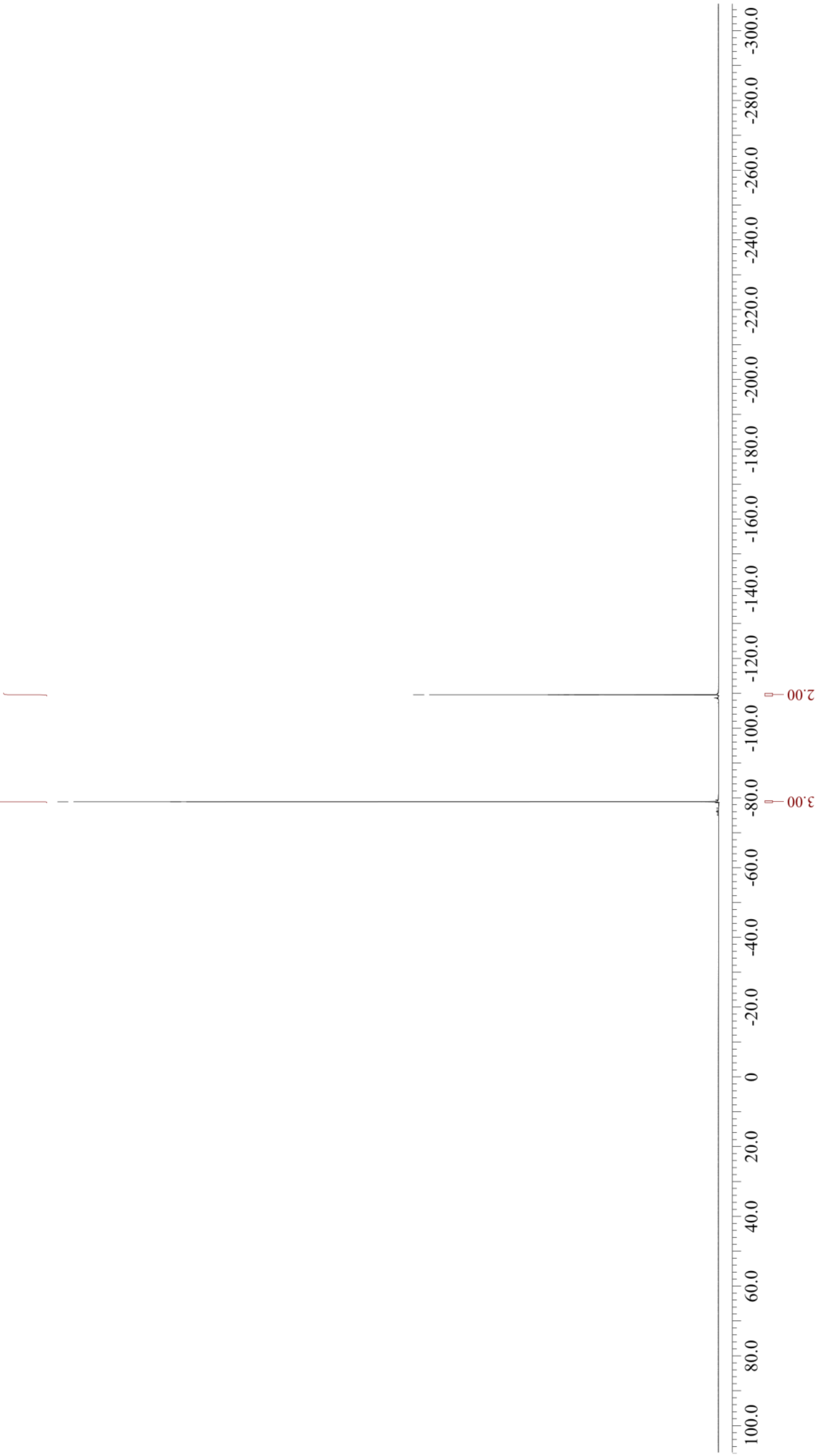

^{1}H NMR of **13g** ($CDCl_3$, 400 MHz, 25 ºC)

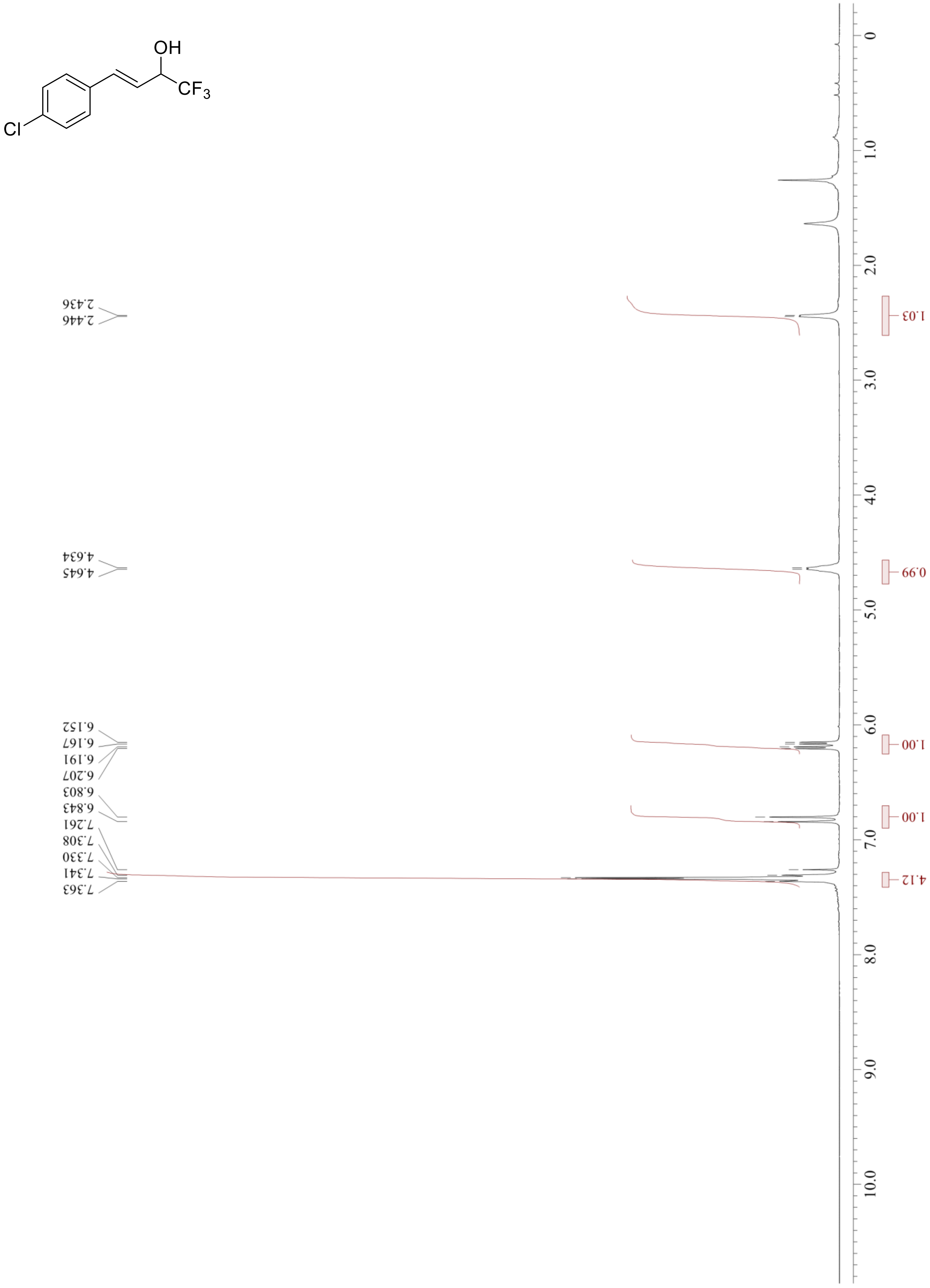

^{13}C NMR of **13g** ($CDCl_3$, 100 MHz, 25 ºC)

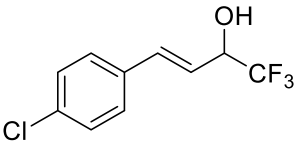

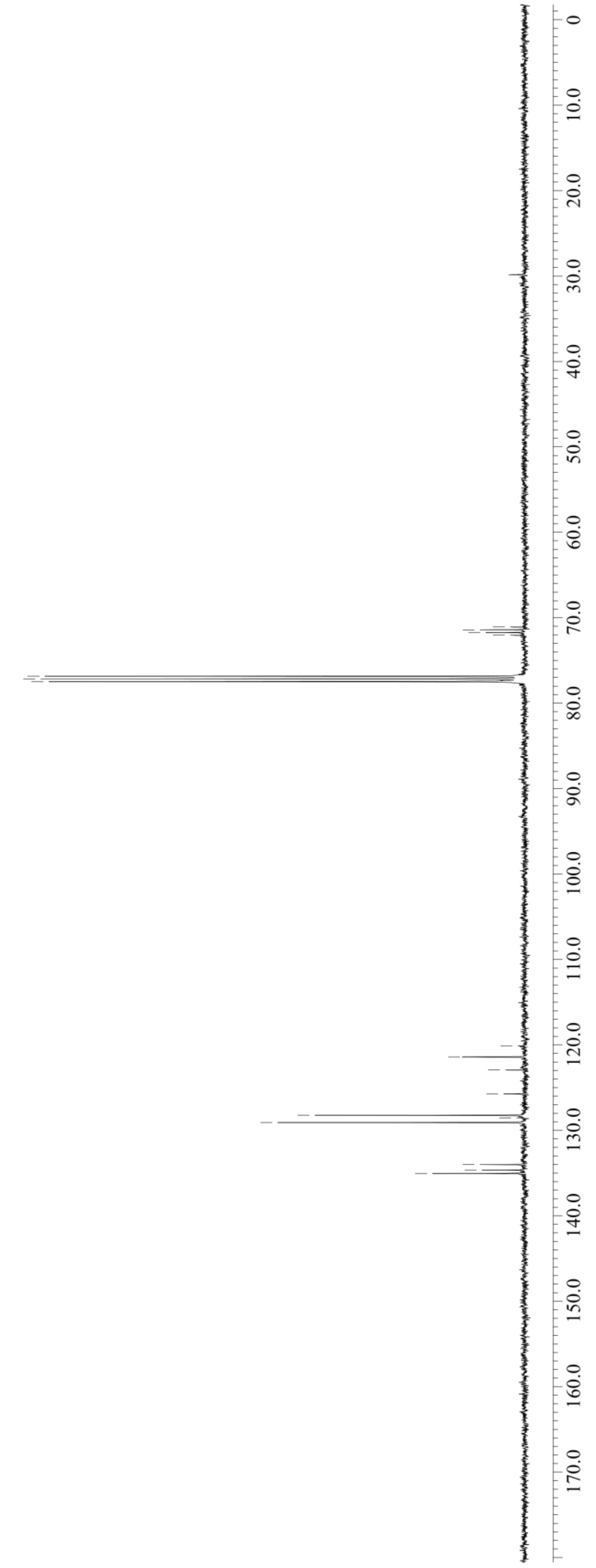

^{19}F NMR of **13g** ($CDCl_3$, 375 MHz, 25 ºC)

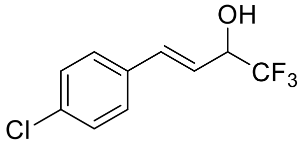

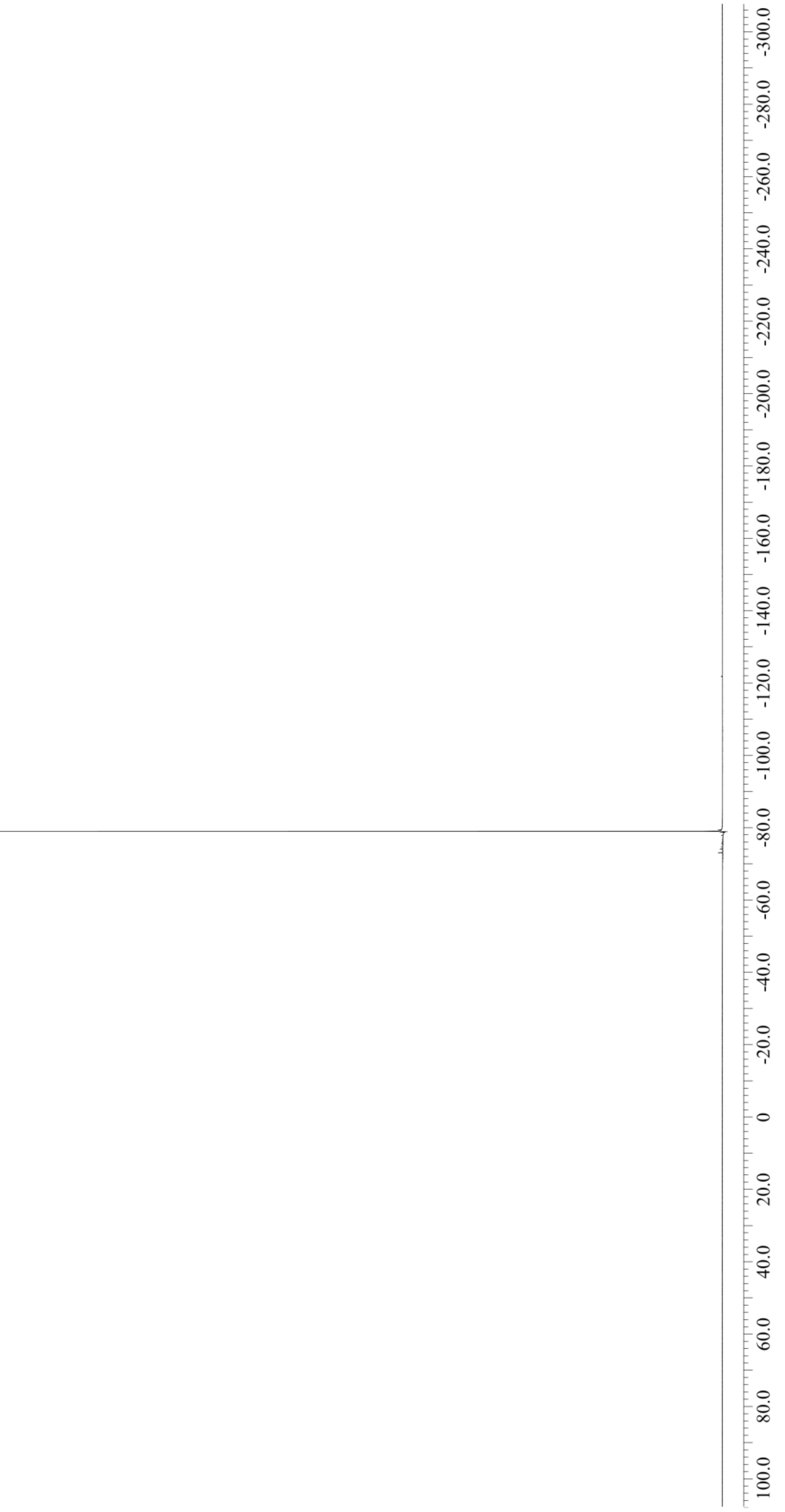

^{1}H NMR of **13h** ($CDCl_3$, 400 MHz, 25 °C)

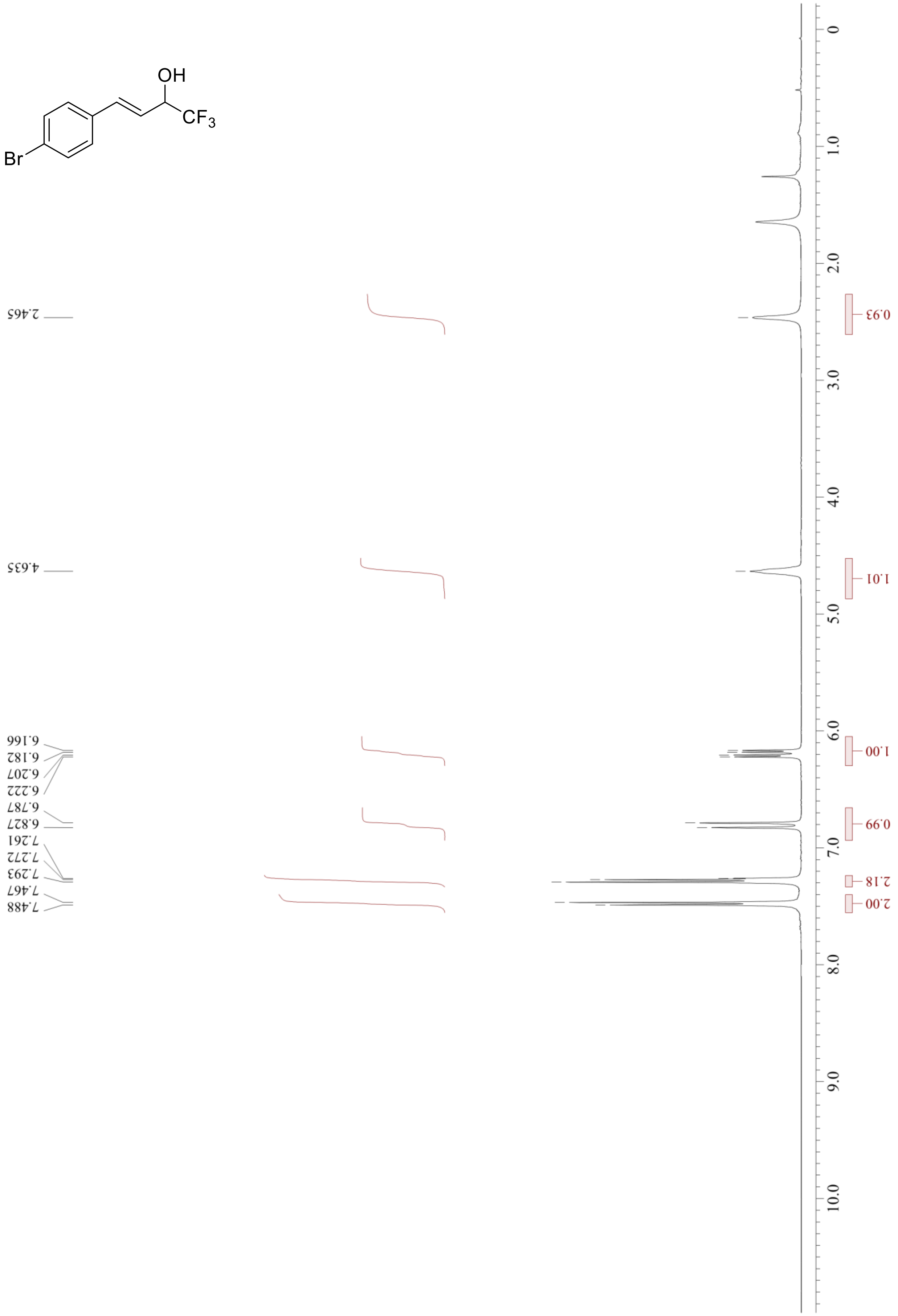

^{13}C NMR of **13h** ($CDCl_3$, 100 MHz, 25 ºC)

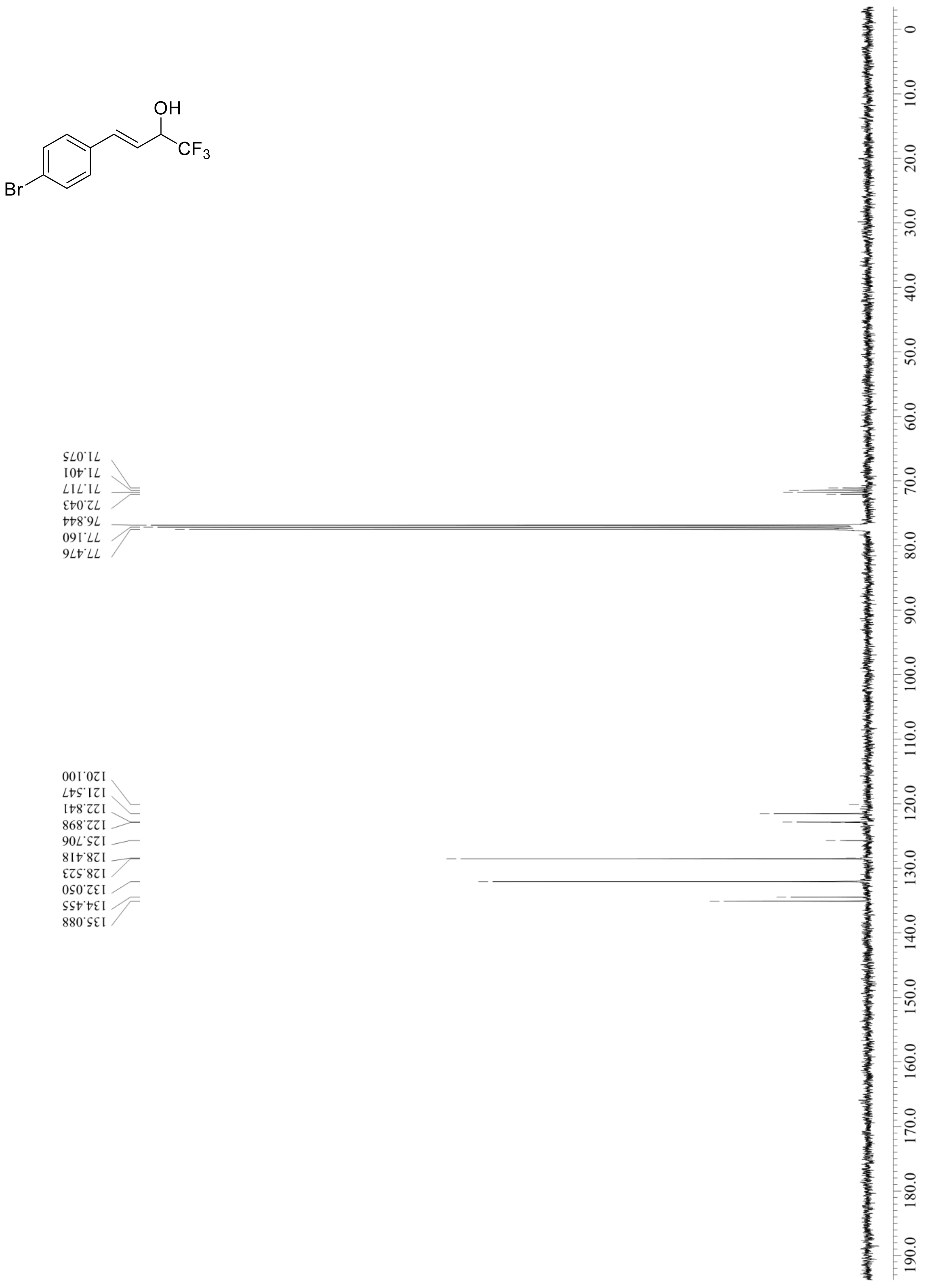

^{19}F NMR of **13h** ($CDCl_3$, 375 MHz, 25 ºC)

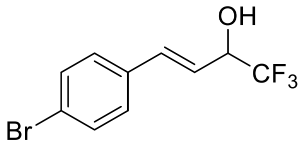

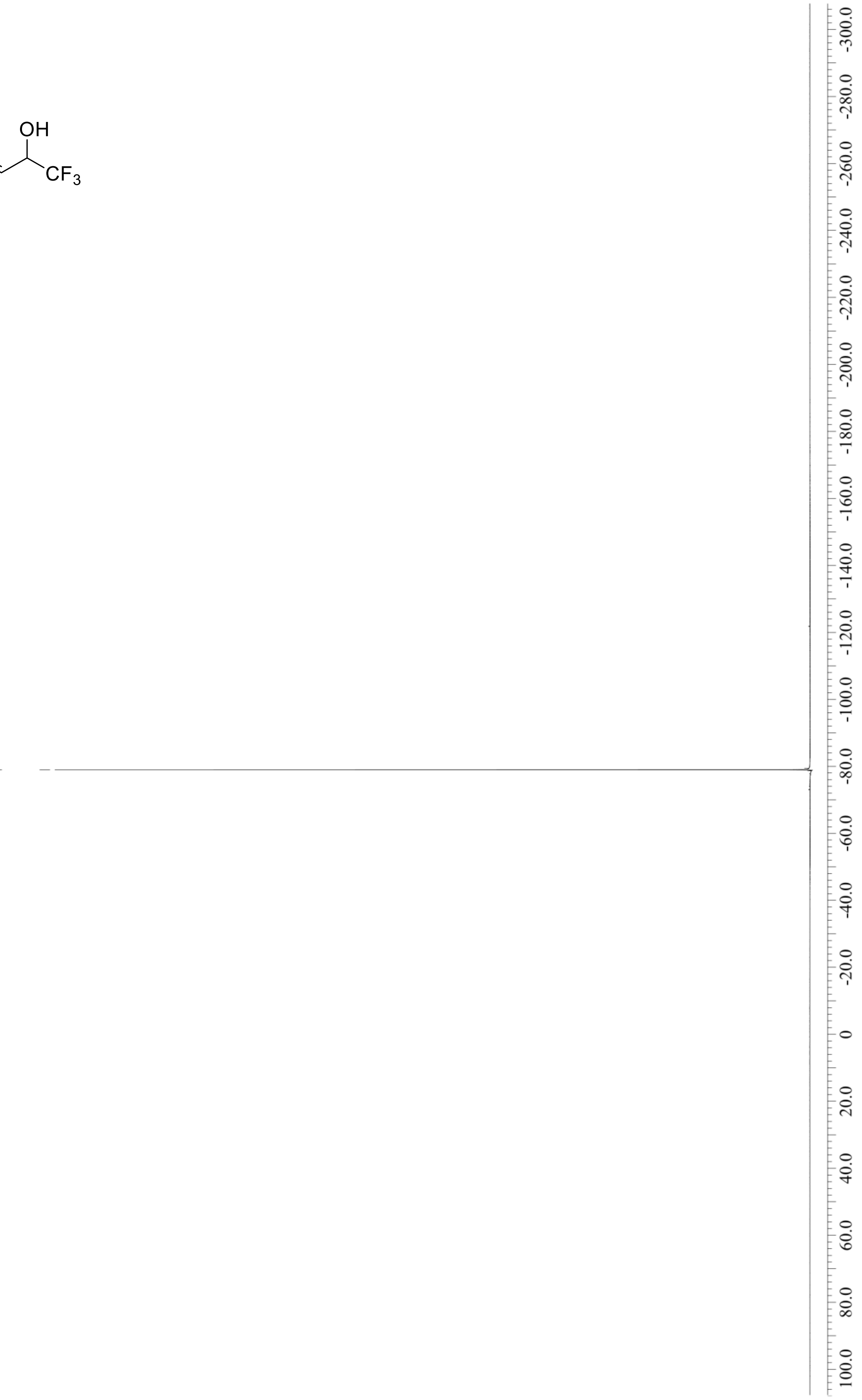

^{1}H NMR of **13i** ($CDCl_3$, 400 MHz, 25 ºC)

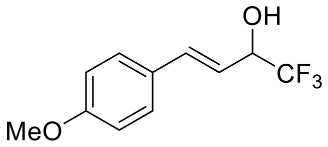

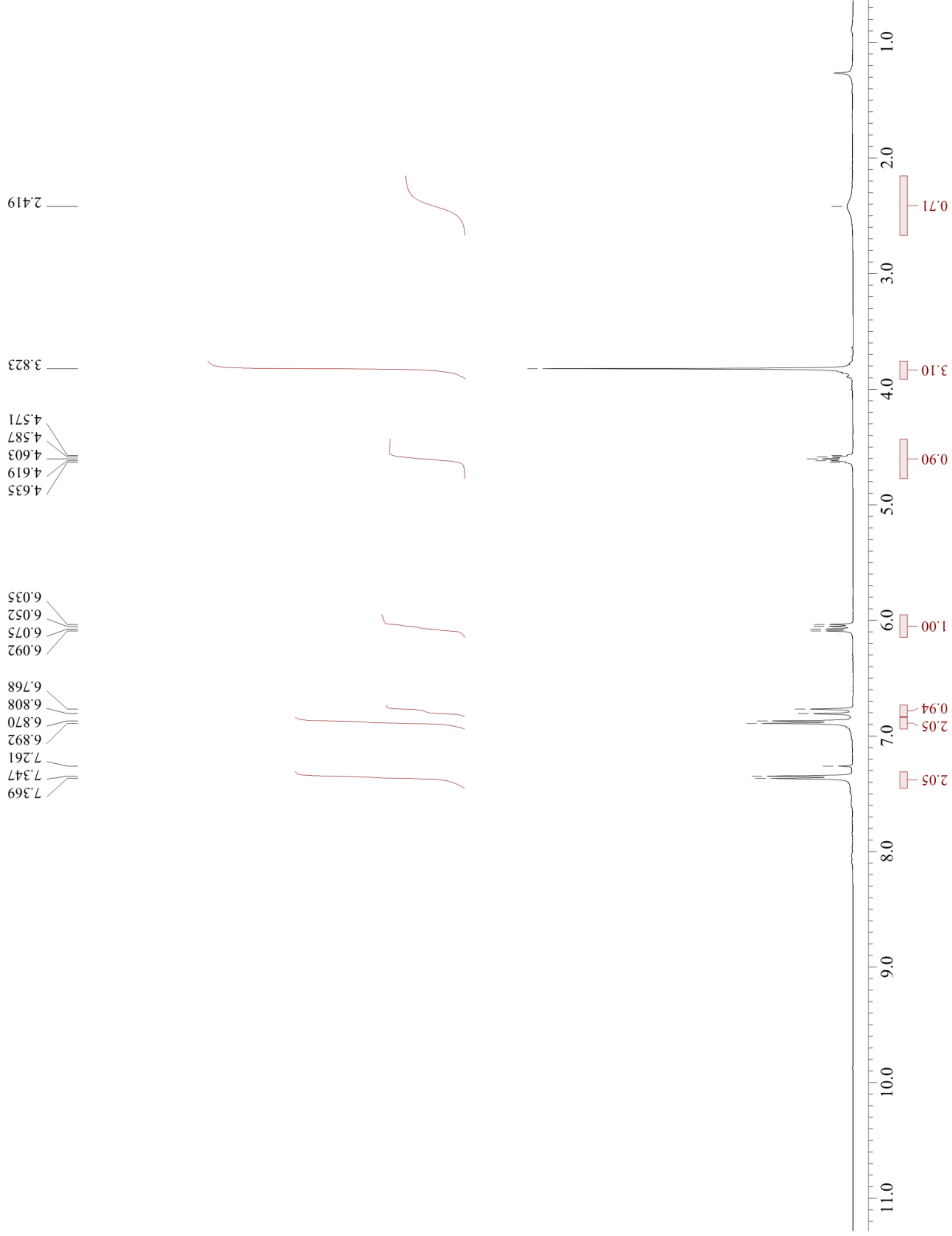

^{13}C NMR of **13i** ($CDCl_3$, 100 MHz, 25 ºC)

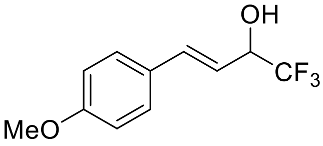

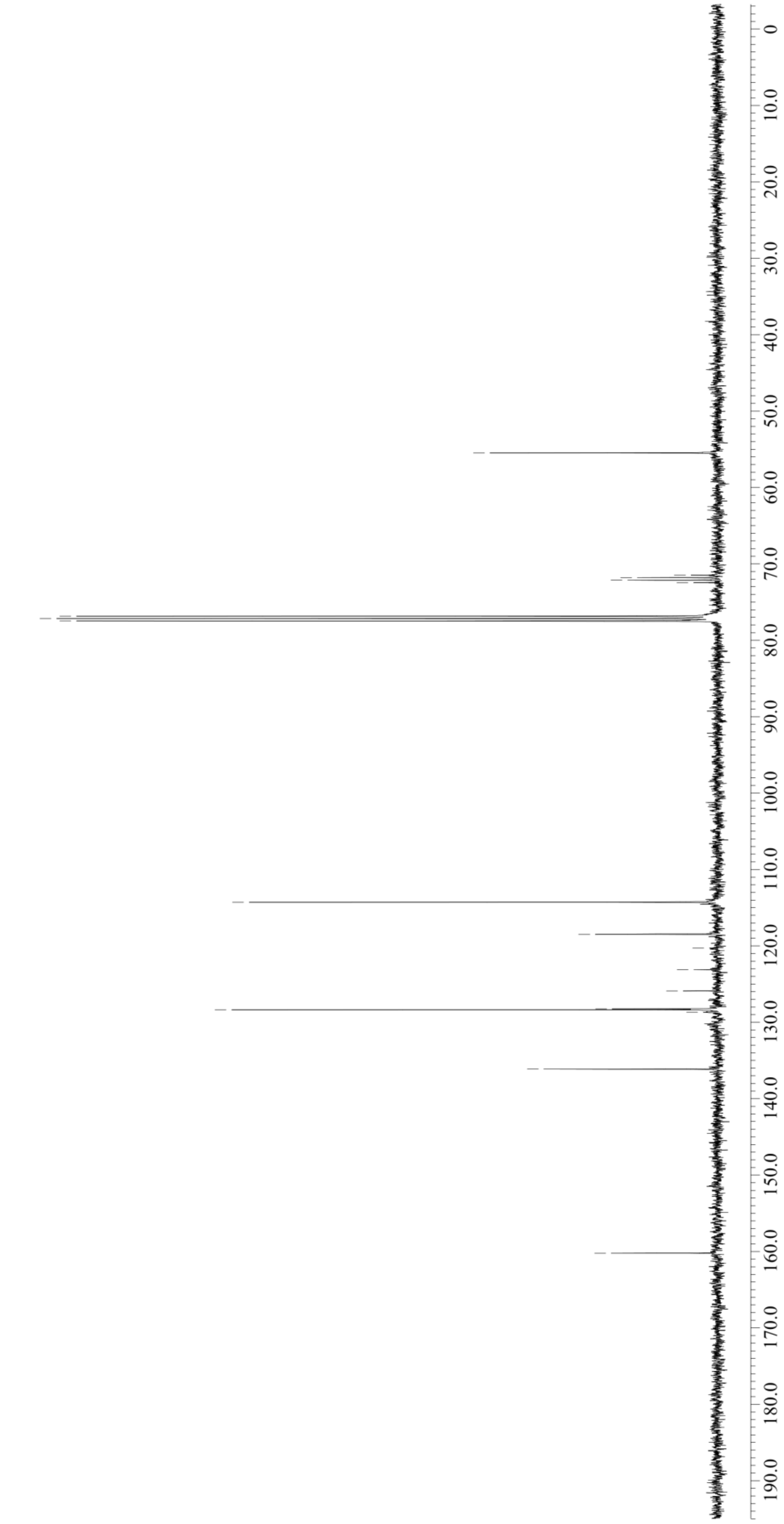

^{19}F NMR of **13i** ($CDCl_3$, 375 MHz, 25 ºC)

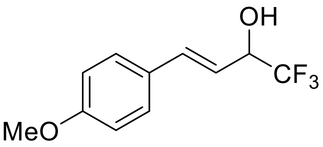

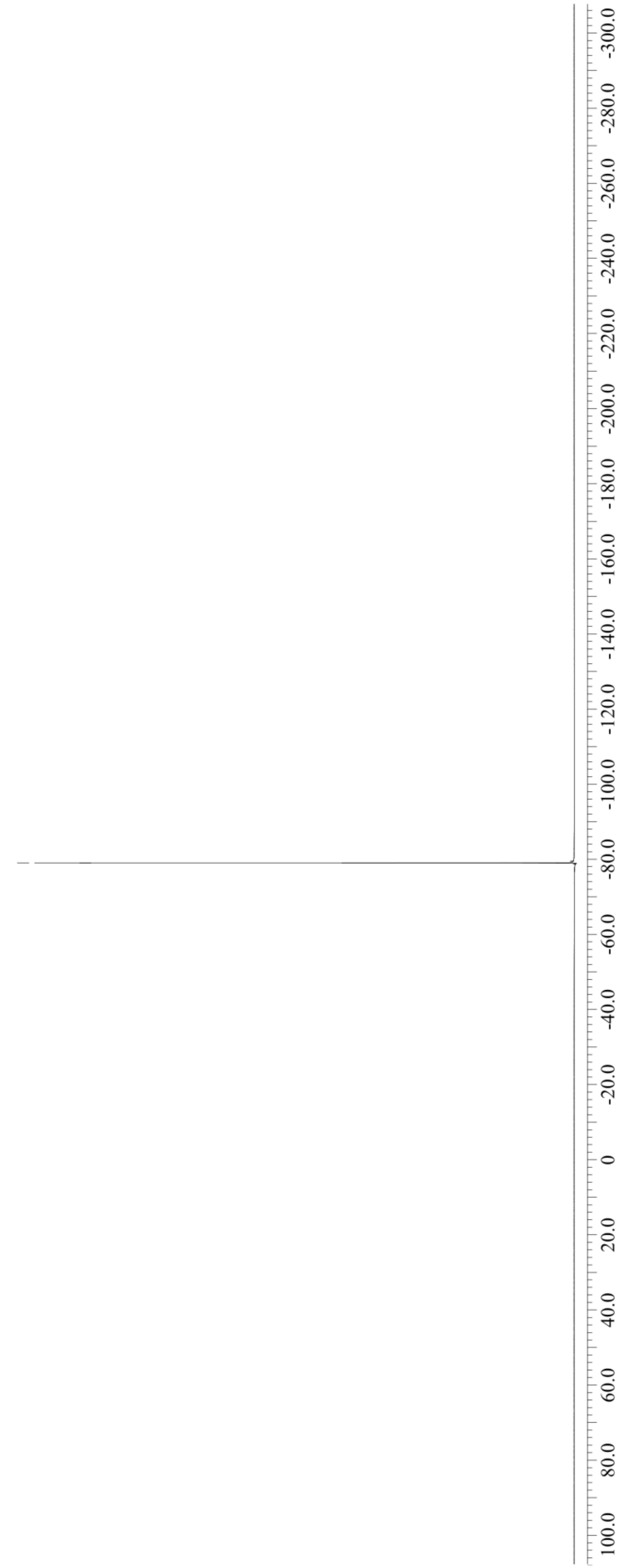

^{1}H NMR of **13j** ($CDCl_3$, 400 MHz, 25 ºC)

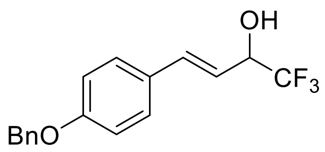

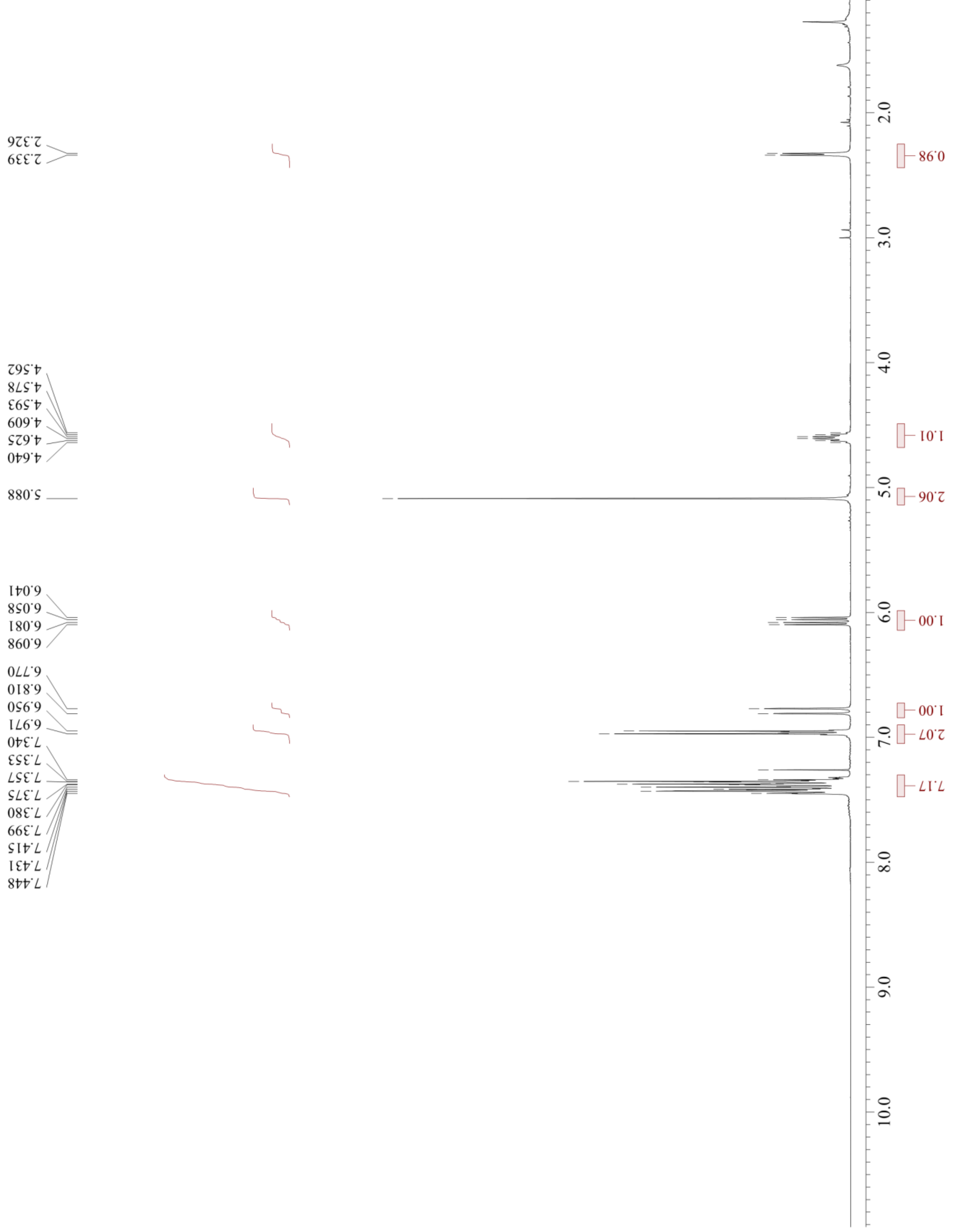

^{13}C NMR of **13j** ($CDCl_3$, 100 MHz, 25 ºC)

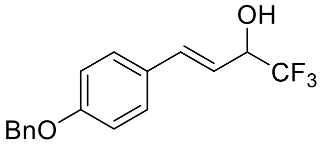

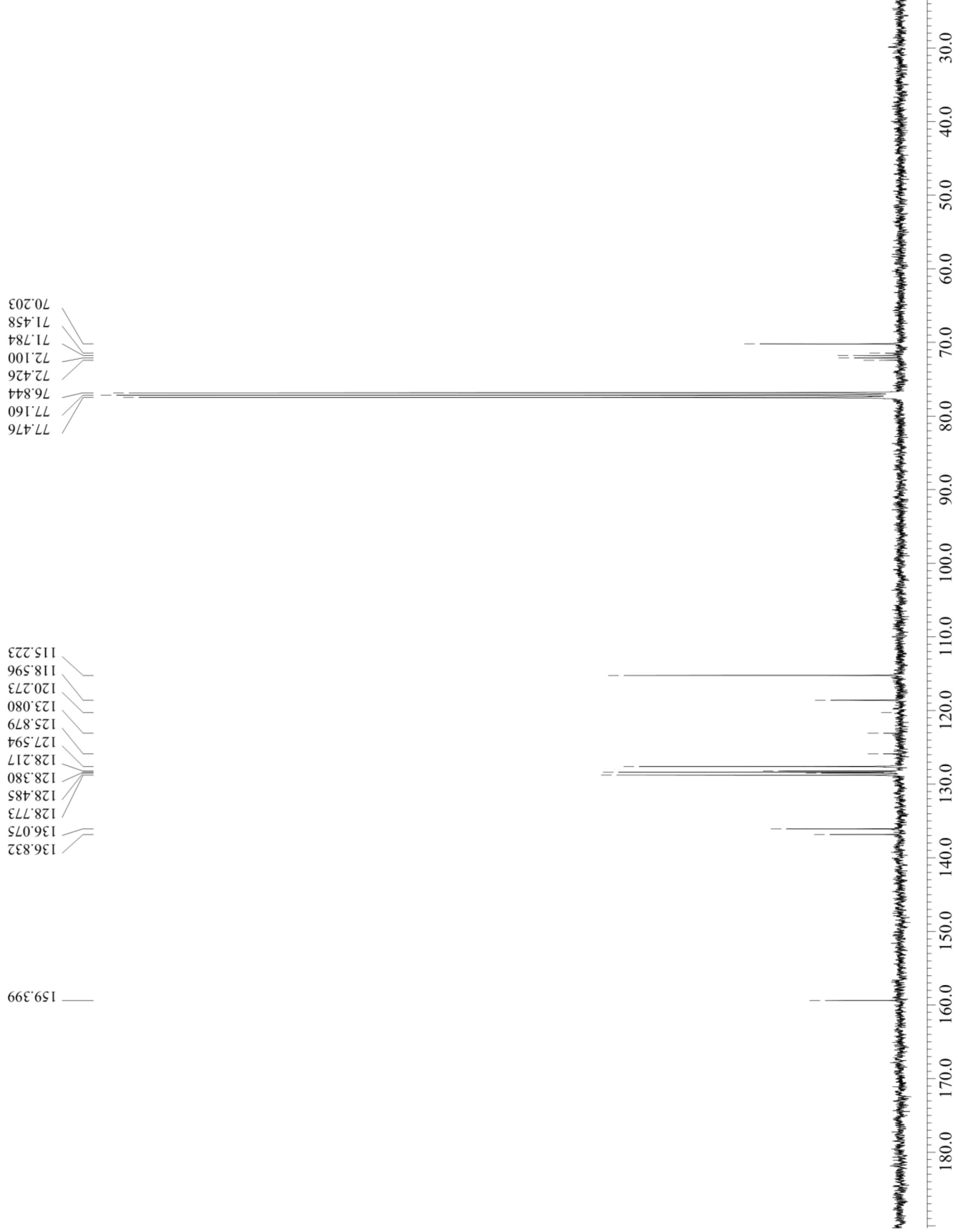

^{19}F NMR of **13j** (CDCl$_3$, 375 MHz, 25 ºC)

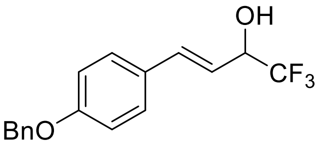

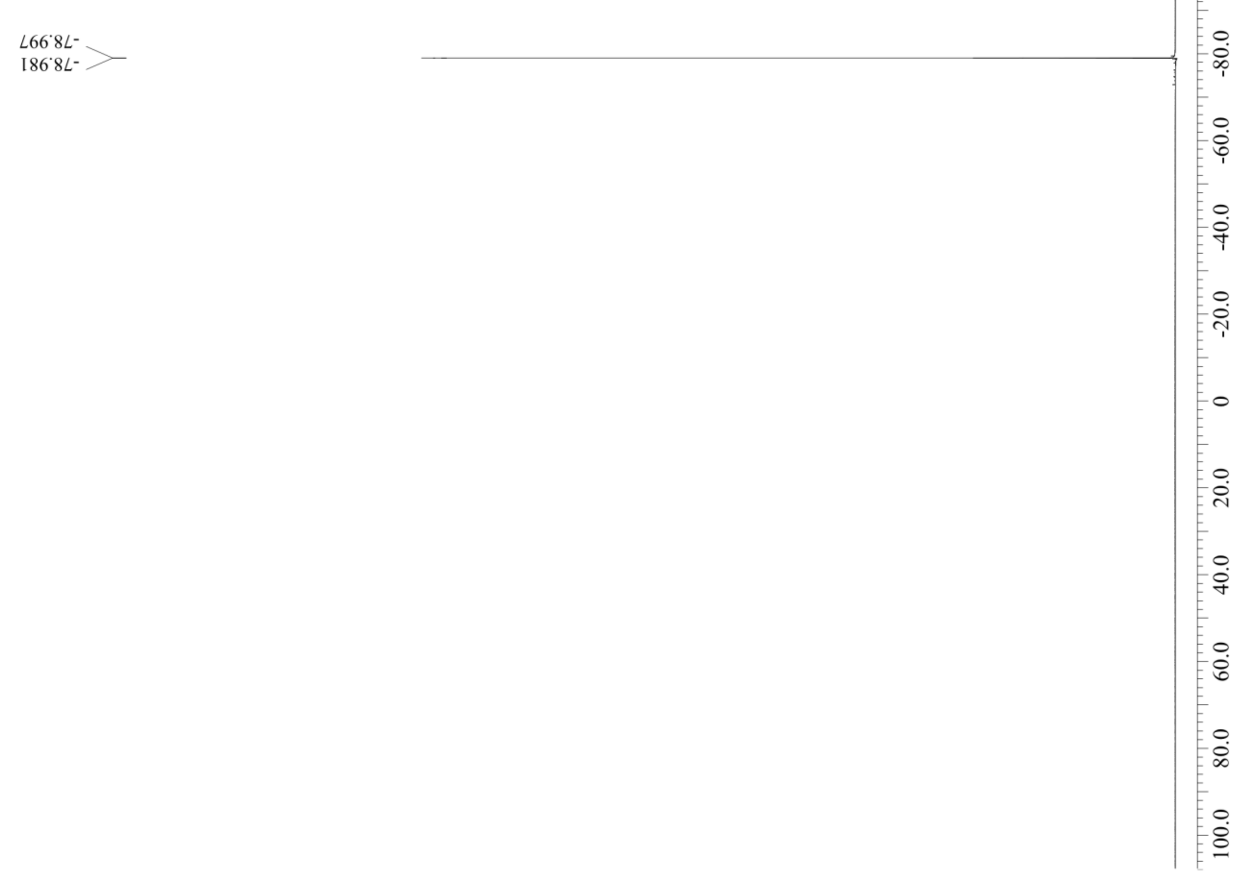

^{1}H NMR of **13k** ($CDCl_3$, 400 MHz, 25 °C)

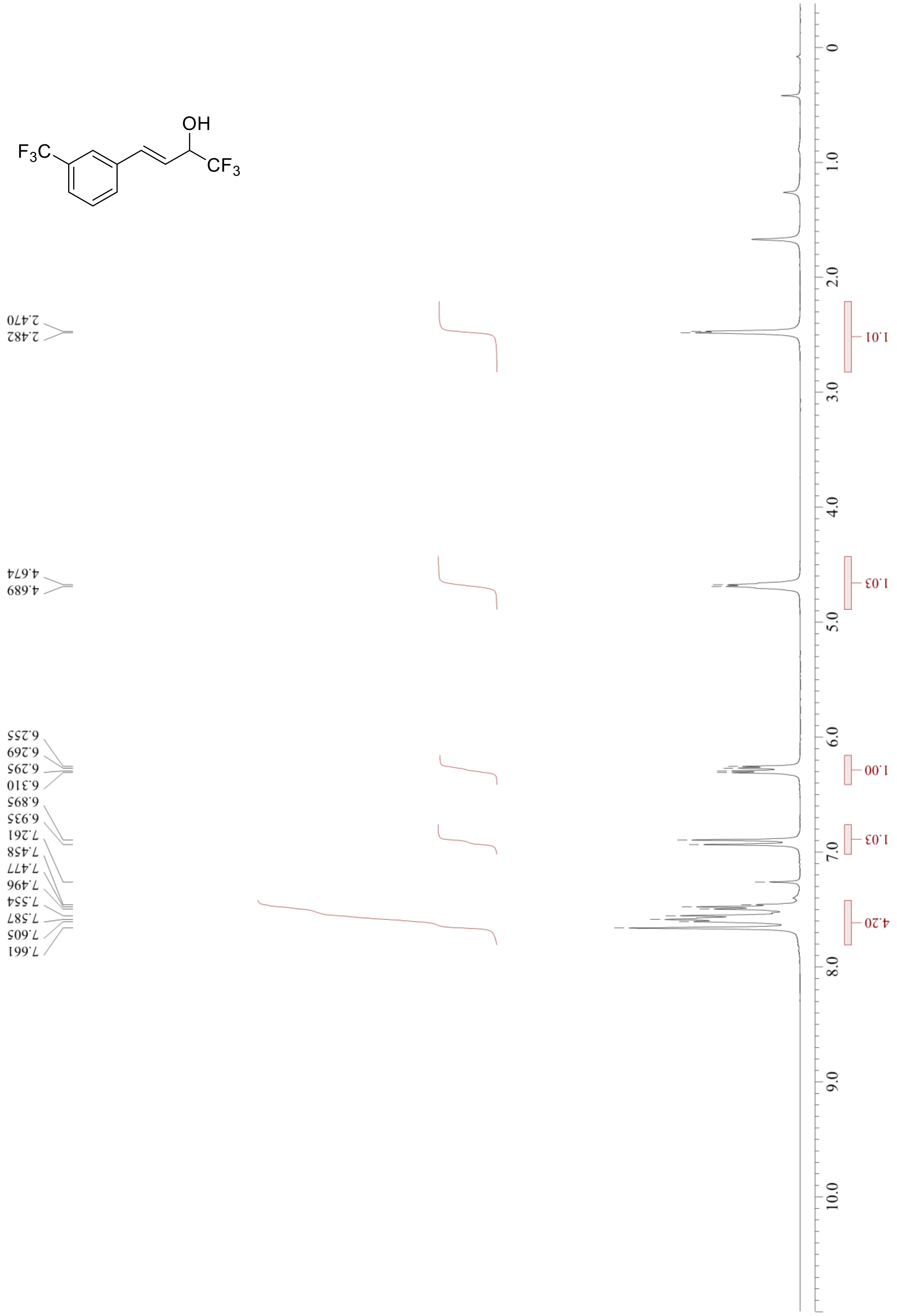

^{13}C NMR of **13k** ($CDCl_3$, 150 MHz, 25 ºC)

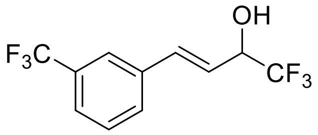

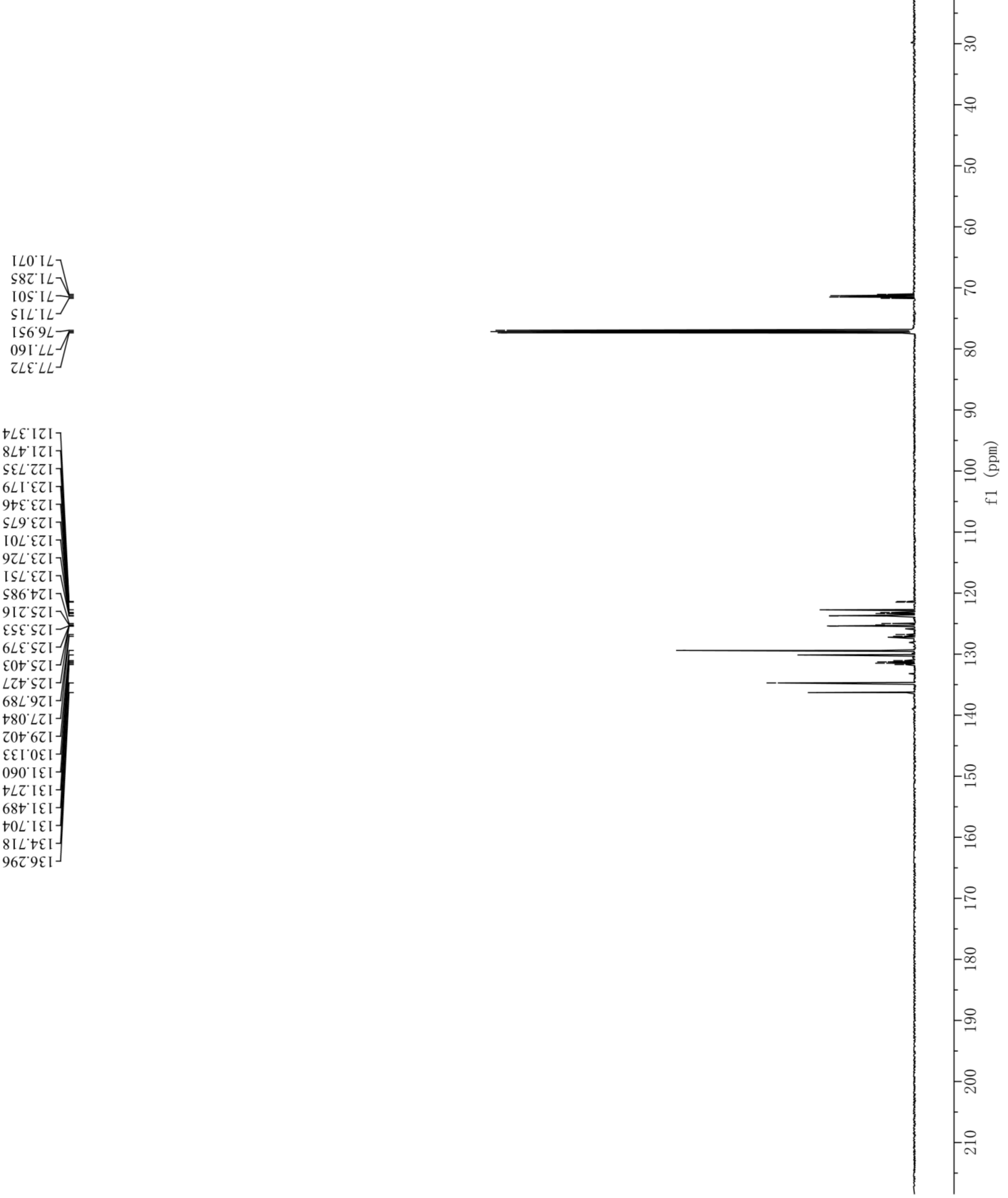

^{19}F NMR of **13k** ($CDCl_3$, 375 MHz, 25 ºC)

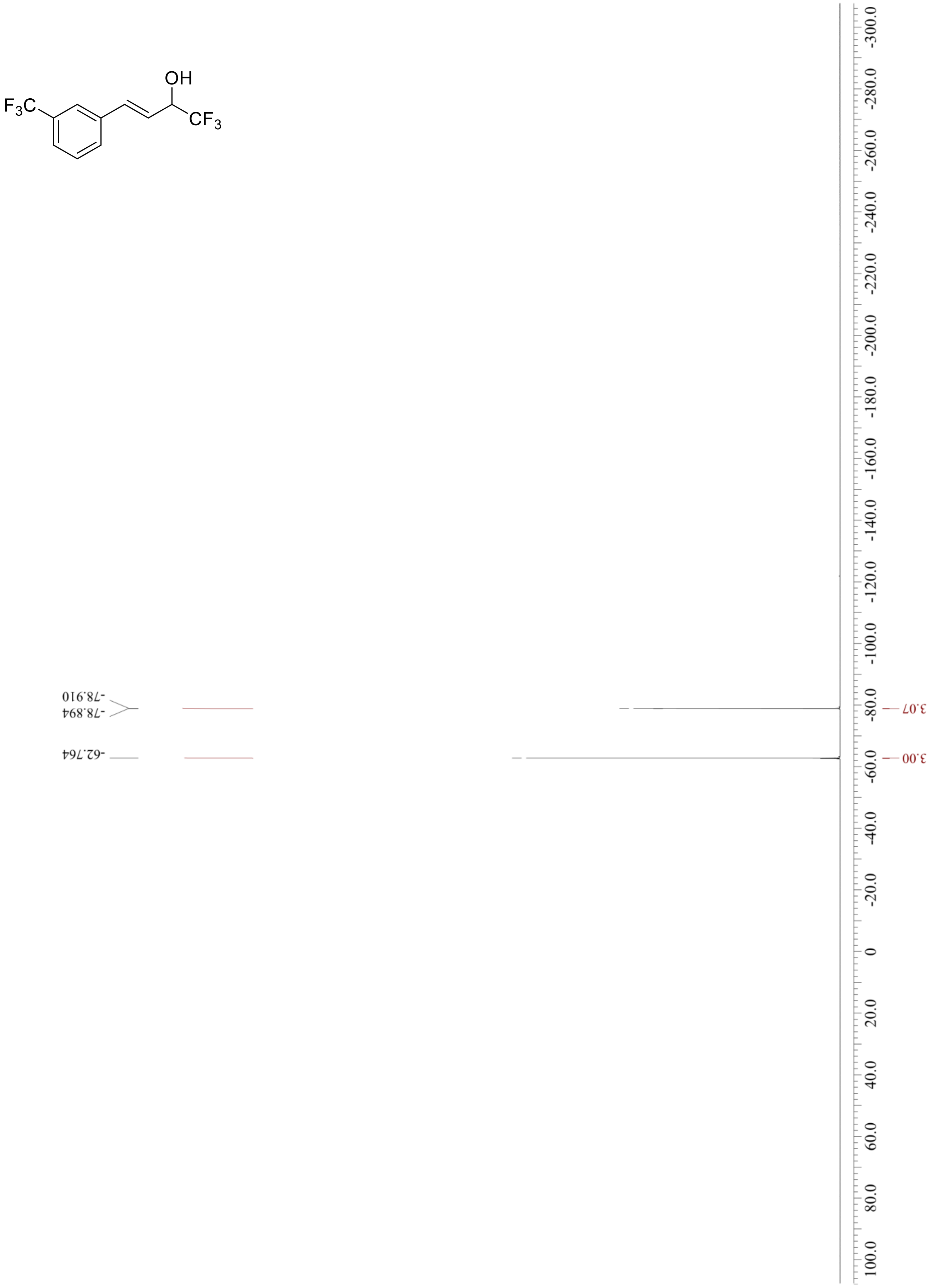

^{1}H NMR of **13l** ($CDCl_3$, 400 MHz, 25 ºC)

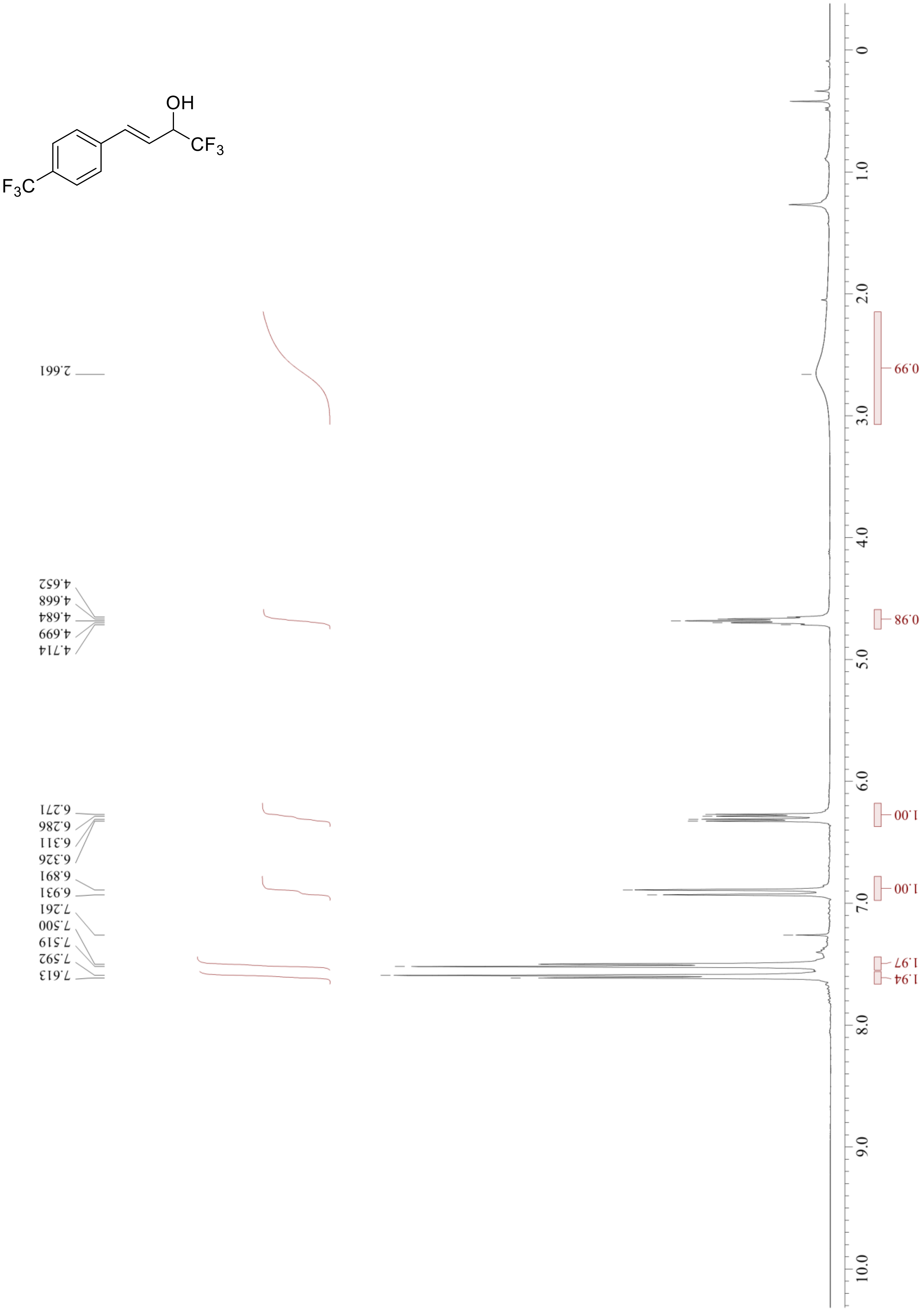

^{13}C NMR of **13l** ($CDCl_3$, 100 MHz, 25 °C)

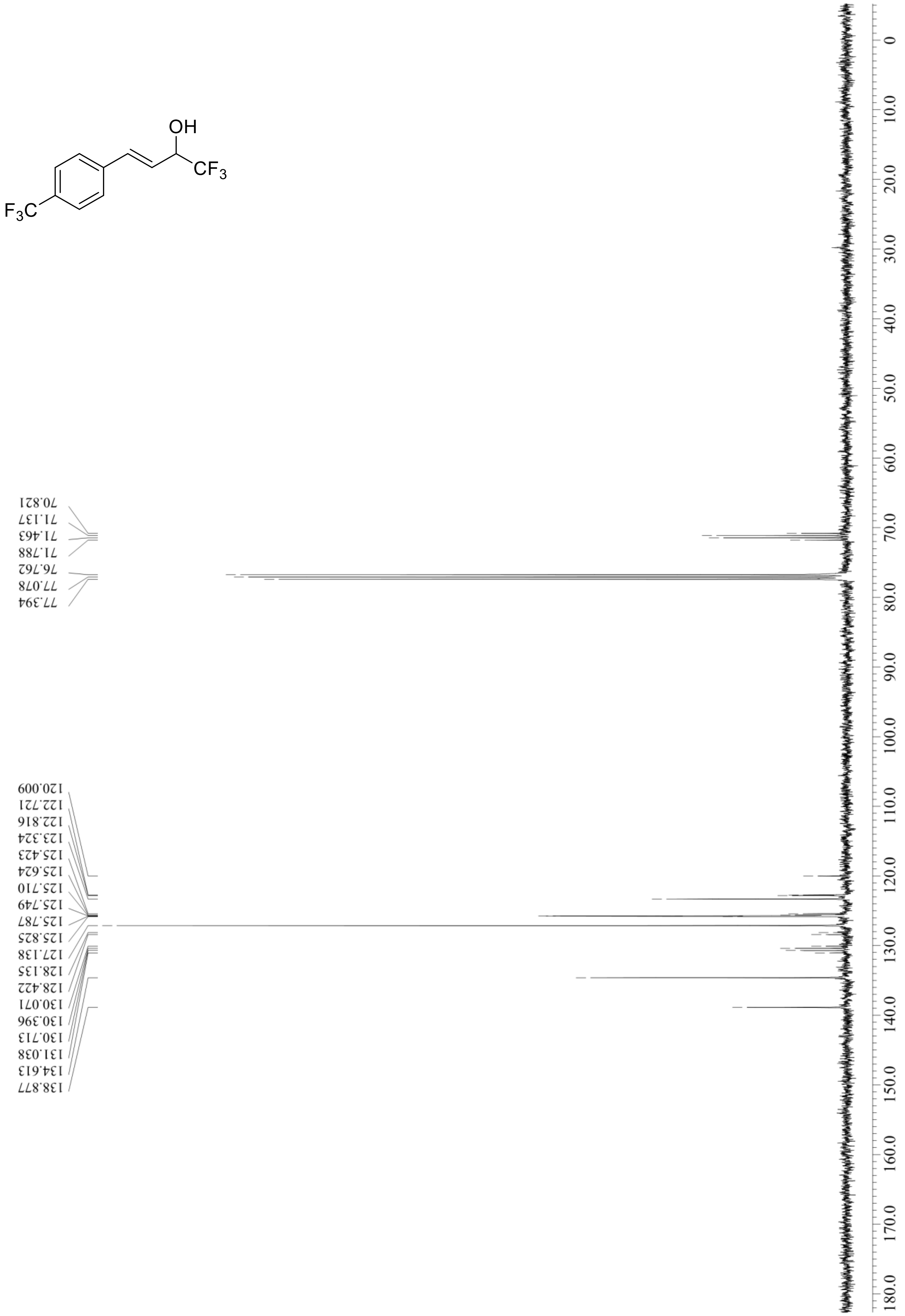

^{19}F NMR of **13l** ($CDCl_3$, 375 MHz, 25 ºC)

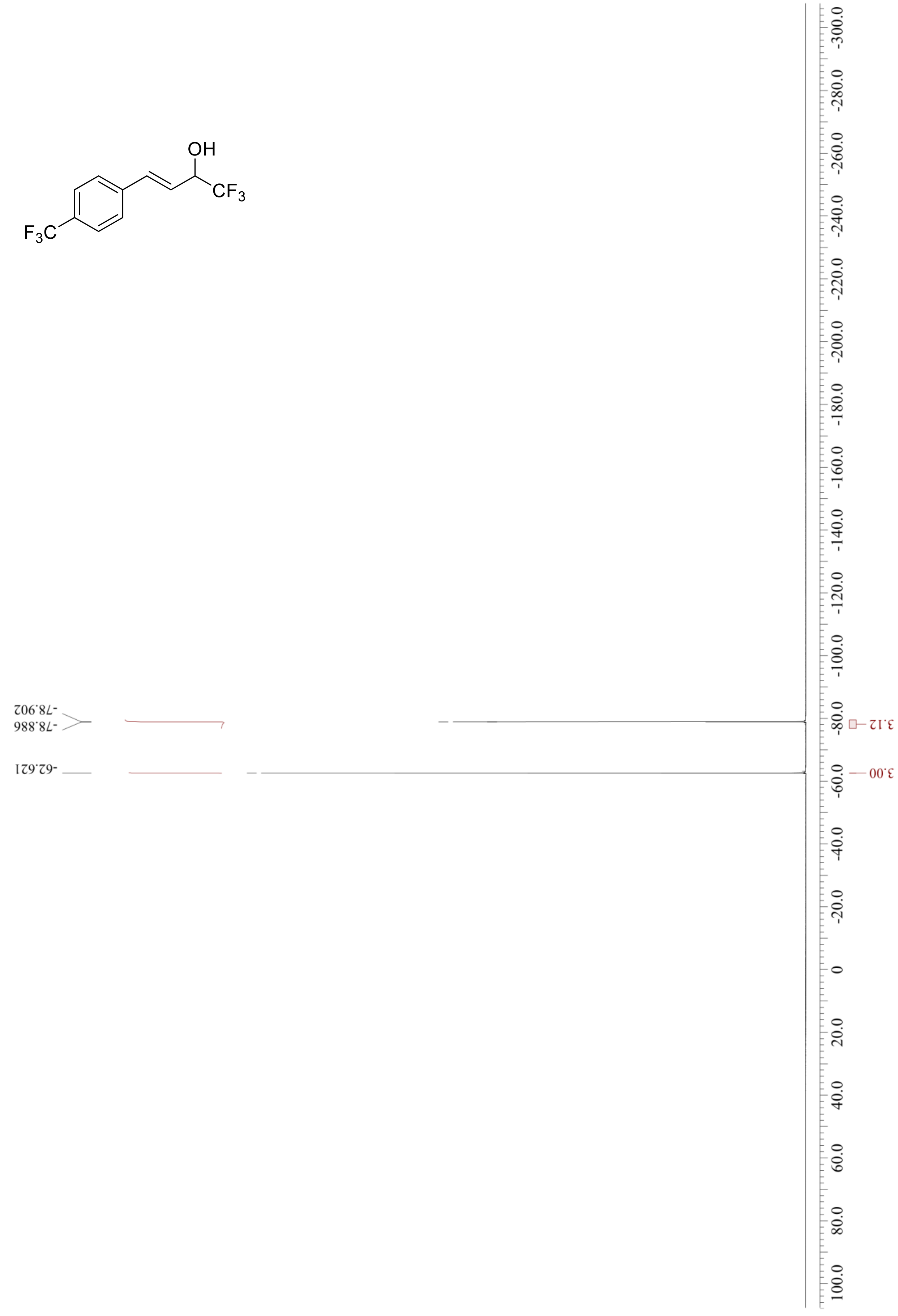

^{1}H NMR of **14a** ($CDCl_3$, 400 MHz, 25 °C)

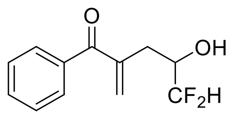

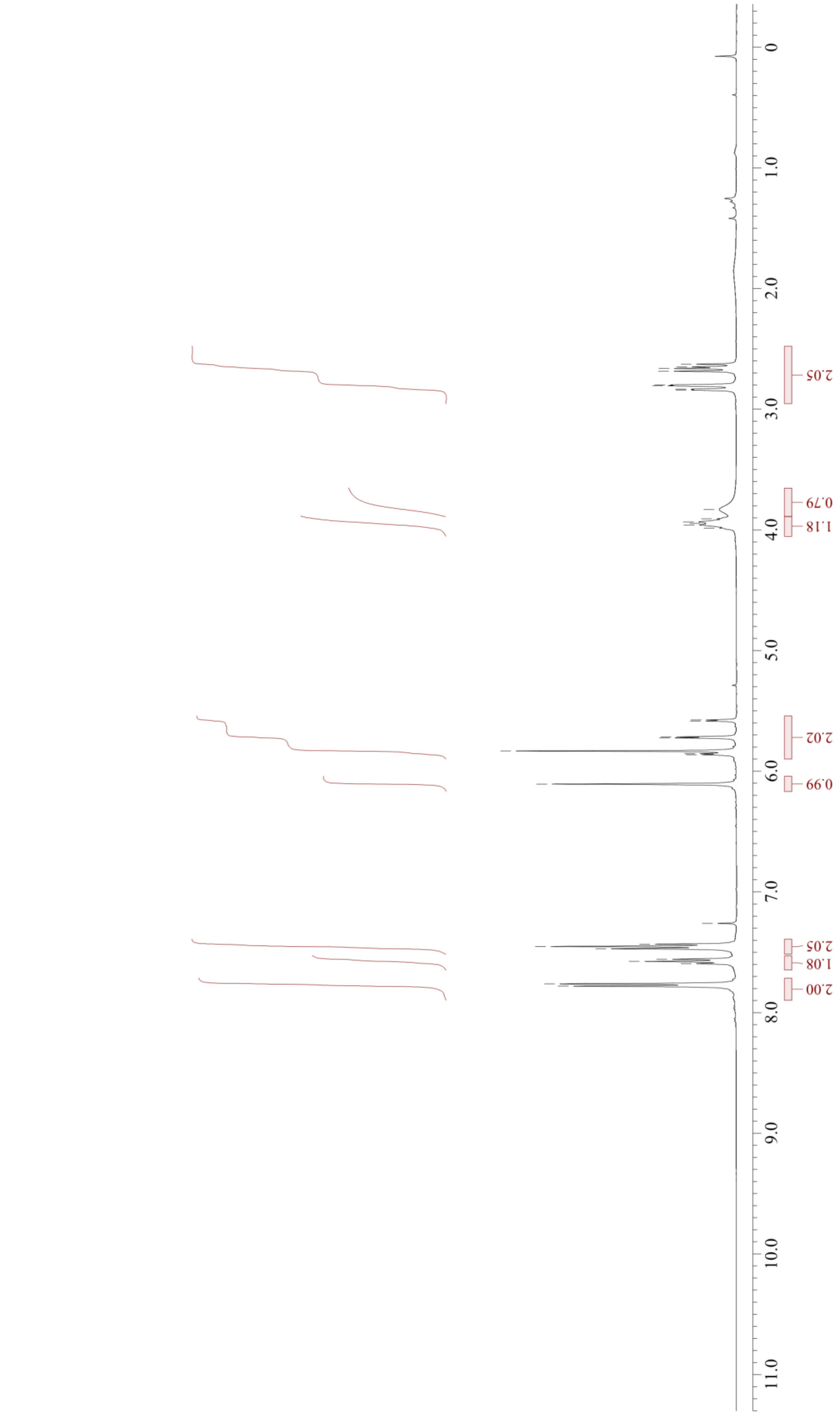

^{13}C NMR of **14a** ($CDCl_3$, 100 MHz, 25 ºC)

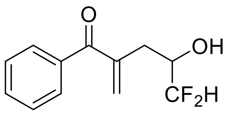

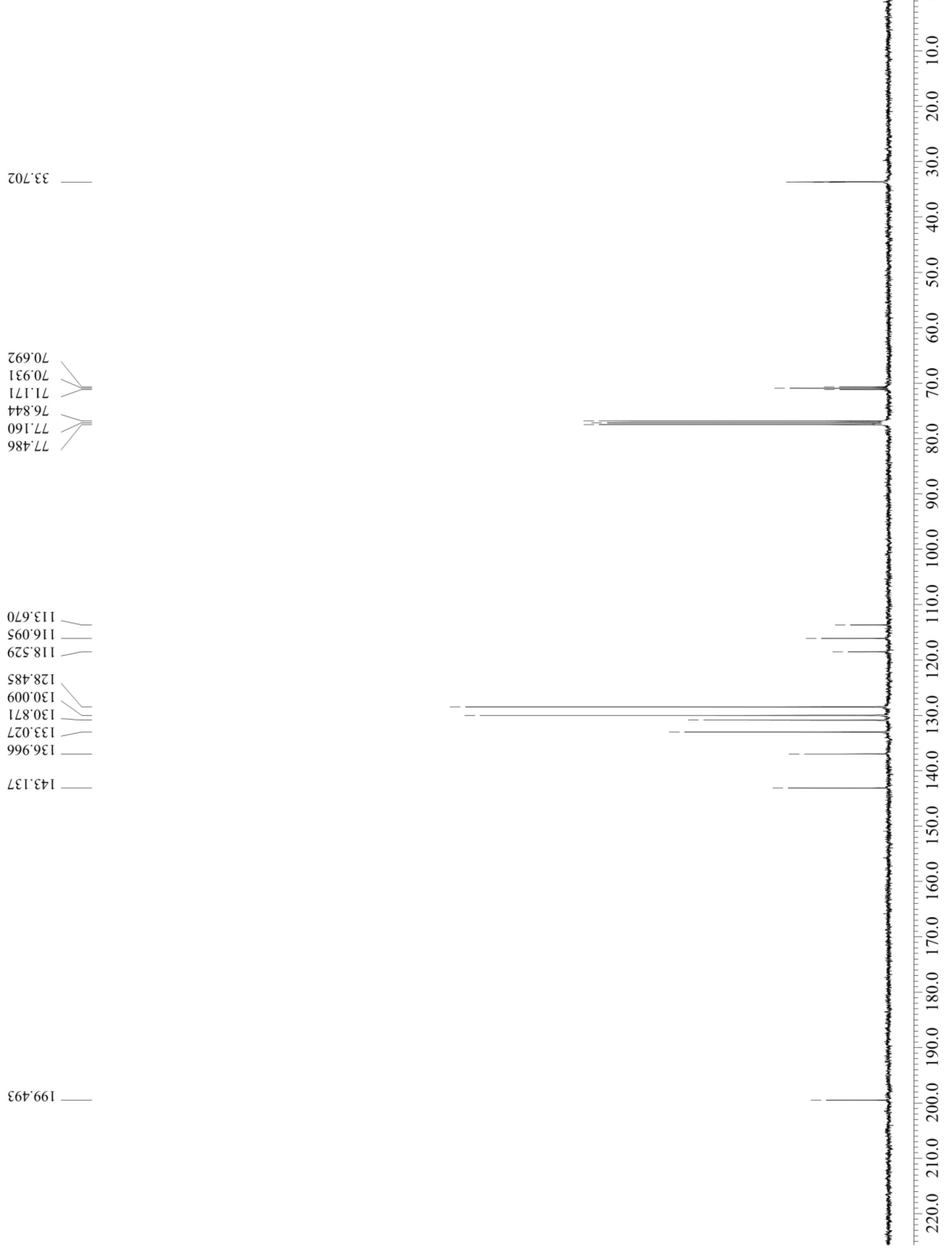

^{19}F NMR of **14a** ($CDCl_3$, 375 MHz, 25 ºC)

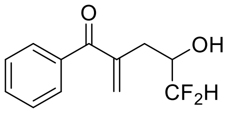

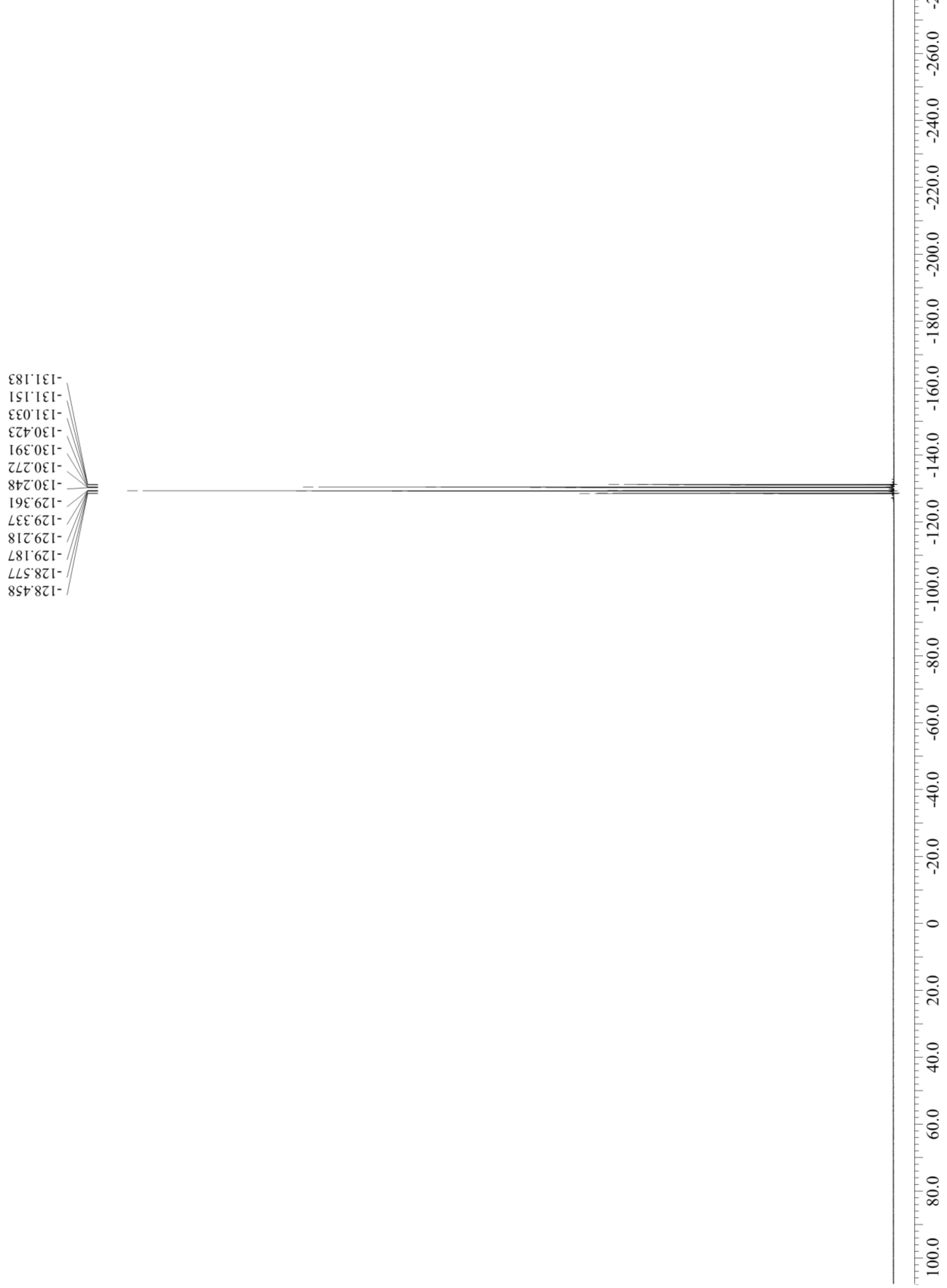

^{1}H NMR of **14b** ($CDCl_3$, 400 MHz, 25 ºC)

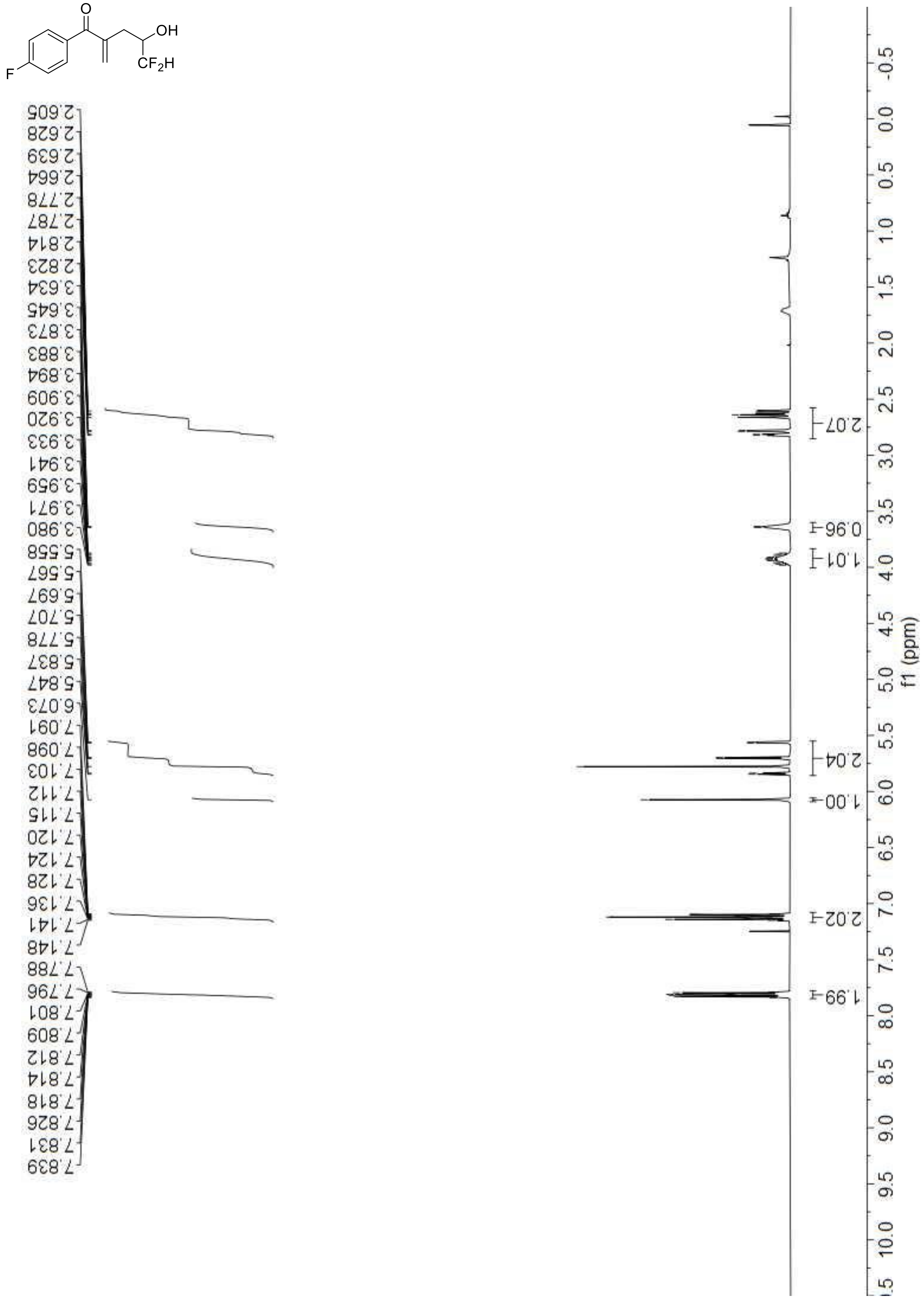

^{13}C NMR of **14b** ($CDCl_3$, 100 MHz, 25 ºC)

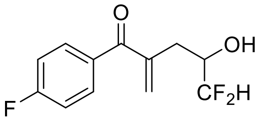

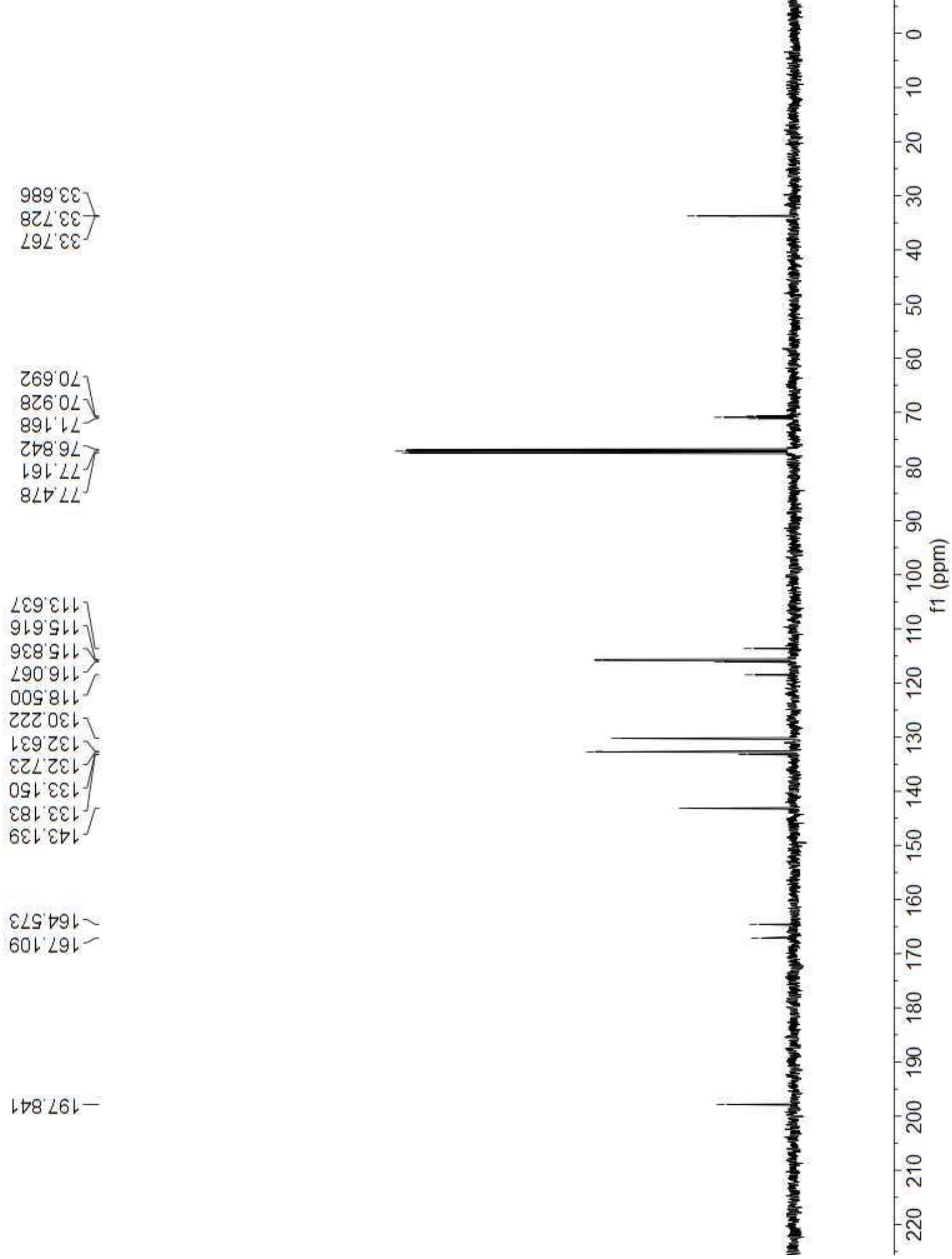

^{19}F NMR of **14b** ($CDCl_3$, 375 MHz, 25 ºC)

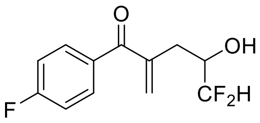

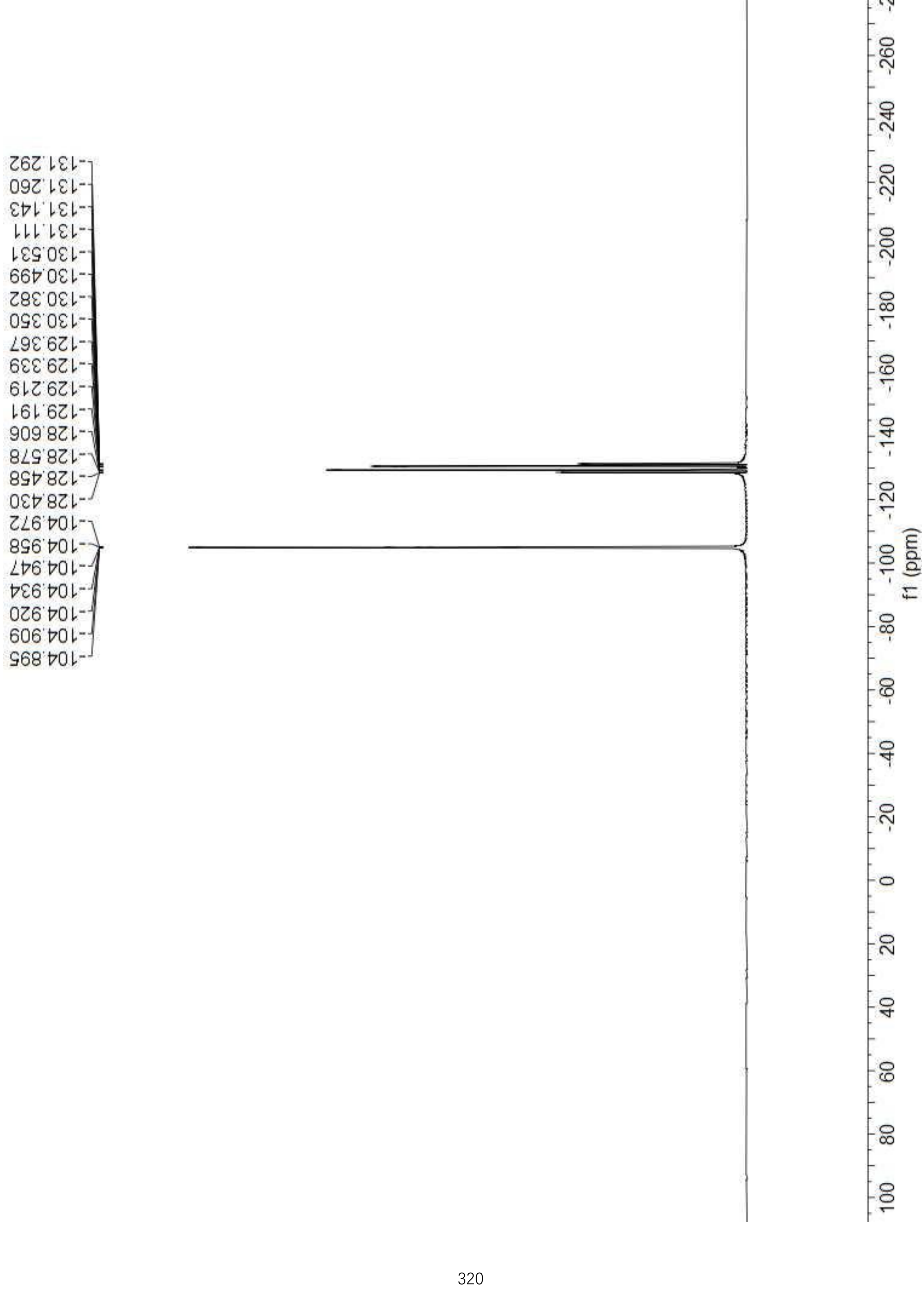

^{1}H NMR of **14c** ($CDCl_3$, 400 MHz, 25 ºC)

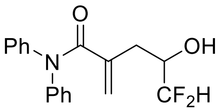

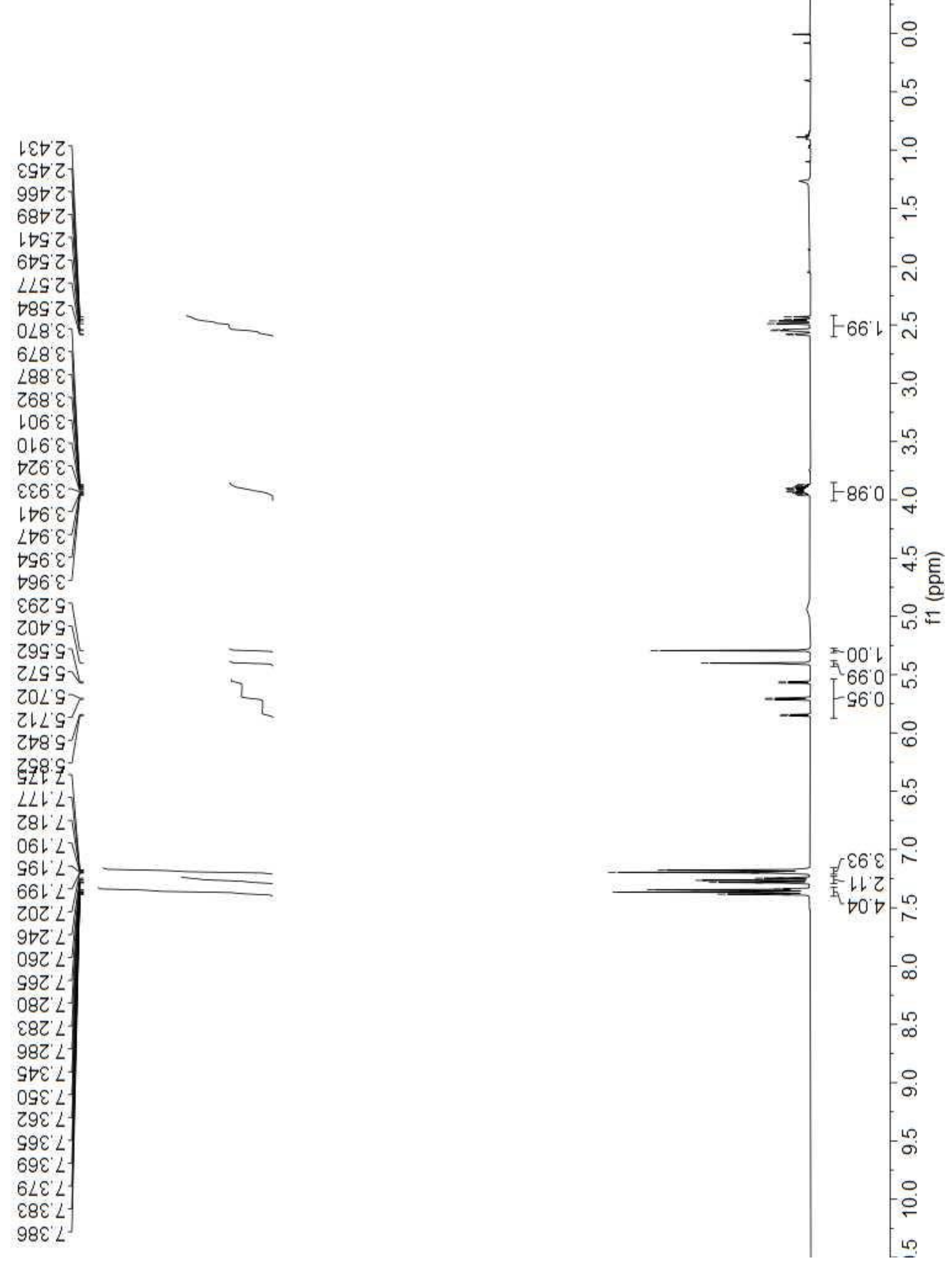

^{13}C NMR of **14c** ($CDCl_3$, 100 MHz, 25 ºC)

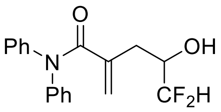

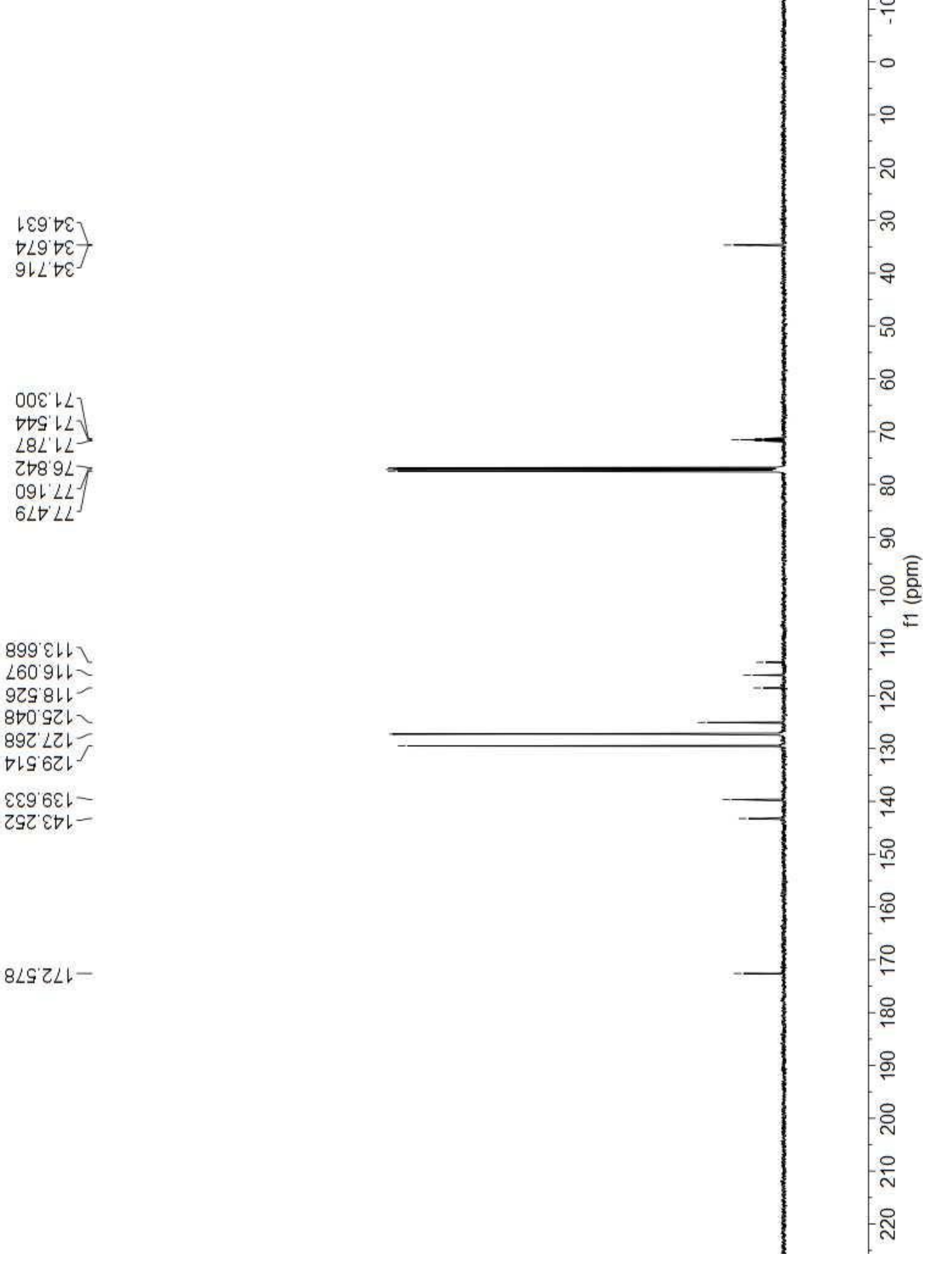

^{19}F NMR of **14c** ($CDCl_3$, 375 MHz, 25 ºC)

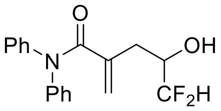

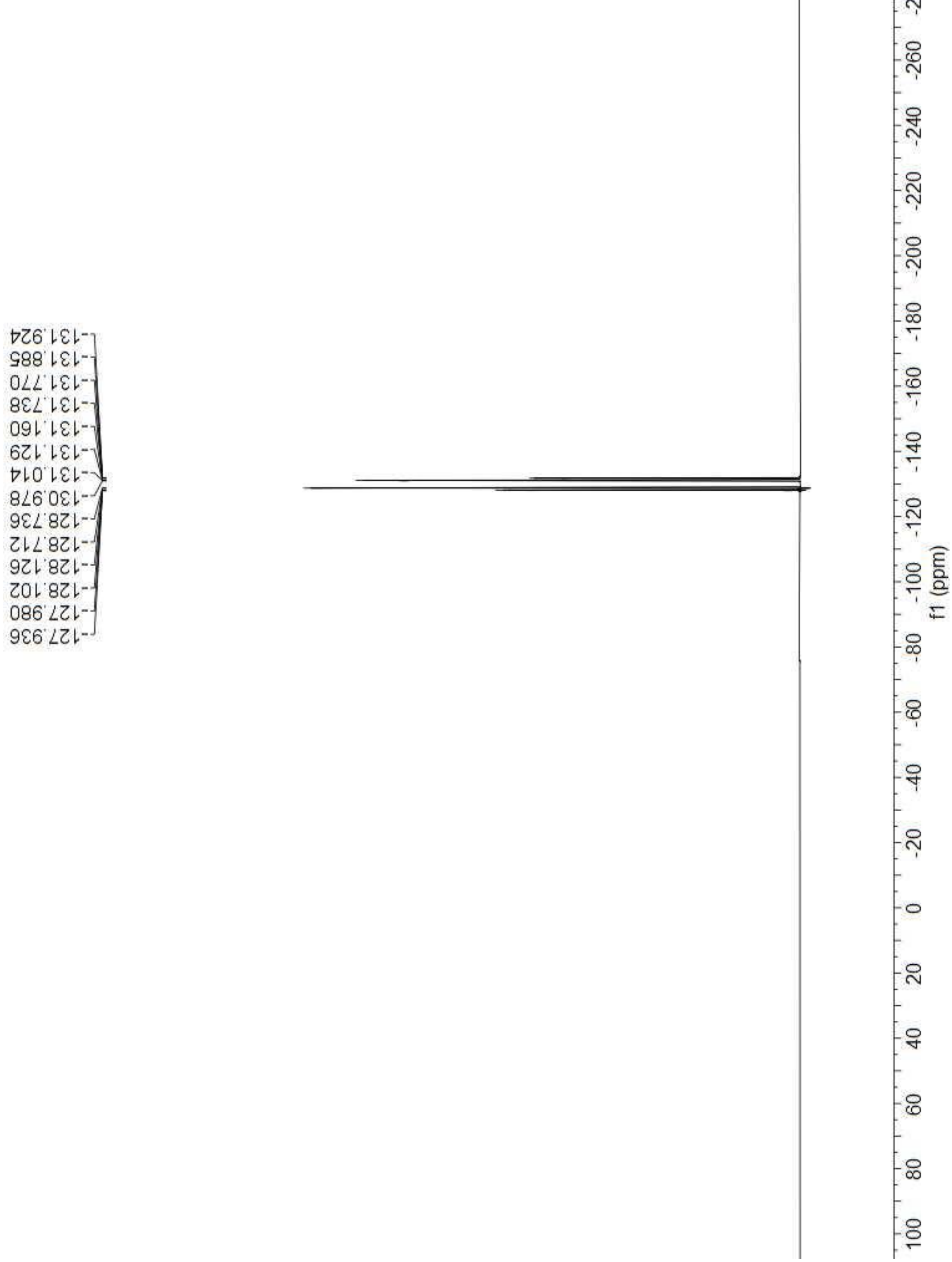

^{1}H NMR of **14d** ($CDCl_3$, 400 MHz, 25 ºC)

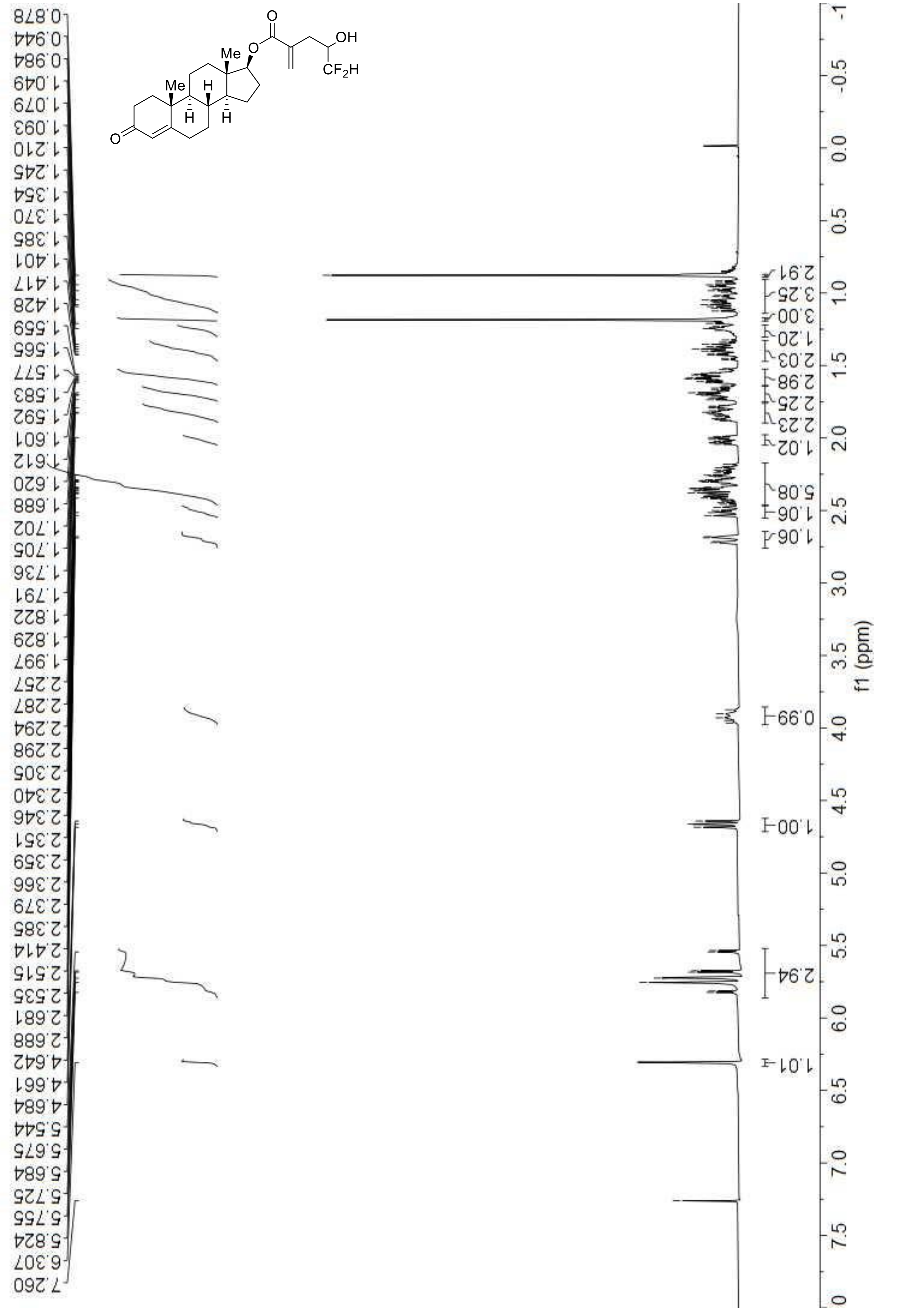

^{13}C NMR of **14d** ($CDCl_3$, 100 MHz, 25 ºC)

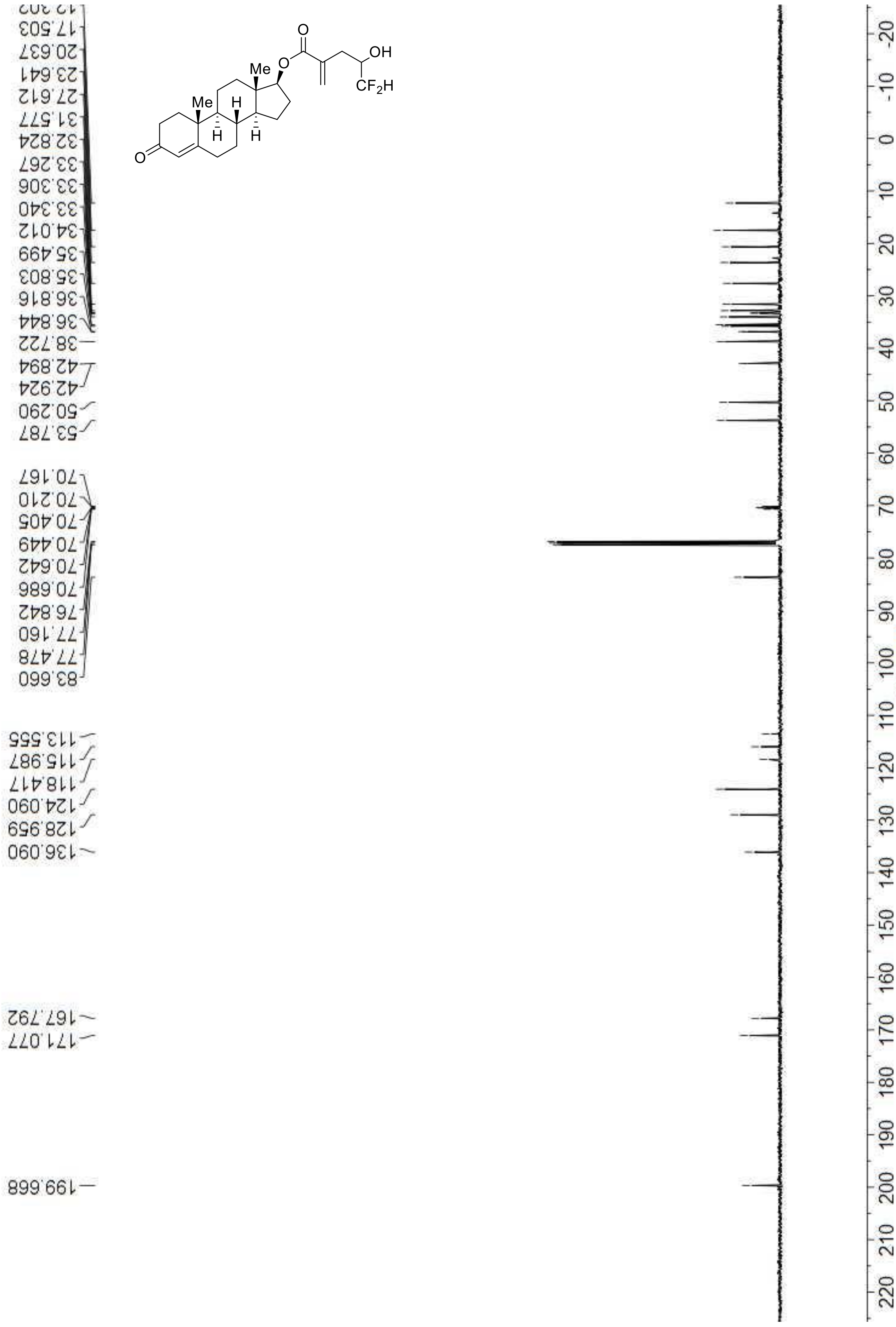

^{19}F NMR of **14d** ($CDCl_3$, 375 MHz, 25 ºC)

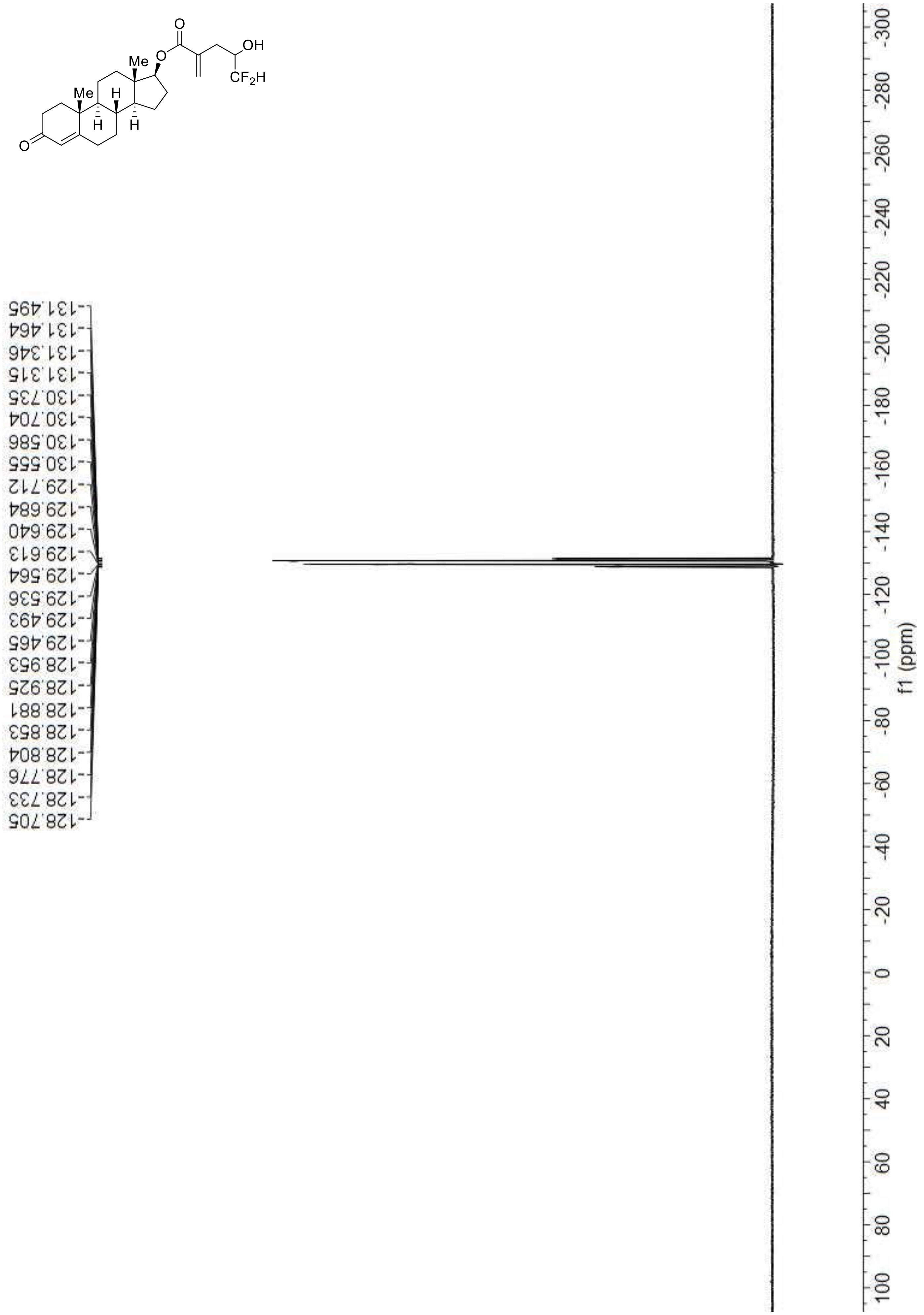

^{1}H NMR of **14e** ($CDCl_3$, 400 MHz, 25 ºC)

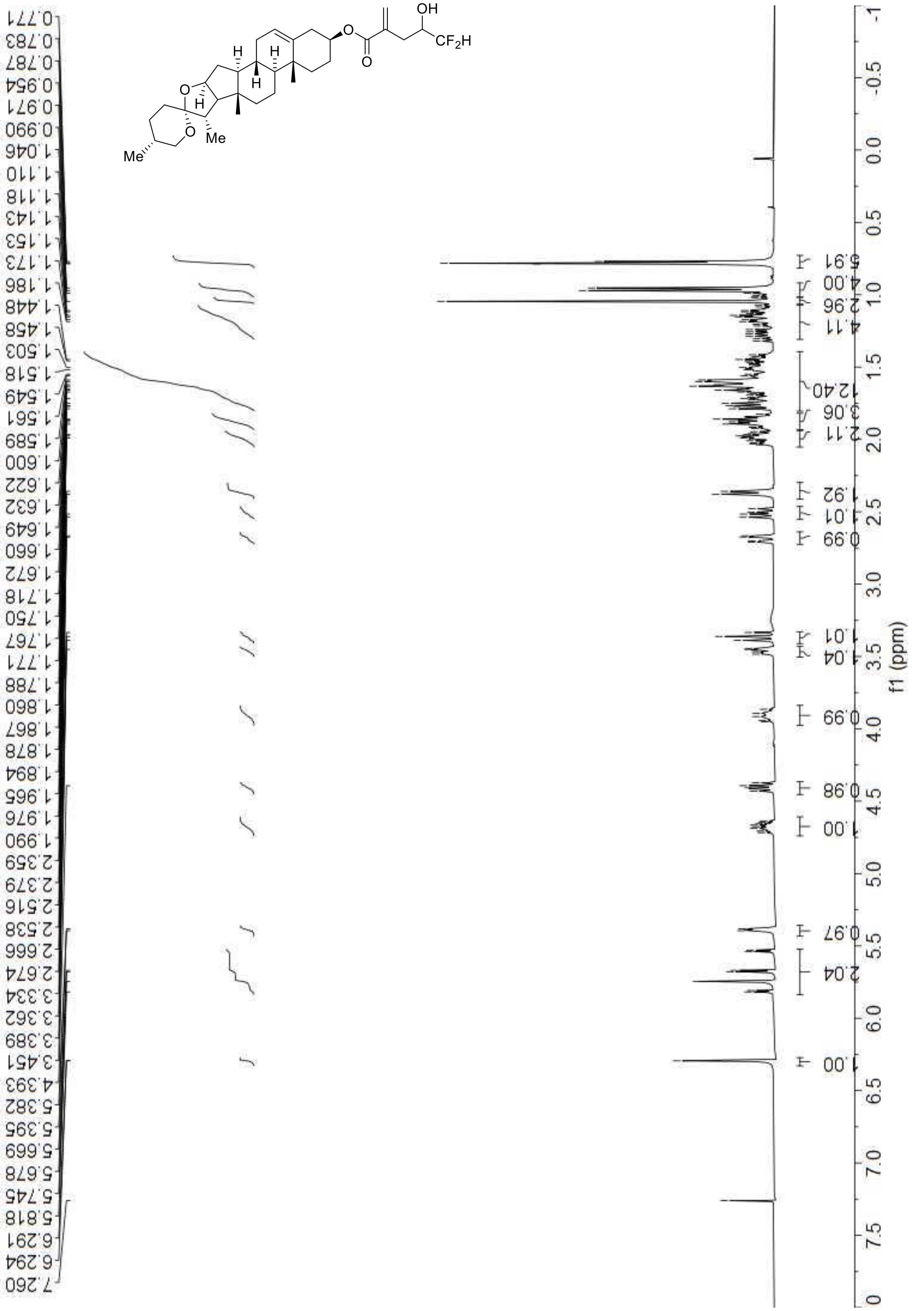

^{13}C NMR of **14e** ($CDCl_3$, 100 MHz, 25 ºC)

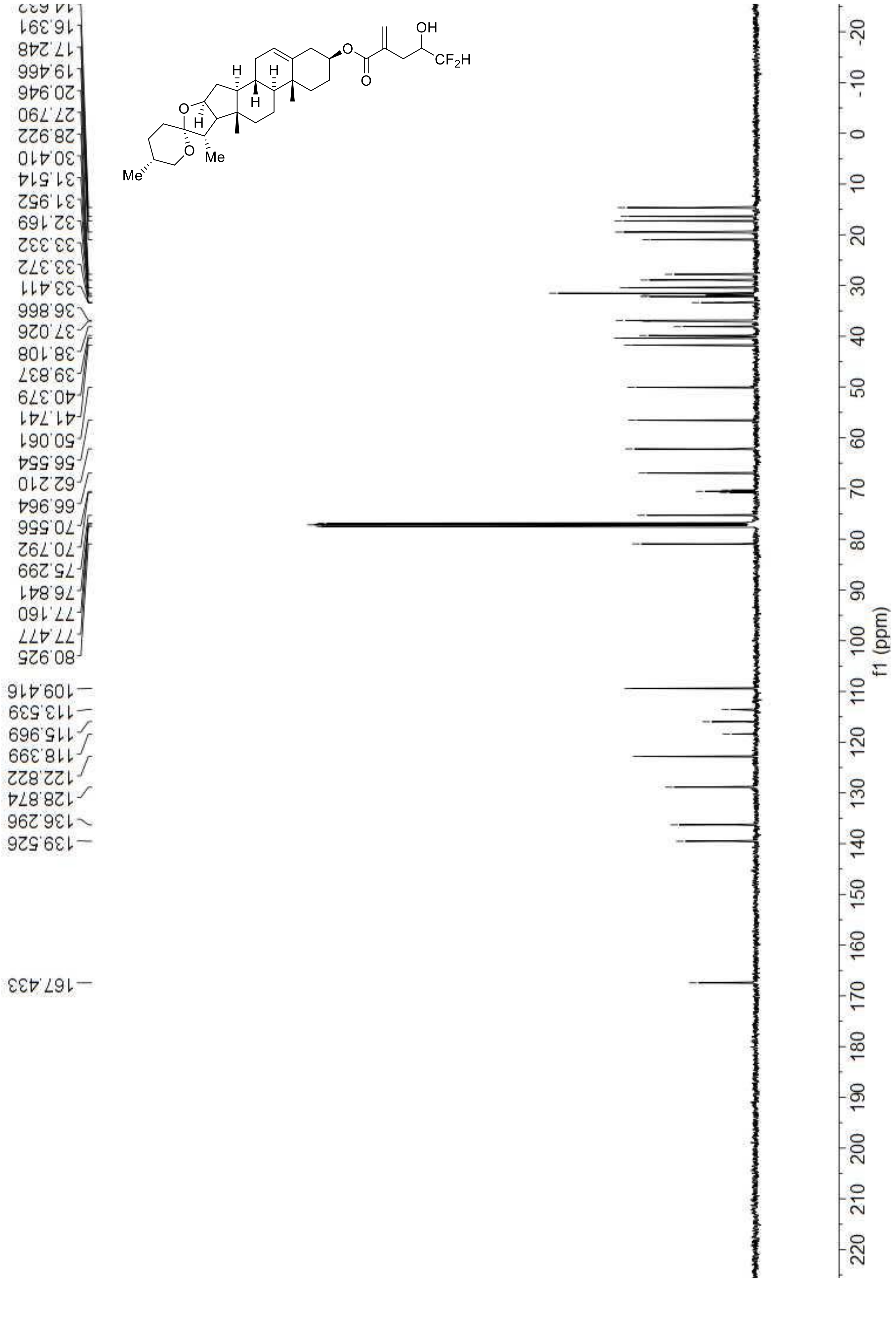

^{19}F NMR of **14e** ($CDCl_3$, 375 MHz, 25 ºC)

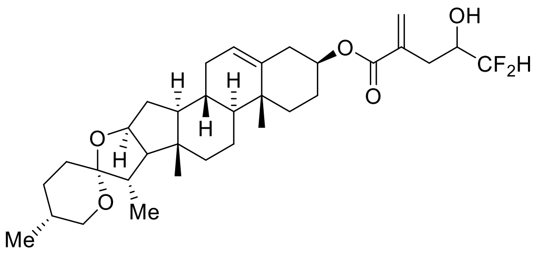

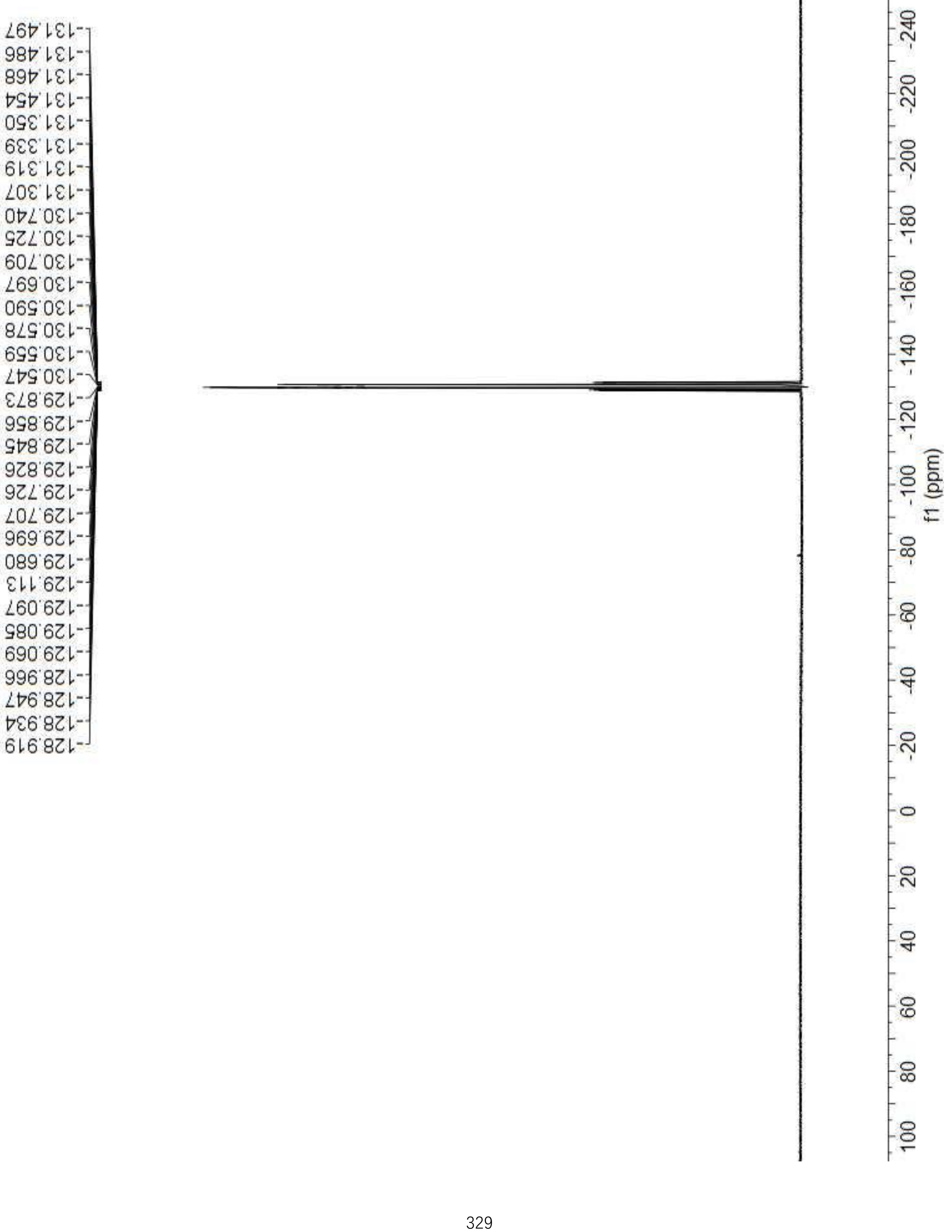

^{1}H NMR of **14f-a** ($CDCl_3$, 400 MHz, 25 ºC)

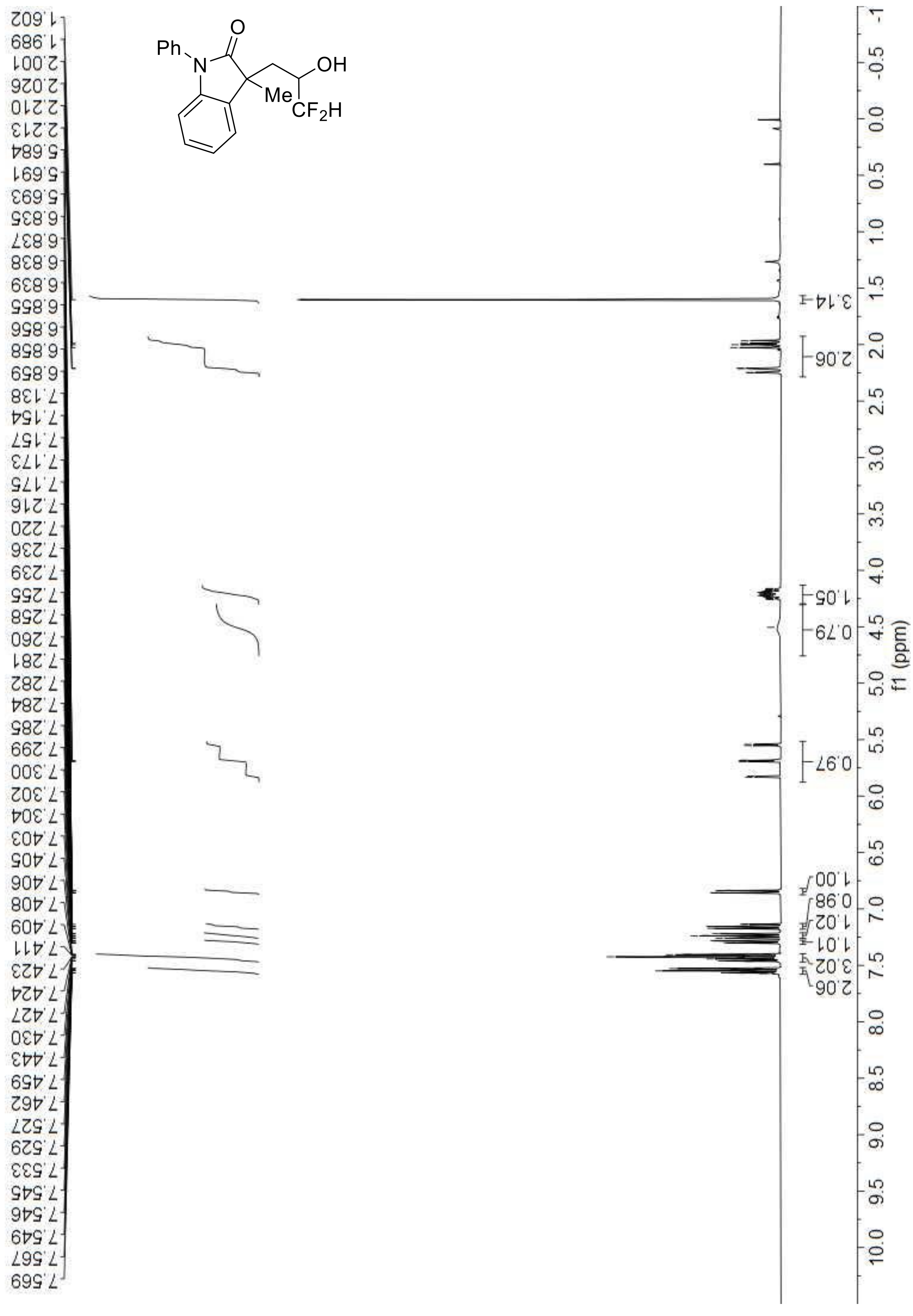

^{13}C NMR of **14f-a** ($CDCl_3$, 100 MHz, 25 ºC)

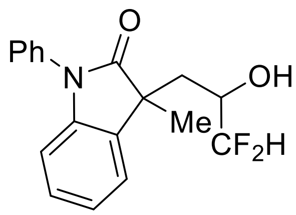

14f-a

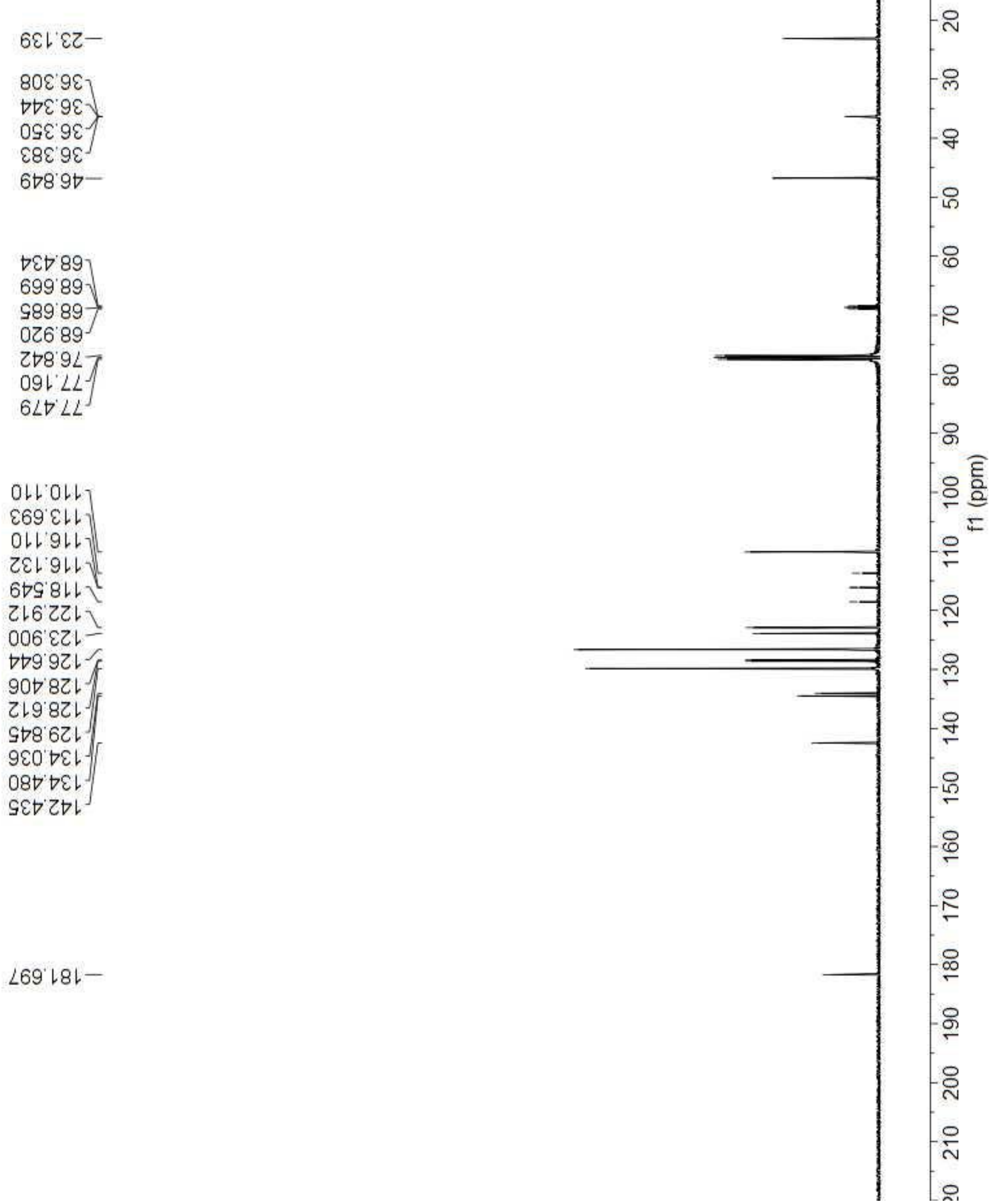

^{19}F NMR of **14f-a** ($CDCl_3$, 375 MHz, 25 ºC)

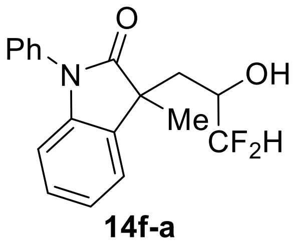

14f-a

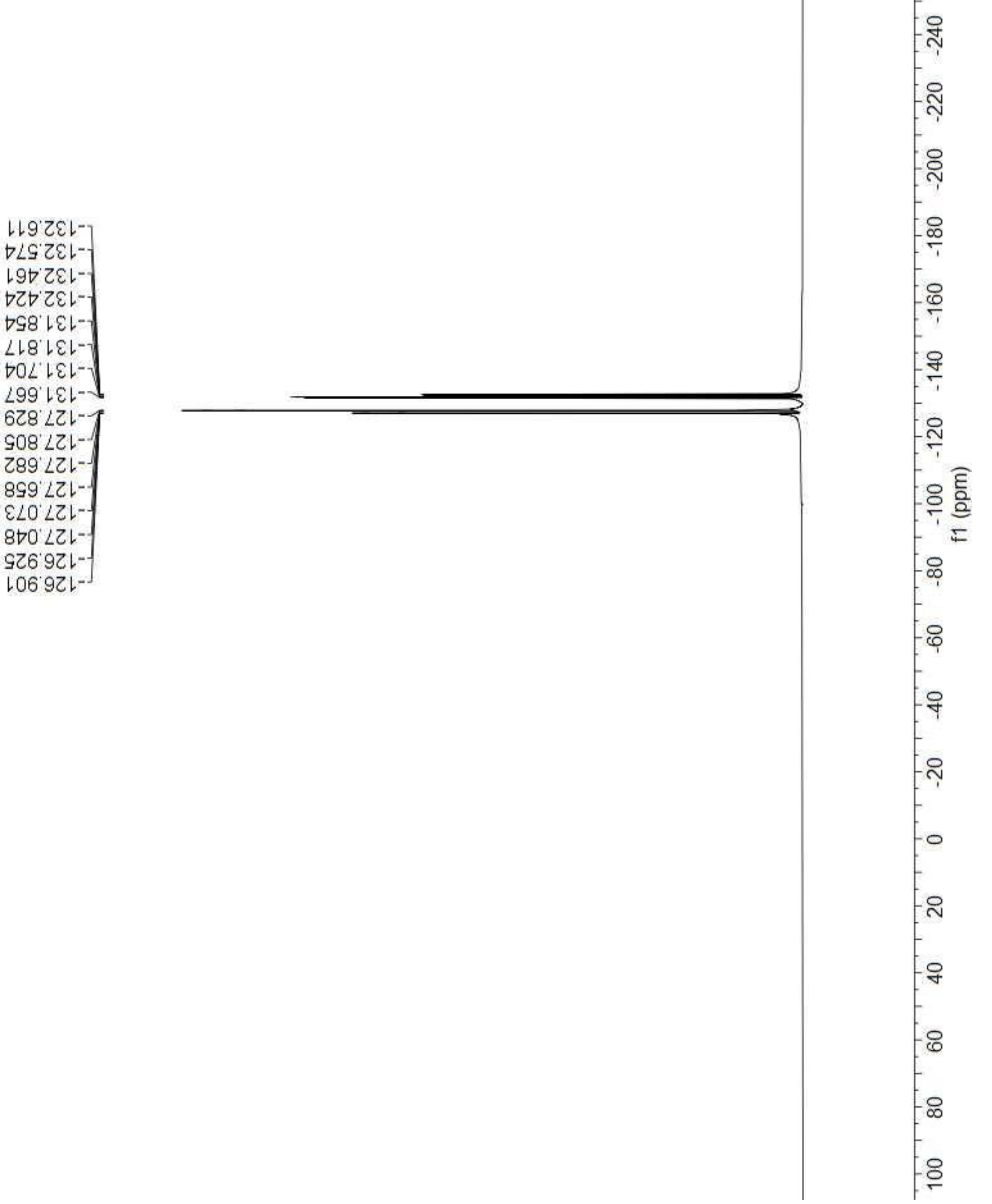

^{1}H NMR of **14f-b** ($CDCl_3$, 400 MHz, 25 ºC)

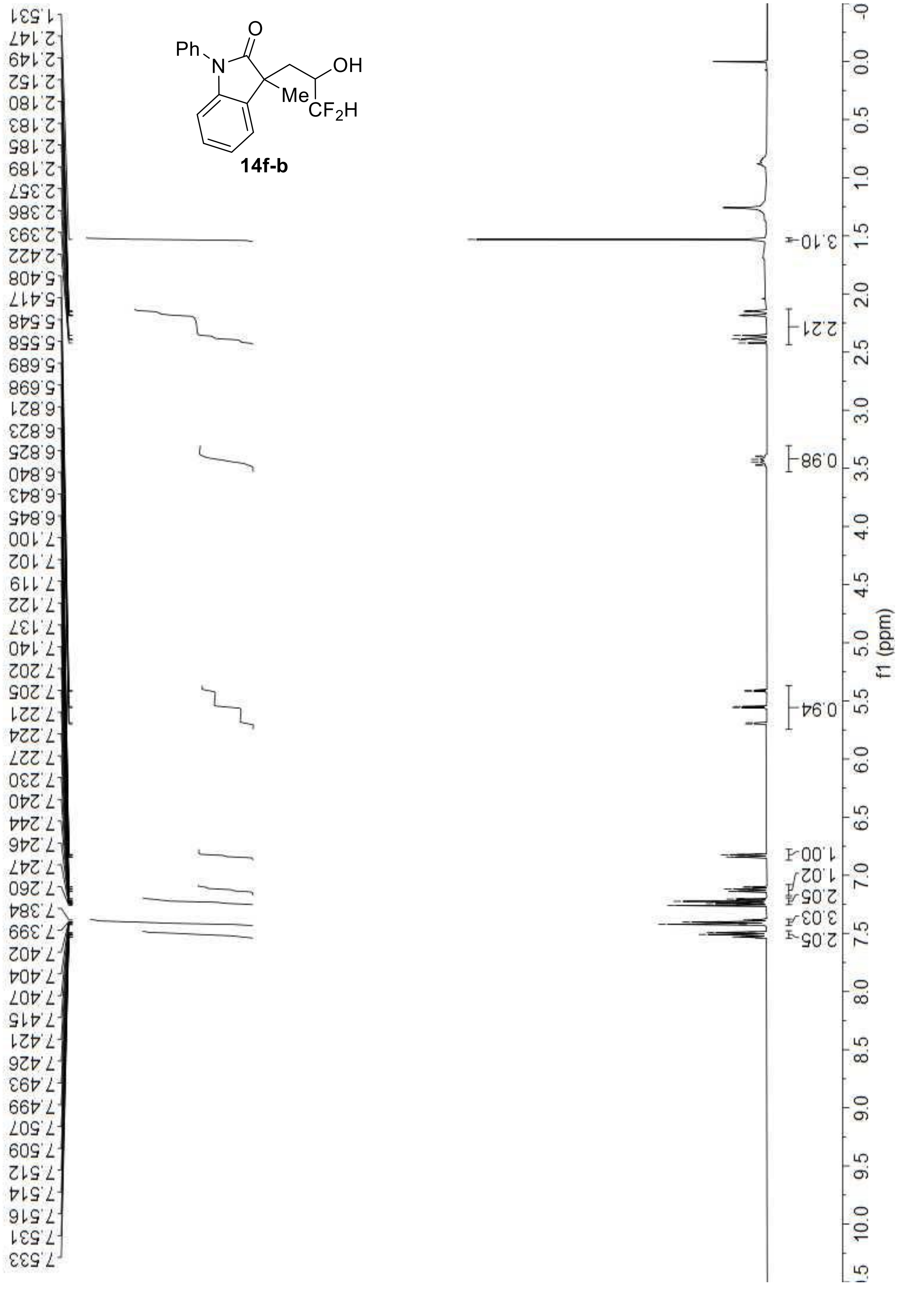

^{13}C NMR of **14f-b** ($CDCl_3$, 100 MHz, 25 ºC)

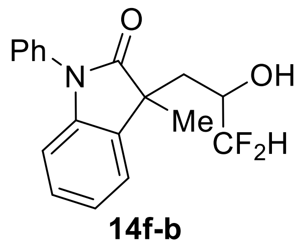

14f-b

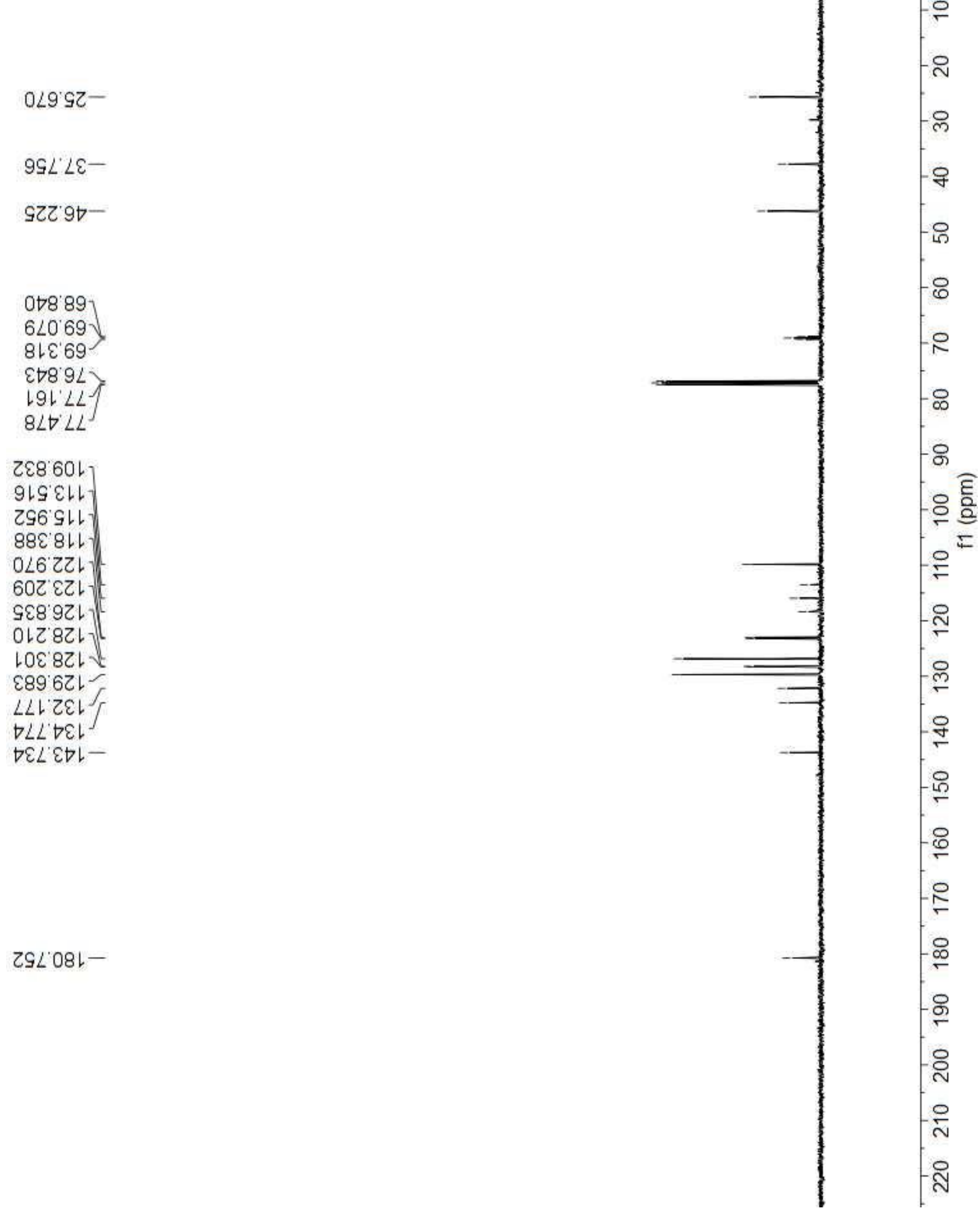

^{19}F NMR of **14f-b** ($CDCl_3$, 375 MHz, 25 ºC)

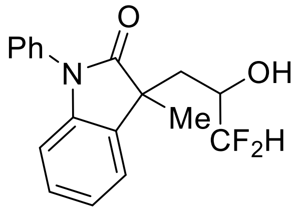

14f-b

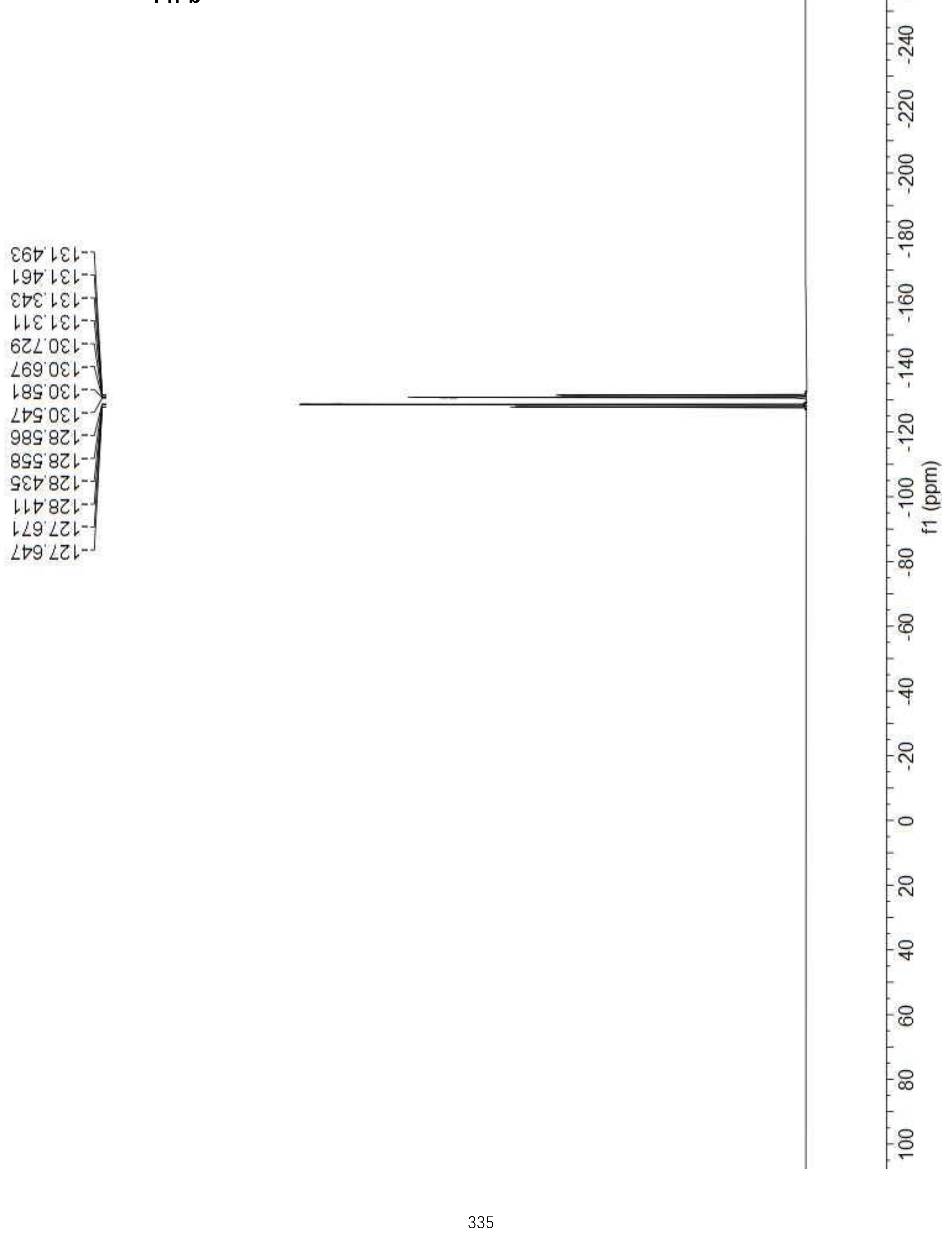

^{1}H NMR of **14g-a** ($CDCl_3$, 400 MHz, 25 ºC)

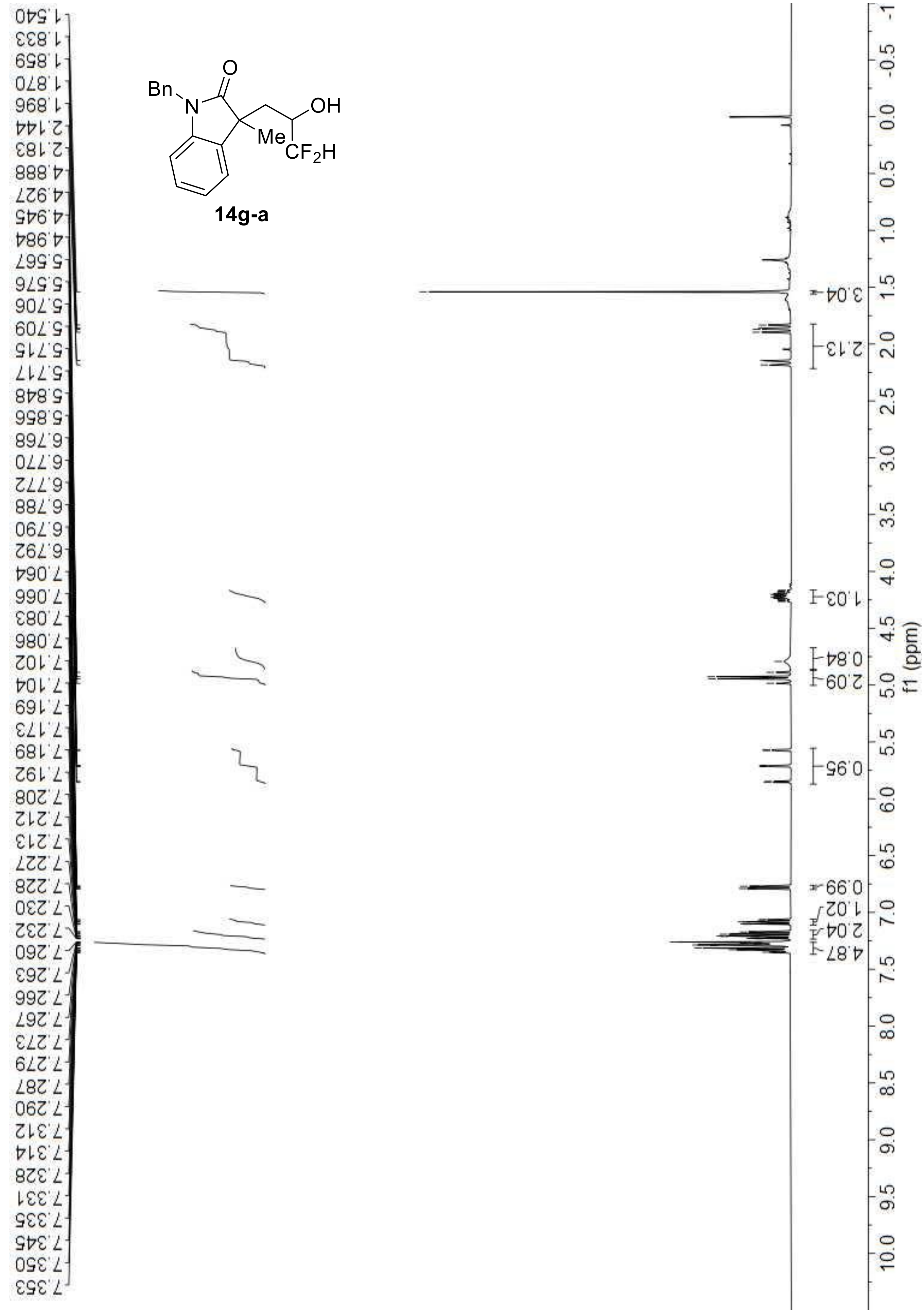

^{13}C NMR of **14g-a** ($CDCl_3$, 100 MHz, 25 °C)

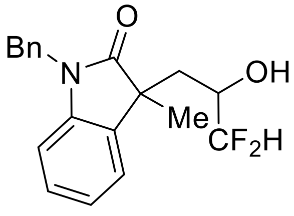

14g-a

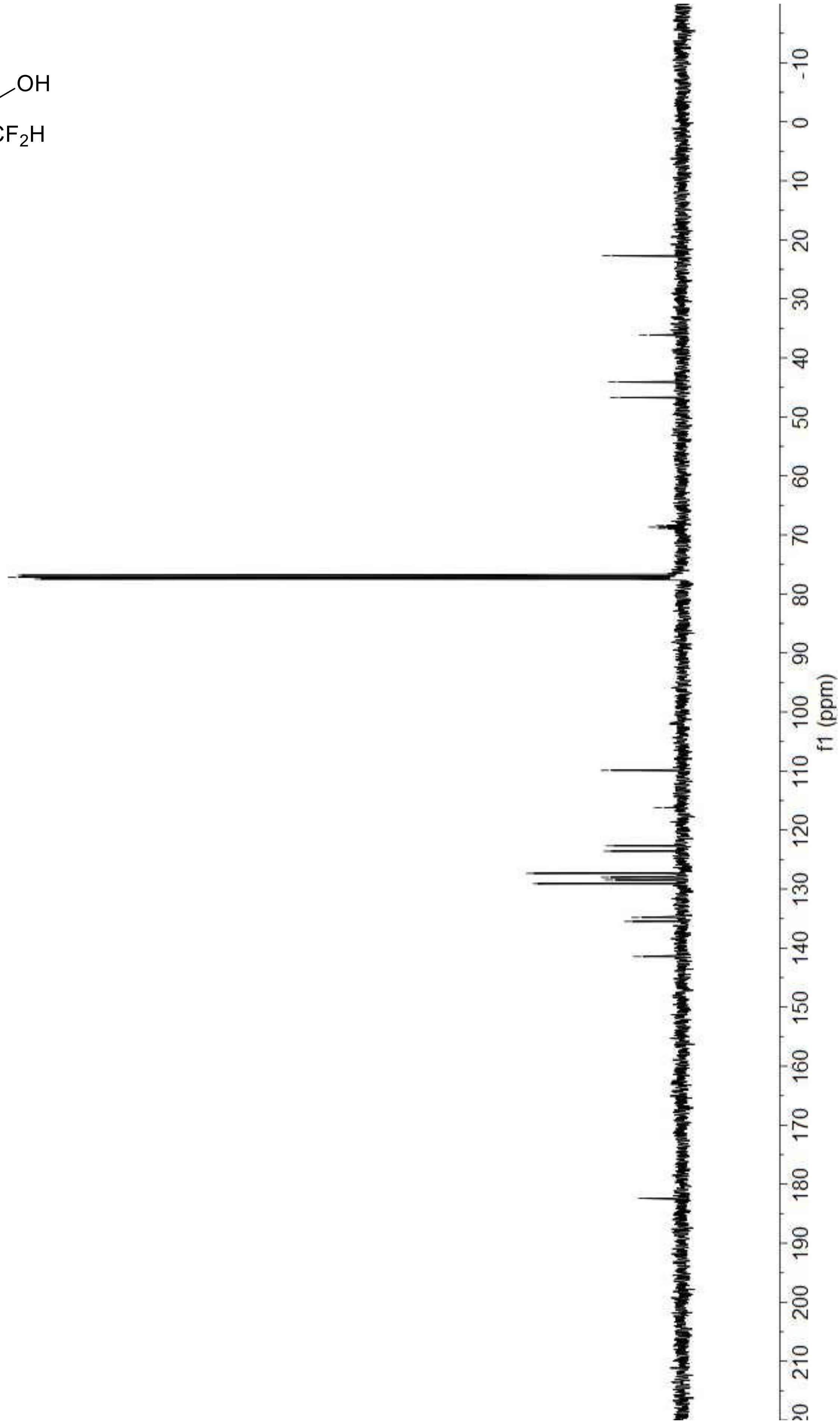

^{19}F NMR of **14g-a** ($CDCl_3$, 375 MHz, 25 ºC)

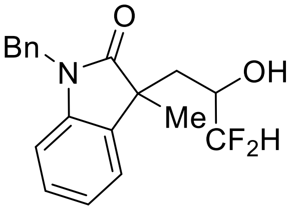

14g-a

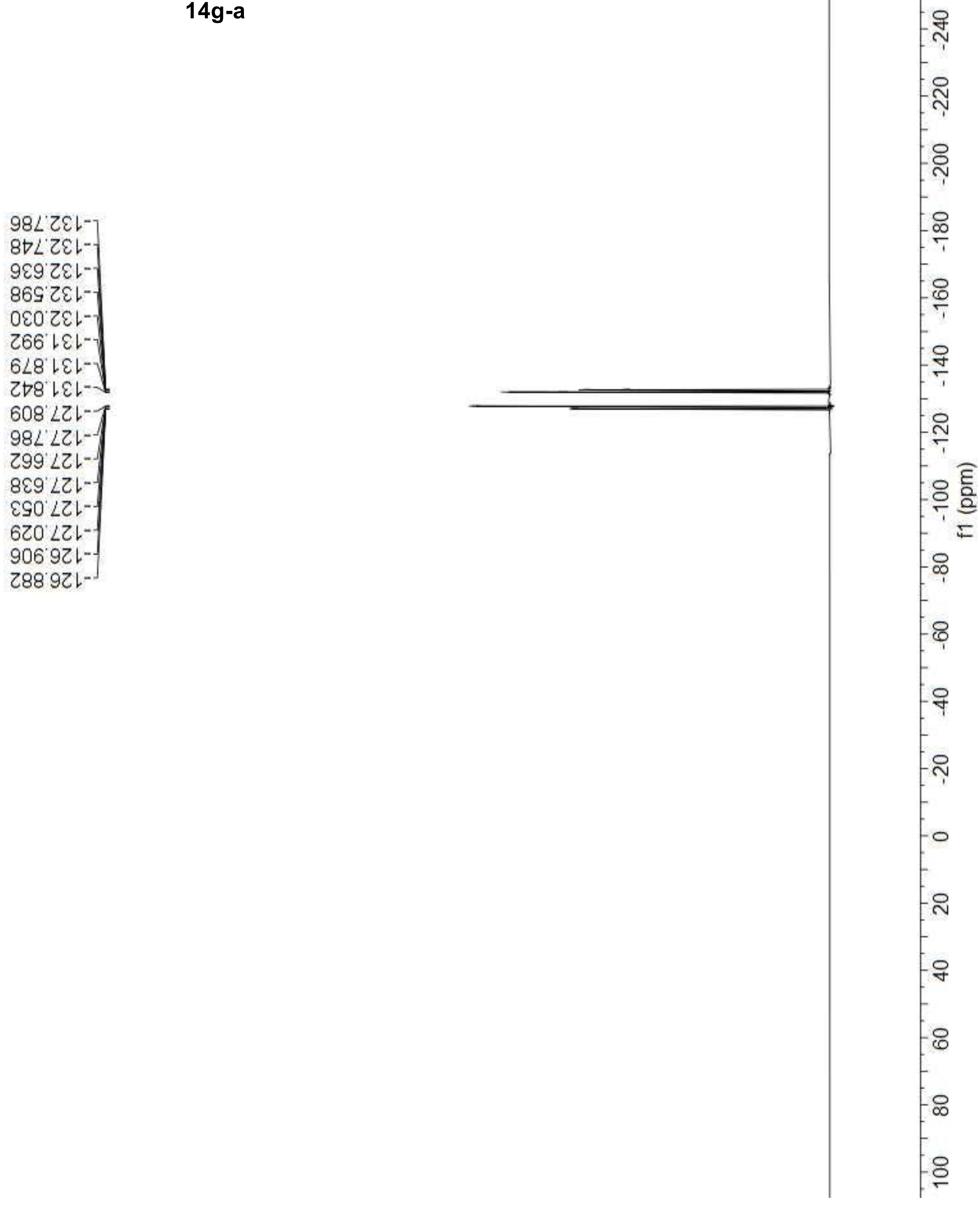

^{1}H NMR of **14g-b** ($CDCl_3$, 400 MHz, 25 ºC)

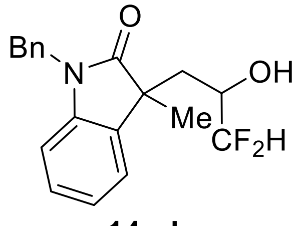

14g-b

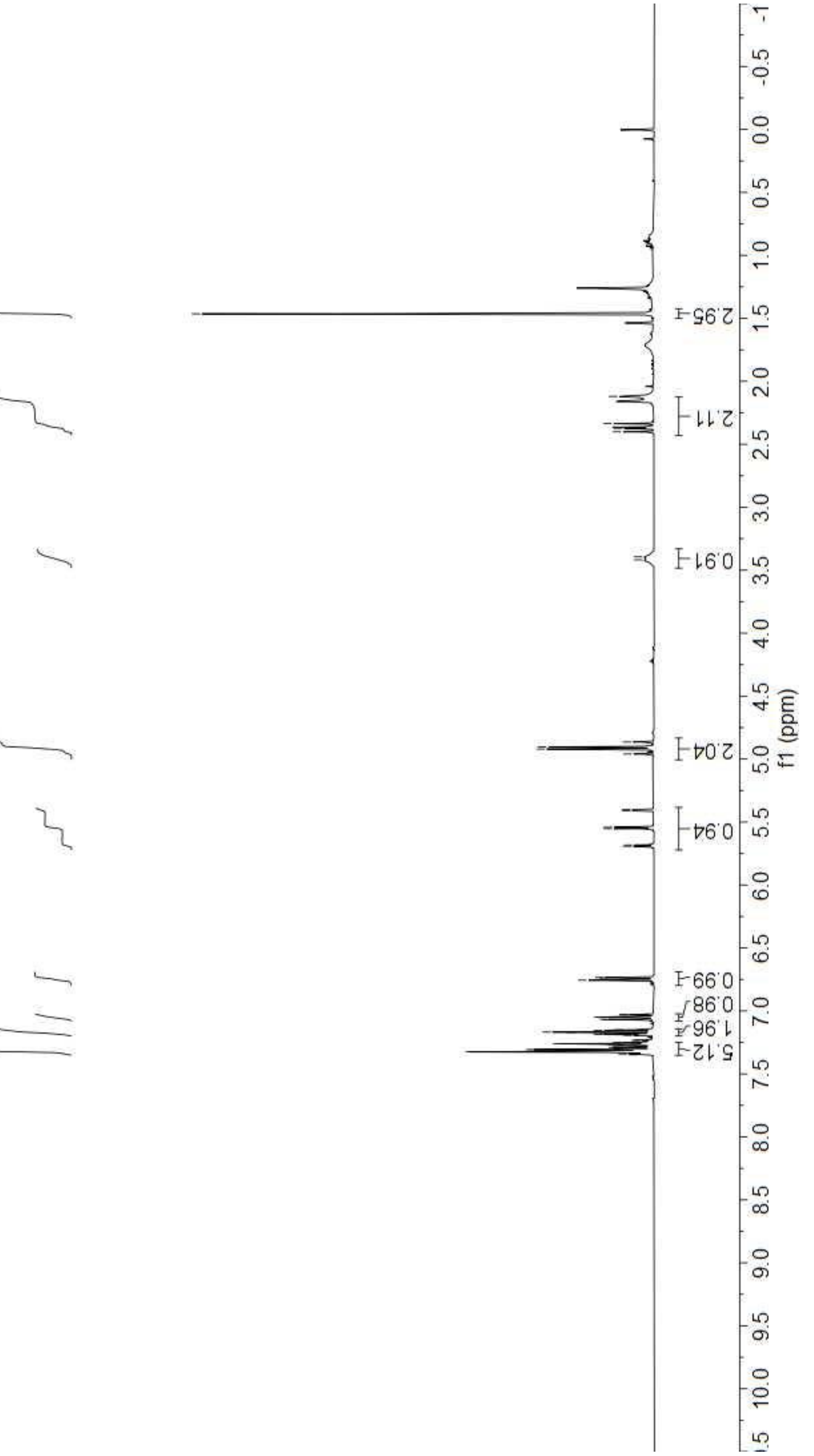

^{13}C NMR of **14g-b** ($CDCl_3$, 100 MHz, 25 ºC)

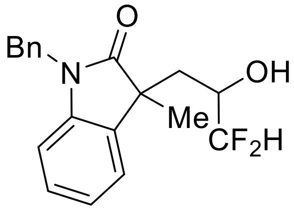

14g-b

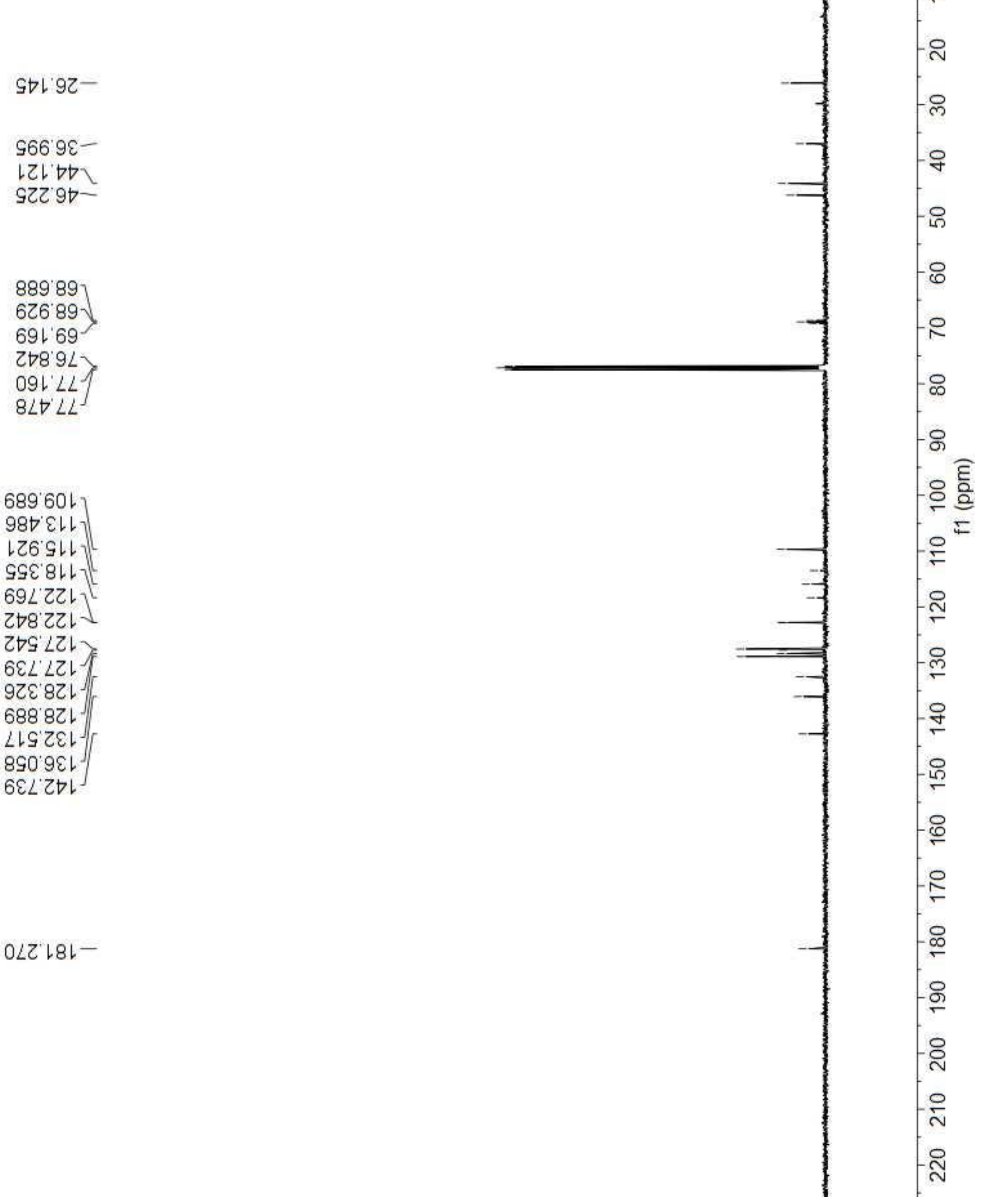

^{19}F NMR of **14g-b** ($CDCl_3$, 375 MHz, 25 ºC)

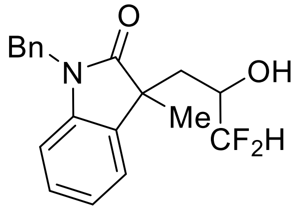

14g-b

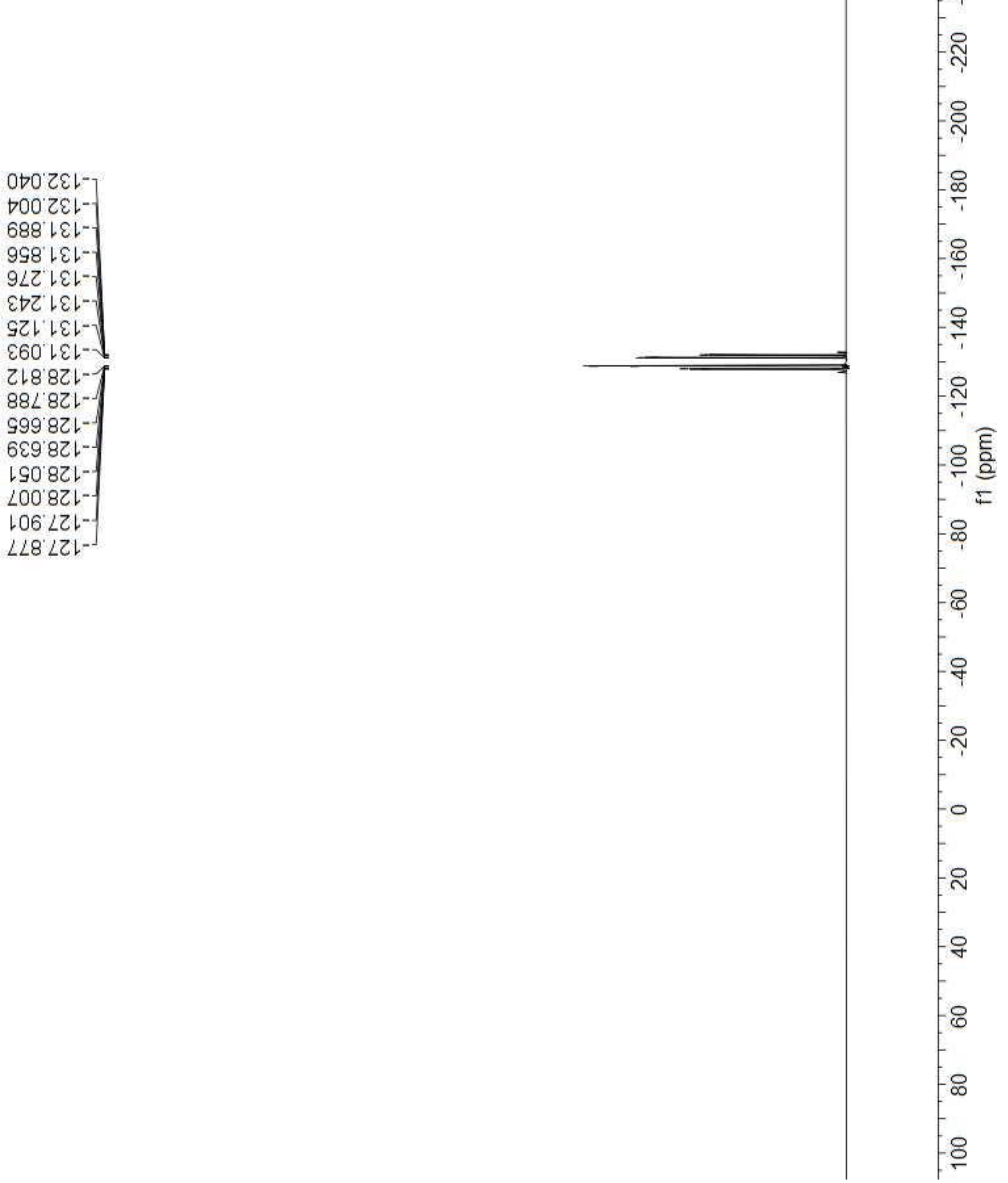

^{1}H NMR of **14h-a** ($CDCl_3$, 400 MHz, 25 ºC)

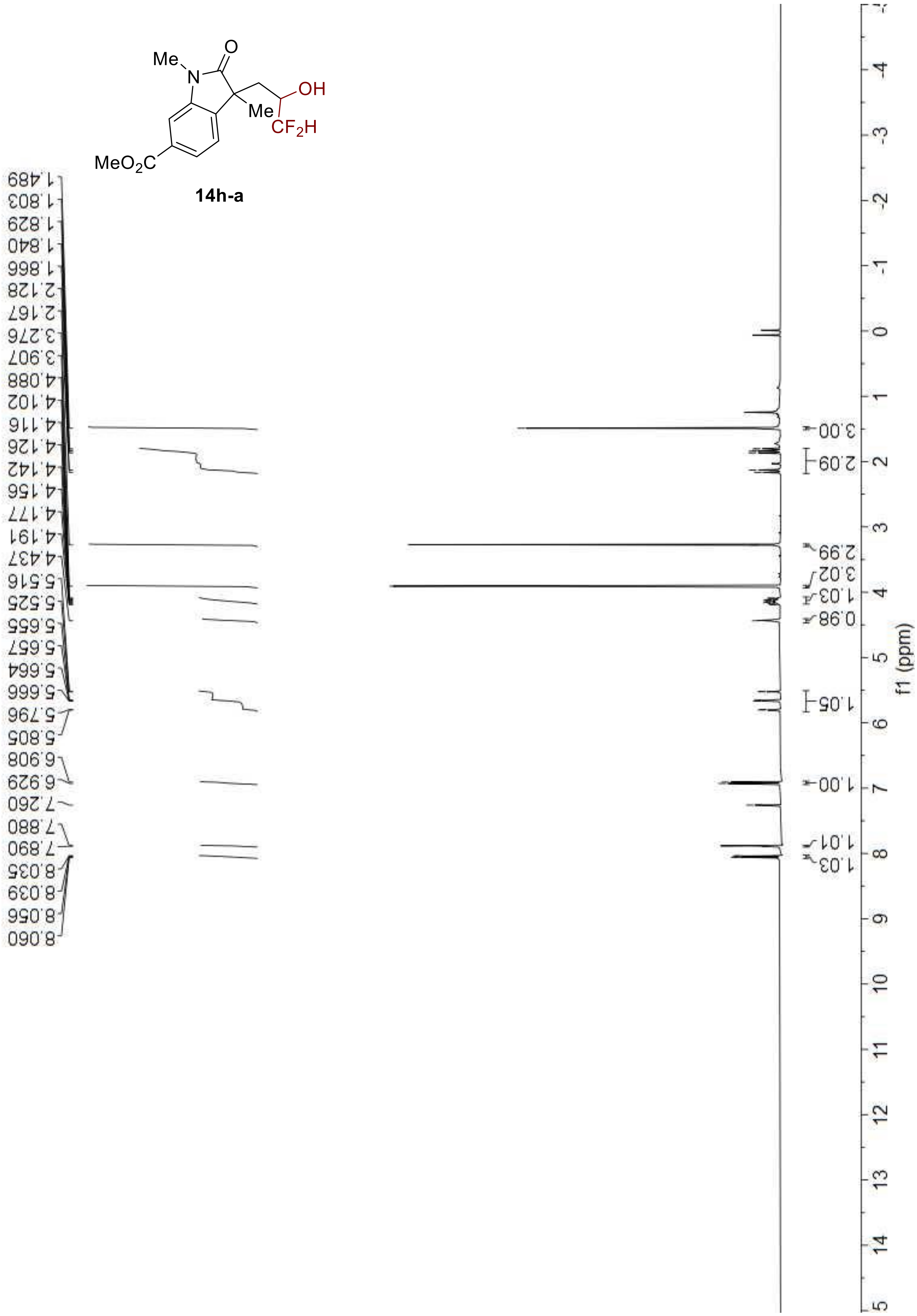

^{13}C NMR of **14h-a** ($CDCl_3$, 100 MHz, 25 ºC)

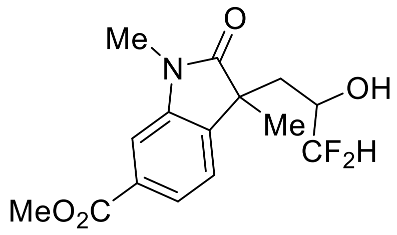

14h-a

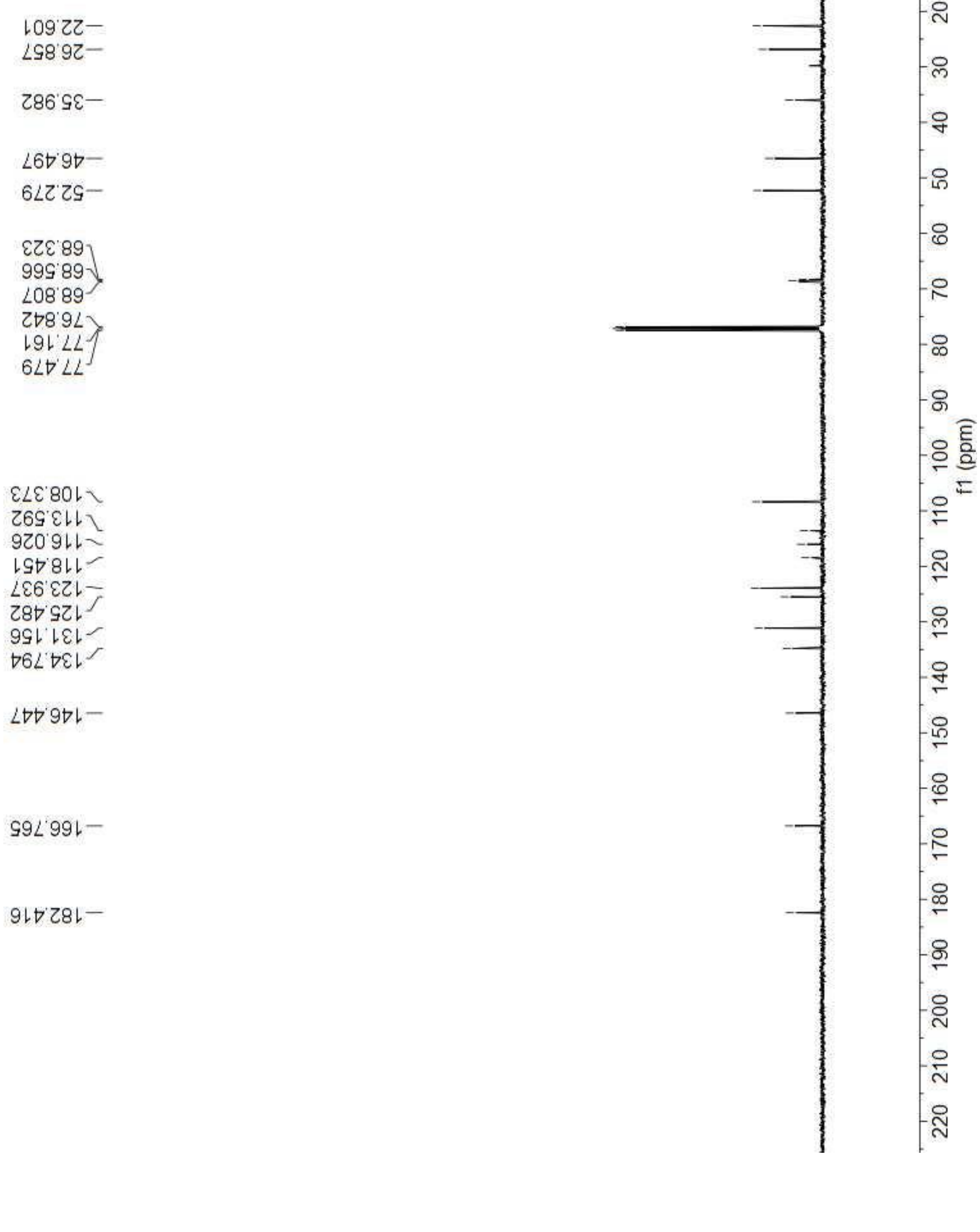

^{19}F NMR of **14h-a** ($CDCl_3$, 375 MHz, 25 ºC)

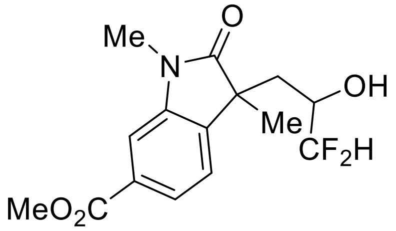

14h-a

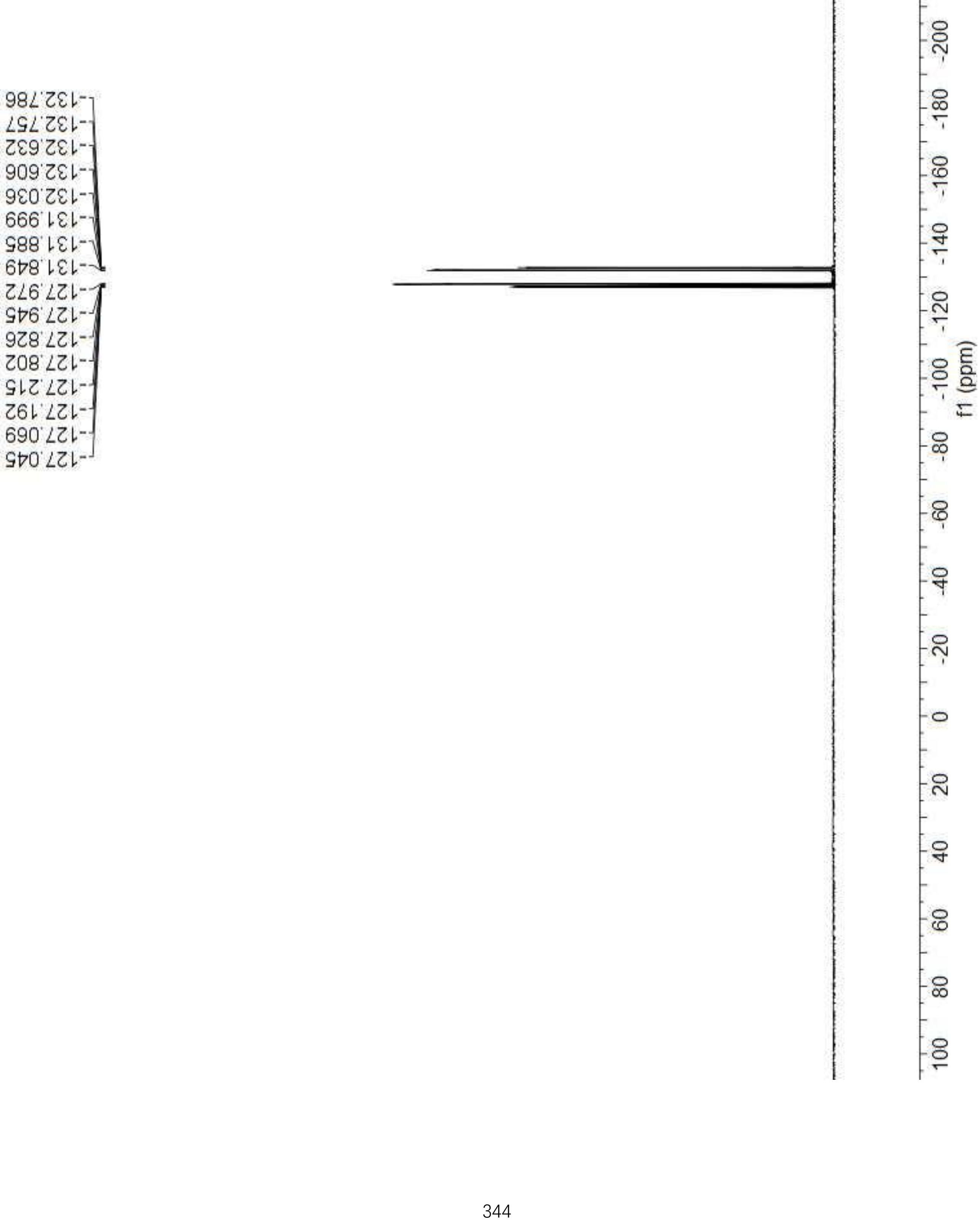

^{1}H NMR of **14h-b** ($CDCl_3$, 400 MHz, 25 ºC)

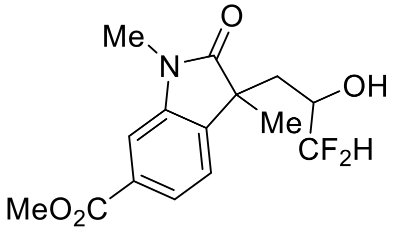

14h-b

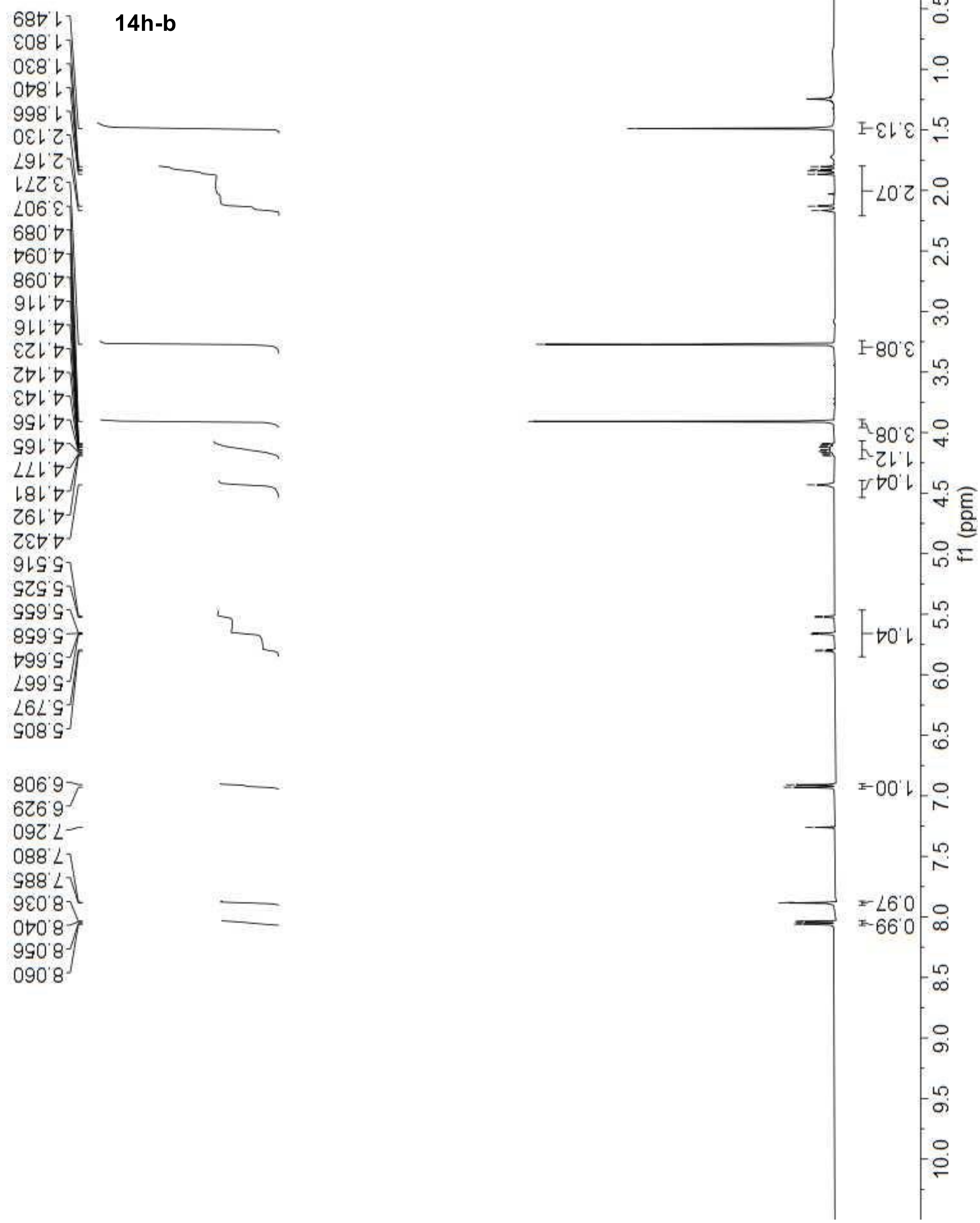

^{13}C NMR of **14h-b** ($CDCl_3$, 100 MHz, 25 ºC)

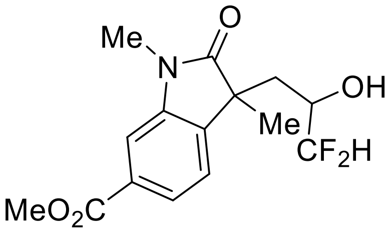

14h-b

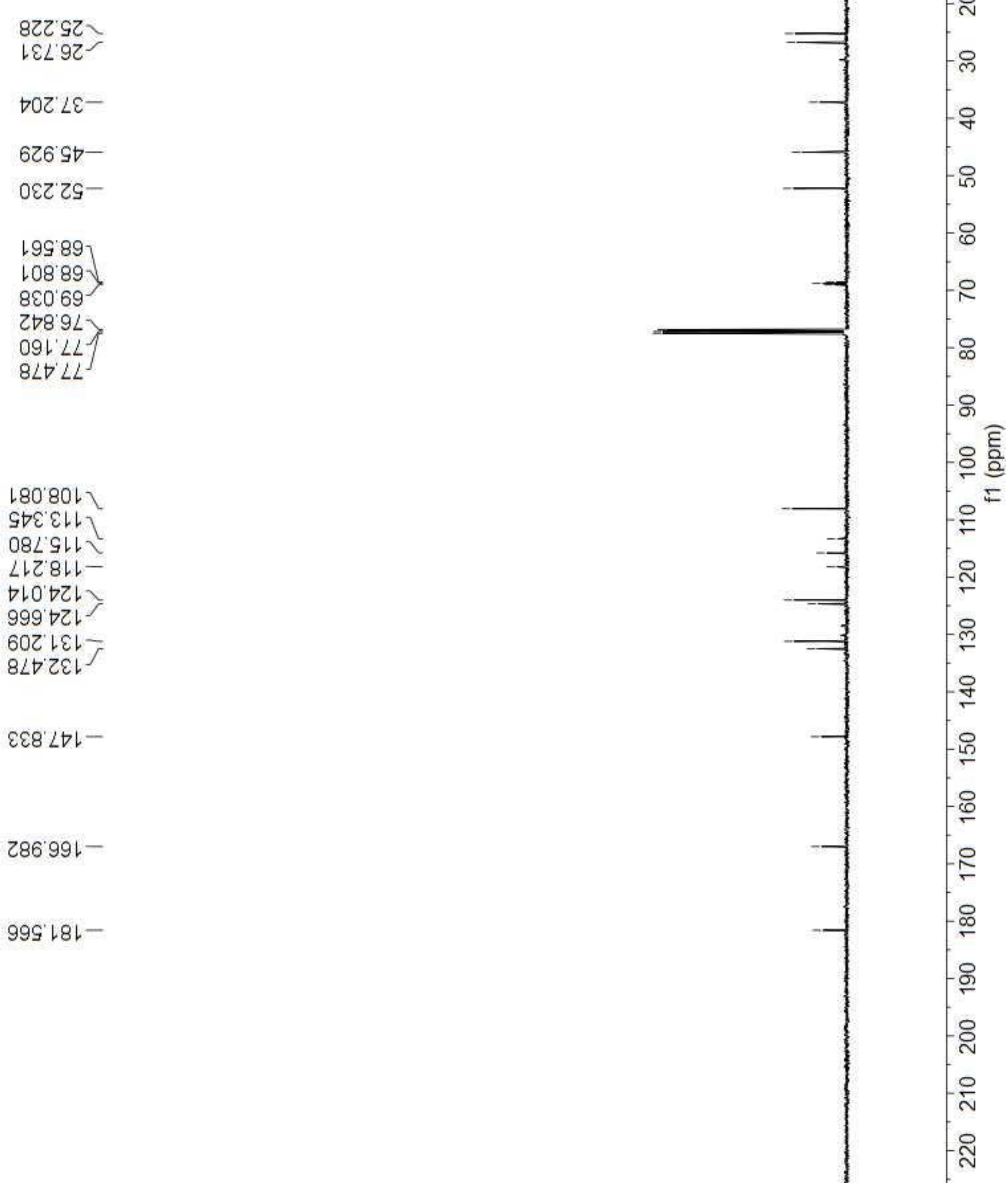

^{19}F NMR of **14h-b** ($CDCl_3$, 375 MHz, 25 ºC)

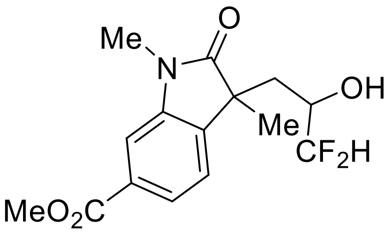

14h-b

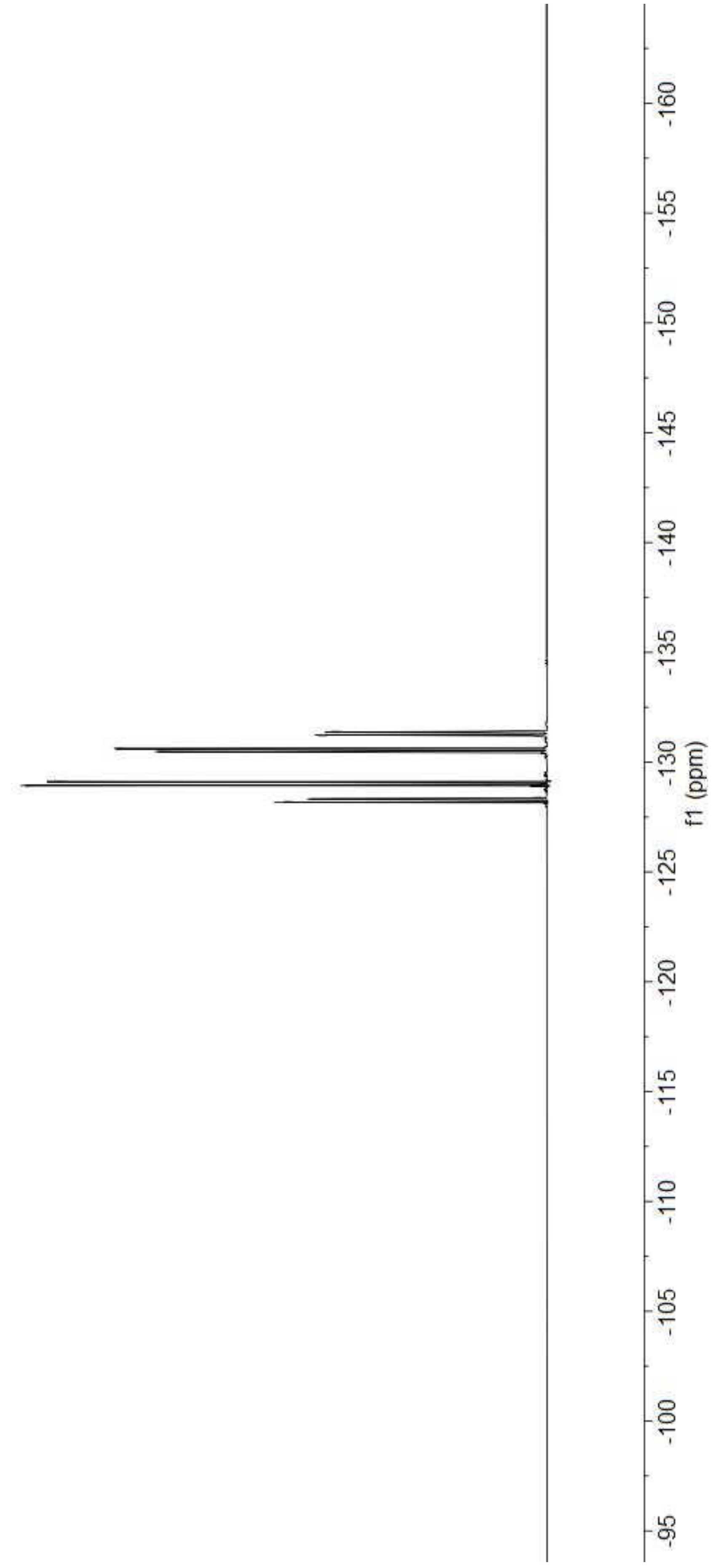

^{1}H NMR of **14i-a** ($CDCl_3$, 400 MHz, 25 ºC)

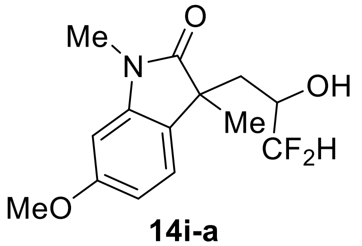

14i-a

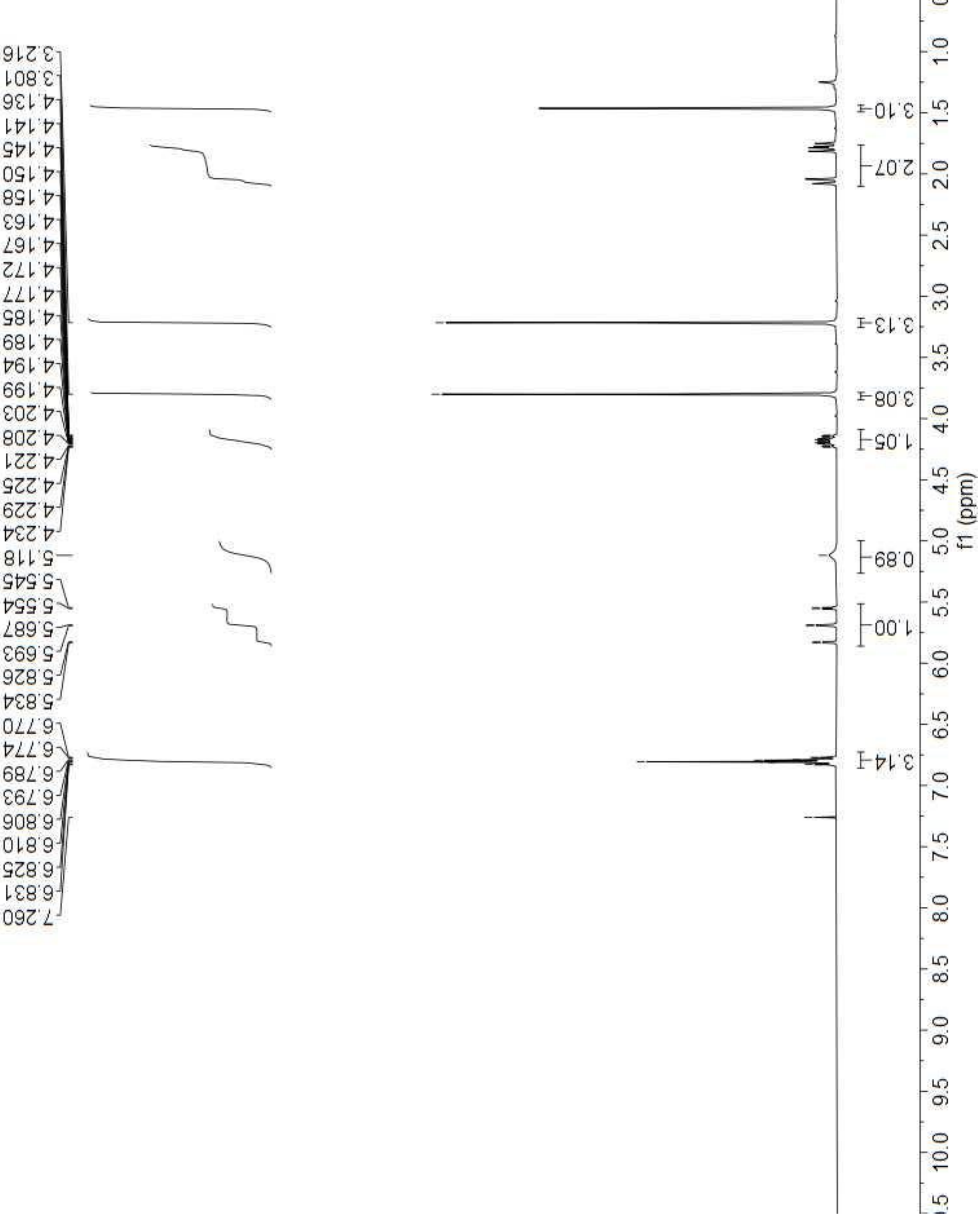

^{13}C NMR of **14i-a** ($CDCl_3$, 100 MHz, 25 ºC)

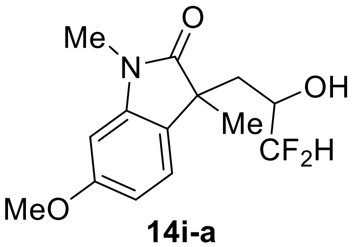

14i-a

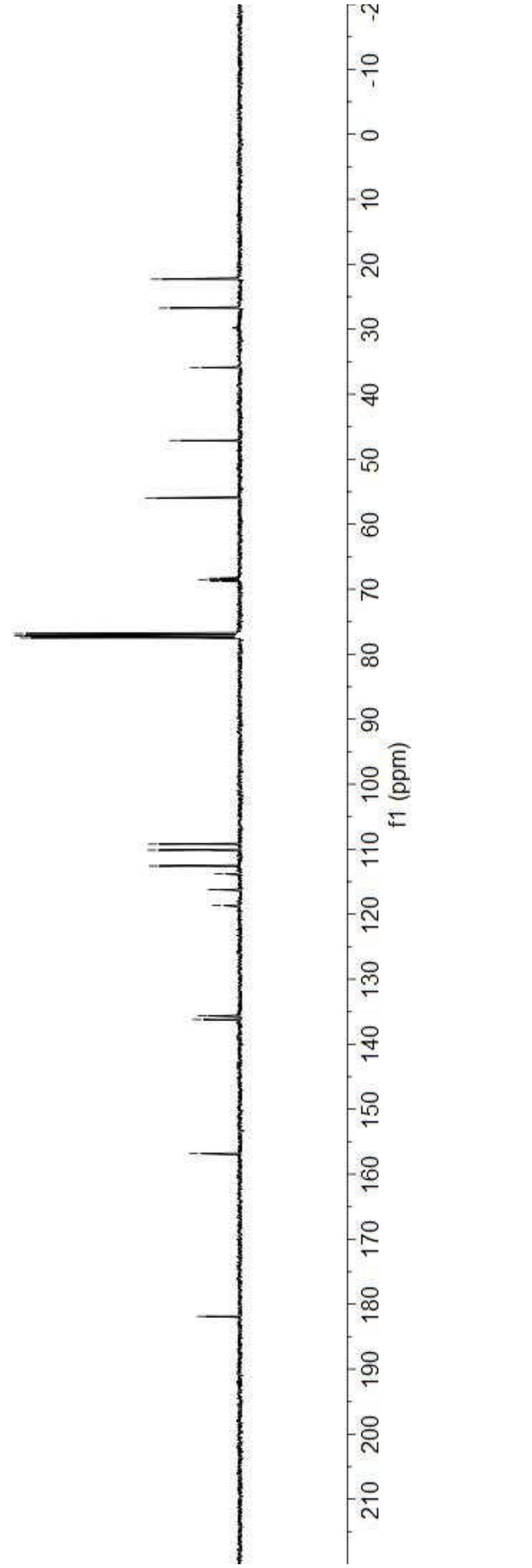

^{19}F NMR of **14i-a** ($CDCl_3$, 375 MHz, 25 ºC)

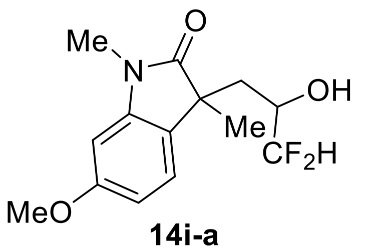

14i-a

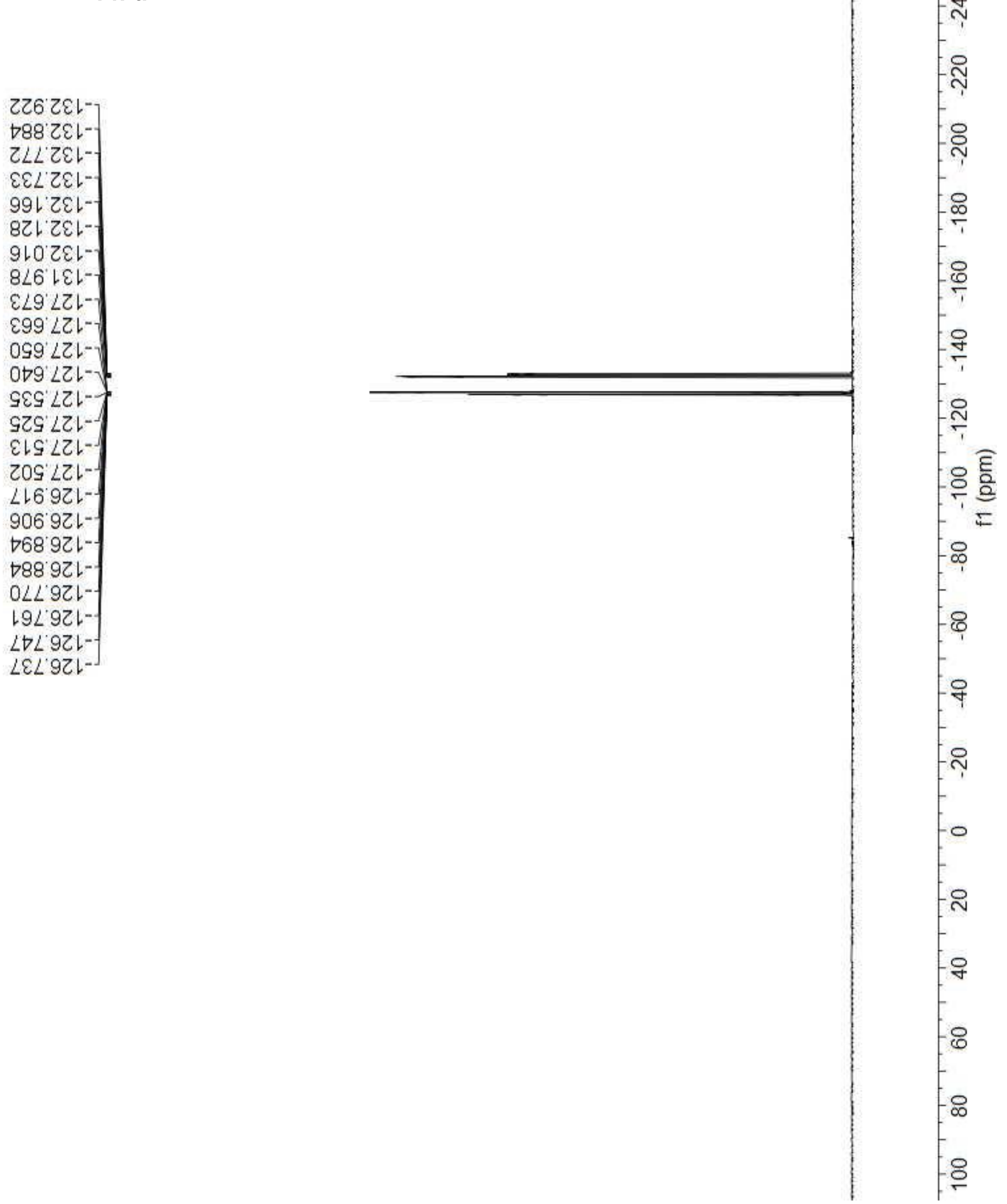

^{1}H NMR of **14i-b** ($CDCl_3$, 400 MHz, 25 ºC)

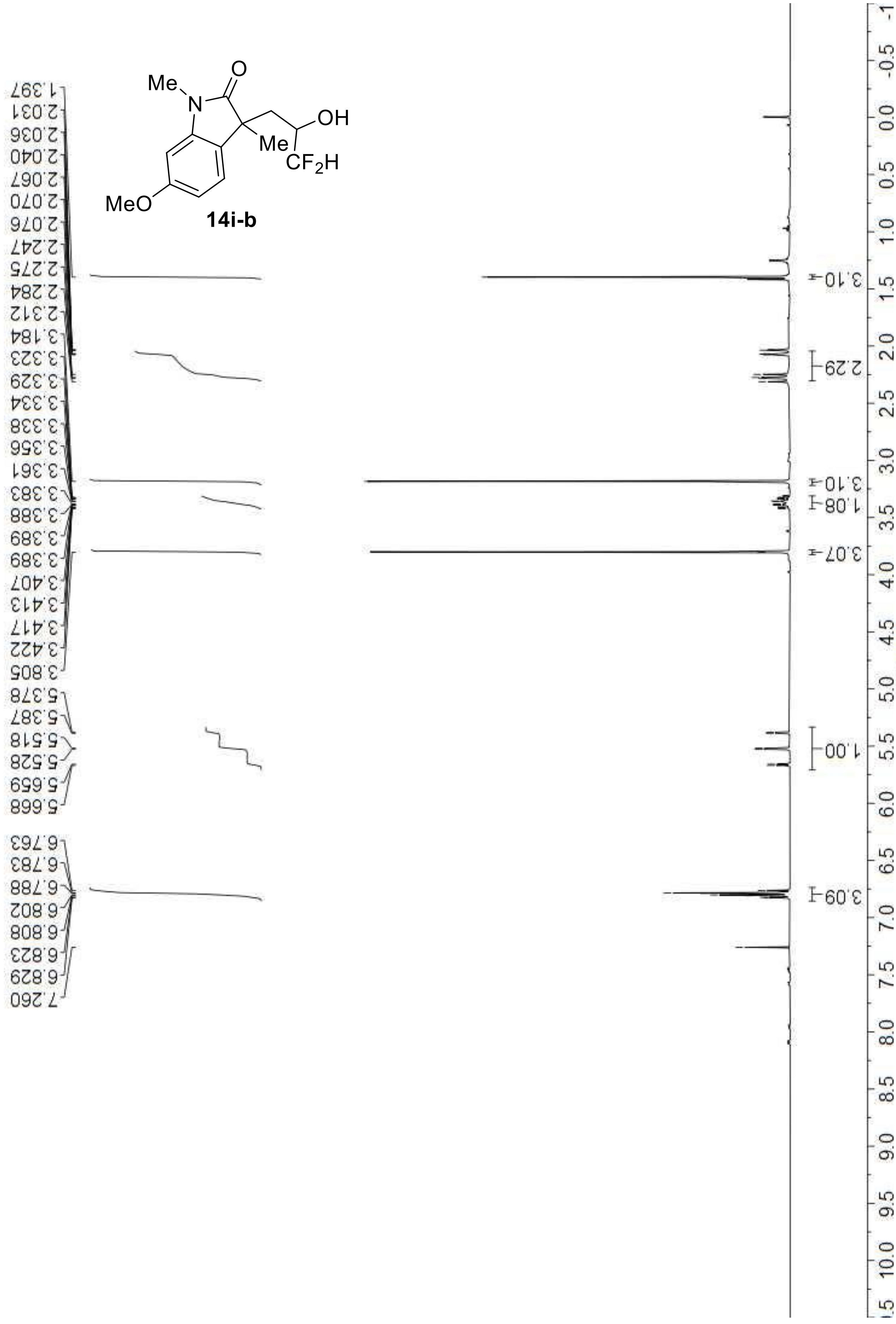

^{13}C NMR of **14i-b** ($CDCl_3$, 100 MHz, 25 ºC)

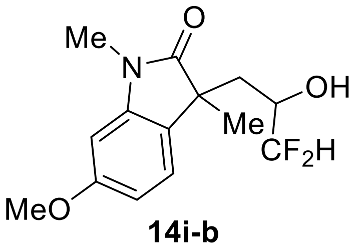

14i-b

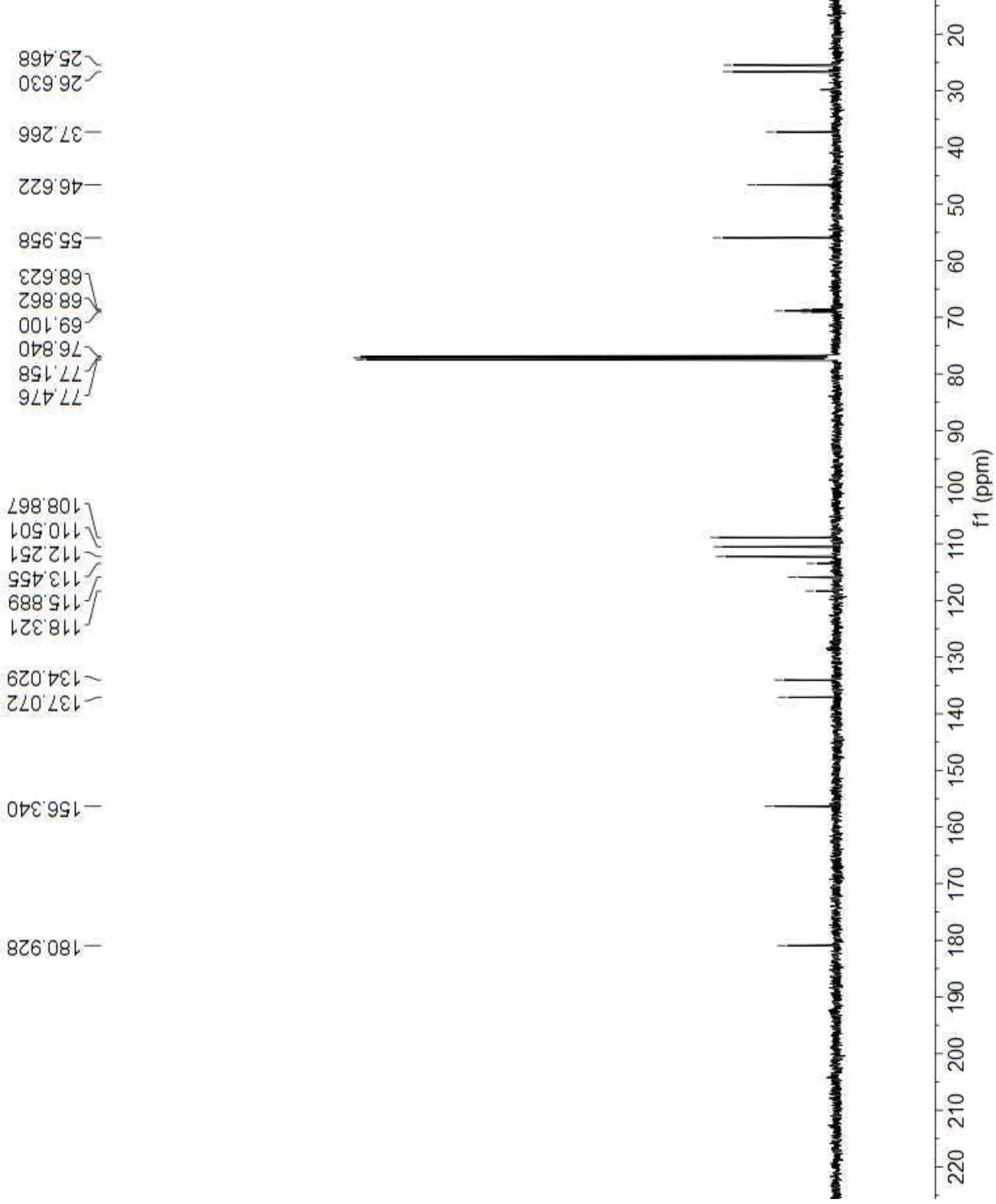

^{19}F NMR of **14i-b** ($CDCl_3$, 375 MHz, 25 ºC)

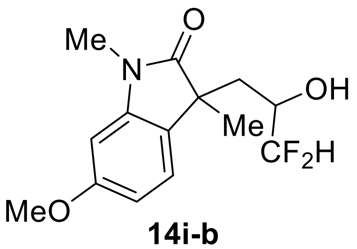

14i-b

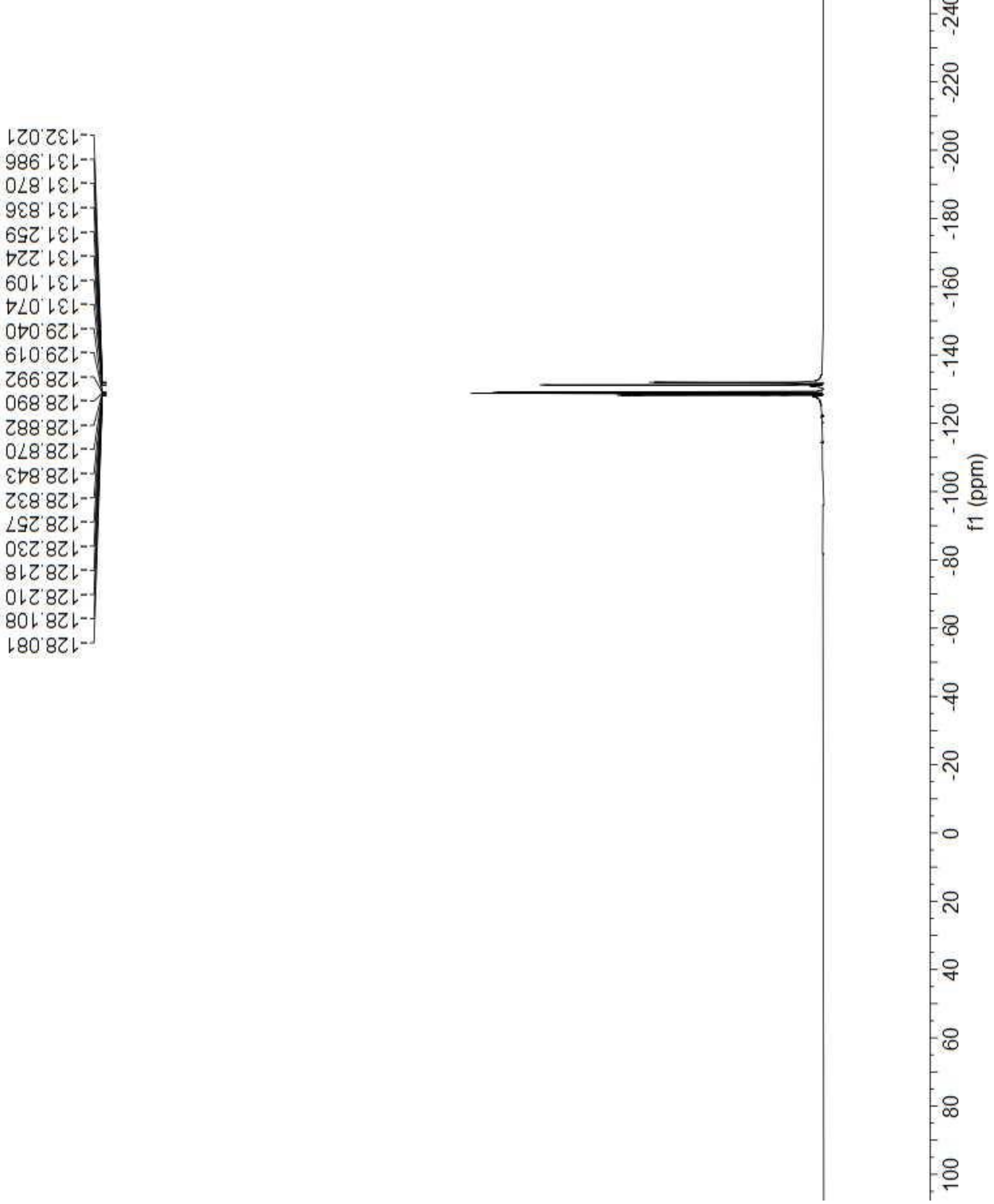

^{1}H NMR of **14j** ($CDCl_3$, 400 MHz, 25 ºC)

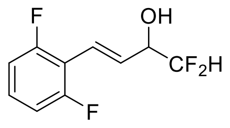

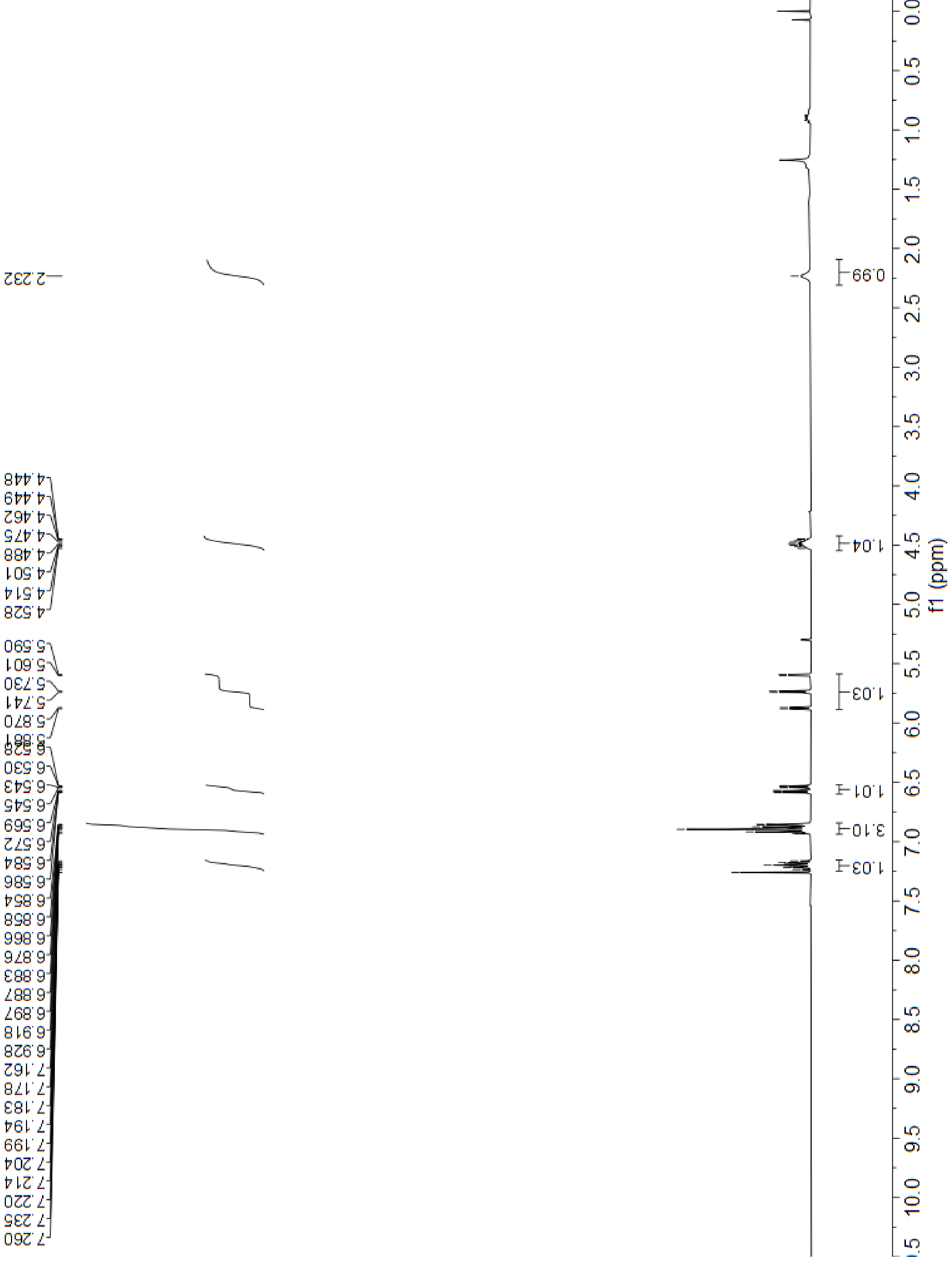

^{13}C NMR of **14j** ($CDCl_3$, 150 MHz, 25 ºC)

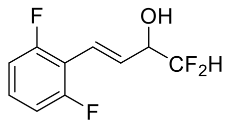

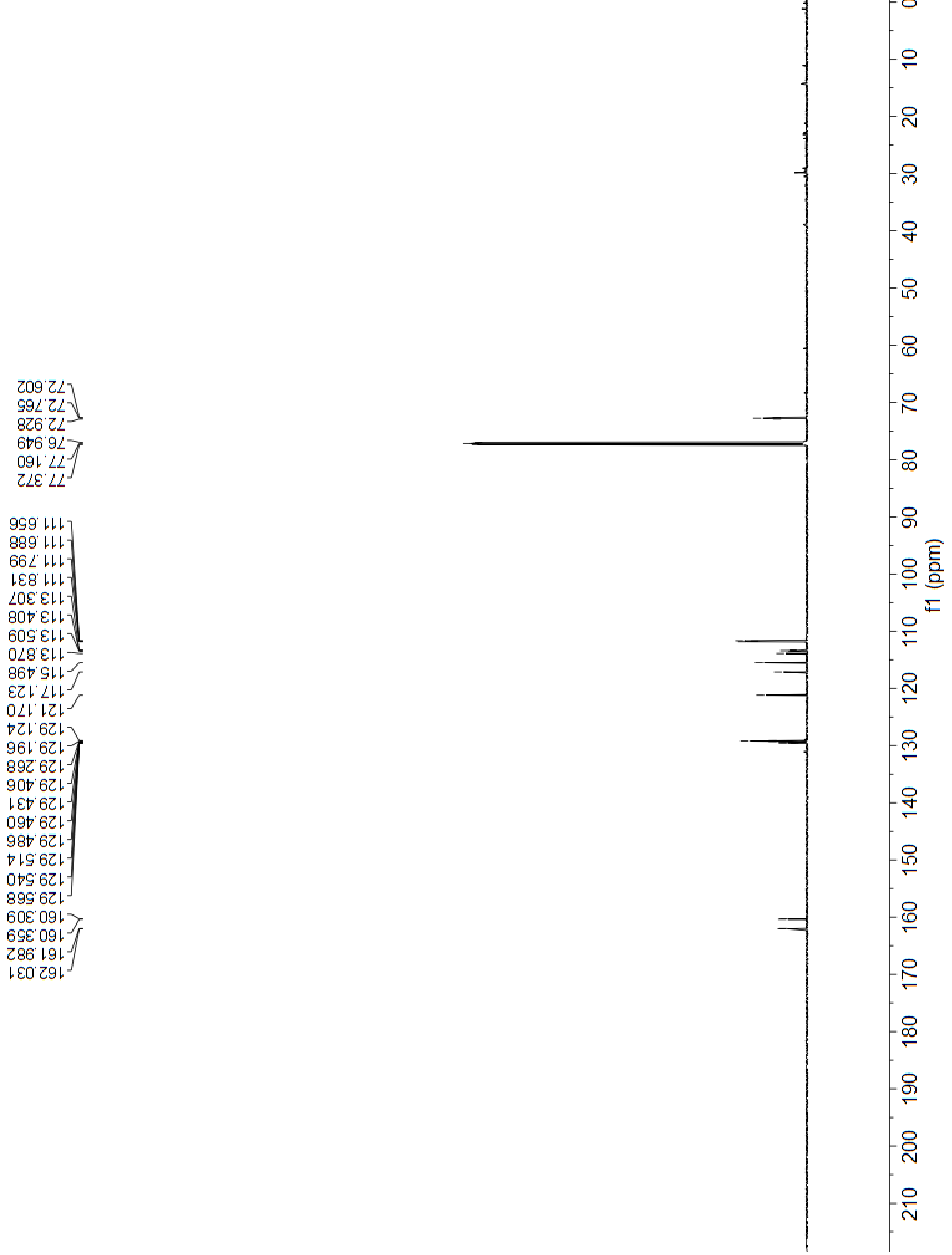

^{19}F NMR of **14j** ($CDCl_3$, 375 MHz, 25 ºC)

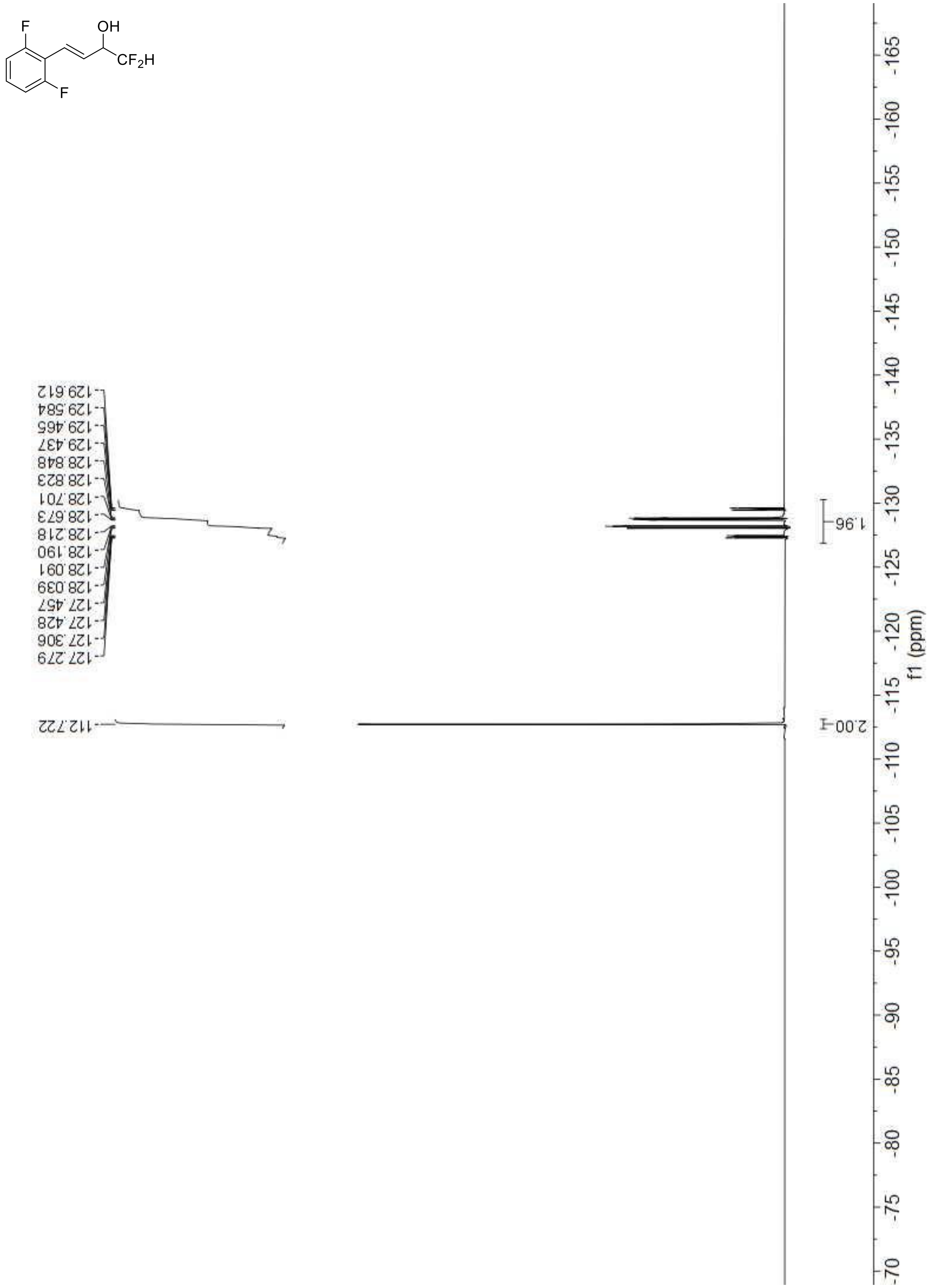

^{1}H NMR of **14k** ($CDCl_3$, 400 MHz, 25 ºC)

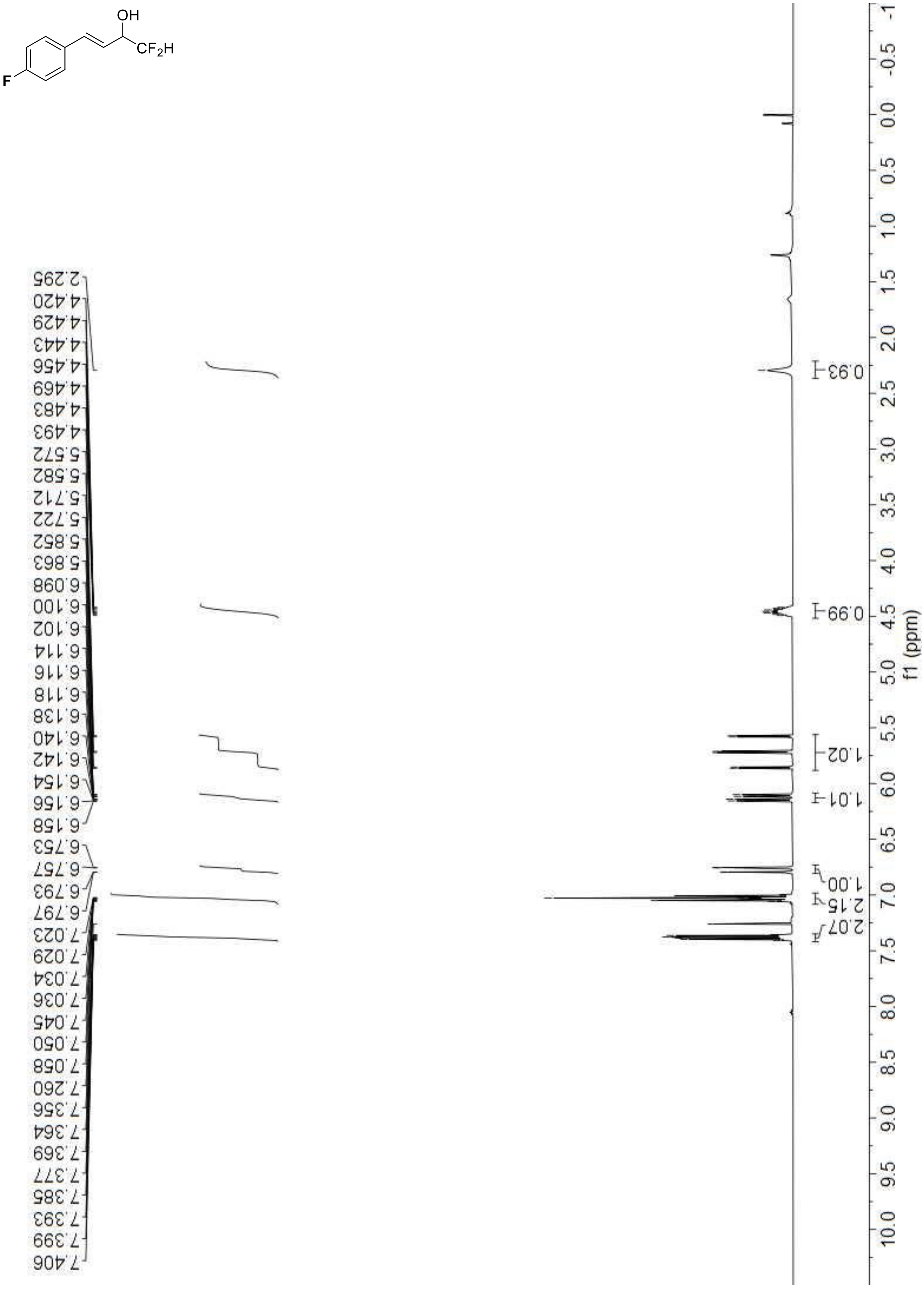

^{13}C NMR of **14k** ($CDCl_3$, 100 MHz, 25 ºC)

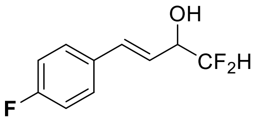

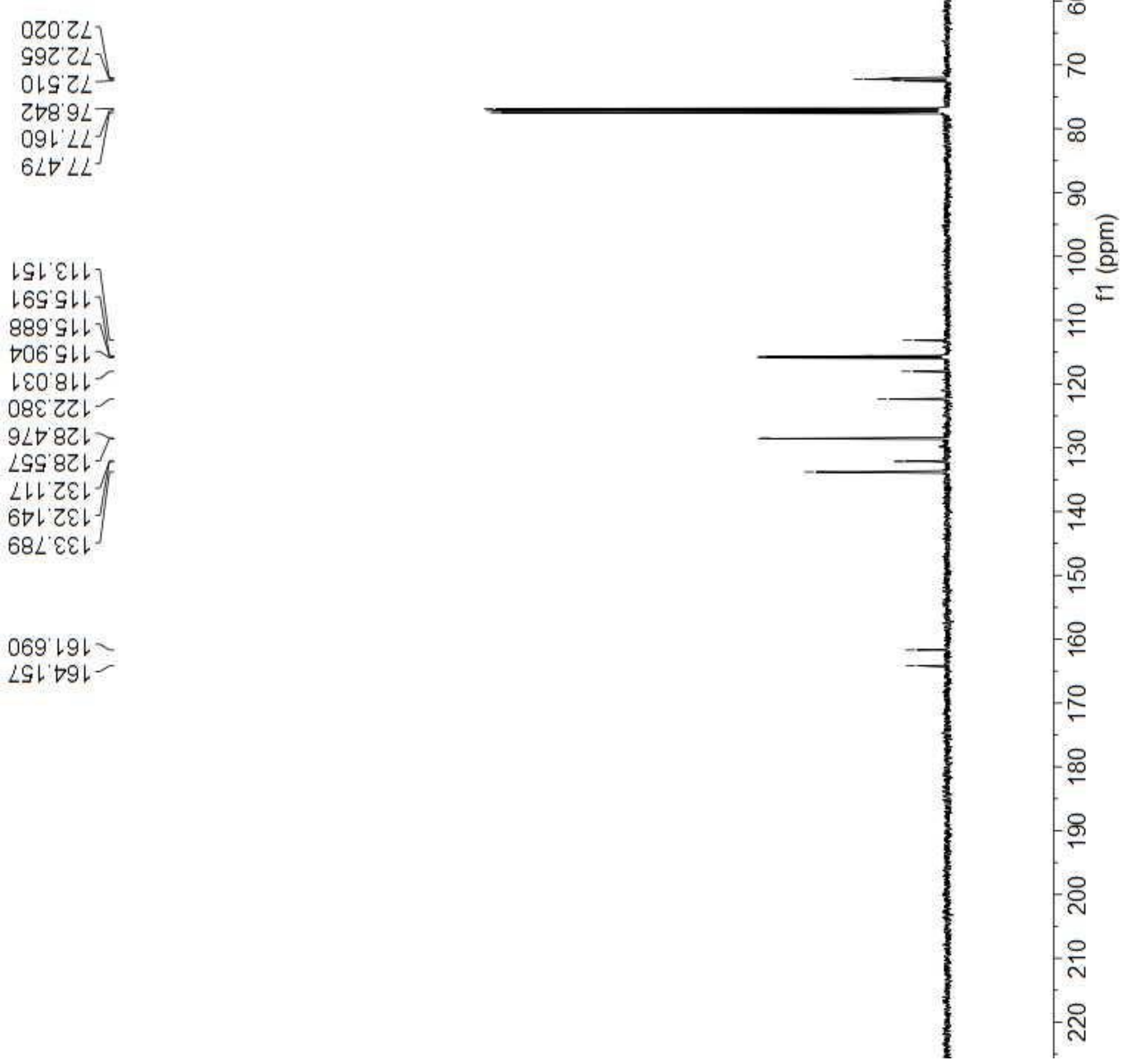

^{19}F NMR of **14k** ($CDCl_3$, 375 MHz, 25 ºC)

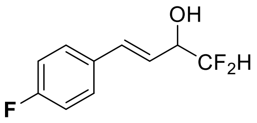

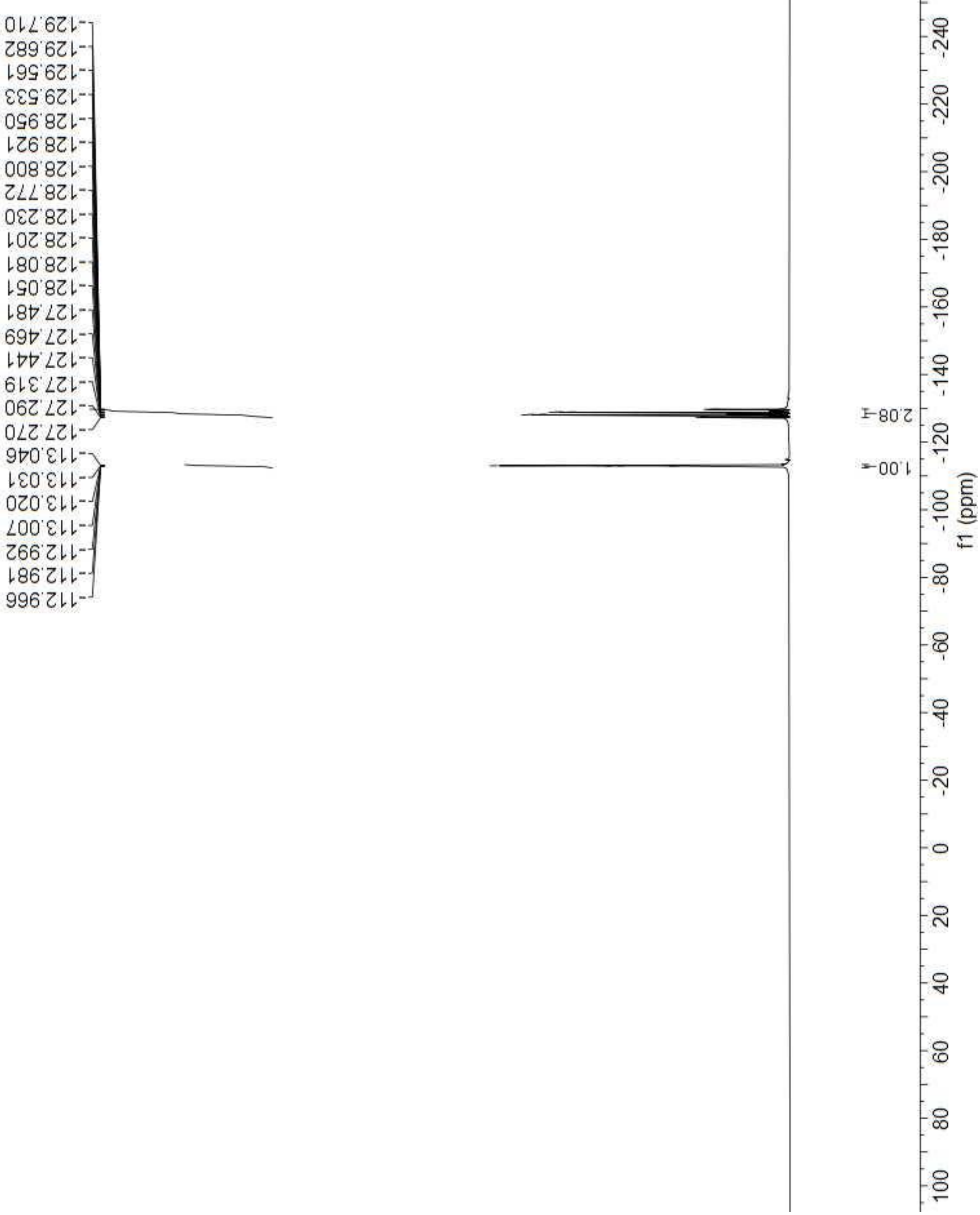

^{1}H NMR of **14l** ($CDCl_3$, 400 MHz, 25 ºC)

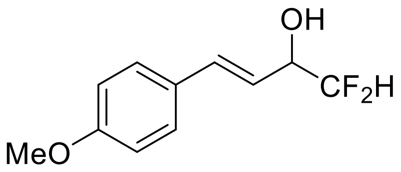

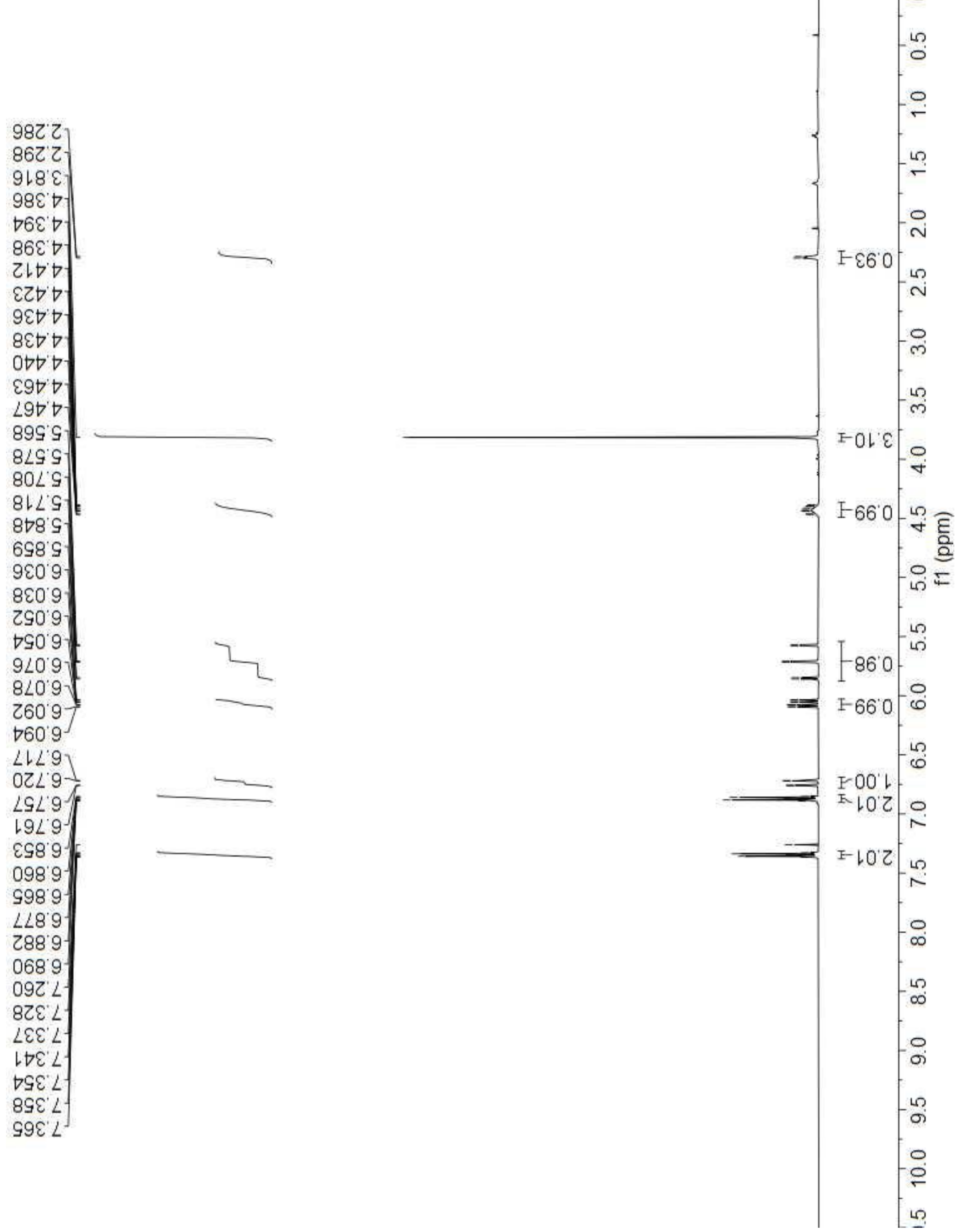

^{13}C NMR of **14l** ($CDCl_3$, 100 MHz, 25 °C)

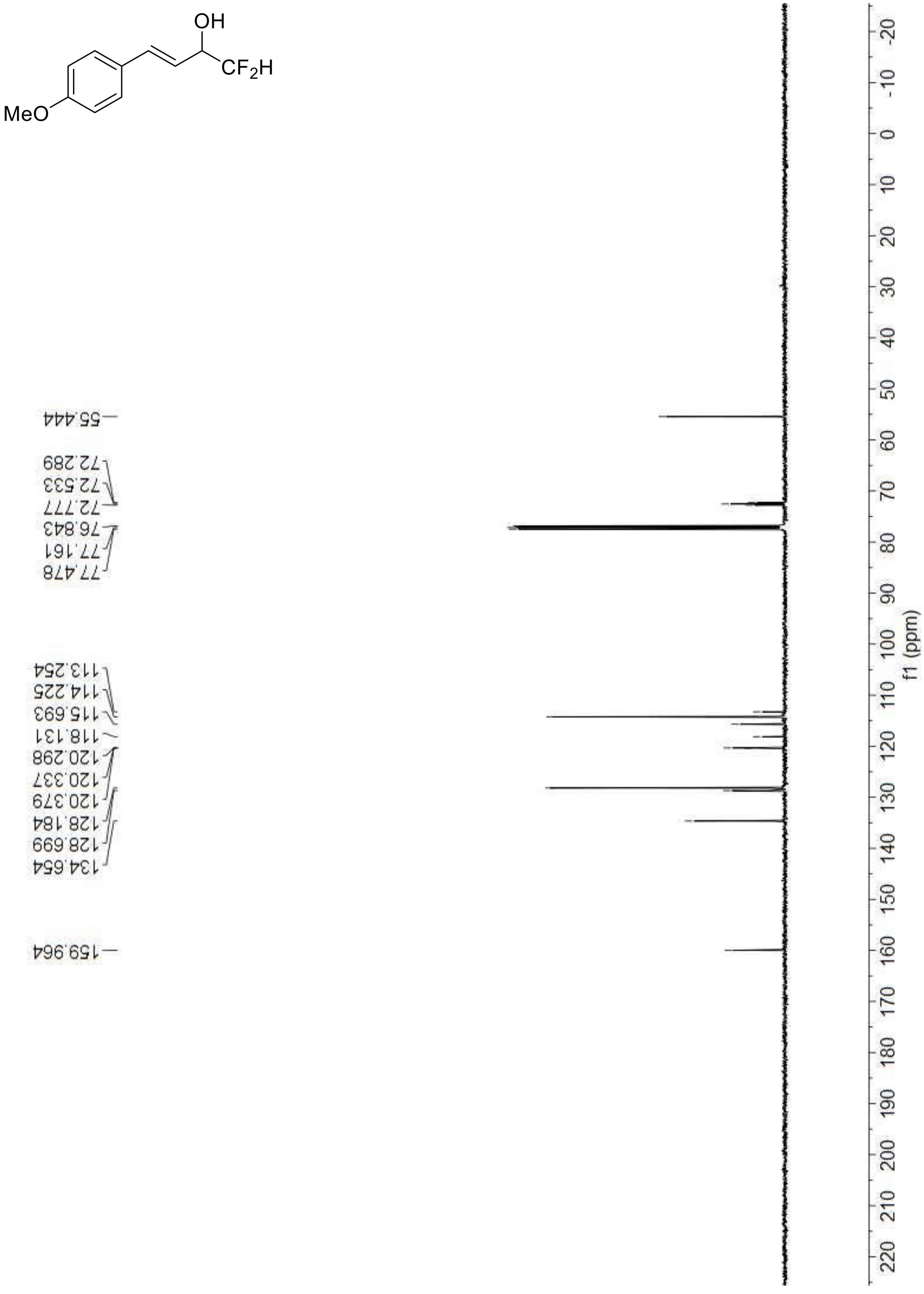

^{19}F NMR of **14l** ($CDCl_3$, 375 MHz, 25 ºC)

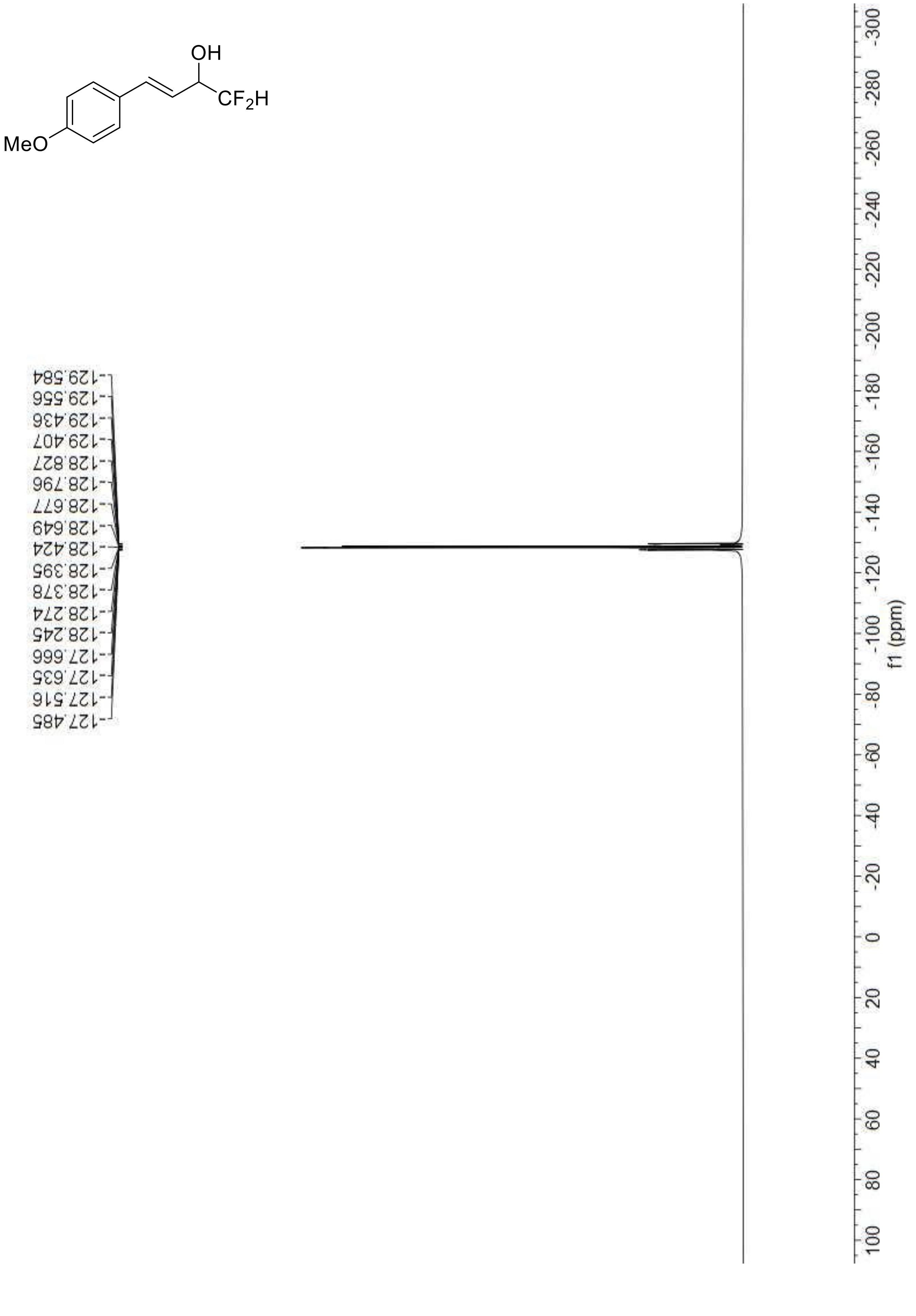

^{1}H NMR of **17** (DMSO-d6, 600 MHz, 25 °C)

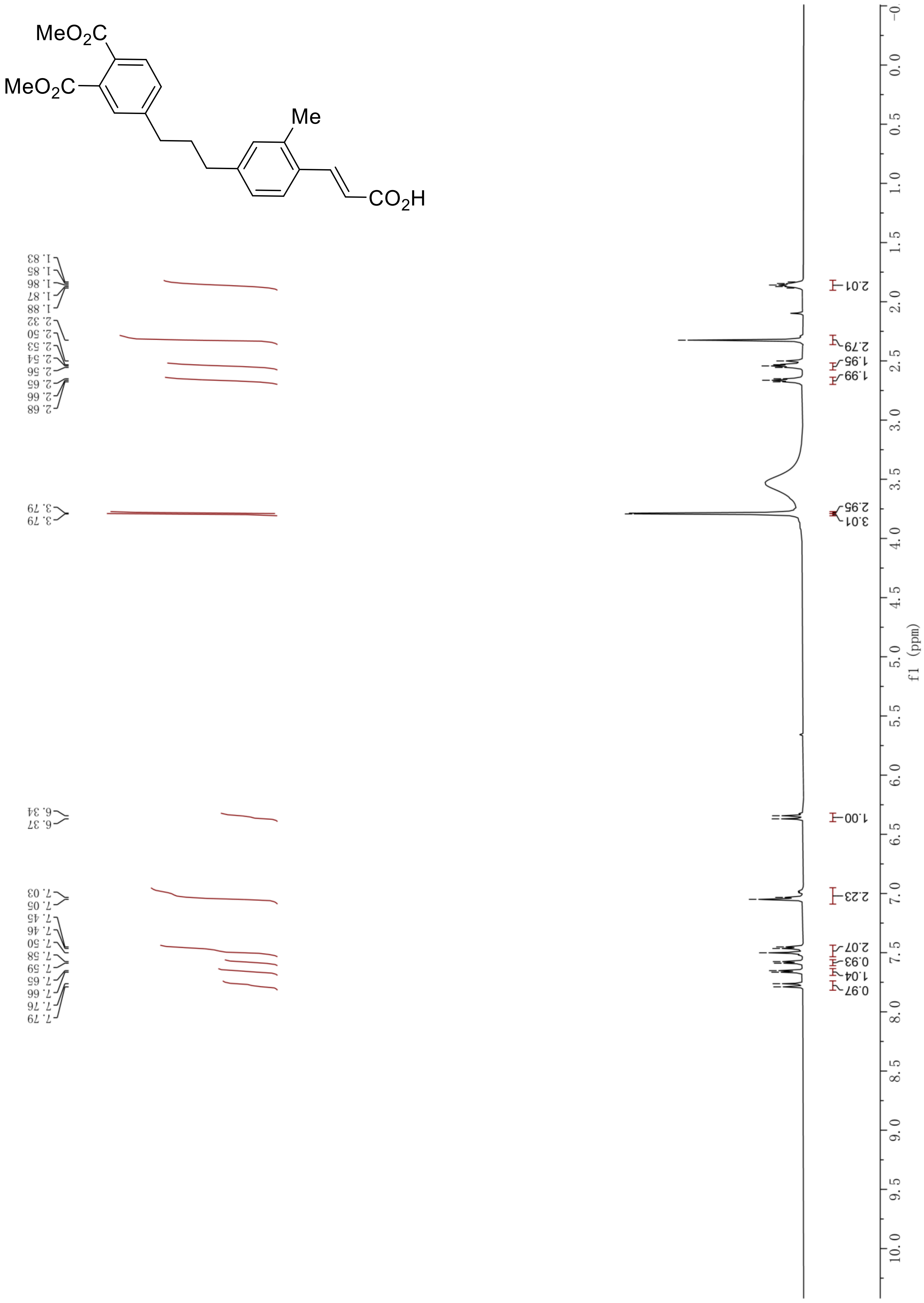

^{13}C NMR of **17** (DMSO-d6, 150 MHz, 25 ºC)

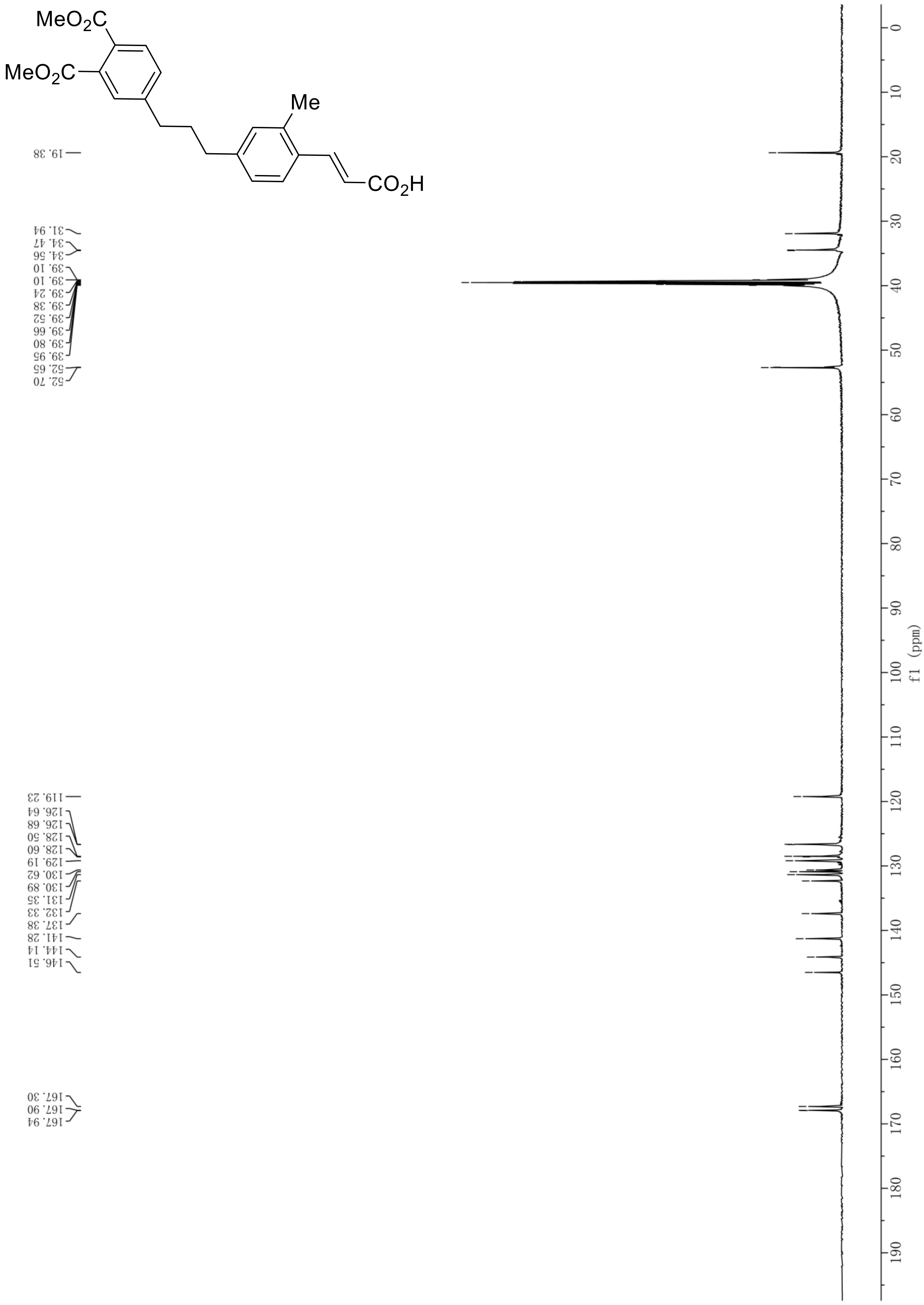

^{1}H NMR of **18** ($CDCl_3$, 400 MHz, 25 ºC)

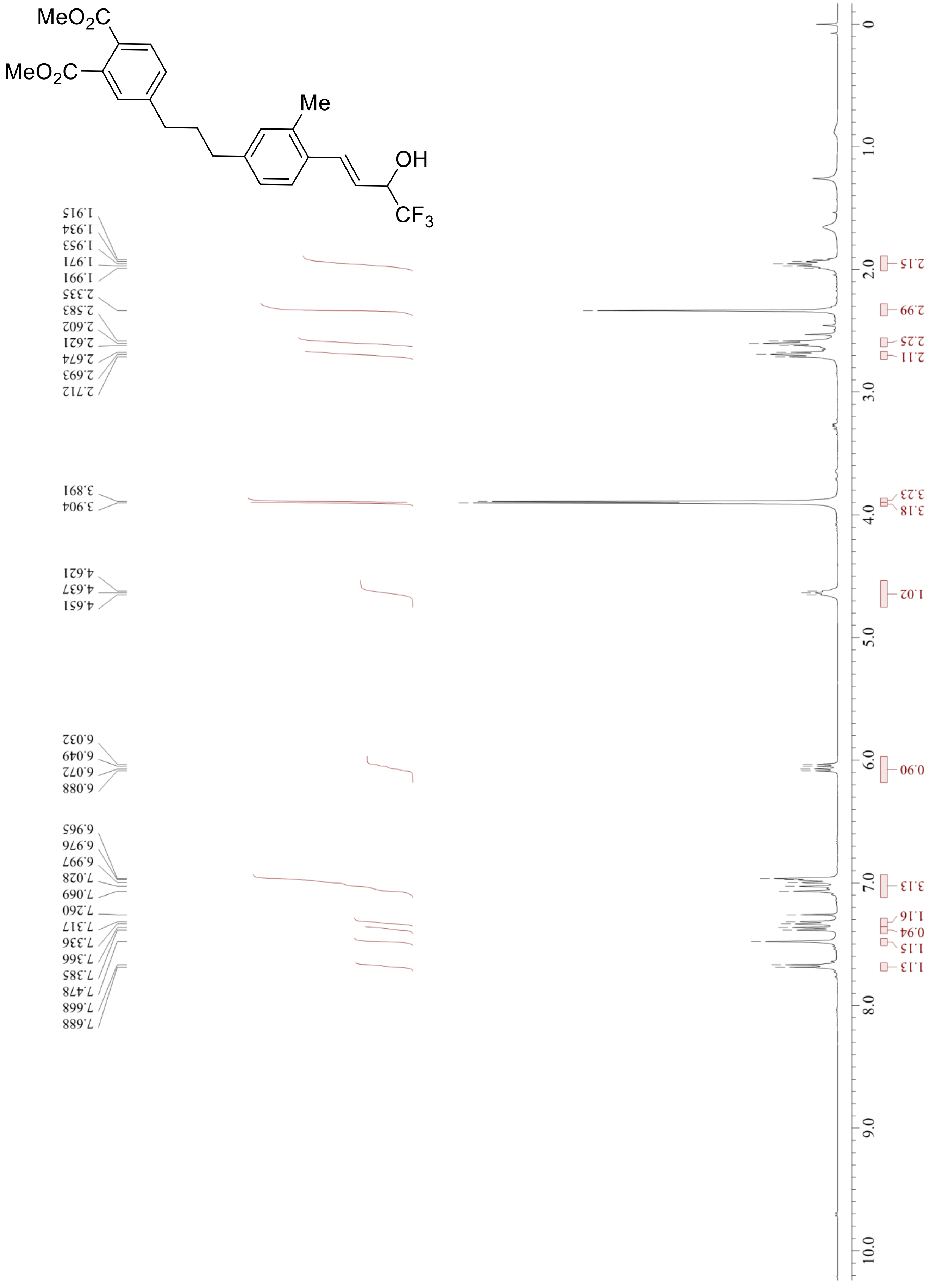

^{13}C NMR of **18** ($CDCl_3$, 100 MHz, 25 ºC)

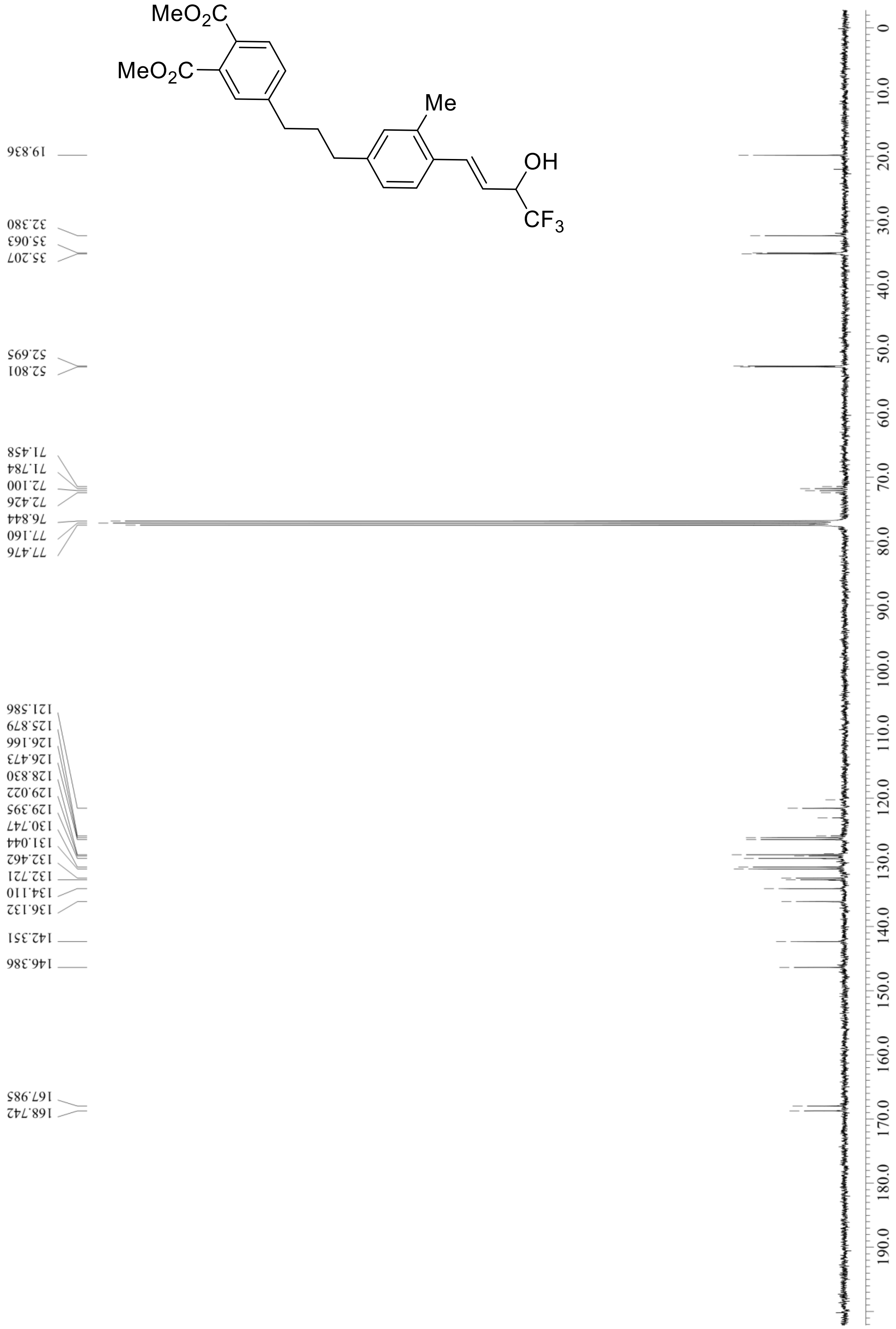

^{19}F NMR of **18** ($CDCl_3$, 375 MHz, 25 ºC)

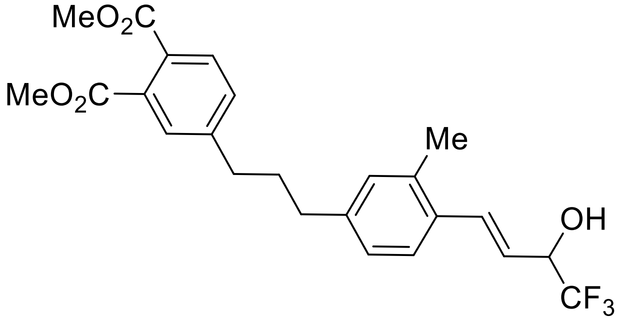

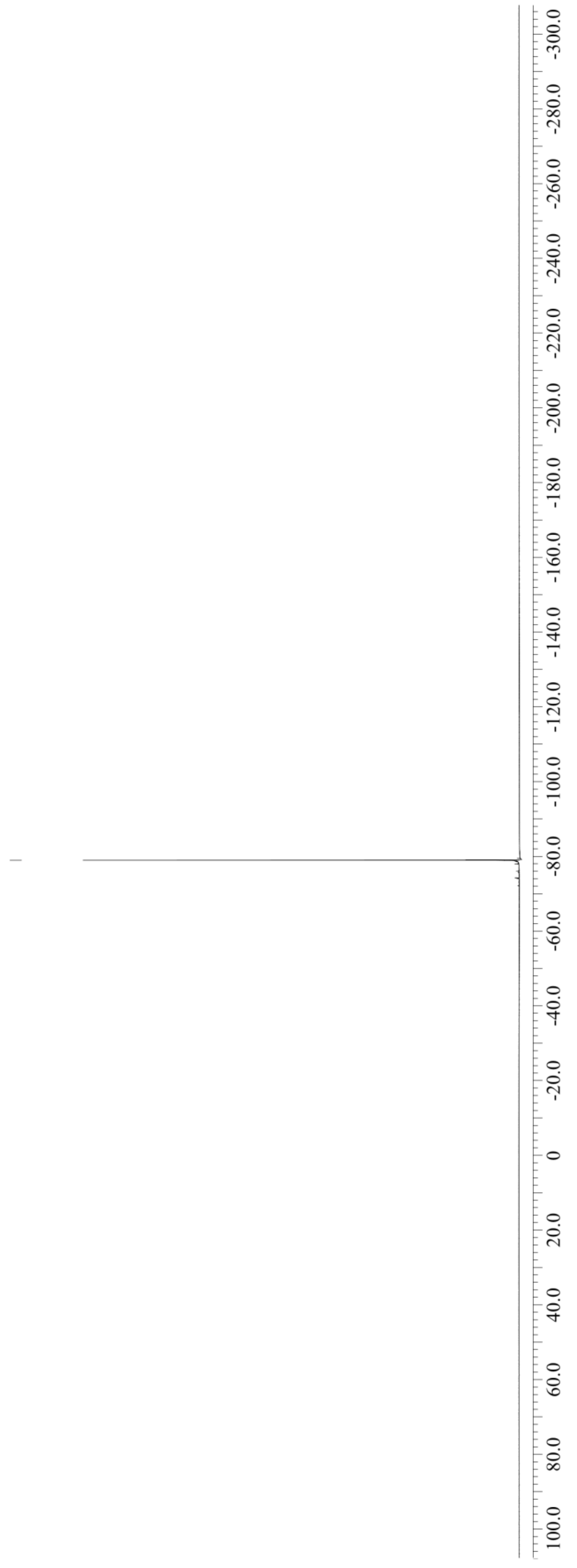

^{1}H NMR of **19** ($CDCl_3$, 400 MHz, 25 °C)

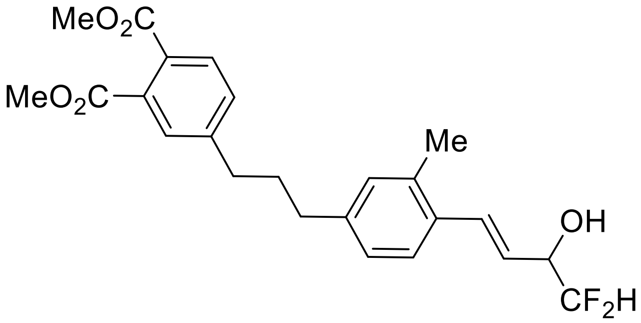

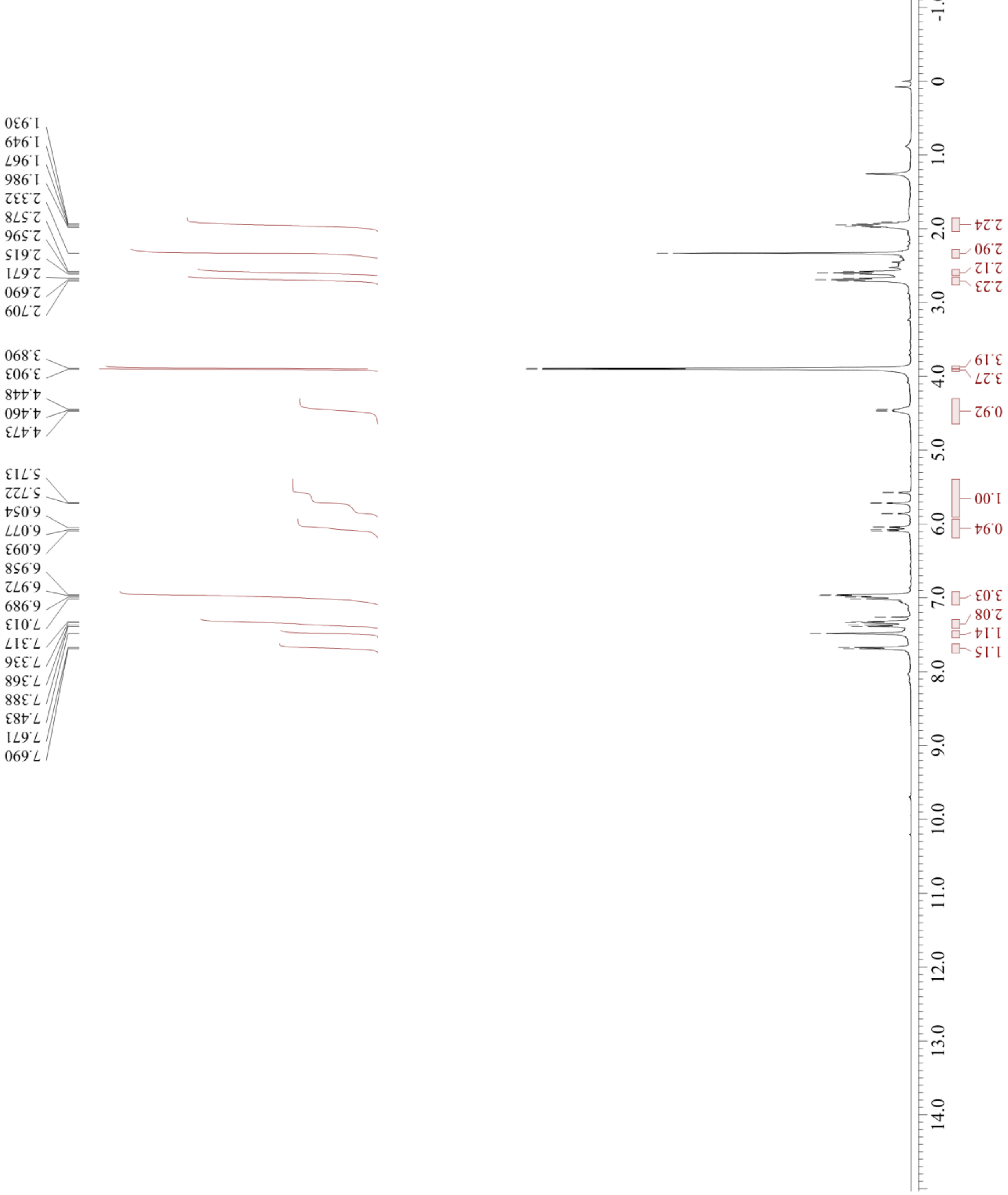

^{13}C NMR of **19**($CDCl_3$, 150 MHz, 25 °C)

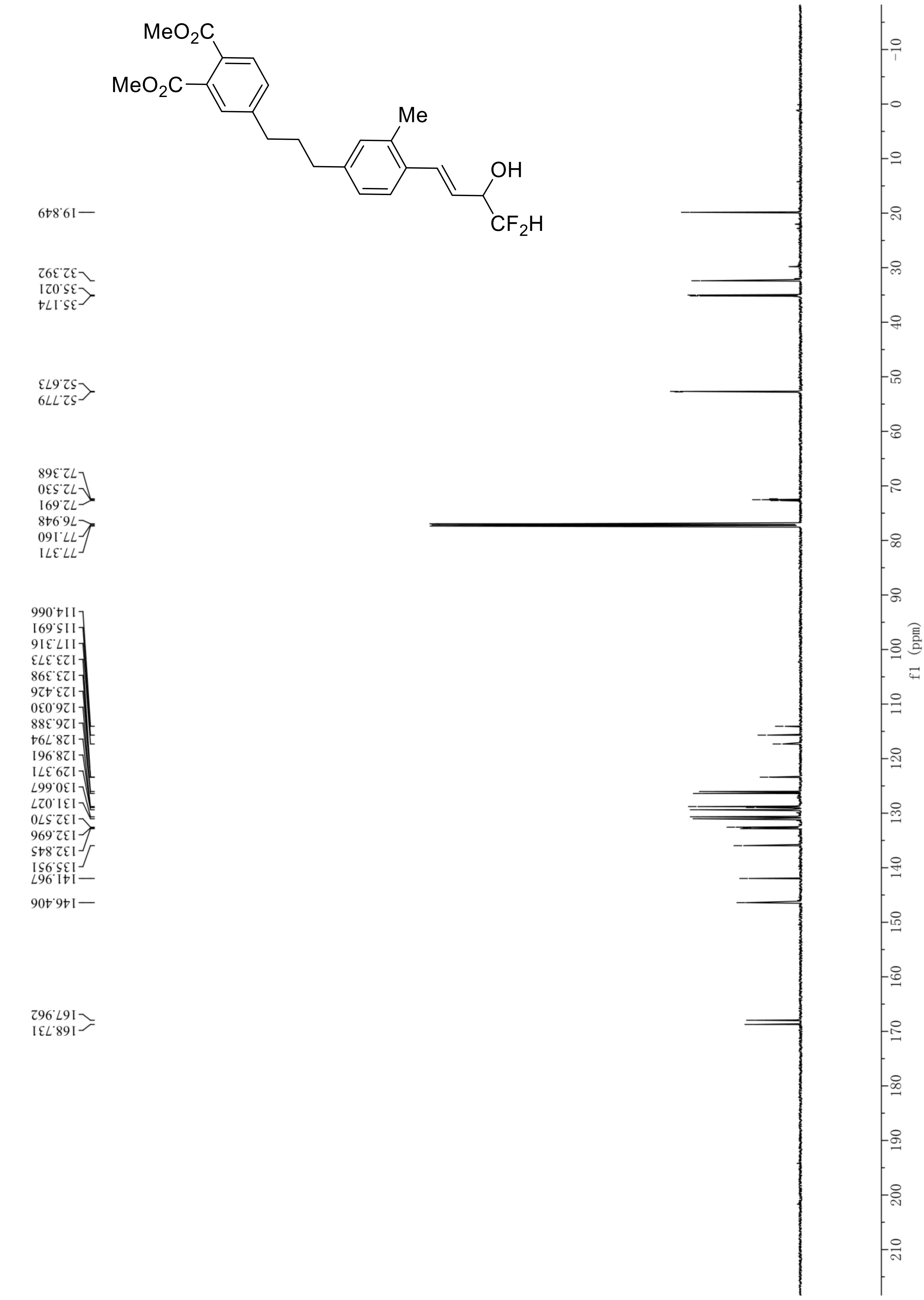

^{19}F NMR of **19** ($CDCl_3$, 375 MHz, 25 ºC)

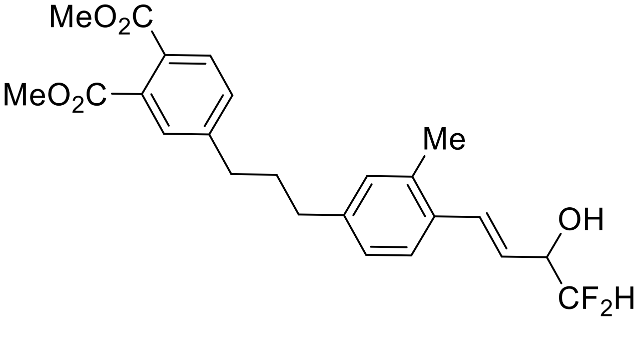

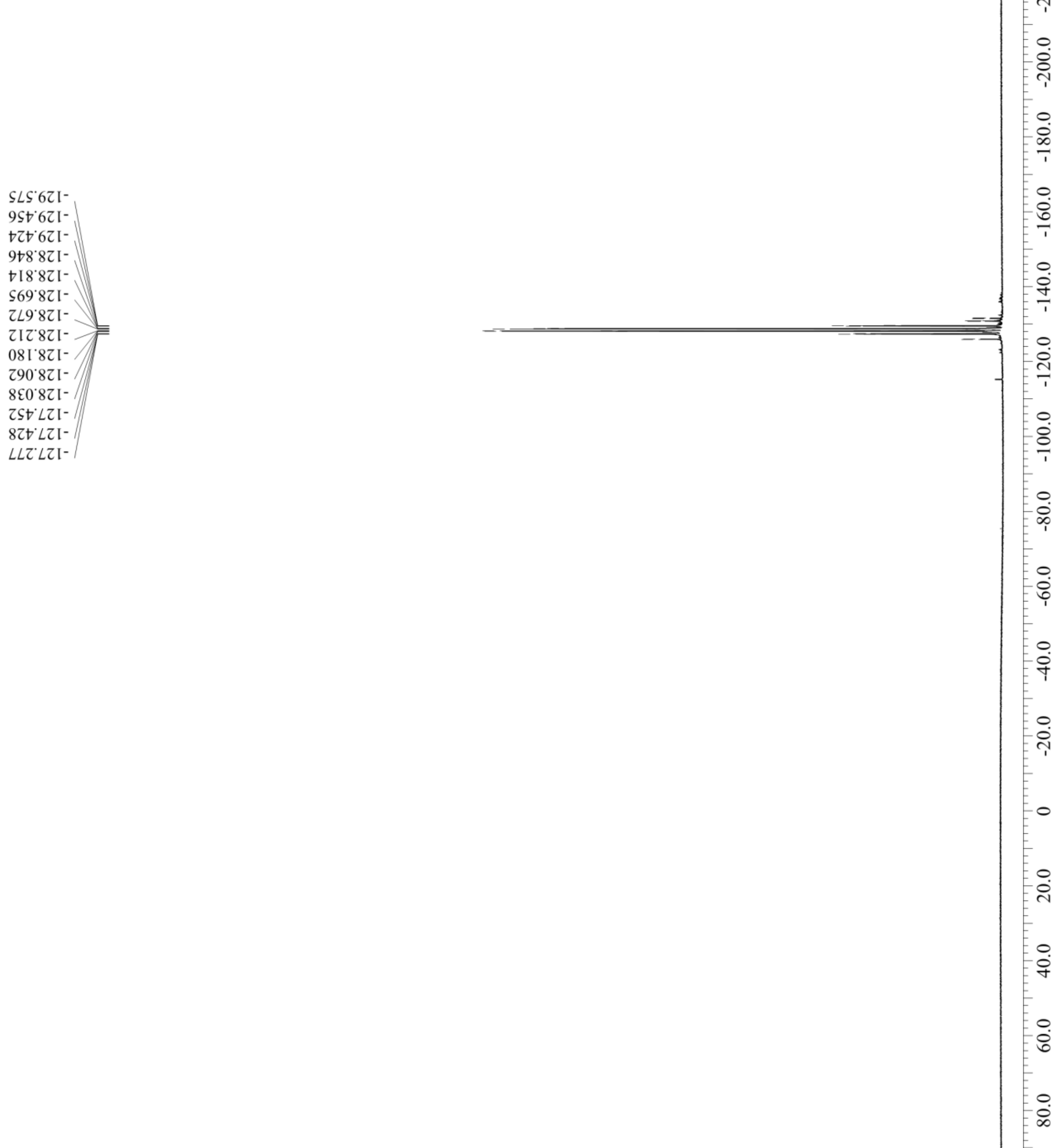

^{1}H NMR of **antitumor agent Z** ($CDCl_3$, 400 MHz, 25 ºC)

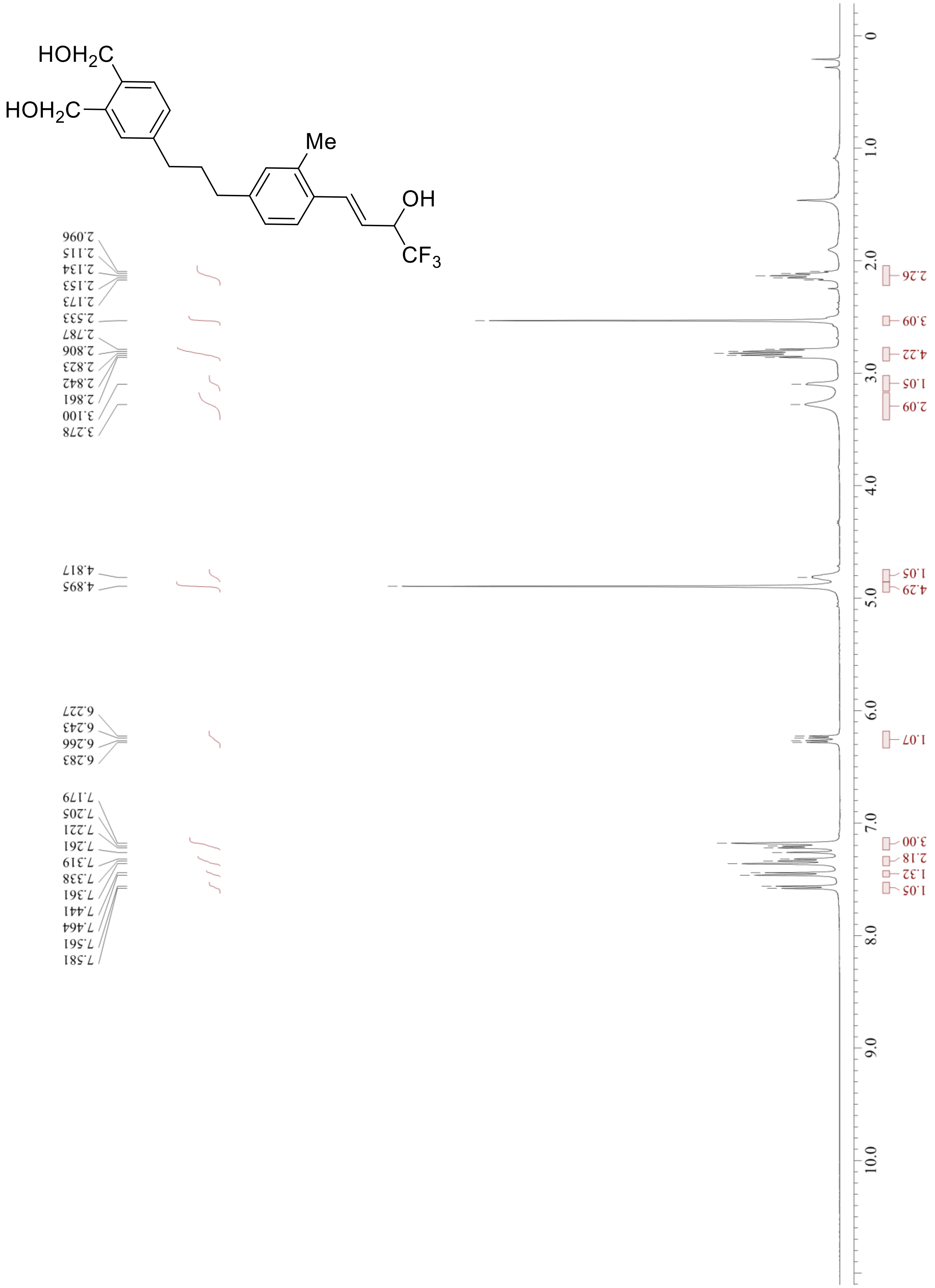

^{13}C NMR of **antitumor agent Z** ($CDCl_3$, 150 MHz, 25 ºC)

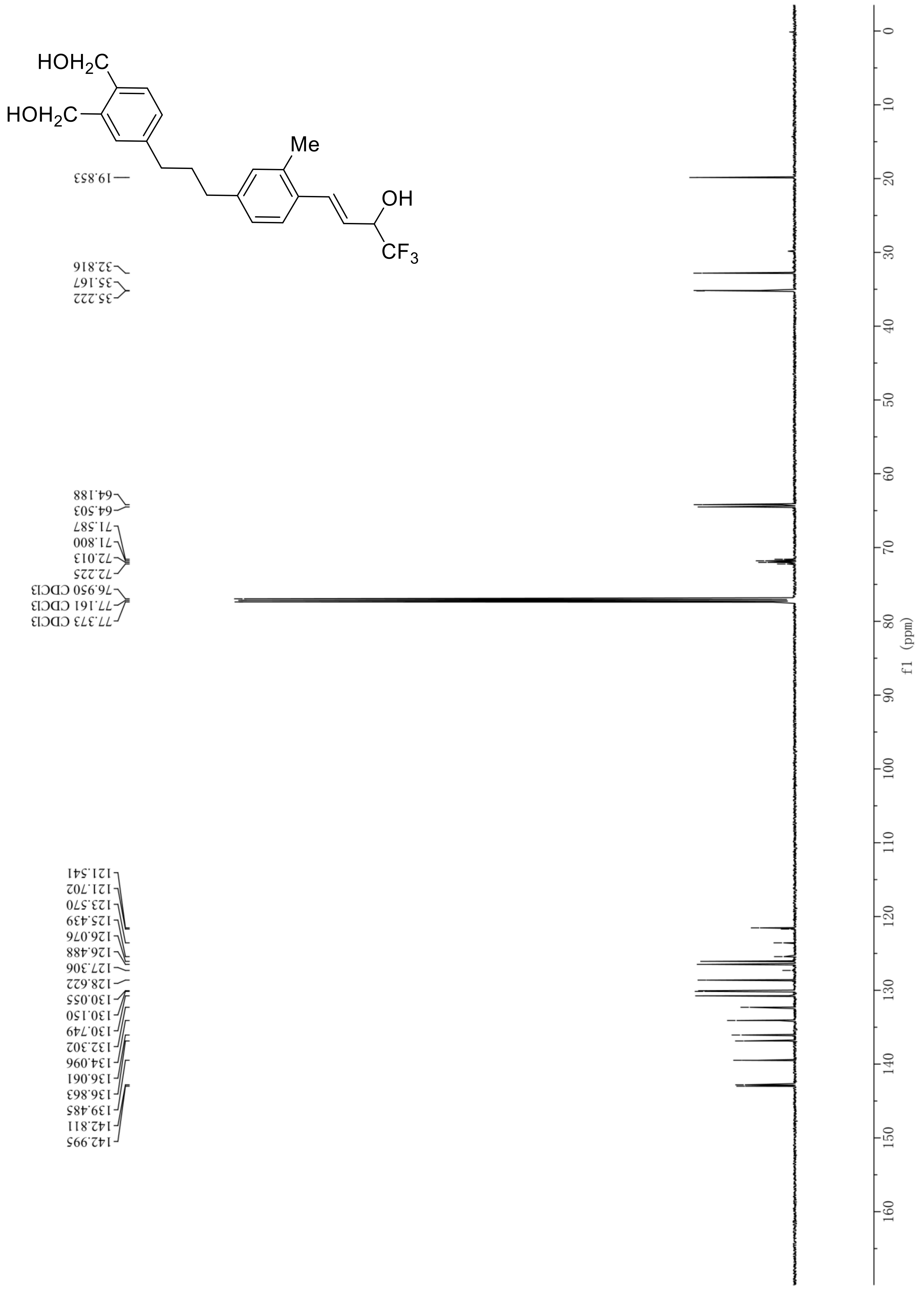

^{19}F NMR of **antitumor agent Z** ($CDCl_3$, 375 MHz, 25 ºC)

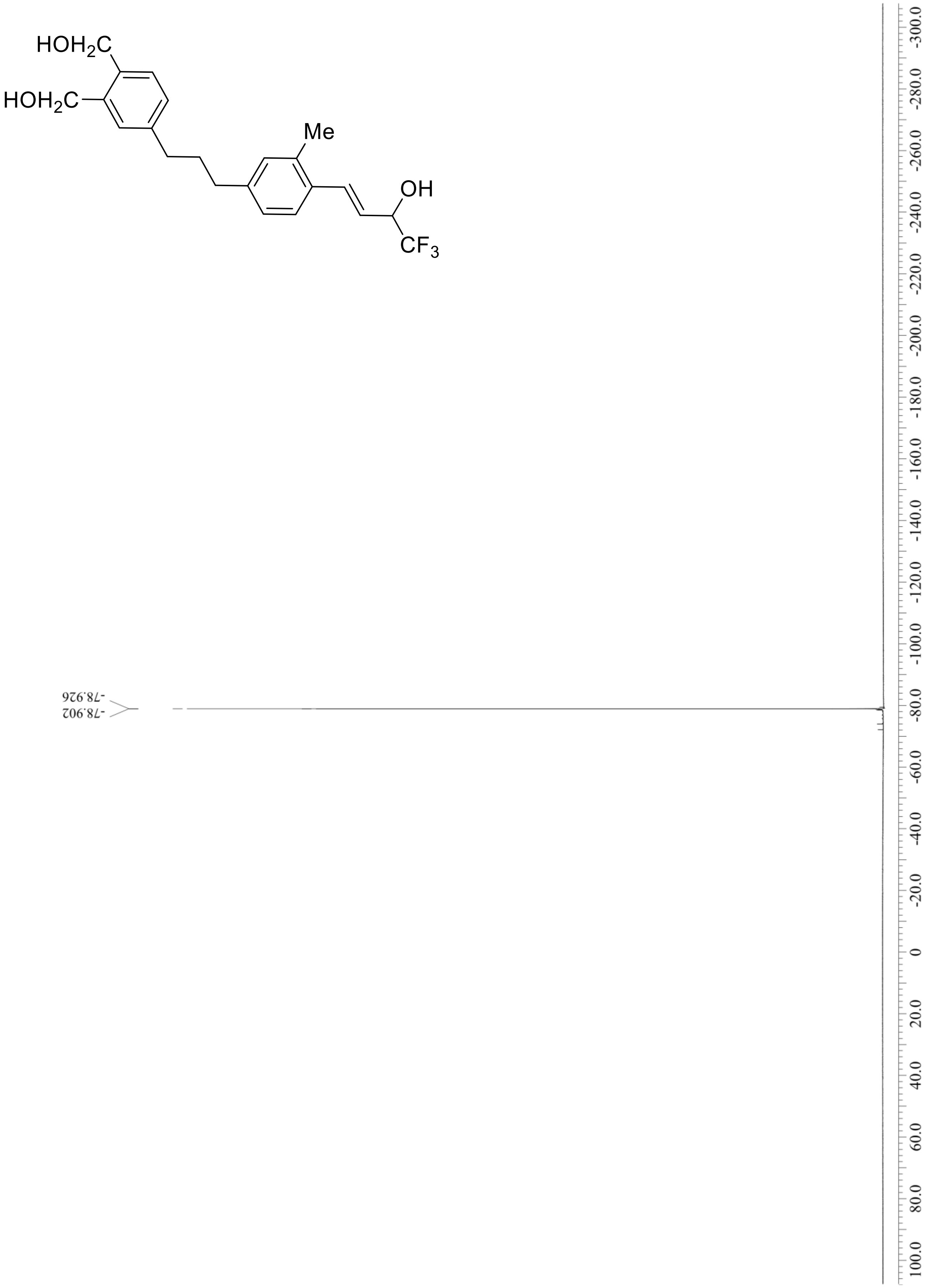

^{1}H NMR of **difluoro analog Z’ of antitumor agent Z** ($CDCl_3$, 400 MHz, 25 ºC)

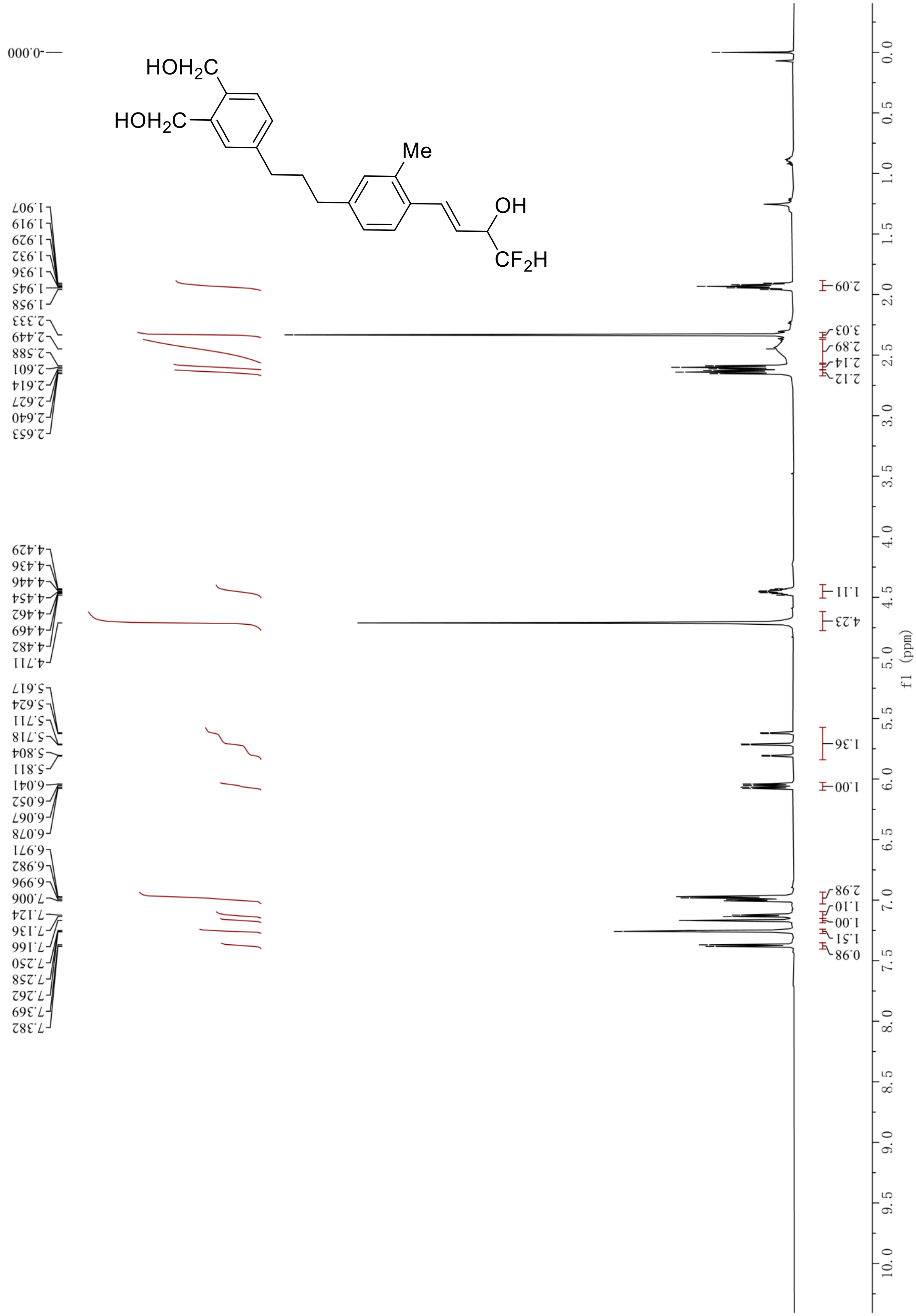

^{13}C NMR of **difluoro analog Z' of antitumor agent Z** ($CDCl_3$, 100 MHz, 25 ºC)

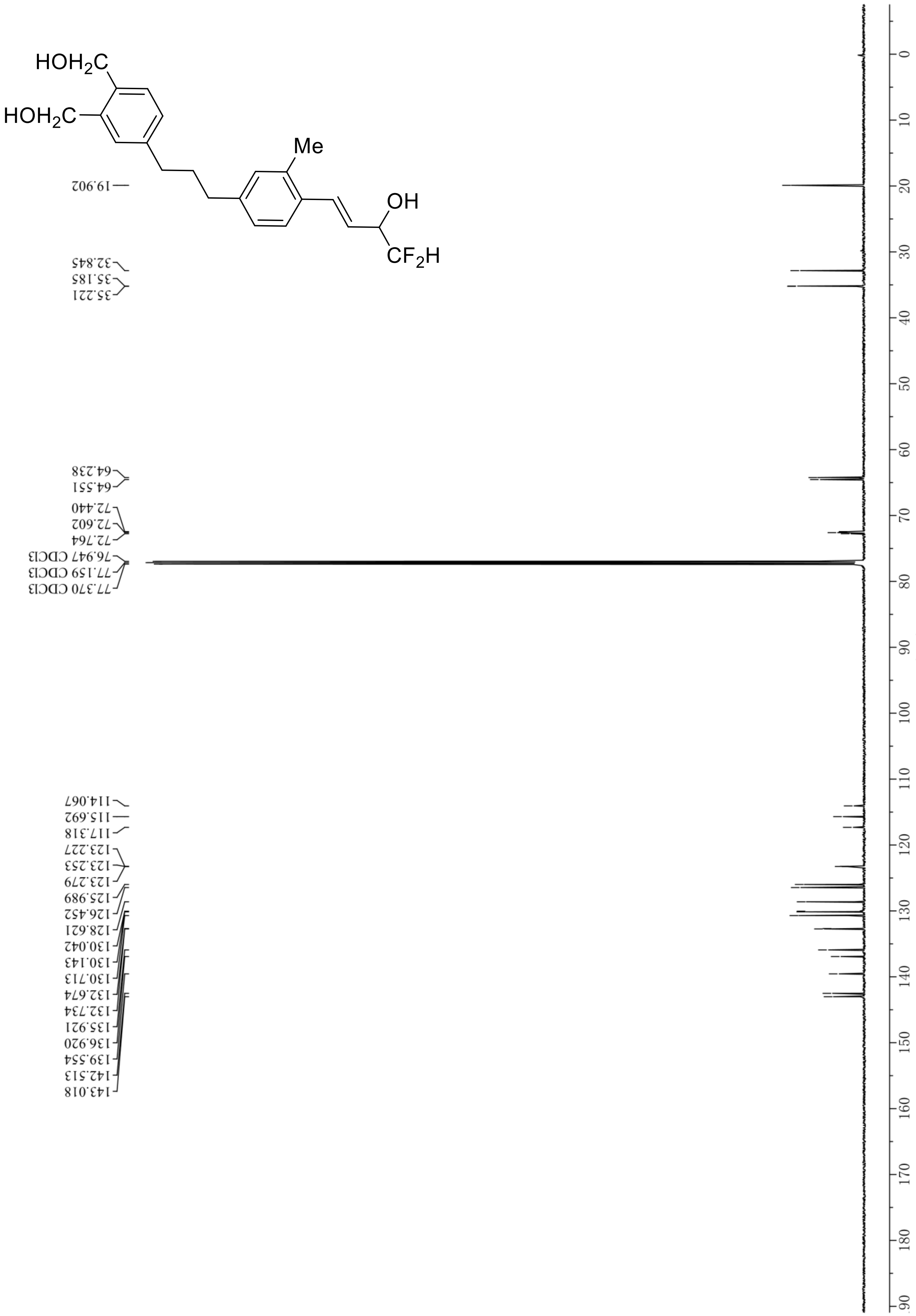

^{19}F NMR of **difluoro analog Z' of antitumor agent Z** (CDCl$_3$, 375 MHz, 25 ºC)

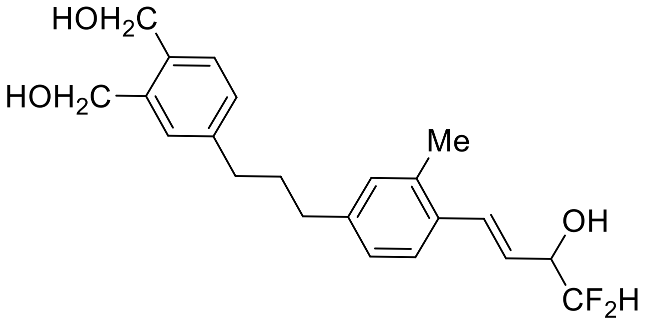

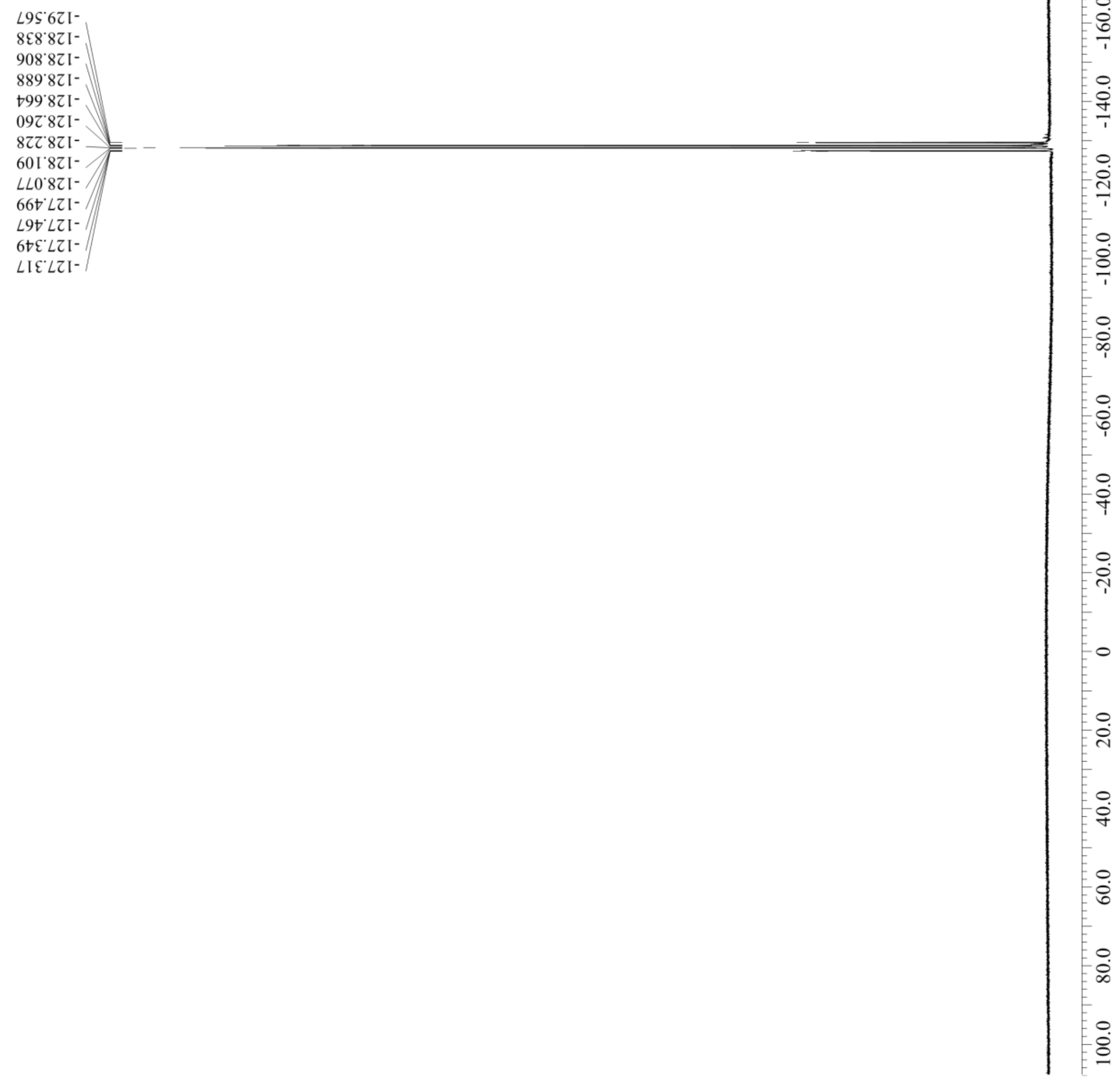

Supplementary References

[1] Chung, W. J. & Welch, J. T. A general method for synthesis of trifluoroacetyltrialkyl(aryl)silanes and the Sakurai reaction of fluorinated acylsilanes with allyl silanes. *J. Fluorine Chem.* **125**, 543–548 (2004).

[2] Xiao, F. Liu, C. Wang, D. Huang, H. & Deng, G -J. Concise synthesis of ketoallyl sulfones through an iron-catalyzed sequential four-component assembly. *Green Chem.* **20**, 973–977 (2018).

[3] Guo, H -M. Zhou, Q -Q. Jiang, X. Shi, D -Q. & Xiao, W -J. Catalyst- and Oxidant-Free Desulfonative C−P Couplings for the Synthesis of Phosphine Oxides and Phosphonates. *Adv. Synth. Catal.* **359**, 4141–4146 (2017).

[4] Jecs, E. & Diver, S. T. Toward the synthesis of amphidinolide P: optimization of a model ene–yne metathesis fragment coupling. *Tetrahedron Lett.* **55**, 4933–4937 (2014).

[5] Shrestha, T. B. Troyer, D. L. & Bossmann, S. H. Strategies for Large-Scale Synthesis of Coelenterazine for in Vivo Applications. *Synthesis,* **46**, 646–652 (2014).

[6] Trost, B. M. & Schmuff, N. R. Stereochemistry of allyl sulfones. On the structure of metalated allyl sulfones and their stereochemistry of alkylation. *J. Am. Chem. Soc.* **107**, 396–405 (1985).

[7] Paquette, L. A. Gilday, J. P. & Maynard, G. D. Intramolecular nucleophilic addition to unsaturated carbon. Dependence of cyclization efficiency on the method of carbon-carbon bond cleavage utilized to generate the reactive species. *J. Org. Chem*. **54**, 5044–5053 (1989).

[8] Geoghegan, K. Evans, P. Rozas, I. & Alkorta, I. Regioselectivity in the Intramolecular Heck Reaction of a Series of Cyclic Sulfonamides: An Experimental and Computational Study. *Chem. Eur., J.* **18**, 13379–13387 (2012).

[9] Larson, E. G. & Connell, G. Anisotropic Conductive Adhesive Compositions. *PCT Int. Appl.* 2008011452, **2008.**

[10] Yang, Z. & Tang, A. Synthesis of Perfluoroalkyl-Substituted Oxindoles through Organophotoredox-Catalyzed Perfluoroalkylation of N-arylacrylamides with Perfluoroalkyl Iodides. *Synlett* **30**, 1061–1066 (2019).

[11] Biadatti, T. Thoreau, E. Voegel, J. & Jomard, A. Analogues of Vitamin D. WO2004020379A1, (2004**)**

[12] Harrowven, D. C. et al. Potassium carbonate–silica: a highly effective stationary phase for the chromatographic removal of organotin impurities. *Chem. Commun.* **46**, 6335–6337 (2010).

[13] Wu, K., Wang, L., Colón-Rodríguez, S., Flechsig, G.-U. & Wang, T. Amidyl Radical Directed Remote Allylation of Unactivated sp3 C−H Bonds by Organic Photoredox Catalysis. *Angew. Chem. Int. Ed.* 58, 1774-1778 (2019).

[14] Zhang, J., Li, Y., Zhang, F., Hu, C.& Chen, Y. Generation of Alkoxyl Radicals by Photoredox Catalysis Enables Selective C(sp3)-H Functionalization under Mild Reaction Conditions. *Angew. Chem. Int. Ed.* 55, 1872-1875 (2016)

[15] Wei, W.-T. et al. Synthesis of Oxindoles by Iron-Catalyzed Oxidative 1,2-Alkylarylation of Activated Alkenes with an Aryl C(sp2)-H Bond and a C(sp3)-H Bond Adjacent to a Heteroatom. *Angew. Chem. Int. Ed.* 52, 3638-3641 (2013).

[16] Li, Y.-M. et. al. Direct Annulations toward Phosphorylated Oxindoles: Silver-Catalyzed Carbo-Phosphorus Functionalization of Alkenes. *Angew. Chem. Int. Ed*. 52, 3972-3976 (2013).

[17] Li, G. et. al. Nickel-Catalyzed Decarboxylative Difluoroalkylation of α,β-Unsaturated Carboxylic Acids. *Angew. Chem. Int. Ed.* 55, 3491-3495 (2016).

[18] Cui, Z., Shang, X., Shao, X.-F. & Liu, Z.-Q. Copper-catalyzed decarboxylative alkenylation of sp3 C–H bonds with cinnamic acids via a radical process. *Chem. Sci.* 3, 2853-2858 (2012)
